# A general photoinduced manganese-catalyzed platform for the sequential difunctionalization of [1.1.1]propellane

**DOI:** 10.1126/sciadv.aeg5293

**Published:** 2026-06-26

**Authors:** Jun Xu, Lin Li, Qing Pang, Manyi Chen, Huamin Wang, Pengfei Zhang

**Affiliations:** ^1^ College of Material, Chemistry and Chemical Engineering, Key Laboratory of Organosilicon Chemistry and Material Technology, Ministry of Education, Zhejiang Key Laboratory of Organosilicon Material Technology, Hangzhou Normal University, Hangzhou 311121, China.; ^2^ Department of Chemistry, Zhejiang University, Hangzhou 310027, China.

## Abstract

Bicyclo[1.1.1]pentanes (BCPs) constitute prominent bioisosteric replacements for aromatic frameworks and are frequently incorporated into diverse pharmaceutical compounds. However, current synthetic routes, especially those for the synthesis of sulfonyl BCP derivatives, are hampered by narrow substrate scope and limited functional group tolerance. These constraints underscore the demand for more versatile and operationally practical synthetic strategies. Here, we disclose a general photoinduced manganese-catalyzed platform for the sequential difunctionalization of [1.1.1]propellane, enabling the modular synthesis of multifunctional sulfonyl BCPs. This platform facilitates the construction of diverse sulfonyl-BCP derivatives, including ─Cl, ─Br, ─I, ─CN, ─SCD_3_, ─H, ─HetAr, ─COAr, ─SAr, ─N═NAr, etc., with exceptional functional group tolerance and scalability. The utility of this approach is further enhanced by its applicability to late-stage functionalization of pharmacologically active compounds and derivative synthesis. Mechanistic investigations and DFT calculation revealed that this work involves the ligand-accelerated manganese-catalyzed halogen-atom transfer (XAT) radical addition process.

## Teaser

A manganese photocatalyst unlocks modular access to diverse sulfonyl BCP building blocks for drug discovery.

## INTRODUCTION

**Fig. 1.**
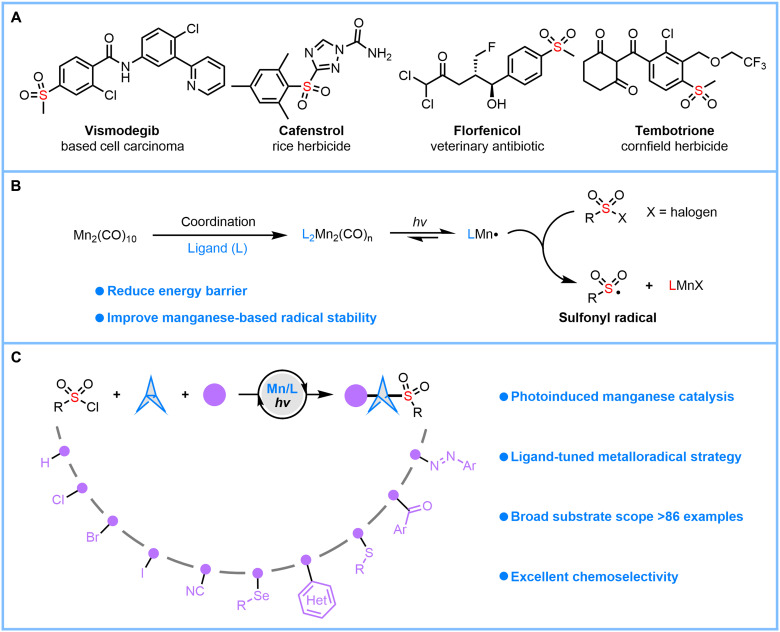
Current research status and our reaction design. (**A**) Pharmaceutical molecules containing sulfonyl groups. (**B**) Ligand-tuned manganese catalysis for sulfonyl radical initiation. (**C**) This work: modular access to diverse sulfonyl BCPs.

The development of straightforward and efficient synthetic routes to access bioisosteric scaffolds has drawn considerable interest, because these scaffolds are critical for enhancing bioavailability and metabolic stability in drug discovery programs (*1*–*7*). Among the various three-dimensional, aliphatic frameworks investigated over the past decade, bicyclo[1.1.1]pentanes (BCPs) have emerged as premier bioisosteres for *para*-substituted aromatic rings and are now ubiquitous motifs in medicinal chemistry (*8*–*24*). Aryl sulfones are privileged framework found in many marketed drugs (Fig. 1A) (*25*–*29*). Replacing their aryl cores with C(*sp*^3^)-rich BCP units could potentially increase three-dimensionality and metabolic robustness (*30*), yet general and highly regioselective access to sulfonyl-functionalized BCPs, especially disubstituted variants, remains scarce.

Conventional synthetic routes to sulfonyl-functionalized BCPs are hampered by substantial practical limitations, which severely restrict access to these valuable scaffolds (*31*). Access to monosubstituted sulfonyl BCPs typically relies on a tedious and unsustainable two-step sequence, a process plagued by inefficiency and the need for hazardous oxidants. The synthesis of disubstituted sulfonyl BCP derivatives presents a greater challenge, yet holds notable importance. Existing strategies to construct these architectures frequently depend on the addition of bespoke bifunctional reagents to [1.1.1]propellane (*32*, *33*). While effective in specific cases, this approach is inherently limited by the structural constraints of the bifunctional reagents used (*34*), which consequently restricts the structural diversity of accessible sulfonyl BCPs. In contrast, multicomponent radical sulfonylation holds promise as a modular strategy for the direct assembly of diverse sulfonyl BCPs from readily available starting materials. However, its development is impeded by two critical shortcomings (*35*–*37*). Practically, these methods often depend on costly metal photocatalysts or stoichiometric sacrificial agents, affecting their sustainability profile. Mechanistically, the reliance on a single-electron transfer pathway to generate electrophilic sulfonyl radicals (RSO_2_·) introduces inherent risks of overoxidation or unselective electron transfer, often leading to competitive side reactions. This difficulty in controlling radical reactivity and selectivity represents a long-standing challenge, limiting the modular construction of complex targets like sulfonyl BCPs.

In this context, halogen-atom transfer (XAT) offers a complementary mechanistic approach (*38*–*40*). By mediating direct homolytic halogen abstraction via a radical “abstractor,” XAT enables precise and selective radical generation under mild conditions, circumventing the need for strong redox systems. Notably, visible light–driven manganese catalysis, particularly with Mn_2_(CO)_10_, has emerged as a sustainable platform for XAT, where photolysis of the Mn─Mn bond efficiently generates carbon radicals from organic halides for C─C bond formation (*41*–*52*). However, the limited efficiency and narrow polarity tolerance of the resulting Mn(CO)_5_· radical constrain its utility in complex synthetic settings. We recognized that to harness this platform for the challenging synthesis of disubstituted sulfonyl BCPs, a strategy to overcome these intrinsic limitations was required. According to coordination chemistry, ligands can fine-tune the properties of metal centers by altering electron density, steric environment, and redox potentials (*53*–*55*). Guided by this principle, we reasoned that strategic ligand coordination could profoundly remodel the reactivity of such metalloradicals, specifically enhancing its XAT efficiency toward sulfonyl chlorides and suppressing unproductive pathways. Inspired by prior work and our interest in strain-release functionalization (*56*–*58*), we thus envisioned a ligand-enhanced manganese catalytic system that could simultaneously address sustainability and the critical challenge of radical control. By tuning the coordination environment of the manganese catalyst, we aimed to achieve the efficient and selective activation of sulfonyl chlorides to generate sulfonyl radicals (Fig. 1B), which would first add to [1.1.1]propellane. The resulting BCP radical could then be intercepted by a diverse array of radical acceptors, enabling a modular, multicomponent synthesis of valuable disubstituted sulfonyl BCP derivatives. Here, we report a visible light–induced, ligand-enhanced, manganese-catalyzed platform for the sequential difunctionalization of [1.1.1]propellane (Fig. 1C). This work establishes (i) exceptional substrate generality; (ii) a ligand-accelerated, manganese-catalyzed XAT process that efficiently replaces conventional SET-based systems; and (iii) a solution to control sulfonyl radicals for selective difunctionalization.

## RESULTS

### Reaction optimization

**Fig. 2.**
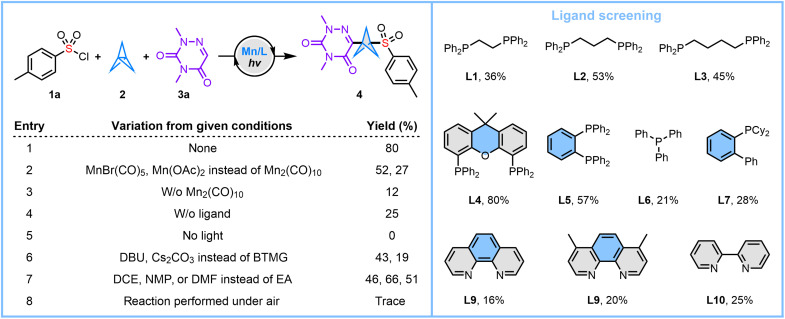
Reaction optimization. Reaction conditions: **1a** (1.5 equiv), **2** (1 equiv), **3a** (0.2 mmol), Mn_2_(CO)_10_ (5 mol %), L4 (10 mol %), BTMG (2 equiv), EA (2.0 ml), blue LEDs, room temperature (rt), N_2_, 12 hours, isolated yields.

In initial exploration of the manganese-catalyzed difunctionalization of [1.1.1]propellane (**2**) with 4-methylbenzenesulfonyl chloride (**1a**) and azauracil (**3a**) under blue light-emitting diode (LED) irradiation, the model reaction using Mn_2_(CO)_10_ as the catalyst, 1,2-bis(diphenylphosphaneyl)ethane (**L1**) as the ligand, BTMG (2-tert-butyl-1,1,3,3-tetramethylguanidine) as the base, and ethyl acetate (EA) as the solvent afforded the target sulfonyl BCP-heteroarene (**4**) in 36% yield. This outcome prompted a systematic evaluation of reaction parameters, with a ligand structure emerging as a critical determinant of efficiency. Screening of various phosphine and nitrogen-based ligands revealed the clear superiority of phosphine donors. Among them, xantphos (**L4**) proved optimal, delivering product (**3**) in 80% yield (Fig. 2, entry 1), an effect attributed to its balanced steric and electronic properties. Catalyst evaluation indicated that Mn_2_(CO)_10_ outperformed MnBr(CO)_5_ (52%) and Mn(OAc)_2_ (27%) (entry 2). Control experiments confirmed the indispensability of the manganese catalyst, phosphine ligand, and visible light irradiation for achieving high conversion (entries 3 to 5). It is worth noting that even in the absence of the Mn_2_(CO)_10_ catalyst, the desired product (**3**) was obtained in 12% yield. This may be attributed to the formation of a photoactive electron donor-acceptor (EDA) complex between the sulfonyl chloride and the nitrogen-containing compound (*59*). The product yield decreased when 1,8-diazabicyclo[5.4.0]undec-7-ene (DBU) or Cs_2_CO_3_ was used as base instead of BTMG (entry 6). Solvent screening identified EA as superior to alternatives such as 1,2-dichloroethane (DCE), *N*-methylpyrrolidone (NMP), and dimethyl formamide (DMF) (entry 7). In addition, we have also explored the use of mixed solvents during reaction optimization (table S1). Specifically, five different mixed solvent systems (EA/DCE, EA/NMP, EA/DMF, EA/acetone, and EA/tetrahydrofuran) were evaluated. Among these, EA/NMP (v/v = 1/1) afforded the highest yield of 72%, which was still inferior to that achieved with EA as the sole solvent. In addition to 455-nm LED irradiation, we examined the reaction at 365, 405, 520, and 660 nm (table S2). The reaction occurred at 365 to 520 nm, all inferior to that at 455 nm, whereas no product was detected at 660 nm. Notably, the reaction was entirely suppressed under aerobic conditions, likely due to quenching of the photoexcited catalyst by oxygen (entry 8) (*60*).

### Reaction substrate scope

**Fig. 3.**
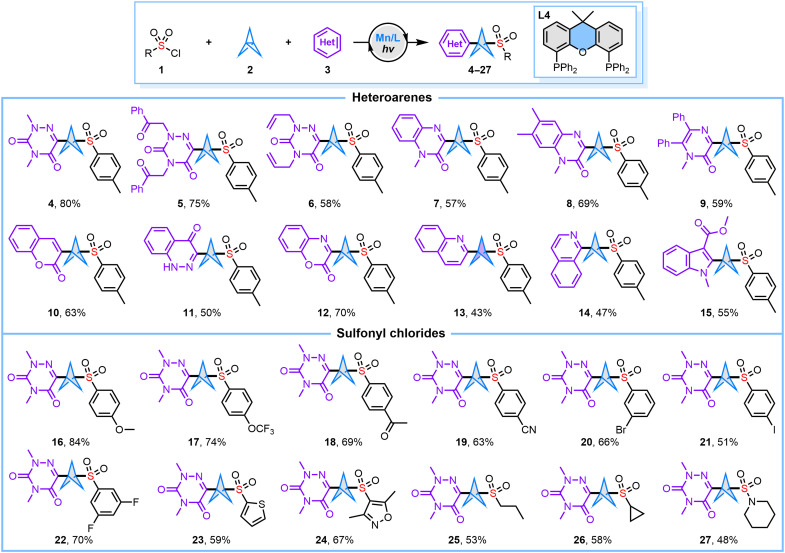
Substrate scope of heterarenes and sulfonyl chlorides. Reaction conditions: **1** (1.5 equiv), **2** (1 equiv), **3** (0.2 mmol), Mn_2_(CO)_10_ (5 mol %), L4 (10 mol %), BTMG (2 equiv), EA (2.0 ml), blue LEDs, rt, N_2_, 12 hours, isolated yields.

With the optimized conditions in hand, we systematically examined the substrate scope of sulfonyl chlorides (**1**) and heteroarenes (**3**) in the photoinduced manganese-catalyzed difunctionalization of [1.1.1]propellane (Fig. 3). Given the prevalence of azauracil as a privileged scaffold in pharmaceuticals and natural products (*61*, *62*), our initial efforts focused on the incorporation of sulfonyl BCP motifs into azauracil derivatives. Substrates bearing different *N,N′*-substituents, including methyl and ketone groups, underwent smooth conversion to afford the corresponding products (**4** and **5**) in 75 to 80% yields. Notably, an azauracil functionalized with an unsaturated alkenyl group also participated effectively in the transformation, yielding product (**6**) in 58% yield. To further broaden the scope of compatible heteroarenes, we examined a diverse array of heterocyclic systems, such as quinoxalin-2(1*H*)-one, pyrazinone, coumarin, cinnolin-4(1*H*)-one, oxazinone, quinoline, isoquinoline, and benzopyrole. Under the standard reaction conditions, these substrates successfully coupled with sulfonyl BCP units via the manganese-catalyzed multicomponent process, leading to the formation of diverse sulfonyl BCP-heteroarenes (**7** to **15**) in 43 to 70% yields. We next turned our attention to the variation of sulfonyl chlorides. A range of substituted benzenesulfonyl chlorides bearing either electron-donating or electron-withdrawing groups reacted efficiently with [1.1.1]propellane (**2**) and azauracil (**3a**), delivering the corresponding adducts (**16** to **19**) in 63 to 84% yields. Halogenated sulfonyl chlorides, which provide useful handles for further derivatization, were also compatible, affording products (**20** to **22**) in 51 to 70% yields. The structures of the products (**20** and **22**) were confirmed by x-ray crystallographic analysis [Cambridge Crystallographic Data Centre (CCDC) 2544084 and 2544085], as shown in the Supplementary Materials. The diminished yield observed for product (**21**) may be attributed to competing side pathways. Heteroaryl sulfonyl chlorides also performed well under the catalytic system, furnishing derivatives (**23** and **24**) in moderate yields. Alkylsulfonyl chlorides, which would generate more unstable sulfonyl radical, also participated in the transformation, providing the desired products (**25** to **27**) in 48 to 58% yields.

**Fig. 4.**
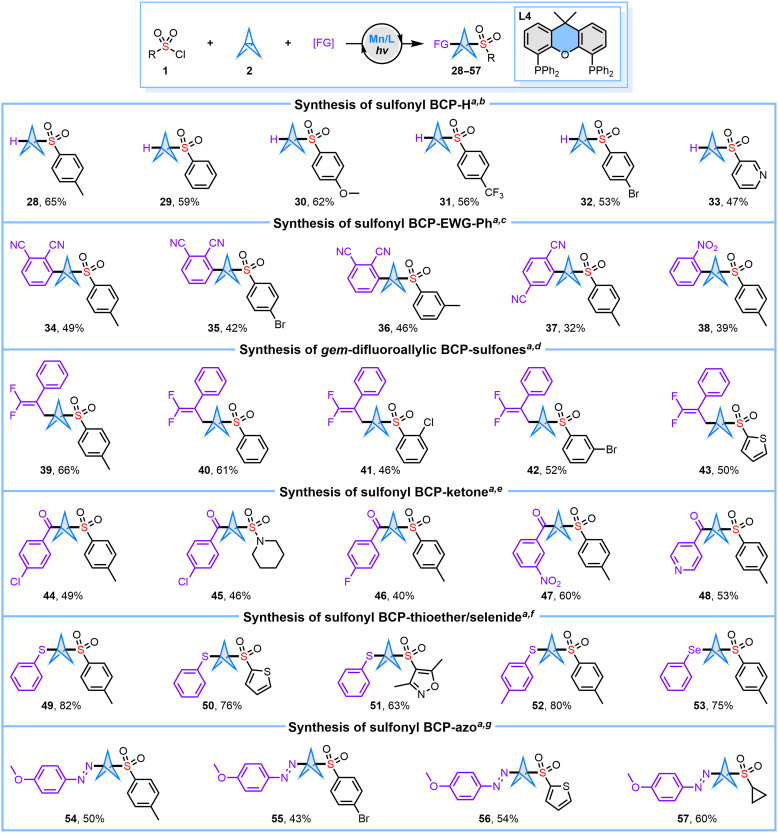
Investigation of diverse radical acceptors for multicomponent reactions. (a) Reaction conditions: **1** (1.5 equiv), **2** (1 equiv), Mn_2_(CO)_10_ (5 mol %), L4 (10 mol %), EA (2.0 ml), blue LEDs, rt, N_2_, 12 hours, isolated yields. (b) Et_3_SiH (0.2 mmol), NMP instead of EA. (c) EWG-Ph (0.2 mmol), BTMG (2 equiv). (d) (3,3,3-Trifluoroprop-1-en-2-yl)benzene (0.2 mmol), NMP instead of EA, 16 hours. (e) Aromatic aldehyde (0.2 mmol), BTMG (2 equiv). (f) Disulfide/diselenide (0.2 mmol), 6 hours. (g) 4-Methoxybenzenediazonium tetrafluoroborate (2 equiv), Cs_2_CO_3_ (2 equiv), acetonitrile instead of EA. FG, functional group.

Encouraged by the performance of above multicomponent transformations and considering that multicomponent reactions for synthesizing sulfonyl BCPs have rarely been explored (*63*), we envisioned that introducing a variety of different types of third components could provide a manganese-catalyzed platform for markedly expanding the applicability of this method. As shown in Fig. 4, it was found that blue LED light irradiation of a NMP solution of sulfonyl chloride (**1**) and [1.1.1]propellane (**2**) in the presence of Mn_2_(CO)_10_ as the catalyst and Et_3_SiH as the hydrogen donor furnished the target products (**28** to **33**) in 47 to 65% yields. In addition, the multicomponent radical sulfonylarylation of [1.1.1]propellane toward the synthesis of sulfonyl BCP-aryls (**34** to **38**) was also realized by the use of electron-deficient arenes, such as aryl cyanide and nitrobenzene, as aryl sources. Of note, the utilization of electron-deficient arenes as aryl precursors not only lowers the kinetic barrier for the arylation of nucleophilic alkyl radicals but also concomitantly suppresses the competing electrophilic sulfonylation pathway, thereby enabling the observed site-selective transformation. Besides, under standard conditions, replacing the solvent with NMP and extending the reaction time to 16 hours, the reaction system was proven effective for the *gem*-difluoroallylation of [1.1.1]propellane, affording a range of *gem*-difluoroallylic BCP-sulfones (**39** to **43**) in 46 to 66% yields. This method involves the addition of in situ–generated sulfonyl radicals to [1.1.1]propellane, forming key BCP radical intermediates. Subsequent trapping of these radicals by α-trifluoromethyl alkenes delivers the final products, demonstrating a versatile pathway for installing *gem*-difluoroallylic motifs onto the BCP scaffold. The synthetic value of this photoinduced manganese-catalyzed radical multicomponent coupling system was further demonstrated by the successful application in the efficient preparation of various sulfonyl BCPs, including sulfonyl BCP-ketones (**44** to **48**), sulfonyl BCP-thioether/selenide (**49** to **53**), and sulfonyl BCP-azo (**54** to **57**). The ability to access such a range of chemotypes further attests to the broad applicability and robustness of this methodology.

**Fig. 5.**
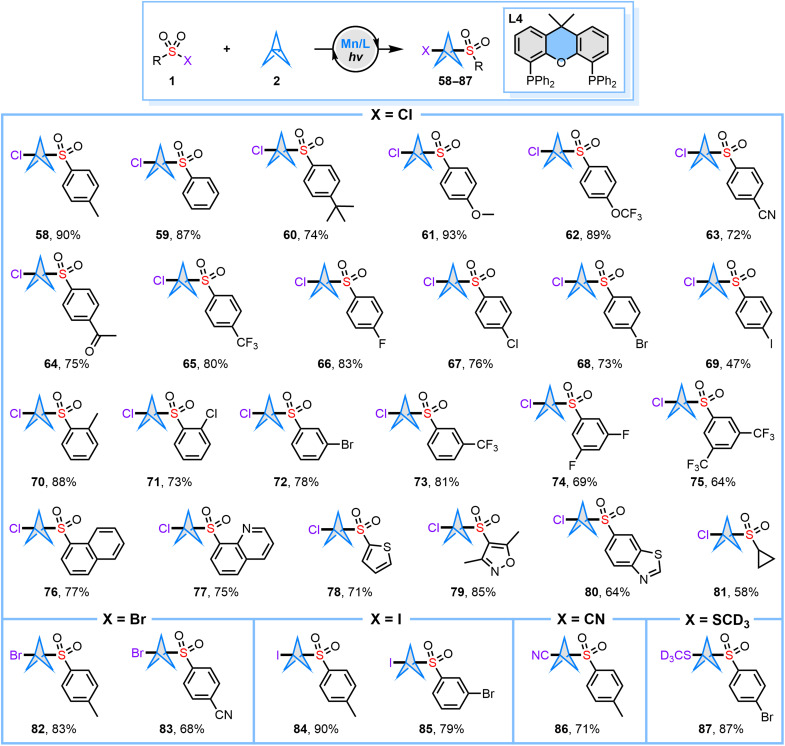
Substrate scope of sulfonyl chlorides for two-component reactions. Reaction conditions: **1** (1.5 equiv), **2** (0.2 mmol), Mn_2_(CO)_10_ (5 mol %), L4 (10 mol %), EA (2.0 ml), blue LEDs, rt, N_2_, 12 hours, isolated yields are given.

Unexpectedly, even in the absence of the third component, the reaction proceeded efficiently. Under the standard conditions, sulfonyl chloride (**1**) acted as a bifunctional reagent and reacted smoothly with [1.1.1]propellane (**2**), yielding sulfonyl BCP-Cl with high efficiency. As shown in Fig. 5, diverse *para*-substituted benzenesulfonyl chlorides, which bearing electron-donating (**58** and **60** to **62**), electron-neutral (**59**), or electron-withdrawing groups (**63** to **65**), could react smoothly with [1.1.1]propellane, affording the corresponding products in 72 to 93% yields. *Para*-halogenated substrates, which offer handles for further functionalization, were also tolerated, delivering products (**66** to **69**) in 47 to 83% yields, although product (**69**) was obtained in a lower yield (47%) likely due to competing side reactions. The reaction efficiency was largely unaffected by the substituent position, as shown by products (**70** to **73**). Substrates featuring additional *meta*–electron-withdrawing groups, such as 3,5-difluoro and 3,5-bis(trifluoromethyl) benzenesulfonyl chlorides, furnished corresponding products (**74** and **75**) in 64 to 69% yields. An extended aromatic system, naphthalene-2-sulfonyl chloride, also performed well, yielding product (**76**) efficiently. Heteroaryl sulfonyl chlorides were likewise suitable substrates, providing derivatives (**77** to **80**) in 64 to 85% yields. Notably, alkylsulfonyl chlorides, which generally more challenging than (hetero)aryl analogs, could furnish target product (**81**) in an acceptable yield. Inspired by the successful difunctionalization of [1.1.1]propellane using sulfonyl chloride as bifunctional reagent, other sulfonyl sources, including sulfonyl bromide, sulfonyl iodide, sulfonyl cyanide, and thiosulfonate, were further explored. Under the standard conditions, sulfonyl BCP derivatives (**82** to **87**) contain ─Br, ─I, ─CN, as well as ─SCD_3_ moieties could be obtained in 68 to 90% yields.

### Late-stage modification and product derivatization

**Fig. 6.**
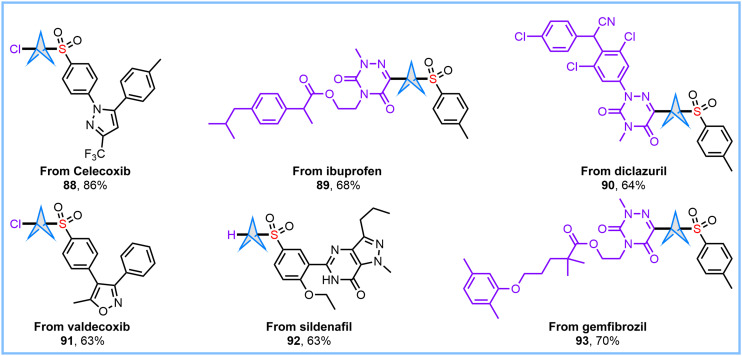
Late-stage functionalization of valuable molecules.

Having the practical protocol toward sulfonyl BCP derivatives in hand, we subjected a panel of marketed drugs and natural products to late-stage BCP grafting. As summarized in Fig. 6, celecoxib, ibuprofen, diclazuril, valdecoxib, sildenafil, and gemfibrozil were smoothly converted to the corresponding sulfonyl BCP conjugates (**88** to **93**) in moderate to good yields, demonstrating that the radical manifold is sufficiently mild to install the sulfonyl BCP skeleton into drug scaffolds.

**Fig. 7.**
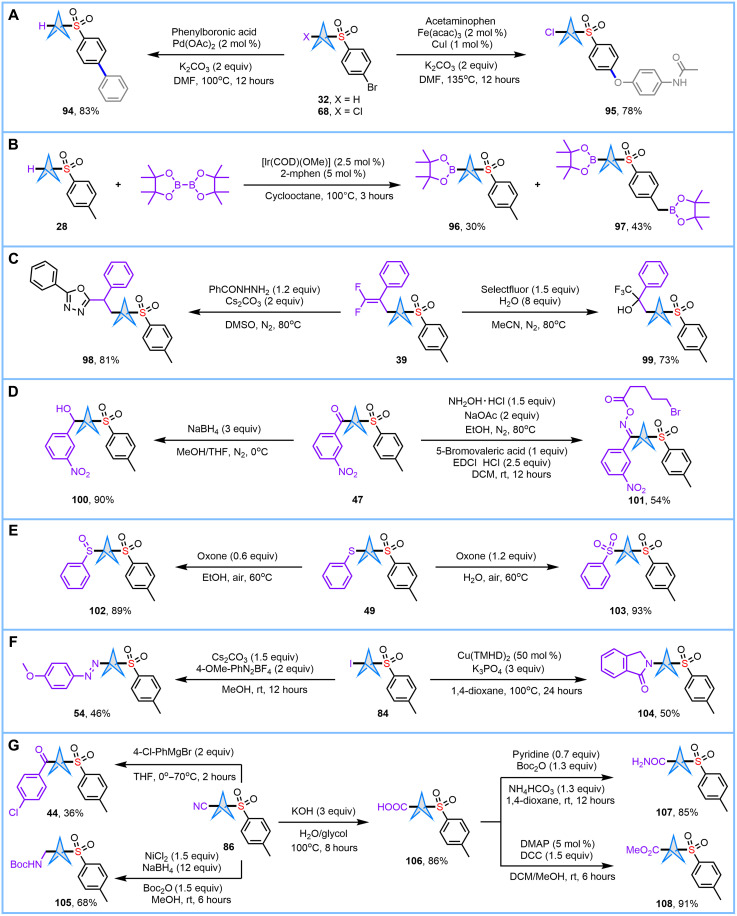
Derivatization of sulfonyl BCPs. (**A**) Late-stage functionalization of sulfonyl skeleton. (**B**) C─H boronation of sulfonyl BCP-H. (**C**) Derivatization of difluoralkene skeleton. (**D**) Further transformation of ketone group. (**E**) Selective oxidation of thioether group. (**F**) Dehalogenation reactions of sulfonyl BCP-I. (**G**) Synthesis of diverse BCPs from sulfonyl BCP-CN. DMSO, dimethyl sulfoxide; MeCN, acetonitrile; MeOH, methanol; THF, tetrahydrofuran; EDCI, 1-ethyl-3-(3-dimethyllaminopropyl)carbodiimide hydrochloride; DMAP, dimethylaminopyridine; DCC, dicyclohexylcarbodiimide; DCM, dichloromethane.

To further demonstrate the versatility of the sulfonyl BCP platform in medicinal chemistry, we performed a concise series of downstream transformations (Fig. 7). Suzuki-Miyaura cross-coupling of sulfonyl-BCP-H with phenylboronic acid afforded adduct (**94**) in 83% yield. Chemoselective iron-promoted C─O coupling with acetaminophen occurred preferentially at the C(*sp*^2^)─Br bond, transferring the sulfonyl BCP unit to the drug scaffold to give conjugate (**95**) in 78% yield (Fig. 7A). The photocatalyzed C─H boronation of sulfonyl BCP-H gave sulfonyl BCP-boronate (**96**) and side product (**97**) (Fig. 7B), which have become exceptionally versatile building blocks in modern organic synthesis (*64*). Condensation of compound (**39**) with benzoylhydrazine in dimethyl sulfoxide provided the sulfonyl BCP-oxadiazole (**98**) in 81% yield. Moreover, treatment of compound (**39**) with Selectfluor selectively yielded the α-trifluoromethyl alcohol derivative (**99**) in 73% yield (Fig. 7C). Reduction of sulfonyl BCP-ketone (**47**) with NaBH_4_ furnished sulfonyl BCP alcohol (**100**) in 90% yield. Besides, sequential treatment of this compound (**47**) with hydroxylamine hydrochloride and 5-bromovaleric acid gave the sulfonyl BCP-oxime ester (**101**) in 54% yield (Fig. 7D). Selective oxidation of the thioether moiety in sulfonyl BCP-thioether (**49**) could be tuned by varying the amount of Oxone and the reaction solvent, delivering sulfonyl BCP sulfoxide (**102**) and disulfonyl BCP (**103**) in good yields, respectively (Fig. 7E). Sulfonyl BCP-azo (**54**) was obtained in 46% yield via reaction of sulfonyl BCP-I (**84**) with a diazonium salt; this substrate also proved compatible with Ullmann-type C─N coupling conditions (Fig. 7F). In addition, sulfonyl BCP-CN (**86**) served as a versatile building block for accessing diverse sulfonyl BCP derivatives (Fig. 7G). For example, treatment with Grignard reagents produced sulfonyl BCP-ketone (**44**) in 36% yield, while a Ni/NaBH_4_ system reduced it to the valuable sulfonyl BCP-alkylamine (**105**). Furthermore, hydrolysis of sulfonyl BCP-CN (**86**) afforded sulfonyl BCP-COOH (**106**), which underwent condensation under various conditions to furnish both sulfonyl BCP-amide (**107**) and sulfonyl BCP-ester (**108**) in good yields. Collectively, these rapid and efficient transformations highlight the utility of this methodology as a versatile approach to structurally diverse, pharmacologically relevant BCP building blocks.

### Mechanistic investigations

**Fig. 8.**
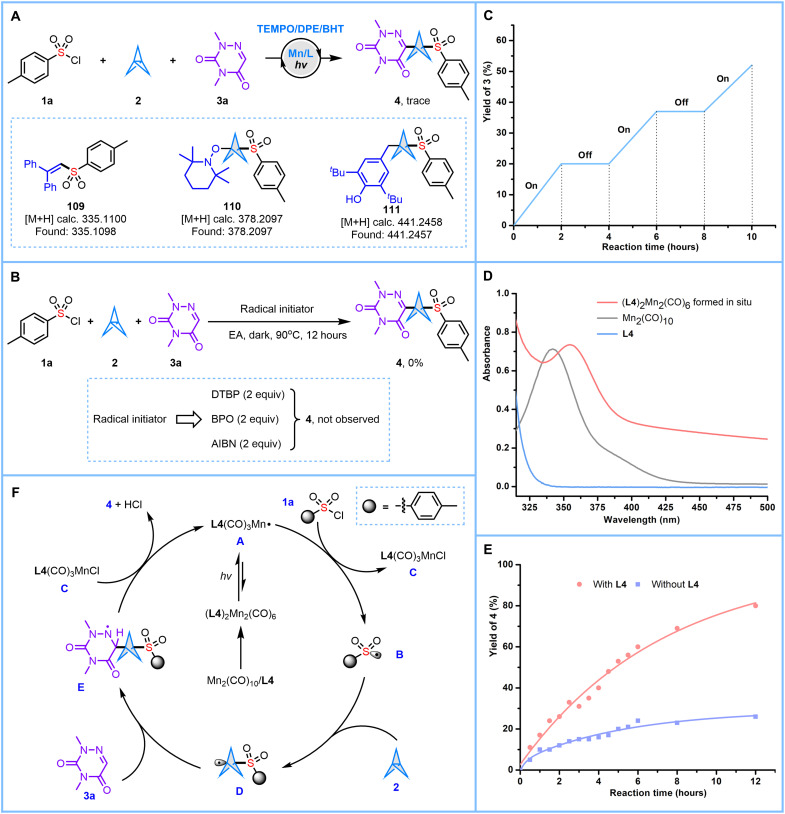
Mechanistic studies. (**A**) Radical scavenging experiment. (**B**) Importance of photoinduced Mn-catalyzed system. (**C**) Turn on/off profile experiment. (**D**) UV-vis absorption spectrum. (**E**) Importance of ligand for the reaction. (**F**) Proposed mechanism. TEMPO, 2,2,6,6-tetramethyl-1-piperidinyloxy; DPE, 1,1-diphenylethylene; BHT, butylated hydroxytoluene; DTBP, di-tert-butyl peroxide; BPO, benzoyl peroxide; AIBN, 2,2′-azobis(isobutyronitrile).

To gain insight into the possible reaction pathway, a series of mechanistic studies was performed (Fig. 8). When the radical scavenger 2,2,6,6-tetramethyl-1-piperidinyloxy, 1,1-diphenylethylene, or butylated hydroxytoluene was added to the reaction mixture, the transformation was completely shut down, and the corresponding adducts (**109** to **111**) were detected by a high-resolution mass spectrometer (Fig. 8A). These results verified the radical nature of the difunctionalization reaction. Standard thermal activation of the archetypal radical sources di-tert-butyl peroxide, benzoyl peroxide, and 2,2′-azobis(isobutyronitrile) proved ineffective for the synthesis of sulfonyl BCP-heteroarene (**4**) (Fig. 8B), underscoring that the transformation was strictly photocatalyst dependent and proceeds only under visible light irradiation. The reaction mixture of Mn_2_(CO)_10_ and L4 in EA was irradiated with 10-W blue LEDs at room temperature under N_2_ for 12 hours to give (L4)_2_Mn_2_(CO)_6_ in 16% yield, and the complex was fully characterized by nuclear magnetic resonance spectroscopy (figs. S10 to S12). The catalytic activity of this complex was then verified by using it as a photocatalyst, which afforded the desired product (**4**) in 36% yield. Turn on/off profile experiments excluded a dominant long-lived radical chain (Fig. 8C), yet transient manganese-centered radicals generated during the photocycle might still dimerize, so a short-chain manifold mediated by Mn species remained plausible (*52*). The ultraviolet-visible (UV-vis) experiments revealed that the adduct formed in situ from Mn_2_(CO)_10_ and L4, assigned as (L4)_2_Mn_2_(CO)_6_, exhibits a red-shifted absorption maximum relative to the parent metal carbonyl (Fig. 8D). This bathochromic shift enabled visible light harvesting by the dinuclear complex, implying that photolysis with blue LEDs generated manganese-centered radicals more efficiently than direct excitation of Mn_2_(CO)_10_. Kinetic analysis revealed that L4 was essential for rate acceleration; omitting the ligand afforded sulfonyl BCP-heteroarene (**4**) in markedly diminished yield under otherwise identical conditions (Fig. 8E).

On the basis of the accumulated experimental evidence and earlier literature (*8*–*10*, *52*, *65*), we proposed the catalytic cycle depicted in Fig. 8F for the visible light–driven manganese-catalyzed difunctionalization of [1.1.1]propellane. After photon absorption by the (L4)_2_Mn_2_(CO)_6_ adduct generated in situ from Mn_2_(CO)_10_ and L4, the Mn─Mn bond undergoes homolytic cleavage, thereby furnishing the manganese-centered radical (**A**). This species undergoes a XAT process to abstract a chlorine atom from the sulfonyl chloride (**1a**), concomitantly producing sulfonyl radical (**B**) and chloro-manganese complex (**C**). Addition of sulfonyl radical (**B**) to the central bond of [1.1.1]propellane (**2**) affords bicyclic alkyl radical (**D**) (4.6 kcal/mol), which subsequently attacks heteroarene (**3a**) to form intermediate (**E**). Last, a hydrogen atom transfer pathway of the L4(CO_3_)MnCl with intermediate (**E**) may take place to deliver the final product (**4**) and regenerate manganese-centered radical (**A**), thereby closing the catalytic manifold. Density functional theory calculations at the wb97xd/6-31 g(d) level were also performed to verify the proposed mechanism (fig. S9). In the three-component pathway, radical **B** converts to intermediate **D** (+4.6 kcal/mol) via a low-energy transition state **TS1** (+11.0 kcal/mol). Subsequently, the system overcomes a higher barrier at **TS2** (+16.4 kcal/mol) to reach intermediate **E** (−15.0 kcal/mol), ultimately affording the thermodynamically favored product **4** (−24.5 kcal/mol). In contrast, the two-component pathway requires **B** to overcome the highest barrier at **TS3** (+18.3 kcal/mol) to form intermediate **F** (+14.8 kcal/mol), which then leads to product **G** (−16.0 kcal/mol). This pathway is less favorable in both reaction kinetics and product stability. Therefore, these results suggest that the reaction selectivity is largely controlled by the three-component pathway.

## DISCUSSION

Here, we reported a visible light–driven, manganese-catalyzed protocol for the rapid assembly of diverse sulfonyl BCP derivatives through the sequential difunctionalization of [1.1.1]propellane. The reaction proceeds via a XAT radical addition process, provides access to sulfonyl BCP─Cl, ─Br, ─I, ─CN, ─SCD_3_, ─H, ─HetAr, ─COAr, ─SAr, ─N═NAr, etc. under standard conditions. The manifold tolerates a wide palette of polar and reducible functional groups, and its operational simplicity is underscored by the direct late-stage derivatization of complex pharmaceuticals and natural products. Mechanistic investigations prove a sulfonyl-radical manifold initiated by photogenerated Mn-centered radicals, delineating a catalytic cycle that operates efficiently at room temperature. Given the escalating demand for BCP motifs as saturated bioisosteres in medicinal chemistry, this platform furnishes a concise and general gateway to richly decorated sulfonyl BCPs, with immediate utility in drug discovery and chemical biology programs.

## MATERIALS AND METHODS

### General procedure for the synthesis of sulfonyl BCP-heteroarenes

A 25-ml reaction tube equipped with a magnetic stirring bar was charged with sulfonyl chlorides (**1**) (1.5 equiv), [1.1.1]propellane (**2**) (1 equiv), heteroarenes (**3**) (0.2 mmol), Mn_2_(CO)_10_ (5 mmol %), L (10 mol %), BTMG (2 equiv), and EA (2 ml). The reaction mixture was allowed to stir with the irradiation of blue LEDs at room temperature under a nitrogen atmosphere for 12 hours. Upon completion, it was quenched by a saturated NH_4_Cl solution and extracted with ethyl acetate (EtOAc), and the combined organic layer was dried over MgSO_4_. The solvent was removed in vacuo, and the obtained residue was further purified by silica gel column chromatography (200- to 300-mesh silica gel).

### General procedure for the synthesis of sulfonyl BCP-H

A 25-ml reaction tube equipped with a magnetic stirring bar was charged with sulfonyl chlorides (**1**) (1.5 equiv), [1.1.1]propellane (**2**) (1 equiv), Et_3_SiH (0.2 mmol), Mn_2_(CO)_10_ (5 mmol %), L (10 mol %), and NMP (2 ml). The reaction mixture was allowed to stir with the irradiation of blue LEDs at room temperature under a nitrogen atmosphere for 12 hours. Upon completion, it was quenched by a saturated NH_4_Cl solution and extracted with EtOAc, and the combined organic layer was dried over MgSO_4_. The solvent was removed in vacuo, and the obtained residue was further purified by silica gel column chromatography (200- to 300-mesh silica gel).

### General procedure for the synthesis of sulfonyl BCP-EWG-Ph

A 25-ml reaction tube equipped with a magnetic stirring bar was charged with sulfonyl chlorides (**1**) (1.5 equiv), [1.1.1]propellane (**2**) (1 equiv), electron-deficient aromatics (0.2 mmol), Mn_2_(CO)_10_ (5 mmol %), L (10 mol %), BTMG (2 equiv), and EA (2 ml). The reaction mixture was allowed to stir with the irradiation of blue LEDs at room temperature under a nitrogen atmosphere for 12 hours. Upon completion, it was quenched by a saturated NH_4_Cl solution and extracted with EtOAc, and the combined organic layer was dried over MgSO_4_. The solvent was removed in vacuo, and the obtained residue was further purified by silica gel column chromatography (200- to 300-mesh silica gel).

### General procedure for the synthesis of gem-difluoroallylic BCP-sulfone

A 25-ml reaction tube equipped with a magnetic stirring bar was charged with sulfonyl chlorides (**1**) (1.5 equiv), [1.1.1]propellane (**2**) (1 equiv), (3,3,3-trifluoroprop-1-en-2-yl)benzene (0.2 mmol), Mn_2_(CO)_10_ (5 mmol %), L (10 mol %), and NMP (2 ml). The reaction mixture was allowed to stir with the irradiation of blue LEDs at room temperature under a nitrogen atmosphere for 16 hours. Upon completion, it was quenched by a saturated NH_4_Cl solution and extracted with EtOAc, and the combined organic layer was dried over MgSO_4_. The solvent was removed in vacuo, and the obtained residue was further purified by silica gel column chromatography (200- to 300-mesh silica gel).

### General procedure for the synthesis of sulfonyl BCP-ketone

A 25-ml reaction tube equipped with a magnetic stirring bar was charged with sulfonyl chlorides (**1**) (1.5 equiv), [1.1.1]propellane (**2**) (1 equiv), aromatic aldehydes (0.2 mmol), Mn_2_(CO)_10_ (5 mmol %), L (10 mol %), BTMG (2 equiv), and EA (2 ml). The reaction mixture was allowed to stir with the irradiation of blue LEDs at room temperature under a nitrogen atmosphere for 12 hours. Upon completion, it was quenched by a saturated NH_4_Cl solution and extracted with EtOAc, and the combined organic layer was dried over MgSO_4_. The solvent was removed in vacuo, and the obtained residue was further purified by silica gel column chromatography (200- to 300-mesh silica gel).

### General procedure for the synthesis of sulfonyl BCP-thioether/selenide

A 25-ml reaction tube equipped with a magnetic stirring bar was charged with sulfonyl chlorides (**1**) (1.5 equiv), [1.1.1]propellane (**2**) (1 equiv), disulfide/diselenide (0.2 mmol), Mn_2_(CO)_10_ (5 mmol %), L (10 mol %), and EA (2 ml). The reaction mixture was allowed to stir with the irradiation of blue LEDs at room temperature under a nitrogen atmosphere for 6 hours. Upon completion, it was quenched by a saturated NH_4_Cl solution and extracted with EtOAc, and the combined organic layer was dried over MgSO_4_. The solvent was removed in vacuo, and the obtained residue was further purified by silica gel column chromatography (200- to 300-mesh silica gel).

### General procedure for the synthesis of sulfonyl BCP-azo

A 25-ml reaction tube equipped with a magnetic stirring bar was charged with sulfonyl chlorides (**1**) (1.5 equiv), [1.1.1]propellane (**2**) (0.2 mmol), 4-methoxybenzenediazonium tetrafluoroborate (2 equiv), Mn_2_(CO)_10_ (5 mmol %), L (10 mol %), Cs_2_CO_3_ (2 equiv), and acetonitrile (2 ml). The reaction mixture was allowed to stir with the irradiation of blue LEDs at room temperature under a nitrogen atmosphere for 12 hours. Upon completion, it was quenched by a saturated NH_4_Cl solution and extracted with EtOAc, and the combined organic layer was dried over MgSO_4_. The solvent was removed in vacuo, and the obtained residue was further purified by silica gel column chromatography (200- to 300-mesh silica gel).

## Supplementary Materials

### This PDF file includes:

Supplementary Text Figs. S1 to S12 Tables S1 to S4 NMR spectra References
