# Supplementary material for: A general photoinduced manganese-catalyzed platform for the sequential difunctionalization of [1.1.1]propellane
Source: Sci Adv. 2026 Jun 26;12(26):eaeg5293. doi: 10.1126/sciadv.aeg5293 (PMC13308620; doi:10.1126/sciadv.aeg5293)
Supplement: Supplementary file 1 — Supplementary Text Figs. S1 to S12 Tables S1 to S4 NMR spectra References [file sciadv.aeg5293_sm.pdf]

Science Advances
AAAS

Supplementary Materials for

A general photoinduced manganese-catalyzed platform for the sequential difunctionalization of [1.1.1]propellane

Jun Xu *et al.*

Corresponding author: Huamin Wang, wanghuamin@zju.edu.cn; Pengfei Zhang, pfzhang@hznu.edu.cn

Sci. Adv. **12**, eaeg5293 (2026)
DOI: 10.1126/sciadv.aeg5293

This PDF file includes:

Supplementary Text
Figs. S1 to S12
Tables S1 to S4
NMR spectra
References

1. Materials and methods

All reagents and deuterated solvents were commercially available and used without further purification. All products were separated by silica gel (200-300 mesh) column chromatography with petroleum ether (PE) (60-90°C) and ethyl acetate (EA). ^{1}H, ^{13}C, and ^{19}F NMR spectra were recorded on a Bruker Advance 400 or 500 spectrometer at ambient temperature with $CDCl_3$ as solvent and tetramethylsilane (TMS) as the internal standard. Melting points were determined on an X-5 Data microscopic melting point apparatus. Analytical thin layer chromatography (TLC) was performed on Merk precoated TLC (silica gel 60 F254) plates. Compounds for HRMS were analyzed by positive mode electrospray ionization (ESI) using Agilent 6530 QTOF mass spectrometer. The photoreactor (PL-SX100A) was purchased from Beijing Princess Technology Co., Ltd.

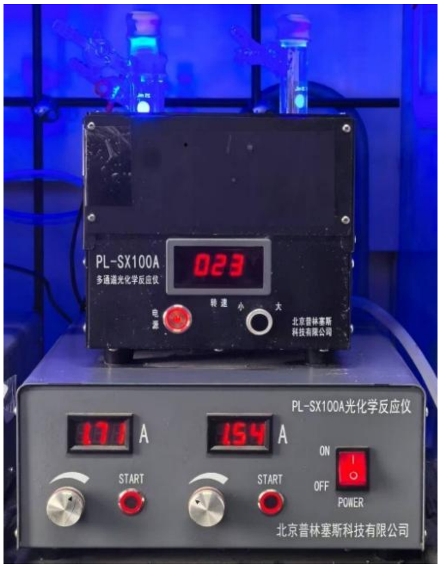

Fig. S1. The photoreactor is shown in photograph. The distance from the light source to the reaction tube was 2.0 cm, and the power was 10 W.

2. Experimental section

2.1 Optimization of reaction conditions

Table S1. Evaluation of mixed solvents

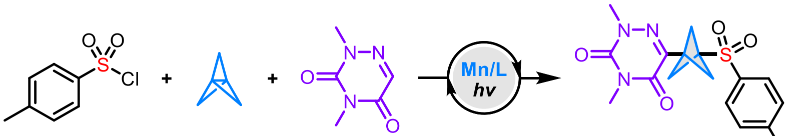

1a 2 3a 4

Entry ^{a}	Mixed solvents	Yield (%) ^{b}
1	EA/DCE (v/v = 1/1)	40
2	EA/NMP (v/v = 1/1)	72
3	EA/DMF (v/v = 1/1)	53
4	EA/acetone (v/v = 1/1)	31
5	EA/THF (v/v = 1/1)	36

^{a} Reaction conditions: **1a** (1.5 equiv), **2** (1 equiv), **3a** (0.2 mmol), $Mn_2(CO)_{10}$ (5 mol%), L4 (10 mol%), BTMG (2 equiv), solvent (2.0 mL), blue LEDs (455 nm), rt, N_2, 12 h. ^{b} Isolated yields.

Table S2. Evaluation of LEDs with different wavelengths

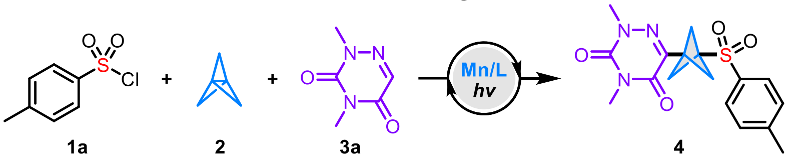

Entry [a]	Wavelength (nm)	Yield (%) [b]
1	365	50
2	405	69
3	455	80
4	520	35
5	660	0

[a] Reaction conditions: **1a** (1.5 equiv), **2** (1 equiv), **3a** (0.2 mmol), $Mn_2(CO)_{10}$ (5 mol%), L4 (10 mol%), BTMG (2 equiv), EA (2.0 mL), LEDs, rt, N_2, 12 h. [b] Isolated yields.

2.2 General procedure for the synthesis of [1.1.1]propellane (*66*)

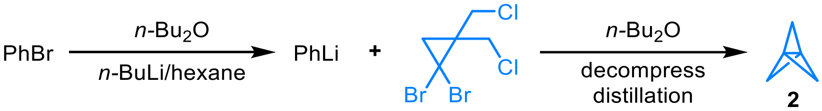

A 150 mL three-neck round bottom flask equipped with a magnetic stirring bar was charged with bromobenzene (100 mmol, 1 equiv.) and *n*-Bu_2O (20 mL). The reaction mixture was cooled down to -30 °C and *n*-BuLi (100 mmol, 1 equiv., 2.5 M in hexane) was added dropwise. After the addition was completed, the reaction mixture was allowed to warm to room temperature, and stirred at room temperature for 1 h. The reaction mixture was used directly in the next step.
A solution of the above prepared PhLi in *n*-Bu_2O/hexane (65 mL) was added dropwise to a suspension of 1,1-dibromo-2,2-bis(chloromethyl)cyclopropane (45 mmol) in anhydrous *n*-Bu_2O (20 mL) at -20 °C. After the addition was completed, the reaction mixture was allowed to warm to 0 °C and stirred for 2 h. Then the addition funnel was swapped out for a distillation head with attached 100 mL round bottom flask in a bath (liquid nitrogen). A vacuum was slowly applied to the system and the distillate was collected, while maintaining the reaction/distillation flask below 0 °C. Approximately 30 mL of distillate was collected. The concentration of [1.1.1]propellane (0.4-0.6 M) was measured by ^{1}H NMR using dichloromethane as the standard.

2.3 General procedure for the synthesis of azauracils (*67*)

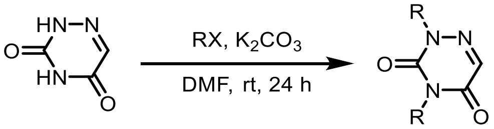

A 100 mL round bottom flask equipped with a magnetic stirring bar was charged with 6-azuracil (5 mmol), alkyl halides (3 equiv), K_2CO_3 (3 equiv) and DMF (25 mL). The reaction mixture was allowed to stir at room temperature for 24 h. Upon completion, it was quenched by water and extracted with EtOAc. The combined organic layer was washed with brine, dried over $MgSO_4$. The solvent was removed *in vacuo*, and the obtained residue was further purified by silica gel column chromatography (200-300 mesh silica gel).

2.4 General procedure for the synthesis of quinoxaline-2(1*H*)-ones (*68*)

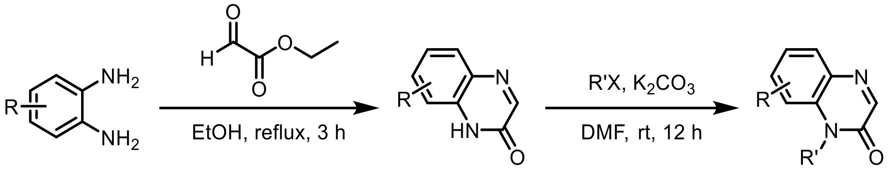

A 100 mL round bottom flask equipped with a magnetic stirring bar was charged with *o*-arylenediamine (5 mmol), ethyl glyoxylate (1.5 equiv) and ethanol (25 mL). The reaction mixture was allowed to stir at reflux for 3 h. The precipitated solid was filtered and washed with ethanol, then dried to give the first step product.

To a suspension of the first step product in DMF was added alkyl halides (1.5 equiv) and potassium carbonate (1.5 equiv). Then the reaction mixture was allowed to stir at room temperature for 12 h. Upon completion, it was quenched by water and extracted with EtOAc. The combined organic layer was washed with brine, dried over $MgSO_4$. The solvent was removed *in vacuo*, and the obtained residue was further purified by silica gel column chromatography (200-300 mesh silica gel).

2.5 General procedure for the synthesis of pyrazinone (*68*)

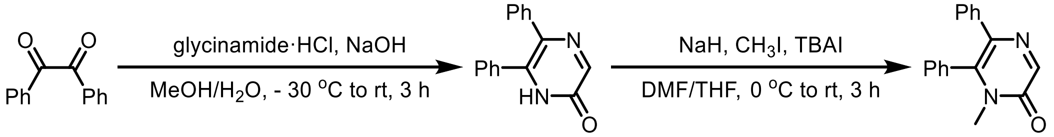

A 100 mL round bottom flask equipped with a magnetic stirring bar was charged with glycinamide hydrochloride (5 mmol), NaOH solution (1.2 equiv, 12.5 M), MeOH (15 mL) and water (5 mL). Then the reaction mixture was allowed to stir at -30 °C, followed by the addition of benzil (1 equiv) and the resulting mixture was allowed to stir at -30 °C for 2 h and room temperature for 1 h. Upon completion, it was cooled in an ice bath and acidified with AcOH. The precipitate separated was collected by filtration and dried to afford crude product as a pale red solid.

To a reaction tube equipped with a magnetic stirring bar was charged with sodium hydride (5.2 mmol, 60 % dispersion in mineral oil), DMF (2 mL). Then, a solution of crude product (3.5 mmol) in DMF (2 mL) and THF (2 mL) was added dropwise at 0 °C. A solution of methyl iodide (4.6 mmol) in THF (1 mL) was added dropwise after 15 min at 0 °C. Then *tetra*-butyl ammonium iodide (0.35 mmol) was added to the reaction mixture. The reaction mixture was allowed to stir at room temperature for 3 h. Upon completion, it was quenched by ice water and extracted with EtOAc. The combined organic layer was washed with brine, dried over $MgSO_4$. The solvent was removed *in vacuo*, and the obtained residue was further purified by silica gel column chromatography (200-300 mesh silica gel).

2.6 General procedure for the synthesis of 4-azacoumarin (*69*)

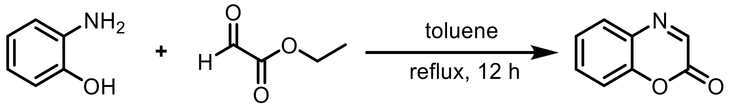

A 100 mL round bottom flask equipped with a magnetic stirring bar was charged with 2-aminophenol (5 mmol), ethyl glyoxylate (1.5 equiv), dried 4Å molecular sieves (0.5 g) and anhydrous toluene (25 mL). The reaction mixture was allowed to stir at reflux for 12 h. Upon completion, the mixture was cooled to room temperature and filtered through a plug of celite. The resulting organic layer was washed with brine, dried over $MgSO_4$. The solvent was removed *in*

vacuo, and the obtained residue was further purified by silica gel column chromatography (200-300 mesh silica gel).

2.7 General procedure for the synthesis of drug derivatives (*70*)

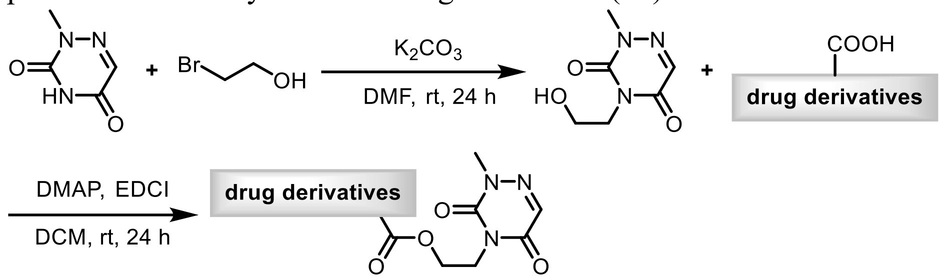

A 100 mL round bottom flask equipped with a magnetic stirring bar was charged with 2-methyl-1,2,4-triazine-3,5(2H,4H)-dione (5 mmol), 2-bromoethanol (1.5 equiv), K_2CO_3 (2 equiv) and DMF (25 mL). The reaction mixture was allowed to stir at room temperature for 24 h. Upon completion, it was quenched by water and extracted with EtOAc. The combined organic layer was washed with brine, dried over $MgSO_4$. The solvent was removed *in vacuo*, and the obtained residue was further purified by silica gel column chromatography (200-300 mesh silica gel).

A 100 mL round bottom flask equipped with a magnetic stirring bar was charged with 4-(2-hydroxyethyl)-2-methyl-1,2,4-triazine-3,5(2H,4H)-dione (4 mmol), carboxylic acid drug molecules (1.1 equiv), DMAP (10 mol%), EDCl (2 equiv), and DMF (25 mL). The reaction mixture was allowed to stir at room temperature for 24 h. Upon completion, it was quenched by water and extracted with EtOAc. The combined organic layer was washed with brine, dried over $MgSO_4$. The solvent was removed *in vacuo*, and the obtained residue was further purified by silica gel column chromatography (200-300 mesh silica gel).

2.8 General procedure for the synthesis of sulfonyl chlorides (*71*)

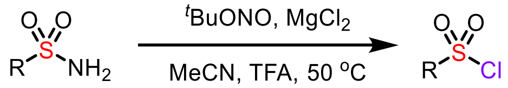

A 25 mL reaction tube equipped with a magnetic stirring bar was charged with sulfonamide (1 mmol), ^{t}BuONO (3 equiv), $MgCl_2$ (1 equiv), TFA (1 equiv) and MeCN (5 mL). The reaction mixture was allowed to stir under N_2 at 50 °C for 5 h. Upon completion, it was quenched by water and extracted with EtOAc. The combined organic layer was dried over $MgSO_4$. The solvent was removed *in vacuo*, and the obtained residue was further purified by silica gel column chromatography (200-300 mesh silica gel).

2.9 General procedure for the synthesis of sulfonyl bromides (*72*)

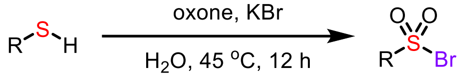

A 25 mL reaction tube equipped with a magnetic stirring bar was charged with thiol (2 mmol), oxone (2.5 equiv), KBr (1 equiv) and water (8 mL). The reaction mixture was allowed to stir at 45 °C for 12 h. Upon completion, it was extracted with EtOAc, and the combined organic layer was dried over $MgSO_4$. The solvent was removed *in vacuo*, and the obtained residue was further purified by silica gel column chromatography (200-300 mesh silica gel).

2.10 General procedure for the synthesis of sulfonyl iodides (*73*)

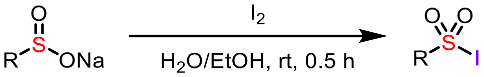

A 25 mL reaction tube equipped with a magnetic stirring bar was charged with sodium sulfite (1 mmol), I_2 (1.1 equiv), H_2O (3 equiv) and EtOH (3 mL). The reaction mixture was allowed to stir at room temperature for 0.5 h. Upon completion, it was extracted with EtOAc, and the combined organic layer was dried over $MgSO_4$. The solvent was removed *in vacuo* to give the residue, which was used without further purification.

2.11 General procedure for the synthesis of sulfonyl BCP-heteroarenes

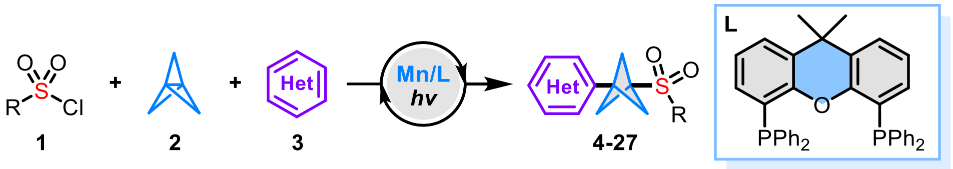

A 25 mL reaction tube equipped with a magnetic stirring bar was charged with sulfonyl chlorides (**1**) (1.5 equiv), [1.1.1]propellane (**2**) (1 equiv), heteroarenes (**3**) (0.2 mmol), $Mn_2(CO)_{10}$ (5 mmol%), L (10 mol%), BTMG (2 equiv) and EA (2 mL). The reaction mixture was allowed to stir with the irradiation of blue LEDs at room temperature under a nitrogen atmosphere for 12 h. Upon completion, it was quenched by a saturated NH_4Cl solution and extracted with EtOAc, and the combined organic layer was dried over $MgSO_4$. The solvent was removed *in vacuo*, and the obtained residue was further purified by silica gel column chromatography (200-300 mesh silica gel).

2.12 General procedure for the synthesis of sulfonyl BCP-H

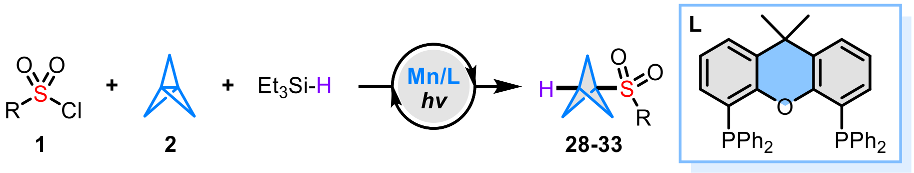

A 25 mL reaction tube equipped with a magnetic stirring bar was charged with sulfonyl chlorides (**1**) (1.5 equiv), [1.1.1]propellane (**2**) (1 equiv), Et_3SiH (0.2 mmol), $Mn_2(CO)_{10}$ (5 mmol%), L (10 mol%), and NMP (2 mL). The reaction mixture was allowed to stir with the irradiation of blue LEDs at room temperature under a nitrogen atmosphere for 12 h. Upon completion, it was quenched by a saturated NH_4Cl solution and extracted with EtOAc, and the combined organic layer was dried over $MgSO_4$. The solvent was removed *in vacuo*, and the obtained residue was further purified by silica gel column chromatography (200-300 mesh silica gel).

2.13 General procedure for the synthesis of sulfonyl BCP-EWG-Ph

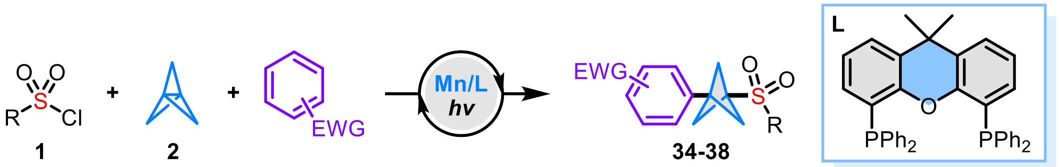

A 25 mL reaction tube equipped with a magnetic stirring bar was charged with sulfonyl chlorides (**1**) (1.5 equiv), [1.1.1]propellane (**2**) (1 equiv), electron-deficient aromatics (0.2 mmol),

$Mn_2(CO)_{10}$ (5 mmol%), L (10 mol%), BTMG (2 equiv), and EA (2 mL). The reaction mixture was allowed to stir with the irradiation of blue LEDs at room temperature under a nitrogen atmosphere for 12 h. Upon completion, it was quenched by a saturated NH_4Cl solution and extracted with EtOAc, and the combined organic layer was dried over $MgSO_4$. The solvent was removed *in vacuo*, and the obtained residue was further purified by silica gel column chromatography (200-300 mesh silica gel).

2.14 General procedure for the synthesis of gem-difluoroallylic BCP-sulfone

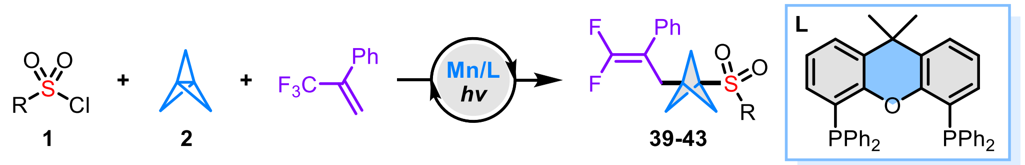

A 25 mL reaction tube equipped with a magnetic stirring bar was charged with sulfonyl chlorides (**1**) (1.5 equiv), [1.1.1]propellane (**2**) (1 equiv), (3,3,3-trifluoroprop-1-en-2-yl)benzene (0.2 mmol), $Mn_2(CO)_{10}$ (5 mmol%), L (10 mol%), and NMP (2 mL). The reaction mixture was allowed to stir with the irradiation of blue LEDs at room temperature under a nitrogen atmosphere for 16 h. Upon completion, it was quenched by a saturated NH_4Cl solution and extracted with EtOAc, and the combined organic layer was dried over $MgSO_4$. The solvent was removed *in vacuo*, and the obtained residue was further purified by silica gel column chromatography (200-300 mesh silica gel).

2.15 General procedure for the synthesis of sulfonyl BCP-ketone

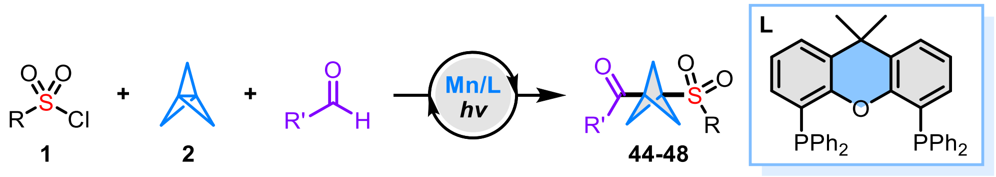

A 25 mL reaction tube equipped with a magnetic stirring bar was charged with sulfonyl chlorides (**1**) (1.5 equiv), [1.1.1]propellane (**2**) (1 equiv), aromatic aldehydes (0.2 mmol), $Mn_2(CO)_{10}$ (5 mmol%), L (10 mol%), BTMG (2 equiv) and EA (2 mL). The reaction mixture was allowed to stir with the irradiation of blue LEDs at room temperature under a nitrogen atmosphere for 12 h. Upon completion, it was quenched by a saturated NH_4Cl solution and extracted with EtOAc, and the combined organic layer was dried over $MgSO_4$. The solvent was removed *in vacuo*, and the obtained residue was further purified by silica gel column chromatography (200-300 mesh silica gel).

2.16 General procedure for the synthesis of sulfonyl BCP-thioether/selenide

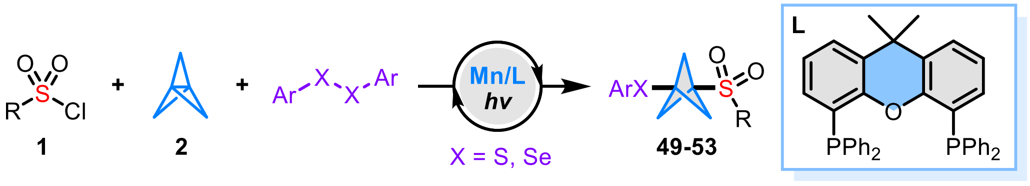

A 25 mL reaction tube equipped with a magnetic stirring bar was charged with sulfonyl chlorides (**1**) (1.5 equiv), [1.1.1]propellane (**2**) (1 equiv), disulfide/diselenide (0.2 mmol), $Mn_2(CO)_{10}$ (5 mmol%), L (10 mol%), and EA (2 mL). The reaction mixture was allowed to stir with the irradiation of blue LEDs at room temperature under a nitrogen atmosphere for 6 h. Upon

completion, it was quenched by a saturated NH_4Cl solution and extracted with EtOAc, and the combined organic layer was dried over $MgSO_4$. The solvent was removed *in vacuo*, and the obtained residue was further purified by silica gel column chromatography (200-300 mesh silica gel).

2.17 General procedure for the synthesis of sulfonyl BCP-azo

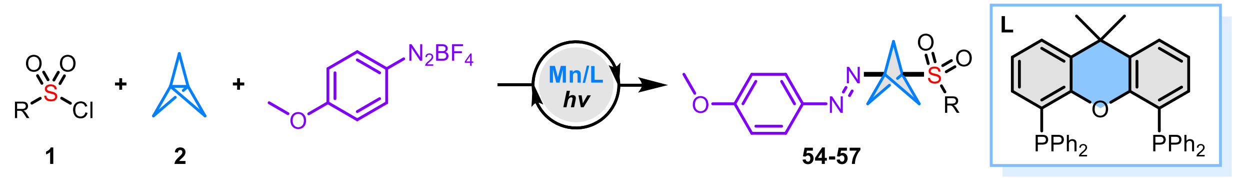

A 25 mL reaction tube equipped with a magnetic stirring bar was charged with sulfonyl chlorides (**1**) (1.5 equiv), [1.1.1]propellane (**2**) (0.2 mmol), 4-methoxybenzenediazonium tetrafluoroborate (2 equiv), $Mn_2(CO)_{10}$ (5 mmol%), L (10 mol%), Cs_2CO_3 (2 equiv), and MeCN (2 mL). The reaction mixture was allowed to stir with the irradiation of blue LEDs at room temperature under a nitrogen atmosphere for 12 h. Upon completion, it was quenched by a saturated NH_4Cl solution and extracted with EtOAc, and the combined organic layer was dried over $MgSO_4$. The solvent was removed *in vacuo*, and the obtained residue was further purified by silica gel column chromatography (200-300 mesh silica gel).

2.18 General procedure for the C-C coupling of sulfonyl BCP-H

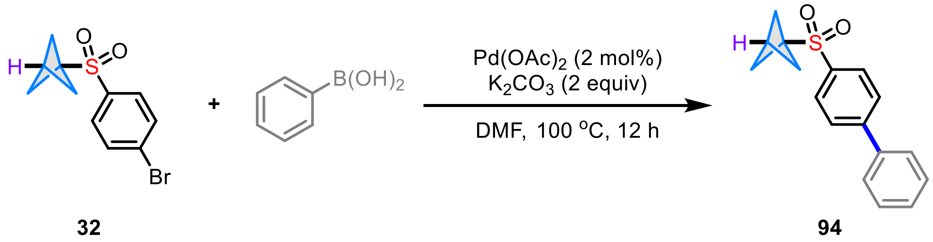

A 25 mL reaction tube equipped with a magnetic stirring bar was charged with sulfonyl BCP-H (**32**) (0.2 mmol), phenylboronic acid (2 equiv), $Pd(OAc)_2$ (2 mol%), K_2CO_3 (2 equiv), and DMF (2 mL). The reaction mixture was allowed to stir at 100 °C under a nitrogen atmosphere for 12 h. Upon completion, it was quenched by a saturated NH_4Cl solution and extracted with EtOAc, and the combined organic layer was dried over $MgSO_4$. The solvent was removed *in vacuo*, and the obtained residue was further purified by silica gel column chromatography (200-300 mesh silica gel).

2.19 General procedure for the C-O coupling of sulfonyl BCP-Cl

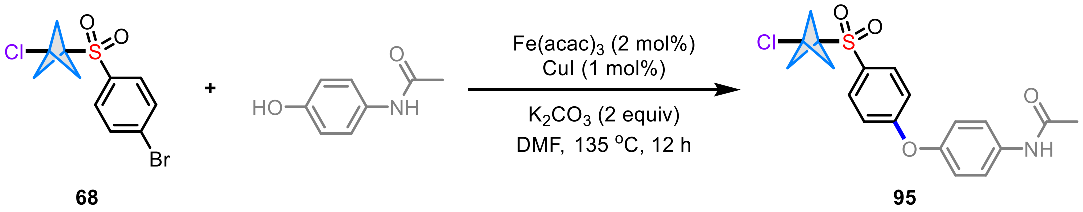

A 25 mL reaction tube equipped with a magnetic stirring bar was charged with sulfonyl BCP-Cl (**68**) (0.2 mmol), acetaminophen (2 equiv), $Fe(acac)_3$ (2 mol%), CuI (1 mol%), K_2CO_3 (2 equiv), and DMF (2 mL). The reaction mixture was allowed to stir at 135 °C under a nitrogen atmosphere for 12 h. Upon completion, it was quenched by a saturated NH_4Cl solution and extracted with

EtOAc, and the combined organic layer was dried over $MgSO_4$. The solvent was removed *in vacuo*, and the obtained residue was further purified by silica gel column chromatography (200-300 mesh silica gel).

2.20 General procedure for the C-H boronation of sulfonyl BCP-H

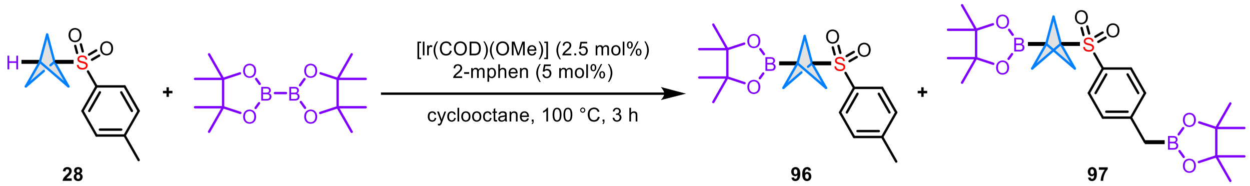

28 96 97

A 25 mL reaction tube equipped with a magnetic stirring bar was charged with sulfonyl BCP-H (**28**) (0.2 mmol), bis(pinacolato)diboron (2 equiv), [Ir(COD)(OMe)] (2.5 mol%), 2-mphen (5 mol%), and cyclooctane (2 mL). The reaction mixture was allowed to stir at 100 °C for 3 h. Upon completion, it was quenched by a saturated NH_4Cl solution and extracted with EtOAc, and the combined organic layer was dried over $MgSO_4$. The solvent was removed *in vacuo*, and the obtained residue was further purified by silica gel column chromatography (200-300 mesh silica gel).

2.21 General procedure for the condensation of sulfonyl BCP

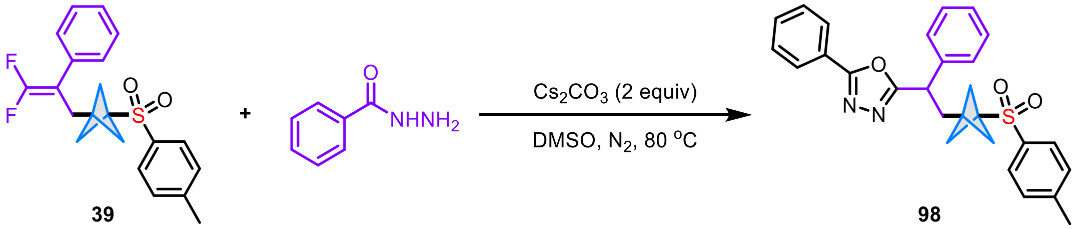

39 98

A 25 mL reaction tube equipped with a magnetic stirring bar was charged with sulfonyl BCP (**39**) (0.2 mmol), benzoylhydrazine (2 equiv), Cs_2CO_3 (2 equiv), and DMSO (2 mL). The reaction mixture was allowed to stir at 80 °C under a nitrogen atmosphere for 12 h. Upon completion, it was quenched by a saturated NH_4Cl solution and extracted with EtOAc, and the combined organic layer was dried over $MgSO_4$. The solvent was removed *in vacuo*, and the obtained residue was further purified by silica gel column chromatography (200-300 mesh silica gel).

2.22 General procedure for the synthesis of sulfonyl BCP-based alcohol

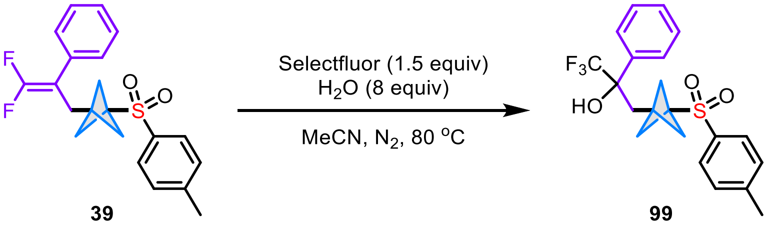

39 99

A 25 mL reaction tube equipped with a magnetic stirring bar was charged with sulfonyl BCP (**39**) (0.2 mmol), Selectfluor (1.5 equiv), H_2O (8 equiv), and MeCN (2 mL). The reaction mixture was allowed to stir at 80 °C under a nitrogen atmosphere for 12 h. Upon completion, it was quenched by a saturated NH_4Cl solution and extracted with EtOAc, and the combined organic layer was

dried over $MgSO_4$. The solvent was removed *in vacuo*, and the obtained residue was further purified by silica gel column chromatography (200-300 mesh silica gel).

2.23 General procedure for the reduction of sulfonyl BCP-ketone

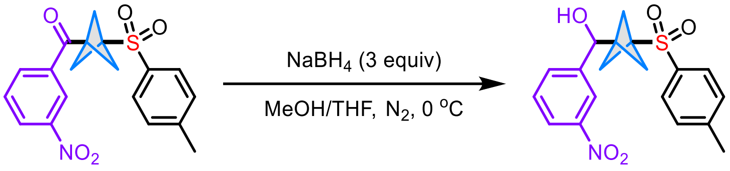

47 **100**

A 25 mL reaction tube equipped with a magnetic stirring bar was charged with sulfonyl BCP-ketone (**47**) (0.2 mmol), $NaBH_4$ (3 equiv), and MeOH/THF (2 mL, v:v = 1:1). The reaction mixture was allowed to stir at 0 °C for 6 h. Upon completion, it was quenched by a saturated NH_4Cl solution and extracted with EtOAc, and the combined organic layer was dried over $MgSO_4$. The solvent was removed *in vacuo*, and the obtained residue was further purified by silica gel column chromatography (200-300 mesh silica gel).

2.24 General procedure for the synthesis of sulfonyl BCP-oxime ester

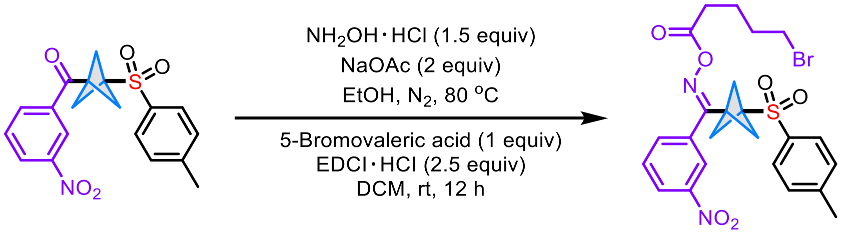

47 **101**

A 25 mL reaction tube equipped with a magnetic stirring bar was charged with sulfonyl BCP-ketone (**47**) (0.2 mmol), $NH_2OH·HCl$ (1.5 equiv), NaOAc (2 equiv), and EtOH (2 mL). The reaction mixture was allowed to stir at 80 °C for 6 h. Upon completion, it was quenched by a saturated NH_4Cl solution and extracted with EtOAc, and the combined organic layer was dried over $MgSO_4$. The solvent was removed *in vacuo* to give the residue. To the residue was added 5-bromovaleric acid (1 equiv), EDCI·HCl (2.5 equiv), and DCM (2 mL). The reaction mixture was allowed to stir at room temperature for 12 h. Upon completion, it was quenched by a saturated NH_4Cl solution and extracted with EtOAc, and the combined organic layer was dried over $MgSO_4$. The solvent was removed *in vacuo*, and the obtained residue was further purified by silica gel column chromatography (200-300 mesh silica gel).

2.25 General procedure for the synthesis of sulfonyl BCP-sulfoxide

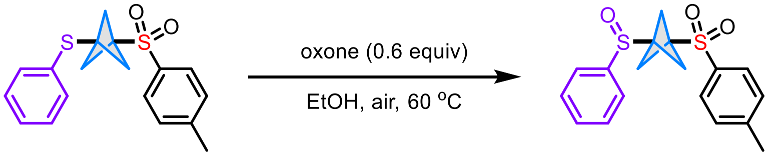

49 **102**

A 25 mL reaction tube equipped with a magnetic stirring bar was charged with sulfonyl BCP-thioether (**49**) (0.2 mmol), oxone (0.6 equiv), and EtOH (2 mL). The reaction mixture was allowed to stir at 60 °C for 12 h. Upon completion, it was quenched by a saturated NH_4Cl solution and extracted with EtOAc, and the combined organic layer was dried over $MgSO_4$. The solvent was

removed *in vacuo*, and the obtained residue was further purified by silica gel column chromatography (200-300 mesh silica gel).

2.26 General procedure for the synthesis of disulfonyl BCP

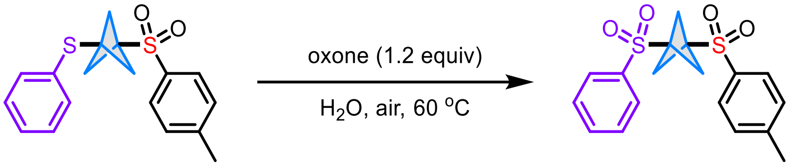

49 **103**

A 25 mL reaction tube equipped with a magnetic stirring bar was charged with sulfonyl BCP-thioether (**49**) (0.2 mmol), oxone (1.2 equiv), and H_2O (2 mL). The reaction mixture was allowed to stir at 60 °C for 12 h. Upon completion, it was quenched by a saturated NH_4Cl solution and extracted with EtOAc, and the combined organic layer was dried over $MgSO_4$. The solvent was removed *in vacuo*, and the obtained residue was further purified by silica gel column chromatography (200-300 mesh silica gel).

2.27 General procedure for the synthesis of sulfonyl BCP-azo

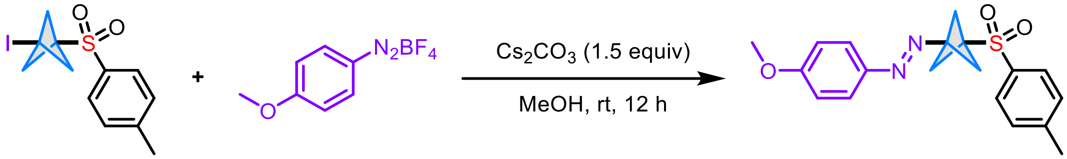

84 **54**

A 25 mL reaction tube equipped with a magnetic stirring bar was charged with sulfonyl BCP-I (**84**) (0.2 mmol), 4-methoxybenzenediazonium tetrafluoroborate (2 equiv), Cs_2CO_3 (1.5 equiv), and MeOH (2 mL). The reaction mixture was allowed to stir at 60 °C for 12 h. Upon completion, it was quenched by a saturated NH_4Cl solution and extracted with EtOAc, and the combined organic layer was dried over $MgSO_4$. The solvent was removed *in vacuo*, and the obtained residue was further purified by silica gel column chromatography (200-300 mesh silica gel).

2.28 General procedure for the synthesis of sulfonyl BCP-amine

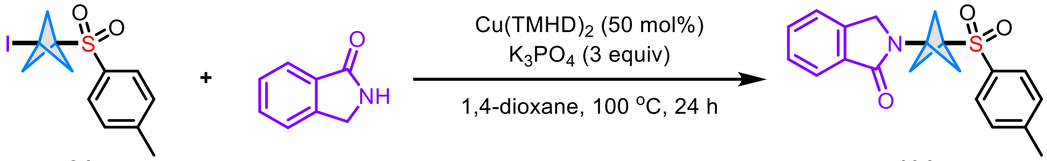

84 **104**

A 25 mL reaction tube equipped with a magnetic stirring bar was charged with sulfonyl BCP-I (**84**) (0.2 mmol), isoindolin-1-one (2 equiv), Cu(TMHD)$_2$ (50 mol%), K_3PO_4 (3 equiv), and 1,4-dioxane (2 mL). The reaction mixture was allowed to stir at 100 °C for 24 h. Upon completion, it was quenched by a saturated NH_4Cl solution and extracted with EtOAc, and the combined organic layer was dried over $MgSO_4$. The solvent was removed *in vacuo*, and the obtained residue was further purified by silica gel column chromatography (200-300 mesh silica gel).

2.29 General procedure for the synthesis of sulfonyl BCP-ketone

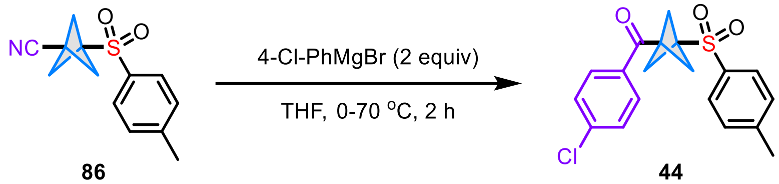

86 44

A 25 mL reaction tube equipped with a magnetic stirring bar was charged with sulfonyl BCP-CN (**86**) (0.2 mmol), and THF (3 mL). The reaction mixture was then cooled to 0 °C using an ice-water bath. Subsequently, 4-Cl-PhMgBr (2 equiv) was added to the tube dropwise via a syringe. Then, the reaction mixture was allowed to stir at 70 °C for 2 h. Upon completion, it was quenched by a saturated NH_4Cl solution and extracted with EtOAc, and the combined organic layer was dried over $MgSO_4$. The solvent was removed *in vacuo*, and the obtained residue was further purified by silica gel column chromatography (200-300 mesh silica gel).

2.30 General procedure for the synthesis of sulfonyl BCP-alkylamine

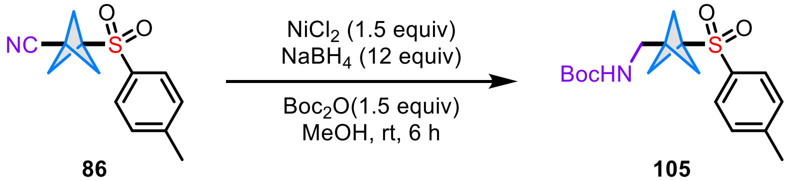

86 105

A 25 mL reaction tube equipped with a magnetic stirring bar was charged with sulfonyl BCP-CN (**86**) (0.2 mmol), $NiCl_2$ (1.5 equiv), Boc_2O (1.5 equiv), and MeOH (5 mL). The reaction mixture was cooled to 0 °C using an ice-water bath, and $NaBH_4$ (12 equiv) was then added over 1 min. Then, the reaction mixture was allowed to stir at room temperature for 6 h. Upon completion, it was quenched by a saturated NH_4Cl solution and extracted with EtOAc, and the combined organic layer was dried over $MgSO_4$. The solvent was removed *in vacuo*, and the obtained residue was further purified by silica gel column chromatography (200-300 mesh silica gel).

2.31 General procedure for the synthesis of sulfonyl BCP-COOH

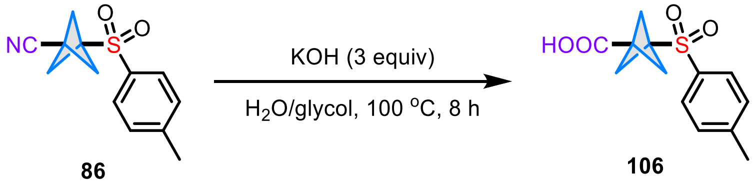

86 106

A 25 mL reaction tube equipped with a magnetic stirring bar was charged with sulfonyl BCP-CN (**86**) (0.2 mmol), KOH (3 equiv), and H_2O/glycol (2 mL, v:v = 1:1). The reaction mixture was allowed to stir at 100 °C for 8 h. Upon completion, it was quenched by a saturated NH_4Cl solution and extracted with EtOAc, and the combined organic layer was dried over $MgSO_4$. The solvent was removed *in vacuo*, and the obtained residue was further purified by silica gel column chromatography (200-300 mesh silica gel).

2.32 General procedure for the synthesis of sulfonyl BCP-amide

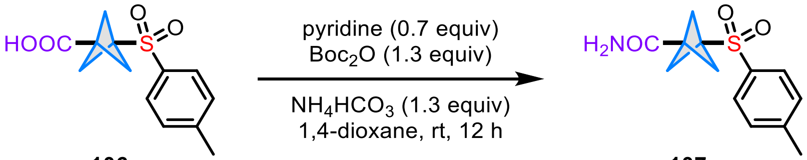

106 107

A 25 mL reaction tube equipped with a magnetic stirring bar was charged with sulfonyl BCP-COOH (**106**) (0.2 mmol), pyridine (0.7 equiv), Boc_2O (1.3 equiv), NH_4HCO_3 (1.3 equiv), and 1,4-dioxane (2 mL). The reaction mixture was allowed to stir at room temperature for 12 h. Upon completion, it was quenched by a saturated NH_4Cl solution and extracted with EtOAc, and the combined organic layer was dried over $MgSO_4$. The solvent was removed *in vacuo*, and the obtained residue was further purified by silica gel column chromatography (200-300 mesh silica gel).

2.33 General procedure for the synthesis of sulfonyl BCP-ester

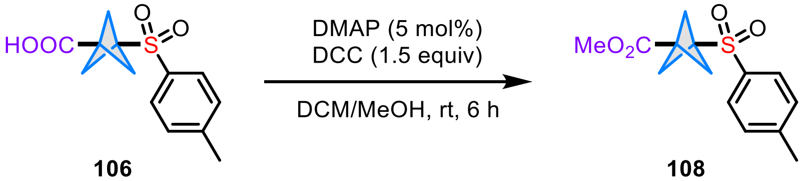

106 108

A 25 mL reaction tube equipped with a magnetic stirring bar was charged with sulfonyl BCP-COOH (**106**) (0.2 mmol), DMAP (5 mol%), DCC (1.5 equiv), and DCM/MeOH (2 mL, v:v = 1:1). The reaction mixture was allowed to stir at room temperature for 6 h. Upon completion, it was quenched by a saturated NH_4Cl solution and extracted with EtOAc, and the combined organic layer was dried over $MgSO_4$. The solvent was removed *in vacuo*, and the obtained residue was further purified by silica gel column chromatography (200-300 mesh silica gel).

3.34 X-ray crystal data for products 20 and 22

Single crystal of **20** ($C_{16}H_{16}BrN_3O_4S$), was recrystallized from mixture solvents of dichlormethane and diethyl ether. The obtained crystal structures have been deposited at the Cambridge Crystallographic Data Centre and allocated the deposition number: 2544084. The crystallographic data and refinement parameters of them are listed in Table S3.

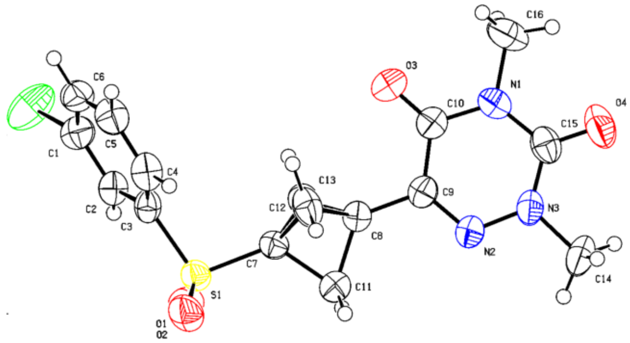

Fig. S2. Single-crystal X-ray structure of 20 is determined.

Table S3. Crystallographic data and structure refinement for **20**.

Identification code	2544084
Empirical formula	$C_{16}H_{16}BrN_3O_4S$
Formula weight	426.29
Temperature, K	289
Wavelength, Å	1.54178
Space group	Pbca
Hall group	-P 2ac 2ab
a, b, c, Å	7.708 (2), 16.085 (5), 27.946 (8)
α, β, γ, °	90, 90, 90
Volume, Å^3	3464.7 (18)
Z	8
Calculated density, mg/m^3	1.634
Absorption coefficient, mm^{-1}	4.607
F (000)	1728.0
F (000)’	1729.67
h, k, l max	9, 19, 33
Nref	3088
T min, T max	0.606, 0.753
T min’	0.379
wR2 (reflections)	0.2252 (3088)

Single crystal of **22** ($C_{16}H_{15}F_2N_3O_4S$), was recrystallized from mixture solvents of dichlormethane and diethyl ether. The obtained crystal structures have been deposited at the Cambridge Crystallographic Data Centre and allocated the deposition number: 2544085. The crystallographic data and refinement parameters of them are listed in Table S4.

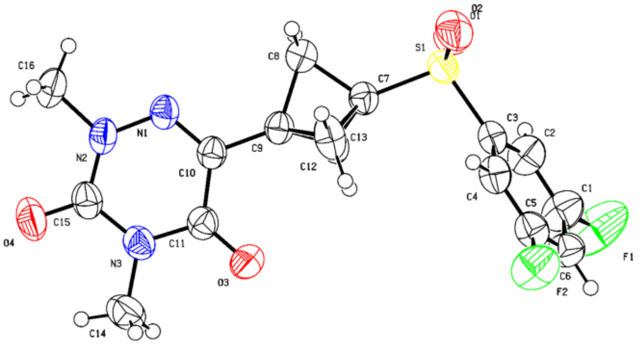

Fig. S3. **Single-crystal X-ray structure of 22 is determined.**

Table S4. Crystallographic data and structure refinement for **22**.

Identification code	2544085
Empirical formula	$C_{16}H_{15}F_2N_3O_4S$
Formula weight	383.37
Temperature, K	289
Wavelength, Å	1.54178
Space group	Pbca
Hall group	-P 2ac 2ab
a, b, c, Å	7.8102 (3), 15.8625 (7), 27.9897(13)
α, β, γ, °	90, 90, 90
Volume, Å^3	3467.6 (3)
Z	8
Calculated density, mg/m^3	1.469
Absorption coefficient, mm^{-1}	2.111
F (000)	1584.0
F (000)’	1592.22
h, k, l max	9, 19, 33
Nref	3132
T min, T max	0.612, 0.753
T min’	0.624
wR2 (reflections)	0.1092 (3132)

3. Mechanistic studies

3.1 Radical scavenging experiment

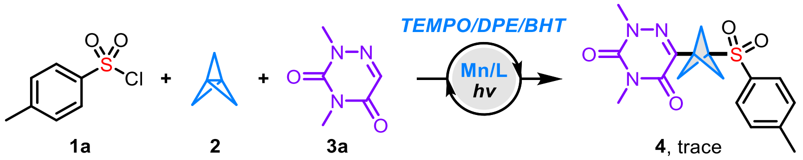

A 25 mL reaction tube equipped with a magnetic stirring bar was charged with tosyl chloride (**1a**) (1.5 equiv), [1.1.1]propellane (**2**) (1 equiv), azauracil (**3a**) (0.2 mmol), $Mn_2(CO)_{10}$ (5 mmol%), L (10 mol%), DIPEA (2 equiv), radical inhibitors (2 equiv), and EA (2 mL). The reaction mixture was allowed to stir with the irradiation of blue LEDs at room temperature under a nitrogen atmosphere for 12 h. Upon completion, trace yield of desired product (**4**) was detected. These results indicated that a radical mechanism might be operative.

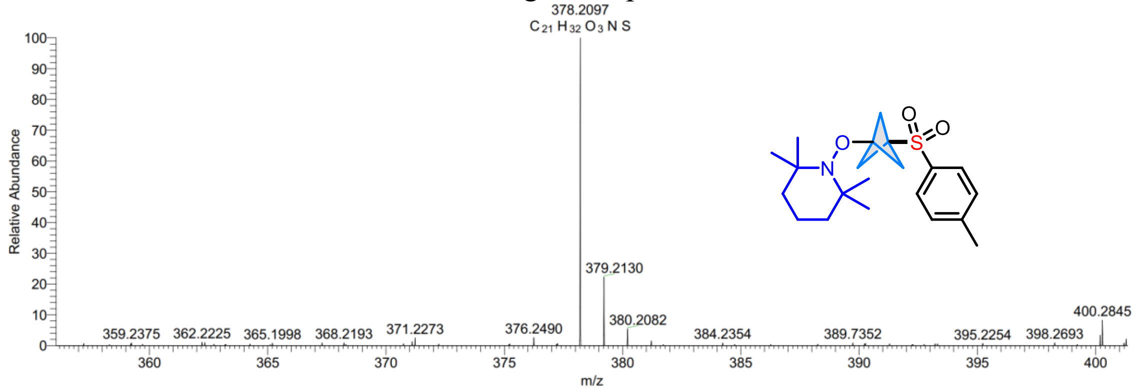

Fig. S4. High resolution mass spectrometer (HRMS) analysis of radical adduct 110 is presented.

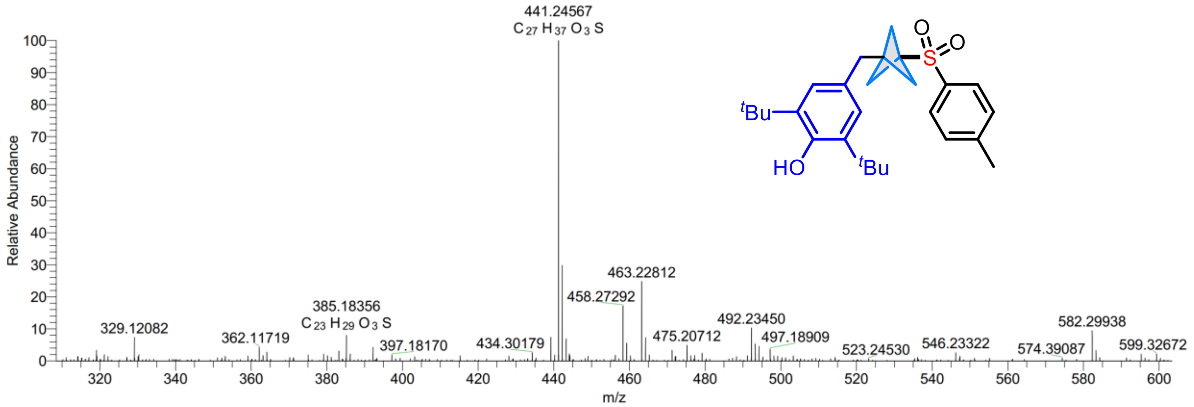

Fig. S5. HRMS analysis of radical adduct 111 is presented.

3.2 Importance of photoinduced Mn-catalysed system

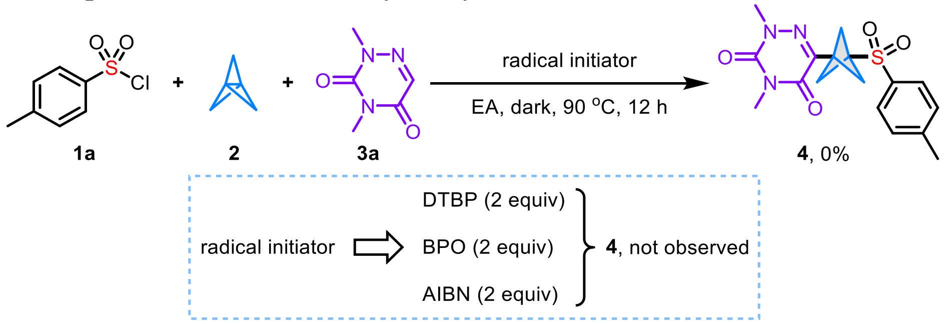

A 25 mL reaction tube equipped with a magnetic stirring bar was charged with tosyl chloride (**1a**) (1.5 equiv), [1.1.1]propellane (**2**) (1 equiv), azauracil (**3a**) (0.2 mmol), DIPEA (2 equiv), radical initiator (2 equiv), and EA (2 mL). The reaction mixture was allowed to stir in dark at 90 °C under a nitrogen atmosphere for 12 h. Upon completion, no desired product (**4**) was detected. These results indicated the importance of photoinduced Mn-catalyzed system.

3.3 Turn on/off profile experiment

The photoinduced manganese-catalysed difunctionalization of [1.1.1]propellane with and without light was carried out. It was found that the formation of product (**4**) needed continuous irradiation of light.

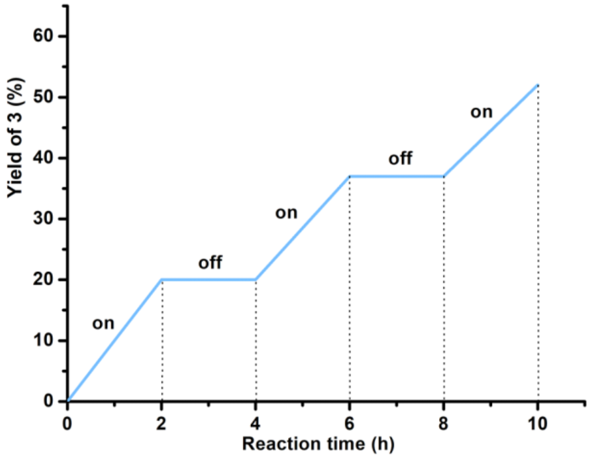

Fig. S6. Time profile of the photocatalytic reaction with and without light is shown.

3.4 UV-vis spectroscopic measurements

The UV-vis experiments were performed on an Agilent Cary 100 UV-visible spectrophotometer with a quartz cuvette (10 mm path length). The reaction mixture of $Mn_2(CO)_{10}$ and L4 was stirred in methylbenzene and irradiated by using blue LEDs at room temperature for 1 h. After completion of the reaction, the mixture was filtered to give the crude $(L4)_2Mn_2(CO)_6$ which was measured in EA with 50 µM concentration. $Mn_2(CO)_{10}$ and L4 are measured in EA with 50 µM concentration. The full spectra were collected, and we observed that the optical absorption spectrum of in situ formed $(L4)_2Mn_2(CO)_6$ generated a bathochromic shift compared to the spectra of $Mn_2(CO)_{10}$, which indicated that the $(L4)_2Mn_2(CO)_6$ complex could be an effective complex for the generation of manganese radicals under blue LEDs irradiation.

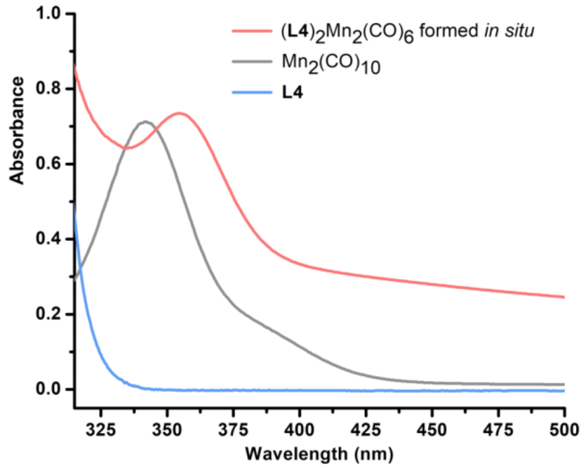

Fig. S7. UV/vis absorption spectrum is presented.

3.5 The total model reaction profile with/without xantphos ligand

The photoinduced manganese-catalysed difunctionalization of [1.1.1]propellane with and without ligand was carried out. After 0, 0.5, 1, 1.5, 2, 2.5, 3, 3.5, 4, 4.5, 5, 5.5, 6, 8, 12 h, the samples were taken from paralleled reaction mixtures in different reaction tubes. Along with the reaction time prolonging, the obvious yield difference of desired product (**4**) was observed, indicating the xantphos (L4) ligand plays a positive promotion in the whole reaction.

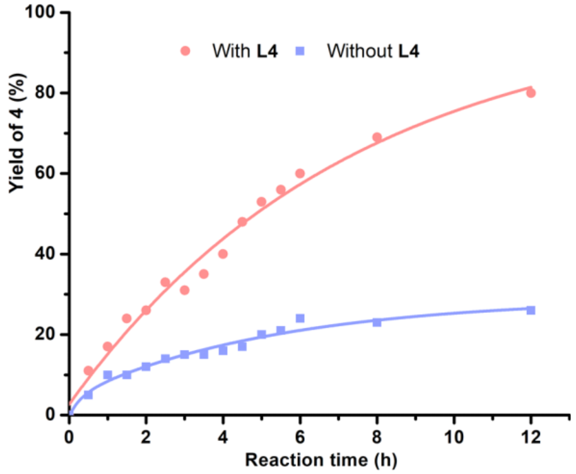

Fig. S8. Total model reaction profile with/without the xantphos ligand is presented.

3.6 Density functional theory (DFT) calculations

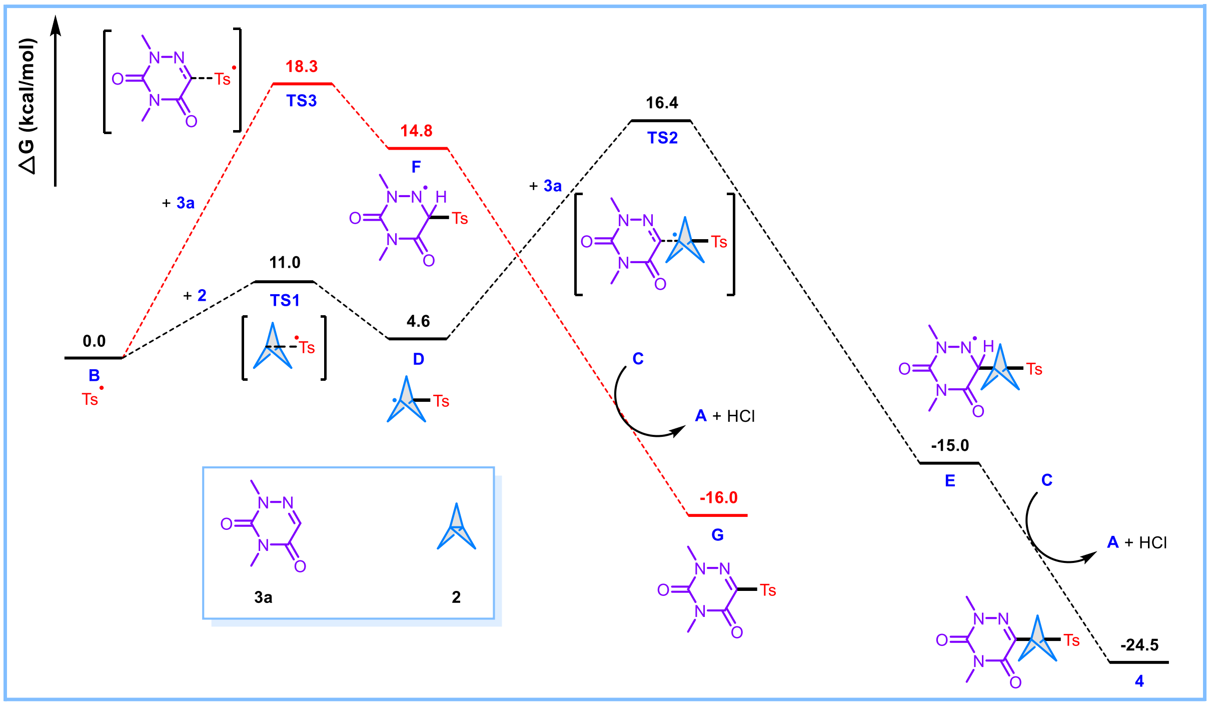

Fig. S9. Density functional theory (DFT) calculations are presented.

The geometries were optimized at wb97xd/6-31g(d) theoretical level by GAUSSIAN 09. The frequencies of all the stationary species are positive, while the transition state has only one imaginary frequency. The SMD implicit solvation model with dichloroethane as the solvent was used to account for the solvation effect when performing single point energy calculations using the same DFT functional. GAUSSIAN 09 was used to calculate the Gibbs free energy of all the optimized structures at 298.15K. Gibbs free energy of equilibrium state and transition state at the wb97xd/6-31g(d)/SMD(1,2-dichloroethane) level of theory and 298.15 K calculated by GAUSSIAN 09.

***p*-Toluenesulfonyl radical**

C	1.69840600	-1.20237200	-0.00098600
C	2.41061900	0.00042600	-0.03339800
C	1.69827100	1.20295300	-0.00094600
C	0.31077200	1.21503800	0.06992700
C	-0.36541500	0.00012000	0.09168200
C	0.31107400	-1.21477900	0.06994200
H	2.23716300	-2.14534500	-0.03602600
H	2.23674400	2.14606300	-0.03597000
H	-0.24151300	2.14849900	0.08468000
H	-0.24105000	-2.14833300	0.08473300
C	3.91679300	-0.00026900	-0.07601300
H	4.33118800	-0.01892700	0.93892600

H	4.30078200	-0.87804700	-0.60374200
H	4.30316100	0.89416200	-0.57295500
S	-2.15632600	-0.00010600	0.24215200
O	-2.64409200	-1.28984100	-0.27841600
O	-2.64445400	1.28945500	-0.27849900

[1.1.1]propellane

C	0.00011900	0.00005700	0.78296000
C	-0.00007600	-0.00009000	-0.78278100
C	1.04071700	-0.77189700	-0.00010300
H	2.05669000	-0.38481700	-0.00030600
H	0.96588500	-1.85660500	0.00001100
C	0.14809000	1.28708900	-0.00017400
H	-0.69429500	1.97454200	-0.00006800
H	1.12523600	1.76367700	-0.00030900
C	-1.18882500	-0.51518900	0.00013800
H	-1.36272200	-1.58845900	0.00025500
H	-2.09093600	0.09184400	0.00017900

2,4-dimethyl-1,2,4-triazine-3,5(2H,4H)-dione

C	0.39851000	-1.79298400	-0.00030500
C	-0.56032200	0.78474800	-0.00053000
C	1.38852500	-0.70792600	-0.00005500
H	0.76568400	-2.81239100	-0.00038300
N	-0.87504900	-1.61771400	-0.00027300
N	0.81072600	0.56220500	-0.00007700
C	1.66432300	1.74557700	0.00028100
H	1.46136000	2.34697800	-0.88789200
H	1.45903300	2.34807400	0.88715700
H	2.69670800	1.40149800	0.00178900
O	2.58960300	-0.89050200	-0.00011300
N	-1.34532100	-0.35932700	-0.00027100
C	-2.79174200	-0.19081000	0.00039900
H	-3.10746200	0.36059300	0.88881000
H	-3.10783200	0.36300400	-0.88636000
H	-3.22722800	-1.18857000	-0.00084800
O	-1.04816700	1.89713000	0.00053000

TS1

C	2.54920600	-0.16626400	-1.20069300
C	3.19210900	-0.47297900	0.00302500

C	2.55335200	-0.15136100	1.20343500
C	1.30523200	0.46148800	1.21132700
C	0.69532400	0.76301900	-0.00122400
C	1.30233400	0.44619100	-1.21270300
H	3.03513100	-0.40694000	-2.14276200
H	3.04137600	-0.37982400	2.14725100
H	0.81211700	0.71760500	2.14333800
H	0.80657900	0.69128900	-2.14628800
C	4.55733000	-1.11177400	-0.00009300
H	4.64848300	-1.85385000	-0.79930300
H	5.33619900	-0.35730300	-0.16168600
H	4.76907400	-1.60741200	0.95158900
S	-0.97030400	1.42107000	-0.00308700
O	-1.17890700	2.10602100	-1.28905600
O	-1.17813300	2.11510100	1.27813300
C	-2.05398300	-0.70475000	0.00249200
C	-2.67434200	-2.19030100	0.00455100
C	-1.76813500	-1.69111400	-1.11518600
H	-0.76223400	-2.10285400	-1.16649200
H	-2.22789900	-1.49172600	-2.08052800
C	-3.55106800	-0.94211800	0.00670500
H	-4.08677500	-0.70909800	0.92407600
H	-4.09184600	-0.70941500	-0.90776600
C	-1.76186300	-1.69058800	1.11898300
H	-0.75569200	-2.10229900	1.16484400
H	-2.21630600	-1.49095800	2.08678700

***p*-Toluenesulfonyl radical-[1.1.1]propellane**

C	2.62564900	0.09695700	1.20455800
C	3.29581300	0.34494000	0.00375200
C	2.62429800	0.11136600	-1.20087600
C	1.31699600	-0.35698300	-1.21306000
C	0.67379900	-0.59410400	-0.00139800
C	1.31700000	-0.37165400	1.21161300
H	3.13599700	0.26853700	2.14837500
H	3.13489100	0.29460800	-2.14259100
H	0.79945900	-0.55214800	-2.14664600
H	0.80003200	-0.57747100	2.14322500
C	4.72413700	0.82586000	0.00068400
H	4.99223200	1.28947400	0.95416400

H	5.41313100	-0.00997600	-0.16783300
H	4.89565600	1.55756900	-0.79486000
S	-1.02700200	-1.12395900	-0.00412600
O	-1.31086800	-1.78596000	1.26613100
O	-1.30949500	-1.77668100	-1.27950800
C	-1.91642600	0.46059800	0.00134200
C	-2.82263900	1.98442400	0.00690800
C	-1.82520700	1.53949700	1.10172600
H	-0.88086900	2.08265100	1.16848300
H	-2.23652700	1.26857800	2.07468300
C	-3.45798600	0.57576100	0.00173800
H	-3.96166400	0.25481100	-0.91047900
H	-3.96158000	0.24809400	0.91161000
C	-1.82517200	1.54747900	-1.09116300
H	-0.88084900	2.09114200	-1.15395700
H	-2.23653900	1.28375900	-2.06607900

TS2

C	-3.49211500	1.62057000	-1.18181100
C	-3.54816600	2.41303300	-0.02982100
C	-3.24708000	1.82802000	1.20259800
C	-2.89154200	0.48706200	1.29183200
C	-2.84077400	-0.27553200	0.12988200
C	-3.14033600	0.27928500	-1.11185100
H	-3.72874800	2.06223900	-2.14617900
H	-3.29082700	2.42964600	2.10628400
H	-2.66587200	0.02814800	2.24885900
H	-3.10717400	-0.33995100	-2.00263400
C	-3.95000400	3.86248200	-0.12168900
H	-5.03511700	3.95598800	-0.24551900
H	-3.66886400	4.41413400	0.77955700
H	-3.47980700	4.34991000	-0.98146600
S	-2.28293400	-1.96383000	0.21901700
O	-2.79751600	-2.69362600	-0.93557800
O	-2.48307300	-2.45486700	1.57909300
C	-0.49878300	-1.75645600	-0.02346700
C	1.24860300	-1.57254900	-0.25805000
C	0.17140200	-1.16314600	-1.28326800
H	0.02343500	-0.09388300	-1.44630500
H	0.10230800	-1.75155300	-2.19775900

C	0.52932600	-2.91436400	-0.01527200
H	0.69139000	-3.40236300	0.94604800
H	0.47348000	-3.61130500	-0.85176200
C	0.43523300	-0.91661800	0.87748900
H	0.29698800	0.16620500	0.83648000
H	0.59640500	-1.29340200	1.88814100
C	3.56204400	-1.01454000	-0.36771400
C	2.86625400	1.51050400	0.47629300
C	3.15813700	-0.04093200	-1.39817400
H	3.90253200	-1.98478500	-0.70879000
N	3.80343500	-0.70249500	0.87294400
N	2.79694600	1.19696900	-0.87939100
C	2.29783300	2.19921800	-1.81248700
H	2.04158400	3.09031500	-1.24378800
H	3.06550300	2.42415300	-2.55545800
H	1.41865800	1.80996200	-2.33157500
O	3.11240500	-0.27672000	-2.58899000
O	2.47936000	2.56716100	0.93962500
N	3.42918800	0.53198700	1.27618500
C	3.56943000	0.81945300	2.69578100
H	4.11168900	1.75589300	2.83819000
H	2.58687200	0.91035900	3.16731600
H	4.11933100	-0.01217300	3.13308900

***p*-Toluenesulfonyl radical-[1.1.1]propellane + 2,4-dimethyl-1,2,4-triazine-3,5(2H,4H)-dione**

C	-4.54165100	1.41983200	-0.92580800
C	-4.72549500	2.04386800	0.31296800
C	-4.21765900	1.42545400	1.45758400
C	-3.53985900	0.21400400	1.37658200
C	-3.37025200	-0.38301700	0.13239600
C	-3.86797000	0.21021000	-1.02493000
H	-4.93730200	1.88749400	-1.82363000
H	-4.35765300	1.89575500	2.42706500
H	-3.15628200	-0.27446100	2.26642600
H	-3.73607500	-0.28317900	-1.98238900
C	-5.47914700	3.34600000	0.40258700
H	-5.35308700	3.81608600	1.38181500
H	-5.13953700	4.05333800	-0.36083800
H	-6.55163000	3.18403300	0.24491000
S	-2.44781500	-1.90292100	0.00821100

O	-2.84882100	-2.58674700	-1.21956400
O	-2.49767000	-2.58322600	1.30044200
C	-0.78397800	-1.31287300	-0.22881800
C	0.95505400	-0.75208300	-0.48213800
C	-0.27066800	-0.41152400	-1.37419500
H	-0.59976500	0.62857700	-1.36779900
H	-0.28909200	-0.85474100	-2.37353800
C	0.44930100	-2.22035800	-0.44655800
H	0.76406100	-2.82616300	0.40472400
H	0.47566300	-2.77421900	-1.38823000
C	0.05801000	-0.47427600	0.75840900
H	-0.25513400	0.56188600	0.90724200
H	0.34046800	-0.97064200	1.68960300
C	2.39088500	-0.32804200	-0.68466000
C	4.83937400	0.69062200	0.20376300
C	2.56190800	1.17971300	-0.54263800
N	3.22984700	-1.14338300	0.16898800
N	3.84159500	1.57876800	-0.19742900
C	4.18820600	2.99759100	-0.19061600
H	3.34309200	3.54125100	-0.60786500
H	4.38133500	3.33461800	0.83032000
H	5.08516000	3.15707500	-0.79014100
O	1.67575100	1.97136100	-0.78192400
O	5.97230500	1.05629600	0.44290900
N	4.45175100	-0.63277300	0.37336200
C	5.42592300	-1.52876500	0.98803200
H	6.27341100	-1.67738900	0.31514000
H	5.79247600	-1.10796000	1.92641300
H	4.91300500	-2.47290500	1.16250700
H	2.66516000	-0.54959500	-1.73421000

L1Mn(CO)$_3$-Cl

C	1.83278800	0.50055900	2.72953400
H	1.78239700	1.50794600	2.33064500
C	1.86769900	0.31598000	4.10829300
H	1.83213800	1.17944800	4.76569700
C	1.95637200	-0.96564800	4.64124800
H	1.99636000	-1.10716600	5.71746500
C	1.98275300	-2.06749900	3.78979500
H	2.03828100	-3.07267800	4.19707200

C	1.92320600	-1.88948700	2.41201800
H	1.90476600	-2.75386300	1.75609900
C	1.86824400	-0.59876300	1.86885900
C	-2.41197000	0.91505600	2.36039700
H	-2.83832900	1.71230000	1.76030000
C	-2.43902900	1.02500000	3.74637000
H	-2.87571800	1.90714400	4.20571900
C	-1.92091000	0.00459000	4.53916400
H	-1.94708400	0.08890500	5.62189700
C	-1.37334200	-1.12258000	3.93694500
H	-0.96212300	-1.92265500	4.54391400
C	-1.32676500	-1.22914500	2.54944400
H	-0.89068100	-2.10670100	2.08396100
C	-1.84699400	-0.20951200	1.74797100
C	0.13398200	-0.00921000	-2.40456900
C	-1.16633900	-2.29313000	-2.34724900
C	1.26419900	-2.41340000	-2.16883600
Mn	0.01991300	-1.40968000	-1.29927400
O	0.21950100	0.89338700	-3.11894700
O	-1.82824200	-2.90825600	-3.05454200
O	1.95792800	-3.12370400	-2.74201400
P	1.85244600	-0.40788500	0.04714800
P	-1.85998900	-0.41323000	-0.08197900
C	3.43012300	1.95828500	-0.44445400
C	2.17501100	1.39824500	-0.18853700
C	1.09105000	2.27143100	-0.15815800
C	1.16829900	3.63340600	-0.44232300
C	2.43111600	4.14251300	-0.73361700
C	3.55275300	3.31650400	-0.71540300
C	-0.11204800	4.46185800	-0.32836800
C	-1.28550400	3.57368400	-0.74267200
C	-1.22117800	2.22580100	-0.41099200
C	-2.24899100	1.30884500	-0.61323400
C	-3.43313700	1.79608300	-1.16593800
C	-3.53114300	3.13519500	-1.53660500
C	-2.46921800	4.01311900	-1.33407800
H	4.31396900	1.33116100	-0.45120400
H	2.55421400	5.19600300	-0.96199300
H	4.53238100	3.73621200	-0.92022700
H	-4.28088900	1.13586000	-1.30892500

H	-4.45199200	3.50206600	-1.97871800
H	-2.58137300	5.05278100	-1.62312200
C	-0.04221700	5.74320700	-1.16425500
H	0.10642700	5.52302900	-2.22607100
H	-0.96157900	6.32550600	-1.05321800
H	0.77417100	6.38664500	-0.82300300
C	-0.31013800	4.83381000	1.16200600
H	-1.23828500	5.40179200	1.28936200
H	-0.37044400	3.93636100	1.78660900
H	0.52831900	5.44424400	1.51513500
O	-0.10521000	1.71820200	0.19604900
C	-3.45801000	-1.27689100	-0.36542900
C	-4.00703600	-1.30459300	-1.65363100
C	-4.12575500	-1.94556000	0.66303800
C	-5.20329900	-1.96661500	-1.90386000
H	-3.50195300	-0.80134800	-2.47364100
C	-5.31751200	-2.61906100	0.40896700
H	-3.71756200	-1.94544400	1.66760500
C	-5.86197600	-2.62798500	-0.87065600
H	-5.61465800	-1.97295300	-2.90863300
H	-5.82069700	-3.13702700	1.21992500
H	-6.79376000	-3.15081100	-1.06449500
C	3.50003800	-1.05163300	-0.47605100
C	3.84671600	-0.88937100	-1.82447300
C	4.42476600	-1.64175500	0.38607800
C	5.07770100	-1.31911900	-2.30126100
H	3.15330800	-0.40680100	-2.50887000
C	5.65682400	-2.08305500	-0.09490100
H	4.19659300	-1.75933000	1.43925000
C	5.98578400	-1.92607100	-1.43613100
H	5.32378000	-1.18496200	-3.35006400
H	6.36100200	-2.54708000	0.58918400
H	6.94516300	-2.27290700	-1.80831000
Cl	-0.12788900	-3.35544100	0.14023000

***p*-Toluenesulfonyl radical-[1.1.1]propellane-2,4-dimethyl-1,2,4-triazine-3,5(2H,4H)-dione**

C	4.45226200	1.39000900	1.21280600
C	4.84334800	1.98933100	0.01103400
C	4.51073300	1.36060600	-1.19158900
C	3.80366600	0.16387500	-1.20320200

C	3.42693100	-0.40941100	0.00665900
C	3.74679700	0.19392400	1.21991700
H	4.70692500	1.86656900	2.15577700
H	4.81153200	1.81283400	-2.13272600
H	3.55579900	-0.33245000	-2.13583800
H	3.45578800	-0.28082600	2.15135400
C	5.63474100	3.27192600	0.01864500
H	5.55877100	3.79349900	-0.93959100
H	5.28771000	3.94953900	0.80477400
H	6.69622300	3.06985600	0.20375600
S	2.46032200	-1.90649500	0.00362100
O	2.65960600	-2.58894900	1.28017200
O	2.68040300	-2.59785900	-1.26462000
C	0.79445100	-1.27589800	-0.00969100
C	-0.95285100	-0.67337600	-0.01848700
C	0.13501500	-0.37875700	1.06115500
H	0.48587700	0.65306700	1.12259200
H	-0.01739700	-0.83550800	2.04212300
C	-0.48334400	-2.14978400	-0.00843300
H	-0.67165500	-2.73084700	-0.91351600
H	-0.67697800	-2.72032900	0.90218000
C	0.14161900	-0.39088800	-1.09474100
H	0.49283700	0.64012900	-1.16577700
H	-0.00479800	-0.85848600	-2.07146100
C	-2.35638700	-0.20318800	-0.01985900
C	-4.98464200	0.65468300	0.00797600
C	-2.60552200	1.25203700	-0.02879800
N	-3.29805500	-1.08257600	-0.00598100
N	-3.95456700	1.58901600	-0.01734800
C	-4.34947700	2.99408300	-0.01371600
H	-3.43958500	3.58789300	-0.07216300
H	-4.99781400	3.19552600	-0.86812900
H	-4.89616600	3.22210400	0.90349200
O	-1.71696300	2.08381600	-0.04314500
O	-6.15728100	0.96957600	0.03426400
N	-4.57664300	-0.66775900	-0.00170600
C	-5.60370800	-1.69951600	0.03902100
H	-6.14977500	-1.65638600	0.98471300
H	-6.31042700	-1.55656100	-0.78059800
H	-5.09509100	-2.65707400	-0.05921600

HCl

Cl	0.00000000	0.00000000	0.07134100
H	0.00000000	0.00000000	-1.21278900

L1Mn(CO)$_3$ radical

C	1.79354600	0.35226800	2.65700600
H	1.70278700	1.38749900	2.34479100
C	1.79907300	0.04497400	4.01588500
H	1.70030400	0.84215800	4.74648700
C	1.93572500	-1.27335500	4.43647200
H	1.94923100	-1.50803100	5.49687300
C	2.04808400	-2.29239000	3.49230000
H	2.15251000	-3.32540800	3.81113700
C	2.01692300	-1.99058100	2.13671100
H	2.08953600	-2.78964800	1.40323600
C	1.90548000	-0.66175000	1.70615400
C	-2.38221600	0.66535000	2.38056800
H	-2.77740400	1.52811200	1.85270800
C	-2.40982100	0.64134100	3.77197100
H	-2.81786600	1.48843200	4.31579300
C	-1.92340800	-0.46537600	4.46240200
H	-1.94644900	-0.48338300	5.54822900
C	-1.40500900	-1.54746400	3.75728900
H	-1.01261400	-2.40959500	4.28675300
C	-1.36306100	-1.51784100	2.36765200
H	-0.93609500	-2.35459900	1.82128900
C	-1.85444200	-0.41366100	1.66641200
C	-0.13422100	-1.06633300	-2.70547100
C	-1.00142600	-3.08960000	-0.96494300
C	1.30638600	-2.89045400	-1.38411600
Mn	0.06944000	-1.64946300	-1.01325700
O	-0.27430800	-0.70375600	-3.79739700
O	-1.62807500	-4.06398800	-0.95211800
O	2.02883700	-3.75974300	-1.63826800
P	1.85305100	-0.36282100	-0.10522000
P	-1.79741000	-0.48248500	-0.16861700
C	3.36087800	2.03463700	-0.64049600
C	2.14176500	1.45652100	-0.26934900
C	1.06129100	2.31712300	-0.11640800

C	1.09934200	3.68589200	-0.37850600
C	2.32398200	4.21839300	-0.77081300
C	3.44748400	3.40145600	-0.88148300
C	-0.19866400	4.47185900	-0.18584900
C	-1.33561200	3.56149300	-0.65118100
C	-1.22701900	2.20940800	-0.34210200
C	-2.18928400	1.25349800	-0.63844300
C	-3.34966400	1.69400100	-1.27795500
C	-3.49263600	3.03704000	-1.61614600
C	-2.49597900	3.96189400	-1.31079200
H	4.24162500	1.41536200	-0.76419300
H	2.41416600	5.27709200	-0.99068900
H	4.39852700	3.83361800	-1.17608300
H	-4.13561600	0.98857300	-1.52394500
H	-4.39339500	3.36792700	-2.12329800
H	-2.63587200	5.00222000	-1.58578800
C	-0.18118300	5.80269500	-0.94123800
H	-0.04227900	5.65353600	-2.01684400
H	-1.11687800	6.34723500	-0.78218900
H	0.62238600	6.44689500	-0.57138500
C	-0.38817800	4.74717700	1.32580700
H	-1.33228100	5.27592400	1.49670500
H	-0.40958900	3.81364400	1.89691100
H	0.43409700	5.36465300	1.70354000
O	-0.11055100	1.74869800	0.30535800
C	-3.40323000	-1.27314500	-0.59571900
C	-3.63716600	-1.61798300	-1.93252700
C	-4.40443300	-1.50387900	0.34943600
C	-4.85191600	-2.17468300	-2.31592200
H	-2.86405900	-1.45296700	-2.67764400
C	-5.61626100	-2.07381700	-0.03442800
H	-4.24111000	-1.24577500	1.39076600
C	-5.84386500	-2.40669100	-1.36568000
H	-5.01917100	-2.43506500	-3.35663100
H	-6.38332200	-2.25648000	0.71240400
H	-6.78924100	-2.85110800	-1.66262000
C	3.50723700	-0.94082800	-0.66689600
C	3.71341900	-1.07647800	-2.04437600
C	4.57299100	-1.17594000	0.20459200
C	4.95825200	-1.44595800	-2.53997600

H	2.89166800	-0.89810900	-2.73313700
C	5.81775900	-1.55556800	-0.29250500
H	4.43579700	-1.06196800	1.27511400
C	6.01286500	-1.69106400	-1.66344300
H	5.10127200	-1.55401300	-3.61069600
H	6.63564900	-1.74388600	0.39681700
H	6.98314000	-1.98944600	-2.04940500

TS3

C	-4.07750400	-1.02139000	-0.19700300
C	-4.49424800	0.31215800	-0.27821400
C	-3.57383900	1.32203200	0.01275800
C	-2.26718500	1.01986800	0.37774800
C	-1.88799500	-0.31574300	0.45675300
C	-2.77606600	-1.34613700	0.16018500
H	-4.78548600	-1.81664900	-0.41462500
H	-3.88405400	2.36180100	-0.04269500
H	-1.55466500	1.80321200	0.61111900
H	-2.45180300	-2.37916600	0.22927800
C	-5.90748900	0.64214500	-0.68439500
H	-6.62882300	0.03824400	-0.12465500
H	-6.13819500	1.69658700	-0.51054600
H	-6.06403100	0.43608100	-1.74940200
S	-0.19354000	-0.70722800	0.84509900
O	-0.10595900	-2.12833000	1.19540400
O	0.33322800	0.33796700	1.73435300
C	0.83296200	-0.19126600	-1.16921400
C	3.09177900	0.81538600	0.02083700
C	1.99645600	-1.10450200	-1.02542600
H	0.04391400	-0.54437300	-1.82621900
N	0.94306500	1.13197200	-1.07106200
N	3.05551000	-0.52762200	-0.33624600
C	4.21410200	-1.33742500	0.02702300
H	4.38347700	-1.26164600	1.10185000
H	3.99386600	-2.36491700	-0.25583800
H	5.10227800	-0.98161600	-0.49950800
O	2.00000500	-2.24455100	-1.43360100
N	2.02301500	1.58975800	-0.43399700
O	4.00928000	1.30485000	0.64296800
C	2.01722400	3.00145900	-0.08054600

H	1.79984700	3.11356700	0.98505900
H	2.99028500	3.44298100	-0.29645200
H	1.23726400	3.47377800	-0.67584700

***p*-Toluenesulfonyl radical + 2,4-dimethyl-1,2,4-triazine-3,5(2H,4H)-dione**

C	-3.98712600	-0.98923400	-0.37417200
C	-4.41134000	0.33467900	-0.21154100
C	-3.49544200	1.28169200	0.25205000
C	-2.18403000	0.92749100	0.54637500
C	-1.79267400	-0.39530900	0.37359000
C	-2.68240000	-1.36317800	-0.08603800
H	-4.69261200	-1.73741500	-0.72545400
H	-3.81282700	2.31145700	0.38986400
H	-1.47561600	1.65993600	0.91662900
H	-2.35502300	-2.39181600	-0.19691700
C	-5.83403600	0.71616700	-0.52985700
H	-6.53816000	0.12980100	0.07010200
H	-6.02011700	1.77506400	-0.33207200
H	-6.06232200	0.52365800	-1.58382600
S	-0.11559300	-0.86332000	0.72444200
O	-0.05761500	-2.30692800	0.90936100
O	0.43145200	0.05719000	1.71806700
C	0.76101700	-0.46138900	-0.90370800
C	2.89122400	1.02161100	0.03726100
C	2.12502900	-1.12146200	-0.83845000
H	0.15967900	-0.95583300	-1.66805400
N	0.72214000	0.94444400	-1.08864000
N	3.08213600	-0.33250800	-0.22694200
C	4.38595800	-0.89596500	0.11055300
H	4.60125600	-0.69626700	1.16064000
H	4.33463600	-1.96719900	-0.07446700
H	5.16632200	-0.44666900	-0.50779100
O	2.33139800	-2.24072300	-1.24346100
N	1.72681700	1.58821300	-0.48469800
O	3.71600900	1.69441300	0.61630300
C	1.54988700	3.02302000	-0.31345300
H	1.27632600	3.23822400	0.72341200
H	2.47626900	3.54490300	-0.55315900
H	0.74768200	3.32388600	-0.98582200

p-Toluenesulfonyl radical-2,4-dimethyl-1,2,4-triazine-3,5(2H,4H)-dione

C	3.55165900	0.47401200	1.18239100
C	4.17209900	0.79312500	-0.03132800
C	3.59852300	0.33558500	-1.21930600
C	2.43030800	-0.41843700	-1.20723600
C	1.83472900	-0.71464200	0.01237600
C	2.38699900	-0.27883600	1.21375900
H	3.99393300	0.81584000	2.11448300
H	4.07525100	0.56536200	-2.16814200
H	1.99142600	-0.78748800	-2.12815500
H	1.91288300	-0.54105300	2.15372100
C	5.44140100	1.60564400	-0.04163600
H	6.24757000	1.07680100	0.47879800
H	5.77806700	1.81113800	-1.06130700
H	5.29818000	2.56453800	0.46806900
S	0.35002800	-1.69486700	0.03818200
O	0.26036700	-2.35821700	1.33066200
O	0.26758100	-2.43316700	-1.21295200
C	-0.95785100	-0.44515800	0.00308200
C	-2.91758200	1.48053600	-0.01815400
C	-2.35650000	-0.91832200	-0.01412800
N	-0.60680500	0.78665700	0.01254100
N	-3.27043000	0.13894800	-0.03099900
C	-4.70104300	-0.15615900	-0.05005600
H	-5.16028700	0.31955300	-0.91808400
H	-4.81064500	-1.23740400	-0.10176100
H	-5.16990700	0.23233300	0.85604600
O	-2.68843000	-2.08096300	-0.01351900
N	-1.54826400	1.73179300	0.00125500
O	-3.72854200	2.38181200	-0.02348800
C	-1.10067900	3.11883300	0.01970100
H	-1.45859800	3.64024200	-0.87033000
H	-1.48784400	3.62538400	0.90613900
H	-0.01217000	3.09802800	0.03761000

3.7 General procedure for the synthesis of $(L4)_2Mn_2(CO)_6$

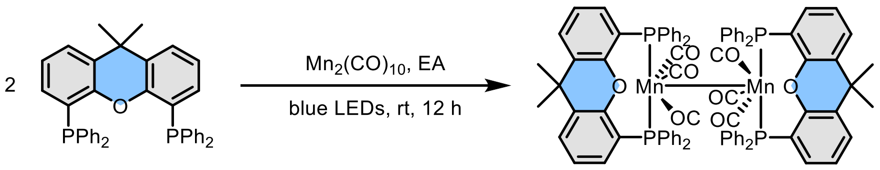

To an oven-dried sealed tube, $Mn_2(CO)_{10}$ (0.2 mmol), xantphos (L4) (1.1 equiv) and EA (2 mL) were added and the mixture was vigorously stirred under the irradiation of blue LEDs at room temperature for 12 h at the atmosphere of nigrogen. After the reaction was finished, the solvent was removed under vacuum, and the resulting residue was purified by recrystallization using dichloromethane (DCM) and *n*-hexane as solvents to afford the product in 16% yield as a yellow powder. ^{1}H NMR (600 MHz, $CDCl_3$) δ 8.02 (s, 8H), 7.50 (d, *J* = 7.8 Hz, 8H), 7.35 (s, 8H), 7.30 (s, 12H), 7.22 (d, *J* = 5.6 Hz, 12H), 7.18 (t, *J* = 7.4 Hz, 4H), 1.63 (s, 6H), 1.55 (s, 6H); ^{13}C NMR (151 MHz, $CDCl_3$) δ 153.5, 136.2, 136.0, 135.9, 134.2, 134.2, 134.1, 132.8, 130.9, 130.9, 130.8, 130.8, 130.7, 130.5, 129.2, 129.1, 128.4, 127.3, 127.3, 127.3, 127.1, 127.1, 127.0, 124.1, 121.2, 121.1, 121.0, 34.6, 33.1, 30.6; ^{31}P NMR (243 MHz, $CDCl_3$) δ 62.1.

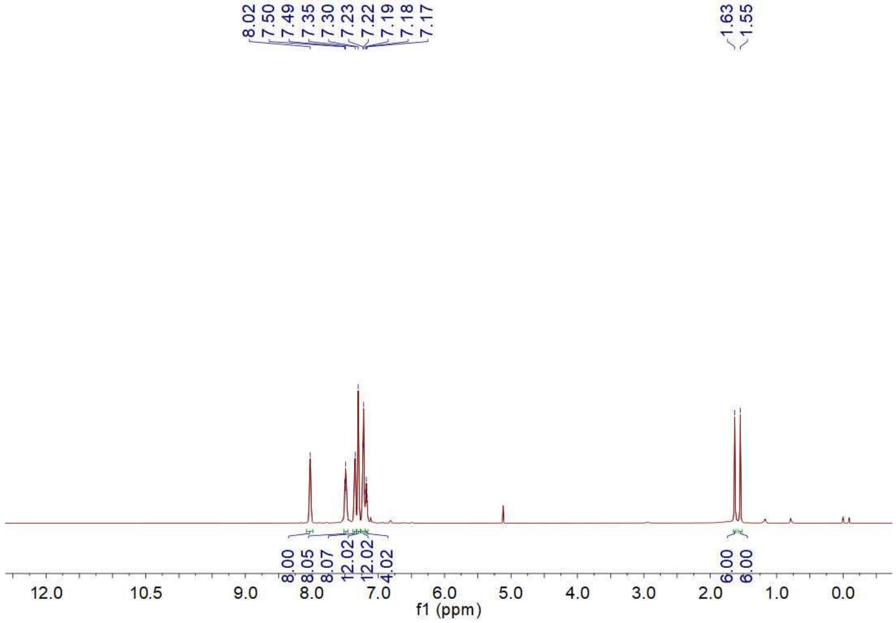

Fig. S10. ^{1}H NMR spectrum of $(L4)_2Mn_2(CO)_6$ is presented.

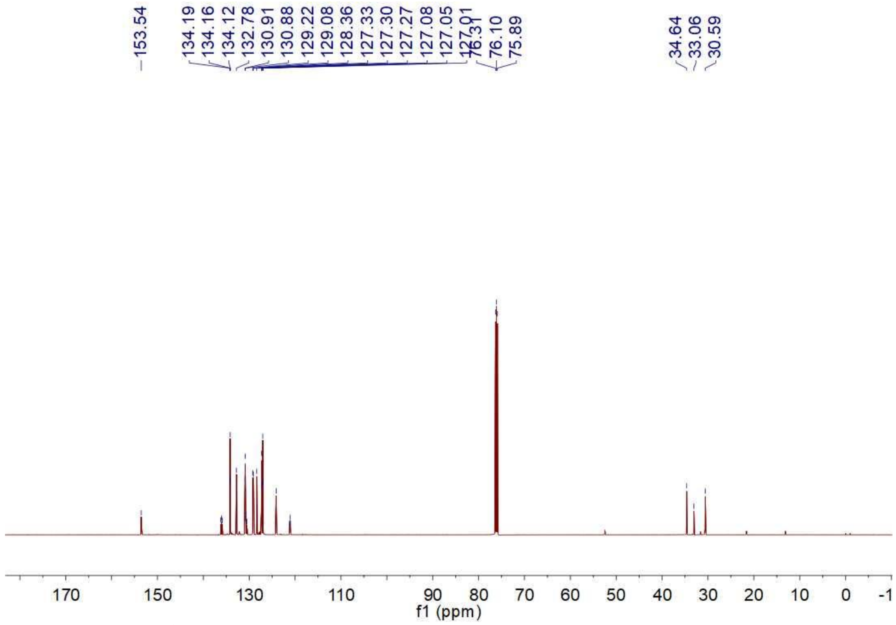

Fig. S11. ^{13}C NMR spectrum of $(L4)_2Mn_2(CO)_6$ is presented.

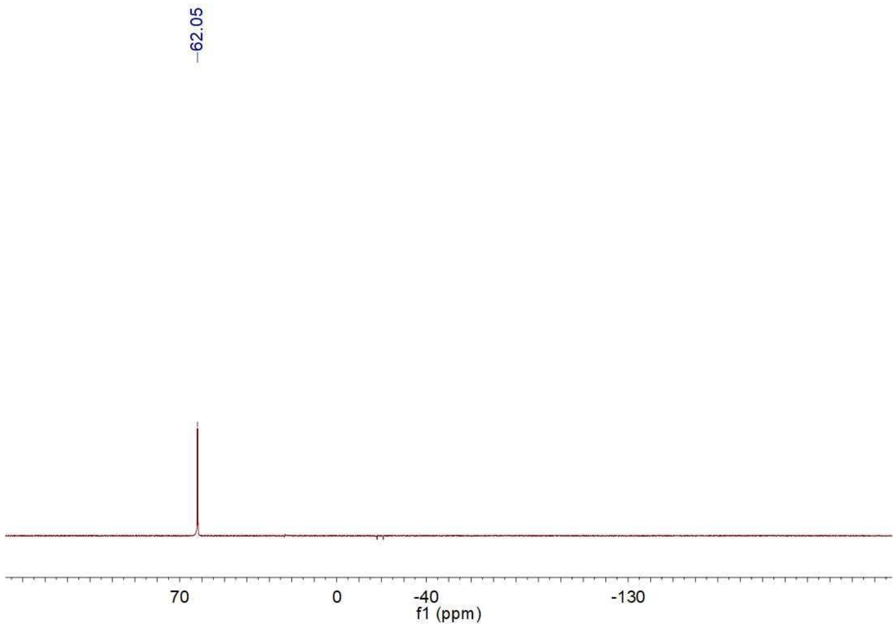

Fig. S12. ^{31}P NMR spectrum of $(L4)_2Mn_2(CO)_6$ is presented.

3.8 Catalytic activity test of $(L4)_2Mn_2(CO)_6$

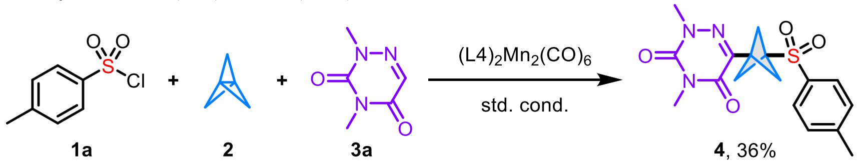

A 25 mL reaction tube equipped with a magnetic stirring bar was charged with sulfonyl chlorides (**1**) (1.5 equiv), [1.1.1]propellane (**2**) (1 equiv), heteroarenes (**3**) (0.2 mmol), $(L4)_2Mn_2(CO)_6$ (5 mmol%), BTMG (2 equiv) and EA (2 mL). The reaction mixture was allowed to stir with the irradiation of blue LEDs at room temperature under a nitrogen atmosphere for 12 h. Upon completion, it was quenched by a saturated NH_4Cl solution and extracted with EtOAc, and the combined organic layer was dried over $MgSO_4$. The solvent was removed *in vacuo*, and the obtained residue was further purified by silica gel column chromatography (200-300 mesh silica gel) to give product (**4**) in 36% yield.

4. Characterization of products

2,4-Dimethyl-6-(3-tosylbicyclo[1.1.1]pentan-1-yl)-1,2,4-triazine-3,5(2H,4H)-dione (4)

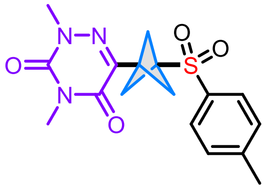

Obtained as a yellow solid (58 mg, 80% yield); M. P. = 132-133 °C; ^{1}H NMR (500 MHz, $CDCl_3$) δ 7.75 (d, J = 8.2 Hz, 2H), 7.36 (d, J = 8.0 Hz, 2H), 3.59 (s, 3H), 3.28 (s, 3H), 2.46 (s, 3H), 2.37 (s, 6H); ^{13}C NMR (126 MHz, $CDCl_3$) δ 155.6, 149.1, 144.9, 139.3, 133.6, 129.9, 128.7, 52.5, 52.0, 39.6, 37.8, 26.9, 21.7; HRMS (ESI+): Calculated for $C_{17}H_{19}N_3O_4SNa$: $[M+Na]^+$ 384.0988, Found 384.0989.

2,4-Bis(2-oxo-2-phenylethyl)-6-(3-tosylbicyclo[1.1.1]pentan-1-yl)-1,2,4-triazine-3,5(2H,4H)-dione (5)

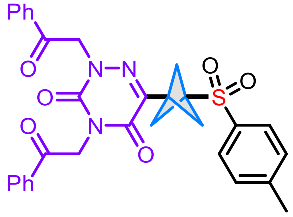

Obtained as a yellow solid (51 mg, 75% yield); M. P. = 126-127 °C; ^{1}H NMR (500 MHz, $CDCl_3$) δ 7.99 – 7.93 (m, 4H), 7.73 (d, *J* = 8.2 Hz, 2H), 7.63 (dd, *J* = 17.8, 7.5 Hz, 2H), 7.50 (dd, *J* = 18.1, 7.9 Hz, 4H), 7.34 (d, *J* = 8.0 Hz, 2H), 5.39 (s, 2H), 5.32 (s, 2H), 2.44 (s, 3H), 2.39 (s, 6H); ^{13}C NMR (126 MHz, $CDCl_3$) δ 191.3, 190.2, 154.9, 148.9, 144.9, 140.5, 134.4, 134.3, 134.2, 133.5, 129.9, 129.0, 128.9, 128.6, 128.2, 128.1, 57.6, 52.5, 52.2, 46.3, 37.8, 21.7; HRMS (ESI+): Calculated for $C_{31}H_{27}N_3O_6SNa$: $[M+Na]^+$ 592.1513, Found 592.1505.

2,4-Diallyl-6-(3-tosylbicyclo[1.1.1]pentan-1-yl)-1,2,4-triazine-3,5(2H,4H)-dione (6)

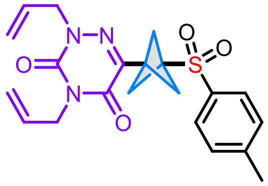

Obtained as a white solid (48 mg, 58% yield); M. P. = 126-127 °C; ^{1}H NMR (500 MHz, $CDCl_3$) δ 7.72 (d, *J* = 8.2 Hz, 2H), 7.34 (d, *J* = 8.1 Hz, 2H), 5.83 (ddd, *J* = 40.1, 17.0, 10.3 Hz, 2H), 5.28 – 5.18 (m, 4H), 4.49 (d, *J* = 6.1 Hz, 2H), 4.44 (d, *J* = 6.1 Hz, 2H), 2.44 (s, 3H), 2.36 (s, 6H); ^{13}C NMR (126 MHz, $CDCl_3$) δ 155.0, 148.3, 144.9, 139.9, 133.6, 131.1, 130.1, 129.9, 128.7, 119.6, 119.4, 54.3, 52.4, 52.1, 42.9, 37.9, 21.7; HRMS (ESI+): Calculated for $C_{21}H_{23}N_3O_4SNa$: $[M+Na]^+$ 436.1301, Found 436.1288.

1-Methyl-3-(3-tosylbicyclo[1.1.1]pentan-1-yl)quinoxalin-2(1H)-one (7)

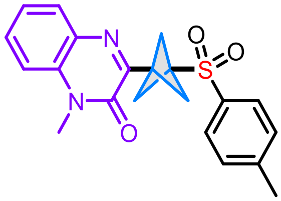

Obtained as a brown solid (43 mg, 57% yield); M. P. = 191-192 °C; ^{1}H NMR (500 MHz, $CDCl_3$) δ 7.81 (dd, *J* = 8.0, 1.3 Hz, 1H), 7.79 (d, *J* = 8.2 Hz, 2H), 7.58 – 7.53 (m, 1H), 7.38 – 7.32 (m, 3H), 7.31 – 7.27 (m, 1H), 3.63 (s, 3H), 2.53 (s, 6H), 2.46 (s, 3H); ^{13}C NMR (126 MHz, $CDCl_3$) δ 154.3, 154.2, 144.7, 133.9, 133.4, 132.7, 130.7, 130.2, 129.9, 128.7, 123.8, 113.7, 52.4, 52.3, 41.0, 28.6, 21.7; HRMS (ESI+): Calculated for $C_{21}H_{20}N_2O_3SNa$: $[M+Na]^+$ 403.1087, Found 403.1075.

1,6,7-Trimethyl-3-(3-tosylbicyclo[1.1.1]pentan-1-yl)quinoxalin-2(1H)-one (8)

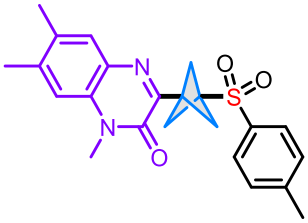

Obtained as a yellow solid (56 mg, 69% yield); M. P. = 163-164 °C; ^{1}H NMR (500 MHz, $CDCl_3$) δ 7.78 (d, *J* = 8.2 Hz, 2H), 7.57 (s, 1H), 7.36 (d, *J* = 8.0 Hz, 2H), 7.04 (s, 1H), 3.60 (s, 3H), 2.52 (s, 6H), 2.46 (s, 3H), 2.40 (s, 3H), 2.33 (s, 3H); ^{13}C NMR (126 MHz, $CDCl_3$) δ 154.4, 152.9, 144.7, 140.7, 133.9, 132.8, 131.4, 131.1, 130.2, 129.8, 128.7, 114.2, 52.4, 52.3, 41.0, 28.5, 21.7, 20.6, 19.1; HRMS (ESI+): Calculated for $C_{23}H_{25}N_2O_3S$: $[M+H]^+$ 409.1580, Found 409.1590.

1-Methyl-5,6-diphenyl-3-(3-tosylbicyclo[1.1.1]pentan-1-yl)pyrazin-2(1H)-one (9)

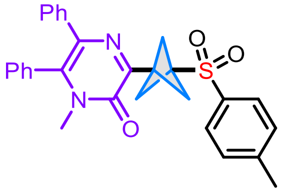

Obtained as a yellow solid (57 mg, 59% yield); M. P. = 206-207 °C; ^{1}H NMR (500 MHz, $CDCl_3$) δ 7.78 (d, *J* = 8.3 Hz, 2H), 7.39 – 7.35 (m, 5H), 7.16 (dd, *J* = 7.8, 1.7 Hz, 2H), 7.12 – 7.08 (m, 5H), 3.26 (s, 3H), 2.53 (s, 6H), 2.46 (s, 3H); ^{13}C NMR (126 MHz, $CDCl_3$) δ 155.1, 150.9, 144.6, 138.2, 137.4, 133.9, 132.1, 132.3, 129.9, 129.8, 129.6, 129.2, 129.2, 128.7, 127.7, 127.1, 52.5, 52.1, 40.7, 33.7, 21.7; HRMS (ESI+): Calculated for $C_{29}H_{27}N_2O_3S$: $[M+H]^+$ 483.1737, Found 483.1737.

3-(3-Tosylbicyclo[1.1.1]pentan-1-yl)-2H-chromen-2-one (10)

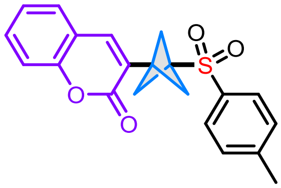

Obtained as a white solid (46 mg, 63% yield); M. P. = 138-139 °C; ^{1}H NMR (500 MHz, $CDCl_3$) δ 7.77 (d, *J* = 8.0 Hz, 2H), 7.50 (t, *J* = 7.7 Hz, 1H), 7.45 (d, *J* = 7.6 Hz, 1H), 7.43 (s, 1H), 7.38 (d, *J* = 7.9 Hz, 2H), 7.29 (dd, *J* = 12.4, 8.7 Hz, 2H), 2.47 (s, 3H), 2.40 (s, 6H); ^{13}C NMR (126 MHz, $CDCl_3$) δ 159.8, 153.6, 144.9, 139.4, 133.6, 131.7, 129.9, 128.7, 127.7, 125.1, 124.6, 118.8, 116.6, 51.9, 51.8, 38.8, 21.7; HRMS (ESI+): Calculated for $C_{21}H_{19}O_4S$: $[M+H]^+$ 367.0999, Found 367.1005.

3-(3-Tosylbicyclo[1.1.1]pentan-1-yl)cinnolin-4(1H)-one (11)

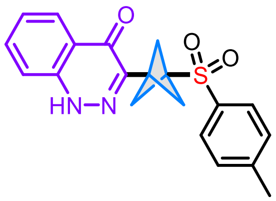

Obtained as a brown solid (50 mg, 50% yield); M. P. = 211-212 °C; ^{1}H NMR (500 MHz, $CDCl_3$) δ 11.15 (s, 1H), 8.20 (d, *J* = 8.2 Hz, 1H), 7.78 (d, *J* = 8.2 Hz, 2H), 7.70 – 7.64 (m, 1H), 7.46 (d, *J*

= 8.5 Hz, 1H), 7.36 (t, J = 7.4 Hz, 3H), 2.55 (s, 6H), 2.47 (s, 3H); ^{13}C NMR (126 MHz, $CDCl_3$) δ 171.1, 145.5, 145.0, 141.2, 133.8, 133.5, 129.9, 128.6, 125.1, 124.9, 123.2, 115.8, 52.8, 52.2, 38.9, 21.7; HRMS (ESI+): Calculated for $C_{20}H_{19}N_2O_3S$: $[M+H]^+$ 367.1111, Found 367.1121.

3-(3-Tosylbicyclo[1.1.1]pentan-1-yl)-2H-benzo[b][1,4]oxazin-2-one (12)

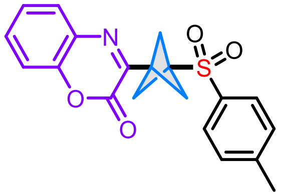

Obtained as a brown solid (51 mg, 70% yield); M. P. = 197-198 °C; ^{1}H NMR (500 MHz, $CDCl_3$) δ 7.71 (d, J = 8.2 Hz, 2H), 7.65 (dd, J = 8.0, 1.4 Hz, 1H), 7.45 – 7.39 (m, 1H), 7.31 (d, J = 8.2 Hz, 2H), 7.30 – 7.25 (m, 1H), 7.20 – 7.18 (m, 1H), 2.44 (s, 6H), 2.40 (s, 3H); ^{13}C NMR (126 MHz, $CDCl_3$) δ 150.7, 150.7, 145.6, 143.9, 132.5, 130.6, 130.0, 128.9, 128.3, 127.7, 124.7, 115.4, 51.3, 51.2, 39.2, 20.7; HRMS (ESI+): Calculated for $C_{20}H_{18}NO_4S$: $[M+H]^+$ 368.0951, Found 368.0950.

2-(3-Tosylbicyclo[1.1.1]pentan-1-yl)quinoline (13)

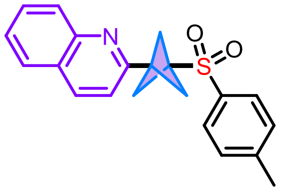

Obtained as a white liquid (30 mg, 43% yield); ^{1}H NMR (500 MHz, $CDCl_3$) δ 8.03 (d, J = 8.4 Hz, 1H), 7.95 (d, J = 8.1 Hz, 1H), 7.74 (d, J = 8.2 Hz, 2H), 7.70 (d, J = 8.1 Hz, 1H), 7.62 (t, J = 7.7 Hz, 1H), 7.43 (t, J = 7.5 Hz, 1H), 7.31 (d, J = 8.0 Hz, 2H), 7.20 (d, J = 9.8 Hz, 1H), 2.42 (s, 6H), 2.39 (s, 3H); ^{13}C NMR (126 MHz, $CDCl_3$) δ 156.8, 144.8, 136.7, 133.9, 129.9, 129.8, 129.1, 128.7, 128.7, 127.6, 127.2, 126.6, 118.5, 52.3, 51.5, 42.1, 21.7; HRMS (ESI+): Calculated for $C_{21}H_{20}NO_2S$: $[M+H]^+$ 350.1209, Found 350.1206.

1-(3-Tosylbicyclo[1.1.1]pentan-1-yl)isoquinoline (14)

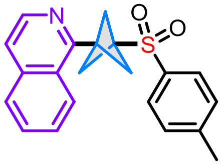

Obtained as a yellow solid (33 mg, 47% yield); M. P. = 153-154 °C; ^{1}H NMR (500 MHz, $CDCl_3$) δ 8.43 (d, J = 5.7 Hz, 1H), 8.22 (d, J = 8.5 Hz, 1H), 7.84 (t, J = 7.3 Hz, 3H), 7.69 (t, J = 7.4 Hz, 1H), 7.63 – 7.55 (m, 2H), 7.39 (d, J = 8.0 Hz, 2H), 2.70 (s, 6H), 2.47 (s, 3H); ^{13}C NMR (126 MHz, $CDCl_3$) δ 155.3, 144.8, 136.5, 133.8, 130.3, 129.9, 129.9, 128.7, 127.7, 127.5, 127.0, 125.1, 120.9, 53.7, 52.6, 42.6, 21.7; HRMS (ESI+): Calculated for $C_{21}H_{20}NO_2S$: $[M+H]^+$ 350.1209, Found 350.1213.

Methyl 1-methyl-2-(3-tosylbicyclo[1.1.1]pentan-1-yl)-1*H*-indole-3-carboxylate (15)

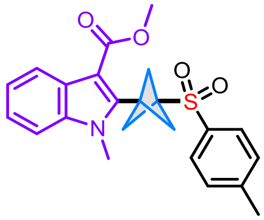

Obtained as a brown solid (45 mg, 55% yield); M. P. = 155-156 °C; ^{1}H NMR (500 MHz, $CDCl_3$) δ 7.97 (d, *J* = 7.9 Hz, 1H), 7.72 (d, *J* = 8.1 Hz, 2H), 7.31 (d, *J* = 8.0 Hz, 2H), 7.20 (d, *J* = 9.2 Hz, 2H), 7.15 (d, *J* = 8.0 Hz, 1H), 3.80 (s, 3H), 3.72 (s, 3H), 2.70 (s, 6H), 2.40 (s, 3H); ^{13}C NMR (126 MHz, $CDCl_3$) δ 165.3, 144.9, 140.3, 136.7, 133.7, 130.0, 128.7, 126.2, 123.2, 122.1, 121.9, 109.3, 106.7, 54.1, 53.2, 51.0, 36.0, 31.3, 21.7; HRMS (ESI+): Calculated for $C_{23}H_{24}NO_4S$: $[M+H]^+$ 412,1577, Found 412.1571.

6-(3-((4-Methoxyphenyl)sulfonyl)bicyclo[1.1.1]pentan-1-yl)-2,4-dimethyl-1,2,4-triazine-3,5(2H,4H)-dione (16)

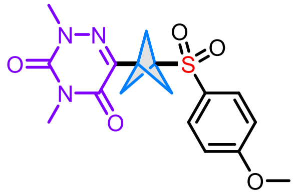

Obtained as a yellow solid (63 mg, 84% yield); M. P. = 185-186 °C; ^{1}H NMR (500 MHz, $CDCl_3$) δ 7.78 (d, *J* = 8.9 Hz, 2H), 7.01 (d, *J* = 8.9 Hz, 2H), 3.88 (s, 3H), 3.58 (s, 3H), 3.27 (s, 3H), 2.36 (s, 6H); ^{13}C NMR (126 MHz, $CDCl_3$) δ 163.9, 155.6, 149.1, 139.3, 130.8, 128.1, 114.5, 55.7, 52.6, 52.0, 39.6, 37.8, 26.9; HRMS (ESI+): Calculated for $C_{17}H_{20}N_3O_5S$: $[M+H]^+$ 378.1118, Found 387.1122.

2,4-Dimethyl-6-(3-((4-(trifluoromethoxy)phenyl)sulfonyl)bicyclo[1.1.1]pentan-1-yl)-1,2,4-triazine-3,5(2H,4H)-dione (17)

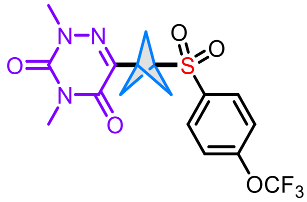

Obtained as a yellow solid (64 mg, 74% yield); M. P. = 153-154 °C; ^{1}H NMR (500 MHz, $CDCl_3$) δ 7.93 (d, *J* = 8.8 Hz, 2H), 7.40 (d, *J* = 8.1 Hz, 2H), 3.59 (s, 3H), 3.28 (s, 3H), 2.40 (s, 6H); ^{13}C NMR (126 MHz, $CDCl_3$) δ 155.6, 153.2 (q, *J* = 1.3 Hz), 149.1, 139.0, 134.9, 130.9, 120.9, 120.2 (q, *J* = 260.8 Hz), 52.4, 52.1, 39.7, 38.0, 27.0; ^{19}F NMR (471 MHz, $CDCl_3$) δ -57.65; HRMS (ESI+): Calculated for $C_{17}H_{17}F_3N_3O_5S$: $[M+H]^+$ 432.0836, Found 432.0825

6-(3-((4-Acetylphenyl)sulfonyl)bicyclo[1.1.1]pentan-1-yl)-2,4-dimethyl-1,2,4-triazine-3,5(2H,4H)-dione (18)

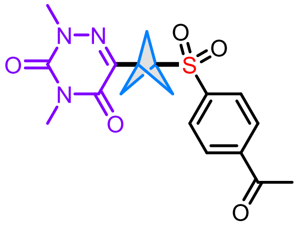

Obtained as a yellow solid (54 mg, 69% yield); M. P. = 192-193 °C; ^{1}H NMR (500 MHz, $CDCl_3$) δ 8.12 (d, J = 8.5 Hz, 2H), 7.98 (d, J = 8.5 Hz, 2H), 3.58 (s, 3H), 3.28 (s, 3H), 2.67 (s, 3H), 2.40 (s, 6H); ^{13}C NMR (126 MHz, $CDCl_3$) δ 196.7, 155.6, 149.1, 141.0, 140.5, 138.9, 129.1, 128.9, 52.4, 52.2, 39.7, 38.0, 27.0; HRMS (ESI+): Calculated for $C_{18}H_{20}N_3O_5S$: $[M+H]^+$ 390.1118, Found 390.1113.

4-((3-(2,4-Dimethyl-3,5-dioxo-2,3,4,5-tetrahydro-1,2,4-triazin-6-yl)bicyclo[1.1.1]pentan-1-yl)sulfonyl)benzonitrile (19)

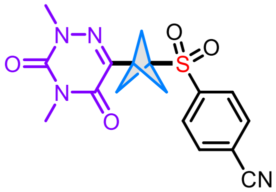

Obtained as a yellow solid (47 mg, 63% yield); M. P. = 128-129 °C; ^{1}H NMR (500 MHz, $CDCl_3$) δ 8.00 (d, J = 8.4 Hz, 2H), 7.88 (d, J = 8.4 Hz, 2H), 3.58 (s, 3H), 3.27 (s, 3H), 2.40 (s, 6H); ^{13}C NMR (126 MHz, $CDCl_3$) δ 155.6, 149.0, 140.9, 138.7, 133.0, 129.4, 117.8, 117.1, 52.2, 39.7, 38.1, 27.0; HRMS (ESI+): Calculated for $C_{17}H_{17}N_4O_4S$: $[M+H]^+$ 373.0965, Found 373.0958.

6-(3-((3-Bromophenyl)sulfonyl)bicyclo[1.1.1]pentan-1-yl)-2,4-dimethyl-1,2,4-triazine-3,5(2H,4H)-dione (20)

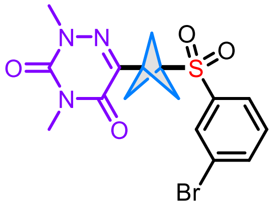

Obtained as a yellow solid (56 mg, 66% yield); M. P. = 190-191 °C; ^{1}H NMR (500 MHz, $CDCl_3$) δ 8.02 (t, J = 1.7 Hz, 1H), 7.84 – 7.79 (m, 2H), 7.47 (t, J = 7.9 Hz, 1H), 3.60 (s, 3H), 3.29 (s, 3H), 2.42 (s, 6H); ^{13}C NMR (126 MHz, $CDCl_3$) δ 155.6, 149.1, 139.0, 138.6, 137.0, 131.5, 130.8, 127.2, 123.4, 52.4, 52.2, 39.7, 38.0, 27.0; HRMS (ESI+): Calculated for $C_{16}H_{16}BrN_3O_4SNa$: $[M+Na]^+$ 447.9937, Found 447.9936.

6-(3-((4-Iodophenyl)sulfonyl)bicyclo[1.1.1]pentan-1-yl)-2,4-dimethyl-1,2,4-triazine-3,5(2H,4H)-dione (21)

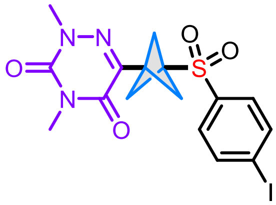

Obtained as a yellow solid (48 mg, 51% yield); M. P. = 225-226 °C; ^{1}H NMR (500 MHz, $CDCl_3$) δ 7.94 (d, J = 8.4 Hz, 2H), 7.57 (d, J = 8.5 Hz, 2H), 3.59 (s, 3H), 3.28 (s, 3H), 2.38 (s, 6H); ^{13}C NMR (126 MHz, $CDCl_3$) δ 155.6, 149.1, 139.0, 138.6, 136.3, 130.0, 102.0, 52.4, 52.1, 39.7, 38.0, 27.0; HRMS (ESI+): Calculated for $C_{16}H_{17}IN_3O_4S$: $[M+H]^+$ 473.9979, Found 473.9990.

6-(3-((3,5-Difluorophenyl)sulfonyl)bicyclo[1.1.1]pentan-1-yl)-2,4-dimethyl-1,2,4-triazine-3,5(2H,4H)-dione (22)

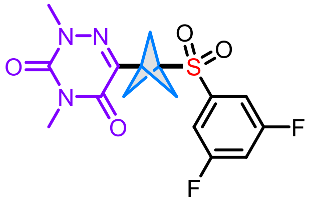

Obtained as a yellow solid (54 mg, 70% yield); M. P. = 208-209 °C; ^{1}H NMR (500 MHz, $CDCl_3$) δ 7.42 (dd, J = 4.2, 1.6 Hz, 2H), 7.13 (tt, J = 8.4, 2.2 Hz, 1H), 3.59 (s, 3H), 3.29 (s, 3H), 2.42 (s, 6H); ^{13}C NMR (126 MHz, $CDCl_3$) δ 162.9 (dd, J = 256.2, 11.4 Hz), 155.6, 149.1, 140.1 (t, J = 7.8 Hz), 138.8, 112.2 (dd, J = 20.9, 7.3 Hz), 109.7 (t, J = 25.0 Hz), 52.3, 52.2, 39.7, 38.0, 27.0; ^{19}F NMR (471 MHz, $CDCl_3$) δ -104.79; HRMS (ESI+): Calculated for $C_{16}H_{15}F_2N_3O_4SNa$: $[M+Na]^+$ 406.0644, Found 406.0640

2,4-Dimethyl-6-(3-(thiophen-2-ylsulfonyl)bicyclo[1.1.1]pentan-1-yl)-1,2,4-triazine-3,5(2H,4H)-dione (23)

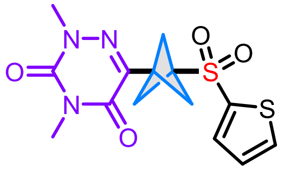

Obtained as a yellow solid (42 mg, 59% yield); M. P. = 203-204 °C; ^{1}H NMR (500 MHz, $CDCl_3$) δ 7.75 (d, J = 4.1 Hz, 1H), 7.67 (d, J = 4.4 Hz, 1H), 7.19 (dd, J = 5.0, 3.8 Hz, 1H), 3.59 (s, 3H), 3.28 (s, 3H), 2.43 (s, 6H); ^{13}C NMR (126 MHz, $CDCl_3$) δ 155.6, 149.1, 139.1, 137.5, 134.7, 134.6, 128.1, 53.2, 52.2, 39.7, 37.3, 27.0; HRMS (ESI+): Calculated for $C_{14}H_{15}N_3O_4S_2Na$: $[M+Na]^+$ 376.0396, Found 376.0396.

6-(3-((3,5-Dimethylisoxazol-4-yl)sulfonyl)bicyclo[1.1.1]pentan-1-yl)-2,4-dimethyl-1,2,4-triazine-3,5(2H,4H)-dione (24)

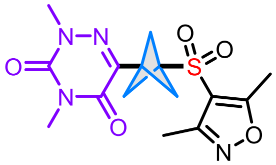

Obtained as a yellow solid (49 mg, 67% yield); M. P. = 204-205 °C; ^{1}H NMR (500 MHz, $CDCl_3$) δ 3.60 (s, 3H), 3.30 (s, 3H), 2.64 (s, 3H), 2.45 (s, 6H), 2.41 (s, 3H); ^{13}C NMR (126 MHz, $CDCl_3$) δ 175.5, 158.5, 155.6, 149.1, 138.8, 113.1, 53.3, 51.8, 39.7, 37.7, 27.0, 12.6, 11.0; HRMS (ESI+): Calculated for $C_{15}H_{19}N_4O_5S$: $[M+H]^+$ 367.1071, Found 367.1080.

2,4-Dimethyl-6-(3-(propylsulfonyl)bicyclo[1.1.1]pentan-1-yl)-1,2,4-triazine-3,5(2H,4H)-dione (25)

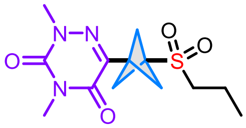

Obtained as a yellow solid (33 mg, 53% yield); M. P. = 172-173 °C; ^{1}H NMR (500 MHz, $CDCl_3$) δ 3.63 (s, 3H), 3.33 (s, 3H), 2.98 – 2.92 (m, 2H), 2.57 (s, 6H), 1.91 (dd, J = 15.6, 7.7 Hz, 2H), 1.10 (t, J = 7.4 Hz, 3H); ^{13}C NMR (126 MHz, $CDCl_3$) δ 155.7, 149.1, 139.1, 52.5, 52.0, 51.3, 39.7, 37.7, 27.0, 15.3, 13.4; HRMS (ESI+): Calculated for $C_{13}H_{20}N_3O_4S$: $[M+H]^+$ 314.1169, Found 314.1168.

6-(3-(Cyclopropylsulfonyl)bicyclo[1.1.1]pentan-1-yl)-2,4-dimethyl-1,2,4-triazine-3,5(2H,4H)-dione (26)

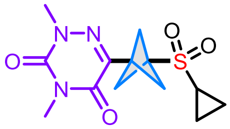

Obtained as a yellow solid (36 mg, 58% yield); M. P. = 161-162 °C; ^{1}H NMR (500 MHz, $CDCl_3$) δ 3.63 (s, 3H), 3.33 (s, 3H), 2.58 (s, 6H), 2.36 – 2.32 (m, 1H), 1.23 (dd, J = 4.8, 2.3 Hz, 2H), 1.05 (dd, J = 7.9, 2.4 Hz, 2H); ^{13}C NMR (126 MHz, $CDCl_3$) δ 155.7, 149.1, 139.2, 52.5, 51.3, 39.79, 37.9, 27.0, 26.5, 4.1; HRMS (ESI+): Calculated for $C_{13}H_{18}N_3O_4S$: $[M+H]^+$ 312.1013, Found 312.1016.

2,4-Dimethyl-6-(3-(piperidin-1-ylsulfonyl)bicyclo[1.1.1]pentan-1-yl)-1,2,4-triazine-3,5(2H,4H)-dione (27)

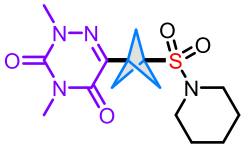

Obtained as a yellow solid (34 mg, 48% yield); M. P. = 182-183 °C; ^{1}H NMR (500 MHz, $CDCl_3$) δ 3.61 (s, 3H), 3.33 (d, J = 5.1 Hz, 4H), 3.31 (s, 3H), 2.53 (s, 6H), 1.63 (t, J = 5.2 Hz, 4H), 1.60 – 1.56 (m, 2H); ^{13}C NMR (126 MHz, $CDCl_3$) δ 155.7, 149.1, 139.3, 53.3, 50.6, 47.0, 39.7, 38.4, 27.0, 26.0, 23.9; HRMS (ESI+): Calculated for $C_{15}H_{23}N_4O_4S$: $[M+H]^+$ 355.1435, Found 355.1438.

1-Tosylbicyclo[1.1.1]pentane (28)

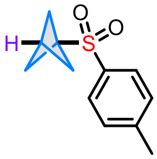

Obtained as white solid (29 mg, 65% yield); M. P. = 92-93 °C; ^{1}H NMR (500 MHz, $CDCl_3$) δ 7.70 (d, J = 8.0 Hz, 2H), 7.32 (d, J = 7.9 Hz, 2H), 2.69 (s, 1H), 2.42 (s, 3H), 2.04 (s, 6H); ^{13}C NMR (126 MHz, $CDCl_3$) δ 144.6, 133.8, 129.8, 128.6, 55.1, 50.3, 26.7, 21.7; HRMS (ESI+): Calculated for $C_{12}H_{14}O_2SNa$: $[M+Na]^+$ 245.0613, Found 245.0621.

1-(Phenylsulfonyl)bicyclo[1.1.1]pentane (29)

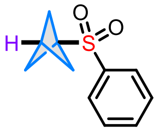

Obtained as white solid (25 mg, 59% yield); M. P. = 88-89 °C; ^{1}H NMR (500 MHz, $CDCl_3$) δ 7.86 (dd, J = 8.3, 1.2 Hz, 2H), 7.66 (t, J = 7.5 Hz, 1H), 7.56 (t, J = 7.6 Hz, 2H), 2.73 (s, 1H), 2.08 (s, 6H); ^{13}C NMR (126 MHz, $CDCl_3$) δ 136.9, 133.6, 129.1, 128.6, 55.1, 50.4, 26.8; HRMS (ESI+): Calculated for $C_{11}H_{12}O_2SNa$: $[M+Na]^+$ 231.0456, Found 231.0466.

1-((4-Methoxyphenyl)sulfonyl)bicyclo[1.1.1]pentane (30)

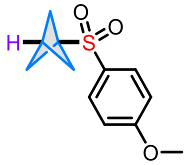

Obtained as white solid (29 mg, 62% yield); M. P. = 89-90 °C; ^{1}H NMR (500 MHz, $CDCl_3$) δ 7.77 (d, J = 8.8 Hz, 2H), 7.01 (d, J = 8.8 Hz, 2H), 3.88 (s, 3H), 2.71 (s, 1H), 2.06 (s, 6H); ^{13}C NMR (126 MHz, $CDCl_3$) δ 163.7, 130.7, 128.4, 114.3, 55.7, 55.3, 50.3, 26.6; HRMS (ESI+): Calculated for $C_{12}H_{14}O_3SH$: $[M+H]^+$ 239.0743, Found 239.0750.

1-((4-(Trifluoromethyl)phenyl)sulfonyl)bicyclo[1.1.1]pentane (31)

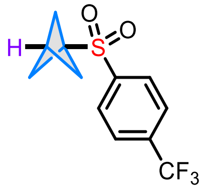

Obtained as white solid (31 mg, 56% yield); M. P. = 103-104 °C; ^{1}H NMR (500 MHz, $CDCl_3$) δ 7.91 (d, J = 8.7 Hz, 2H), 7.38 (d, J = 8.3 Hz, 2H), 2.75 (s, 1H), 2.09 (s, 6H); ^{13}C NMR (126 MHz, $CDCl_3$) δ 153.0, 135.2, 130.9, 120.8, 120.2 (q, J = 252.0 Hz), 55.0 (q, J = 17.6 Hz), 50.5, 26.9; ^{19}F NMR (471 MHz, $CDCl_3$) δ -57.67; HRMS (ESI+): Calculated for $C_{12}H_{11}F_3O_2SNa$: $[M+Na]^+$ 299.0330, Found 299.0331.

1-((3-Bromophenyl)sulfonyl)bicyclo[1.1.1]pentane (32)

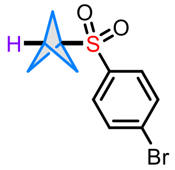

Obtained as a yellow solid (30 mg, 53% yield); M. P. = 115-1116 °C; ^{1}H NMR (500 MHz, $CDCl_3$) δ 7.67 (s, 4H), 2.70 (s, 1H), 2.04 (s, 6H); ^{13}C NMR (126 MHz, $CDCl_3$) δ 135.9, 132.5, 130.2, 129.0, 55.0, 50.4, 26.9; HRMS (ESI+): Calculated for $C_{11}H_{12}BrO_2S$: $[M+H]^+$ 286.9742, Found 286.9750.

3-(Bicyclo[1.1.1]pentan-1-ylsulfonyl)pyridine (33)

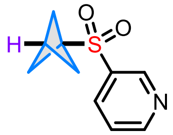

Obtained as a yellow solid (20 mg, 47% yield); M. P. = 127-128 °C; ^{1}H NMR (500 MHz, $CDCl_3$) δ 9.05 (s, 1H), 8.88 (d, J = 3.1 Hz, 1H), 8.14 (d, J = 8.0 Hz, 1H), 7.52 (dd, J = 7.7, 4.8 Hz, 1H), 2.77 (s, 1H), 2.11 (s, 6H); ^{13}C NMR (126 MHz, $CDCl_3$) δ 154.2, 149.6, 136.4, 133.5, 123.8, 55.2, 50.5, 27.1; HRMS (ESI+): Calculated for $C_{10}H_{12}NO_2S$: $[M+H]^+$ 210.0589, Found 210.0566.

3-(3-Tosylbicyclo[1.1.1]pentan-1-yl)phthalonitrile (34)

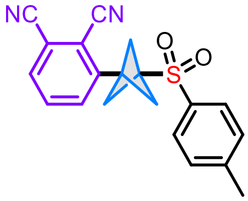

Obtained as a yellow solid (34 mg, 49% yield); M. P. = 190-191 °C; ^{1}H NMR (400 MHz, $CDCl_3$) δ 7.76 (t, J = 8.0 Hz, 3H), 7.55 (d, J = 1.3 Hz, 1H), 7.49 (dd, J = 8.1, 1.6 Hz, 1H), 7.39 (d, J = 8.0 Hz, 2H), 2.47 (s, 3H), 2.37 (s, 6H); ^{13}C NMR (126 MHz, $CDCl_3$) δ 145.3, 144.0, 133.8, 133.3, 131.4, 130.9, 130.1, 128.7, 116.3, 115.1, 115.0, 114.7, 52.4, 51.0, 40.3, 21.7; HRMS (ESI+): Calculated for $C_{20}H_{16}N_2O_2SNa$: $[M+Na]^+$ 371.0825, Found 371.0824.

3-(3-((4-Bromophenyl)sulfonyl)bicyclo[1.1.1]pentan-1-yl)phthalonitrile (35)

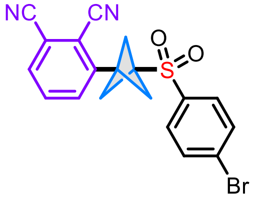

Obtained as a yellow solid (35 mg, 42% yield); M. P. = 179-180 °C; ^{1}H NMR (500 MHz, $CDCl_3$) δ 7.79 – 7.74 (m, 5H), 7.56 (d, J = 1.1 Hz, 1H), 7.49 (dd, J = 8.1, 1.4 Hz, 1H), 2.39 (s, 6H); ^{13}C NMR (126 MHz, $CDCl_3$) δ 143.6, 135.3, 133.8, 132.8, 131.3, 130.9, 130.2, 129.7, 116.4, 115.0, 114.9, 114.9, 52.5, 51.0, 40.4; HRMS (ESI+): Calculated for $C_{19}H_{13}BrN_2O_2SNa$: $[M+Na]^+$ 434.9773, Found 434.9755.

3-(3-(3-Tolylsulfonyl)bicyclo[1.1.1]pentan-1-yl)phthalonitrile (36)

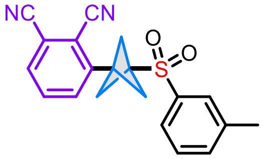

Obtained as a yellow solid (32 mg, 46% yield); M. P. = 182-183 °C; ^{1}H NMR (500 MHz, $CDCl_3$) δ 7.75 (d, J = 8.1 Hz, 1H), 7.70 (d, J = 8.1 Hz, 2H), 7.56 (s, 1H), 7.53 – 7.46 (m, 3H), 2.47 (s, 3H), 2.39 (s, 6H); ^{13}C NMR (126 MHz, $CDCl_3$) δ 143.9, 139.8, 136.1, 135.0, 133.8, 131.4, 130.9, 129.3, 128.9, 125.8, 116.4, 115.1, 114.9, 114.8, 52.5, 51.0, 40.3, 21.4; HRMS (ESI+): Calculated for $C_{20}H_{16}N_2O_2SNa$: $[M+Na]^+$ 371.0825, Found 371.0826.

2-(3-Tosylbicyclo[1.1.1]pentan-1-yl)terephthalonitrile (37)

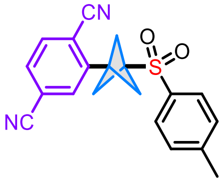

Obtained as a yellow solid (22 mg, 32% yield); M. P. = 187-188 °C; ^{1}H NMR (500 MHz, $CDCl_3$) δ 7.78 (d, J = 8.3 Hz, 2H), 7.72 (d, J = 8.0 Hz, 1H), 7.66 (d, J = 8.0 Hz, 1H), 7.50 (s, 1H), 7.39 (d, J = 7.9 Hz, 2H), 2.56 (s, 6H), 2.48 (s, 3H); ^{13}C NMR (126 MHz, $CDCl_3$) δ 145.3, 142.3, 134.3,

133.3, 131.7, 131.4, 130.1, 128.7, 116.9, 116.7, 116.3, 114.9, 52.4, 51.3, 39.9, 21.7; HRMS (ESI+): Calculated for $C_{20}H_{16}N_2O_2SNa$: $[M+Na]^+$ 371.0825, Found 371.0832.

1-(2-Nitrophenyl)-3-tosylbicyclo[1.1.1]pentane (38)

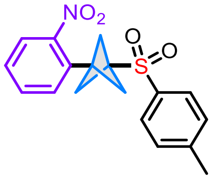

Obtained as a brown liquid (27 mg, 39% yield); ^{1}H NMR (500 MHz, $CDCl_3$) δ 7.74 (dd, J = 16.0, 7.7 Hz, 3H), 7.52 (s, 1H), 7.42 (d, J = 7.2 Hz, 1H), 7.37 (d, J = 7.1 Hz, 2H), 7.27 (s, 1H), 2.47 (s, 3H), 2.43 (s, 6H); ^{13}C NMR (126 MHz, $CDCl_3$) δ 149.3, 145.0, 133.6, 132.6, 131.3, 130.2, 130.0, 128.8, 128.7, 124.2, 52.5, 51.5, 39.6, 21.7; HRMS (ESI+): Calculated for $C_{18}H_{18}NO_4S$: $[M+H]^+$ 344.0951, Found 344.0951.

1-(3,3-Difluoro-2-phenylallyl)-3-tosylbicyclo[1.1.1]pentane (39)

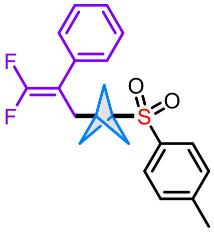

Obtained as a colourless liquid (49 mg, 66% yield); ^{1}H NMR (500 MHz, $CDCl_3$) δ 7.66 (d, J = 8.2 Hz, 2H), 7.34 – 7.29 (m, 4H), 7.27 – 7.25 (m, 1H), 7.23 (d, J = 8.2 Hz, 2H), 2.65 – 2.60 (m, 2H), 2.43 (s, 3H), 1.80 (s, 6H); ^{13}C NMR (126 MHz, $CDCl_3$) δ 154.34 (dd, J = 293.0, 287.7 Hz), 144.5, 133.9, 133.2 (t, J = 3.9 Hz), 129.7, 128.6, 128.6, 127.9 (t, J = 3.4 Hz), 127.6, 89.4 (dd, J = 21.8, 14.0 Hz), 51.5, 50.9, 38.1 (dd, J = 3.9, 2.3 Hz), 29.6, 21.6; ^{19}F NMR (471 MHz, $CDCl_3$) δ -89.71, -89.79, -90.47, -90.56; HRMS (ESI+): Calculated for $C_{21}H_{20}F_2O_2SNa$: $[M+Na]^+$ 397.1044, Found 397.1046.

1-(3,3-Difluoro-2-phenylallyl)-3-(phenylsulfonyl)bicyclo[1.1.1]pentane (40)

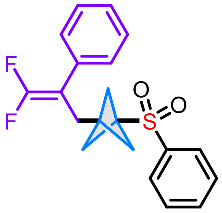

Obtained as a colourless liquid (44 mg, 61% yield); ^{1}H NMR (500 MHz, $CDCl_3$) δ 7.82 – 7.76 (m, 2H), 7.63 (dd, J = 10.6, 4.3 Hz, 1H), 7.53 (t, J = 7.7 Hz, 2H), 7.33 (t, J = 7.4 Hz, 2H), 7.27 (d, J = 1.1 Hz, 1H), 7.25 – 7.22 (m, 2H), 2.66 – 2.61 (m, 2H), 1.81 (s, 6H); ^{13}C NMR (126 MHz, $CDCl_3$) δ 153.9 (dd, J = 293.0, 287.7 Hz), 136.9, 133.6, 133.1, 129.1, 128.6, 128.6, 127.9 (t, J = 3.4 Hz), 127.6, 89.4 (dd, J = 21.8, 14.0 Hz), 51.5, 51.0, 38.2 (dd, J = 3.7, 2.1 Hz), 29.6; ^{19}F NMR (471 MHz, $CDCl_3$) δ -89.71, -89.79, -90.44, -90.53; HRMS (ESI+): Calculated for $C_{20}H_{18}F_2O_2SNa$: $[M+Na]^+$ 383.0888, Found 383.0886.

1-((2-Chlorophenyl)sulfonyl)-3-(3,3-difluoro-2-phenylallyl)bicyclo[1.1.1]pentane (41)

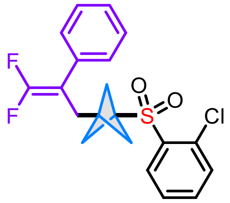

Obtained as a colourless liquid (36 mg, 46% yield); ^{1}H NMR (500 MHz, $CDCl_3$) δ 8.01 (dd, J = 7.9, 1.3 Hz, 1H), 7.55 – 7.51 (m, 2H), 7.43 – 7.40 (m, 1H), 7.33 (t, J = 7.5 Hz, 2H), 7.27 – 7.24 (m, 3H), 2.68 – 2.64 (m, 2H), 1.94 (s, 6H); ^{13}C NMR (126 MHz, $CDCl_3$) δ 154.4 (dd, J = 293.0, 287.6 Hz), 135.3, 134.7, 133.6, 133.1 (t, J = 3.4 Hz), 132.4, 132.0, 128.6, 127.9 (t, J = 3.4 Hz), 127.6, 127.3, 89.4 (dd, J = 21.7, 14.0 Hz), 52.0, 51.4, 37.7 (dd, J = 3.9, 2.3 Hz), 29.5; ^{19}F NMR (471 MHz, $CDCl_3$) δ -89.65, -89.74, -90.43, -90.51; HRMS (ESI+): Calculated for $C_{20}H_{17}ClF_2O_2SNa$: $[M+Na]^+$ 417.0498, Found 417.0498.

1-((3-Bromophenyl)sulfonyl)-3-(3,3-difluoro-2-phenylallyl)bicyclo[1.1.1]pentane (42)

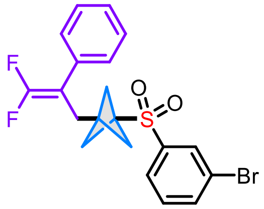

Obtained as a colourless liquid (46 mg, 52% yield); ^{1}H NMR (500 MHz, $CDCl_3$) δ 7.93 (t, J = 1.7 Hz, 1H), 7.77 – 7.74 (m, 1H), 7.73 – 7.70 (m, 1H), 7.41 (t, J = 7.9 Hz, 1H), 7.34 (t, J = 7.4 Hz, 2H), 7.28 (d, J = 7.3 Hz, 1H), 7.24 (d, J = 8.2 Hz, 2H), 2.67 – 2.63 (m, 2H), 1.83 (s, 6H); ^{13}C NMR (126 MHz, $CDCl_3$) δ 154.3 (dd, J = 293.0, 287.6 Hz), 138.8, 136.7, 133.0, 131.4, 130.6, 128.7, 127.9 (t, J = 3.3 Hz), 127.6, 127.2, 123.2, 89.3 (dd, J = 21.7, 13.9 Hz), 51.5, 51.1, 38.4 (dd, J = 3.8, 2.5 Hz), 29.5; ^{19}F NMR (471 MHz, $CDCl_3$) δ -89.59, -89.68, -90.30, -90.38; HRMS (ESI+): Calculated for $C_{20}H_{17}BrF_2O_2SNa$: $[M+Na]^+$ 460.9993, Found 460.9998.

2-((3-(3,3-Difluoro-2-phenylallyl)bicyclo[1.1.1]pentan-1-yl)sulfonyl)thiophene (43)

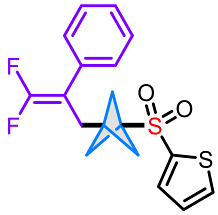

Obtained as a colourless liquid (37 mg, 50% yield); ^{1}H NMR (500 MHz, $CDCl_3$) δ 7.70 (dd, J = 5.0, 1.3 Hz, 1H), 7.58 (dd, J = 3.7, 1.3 Hz, 1H), 7.34 (t, J = 7.4 Hz, 2H), 7.28 (d, J = 1.3 Hz, 1H), 7.26 (dd, J = 5.2, 1.8 Hz, 1H), 7.24 (d, J = 1.2 Hz, 1H), 7.14 (dd, J = 4.9, 3.8 Hz, 1H), 2.68 – 2.65 (m, 2H), 1.86 (s, 6H); ^{13}C NMR (151 MHz, $CDCl_3$) δ 154.3 (dd, J = 292.6, 287.7 Hz), 137.7, 134.4, 134.2, 133.1 (t, J = 3.8 Hz), 128.6, 127.9, 127.9 (t, J = 3.2 Hz), 127.6, 89.4 (dd, J = 21.8, 14.1 Hz), 52.2, 51.1, 37.6 (dd, J = 3.8, 2.5 Hz), 29.5; ^{19}F NMR (376 MHz, $CDCl_3$) δ -89.53, -89.64, -90.31, -90.42; HRMS (ESI+): Calculated for $C_{18}H_{16}F_2O_2S_2Na$: $[M+Na]^+$ 389.0452, Found 389.0449.

(4-Chlorophenyl)(3-tosylbicyclo[1.1.1]pentan-1-yl)methanone (44)

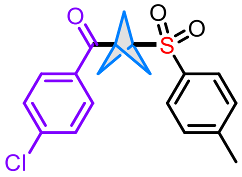

Obtained as a yellow solid (35 mg, 49% yield); M. P. = 155-156 °C; ^{1}H NMR (500 MHz, $CDCl_3$) δ 7.83 (d, *J* = 8.5 Hz, 2H), 7.77 (d, *J* = 8.2 Hz, 2H), 7.42 (d, *J* = 8.5 Hz, 2H), 7.39 (d, *J* = 8.2 Hz, 2H), 2.50 (s, 6H), 2.47 (s, 3H); ^{13}C NMR (126 MHz, $CDCl_3$) δ 193.7, 145.2, 140.2, 133.9, 133.3, 130.1, 130.0, 129.1, 128.7, 53.3, 52.1, 42.3, 29.7; HRMS (ESI+): Calculated for $C_{19}H_{17}ClO_3SNa$: $[M+Na]^+$ 383.0479, Found 383.0486.

(4-Chlorophenyl)(3-(piperidin-1-ylsulfonyl)bicyclo[1.1.1]pentan-1-yl)methanone (45)

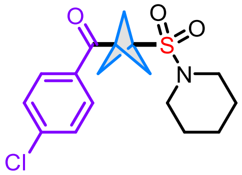

Obtained as a yellow solid (32 mg, 46% yield); M. P. = 166-167 °C; ^{1}H NMR (400 MHz, $CDCl_3$) δ 7.88 (d, *J* = 8.4 Hz, 2H), 7.45 (d, *J* = 8.4 Hz, 2H), 3.37 – 3.33 (m, 4H), 2.66 (s, 6H), 1.66 – 1.62 (m, 6H); ^{13}C NMR (101 MHz, $CDCl_3$) δ 193.8, 140.3, 133.9, 130.2, 129.2, 54.6, 50.2, 47.1, 43.2, 26.1, 23.9; HRMS (ESI+): Calculated for $C_{17}H_{21}ClNO_3S$: $[M+H]^+$ 354.0925, Found 354.0923.

(4-Fluorophenyl)(3-tosylbicyclo[1.1.1]pentan-1-yl)methanone (46)

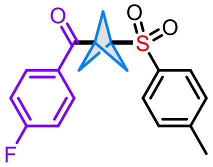

Obtained as a yellow solid (27 mg, 40% yield); M. P. = 126-127 °C; ^{1}H NMR (500 MHz, $CDCl_3$) δ 7.93 (dd, *J* = 8.1, 5.6 Hz, 2H), 7.77 (d, *J* = 7.9 Hz, 2H), 7.39 (d, *J* = 8.0 Hz, 2H), 7.12 (t, *J* = 8.4 Hz, 2H), 2.51 (s, 6H), 2.48 (s, 3H); ^{13}C NMR (151 MHz, $CDCl_3$) δ 193.2, 165.9 (d, *J* = 256.7 Hz), 145.2, 133.3, 132.0, 131.5 (d, *J* = 10.6 Hz), 130.0, 128.7, 116.0 (d, *J* = 22.7 Hz), 53.3, 52.1, 42.3, 21.7; ^{19}F NMR (376 MHz, $CDCl_3$) δ -103.30; HRMS (ESI+): Calculated for $C_{19}H_{18}FO_3S$: $[M+H]^+$ 345.0955, Found 345.0951.

(3-Nitrophenyl)(3-tosylbicyclo[1.1.1]pentan-1-yl)methanone (47)

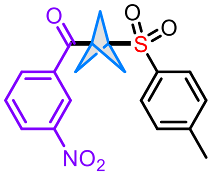

Obtained as a yellow solid (44 mg, 60% yield); M. P. = 163-164 °C; ^{1}H NMR (500 MHz, $CDCl_3$) δ 10.02 (s, 1H), 8.19 (d, *J* = 1.2 Hz, 1H), 8.03 (dd, *J* = 7.9, 1.3 Hz, 1H), 7.76 (d, *J* = 8.2 Hz, 2H), 7.46 (d, *J* = 7.9 Hz, 1H), 7.38 (d, *J* = 8.0 Hz, 2H), 2.47 (s, 3H), 2.46 (s, 6H); ^{13}C NMR (126 MHz, $CDCl_3$) δ 189.1, 149.8, 145.2, 137.0, 136.4, 133.4, 132.5, 131.3, 130.0, 128.7, 125.0, 52.7, 51.6, 39.6, 21.7; HRMS (ESI+): Calculated for $C_{19}H_{18}NO_5S$: [M+H]+ 372.0900, Found 372.0900.

Pyridin-4-yl(3-tosylbicyclo[1.1.1]pentan-1-yl)methanone (48)

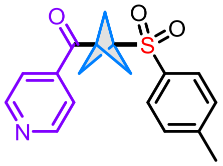

Obtained as a yellow solid (35 mg, 53% yield); M. P. = 126-127 °C; ^{1}H NMR (400 MHz, $CDCl_3$) δ 8.79 (dd, *J* = 4.5, 1.5 Hz, 2H), 7.76 (d, *J* = 8.2 Hz, 2H), 7.66 – 7.61 (m, 2H), 7.39 (d, *J* = 8.0 Hz, 2H), 2.52 (s, 6H), 2.47 (s, 3H); ^{13}C NMR (101 MHz, $CDCl_3$) δ 194.6, 151.0, 150.5, 145.4, 141.4, 133.1, 130.1, 129.8, 128.7, 121.5, 53.2, 49.6, 42.3, 21.8; HRMS (ESI+): Calculated for $C_{18}H_{18}NO_3S$: [M+H]+ 328.1002, Found 328.0998.

Phenyl(3-tosylbicyclo[1.1.1]pentan-1-yl)sulfane (49)

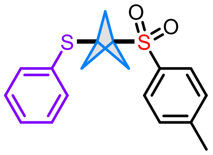

Obtained as a white solid (54 mg, 82% yield); M. P. = 127-128 °C; ^{1}H NMR (500 MHz, $CDCl_3$) δ 7.68 (d, *J* = 8.2 Hz, 2H), 7.38 (dd, *J* = 7.3, 2.2 Hz, 2H), 7.32 (dd, *J* = 10.5, 5.6 Hz, 5H), 2.43 (s, 3H), 2.10 (s, 6H); ^{13}C NMR (126 MHz, $CDCl_3$) δ 144.9, 134.5, 133.8, 131.6, 129.9, 129.2, 128.6, 128.6, 53.8, 52.4, 42.4, 21.7; HRMS (ESI+): Calculated for $C_{18}H_{18}O_2S_2Na$: $[M+Na]^+$ 353.0640, Found 353.0646.

2-((3-(Phenylthio)bicyclo[1.1.1]pentan-1-yl)sulfonyl)thiophene (50)

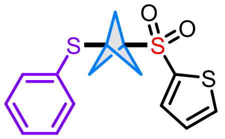

Obtained as a yellow solid (49 mg, 76% yield); M. P. = 72-73 °C; ^{1}H NMR (500 MHz, $CDCl_3$) δ 7.74 – 7.72 (m, 1H), 7.63 – 7.60 (m, 1H), 7.43 – 7.38 (m, 2H), 7.35 – 7.29 (m, 3H), 7.18 – 7.14 (m, 1H), 2.17 (s, 6H); ^{13}C NMR (126 MHz, $CDCl_3$) δ 137.6, 134.7, 134.6, 134.5, 131.5, 129.2, 128.7, 128.1, 53.9, 53.1, 41.9; HRMS (ESI+): Calculated for $C_{15}H_{14}O_2S_3Na$: $[M+Na]^+$ 345.0048, Found 345.0054.

3,5-Dimethyl-4-((3-(phenylthio)bicyclo[1.1.1]pentan-1-yl)sulfonyl)isoxazole (51)

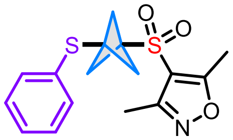

Obtained as a yellow solid (42 mg, 63% yield); M. P. = 82-83 °C; ^{1}H NMR (500 MHz, $CDCl_3$) δ 7.43 – 7.39 (m, 2H), 7.35 (t, *J* = 5.7 Hz, 3H), 2.59 (s, 3H), 2.35 (s, 3H), 2.17 (s, 6H); ^{13}C NMR (126 MHz, $CDCl_3$) δ 175.4, 158.4, 134.6, 131.2, 129.3, 128.9, 113.2, 53.4, 49.9, 42.4, 12.6, 10.9; HRMS (ESI+): Calculated for $C_{16}H_{17}NO_3S_2Na$: $[M+Na]^+$ 358.0542, Found 358.0548.

4--Tolyl(3-tosylbicyclo[1.1.1]pentan-1-yl)sulfane (52)

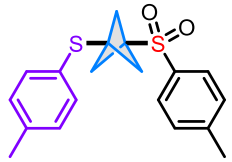

Obtained as a yellow solid (55 mg, 80% yield); M. P. = 79-80 °C; ^{1}H NMR (500 MHz, $CDCl_3$) δ 7.68 (d, J = 8.2 Hz, 2H), 7.32 (d, J = 8.0 Hz, 2H), 7.29 – 7.25 (m, 2H), 7.11 (d, J = 7.8 Hz, 2H), 2.44 (s, 3H), 2.33 (s, 3H), 2.08 (s, 6H); ^{13}C NMR (126 MHz, $CDCl_3$) δ 144.9, 138.9, 134.7, 133.8, 130.0, 129.9, 128.6, 127.9, 53.7, 52.3, 42.5, 21.7, 21.2; HRMS (ESI+): Calculated for $C_{19}H_{20}O_2S_2Na$: $[M+Na]^+$ 367.0796, Found 367.0802.

Phenyl(3-tosylbicyclo[1.1.1]pentan-1-yl)selane (53)

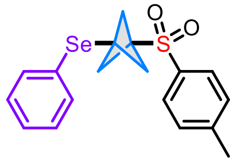

Obtained as a yellow solid (56 mg, 75% yield); M. P. = 119-120 °C; ^{1}H NMR (500 MHz, $CDCl_3$) δ 7.68 (d, J = 8.2 Hz, 2H), 7.52 – 7.47 (m, 2H), 7.33 (d, J = 7.9 Hz, 3H), 7.28 (d, J = 7.6 Hz, 2H), 2.44 (s, 3H), 2.14 (s, 6H); ^{13}C NMR (126 MHz, $CDCl_3$) δ 144.9, 135.9, 133.8, 129.9, 129.2, 128.6, 128.5, 127.0, 54.6, 53.8, 33.8, 21.7; HRMS (ESI+): Calculated for $C_{18}H_{18}O_2SSeNa$: [M+Na]+ 401.0091, Found 401.0095.

1-(4-Methoxyphenyl)-2-(3-tosylbicyclo[1.1.1]pentan-1-yl)diazene (54)

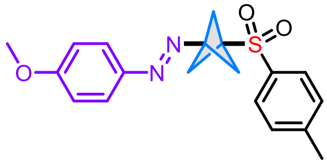

Obtained as a yellow solid (36 mg, 50% yield); M. P. = 149-150 °C; ^{1}H NMR (500 MHz, $CDCl_3$) δ 7.80 (d, J = 8.1 Hz, 2H), 7.68 (d, J = 8.9 Hz, 2H), 7.38 (d, J = 8.0 Hz, 2H), 6.93 (d, J = 8.9 Hz, 2H), 3.86 (s, 3H), 2.47 (s, 3H), 2.43 (s, 6H); ^{13}C NMR (126 MHz, $CDCl_3$) δ 162.5, 146.2, 144.9, 134.0, 129.9, 128.7, 124.6, 114.1, 61.4, 55.6, 52.8, 50.1, 21.7; HRMS (ESI+): Calculated for $C_{19}H_{21}N_2O_3S$: $[M+H]^+$ 357.1267, Found 357.1264.

1-(3-((4-Bromophenyl)sulfonyl)bicyclo[1.1.1]pentan-1-yl)-2-(4-methoxyphenyl)diazene (55)

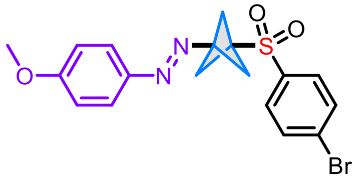

Obtained as a yellow solid (36 mg, 43% yield); M. P. = 157-158 °C; ^{1}H NMR (400 MHz, $CDCl_3$) δ 7.78 (d, J = 8.8 Hz, 2H), 7.74 (d, J = 4.9 Hz, 2H), 7.68 (d, J = 9.1 Hz, 2H), 6.94 (d, J = 9.1 Hz, 2H), 3.86 (s, 3H), 2.44 (s, 6H); ^{13}C NMR (101 MHz, $CDCl_3$) δ 162.6, 146.2, 136.1, 132.7, 130.2, 129.4, 124.7, 114.2, 61.5, 55.7, 52.9, 50.2; HRMS (ESI+): Calculated for $C_{18}H_{18}BrN_2O_3S$: $[M+H]^+$ 421.0216, Found 421.0210.

1-(4-Methoxyphenyl)-2-(3-(thiophen-2-ylsulfonyl)bicyclo[1.1.1]pentan-1-yl)diazene (56)

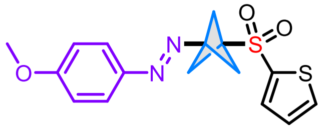

Obtained as a yellow solid (37 mg, 54% yield); M. P. = 143-144 °C; ^{1}H NMR (400 MHz, $CDCl_3$) δ 7.77 (dd, J = 5.0, 1.3 Hz, 1H), 7.73 (dd, J = 3.8, 1.3 Hz, 1H), 7.71 – 7.68 (m, 2H), 7.21 (dd, J = 4.9, 3.8 Hz, 1H), 6.97 – 6.93 (m, 2H), 3.87 (s, 3H), 2.50 (s, 6H); ^{13}C NMR (101 MHz, $CDCl_3$) δ 162.5, 146.2, 137.8, 134.7, 134.6, 128.1, 124.6, 114.2, 60.9, 55.6, 53.0, 51.0; $C_{16}H_{17}N_2O_3S_2$: $[M+H]^+$ 349.0675, Found 349.0671.

1-(3-(Cyclopropylsulfonyl)bicyclo[1.1.1]pentan-1-yl)-2-(4-methoxyphenyl)diazene (57)

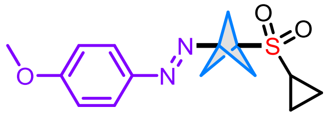

Obtained as a yellow solid (37 mg, 60% yield); M. P. = 152-153 °C; ^{1}H NMR (400 MHz, $CDCl_3$) δ 7.74 (d, J = 9.0 Hz, 2H), 6.97 (d, J = 8.9 Hz, 2H), 3.88 (s, 3H), 2.64 (s, 6H), 2.41 (dd, J = 6.5, 3.1 Hz, 1H), 1.28 (dd, J = 4.7, 2.1 Hz, 2H), 1.08 (dd, J = 7.8, 1.7 Hz, 2H); ^{13}C NMR (101 MHz, $CDCl_3$) δ 162.5, 146.2, 124.7, 114.2, 61.3, 55.6, 53.2, 48.8, 27.0, 4.3; HRMS (ESI+): Calculated for $C_{15}H_{19}N_2O_3S$: $[M+H]^+$ 307.1111, Found 307.1106.

1-Chloro-3-tosylbicyclo[1.1.1]pentane (58)

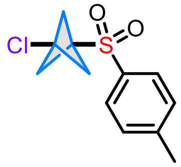

Obtained as white solid (46 mg, 90% yield); M. P. = 168-169 °C; ^{1}H NMR (500 MHz, $CDCl_3$) δ 7.72 (d, J = 8.3 Hz, 2H), 7.36 (d, J = 8.0 Hz, 2H), 2.46 (s, 3H), 2.38 (s, 6H); ^{13}C NMR (126 MHz, $CDCl_3$) δ 145.3, 133.7, 130.0, 128.5, 57.0, 49.7, 48.5, 21.7; HRMS (ESI+): Calculated for $C_{12}H_{13}ClO_2SNa$: $[M+Na]^+$ 279.0223, Found 279.0230.

1-Chloro-3-(phenylsulfonyl)bicyclo[1.1.1]pentane (59)

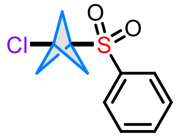

Obtained as white solid (42 mg, 87% yield); M. P. = 181-182 °C; ^{1}H NMR (500 MHz, $CDCl_3$) δ 7.89 – 7.81 (m, 2H), 7.68 (dd, J = 10.7, 4.2 Hz, 1H), 7.58 (t, J = 7.8 Hz, 2H), 2.39 (s, 6H); ^{13}C NMR (126 MHz, $CDCl_3$) δ 136.8, 134.2, 129.4, 128.5, 57.1, 49.7, 48.5; HRMS (ESI+): Calculated for $C_{11}H_{11}ClO_2SNa$: $[M+Na]^+$ 265.0066, Found 265.0071.

1-((4-(Tert-butyl)phenyl)sulfonyl)-3-chlorobicyclo[1.1.1]pentane (60)

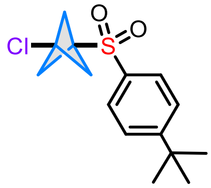

Obtained as white solid (44 mg, 74% yield); M. P. = 175-176 °C; ^{1}H NMR (500 MHz, $CDCl_3$) δ 7.76 (d, J = 8.5 Hz, 2H), 7.58 (d, J = 8.5 Hz, 2H), 2.41 (s, 6H), 1.36 (s, 9H); ^{13}C NMR (101 MHz, $CDCl_3$) δ 158.2, 133.7, 128.4, 126.4, 57.0, 49.7, 48.5, 35.4, 31.1; HRMS (ESI+): Calculated for $C_{15}H_{19}ClO_2SNa$: $[M+Na]^+$ 321.0692, Found 321.0690.

1-Chloro-3-((4-methoxyphenyl)sulfonyl)bicyclo[1.1.1]pentane (61)

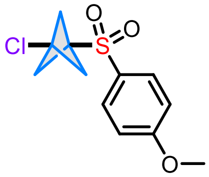

Obtained as white solid (51 mg, 93% yield); M. P. = 161-162 °C; ^{1}H NMR (500 MHz, $CDCl_3$) δ 7.76 (d, J = 8.9 Hz, 2H), 7.02 (d, J = 8.9 Hz, 2H), 3.89 (s, 3H), 2.38 (s, 6H); ^{13}C NMR (126 MHz, $CDCl_3$) δ 164.1, 130.7, 128.2, 114.6, 57.0, 55.7, 49.9, 48.5; HRMS (ESI+): Calculated for $C_{12}H_{14}ClO_3S$: $[M+H]^+$ 273.0353, Found 273.0355.

1-Chloro-3-((4-(trifluoromethoxy)phenyl)sulfonyl)bicyclo[1.1.1]pentane (62)

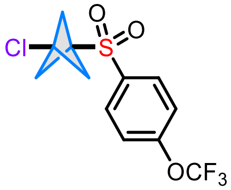

Obtained as white solid (58 mg, 89% yield); M. P. = 193-194 °C; ^{1}H NMR (500 MHz, $CDCl_3$) δ 7.91 (d, J = 8.8 Hz, 2H), 7.40 (d, J = 8.2 Hz, 2H), 2.41 (s, 6H); ^{13}C NMR (126 MHz, $CDCl_3$) δ 153.4, 135.0, 130.8, 121.0, 120.2 (q, J = 260.8 Hz), 57.1, 49.7, 48.4; ^{19}F NMR (377 MHz, $CDCl_3$) δ -57.64; HRMS (ESI+): Calculated for $C_{12}H_{10}ClF_3O_3SNa$: $[M+Na]^+$ 348.9889, Found 348.9901.

4-((3-Chlorobicyclo[1.1.1]pentan-1-yl)sulfonyl)benzonitrile (63)

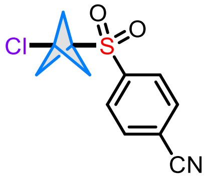

Obtained as white solid (39 mg, 72% yield); M. P. = 197-198 °C; ^{1}H NMR (400 MHz, $CDCl_3$) δ 7.99 (d, J = 8.4 Hz, 2H), 7.90 (d, J = 8.4 Hz, 2H), 2.43 (s, 6H); ^{13}C NMR (101 MHz, $CDCl_3$) δ 139.9, 132.1, 128.2, 117.0, 115.9, 56.1, 48.6, 47.3; HRMS (ESI+): Calculated for $C_{12}H_{10}ClNO_2SNa$: $[M+Na]^+$ 290.0019, Found 290.0021.

1-(4-((3-Chlorobicyclo[1.1.1]pentan-1-yl)sulfonyl)phenyl)ethan-1-one (64)

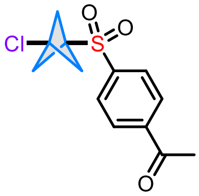

Obtained as white solid (43 mg, 75% yield); M. P. = 182-183 °C; ^{1}H NMR (500 MHz, $CDCl_3$) δ 8.13 (d, J = 8.4 Hz, 2H), 7.96 (d, J = 8.5 Hz, 2H), 2.68 (s, 3H), 2.42 (s, 6H); ^{13}C NMR (126 MHz,

CDCl3) δ 196.6, 141.2, 140.5, 129.1, 129.0, 57.1, 49.6, 48.4, 26.9; HRMS (ESI+): Calculated for $C_{13}H_{13}ClO_3SNa$: [M+Na]+ 307.0166, Found 307.0161.

1-Chloro-3-((4-(trifluoromethyl)phenyl)sulfonyl)bicyclo[1.1.1]pentane (65)

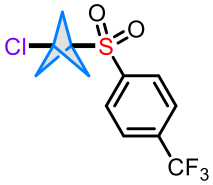

Obtained as white solid (50 mg, 80% yield); M. P. = 167-168 °C; ^{1}H NMR (500 MHz, $CDCl_3$) δ 8.00 (d, J = 8.2 Hz, 2H), 7.86 (d, J = 8.3 Hz, 2H), 2.42 (s, 6H); ^{13}C NMR (126 MHz, $CDCl_3$) δ 140.4, 135.9 (q, J = 34.0 Hz), 129.2, 126.6 (q, J = 3.8 Hz), 123.0 (q, J = 273.4 Hz), 57.1, 49.7, 48.4; ^{19}F NMR (471 MHz, $CDCl_3$) δ -63.24; HRMS (ESI+): Calculated for $C_{12}H_{11}ClF_3O_2S$: $[M+H]^+$ 311.0115, Found 311.0110.

1-Chloro-3-((4-fluorophenyl)sulfonyl)bicyclo[1.1.1]pentane (66)

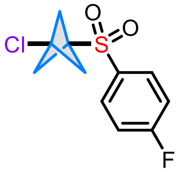

Obtained as white solid (43 mg, 83% yield); M. P. = 134-135 °C; ^{1}H NMR (500 MHz, $CDCl_3$) δ 7.88 (dd, J = 8.8, 5.0 Hz, 2H), 7.27 (t, J = 8.5 Hz, 2H), 2.40 (s, 6H); ^{13}C NMR (126 MHz, $CDCl_3$) δ 166.1 (d, J = 258.3 Hz), 132.8 (d, J = 3.8 Hz), 131.4 (d, J = 8.8 Hz), 116.8 (d, J = 22.7 Hz), 57.0, 49.8, 48.4; ^{19}F NMR (471 MHz, $CDCl_3$) δ -102.38; HRMS (ESI+): Calculated for $C_{11}H_{10}ClFO_2SNa$: $[M+Na]^+$ 282.9972, Found 282.9981.

1-((4-(Tert-butyl)phenyl)sulfonyl)-3-chlorobicyclo[1.1.1]pentane (67)

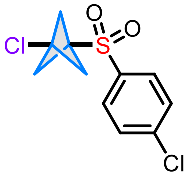

Obtained as white solid (42 mg, 76% yield); M. P. = 123-124 °C; ^{1}H NMR (500 MHz, $CDCl_3$) δ 7.79 (d, J = 8.5 Hz, 2H), 7.56 (d, J = 8.5 Hz, 2H), 2.40 (s, 6H); ^{13}C NMR (126 MHz, $CDCl_3$) δ 141.1, 135.2, 130.0, 129.8, 57.1, 49.7, 48.4; HRMS (ESI+): Calculated for $C_{11}H_{10}Cl_2O_2SNa$: $[M+Na]^+$ 298.9677.0692, Found 298.9681.

1-((4-Bromophenyl)sulfonyl)-3-chlorobicyclo[1.1.1]pentane (68)

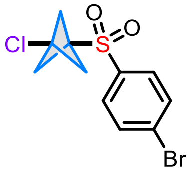

Obtained as white solid (47 mg, 73% yield); M. P. = 131-132 °C; ^{1}H NMR (500 MHz, $CDCl_3$) δ 7.72 (q, J = 8.7 Hz, 4H), 2.40 (s, 6H); ^{13}C NMR (126 MHz, $CDCl_3$) δ 134.7, 131.8, 129.0, 128.7,

56.0, 48.7, 47.4; HRMS (ESI+): Calculated for $C_{11}H_{11}BrClO_2S$: $[M+H]^+$ 320.9346, Found 320.9342.

1-Chloro-3-((4-iodophenyl)sulfonyl)bicyclo[1.1.1]pentane (69)

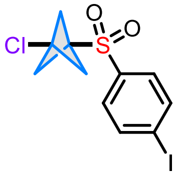

Obtained as white solid (35 mg, 47% yield); M. P. = 134-135 °C; ^{1}H NMR (500 MHz, $CDCl_3$) δ 7.95 (d, J = 8.5 Hz, 2H), 7.55 (d, J = 8.5 Hz, 2H), 2.40 (s, 6H); ^{13}C NMR (126 MHz, $CDCl_3$) δ 138.8, 136.4, 129.8, 102.4, 57.1, 49.7, 48.4; HRMS (ESI+): Calculated for $C_{11}H_{10}ClIO_2SNa$: $[M+Na]^+$ 390.9027, Found 390.9034.

1-Chloro-3-(o-tolylsulfonyl)bicyclo[1.1.1]pentane (70)

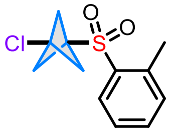

Obtained as white solid (45 mg, 88% yield); M. P. = 103-104 °C; ^{1}H NMR (500 MHz, $CDCl_3$) δ 7.94 – 7.88 (m, 1H), 7.53 (td, J = 7.5, 1.0 Hz, 1H), 7.37 (t, J = 7.7 Hz, 1H), 7.33 (d, J = 7.6 Hz, 1H), 2.64 (s, 3H), 2.41 (s, 6H); ^{13}C NMR (126 MHz, $CDCl_3$) δ 139.0, 134.9, 134.2, 132.9, 130.9, 126.8, 57.2, 50.1, 48.2, 20.8; HRMS (ESI+): Calculated for $C_{12}H_{14}ClO_2S$: $[M+H]^+$ 257.0404, Found 257.0412.

1-Chloro-3-((2-chlorophenyl)sulfonyl)bicyclo[1.1.1]pentane (71)

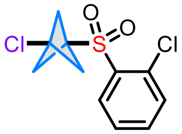

Obtained as white solid (41 mg, 73% yield); M. P. = 117-118 °C; ^{1}H NMR (500 MHz, $CDCl_3$) δ 8.06 (dd, J = 7.9, 1.5 Hz, 1H), 7.60 – 7.53 (m, 2H), 7.46 (ddd, J = 8.6, 7.2, 1.5 Hz, 1H), 2.51 (s, 6H); ^{13}C NMR (126 MHz, $CDCl_3$) δ 135.3, 135.0, 133.5, 132.4, 132.3, 127.6, 57.9, 50.0, 47.9; HRMS (ESI+): Calculated for $C_{11}H_{10}Cl_2O_2SNa$: $[M+Na]^+$ 298.9677, Found 298.9683.

1-((3-Bromophenyl)sulfonyl)-3-chlorobicyclo[1.1.1]pentane (72)

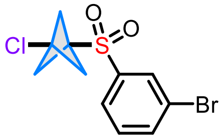

Obtained as white solid (50 mg, 78% yield); M. P. = 123-124 °C; ^{1}H NMR (500 MHz, $CDCl_3$) δ 7.99 (s, 1H), 7.79 (dd, J = 15.0, 7.9 Hz, 2H), 7.46 (t, J = 7.9 Hz, 1H), 2.41 (s, 6H); ^{13}C NMR (126 MHz, $CDCl_3$) δ 137.7, 136.3, 130.3, 129.9, 126.0, 122.4, 56.1, 48.7, 47.4; HRMS (ESI+): Calculated for $C_{11}H_{10}BrClO_2SNa$: $[M+Na]^+$ 342.9171, Found 342.9181.

1-Chloro-3-((3-(trifluoromethyl)phenyl)sulfonyl)bicyclo[1.1.1]pentane (73)

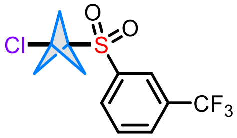

Obtained as white solid (50 mg, 81% yield); M. P. = 113-114 °C; ^{1}H NMR (500 MHz, $CDCl_3$) δ 8.12 (s, 1H), 8.06 (d, J = 7.8 Hz, 1H), 7.95 (d, J = 7.8 Hz, 1H), 7.76 (t, J = 7.8 Hz, 1H), 2.42 (s, 6H); ^{13}C NMR (126 MHz, $CDCl_3$) δ 138.1, 132.3 (q, J = 34.0 Hz), 131.8, 130.9 (q, J = 3.8 Hz), 130.3, 125.6 (q, J = 3.8 Hz), 123.0 (q, J = 273.4 Hz), 57.1, 49.7, 48.4; ^{19}F NMR (471 MHz, $CDCl_3$) δ -62.87; HRMS (ESI+): Calculated for $C_{12}H_{11}ClF_3O_2S$: $[M+H]^+$ 311.0121, Found 311.0121.

1-Chloro-3-((3,5-difluorophenyl)sulfonyl)bicyclo[1.1.1]pentane (74)

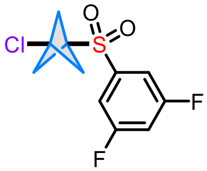

Obtained as white solid (38 mg, 69% yield); M. P. = 193-194 °C; ^{1}H NMR (500 MHz, $CDCl_3$) δ 7.40 (d, J = 3.8 Hz, 2H), 7.18 – 7.12 (m, 1H), 2.44 (s, 6H); ^{13}C NMR (126 MHz, $CDCl_3$) δ 163.0 (dd, J = 256.5, 11.4 Hz), 140.2, 112.1 (dd, J = 20.9, 7.4 Hz), 110.0 (t, J = 25.0 Hz), 57.2, 49.5, 48.3; ^{19}F NMR (471 MHz, $CDCl_3$) δ -104.27. HRMS (ESI+): Calculated for $C_{11}H_9ClF_2O_2SNa$: $[M+Na]^+$ 300.9878, Found 300.9878.

1-Chloro-3-((3,5-dichlorophenyl)sulfonyl)bicyclo[1.1.1]pentane (75)

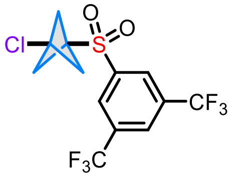

Obtained as white solid (48 mg, 64% yield); M. P. = 133-134 °C; ^{1}H NMR (500 MHz, $CDCl_3$) δ 8.30 (s, 2H), 8.19 (s, 1H), 2.46 (s, 6H); ^{13}C NMR (126 MHz, $CDCl_3$) δ 139.8, 133.5 (q, J = 34.8 Hz), 128.8 (q, J = 3.2 Hz), 127.9 (q, J =3.8 Hz), 122.2 (q, J = 273.5 Hz), 57.2, 49.7, 48.3; ^{19}F NMR (471 MHz, $CDCl_3$) δ -62.92; HRMS (ESI+): Calculated for $C_{13}H_9ClF_6O_2SNa$: $[M+Na]^+$ 400.9814, Found 400.9822.

1-Chloro-3-(naphthalen-1-ylsulfonyl)bicyclo[1.1.1]pentane (76)

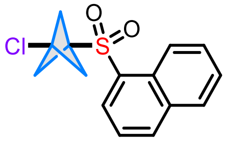

Obtained as white solid (45 mg, 77% yield); M. P. = 153-154 °C; ^{1}H NMR (500 MHz, $CDCl_3$) δ 8.45 (s, 1H), 8.02 (dd, J = 8.2, 3.8 Hz, 2H), 7.96 (d, J = 8.1 Hz, 1H), 7.80 (dd, J = 8.6, 1.6 Hz, 1H), 7.71 (t, J = 7.1 Hz, 1H), 7.66 (t, J = 7.2 Hz, 1H), 2.43 (s, 6H); ^{13}C NMR (126 MHz, $CDCl_3$) δ 135.5, 133.7, 132.2, 130.4, 129.6, 129.6, 129.5, 128.1, 127.9, 123.0, 57.2, 49.8, 48.5; HRMS (ESI+): Calculated for $C_{15}H_{13}ClO_2SNa$: $[M+Na]^+$ 315.0223, Found 315.0231.

8-((3-Chlorobicyclo[1.1.1]pentan-1-yl)sulfonyl)quinoline (77)

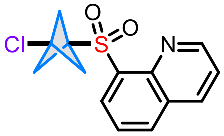

Obtained as white solid (44 mg, 75% yield); M. P. = 143-144 °C; ^{1}H NMR (500 MHz, $CDCl_3$) δ 9.12 (dd, *J* = 4.2, 1.6 Hz, 1H), 8.53 (dd, *J* = 7.3, 1.2 Hz, 1H), 8.28 (dd, *J* = 8.3, 1.5 Hz, 1H), 8.18 – 8.10 (m, 1H), 7.70 (t, *J* = 7.8 Hz, 1H), 7.57 (dd, *J* = 8.3, 4.2 Hz, 1H), 2.55 (s, 6H); ^{13}C NMR (126 MHz, $CDCl_3$) δ 151.6, 144.5, 136.5, 135.5, 135.0, 133.2, 129.0, 125.8, 122.5, 58.4, 50.9, 48.0; HRMS (ESI+): Calculated for $C_{14}H_{13}ClNO_2S$: $[M+H]^+$ 294.0356, Found 294.0361.

2-((3-Chlorobicyclo[1.1.1]pentan-1-yl)sulfonyl)thiophene (78)

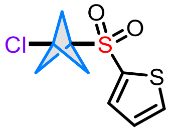

Obtained as white solid (35 mg, 71% yield); M. P. = 123-124 °C; ^{1}H NMR (500 MHz, $CDCl_3$) δ 7.77 (dd, *J* = 5.0, 1.3 Hz, 1H), 7.66 (dd, *J* = 3.8, 1.3 Hz, 1H), 7.19 (dd, *J* = 4.9, 3.8 Hz, 1H), 2.45 (s, 6H); ^{13}C NMR (126 MHz, $CDCl_3$) δ 137.5, 135.0, 134.9, 128.2, 57.2, 50.5, 48.1; HRMS (ESI+): Calculated for $C_9H_9ClO_2S_2Na$: $[M+Na]^+$ 270.9630, Found 270.9641.

4-((3-Chlorobicyclo[1.1.1]pentan-1-yl)sulfonyl)-3,5-dimethylisoxazole (79)

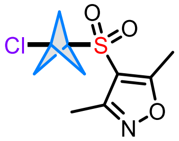

Obtained as white solid (44 mg, 85% yield); M. P. = 142-143 °C; ^{1}H NMR (500 MHz, $CDCl_3$) δ 2.62 (s, 3H), 2.46 (s, 6H), 2.38 (s, 3H); ^{13}C NMR (126 MHz, $CDCl_3$) δ 175.6, 158.2, 113.4, 56.7, 50.6, 48.1, 12.6, 10.9; HRMS (ESI+): Calculated for $C_{10}H_{13}ClNO_3S$: $[M+H]^+$ 262.0305, Found 262.0304.

6-((3-Chlorobicyclo[1.1.1]pentan-1-yl)sulfonyl)benzo[d]thiazole (80)

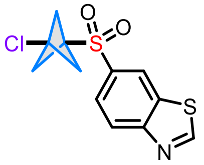

Obtained as white solid (38 mg, 64% yield); M. P. = 193-194 °C; ^{1}H NMR (500 MHz, $CDCl_3$) δ 9.28 (s, 1H), 8.55 (d, *J* = 1.7 Hz, 1H), 8.32 (d, *J* = 8.6 Hz, 1H), 7.97 (dd, *J* = 8.6, 1.8 Hz, 1H), 2.44 (s, 6H); ^{13}C NMR (126 MHz, $CDCl_3$) δ 158.8, 134.0, 128.4, 125.9, 125.9, 124.6, 123.6, 57.2, 50.0, 48.5; HRMS (ESI+): Calculated for $C_{12}H_{11}ClNO_2S_2$: $[M+H]^+$ 300.7949, Found 300.7952.

1-Chloro-3-(cyclopropylsulfonyl)bicyclo[1.1.1]pentane (81)

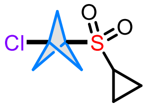

Obtained as colorless liquid (24 mg, 58% yield); ^{1}H NMR (500 MHz, $CDCl_3$) δ 2.59 (s, 6H), 2.36 – 2.29 (m, 1H), 1.23 (dd, *J* = 4.6, 2.1 Hz, 2H), 1.07 (dd, *J* = 7.7, 2.1 Hz, 2H); ^{13}C NMR (126 MHz,

$CDCl_3$) δ 57.5, 50.0, 48.4, 27.4, 4.5; HRMS (ESI+): Calculated for $C_8H_{12}ClO_2S$: $[M+H]^+$ 207.0247, Found 207.0253.

1-Bromo-3-tosylbicyclo[1.1.1]pentane (82)

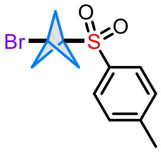

Obtained as a white solid (50 mg, 83% yield); M. P. = 161-162 °C; ^{1}H NMR (400 MHz, $CDCl_3$) δ 7.72 (d, *J* = 8.2 Hz, 2H), 7.37 (d, *J* = 8.0 Hz, 2H), 2.46 (s, 3H), 2.45 (s, 6H); ^{13}C NMR (101 MHz, $CDCl_3$) δ 145.4, 133.7, 130.1, 128.5, 57.9, 52.5, 35.3, 21.7.

4-((3-Bromobicyclo[1.1.1]pentan-1-yl)sulfonyl)benzonitrile (83)

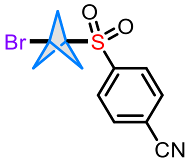

Obtained as a white solid (42 mg, 68% yield); M. P. = 242-243 °C; ^{1}H NMR (400 MHz, Chloroform-*d*) δ 7.99 (d, *J* = 8.3 Hz, 2H), 7.90 (d, *J* = 8.3 Hz, 2H), 2.48 (s, 6H); ^{13}C NMR (101 MHz, $CDCl_3$) δ 141.0, 133.2, 129.3, 118.1, 116.9, 57.9, 52.4, 34.8.

1-Iodo-3-tosylbicyclo[1.1.1]pentane (84)

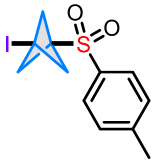

Obtained as a white solid (63 mg, 90% yield); M. P. = 187-188 °C; ^{1}H NMR (500 MHz, $CDCl_3$) δ 7.71 (d, *J* = 8.2 Hz, 2H), 7.36 (d, *J* = 8.0 Hz, 2H), 2.50 (s, 6H), 2.46 (s, 3H); ^{13}C NMR (126 MHz, $CDCl_3$) δ 145.3, 133.7, 130.1, 128.6, 59.2, 57.8, 21.7, 2.5; HRMS (ESI+): Calculated for $C_{12}H_{13}IO_2SNa$: $[M+Na]^+$ 370.9573, Found 370.9577.

1-((3-Bromophenyl)sulfonyl)-3-iodobicyclo[1.1.1]pentane (85)

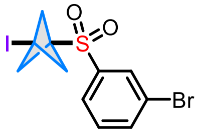

Obtained as a yellow solid (65 mg, 79% yield); M. P. = 223-224 °C; ^{1}H NMR (500 MHz, $CDCl_3$) δ 7.99 (t, *J* = 1.7 Hz, 1H), 7.83 – 7.80 (m, 1H), 7.78 (d, *J* = 7.8 Hz, 1H), 7.46 (t, *J* = 7.9 Hz, 1H), 2.53 (s, 6H); ^{13}C NMR (126 MHz, $CDCl_3$) δ 138.6, 137.3, 131.4, 130.9, 127.1, 123.6, 59.2, 57.7, 1.8; HRMS (ESI+): Calculated for $C_{11}H_{10}BrIO_2SNa$: $[M+Na]^+$ 434.8528, Found 434.8531.

3-Tosylbicyclo[1.1.1]pentane-1-carbonitrile (86)

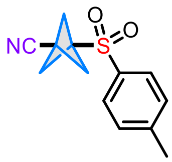

Obtained as a yellow solid (35 mg, 71% yield); M. P. = 188-189 °C; ^{1}H NMR (500 MHz, $CDCl_3$) δ 7.71 (d, *J* = 8.2 Hz, 2H), 7.39 (d, *J* = 8.0 Hz, 2H), 2.48 (s, 9H); ^{13}C NMR (126 MHz, $CDCl_3$) δ 145.7, 132.7, 130.2, 128.7, 115.6, 54.1, 54.0, 23.5, 21.7; HRMS (ESI+): Calculated for $C_{13}H_{14}NO_2S$: $[M+H]^+$ 248.0746, Found 248.0744.

(3-((4-Bromophenyl)sulfonyl)bicyclo[1.1.1]pentan-1-yl)(methyl-d3)sulfane (87)

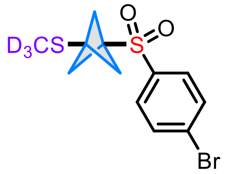

Obtained as a yellow solid (47 mg, 87% yield); M. P. = 92-93 °C; ^{1}H NMR (500 MHz, $CDCl_3$) δ 7.69 (d, *J* = 9.8 Hz, 4H), 2.14 (s, 6H); ^{13}C NMR (126 MHz, $CDCl_3$) δ 135.8, 132.6, 130.1, 129.4, 52.8, 51.9, 49.8, 41.3; HRMS (ESI+): Calculated for $C_{12}H_{11}D_3BrO_2S_2$: $[M+H]^+$ 335.9808, Found 335.9818.

1-(4-((3-Chlorobicyclo[1.1.1]pentan-1-yl)sulfonyl)phenyl)-5-(p-tolyl)-3-(trifluoromethyl)-1H-pyrazole (88)

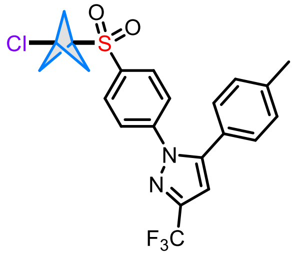

Obtained as a white solid (80 mg, 86% yield); M. P. = 193-194 °C; ^{1}H NMR (500 MHz, $CDCl_3$) δ 7.84 (d, *J* = 8.6 Hz, 2H), 7.55 (d, *J* = 8.6 Hz, 2H), 7.19 (d, *J* = 8.0 Hz, 2H), 7.10 (d, *J* = 8.1 Hz, 2H), 6.76 (s, 1H), 2.39 (s, 9H); ^{13}C NMR (126 MHz, $CDCl_3$) δ 145.5, 144.4 (q, *J* = 39.0 Hz), 143.8, 140.0, 136.0, 129.8, 129.6, 128.7, 125.7, 125.6, 121.0 (q, *J* = 269.6 Hz), 106.6, 57.1, 49.7, 48.4, 21.4; ^{19}F NMR (471 MHz, $CDCl_3$) δ -62.50; HRMS (ESI+): Calculated for $C_{22}H_{18}ClF_3N_2O_2SNa$: $[M+Na]^+$ 489.0628, Found 489.0633.

2-(2-Methyl-3,5-dioxo-6-(3-tosylbicyclo[1.1.1]pentan-1-yl)-2,5-dihydro-1,2,4-triazin-4(3H)-yl)ethyl 2-(4-isobutylphenyl)propanoate (89)

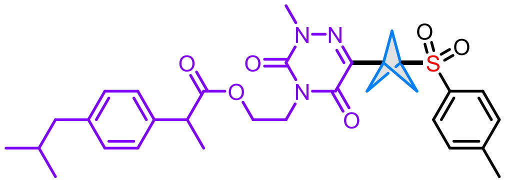

Obtained as a light yellow liquid (79 mg, 68% yield); ^{1}H NMR (500 MHz, $CDCl_3$) δ 7.76 (d, *J* = 8.2 Hz, 2H), 7.37 (d, *J* = 8.0 Hz, 2H), 7.10 (d, *J* = 8.1 Hz, 2H), 7.03 (d, *J* = 8.1 Hz, 2H), 4.42 – 4.35 (m, 1H), 4.30 (t, *J* = 9.4 Hz, 1H), 4.26 – 4.19 (m, 1H), 4.15 – 4.07 (m, 1H), 3.61 (q, *J* = 7.1

Hz, 1H), 3.24 (s, 3H), 2.47 (s, 3H), 2.42 (d, J = 7.2 Hz, 2H), 2.35 (s, 6H), 1.85 – 1.77 (m, 1H), 1.43 (d, J = 7.2 Hz, 3H), 0.89 (d, J = 6.6 Hz, 6H); ^{13}C NMR (126 MHz, $CDCl_3$) δ 174.4, 155.3, 149.0, 144.9, 140.7, 139.7, 137.3, 133.6, 129.9, 129.3, 128.7, 127.0, 61.2, 52.5, 52.0, 50.4, 45.0, 45.0, 37.8, 30.2, 26.9, 22.4, 21.7, 18.5; HRMS (ESI+): Calculated for $C_{31}H_{37}N_3O_6SNa$: $[M+Na]^+$ 602.2295, Found 602.2298.

2-(4-Chlorophenyl)-2-(2,6-dichloro-4-(4-methyl-3,5-dioxo-6-(3-tosylbicyclo[1.1.1]pentan-1-yl)-4,5-dihydro-1,2,4-triazin-2(3H)-yl)phenyl)acetonitrile (90)

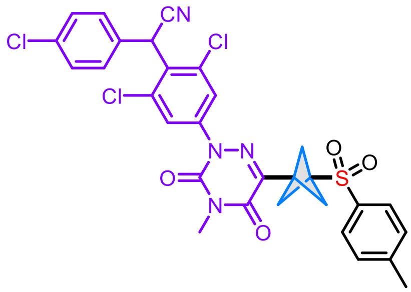

Obtained as a white solid (82 mg, 64% yield); M. P. = 214-215 °C; ^{1}H NMR (500 MHz, $CDCl_3$) δ 7.75 (d, J = 8.2 Hz, 2H), 7.66 (s, 2H), 7.37 (d, J = 8.1 Hz, 2H), 7.35 (d, J = 8.6 Hz, 2H), 7.29 (d, J = 8.5 Hz, 2H), 6.17 (s, 1H), 3.37 (s, 3H), 2.47 (s, 3H), 2.44 (s, 6H); ^{13}C NMR (126 MHz, $CDCl_3$) δ 154.6, 147.9, 145.1, 141.8, 141.2, 135.8, 134.7, 133.5, 130.7, 130.2, 130.0, 129.1, 128.7, 128.2, 125.0, 116.2, 52.5, 52.2, 37.8, 37.0, 27.4, 21.7; HRMS (ESI+): Calculated for $C_{30}H_{23}Cl_3N_4O_4Na$: $[M+Na]^+$ 663.0398, Found 663.0390.

4-(4-((3-Chlorobicyclo[1.1.1]pentan-1-yl)sulfonyl)phenyl)-5-methyl-3-phenylisoxazole (91)

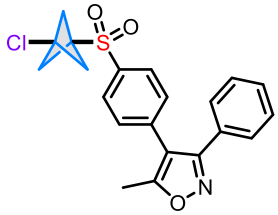

Obtained as white solid (50 mg, 63% yield); M. P. = 159-160 °C; ^{1}H NMR (500 MHz, $CDCl_3$) δ 7.84 (d, J = 8.3 Hz, 2H), 7.42 (dd, J = 8.6, 4.2 Hz, 1H), 7.38 (d, J = 8.3 Hz, 2H), 7.35 (d, J = 4.4 Hz, 4H), 2.53 (s, 3H), 2.43 (s, 6H); ^{13}C NMR (126 MHz, $CDCl_3$) δ 167.4, 136.9, 135.8, 130.5, 129.9, 128.9, 128.8, 128.5, 128.3, 114.3, 100,0, 57.1, 49.7, 48.4, 11.9; HRMS (ESI+): Calculated for $C_{21}H_{18}ClNO_3SNa$: $[M+Na]^+$ 422.0594, Found 422.0599.

5-(5-(Bicyclo[1.1.1]pentan-1-ylsulfonyl)-2-ethoxyphenyl)-1-methyl-3-propyl-1,6-dihydro-7H-pyrazolo[4,3-d]pyrimidin-7-one (92)

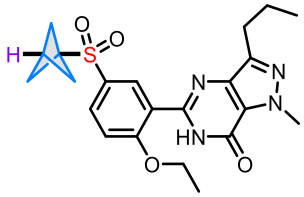

Obtained as a white solid (56 mg, 63% yield); M. P. = 143-144 °C; ^{1}H NMR (500 MHz, $CDCl_3$) δ 10.81 (s, 1H), 8.89 (d, J = 2.3 Hz, 1H), 7.91 (dd, J = 8.7, 2.3 Hz, 1H), 7.17 (d, J = 8.8 Hz, 1H), 4.38 (q, J = 6.9 Hz, 2H), 4.26 (s, 3H), 2.93 (t, J = 7.5 Hz, 2H), 2.75 (s, 1H), 2.13 (s, 6H), 1.86 (dd,

J = 14.9, 7.4 Hz, 2H), 1.64 (t, J = 6.9 Hz, 3H), 1.02 (t, J = 7.4 Hz, 3H); ^{13}C NMR (126 MHz, $CDCl_3$) δ 159.9, 153.6, 147.1, 146.4, 138.4, 132.4, 132.2, 130.2, 124.5, 121.3, 113.1, 66.2, 55.2, 50.5, 38.2, 27.7, 26.9, 22.3, 14.6, 14.0; HRMS (ESI+): Calculated for $C_{22}H_{27}N_4O_4S$: $[M+H]^+$ 443.1754, Found 443.1760.

2-(2-Methyl-3,5-dioxo-6-(3-tosylbicyclo[1.1.1]pentan-1-yl)-2,5-dihydro-1,2,4-triazin-4(3H)-yl)ethyl 5-(2,5-dimethylphenoxy)-2,2-dimethylpentanoate (93)

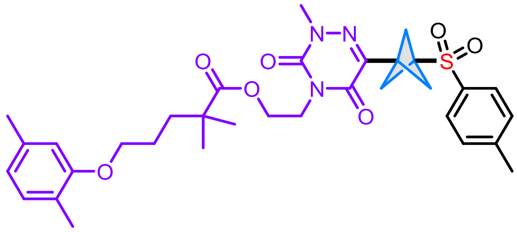

Obtained as white solid (87 mg, 70% yield); M. P. = 180-181 °C; ^{1}H NMR (500 MHz, $CDCl_3$) δ 7.74 (d, J = 8.2 Hz, 2H), 7.36 (d, J = 8.0 Hz, 2H), 7.00 (d, J = 7.5 Hz, 1H), 6.66 (d, J = 7.5 Hz, 1H), 6.59 (s, 1H), 4.35 (t, J = 5.2 Hz, 2H), 4.19 (t, J = 5.2 Hz, 2H), 3.88 (t, J = 4.6 Hz, 2H), 3.26 (s, 3H), 2.46 (s, 3H), 2.37 (s, 6H), 2.30 (s, 3H), 2.15 (s, 3H), 1.67 (d, J = 2.8 Hz, 4H), 1.16 (s, 6H); ^{13}C NMR (126 MHz, $CDCl_3$) δ 177.5, 156.9, 155.4, 149.0, 144.9, 139.7, 136.5, 133.6, 130.3, 129.9, 128.7, 123.5, 120.79, 111.98, 67.8, 61.1, 52.5, 52.1, 51.1, 42.1, 37.8, 36.9, 27.0, 25.1, 21.7, 21.7, 21.4, 15.8; HRMS (ESI+): Calculated for $C_{33}H_{41}N_3O_7SNa$: $[M+Na]^+$ 646.2557, Found 646.2553.

1-([1,1'-Biphenyl]-4-ylsulfonyl)bicyclo[1.1.1]pentane (94)

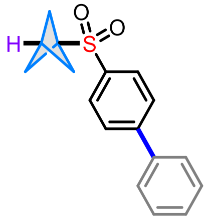

Obtained as a white solid (47 mg, 83% yield); M. P. = 156-157 °C; ^{1}H NMR (500 MHz, $CDCl_3$) δ 7.91 (d, J = 8.4 Hz, 2H), 7.76 (d, J = 8.4 Hz, 2H), 7.64 – 7.61 (m, 2H), 7.49 (t, J = 7.4 Hz, 2H), 7.43 (t, J = 7.3 Hz, 1H), 2.75 (s, 1H), 2.12 (s, 6H); ^{13}C NMR (126 MHz, $CDCl_3$) δ 146.5, 139.2, 135.4, 129.2, 129.1, 128.7, 127.7, 127.4, 55.2, 50.5, 26.8; HRMS (ESI+): Calculated for $C_{17}H_{16}O_2SNa$: $[M+Na]^+$ 307.0769, Found 307.0777.

***N*-(4-(4-((3-Chlorobicyclo[1.1.1]pentan-1-yl)sulfonyl)phenoxy)phenyl)acetamide (95)**

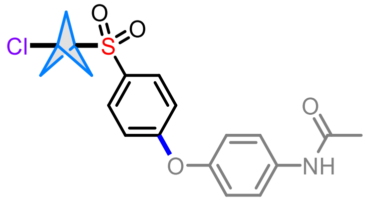

Obtained as a white solid (61 mg, 78% yield); M. P. = 193-194 °C; ^{1}H NMR (500 MHz, $CDCl_3$) δ 7.76 (d, J = 8.7 Hz, 2H), 7.57 (d, J = 8.7 Hz, 2H), 7.31 (s, 1H), 7.05 (d, J = 8.5 Hz, 4H), 2.40 (s, 6H), 2.20 (s, 3H); ^{13}C NMR (126 MHz, $CDCl_3$) δ 168.3, 163.2, 150.7, 135.3, 130.8, 129.9, 121.8, 121.2, 117.3, 57.0, 49.8, 48.5, 24.5. HRMS (ESI+): Calculated for $C_{19}H_{18}ClNO_4SNa$: $[M+Na]^+$ 414.0543, Found 414.0551.

4,4,5,5-Tetramethyl-2-(3-tosylbicyclo[1.1.1]pentan-1-yl)-1,3,2-dioxaborolane (96)

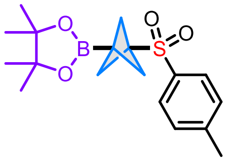

Obtained as a yellow solid (21 mg, 30% yield); M. P. = 127-128 °C; ^{1}H NMR (500 MHz, $CDCl_3$) δ 7.70 (d, J = 8.3 Hz, 2H), 7.37 (d, J = 8.3 Hz, 2H), 2.40 (s, 3H), 2.38 (s, 6H), 1.23 (s, 12H); ^{13}C NMR (126 MHz, $CDCl_3$) δ 146.6, 133.1, 129.9, 128.5, 83.9, 57.0, 49.7, 48.5, 24.9, 24.7; ^{11}B NMR (128 MHz, $CDCl_3$) δ 32.14; HRMS (ESI+): Calculated for $C_{18}H_{25}BO_4SNa$: $[M+Na]^+$ 371.1465, Found 371.1468.

4,4,5,5-Tetramethyl-2-(4-((3-(4,4,5,5-tetramethyl-1,3,2-dioxaborolan-2-yl)bicyclo[1.1.1]pentan-1-yl)sulfonyl)benzyl)-1,3,2-dioxaborolane (97)

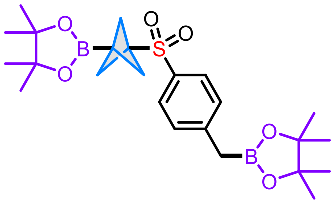

Obtained as a yellow solid (41 mg, 43% yield); M. P. = 146-147 °C; ^{1}H NMR (500 MHz, $CDCl_3$) δ 7.67 (d, J = 8.3 Hz, 2H), 7.32 (d, J = 8.3 Hz, 2H), 2.36 (s, 2H), 2.09 (s, 6H), 1.22 (s, 12H), 1.19 (s, 12H); ^{13}C NMR (126 MHz, $CDCl_3$) δ 145.7, 132.9, 129.5, 128.7, 84.0, 83.8, 55.1, 51.1, 24.9, 24.7; ^{11}B NMR (128 MHz, $CDCl_3$) δ 33.18, 22.39; HRMS (ESI+): Calculated for $C_{24}H_{36}B_2O_6SNa$: $[M+Na]^+$ 497.2317, Found 497.2320.

2-Phenyl-5-(1-phenyl-2-(3-tosylbicyclo[1.1.1]pentan-1-yl)ethyl)-1,3,4-oxadiazole (98)

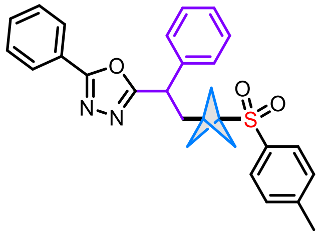

Obtained as a green liquid (76 mg, 81% yield); ^{1}H NMR (500 MHz, $CDCl_3$) δ 8.02 – 7.93 (m, 2H), 7.65 (d, J = 8.2 Hz, 2H), 7.53 – 7.44 (m, 3H), 7.35 – 7.24 (m, 7H), 4.23 (t, J = 7.8 Hz, 1H), 2.63 (dd, J = 14.6, 8.3 Hz, 1H), 2.41 (d, J = 9.9 Hz, 3H), 2.36 (dd, J = 14.7, 7.3 Hz, 1H), 1.82 (s, 6H); ^{13}C NMR (126 MHz, $CDCl_3$) δ 167.3, 165.0, 144.6, 138.0, 133.7, 131.8, 129.8, 129.1, 129.0, 128.6, 128.0, 127.7, 126.8, 123.7, 51.5, 50.9, 41.0, 38.0, 35.0, 21.7; HRMS (ESI+): Calculated for $C_{28}H_{27}N_2O_3S$: $[M+H]^+$ 471.1737, Found 471.1728.

1,1,1-Trifluoro-2-phenyl-3-(3-tosylbicyclo[1.1.1]pentan-1-yl)propan-2-ol (99)

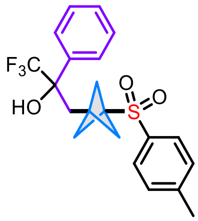

Obtained as a white solid (60 mg, 73% yield); M. P. = 176-177 °C; ^{1}H NMR (400 MHz, $CDCl_3$) δ 7.60 (d, J = 8.2 Hz, 2H), 7.45 (dd, J = 6.4, 2.6 Hz, 2H), 7.41 – 7.35 (m, 3H), 7.29 (d, J = 8.0 Hz, 2H), 2.71 (s, 1H), 2.49 (d, J = 15.1 Hz, 1H), 2.43 (s, 3H), 2.21 (d, J = 15.1 Hz, 1H), 1.75 (dd, J = 9.3, 1.8 Hz, 3H), 1.60 (dd, J = 9.3, 1.8 Hz, 3H); ^{13}C NMR (101 MHz, $CDCl_3$) δ 144.6, 135.1, 133.7, 129.7, 128.8, 128.5, 128.4, 126.3, 52.3, 52.1, 35.4, 35.2, 21.7; ^{19}F NMR (377 MHz, $CDCl_3$) δ -81.29; HRMS (ESI+): Calculated for $C_{21}H_{22}F_3O_3S$: $[M+H]^+$ 411.1236, Found 411.1233.

(3-Nitrophenyl)(3-tosylbicyclo[1.1.1]pentan-1-yl)methanol (100)

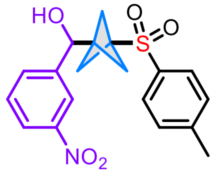

Obtained as a yellow liquid (67 mg, 90% yield); ^{1}H NMR (400 MHz, $CDCl_3$) δ 7.78 – 7.72 (m, 3H), 7.52 (d, J = 7.6 Hz, 1H), 7.37 (d, J = 8.0 Hz, 2H), 7.26 – 7.21 (m, 2H), 4.75 (s, 2H), 2.47 (s, 3H), 2.41 (s, 6H); ^{13}C NMR (101 MHz, $CDCl_3$) δ 145.0, 142.4, 134.0, 133.5, 130.4, 130.3, 130.2, 130.0, 128.7, 122.2, 63.5, 52.5, 51.5, 39.5, 21.7; HRMS (ESI+): Calculated for $C_{19}H_{20}NO_5S$: $[M+H]^+$ 374.1057, Found 374.1053.

3-Nitrophenyl)(3-tosylbicyclo[1.1.1]pentan-1-yl)methanone O-(5-bromopentanoyl) oxime (101)

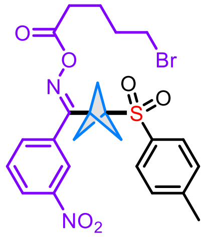

Obtained as a yellow liquid (59 mg, 54% yield); ^{1}H NMR (400 MHz, $CDCl_3$) δ 8.36 (s, 1H), 8.06 (s, 1H), 7.91 (d, J = 7.9 Hz, 1H), 7.75 (d, J = 8.0 Hz, 2H), 7.37 (d, J = 7.9 Hz, 2H), 7.32 (d, J = 8.0 Hz, 1H), 3.43 (t, J = 6.3 Hz, 2H), 2.54 – 2.50 (m, 2H), 2.47 (s, 3H), 2.44 (s, 6H), 2.00 – 1.93 (m, 2H), 1.89 (d, J = 7.4 Hz, 2H); ^{13}C NMR (126 MHz, $CDCl_3$) δ 170.2, 153.2, 149.6, 145.1, 134.2, 133.5, 131.4, 131.2, 130.9, 130.0, 128.7, 123.7, 52.6, 51.5, 39.5, 32.8, 31.8, 29.7, 23.3, 21.7; HRMS (ESI+): Calculated for $C_{24}H_{26}BrN_2O_6S$: $[M+H]^+$ 549.0689, Found 549.0684.

1-(Phenylsulfinyl)-3-tosylbicyclo[1.1.1]pentane (102)

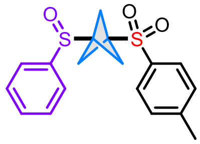

Obtained as a yellow solid (62 mg, 89% yield); M. P. = 151-152 °C; ^{1}H NMR (500 MHz, $CDCl_3$) δ 7.60 (d, J = 7.9 Hz, 2H), 7.43 (dd, J = 21.5, 2.1 Hz, 5H), 7.27 (d, J = 7.8 Hz, 2H), 2.37 (s, 3H), 2.00 (s, 6H); ^{13}C NMR (126 MHz, $CDCl_3$) δ 145.4, 140.5, 133.0, 131.6, 130.0, 129.4, 128.6, 123.9, 52.0, 51.5, 49.4, 21.7; HRMS (ESI+): Calculated for $C_{18}H_{19}O_3S_2$: $[M+H]^+$ 347.0770, Found 347.0778.

1-(Phenylsulfonyl)-3-tosylbicyclo[1.1.1]pentane (103)

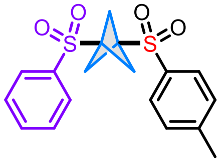

Obtained as a white solid (67 mg, 93% yield); M. P. = 184-185 °C; ^{1}H NMR (500 MHz, $CDCl_3$) δ 7.81 (d, J = 7.3 Hz, 2H), 7.69 (dd, J = 12.7, 7.8 Hz, 3H), 7.58 (t, J = 7.8 Hz, 2H), 7.36 (d, J = 8.0 Hz, 2H), 2.46 (s, 3H), 2.25 (s, 6H); ^{13}C NMR (126 MHz, $CDCl_3$) δ 145.6, 135.9, 134.4, 132.8, 130.2, 129.5, 128.6, 128.6, 51.0, 50.8, 50.8, 21.7; HRMS (ESI+): Calculated for $C_{18}H_{18}O_4S_2Na$: $[M+Na]^+$ 385.0539, Found 385.0540.

2-(3-Tosylbicyclo[1.1.1]pentan-1-yl)isoindolin-1-one (104)

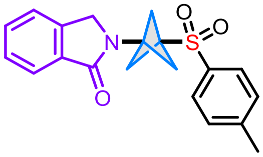

Obtained as a yellow solid (35 mg, 50% yield); M. P. = 175-176 °C; ^{1}H NMR (400 MHz, $CDCl_3$) δ 7.77 (d, J = 8.1 Hz, 3H), 7.54 (t, J = 7.5 Hz, 1H), 7.43 (dd, J = 16.3, 7.7 Hz, 2H), 7.38 (d, J = 8.1 Hz, 2H), 4.33 (s, 2H), 2.53 (s, 6H), 2.47 (s, 3H); ^{13}C NMR (101 MHz, $CDCl_3$) δ 168.8, 145.1, 140.6, 133.8, 132.5 132.0, 130.0, 128.6, 128.4, 123.8, 122.9, 53.3, 50.1, 48.4, 47.7, 21.7; HRMS (ESI+): Calculated for $C_{20}H_{20}NO_3S$: $[M+H]^+$ 354.1158, Found 354.1156.

Tert-butyl ((3-tosylbicyclo[1.1.1]pentan-1-yl)methyl)carbamate (105)

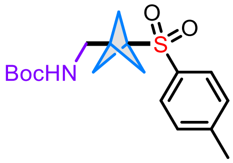

Obtained as a yellow liquid (48 mg, 68% yield); ^{1}H NMR (400 MHz, $CDCl_3$) δ 7.71 (d, J = 8.2 Hz, 2H), 7.34 (d, J = 8.0 Hz, 2H), 4.54 (s, 1H), 3.21 (d, J = 6.1 Hz, 2H), 2.45 (s, 3H), 1.93 (s, 6H), 1.40 (s, 9H); ^{13}C NMR (101 MHz, $CDCl_3$) δ 155.8, 144.7, 133.7, 129.8, 128.7, 79.7, 51.7, 49.7, 41.0, 38.4, 28.3, 21.7; HRMS (ESI+): Calculated for $C_{18}H_{25}NO_4SNa$: $[M+Na]^+$ 374.1397, Found 374.1394.

3-Tosylbicyclo[1.1.1]pentane-1-carboxylic acid (106)

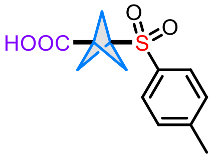

Obtained as a white solid (46 mg, 86% yield); M. P. = 174-175 °C; ^{1}H NMR (400 MHz, $CDCl_3$) δ 8.45 (s, 1H), 7.70 (d, J = 8.2 Hz, 2H), 7.35 (d, J = 8.0 Hz, 2H), 2.44 (s, 3H), 2.28 (s, 6H); ^{13}C NMR (101 MHz, $CDCl_3$) δ 173.3, 145.3, 133.0, 130.0, 128.6, 51.8, 51.4, 36.6, 21.7.

3-Tosylbicyclo[1.1.1]pentane-1-carboxamide (107)

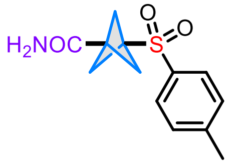

Obtained as a white solid (45 mg, 85% yield); M. P. = 193-194 °C; ^{1}H NMR (500 MHz, $CDCl_3$) δ 7.73 (d, J = 8.1 Hz, 2H), 7.37 (d, J = 7.9 Hz, 2H), 5.47 (s, 2H), 2.46 (s, 3H), 2.28 (s, 6H); ^{13}C NMR (151 MHz, $CDCl_3$) δ 169.5, 145.2, 133.2, 130.0, 128.7, 51.4, 50.8, 37.8, 21.7.

Methyl-3-tosylbicyclo[1.1.1]pentane-1-carboxylate (108)

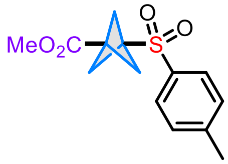

Obtained as a yellow solid (51 mg, 91% yield); M. P. = 134-135 °C; ^{1}H NMR (400 MHz, $CDCl_3$) δ 7.71 (d, J = 8.2 Hz, 2H), 7.35 (d, J = 8.1 Hz, 2H), 3.65 (s, 3H), 2.44 (s, 3H), 2.27 (s, 6H); ^{13}C NMR (101 MHz, $CDCl_3$) δ 168.4, 145.1, 133.3, 130.0, 128.6, 52.2, 51.8, 51.4, 36.7, 21.7; HRMS (ESI+): Calculated for $C_{14}H_{17}O_4S$: $[M+H]^+$ 281.0842, Found 281.0833.

5. Copies of NMR spectra

4 ^{1}H NMR (500 MHz, $CDCl_3$)

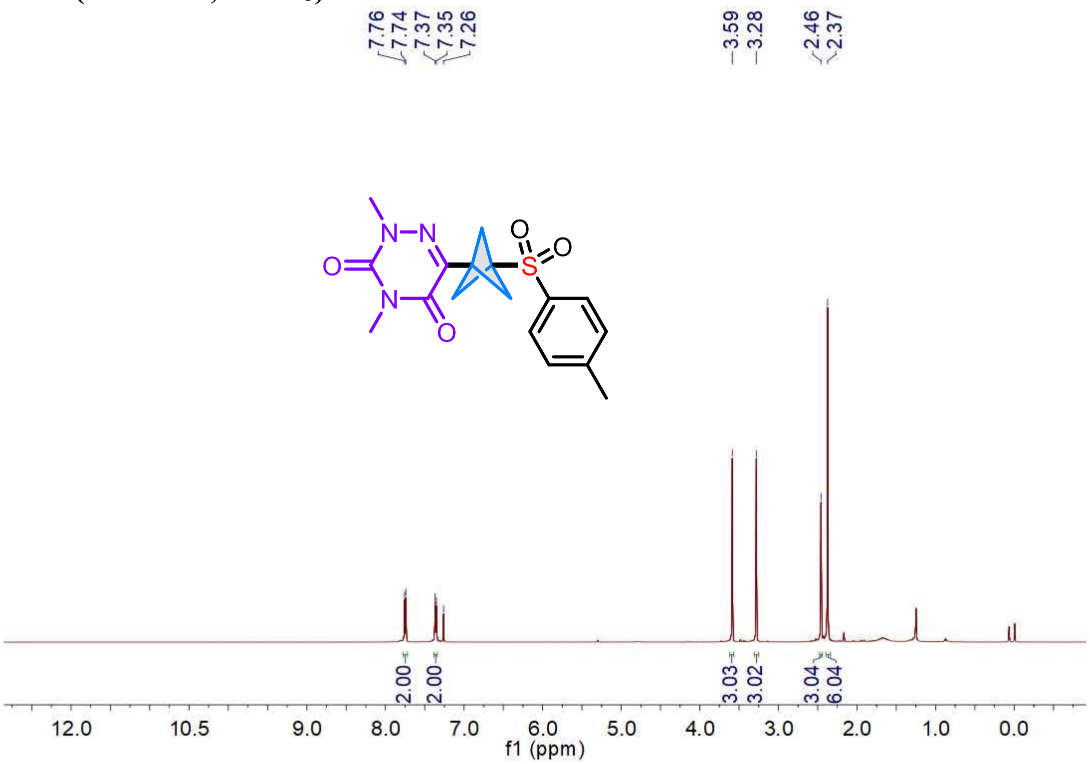

4 ^{13}C NMR (126 MHz, $CDCl_3$)

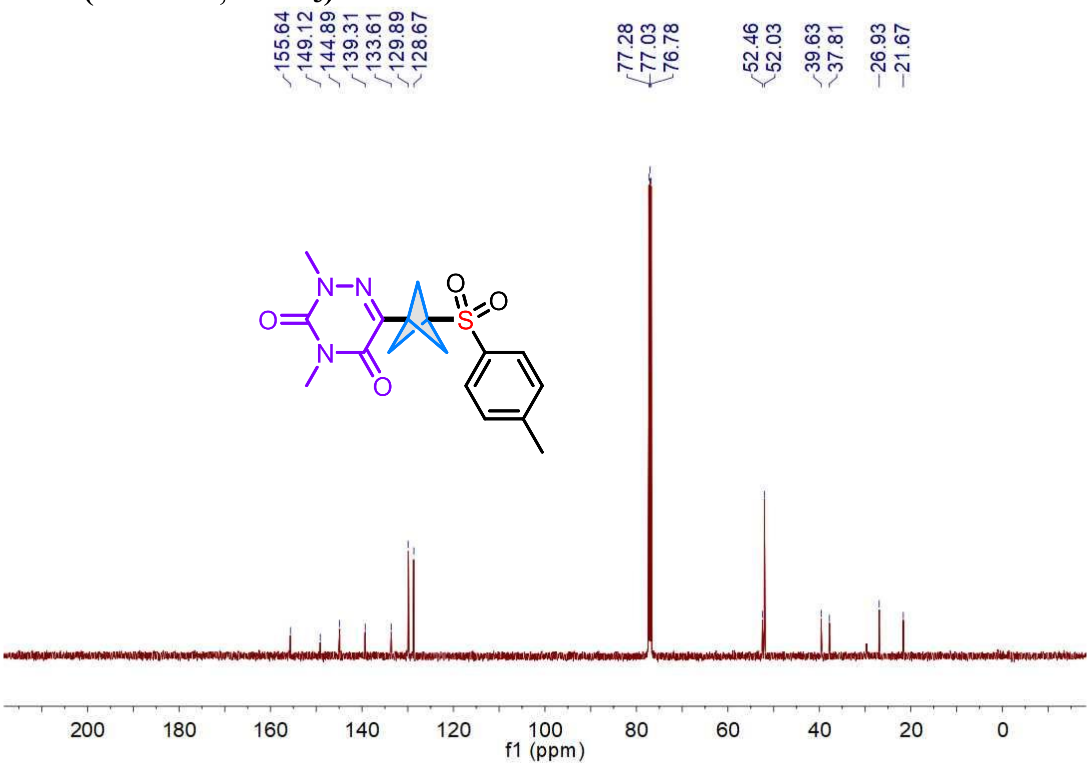

5 ^{1}H NMR (500 MHz, $CDCl_3$)

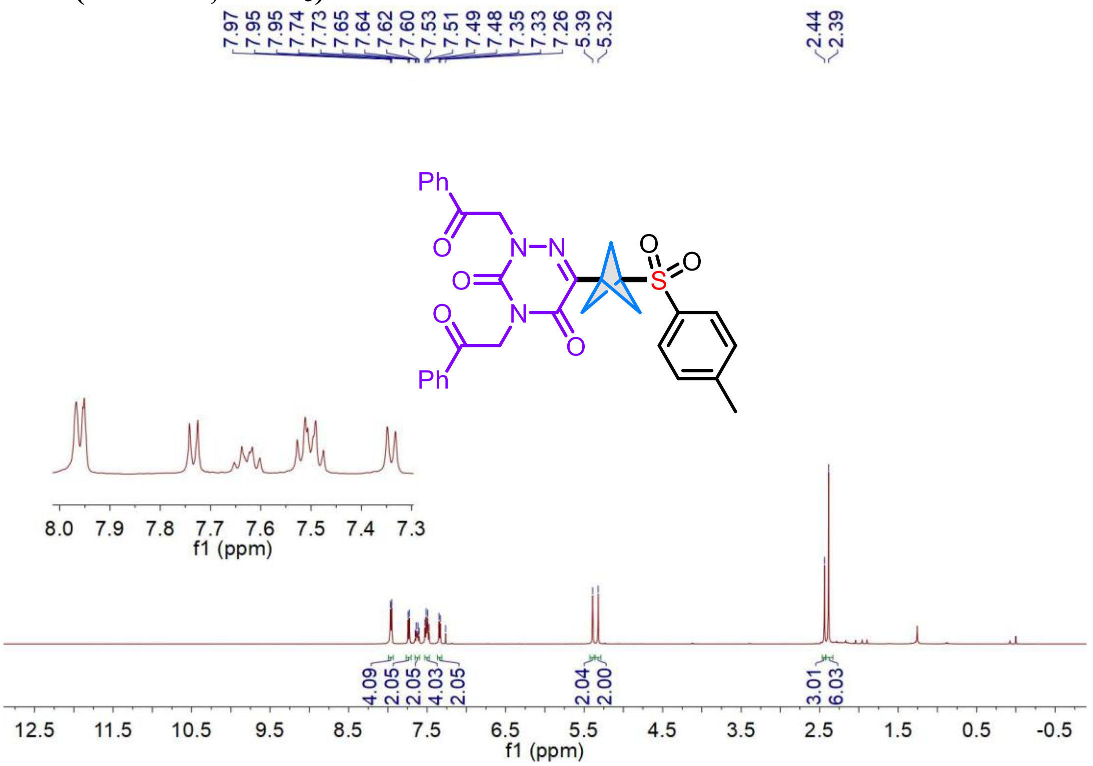

5 ^{13}C NMR (126 MHz, $CDCl_3$)

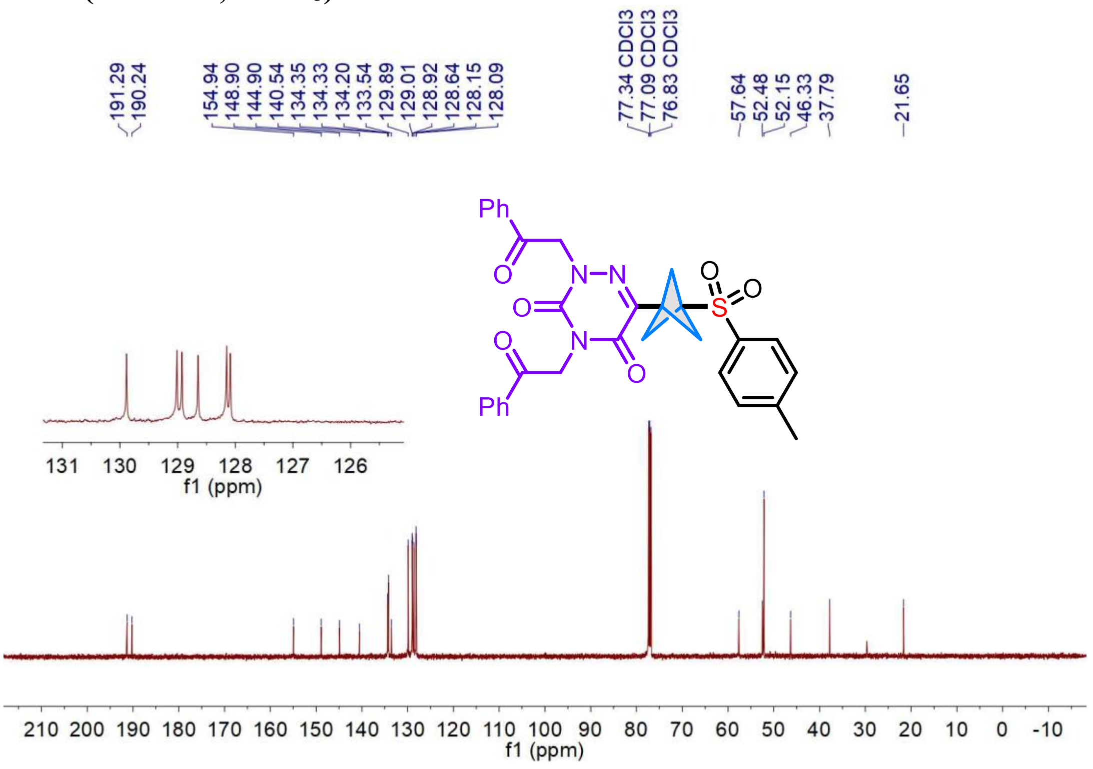

6 ¹H NMR (500 MHz, CDCl₃)

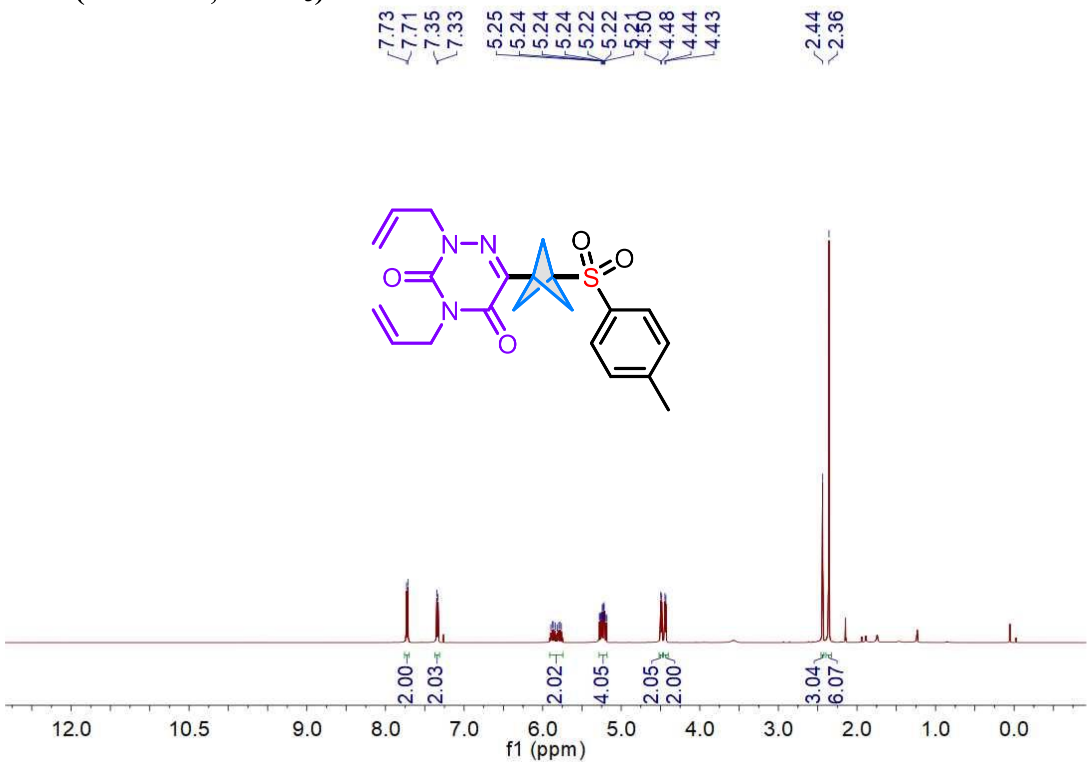

6 ¹³C NMR (126 MHz, CDCl₃)

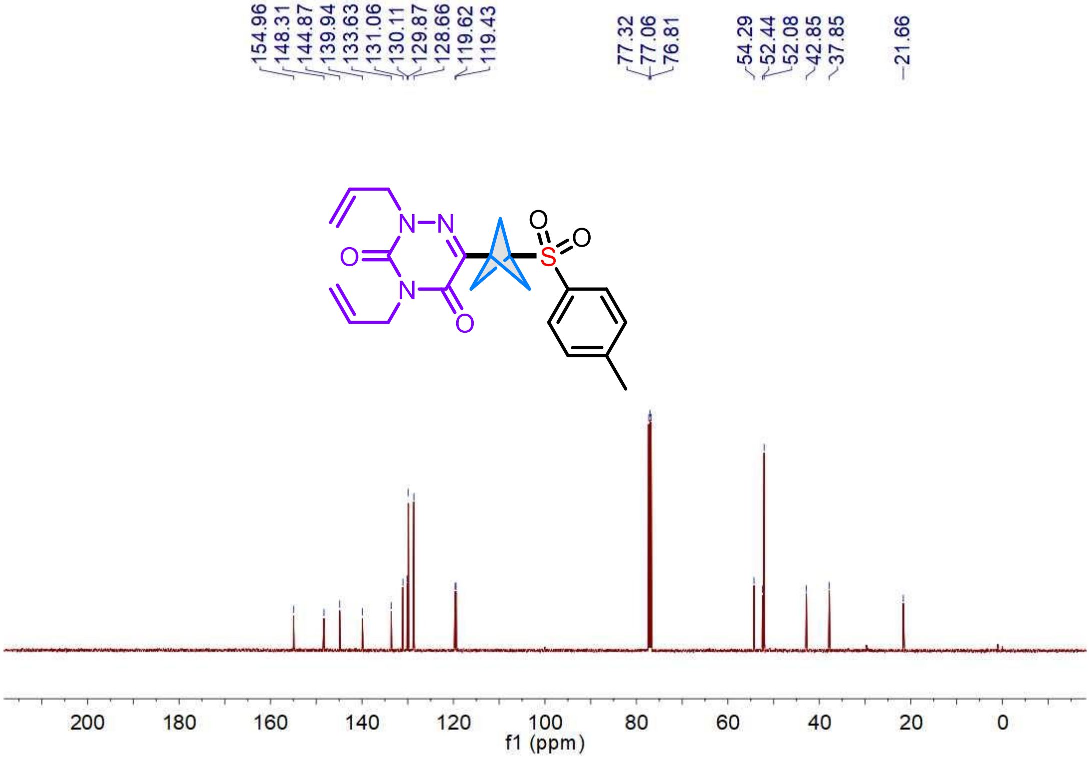

7 ^{1}H NMR (500 MHz, $CDCl_3$)

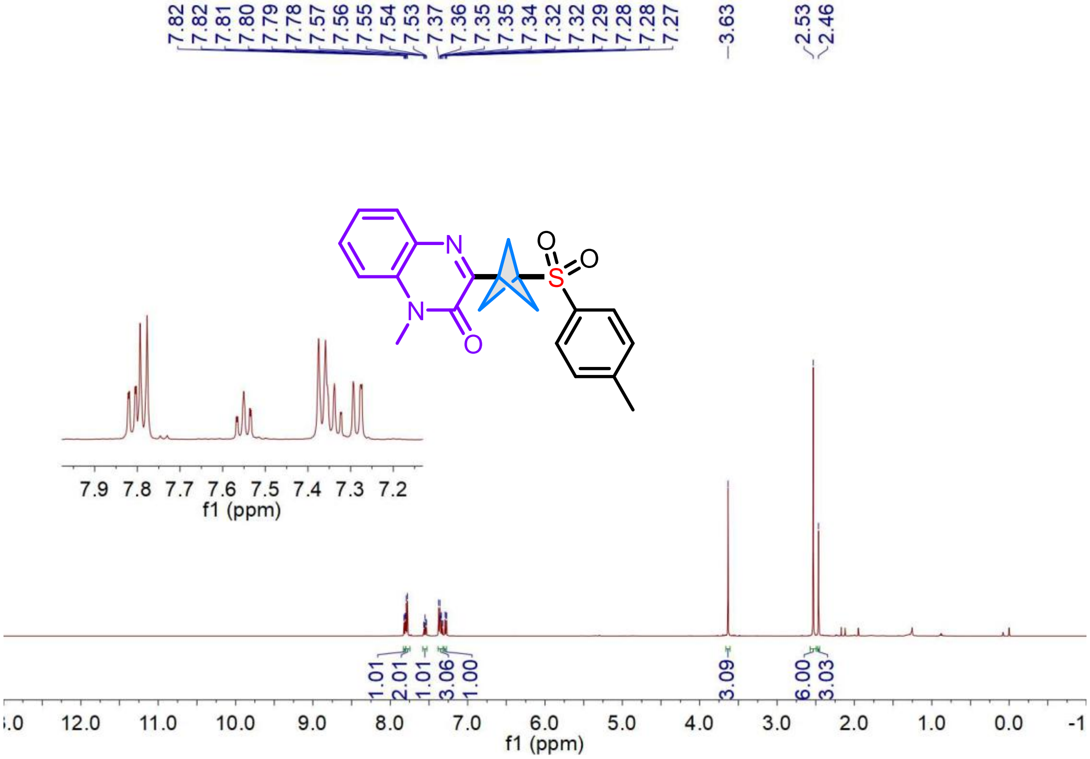

7 ^{13}C NMR (126 MHz, $CDCl_3$)

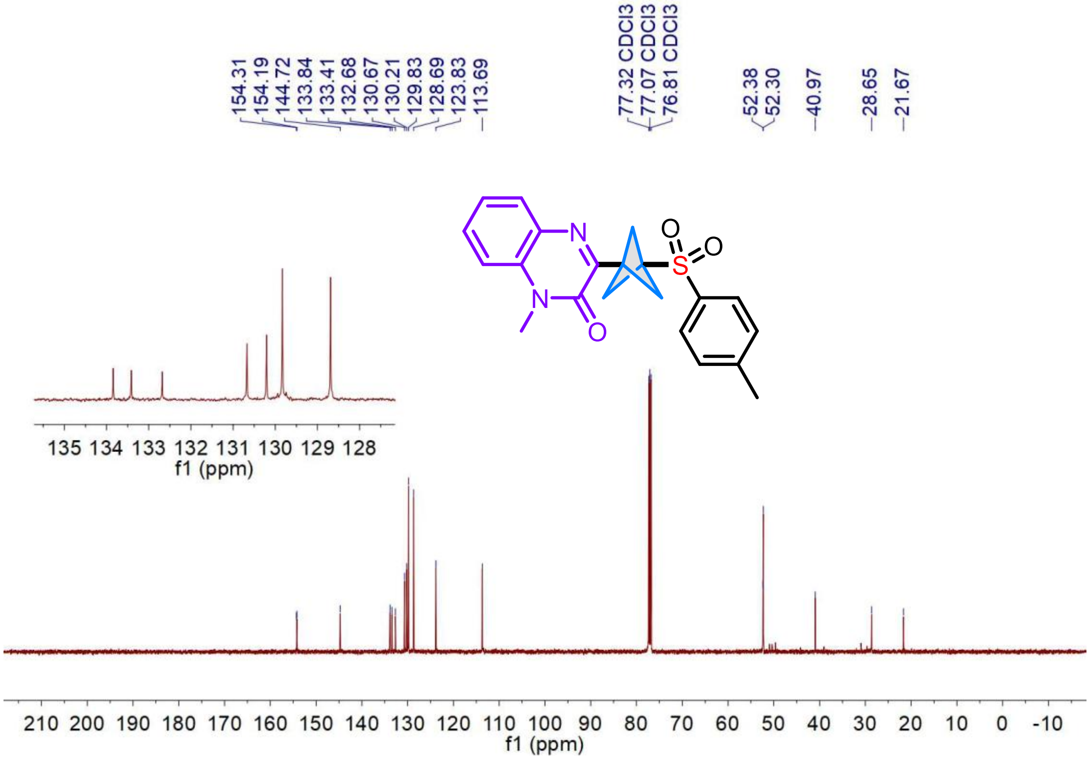

8 ^{1}H NMR (500 MHz, $CDCl_3$)

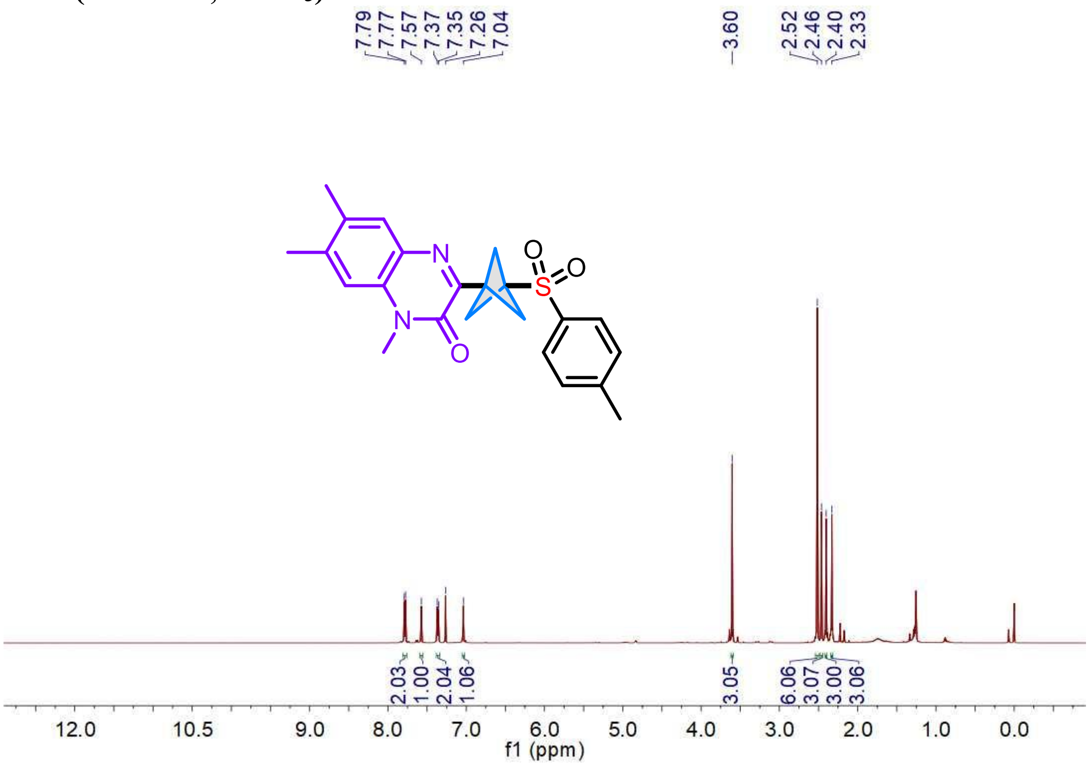

8 ^{13}C NMR (126 MHz, $CDCl_3$)

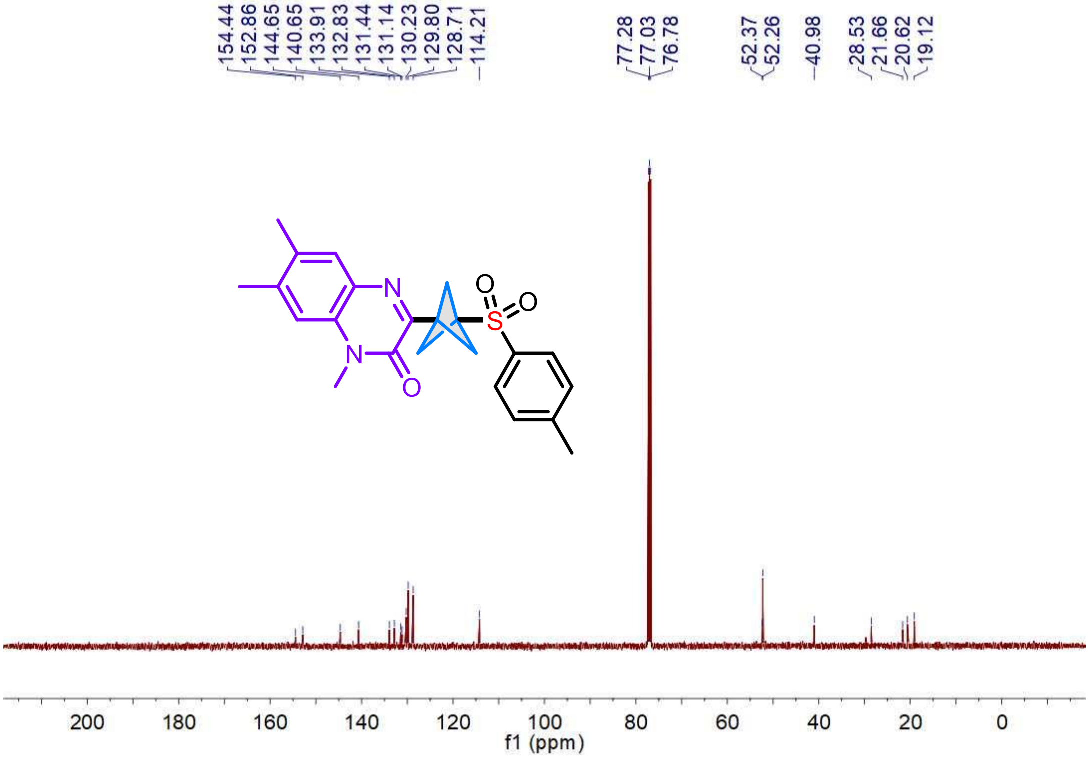

9 ^{1}H NMR (500 MHz, CDCl$_3$)

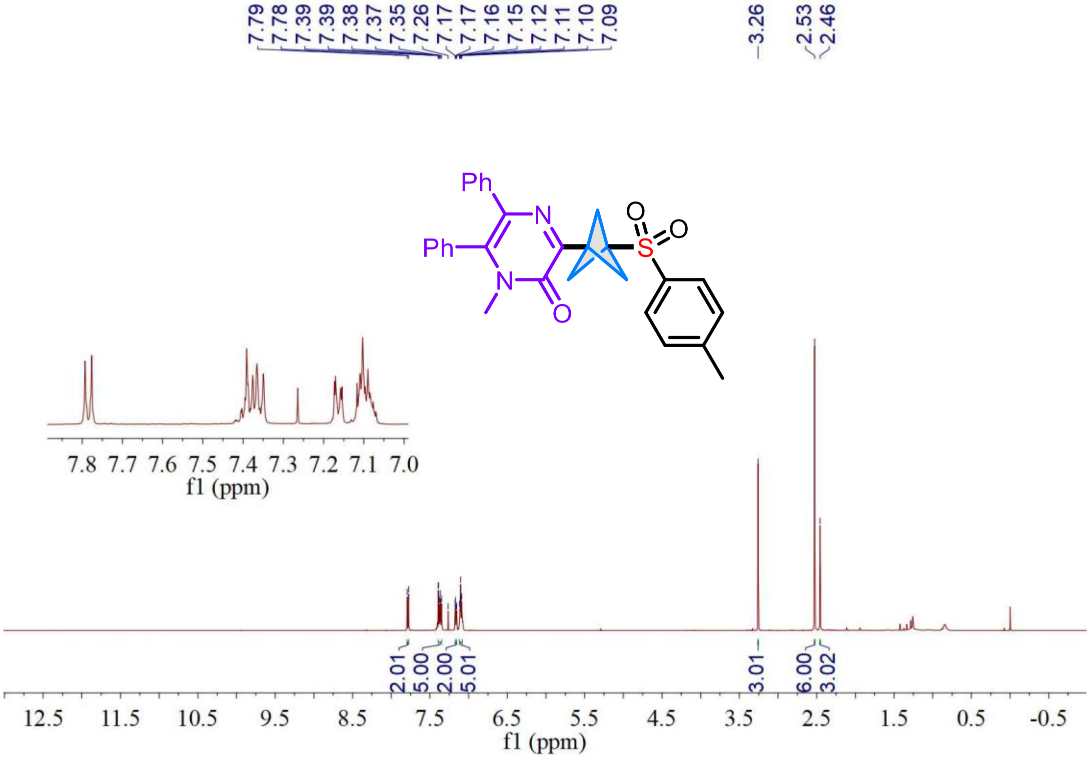

9 ^{13}C NMR (126 MHz, CDCl$_3$)

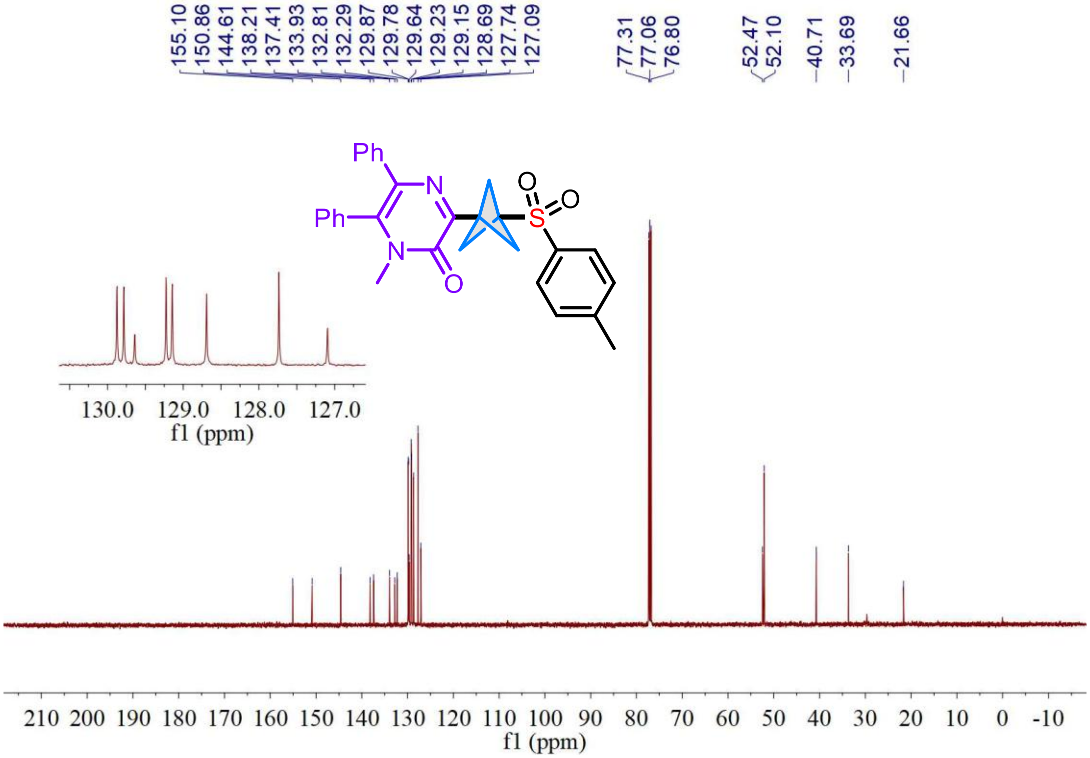

10 ^{1}H NMR (500 MHz, $CDCl_3$)

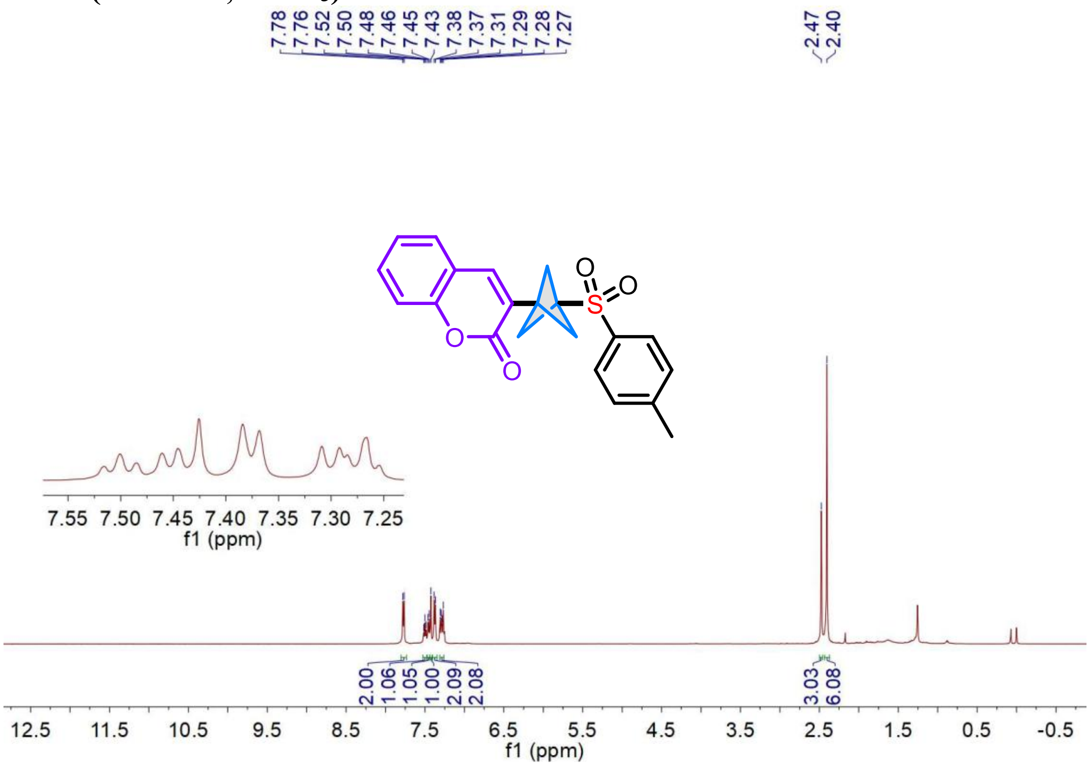

10 ^{13}C NMR (126 MHz, $CDCl_3$)

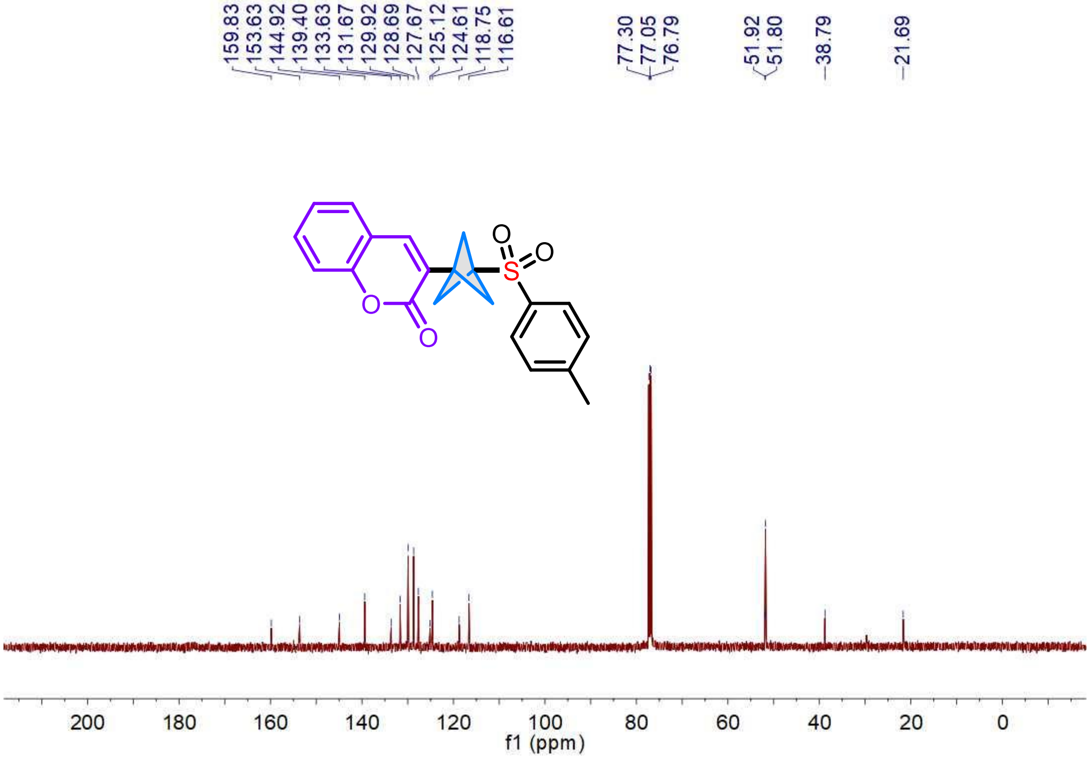

11 ^{1}H NMR (500 MHz, $CDCl_3$)

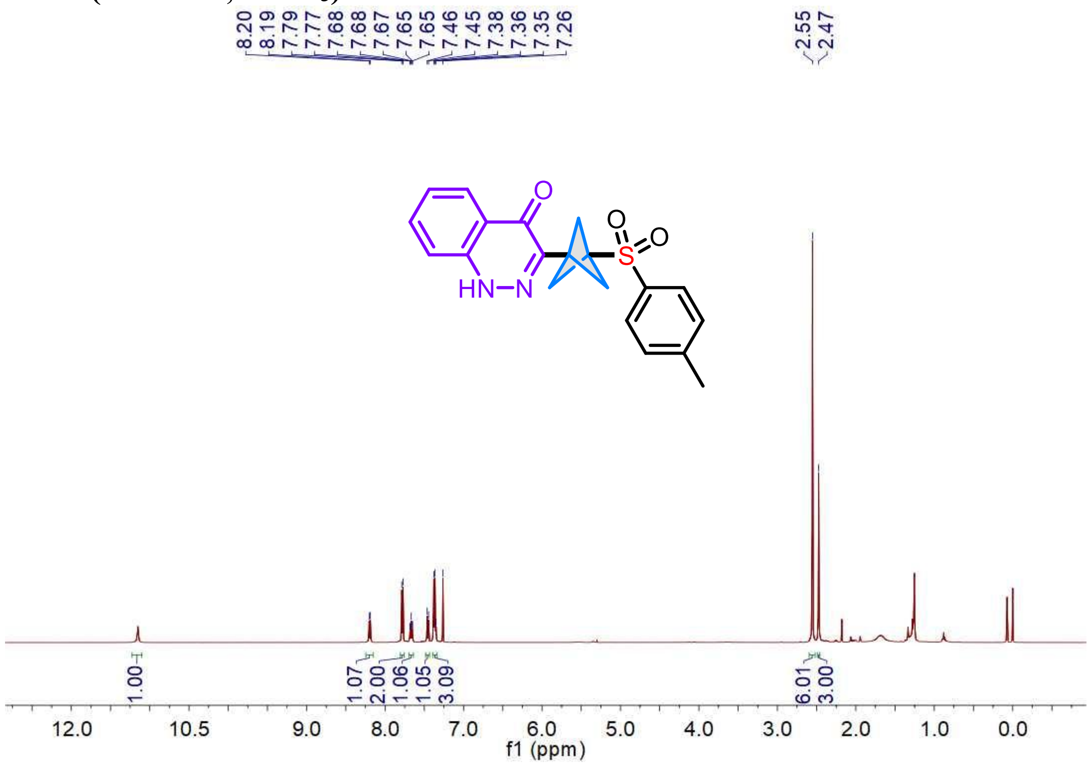

11 ^{13}C NMR (126 MHz, $CDCl_3$)

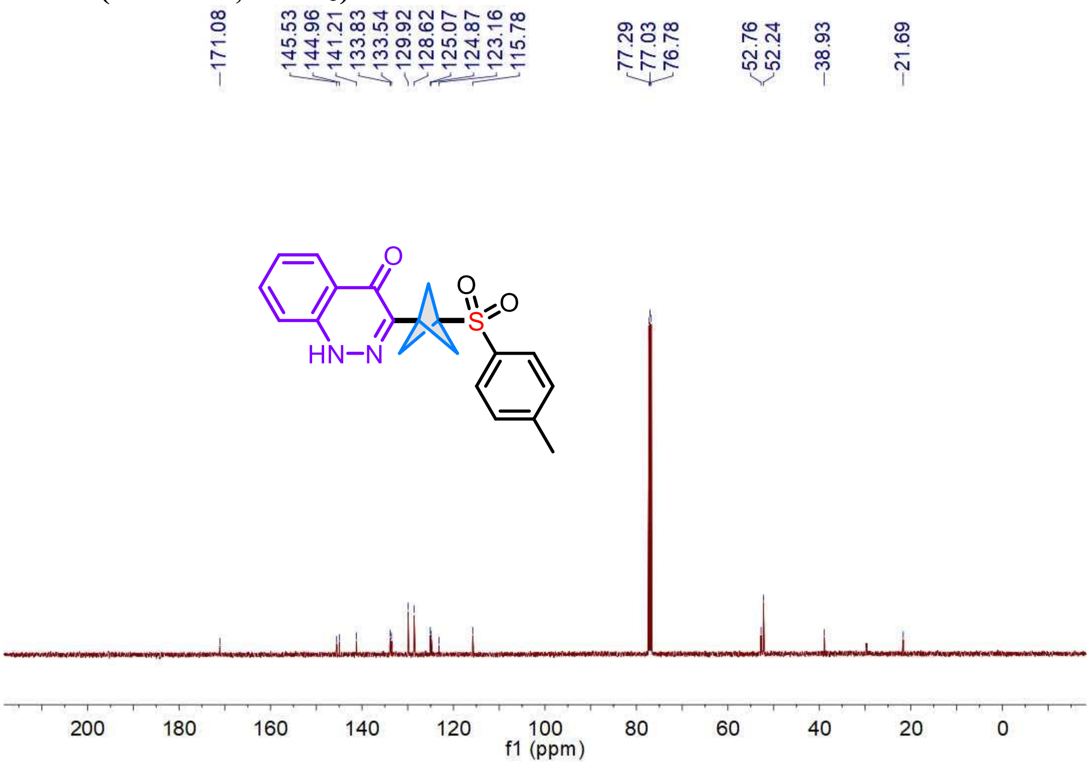

12 ^{1}H NMR (500 MHz, $CDCl_3$)

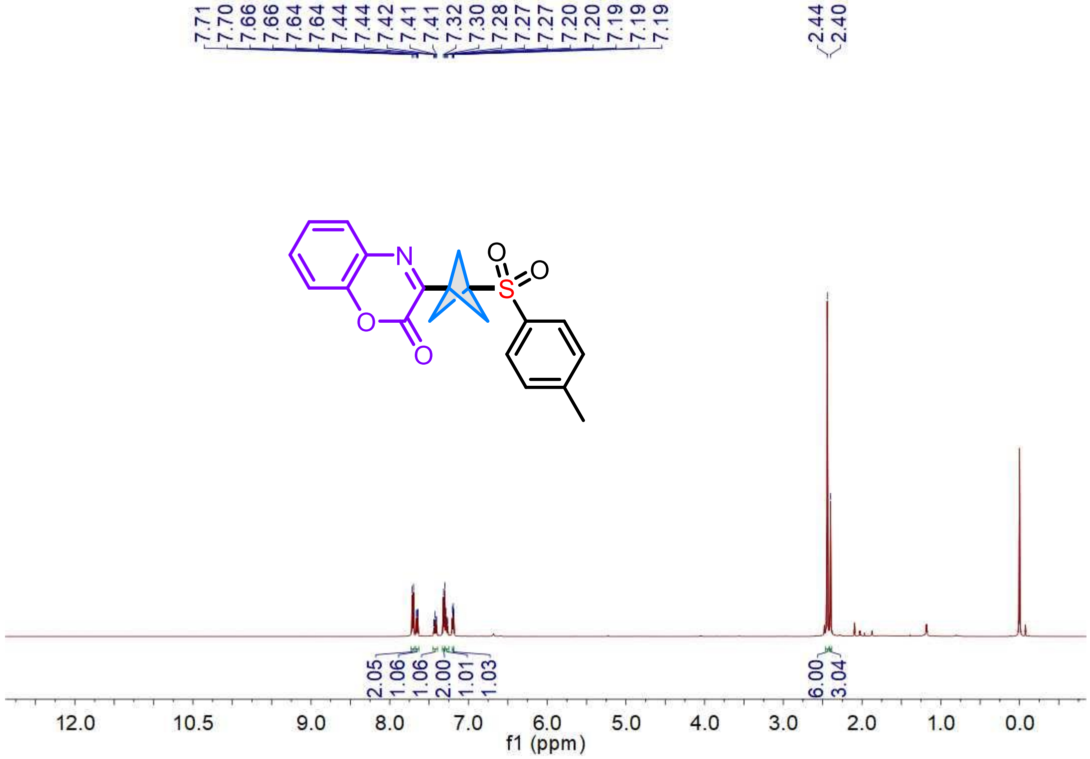

12 ^{13}C NMR (126 MHz, $CDCl_3$)

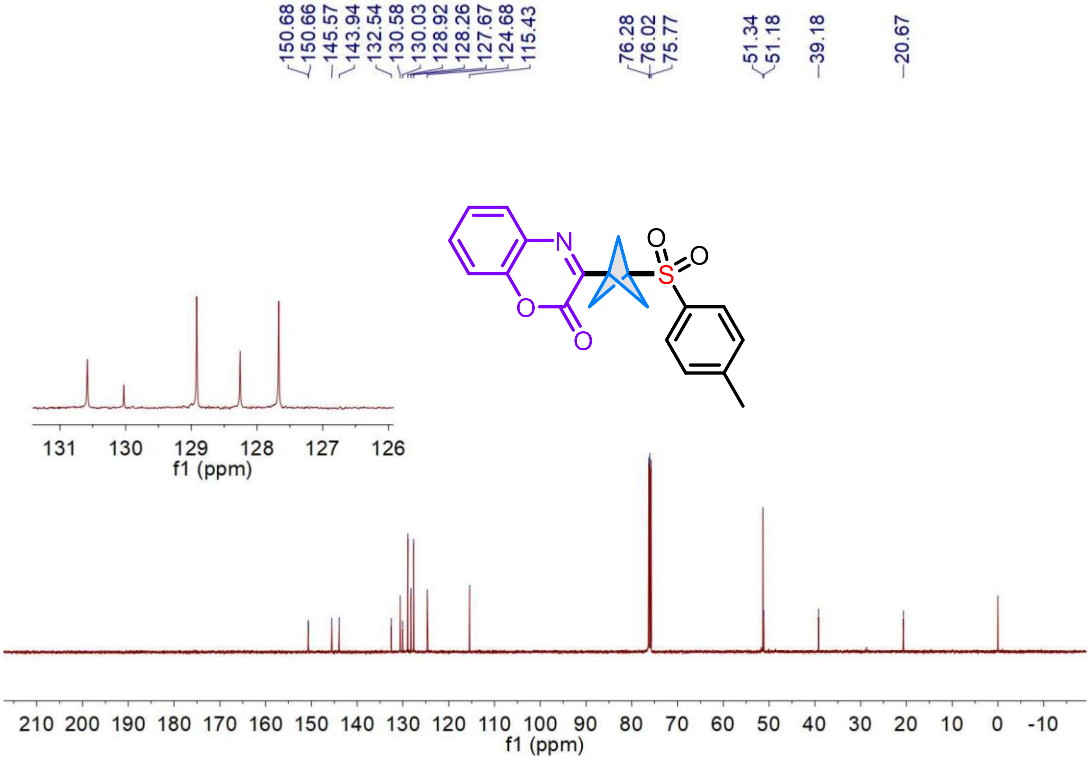

13 ^{1}H NMR (500 MHz, $CDCl_3$)

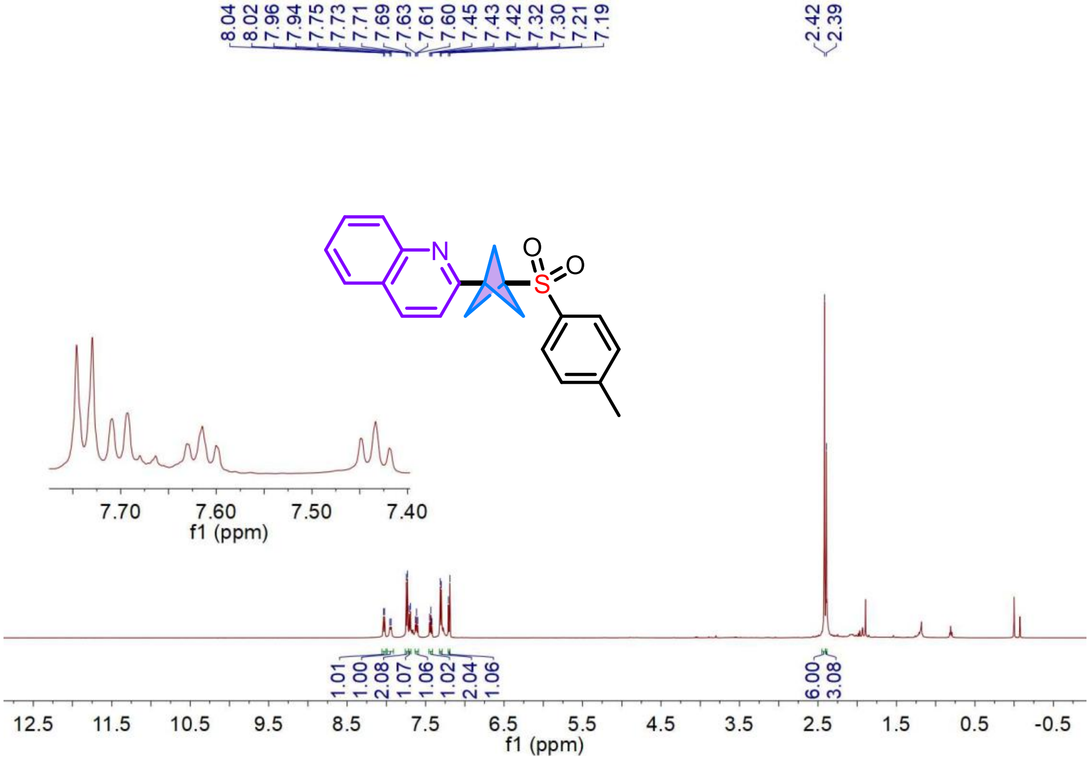

13 ^{13}C NMR (126 MHz, $CDCl_3$)

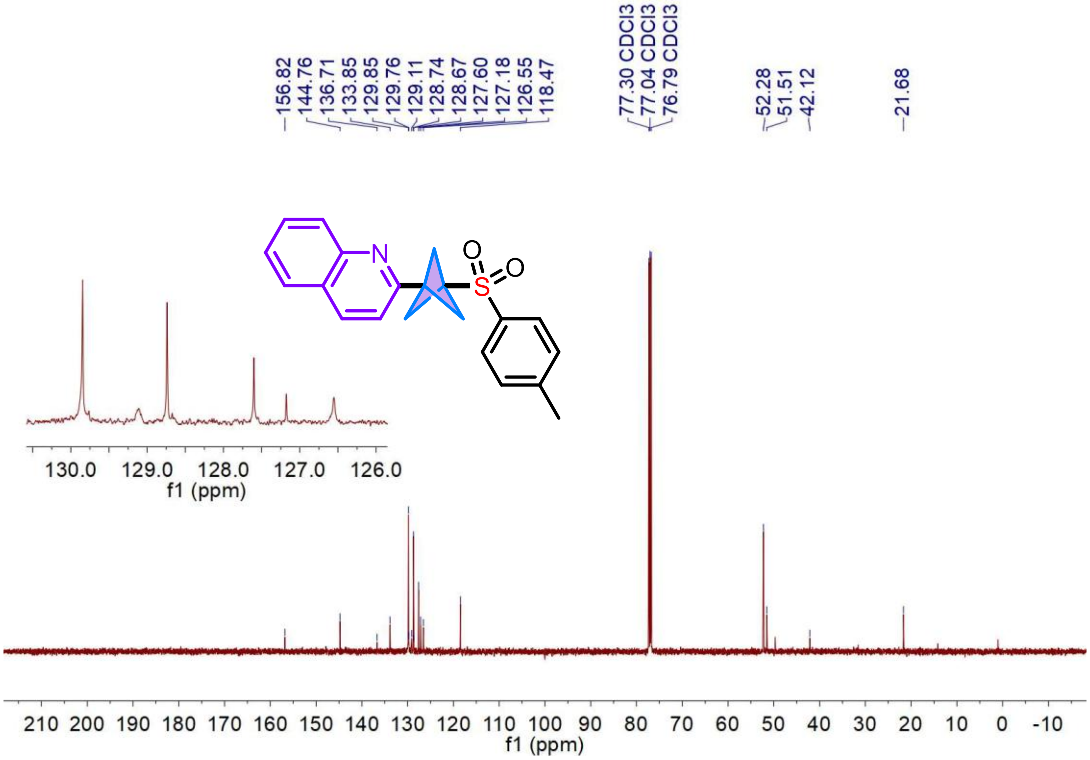

14 ^{1}H NMR (500 MHz, $CDCl_3$)

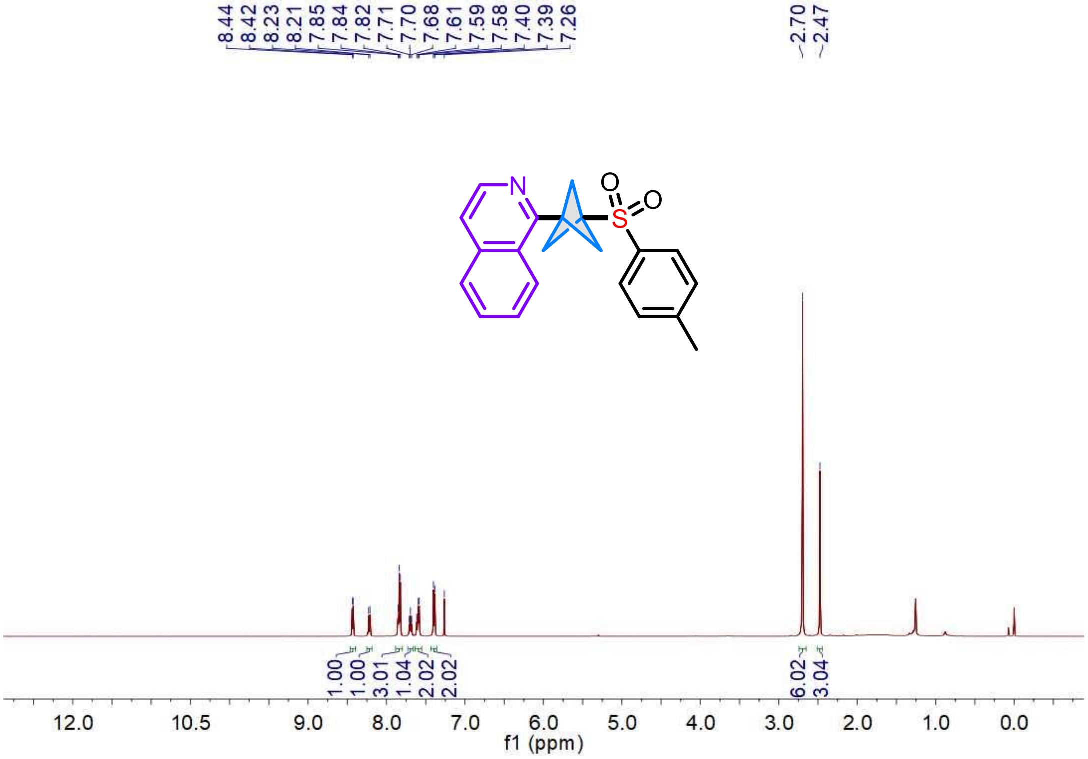

14 ^{13}C NMR (126 MHz, $CDCl_3$)

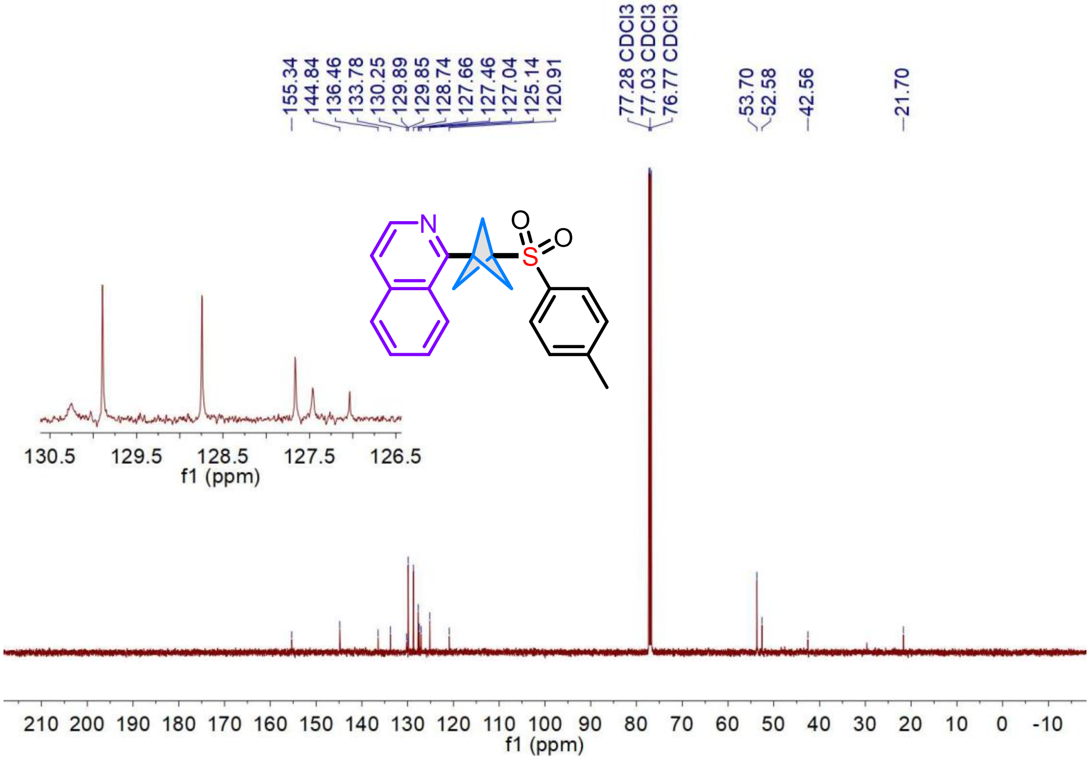

15 ^{1}H NMR (500 MHz, $CDCl_3$)

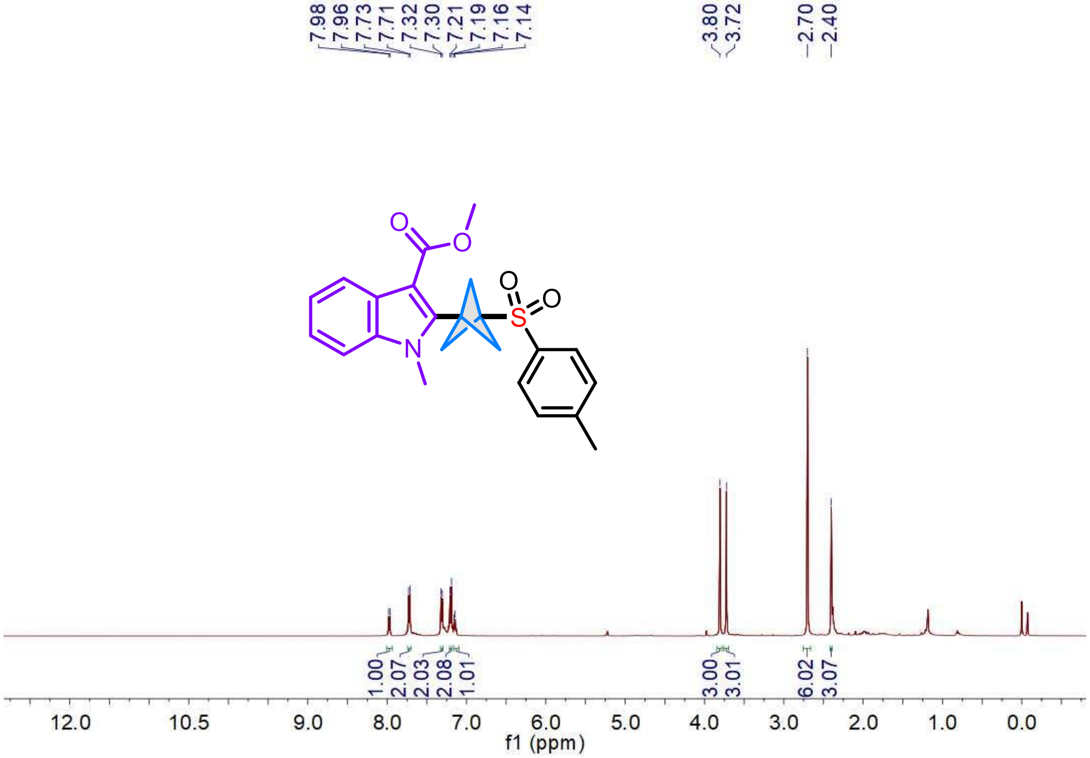

15 ^{13}C NMR (126 MHz, $CDCl_3$)

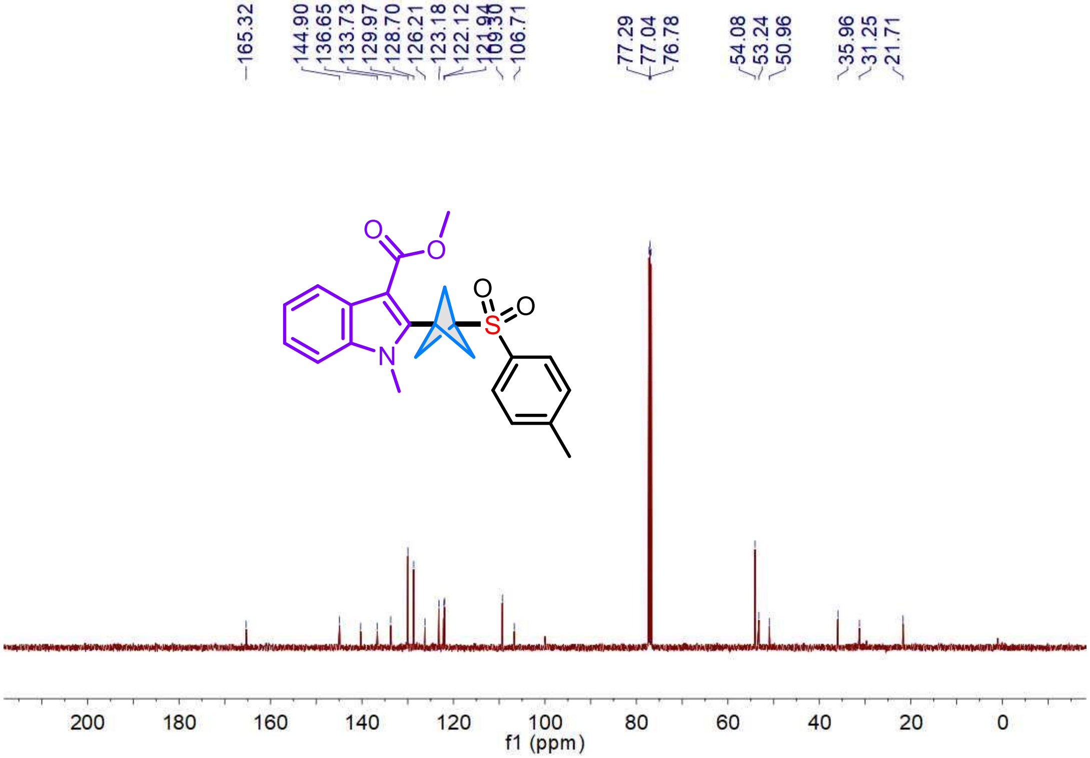

16 ¹H NMR (500 MHz, $CDCl_3$)

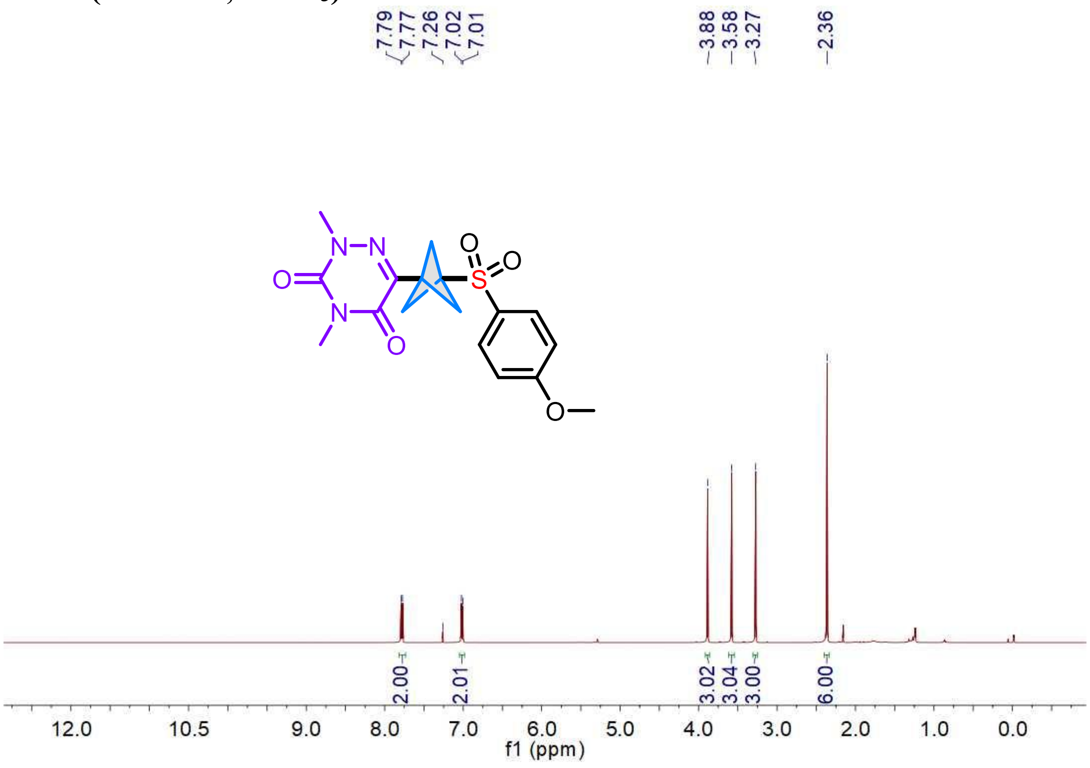

16 ¹³C NMR (126 MHz, $CDCl_3$)

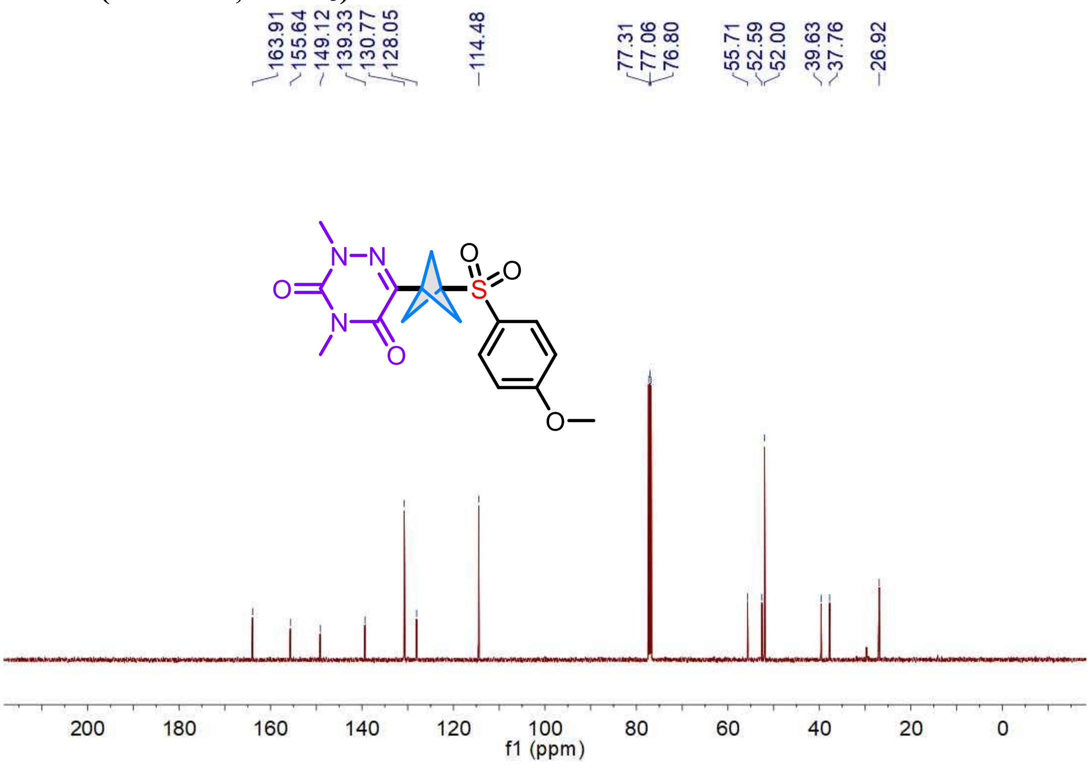

17 ^{1}H NMR (500 MHz, $CDCl_3$)

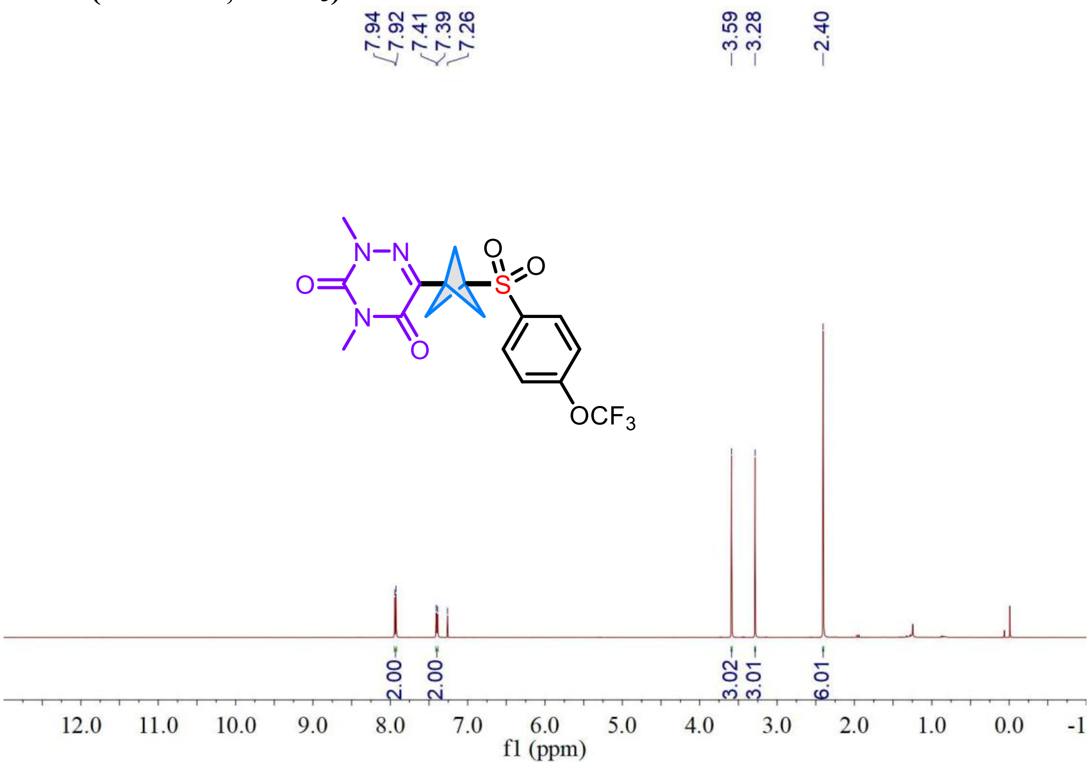

17 ^{13}C NMR (126 MHz, $CDCl_3$)

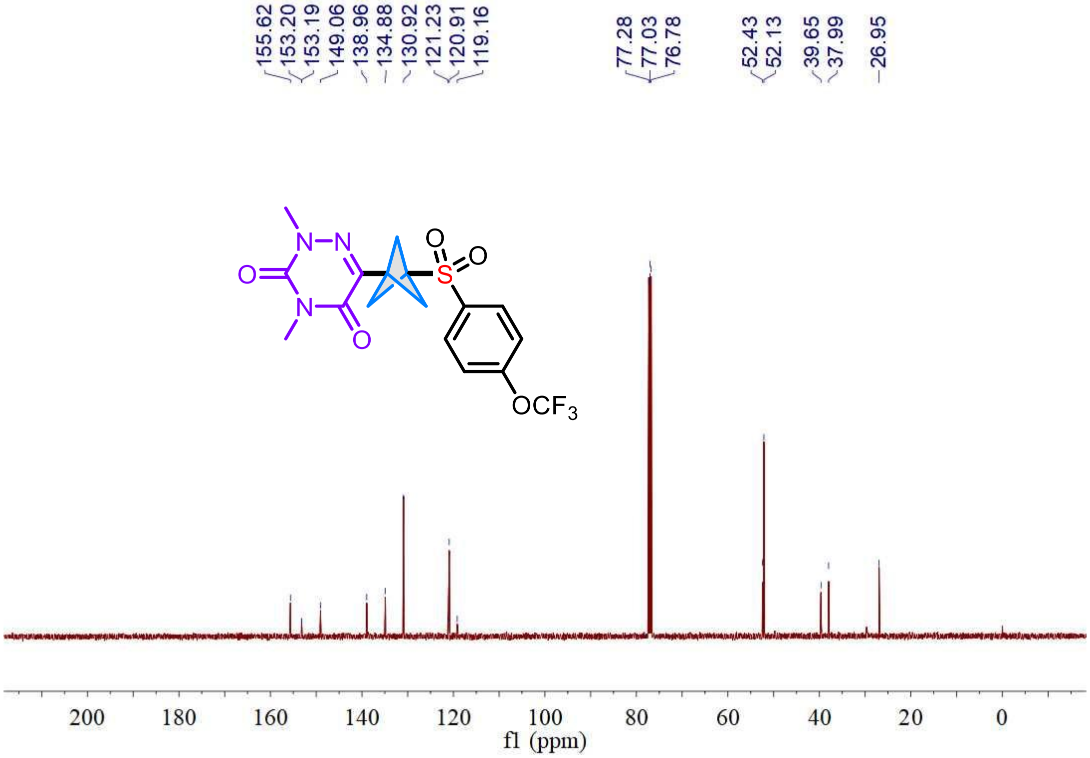

17 ^{19}F NMR (471 MHz, $CDCl_3$)

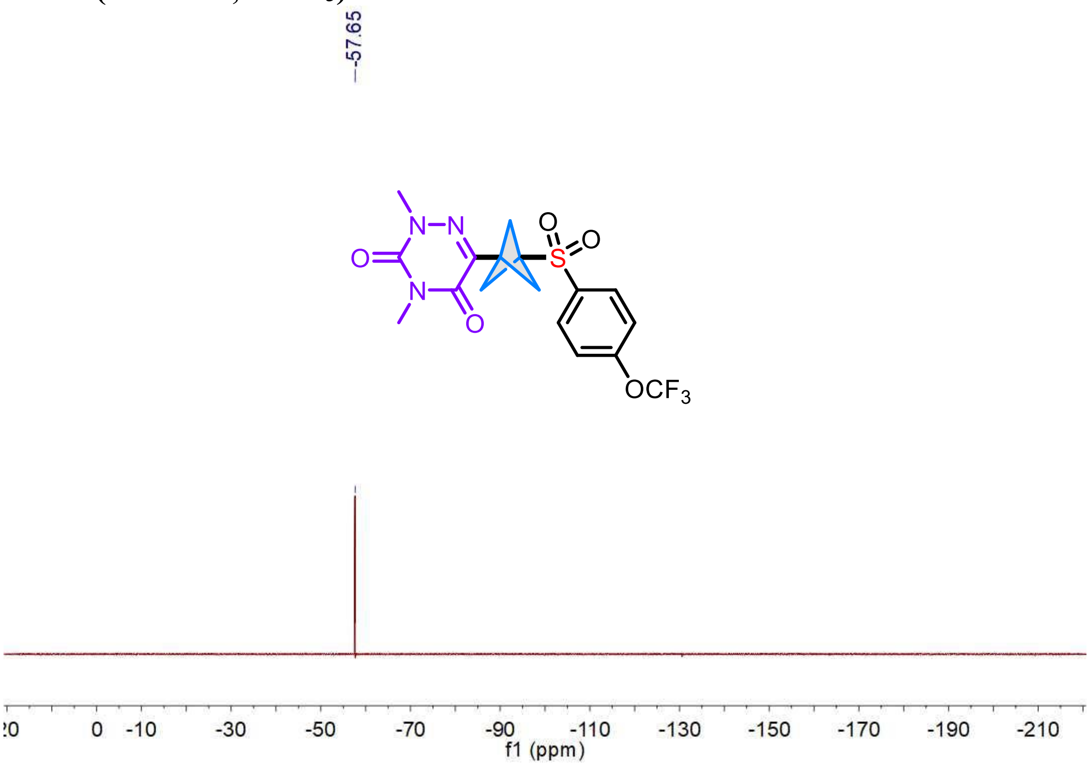

18 ^{1}H NMR (500 MHz, $CDCl_3$)

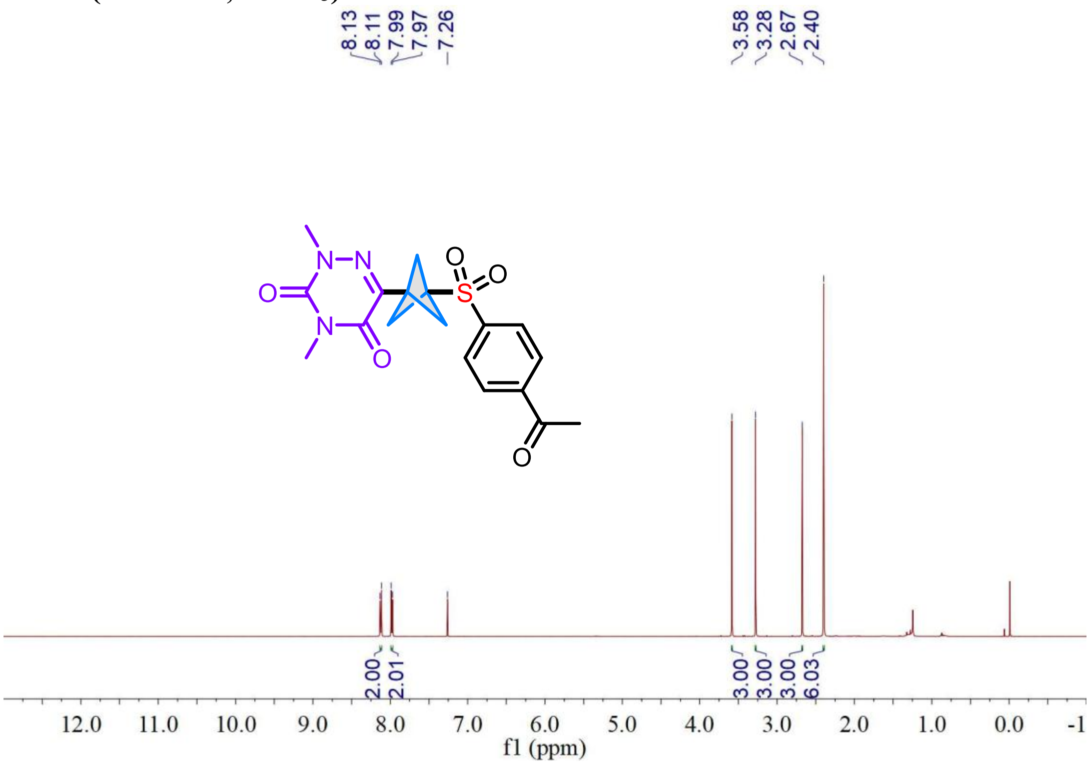

18 ^{13}C NMR (126 MHz, CDCl$_3$)

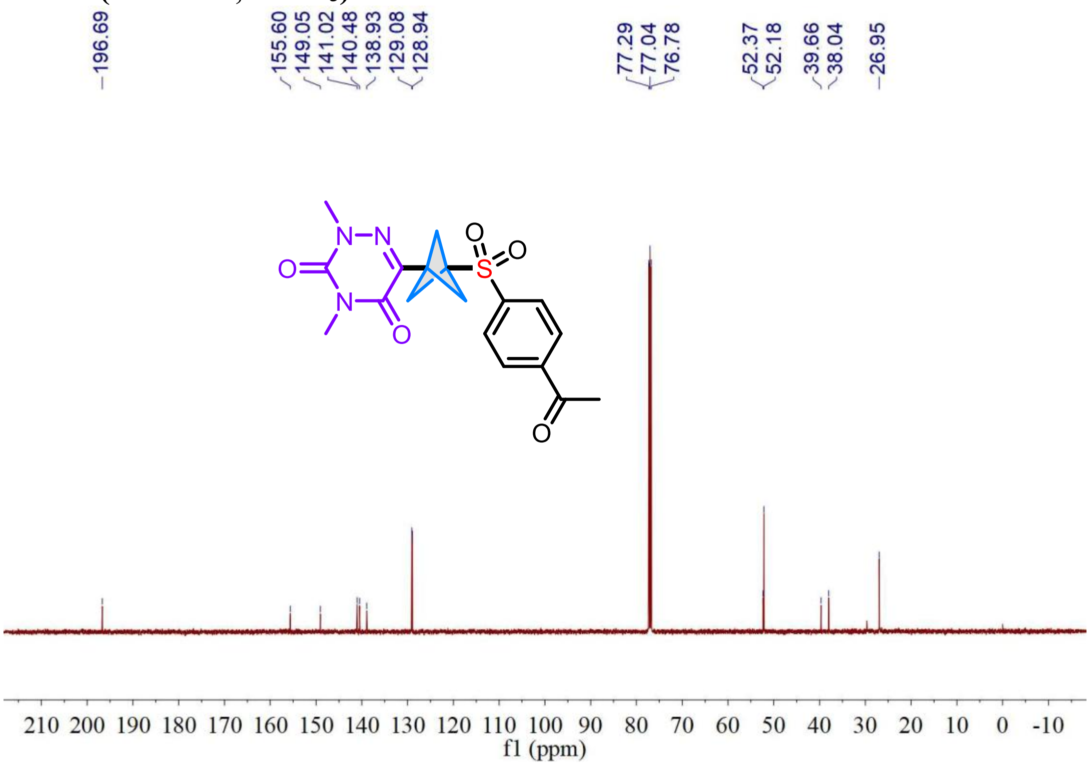

19 ^{1}H NMR (500 MHz, CDCl$_3$)

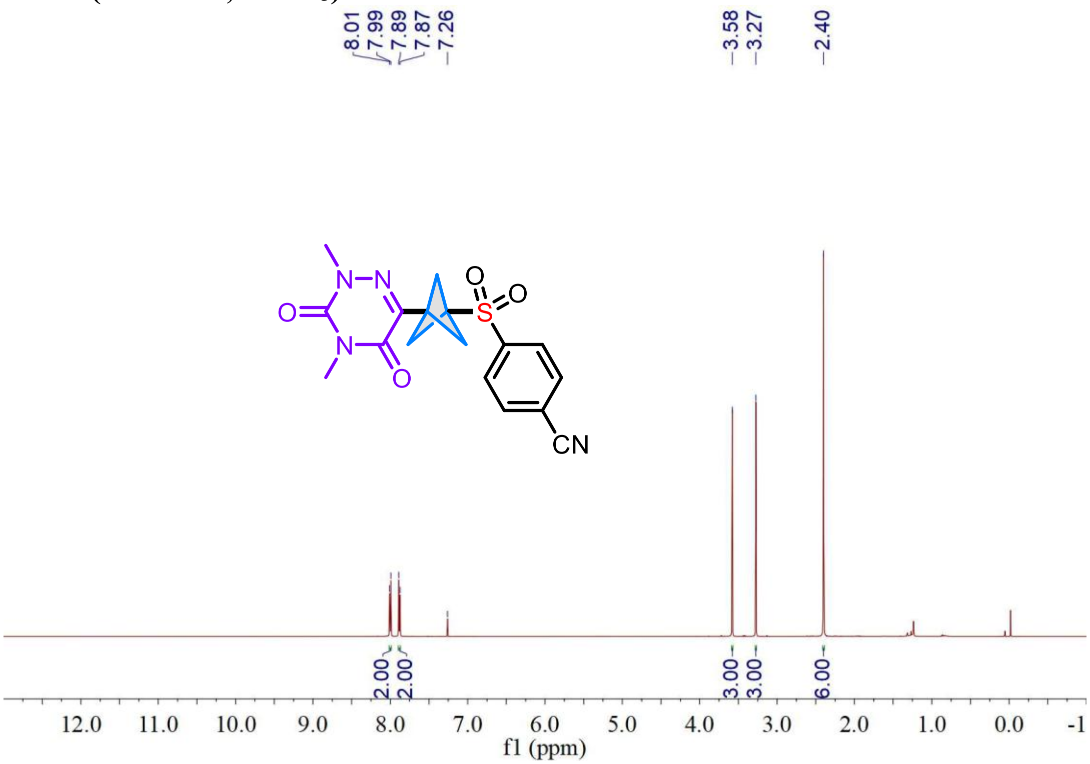

19 ^{13}C NMR (126 MHz, CDCl$_3$)

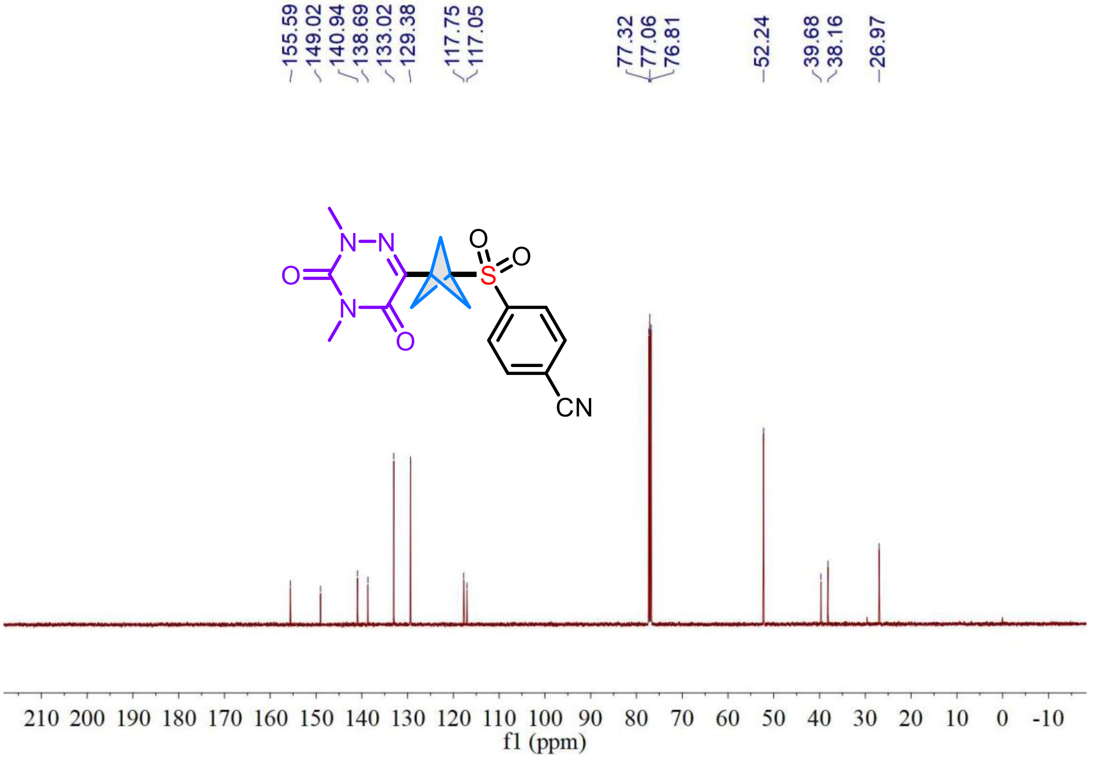

20 ^{1}H NMR (500 MHz, CDCl$_3$)

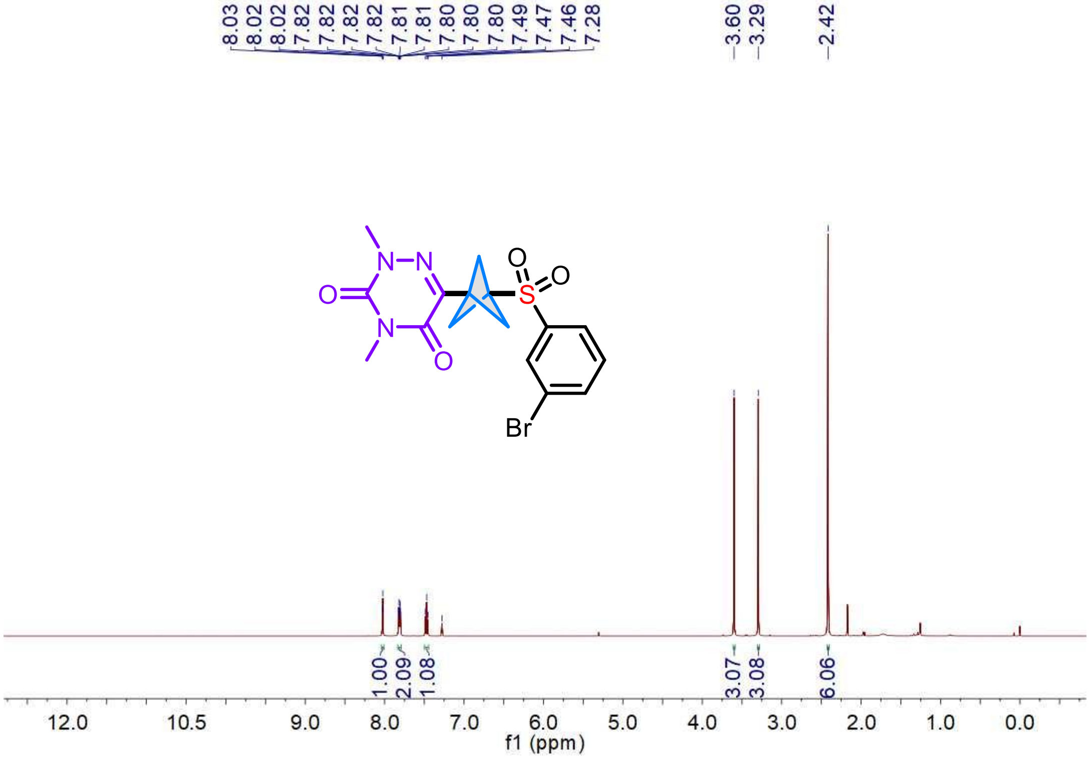

20 ^{13}C NMR (126 MHz, $CDCl_3$)

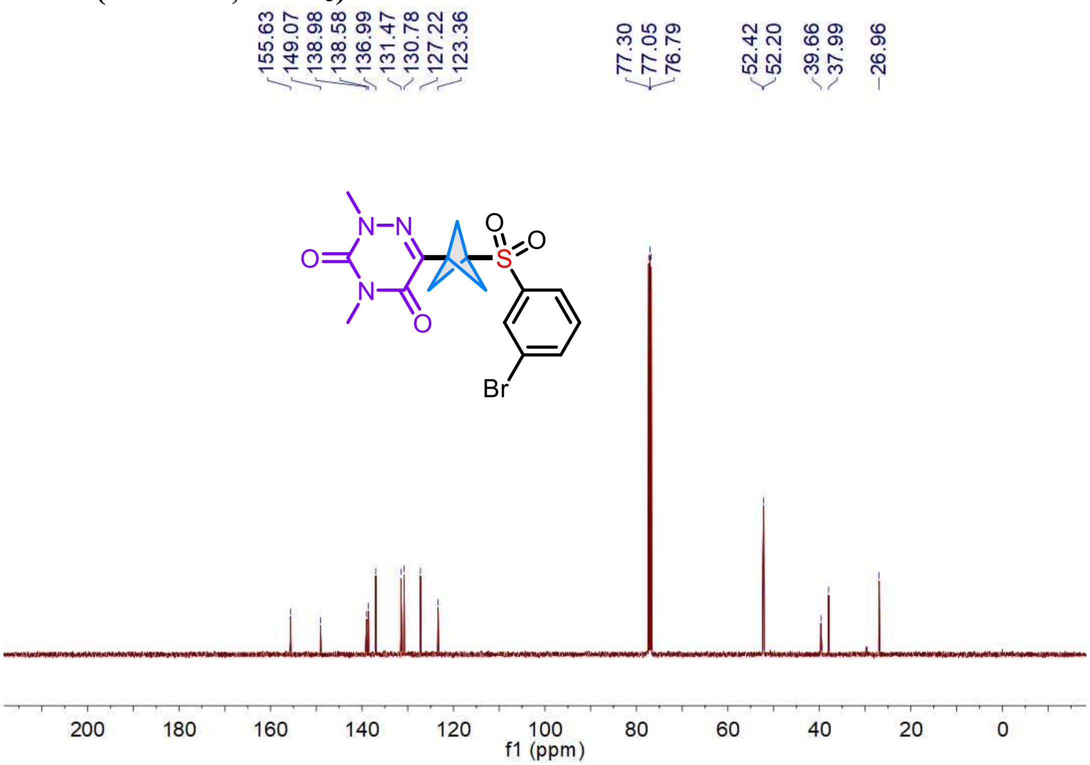

21 ^{1}H NMR (500 MHz, $CDCl_3$)

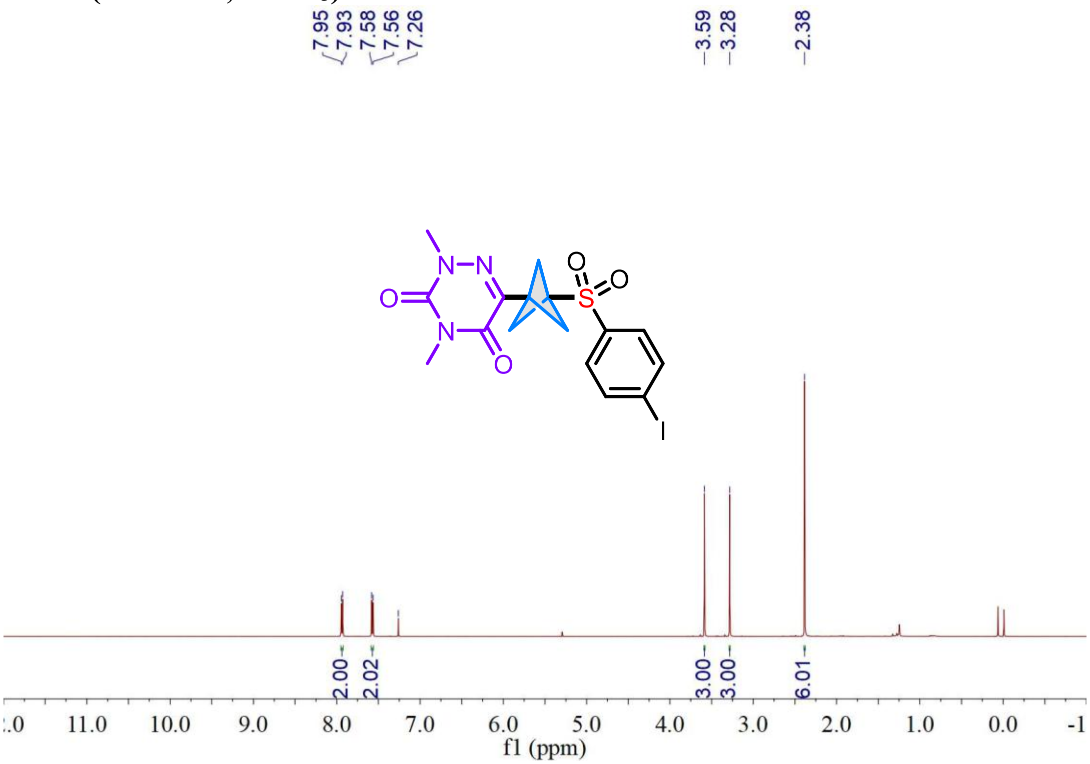

21 ^{13}C NMR (126 MHz, CDCl$_3$)

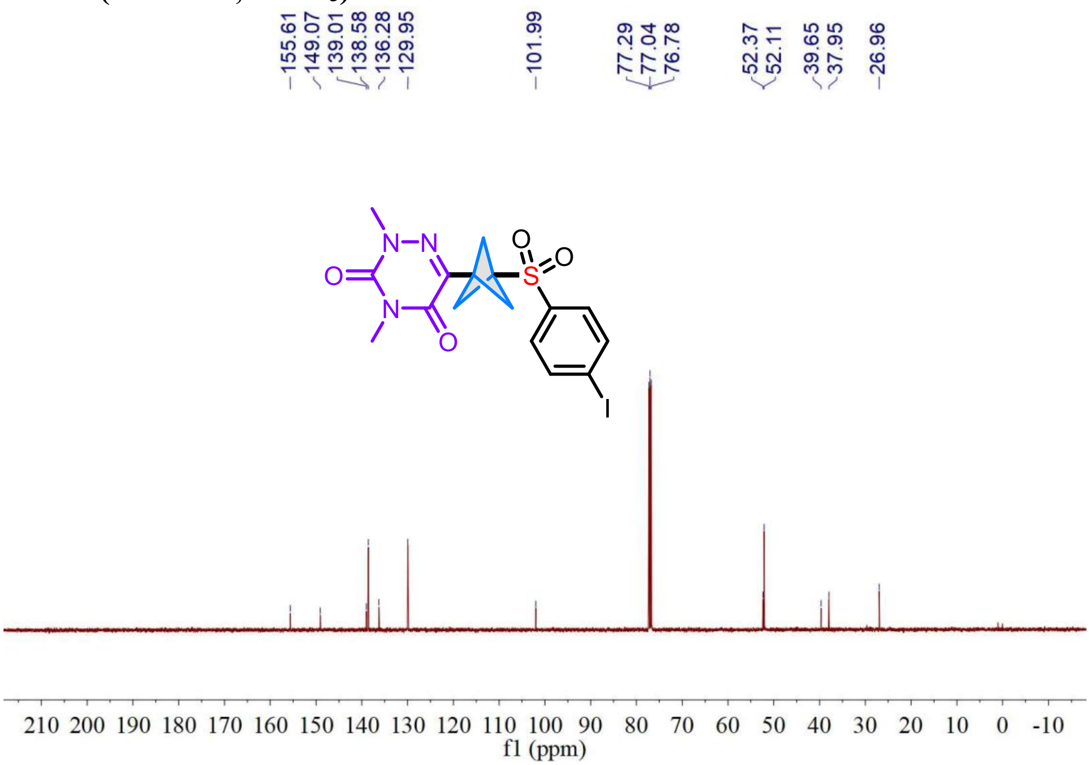

22 ^{1}H NMR (500 MHz, CDCl$_3$)

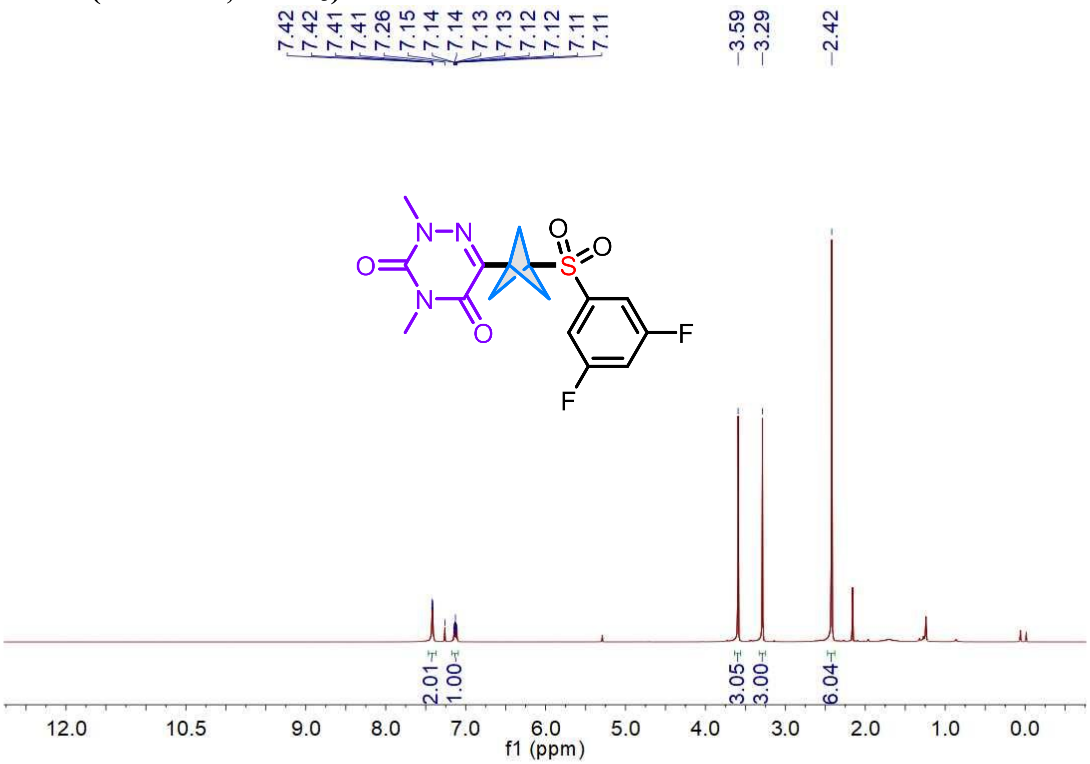

22 ^{13}C NMR (126 MHz, CDCl$_3$)

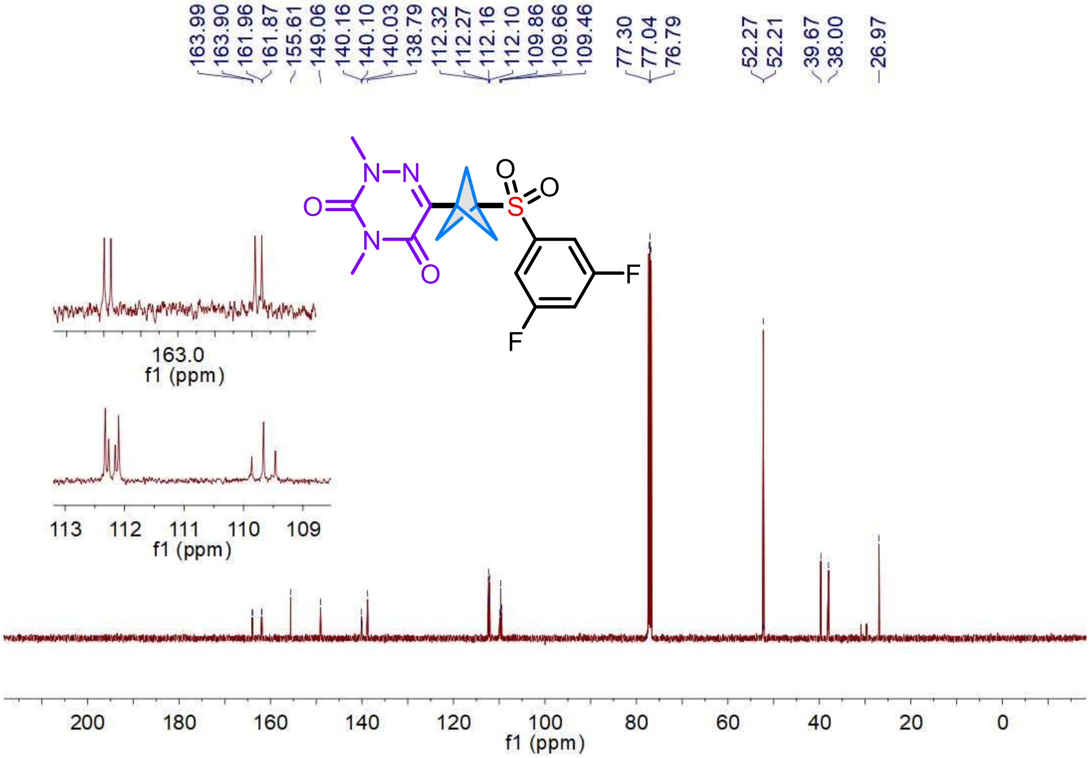

22 ^{19}F NMR (471 MHz, CDCl$_3$)

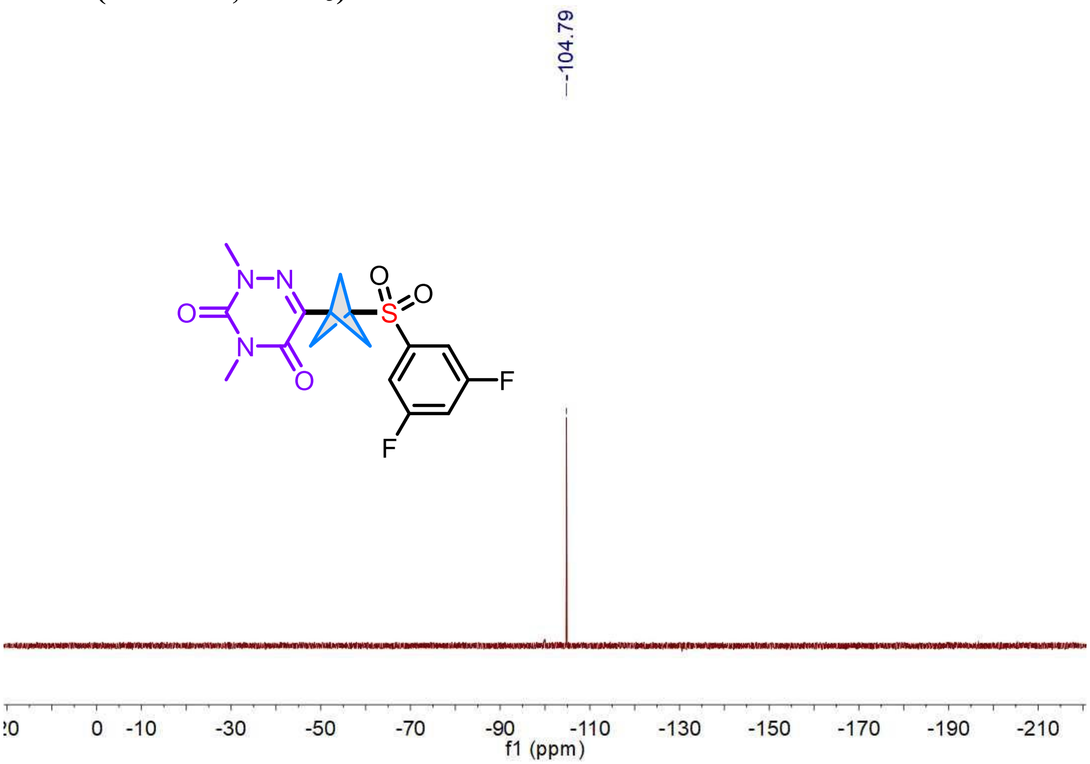

23 ^{1}H NMR (500 MHz, $CDCl_3$)

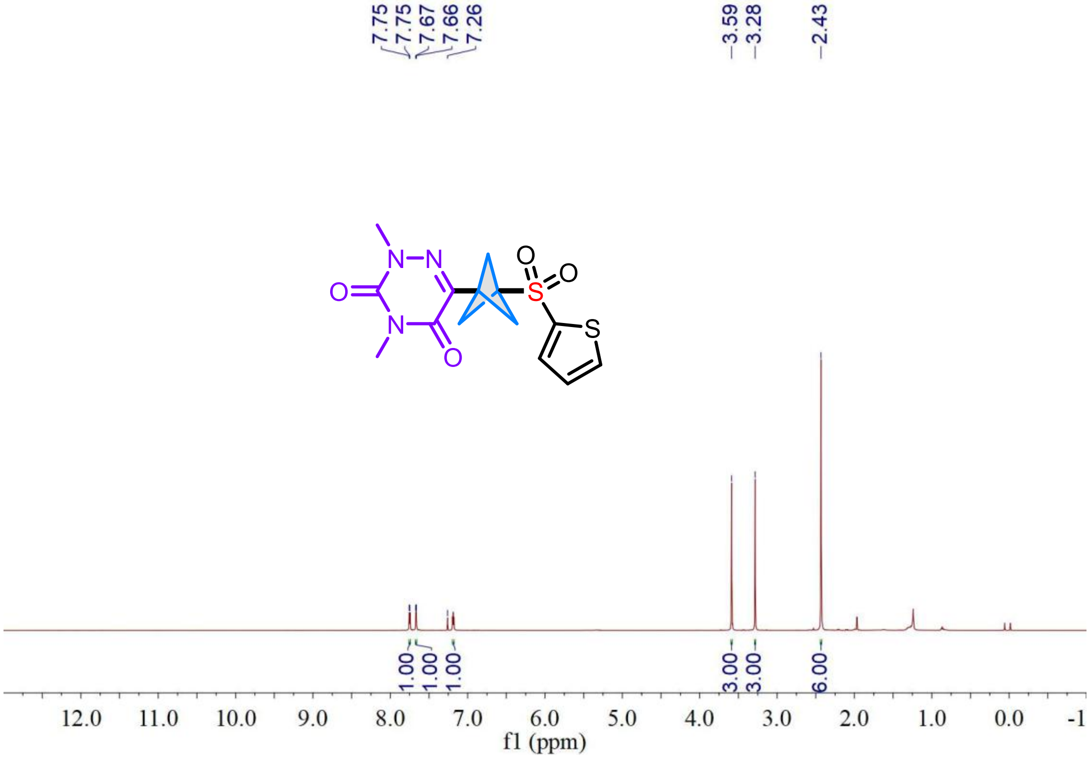

23 ^{13}C NMR (126 MHz, $CDCl_3$)

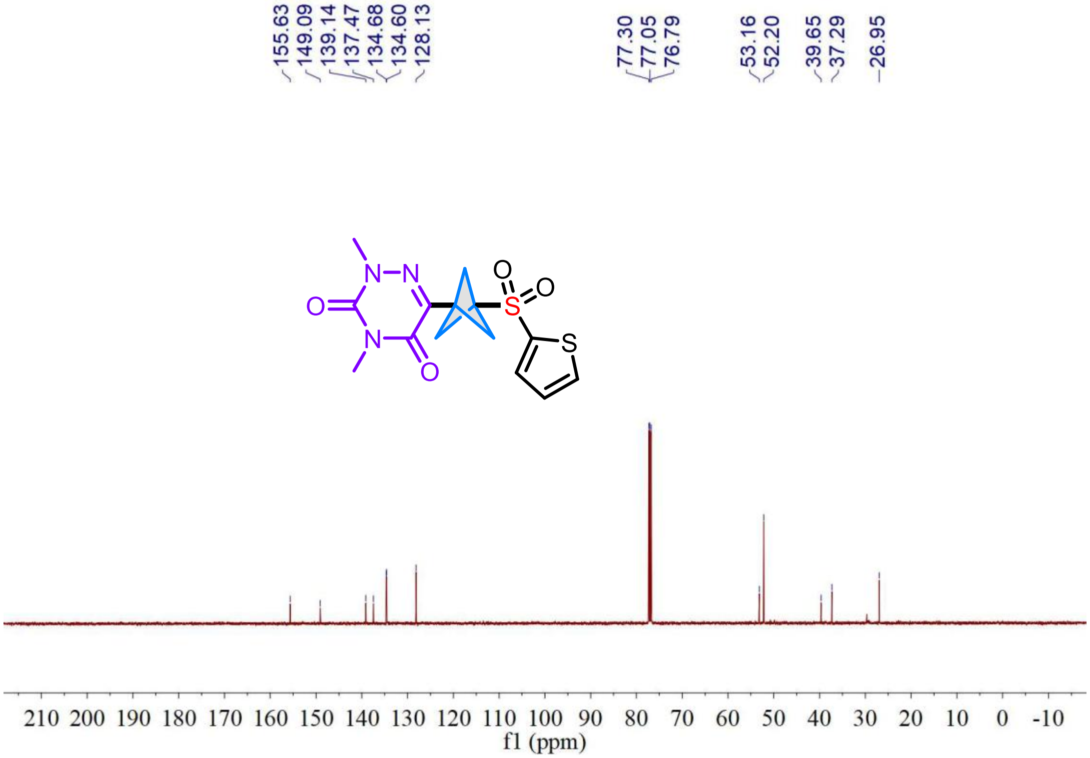

24 ^{1}H NMR (500 MHz, $CDCl_3$)

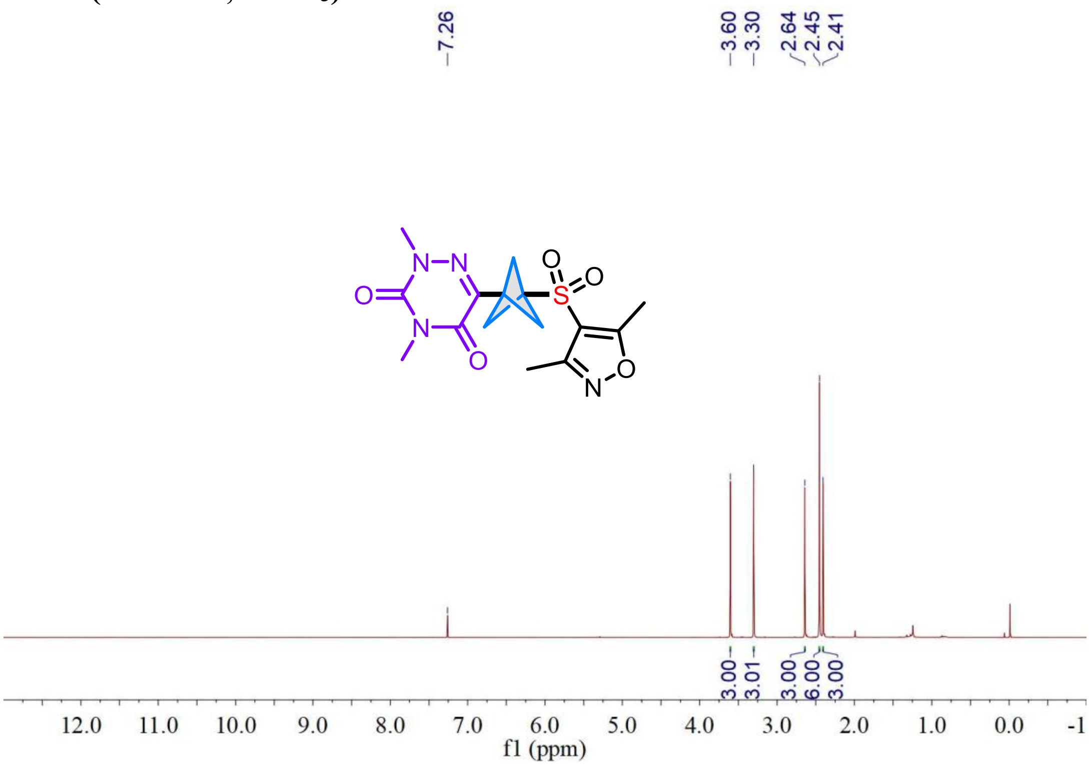

24 ^{13}C NMR (126 MHz, $CDCl_3$)

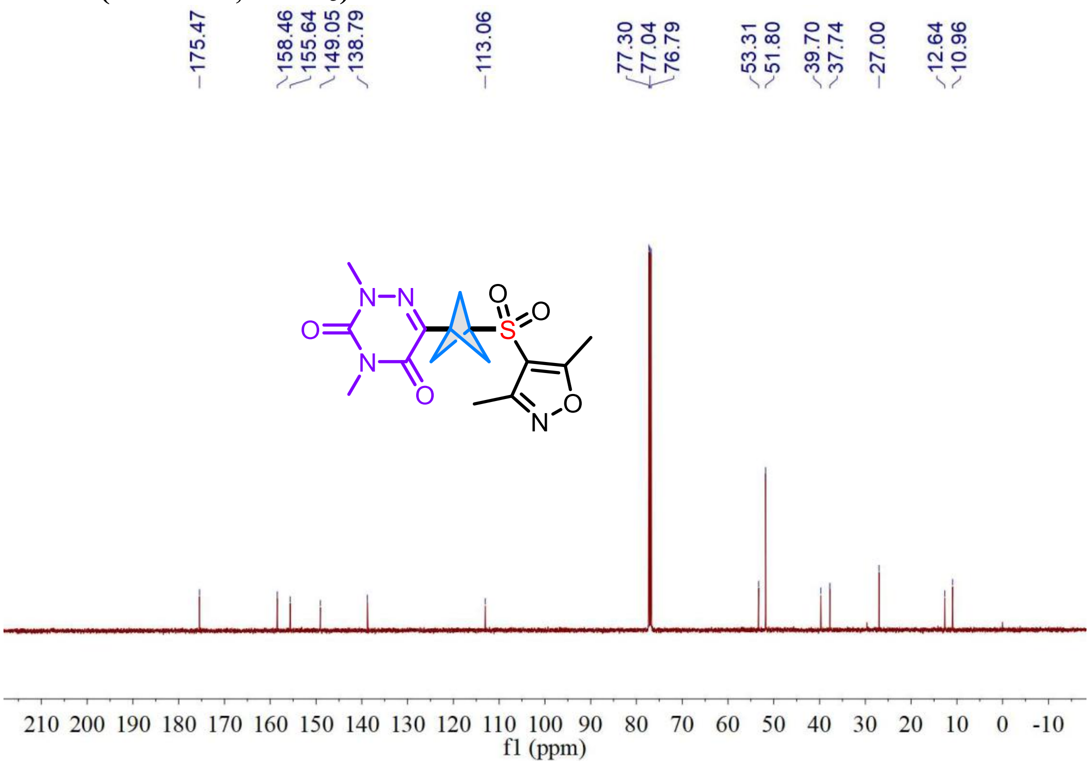

25 ^{1}H NMR (500 MHz, $CDCl_3$)

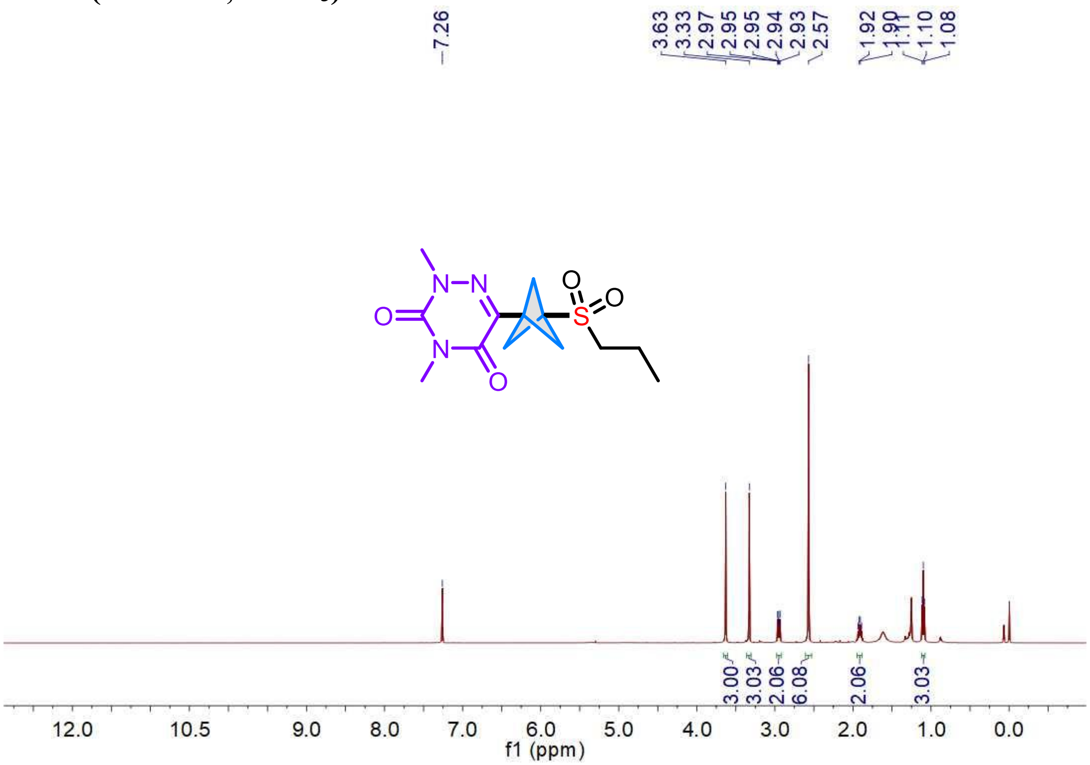

25 ^{13}C NMR (126 MHz, $CDCl_3$)

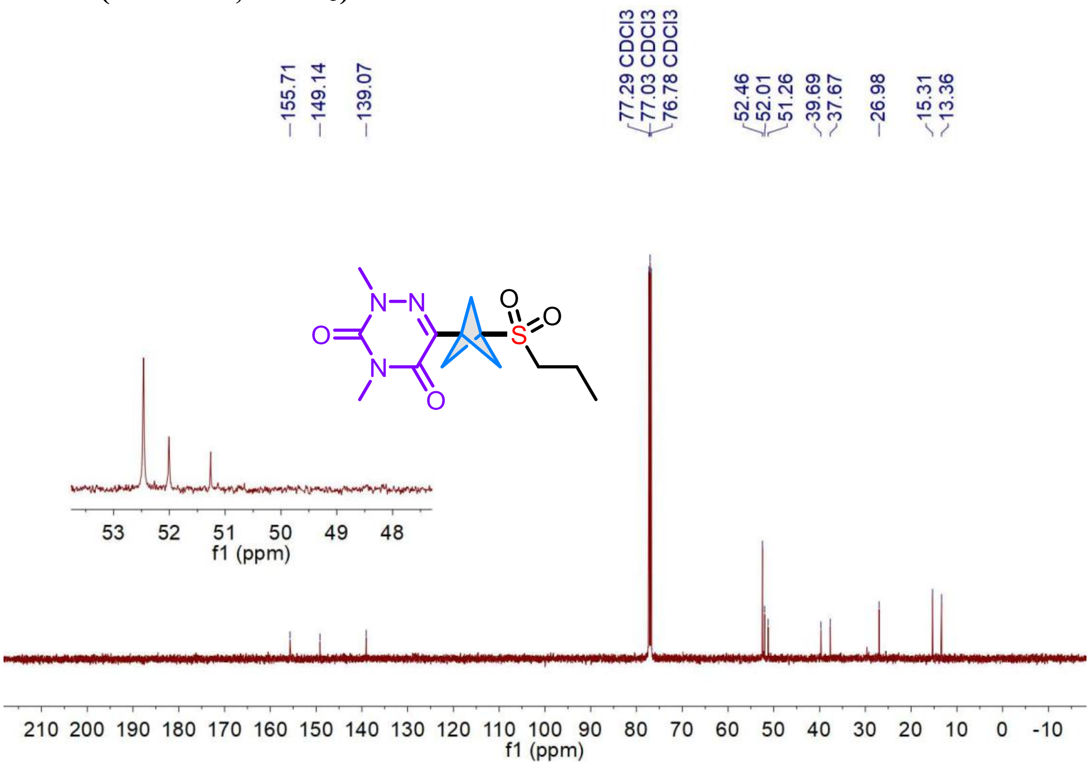

26 ^{1}H NMR (500 MHz, CDCl$_3$)

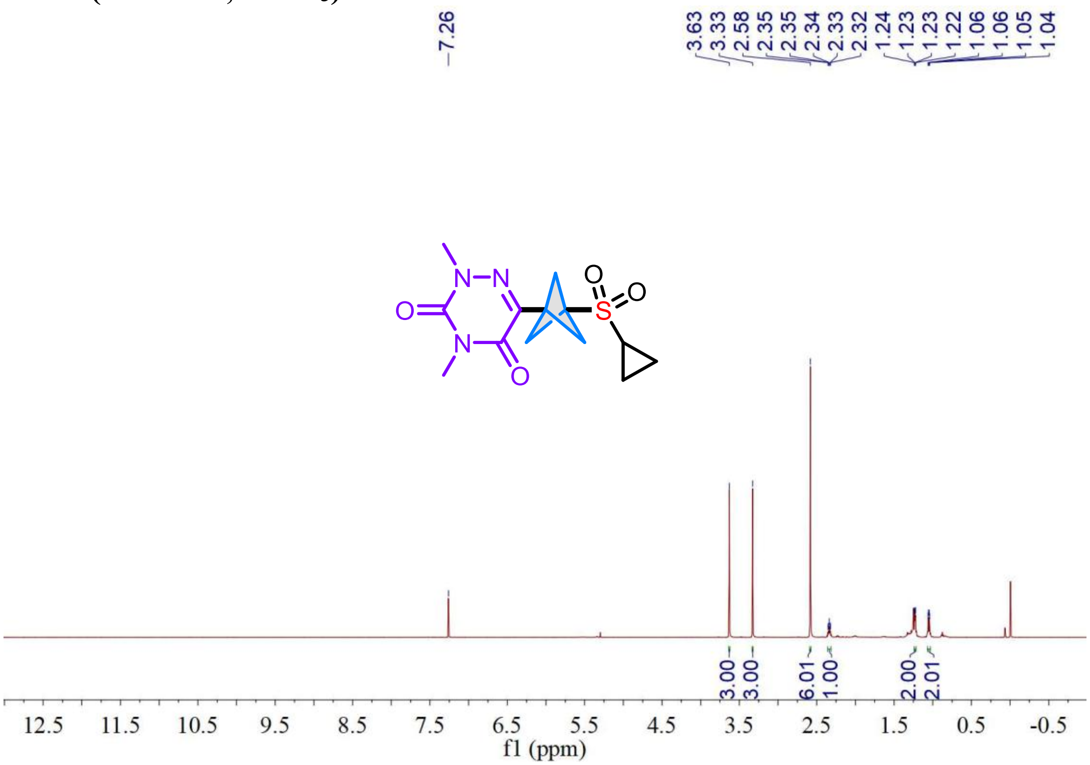

26 ^{13}C NMR (126 MHz, CDCl$_3$)

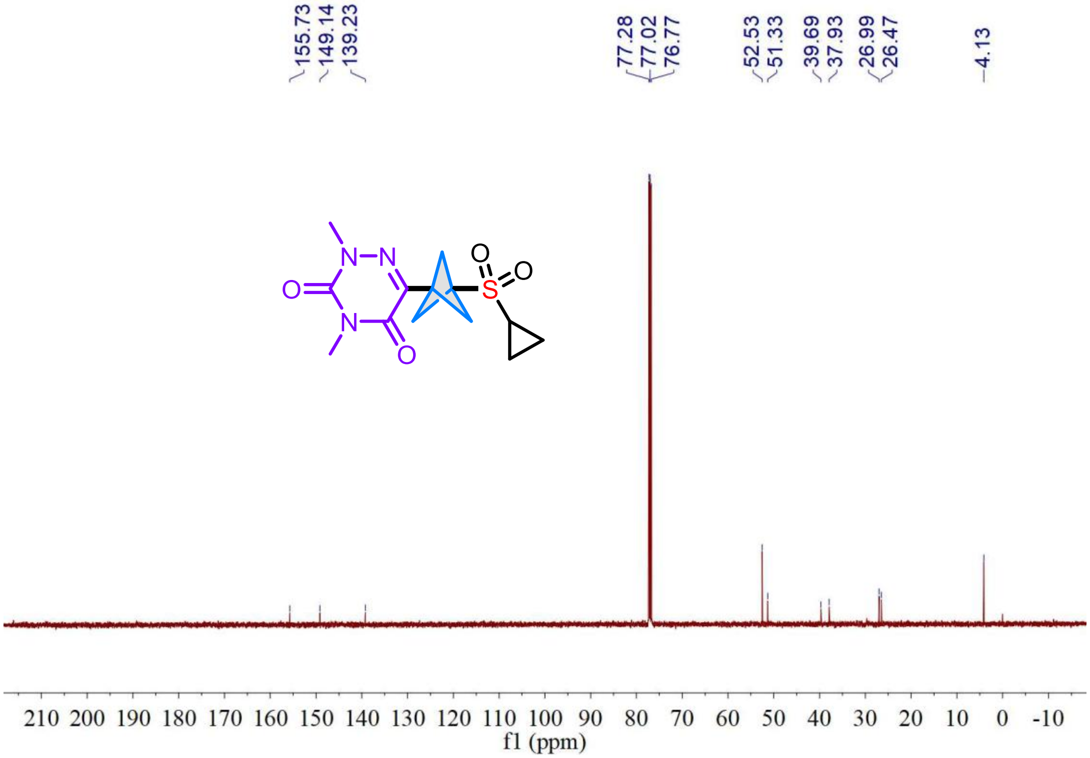

27 ^{1}H NMR (500 MHz, $CDCl_3$)

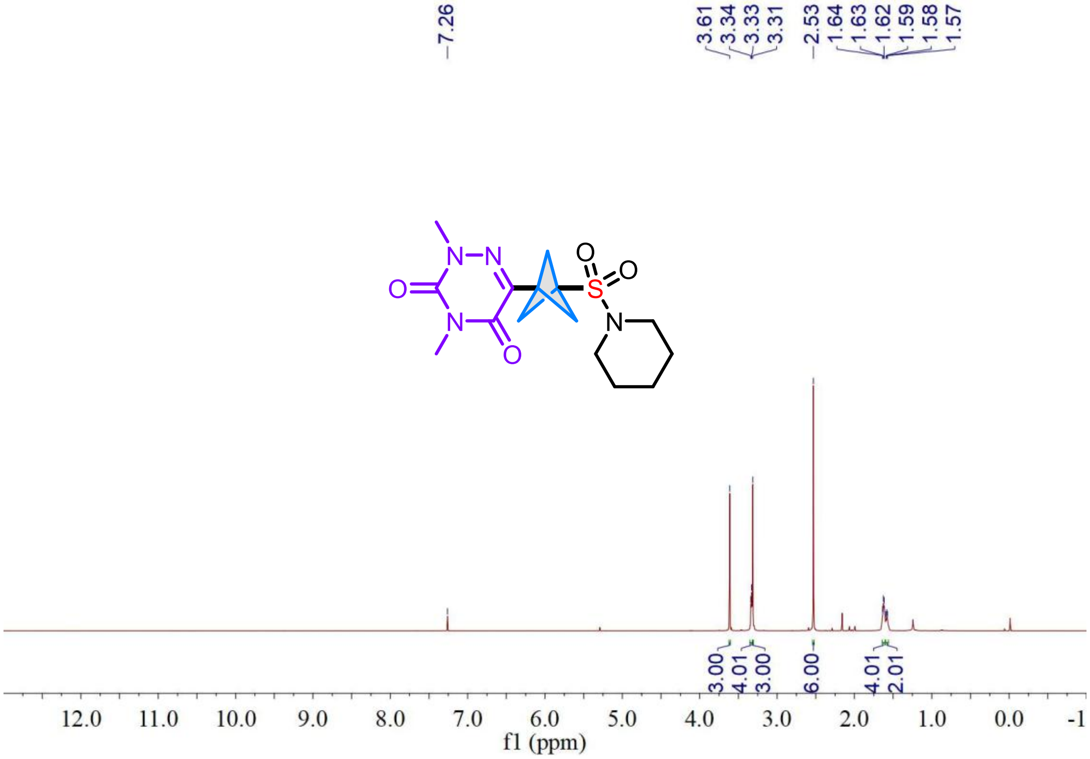

27 ^{13}C NMR (126 MHz, $CDCl_3$)

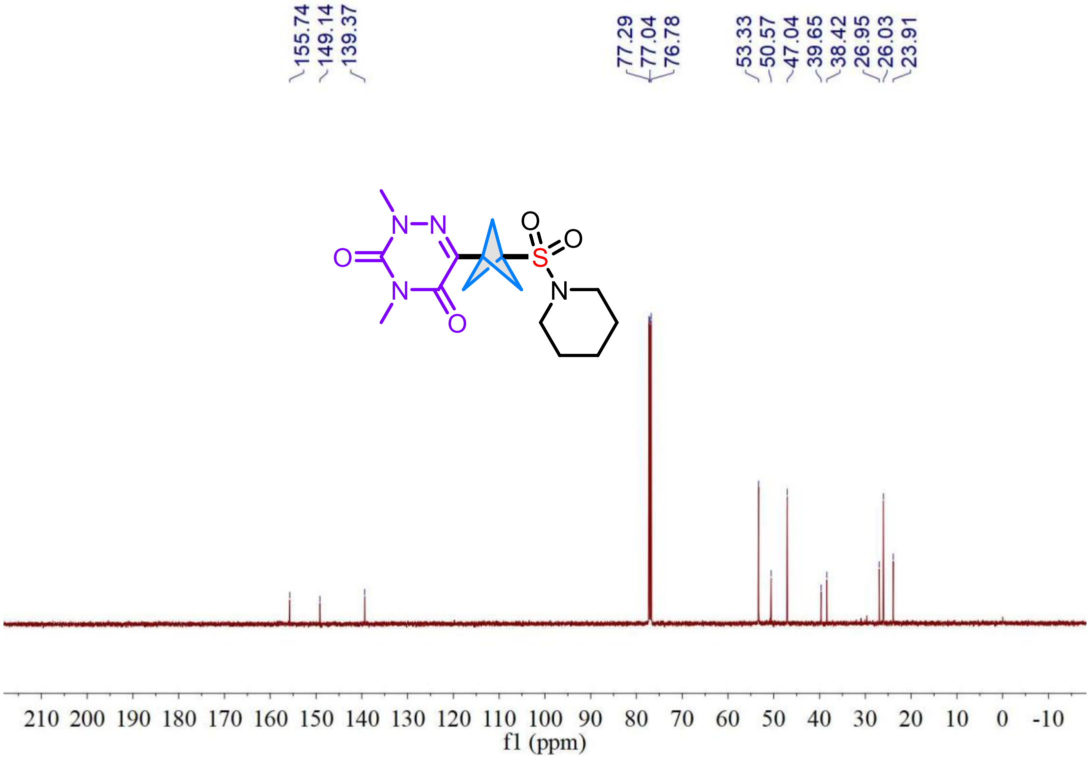

28 ^{1}H NMR (500 MHz, $CDCl_3$)

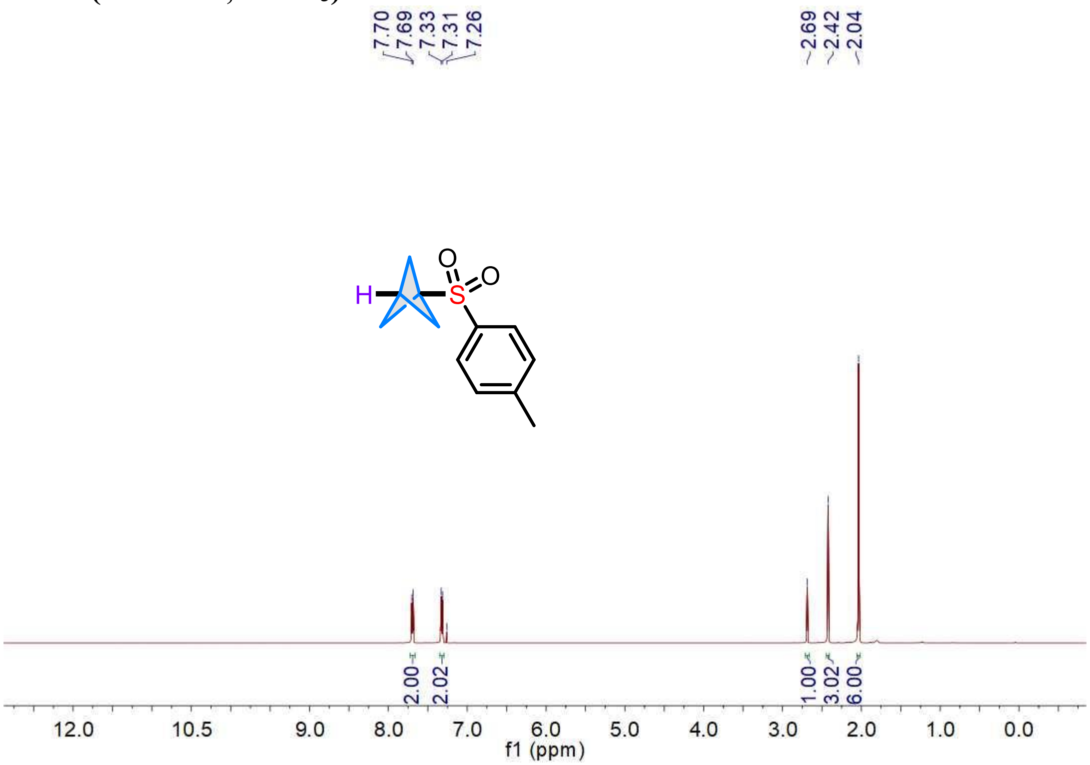

28 ^{13}C NMR (126 MHz, $CDCl_3$)

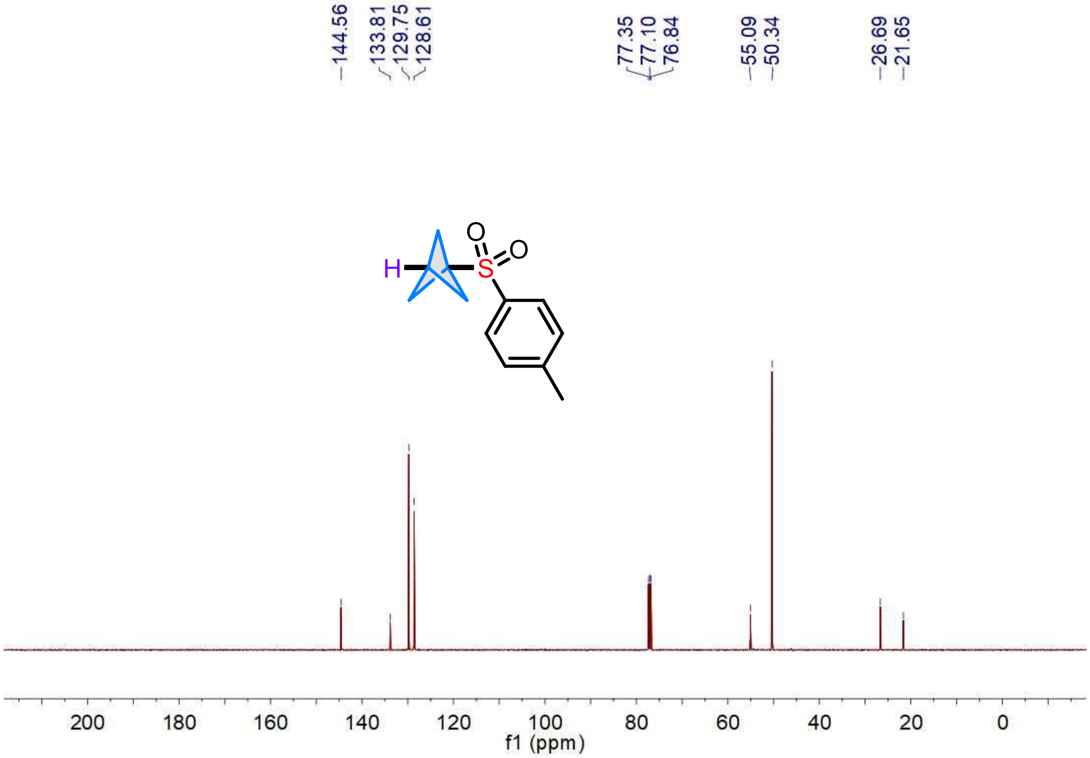

29 ^{1}H NMR (500 MHz, CDCl$_3$)

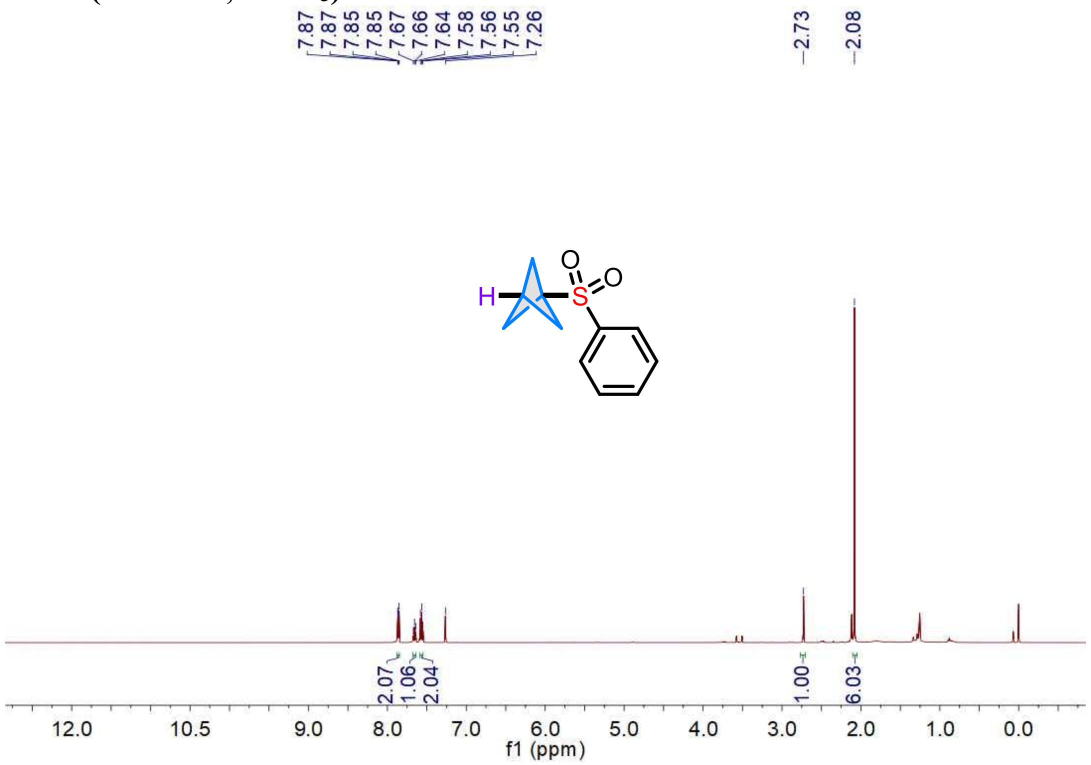

29 ^{13}C NMR (126 MHz, CDCl$_3$)

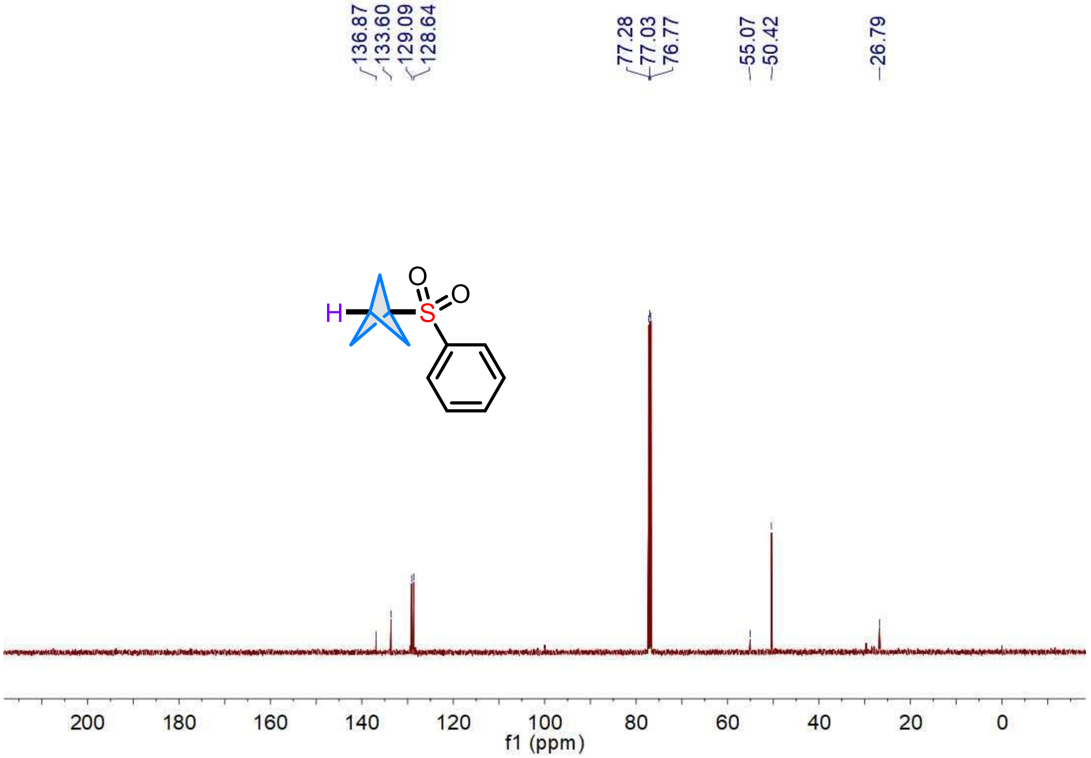

30 ^{1}H NMR (500 MHz, $CDCl_3$)

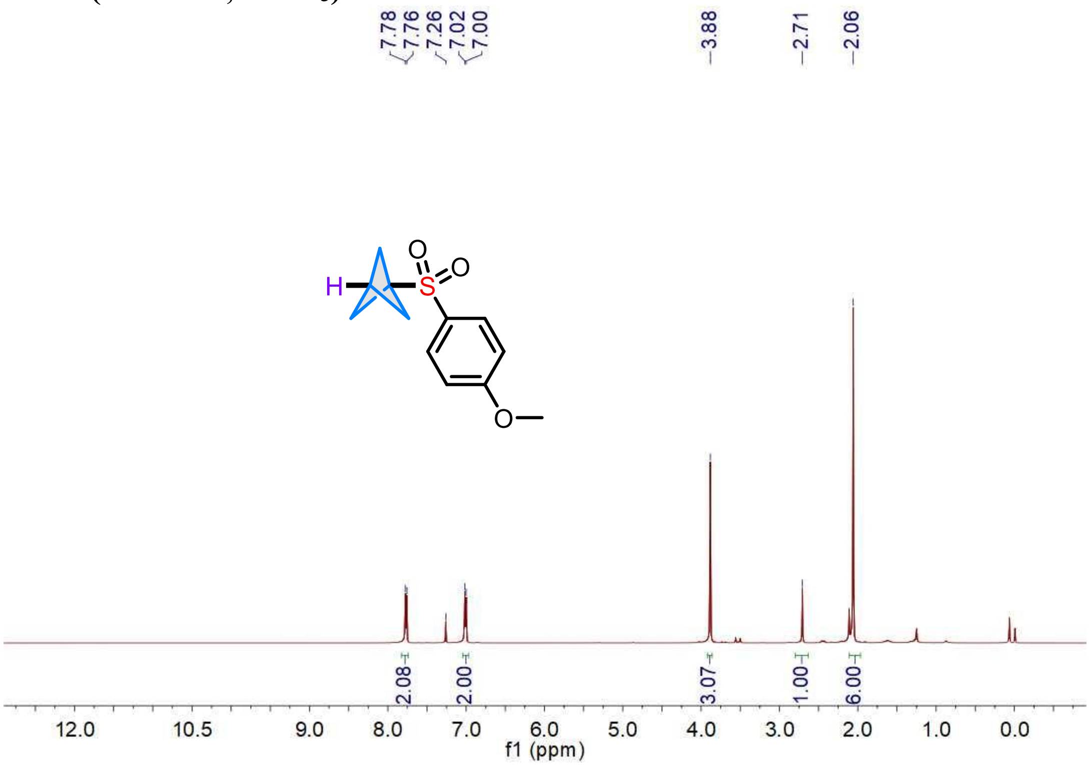

30 ^{13}C NMR (126 MHz, $CDCl_3$)

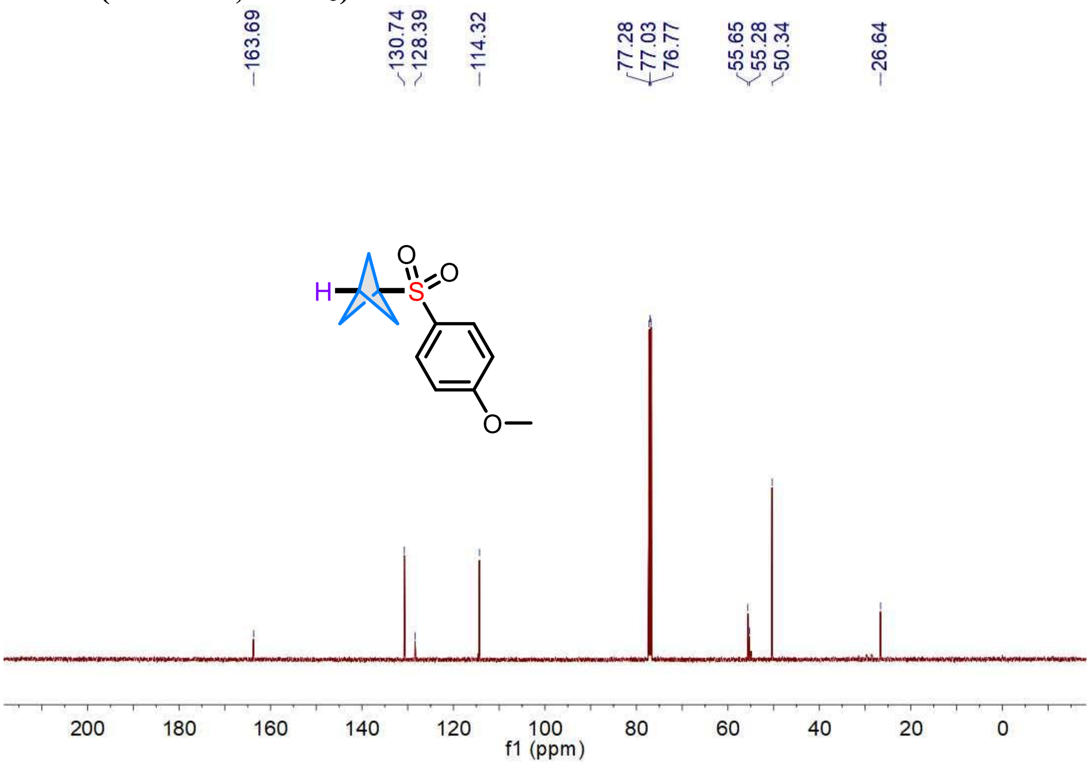

31 ^{1}H NMR (500 MHz, $CDCl_3$)

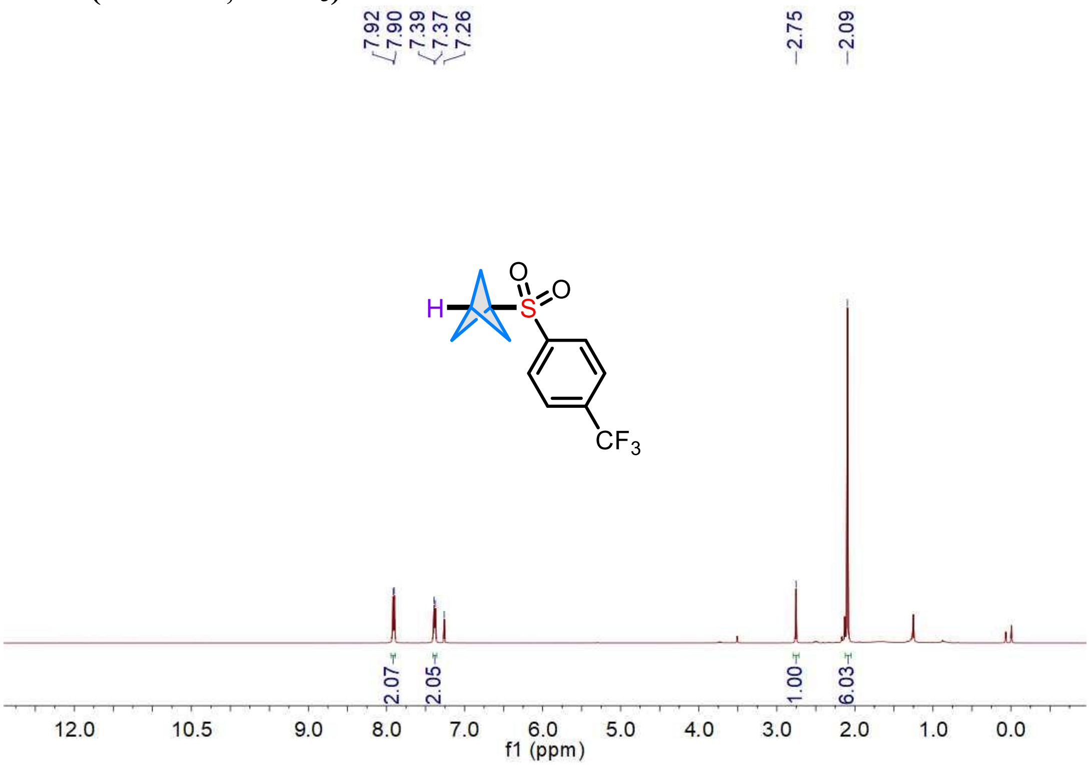

31 ^{13}C NMR (126 MHz, $CDCl_3$)

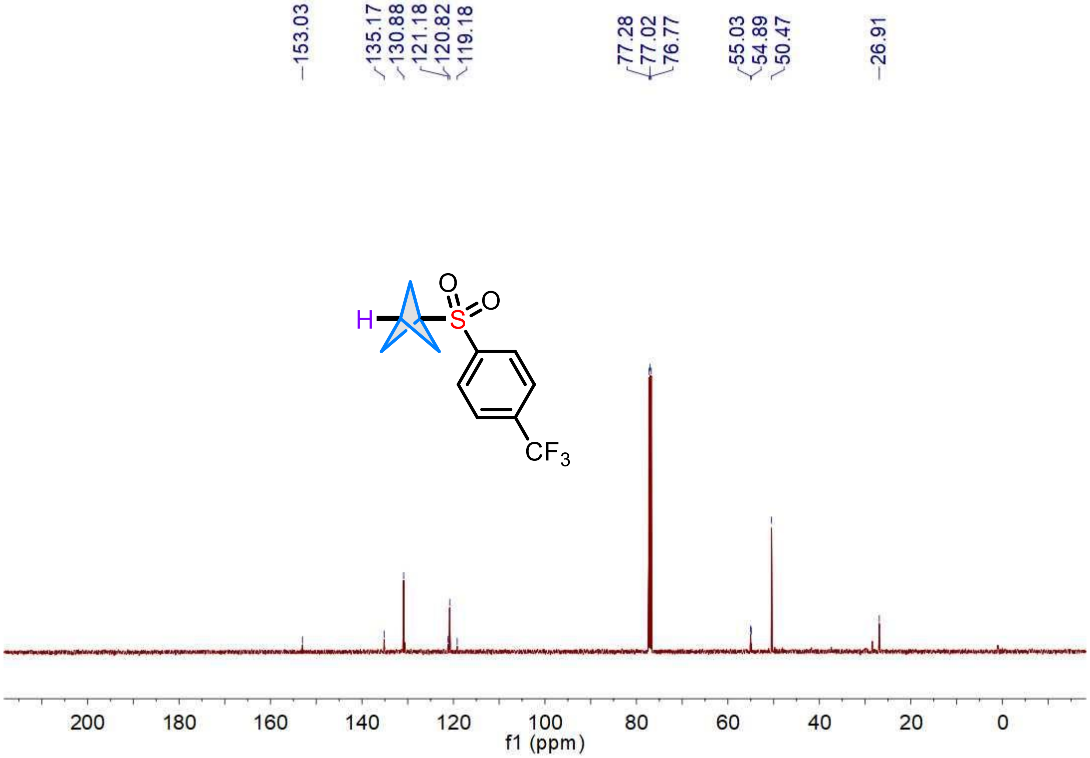

31 ^{19}F NMR (471 MHz, $CDCl_3$)

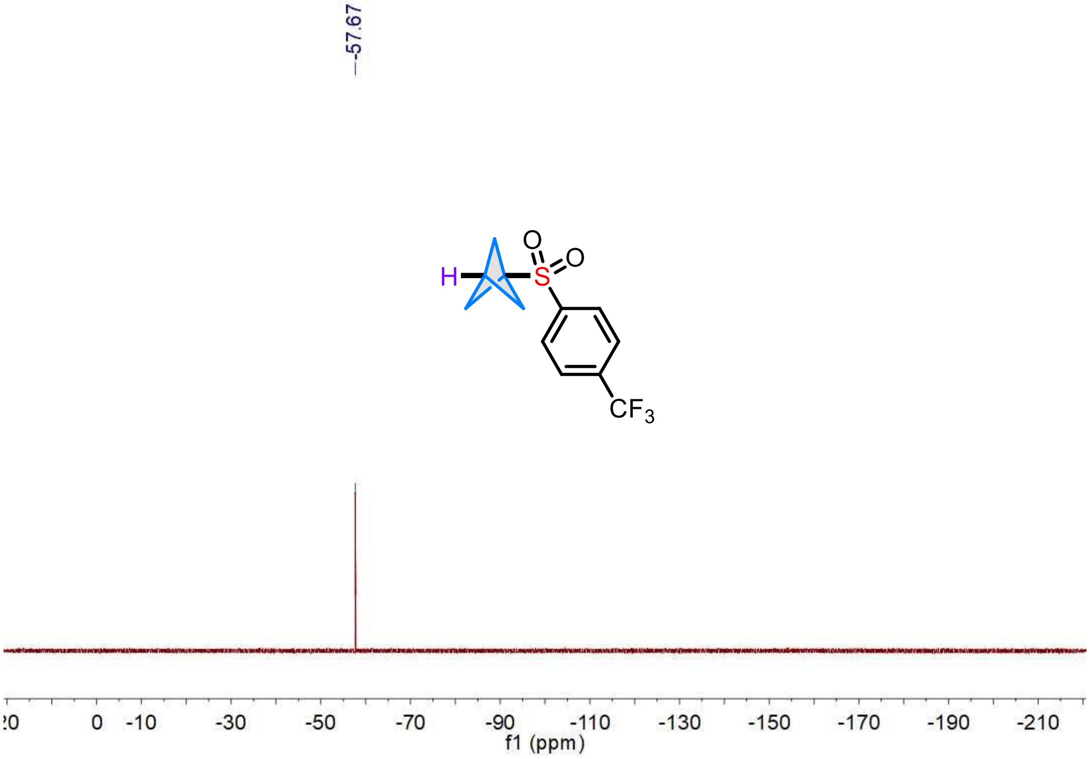

32 ^{1}H NMR (500 MHz, $CDCl_3$)

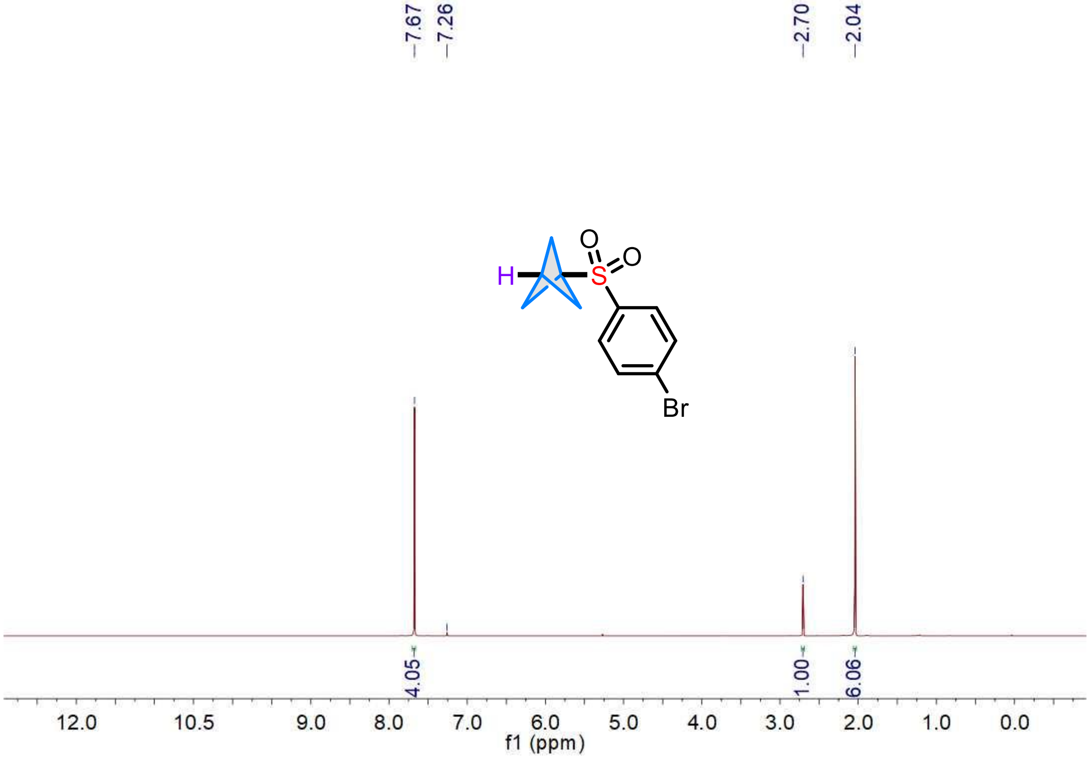

32 ^{13}C NMR (126 MHz, CDCl$_3$)

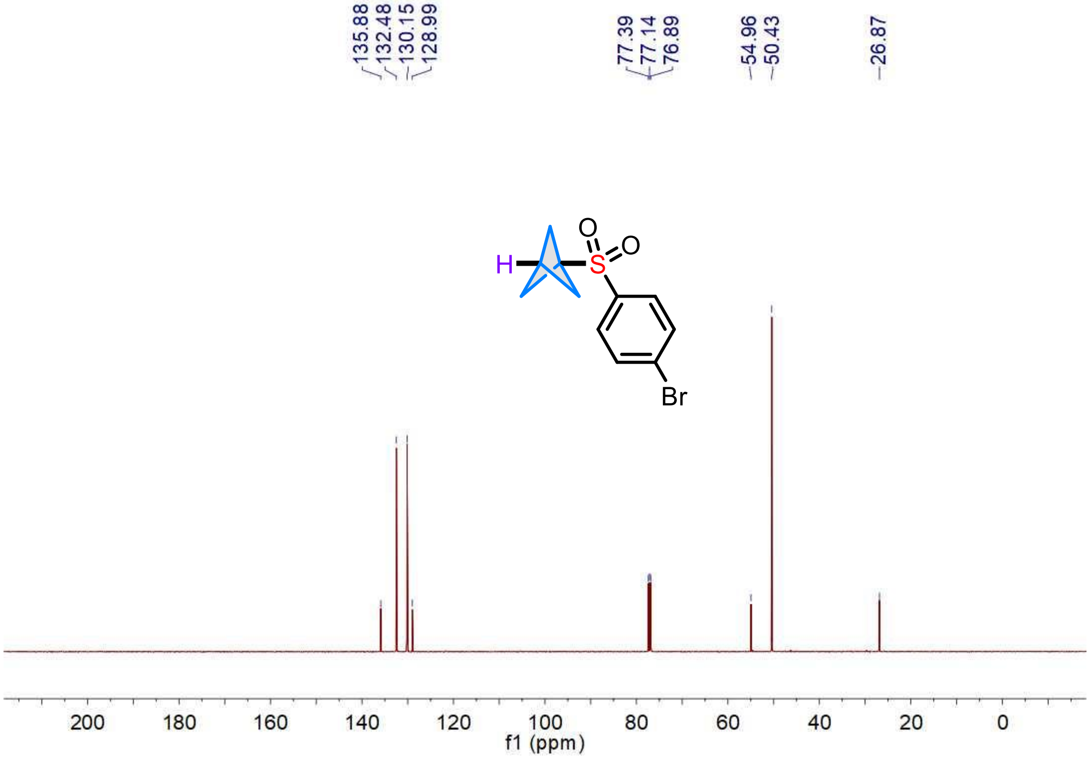

33 ^{1}H NMR (500 MHz, CDCl$_3$)

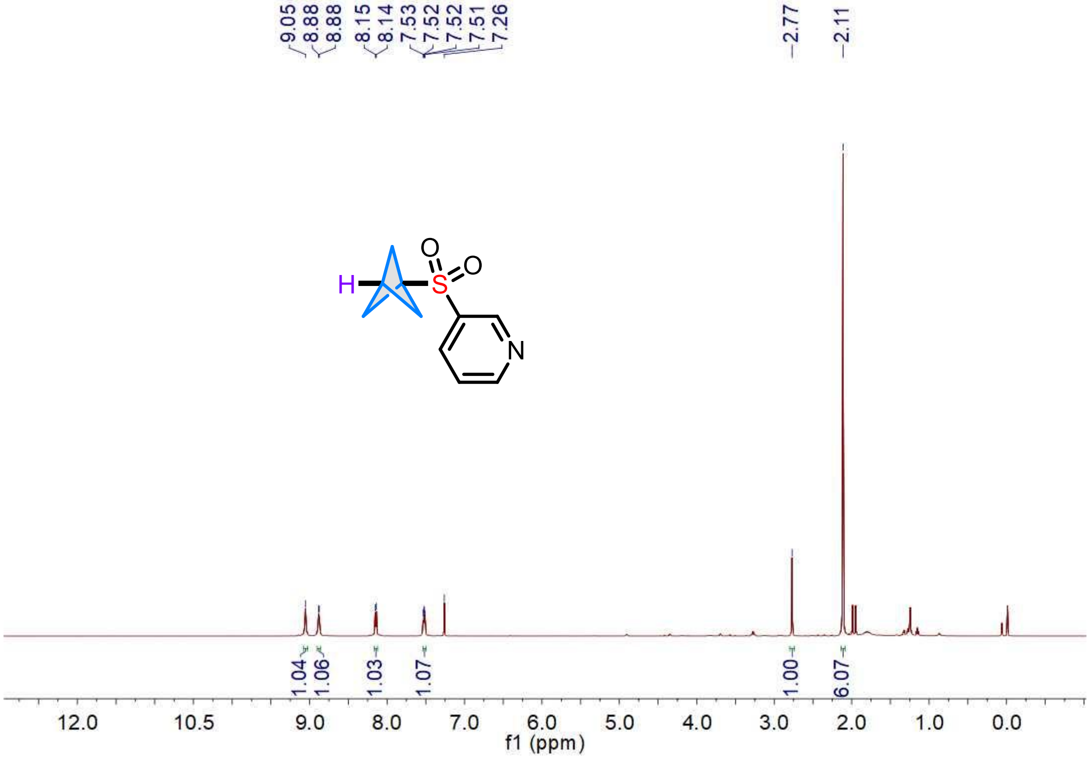

33 ^{13}C NMR (126 MHz, $CDCl_3$)

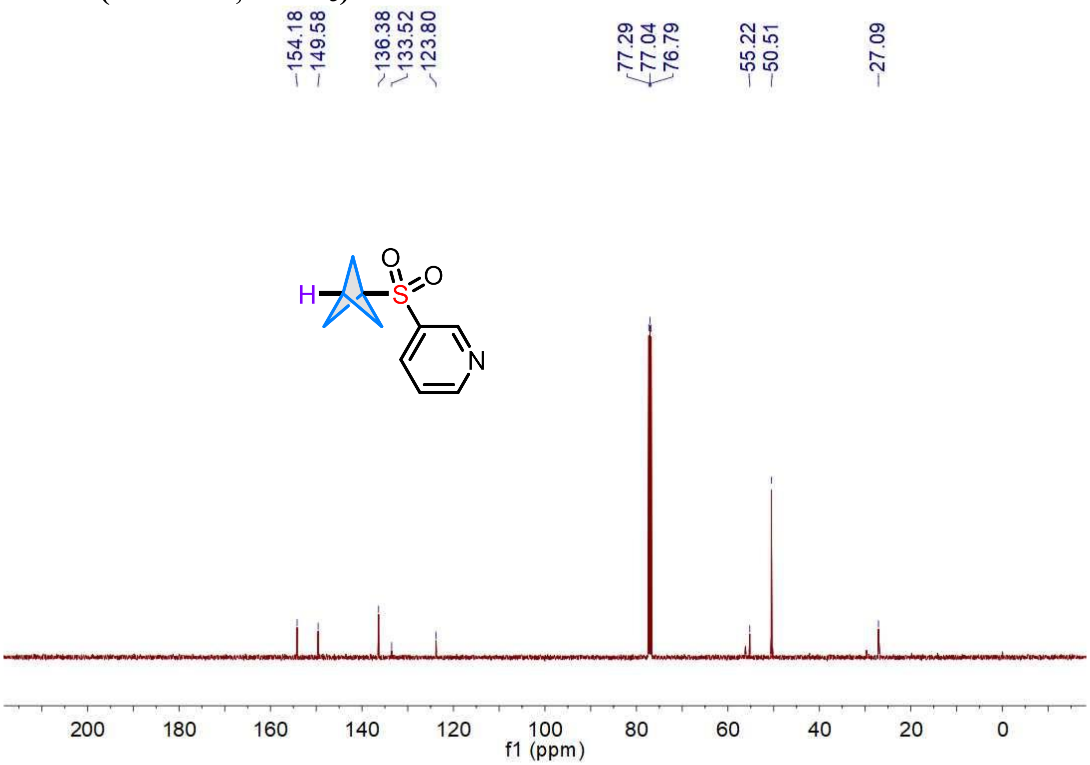

34 ^{1}H NMR (400 MHz, $CDCl_3$)

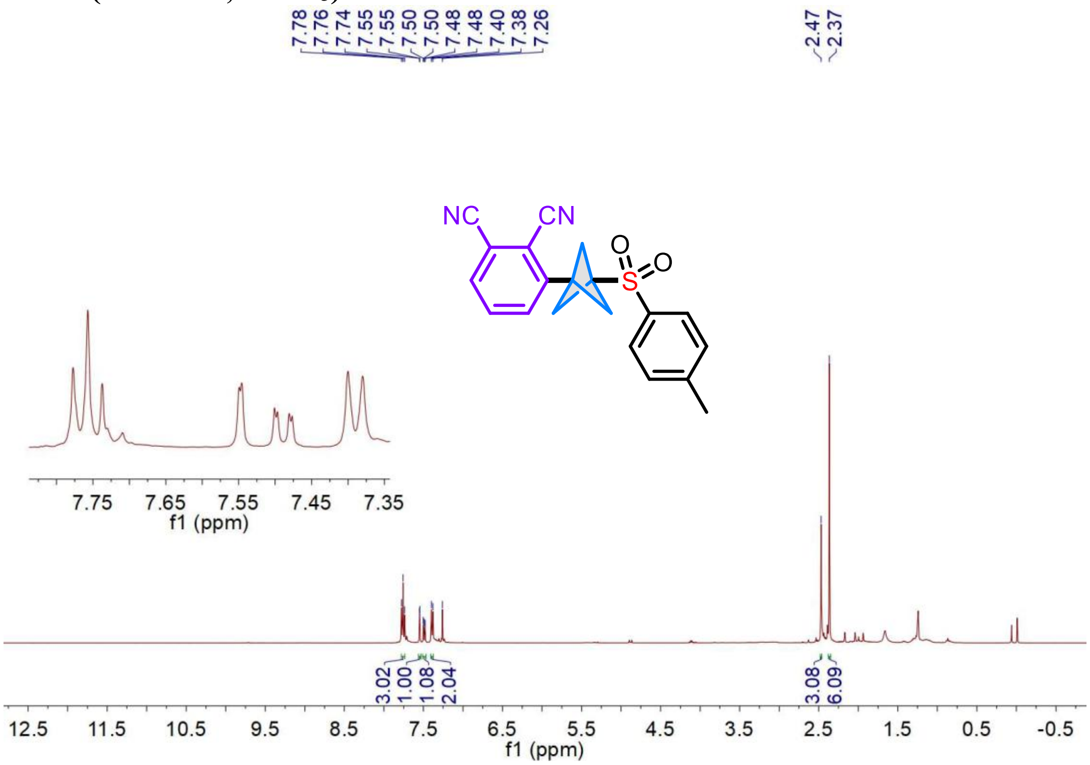

34 ^{13}C NMR (126 MHz, $CDCl_3$)

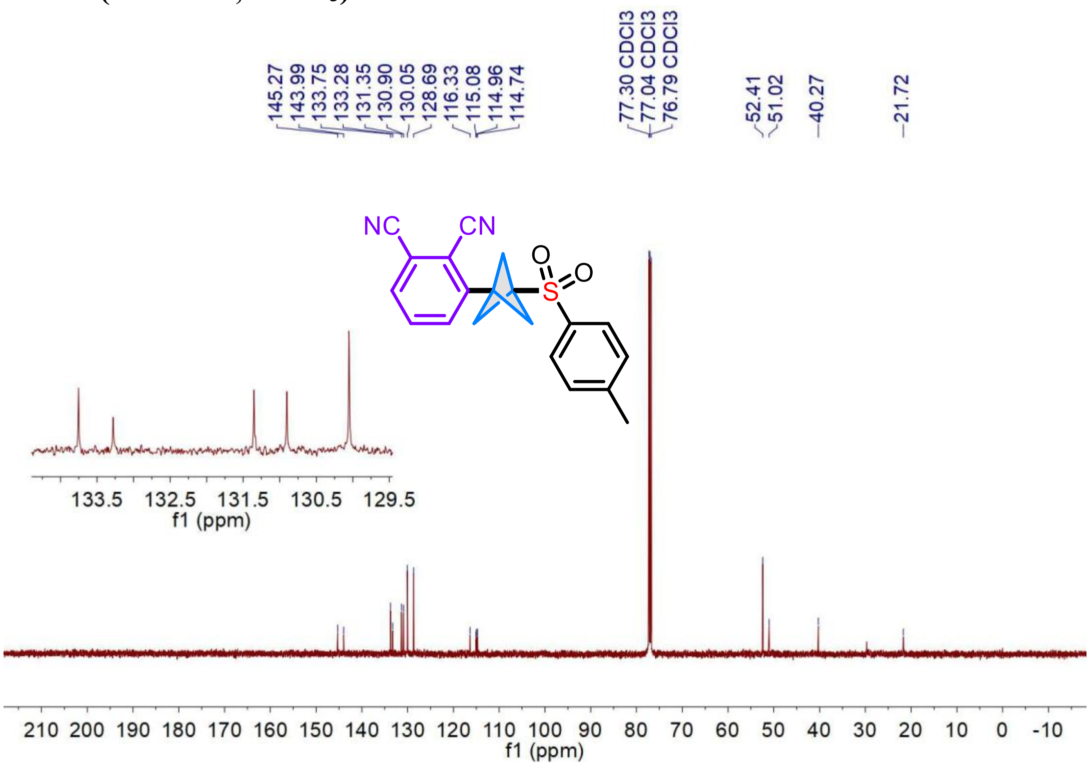

35 ^{1}H NMR (500 MHz, $CDCl_3$)

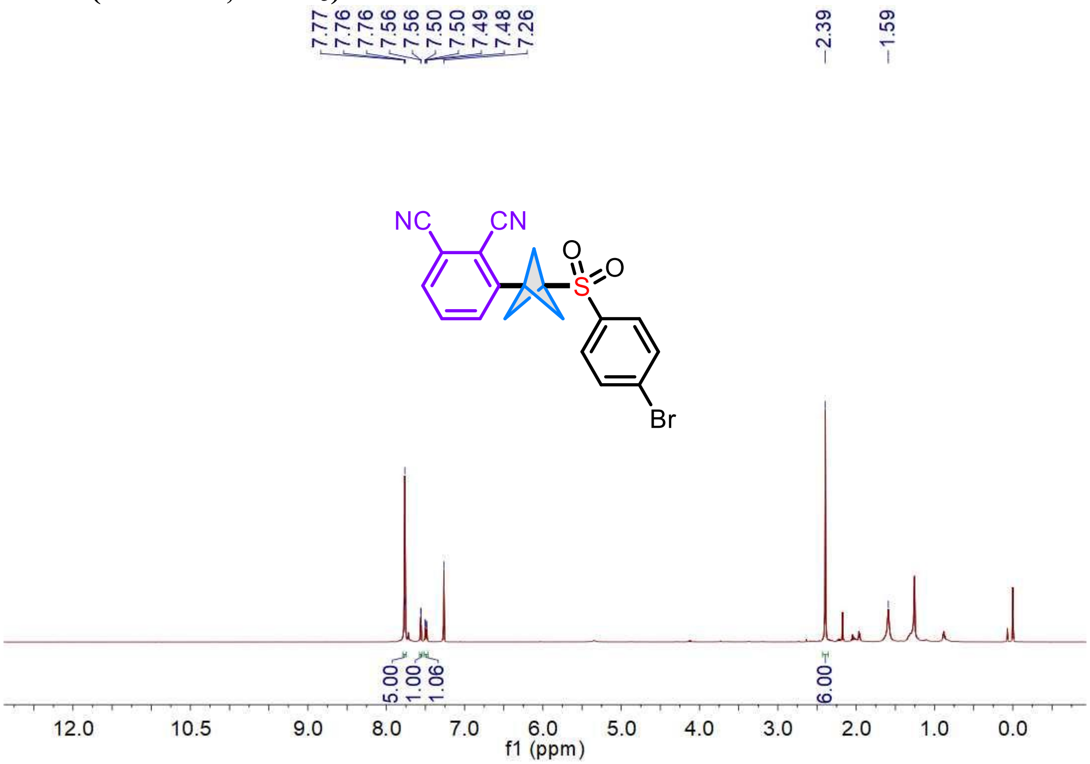

35 ^{13}C NMR (126 MHz, CDCl$_3$)

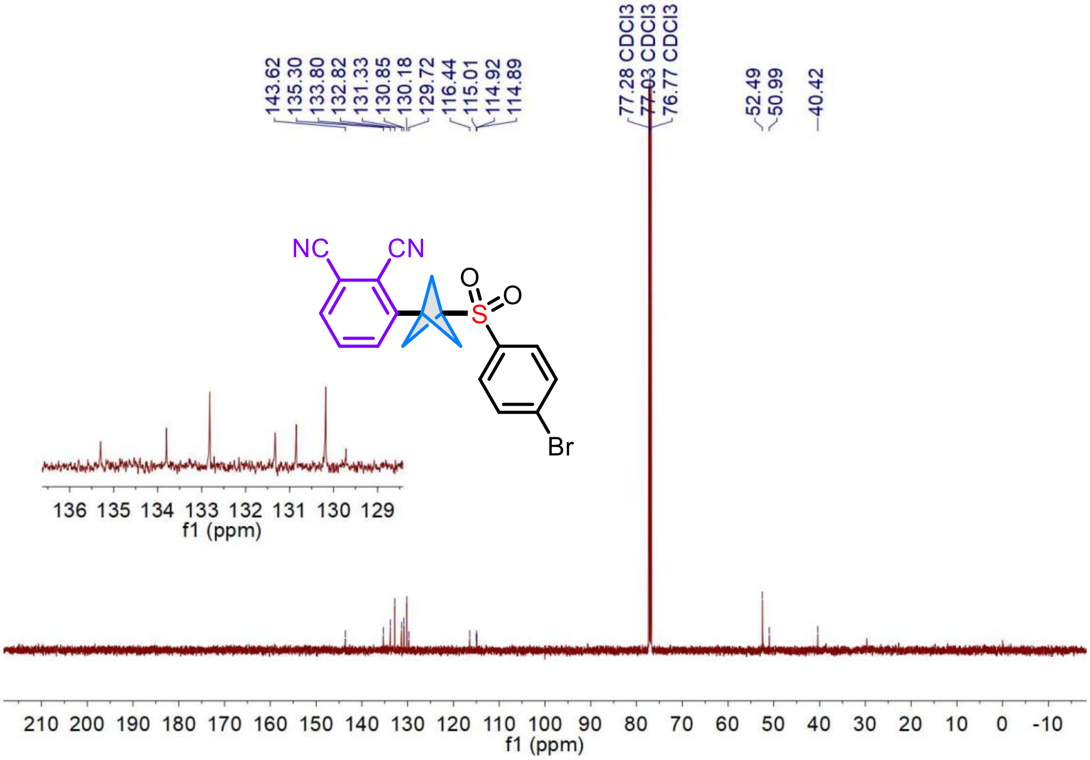

36 ^{1}H NMR (500 MHz, CDCl$_3$)

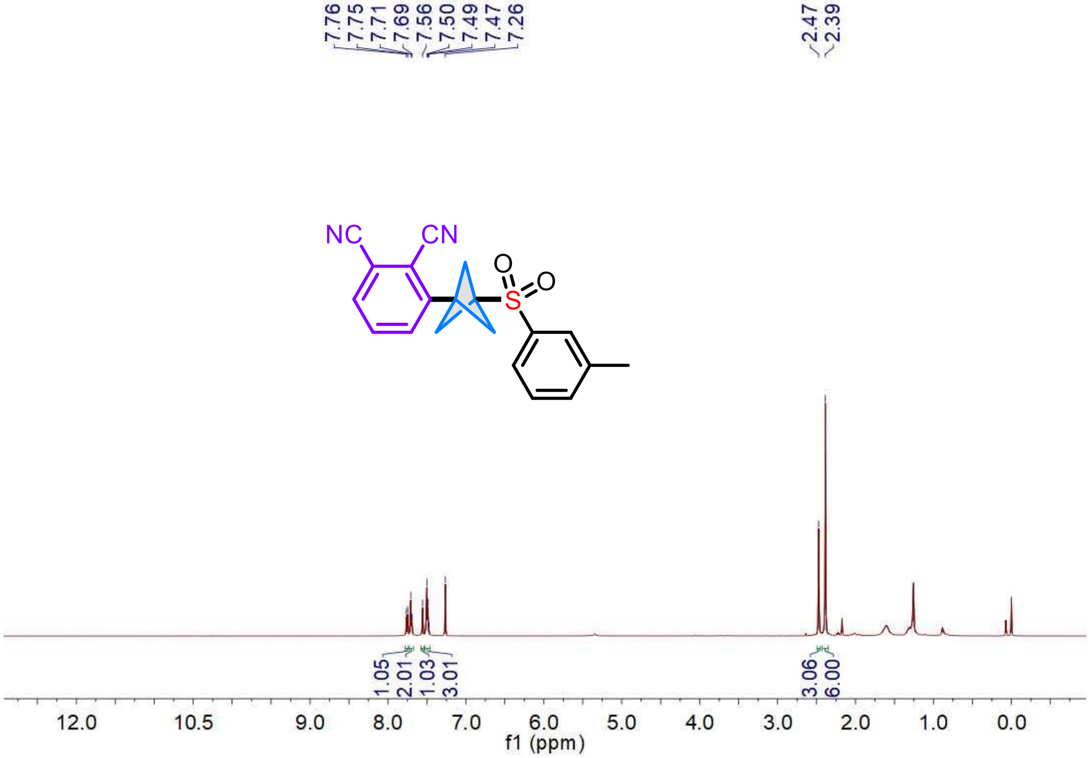

36 ^{13}C NMR (126 MHz, CDCl$_3$)

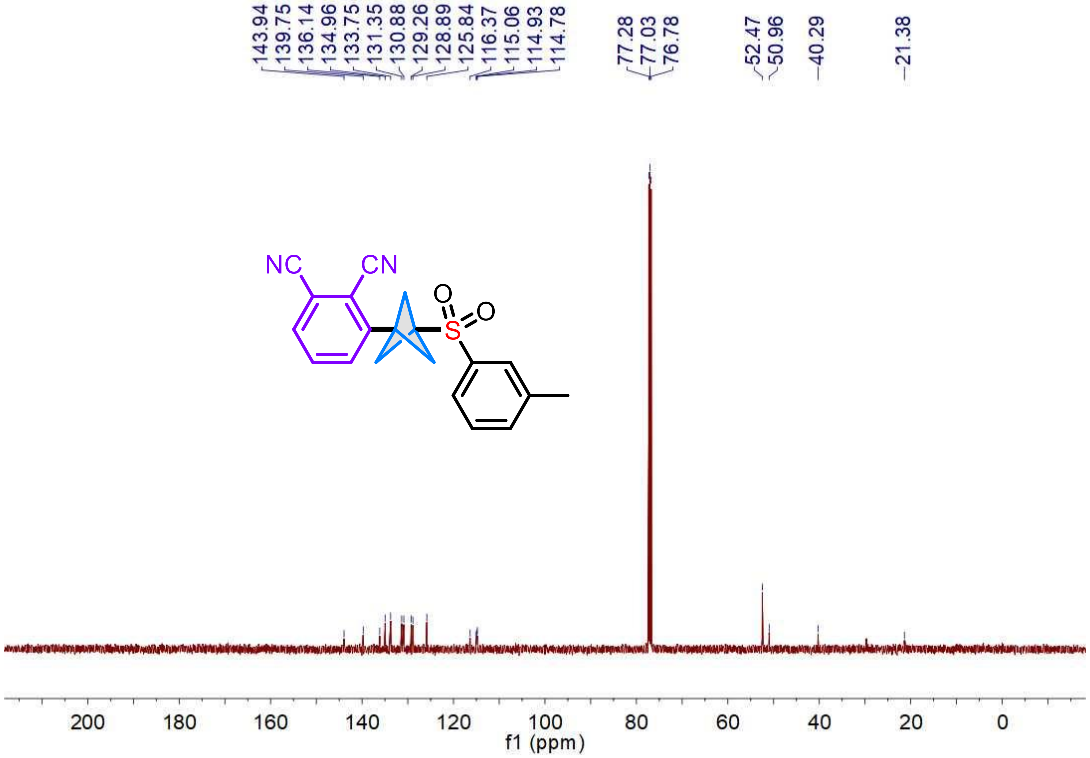

37 ^{1}H NMR (500 MHz, CDCl$_3$)

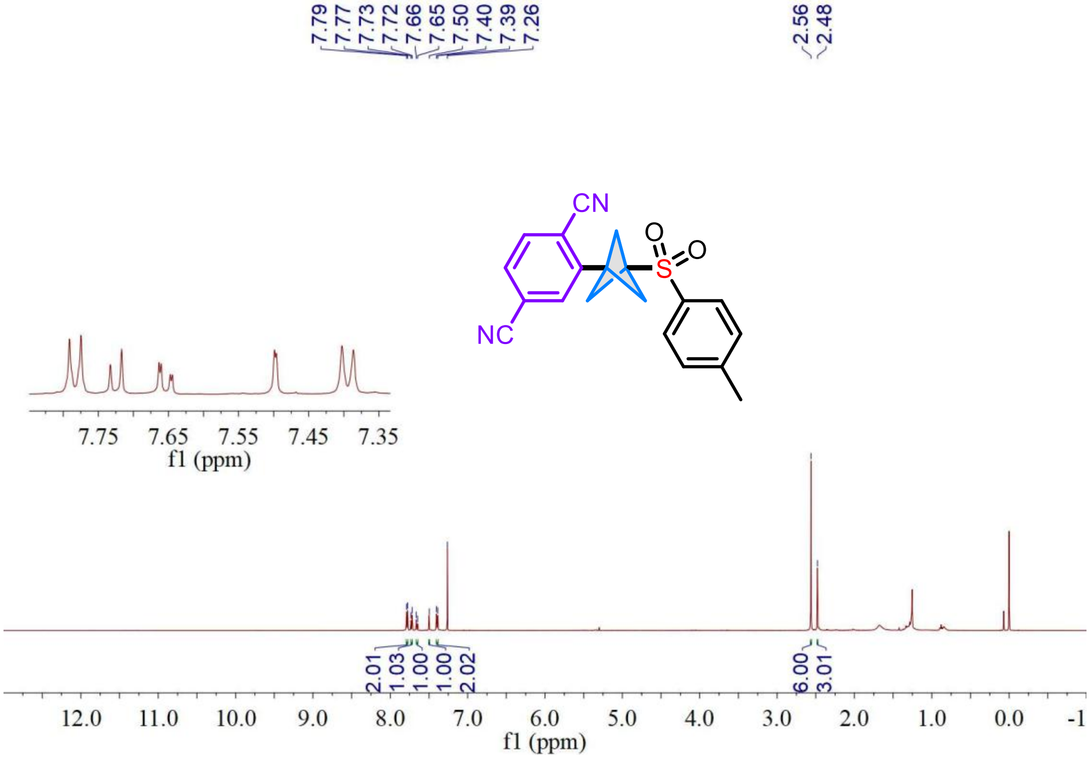

37 ^{13}C NMR (126 MHz, $CDCl_3$)

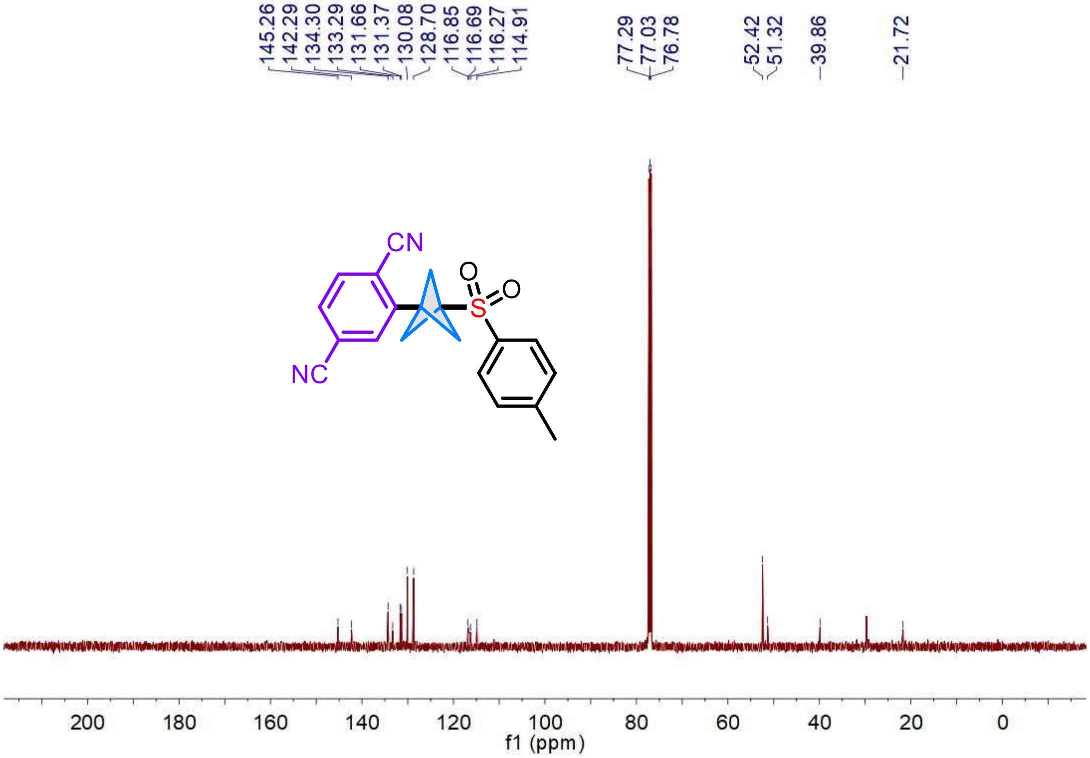

38 ^{1}H NMR (500 MHz, $CDCl_3$)

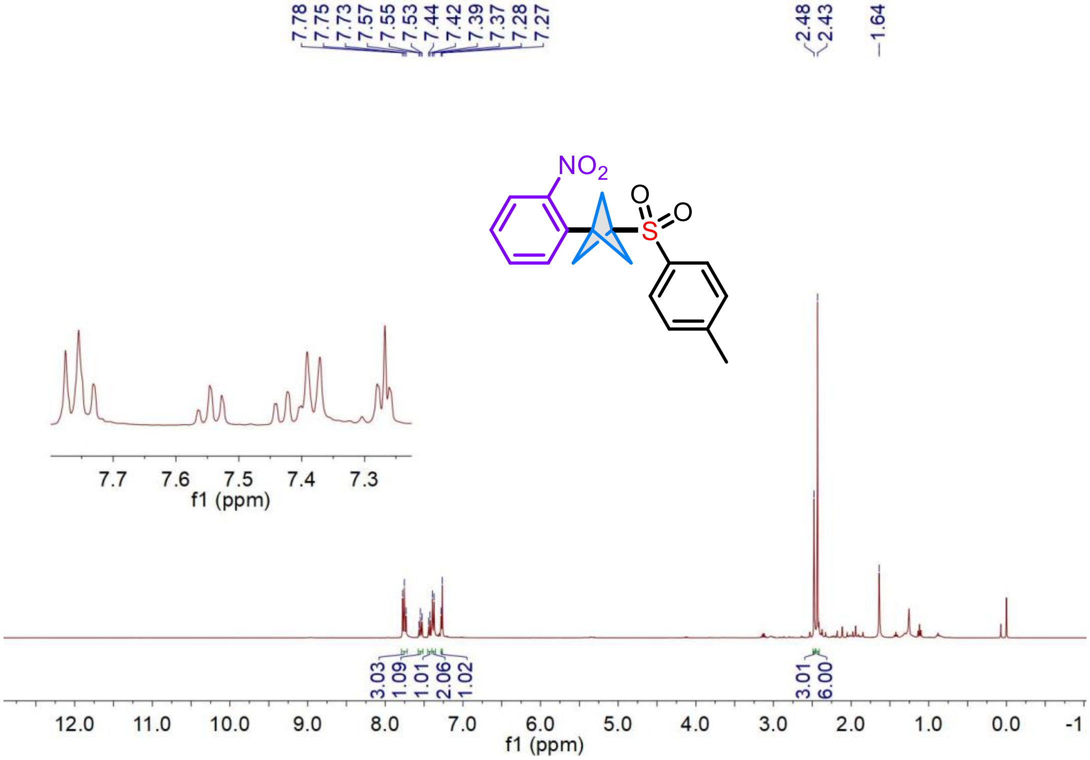

38 ^{13}C NMR (126 MHz, $CDCl_3$)

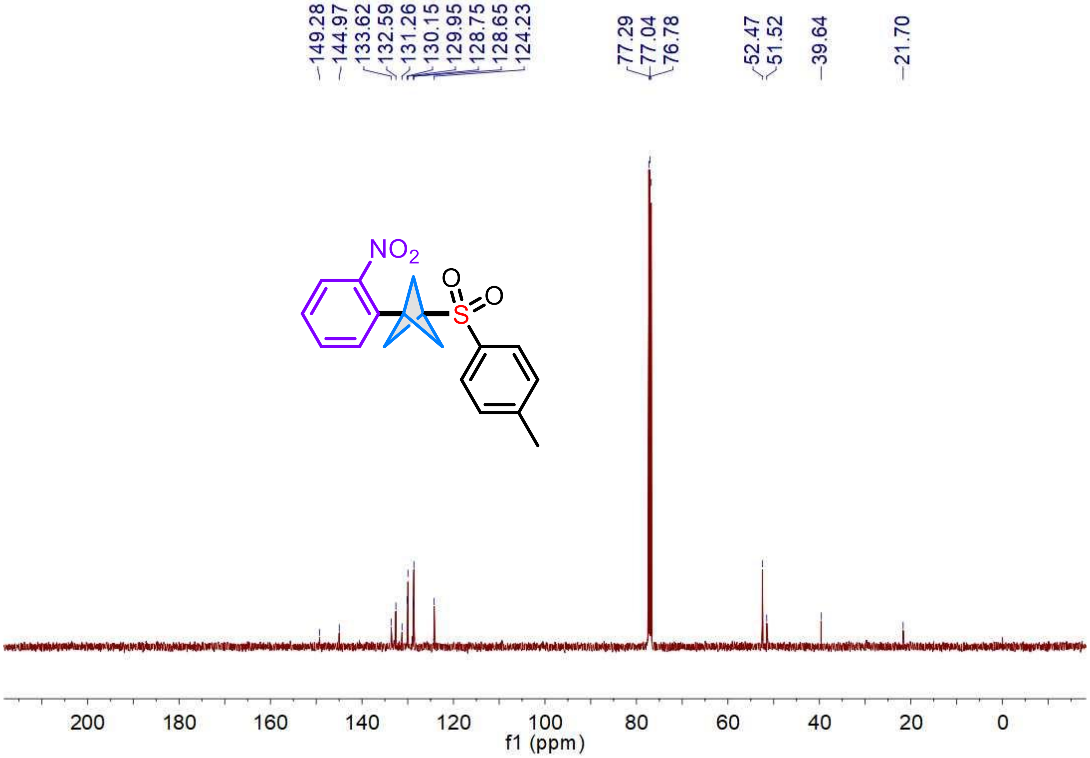

39 ^{1}H NMR (500 MHz, $CDCl_3$)

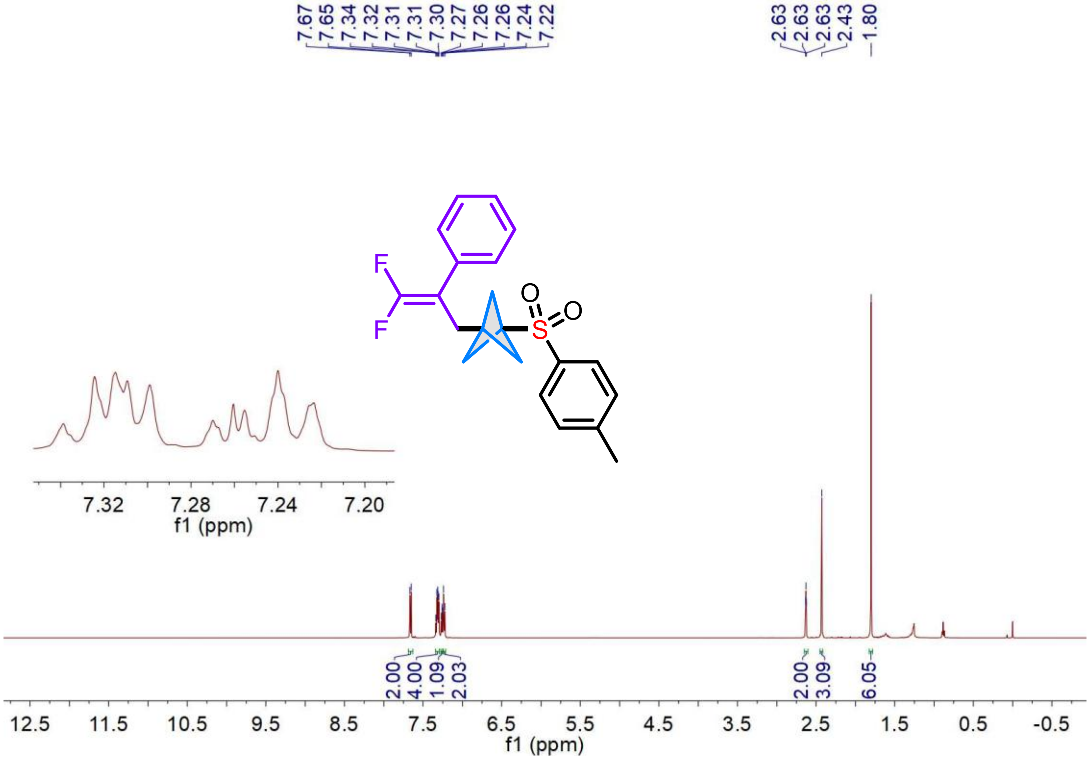

39 ^{13}C NMR (126 MHz, $CDCl_3$)

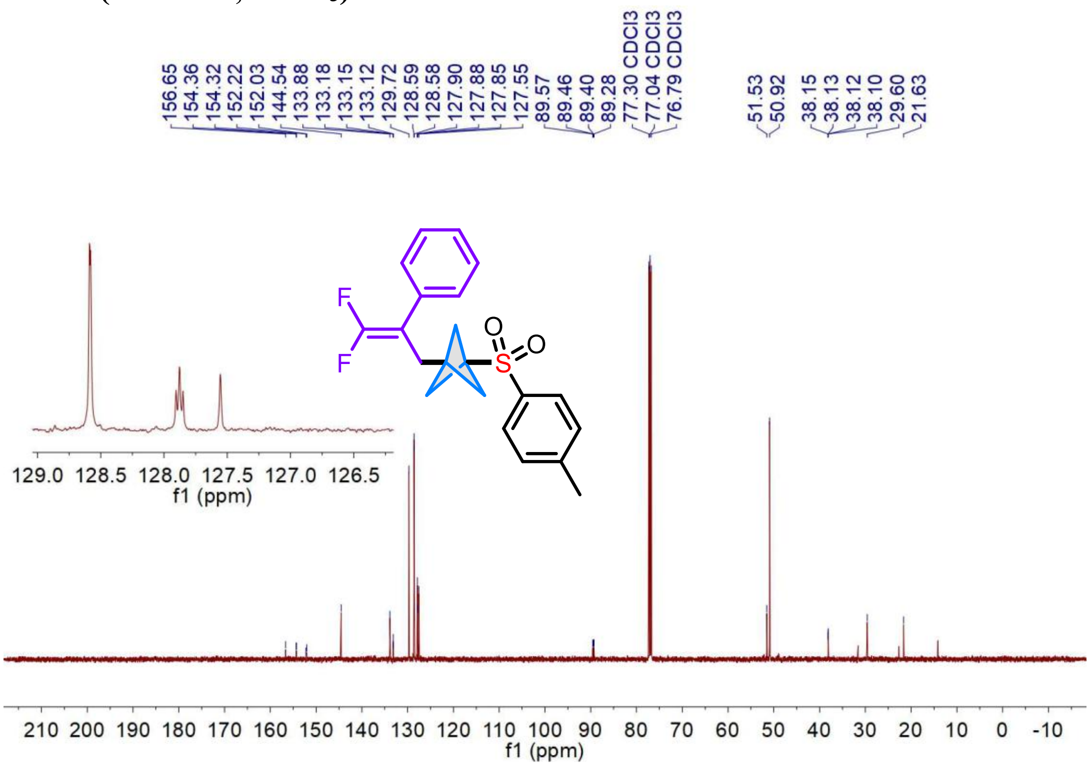

39 ^{19}F NMR (471 MHz, $CDCl_3$)

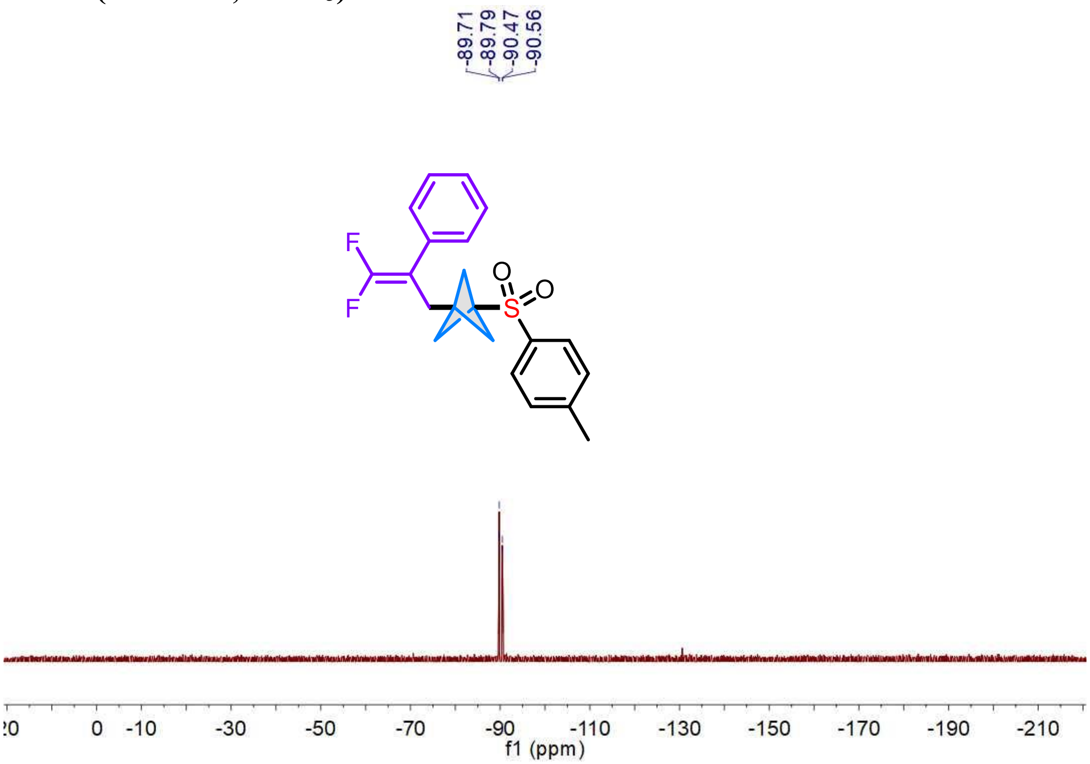

40 ^{1}H NMR (500 MHz, $CDCl_3$)

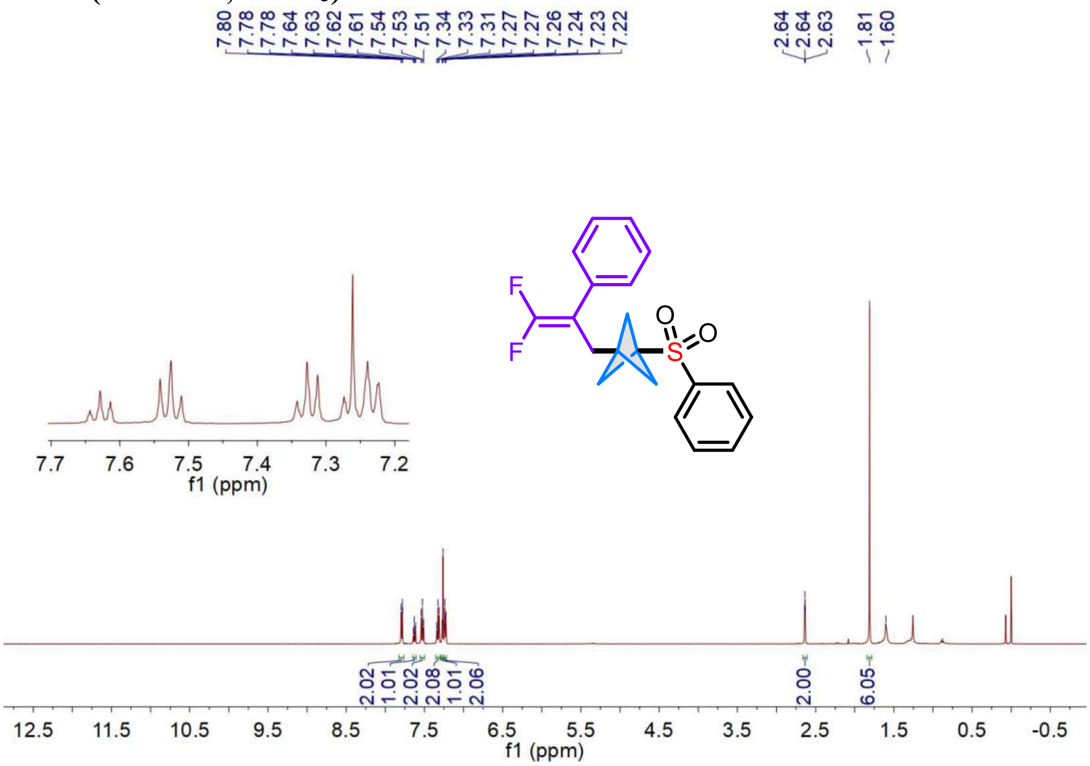

40 ^{13}C NMR (126 MHz, $CDCl_3$)

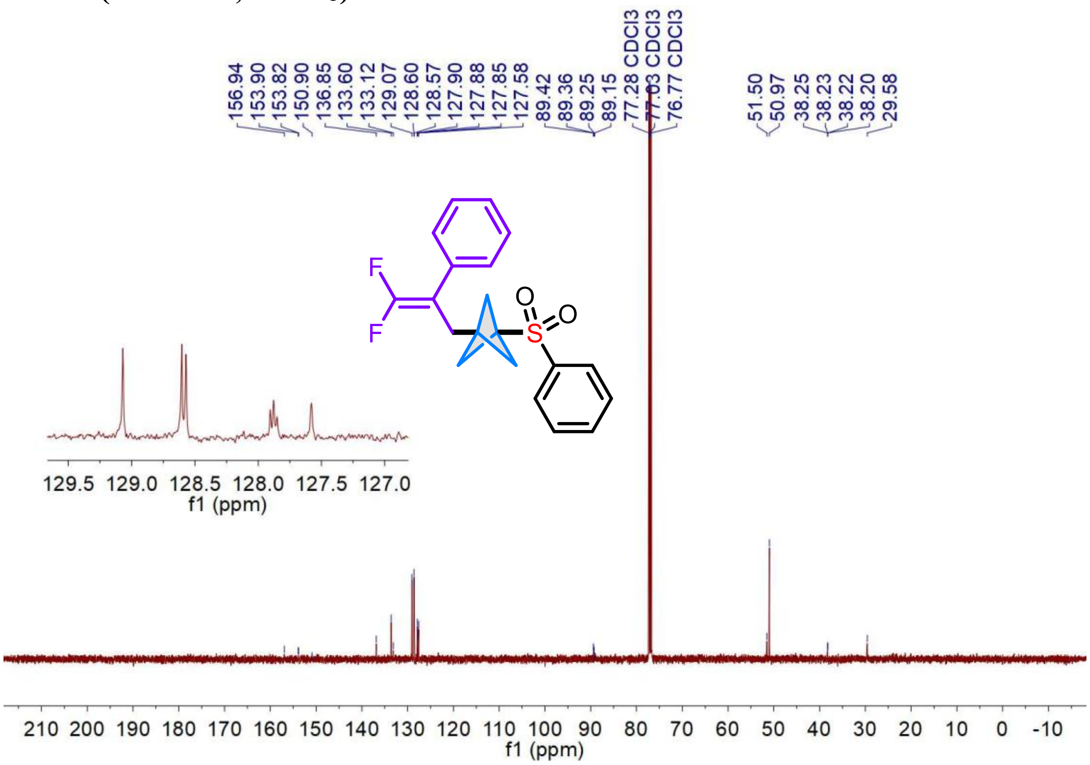

40 ^{19}F NMR (471 MHz, $CDCl_3$)

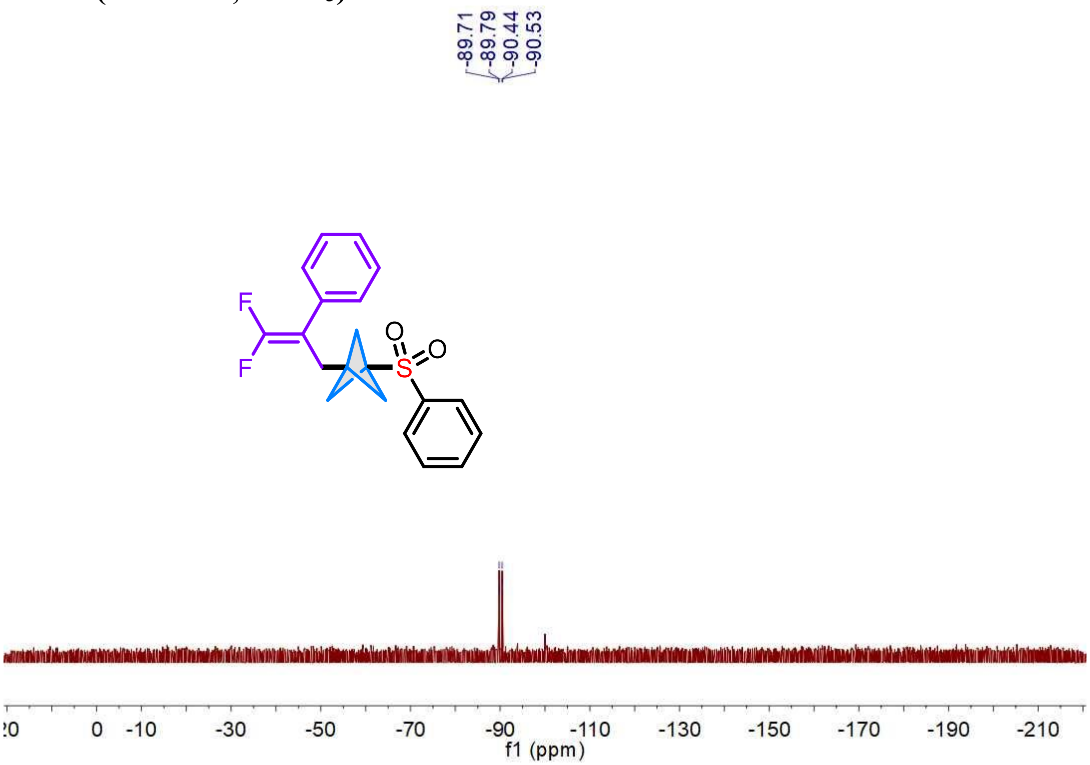

41 ^{1}H NMR (500 MHz, $CDCl_3$)

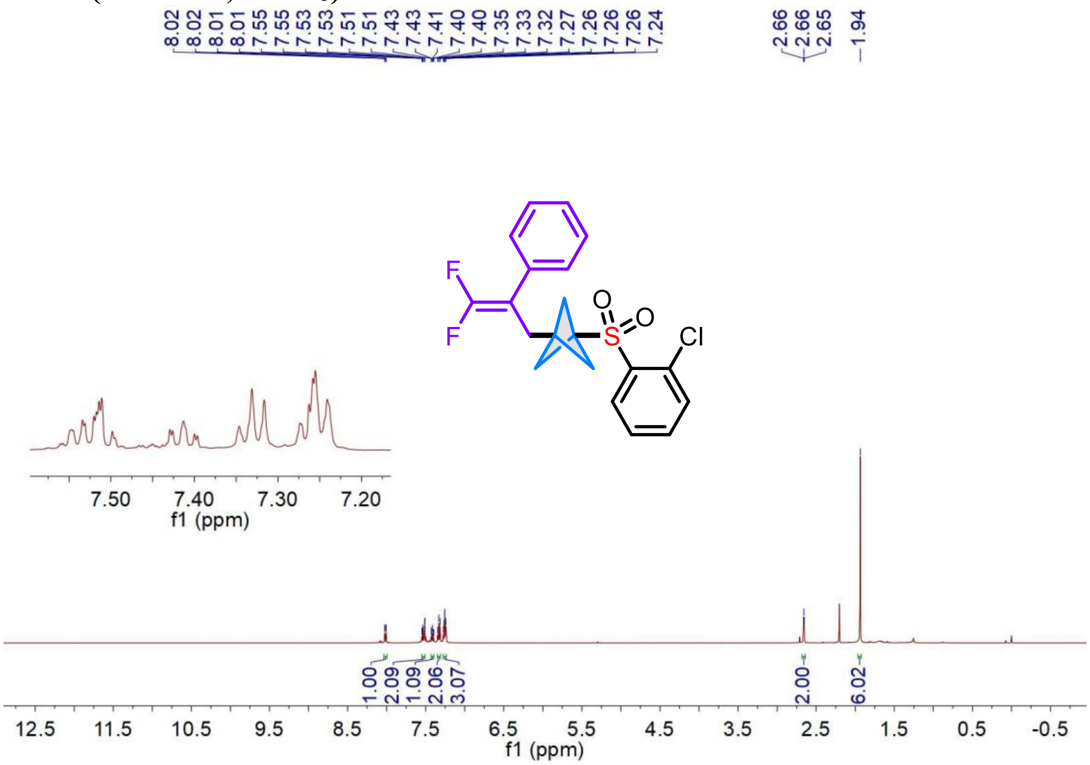

41 ^{13}C NMR (126 MHz, $CDCl_3$)

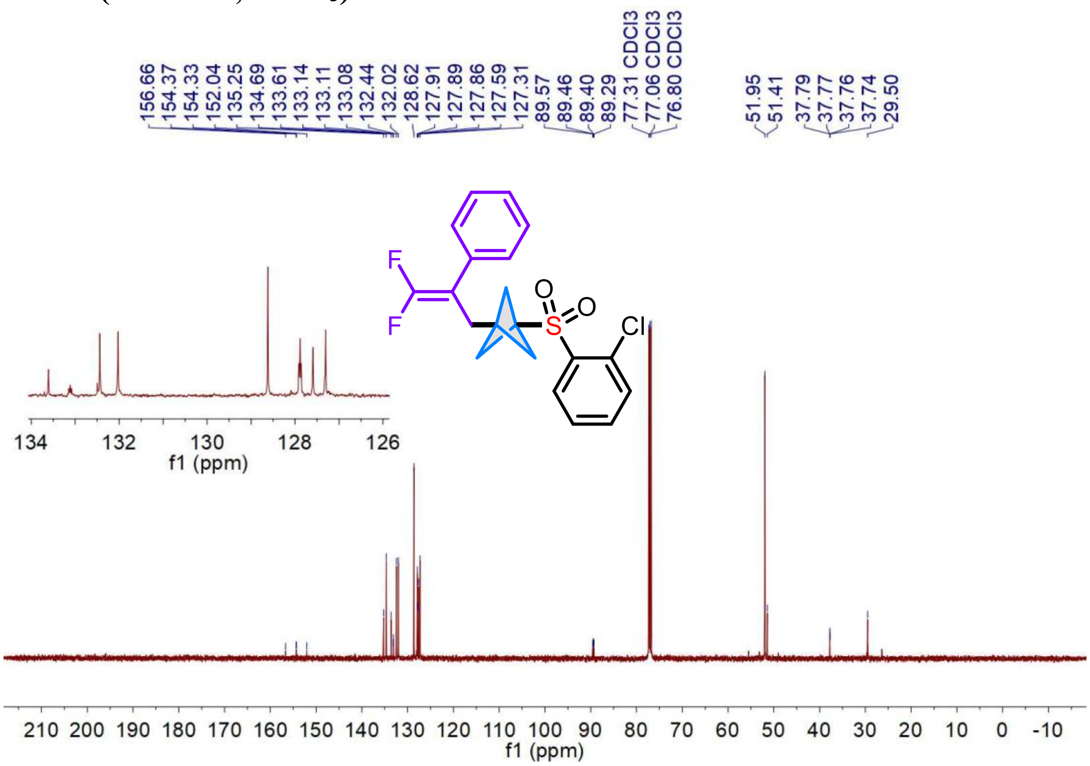

41 ^{19}F NMR (471 MHz, $CDCl_3$)

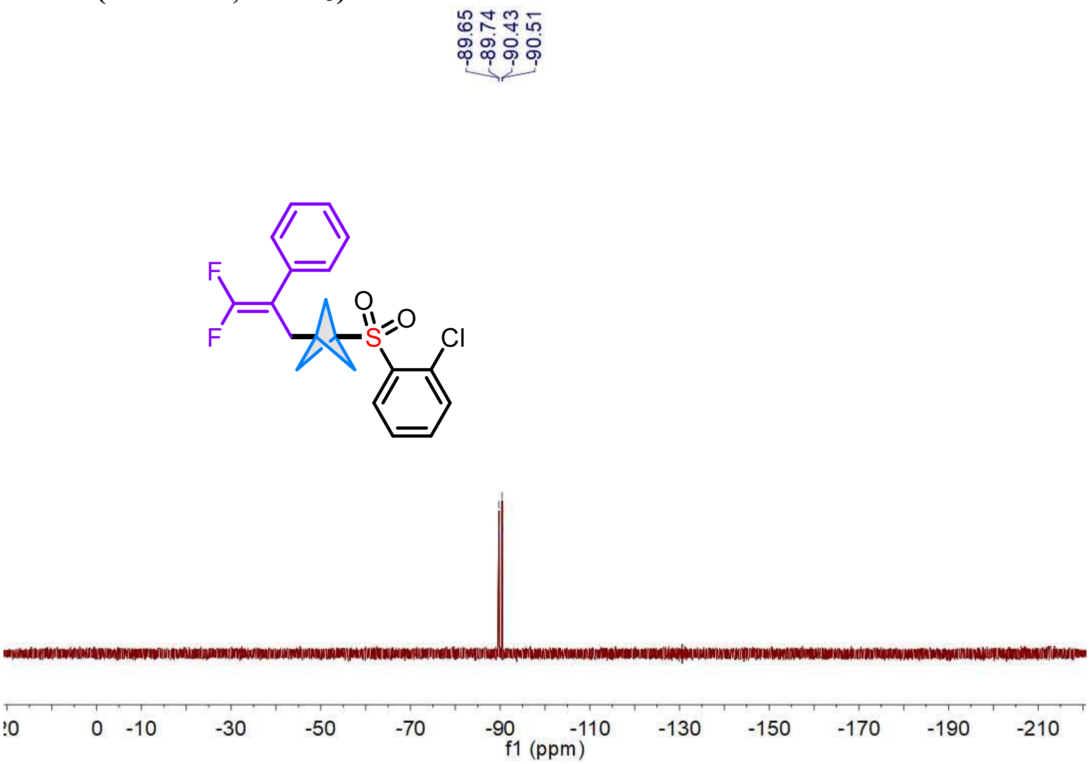

42 ^{1}H NMR (500 MHz, $CDCl_3$)

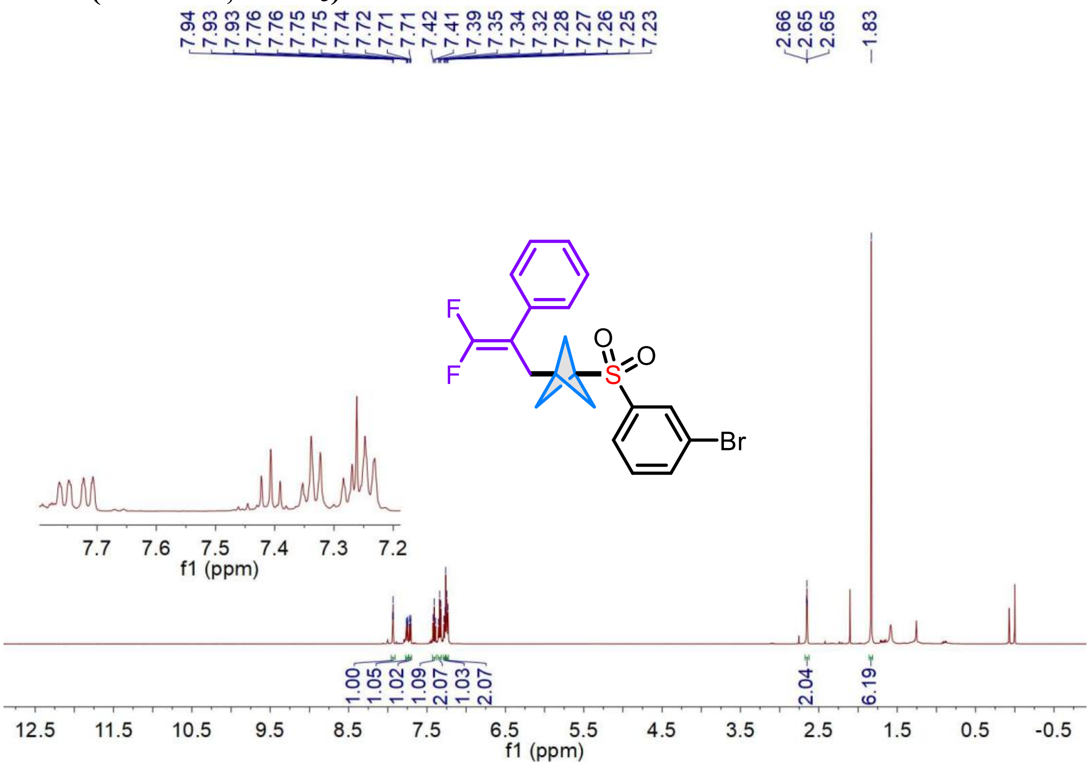

42 ^{13}C NMR (126 MHz, $CDCl_3$)

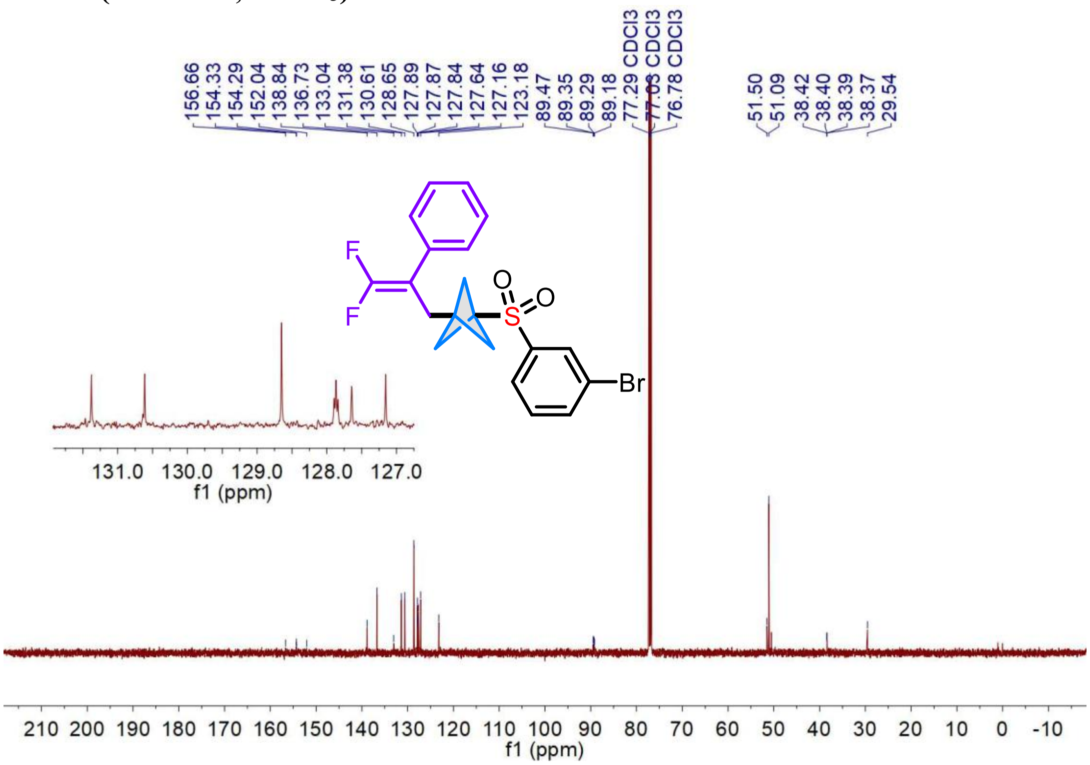

42 ^{19}F NMR (471 MHz, $CDCl_3$)

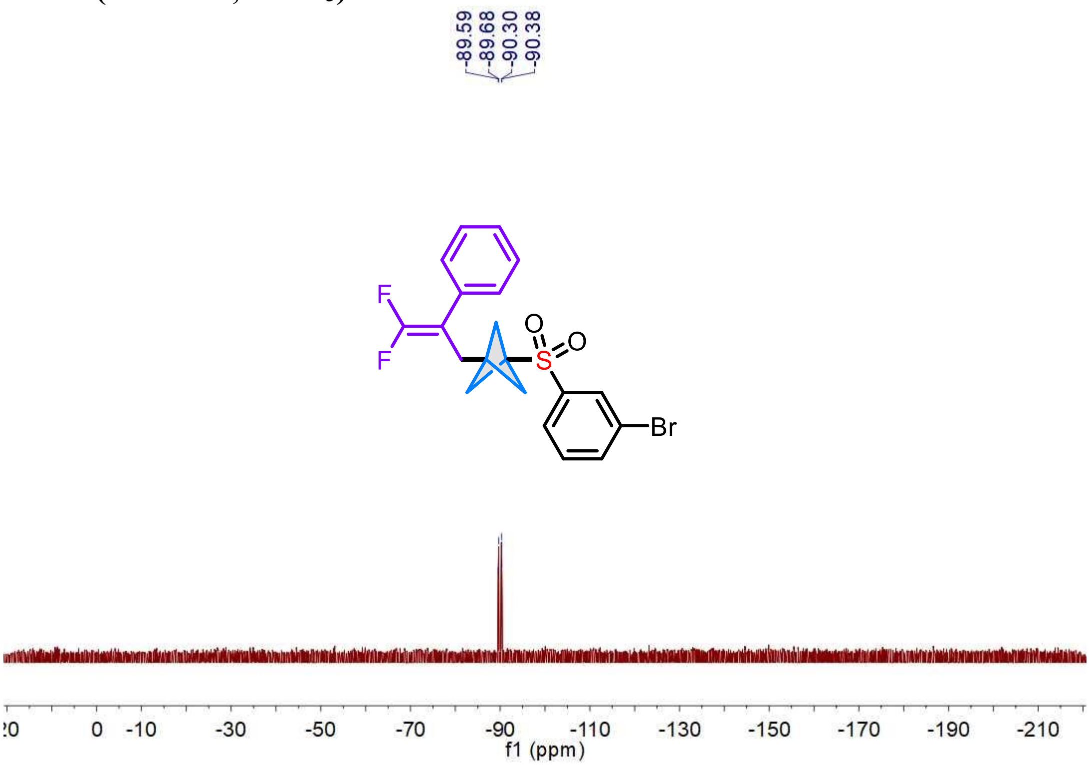

43 ^{1}H NMR (500 MHz, $CDCl_3$)

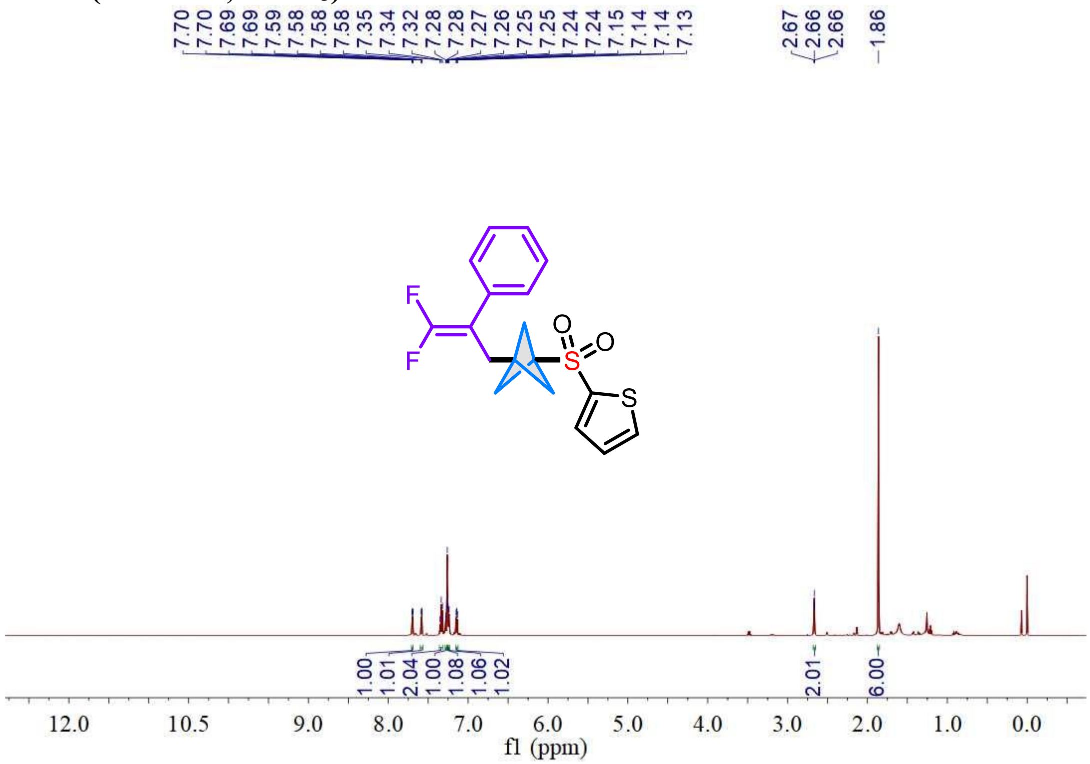

43 ^{13}C NMR (126 MHz, CDCl$_3$)

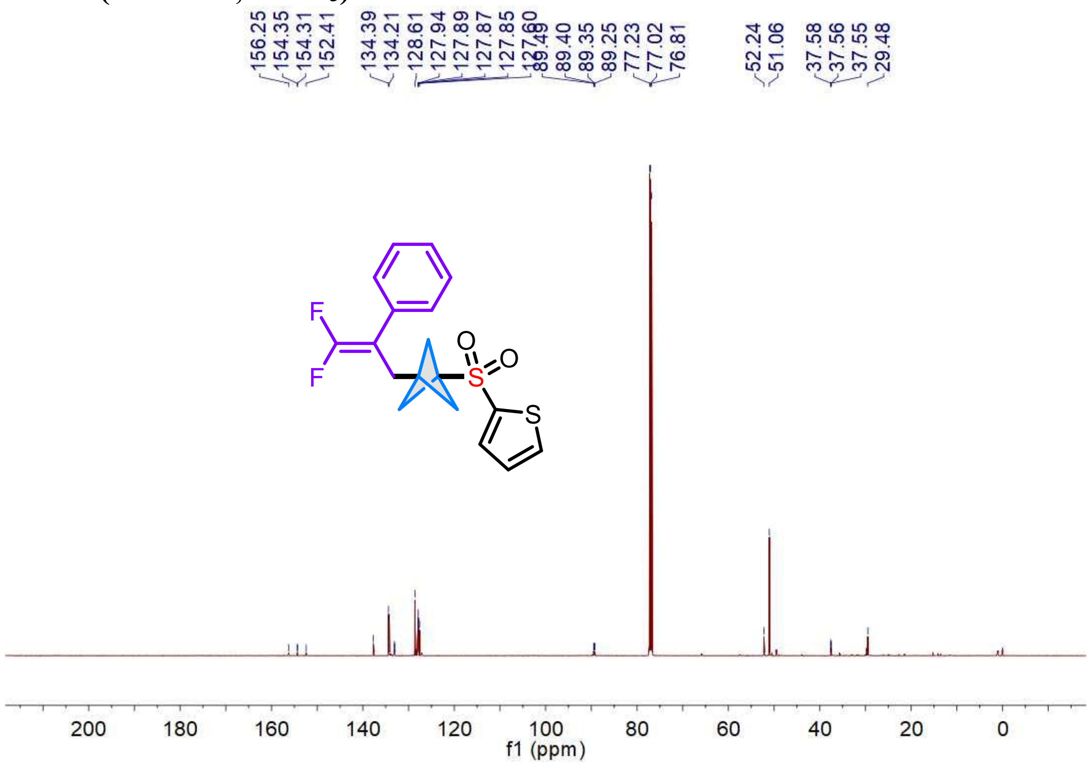

43 ^{19}F NMR (471 MHz, CDCl$_3$)

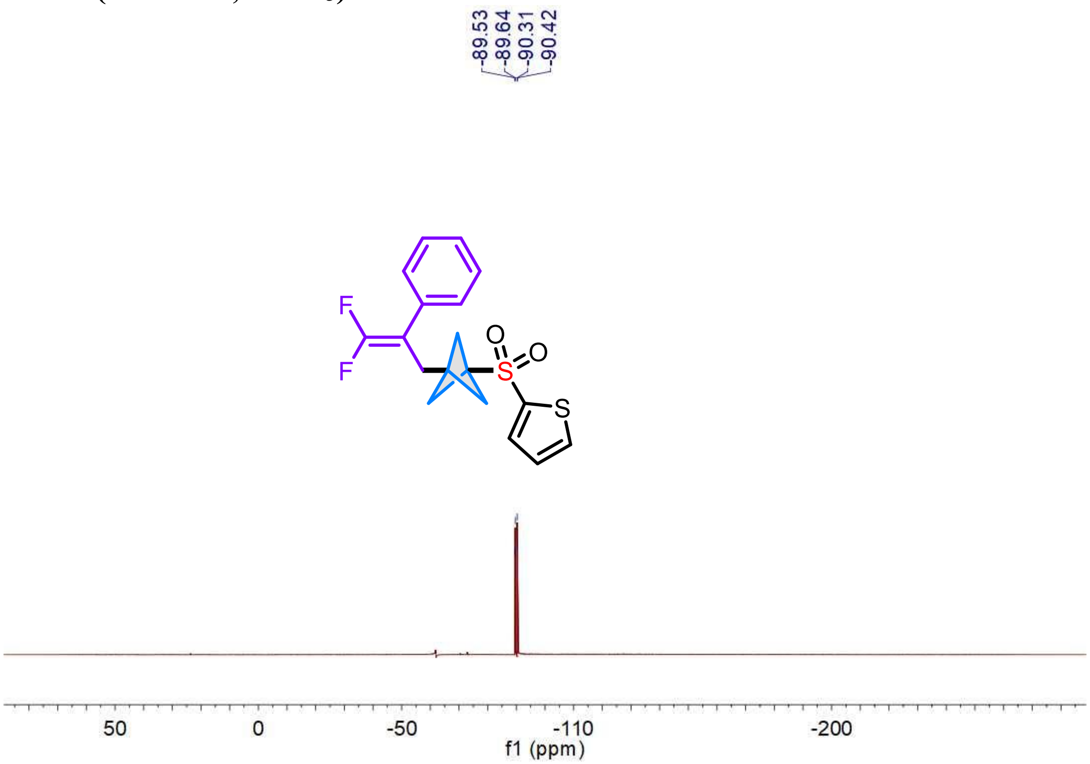

44 ^{1}H NMR (500 MHz, $CDCl_3$)

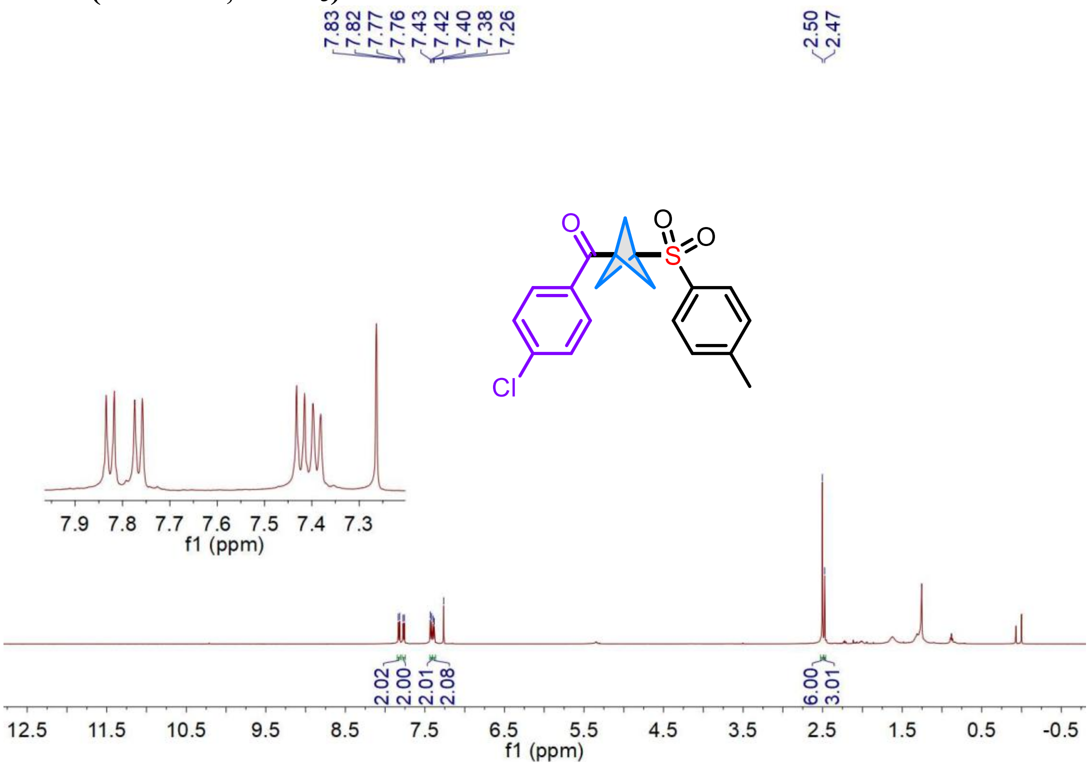

44 ^{13}C NMR (126 MHz, $CDCl_3$)

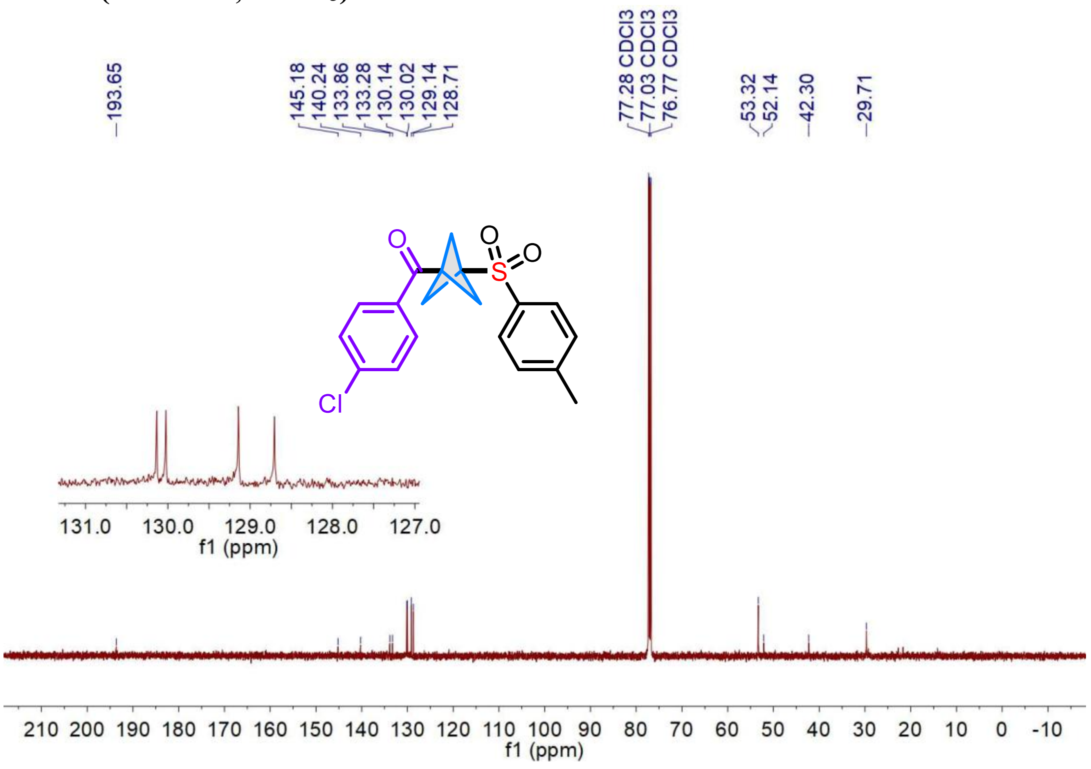

45 ^{1}H NMR (400 MHz, $CDCl_3$)

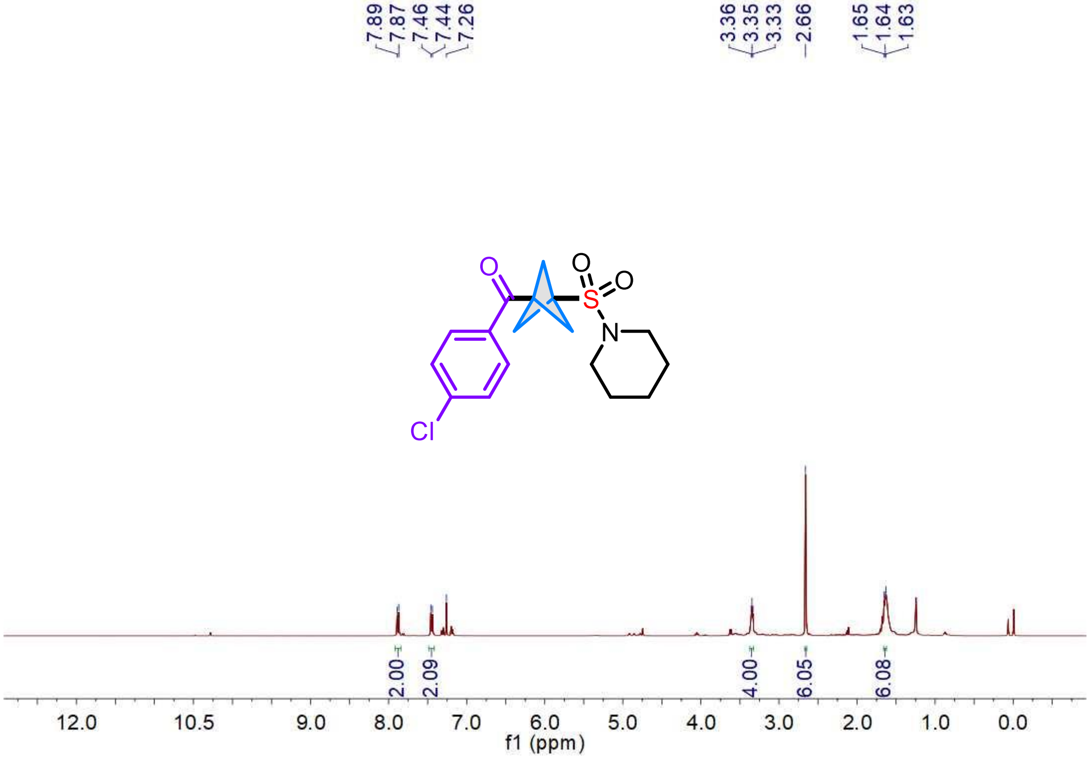

45 ^{13}C NMR (101 MHz, $CDCl_3$)

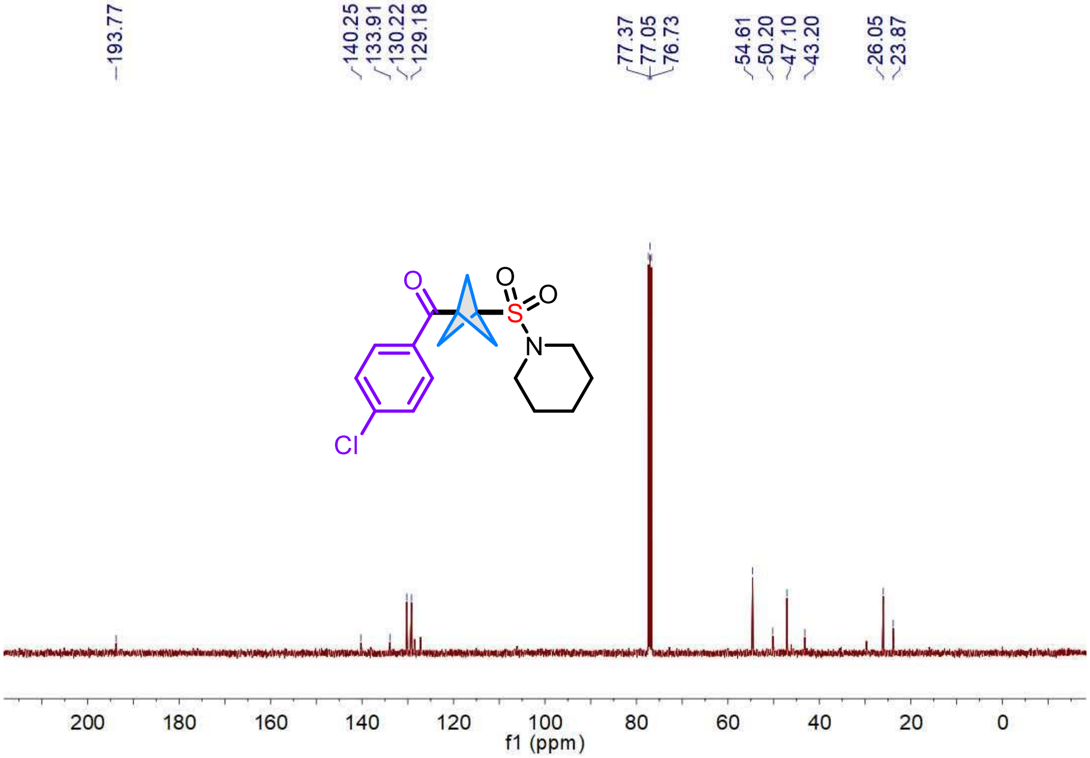

46 ^{1}H NMR (500 MHz, $CDCl_3$)

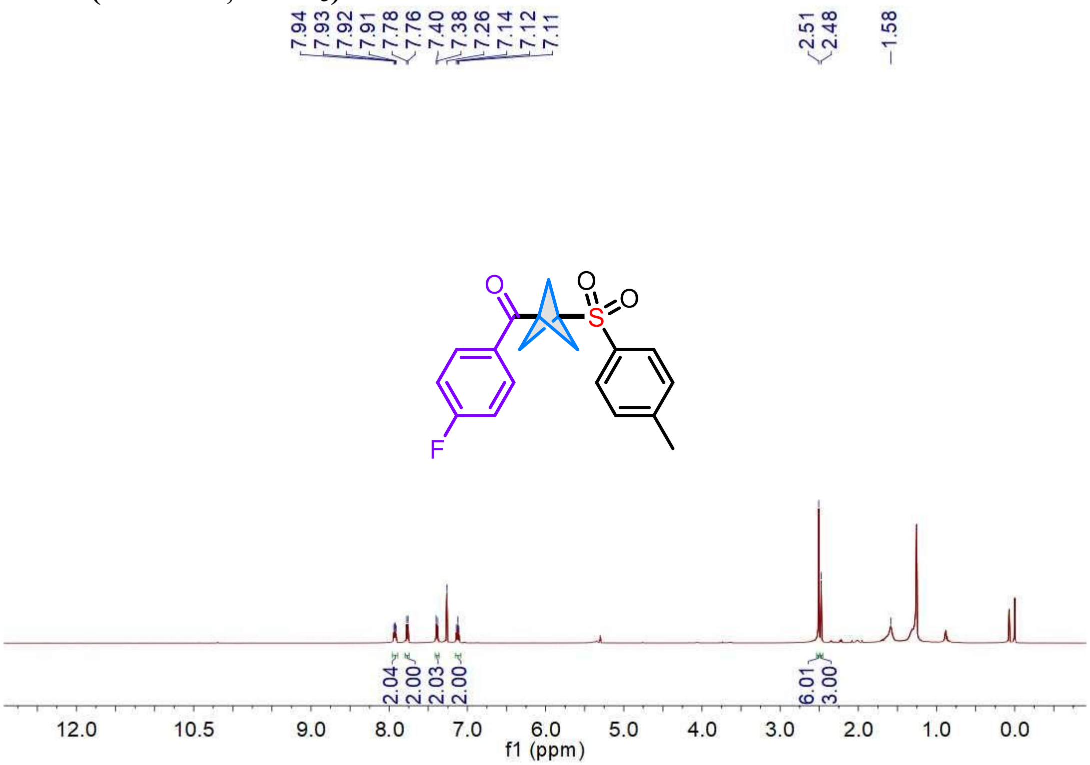

46 ^{13}C NMR (151 MHz, $CDCl_3$)

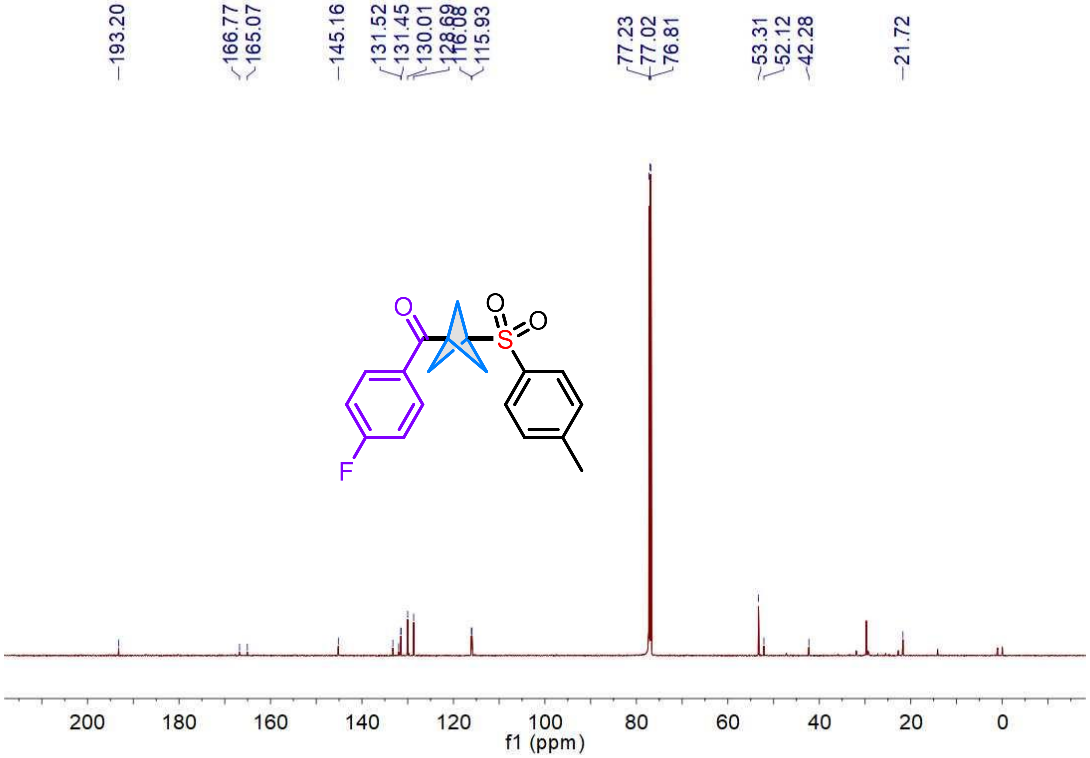

46 ^{19}F NMR (376 MHz, CDCl$_3$)

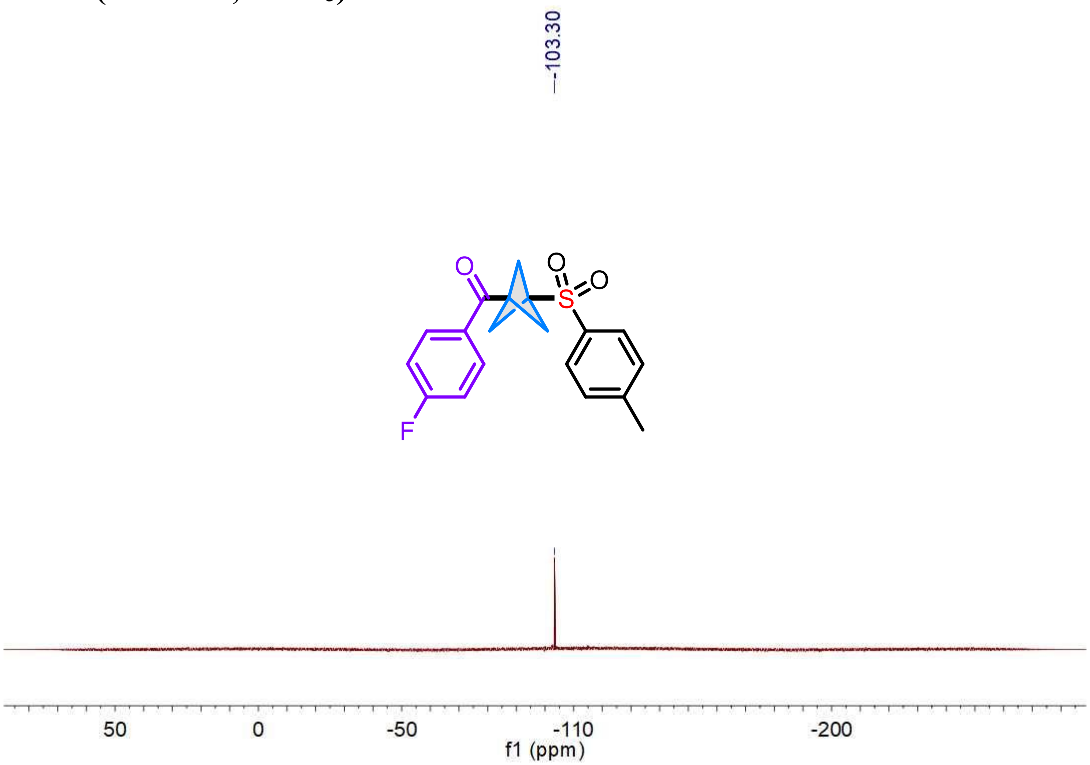

47 ^{1}H NMR (500 MHz, CDCl$_3$)

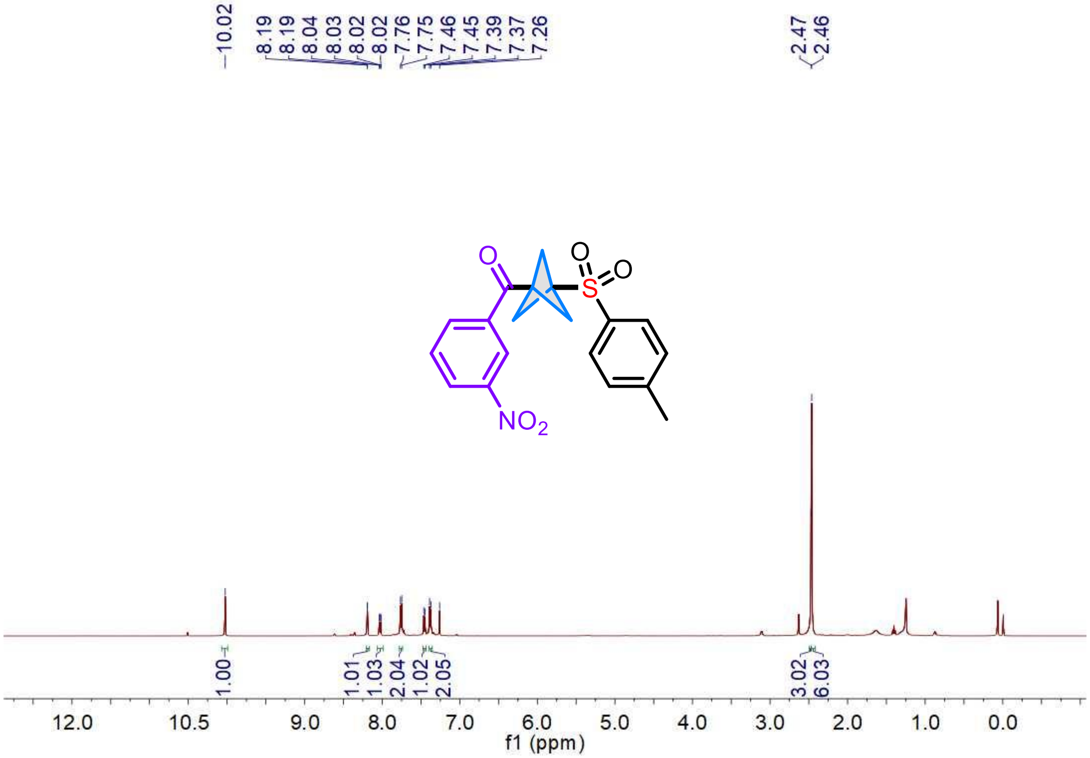

47 ^{13}C NMR (126 MHz, $CDCl_3$)

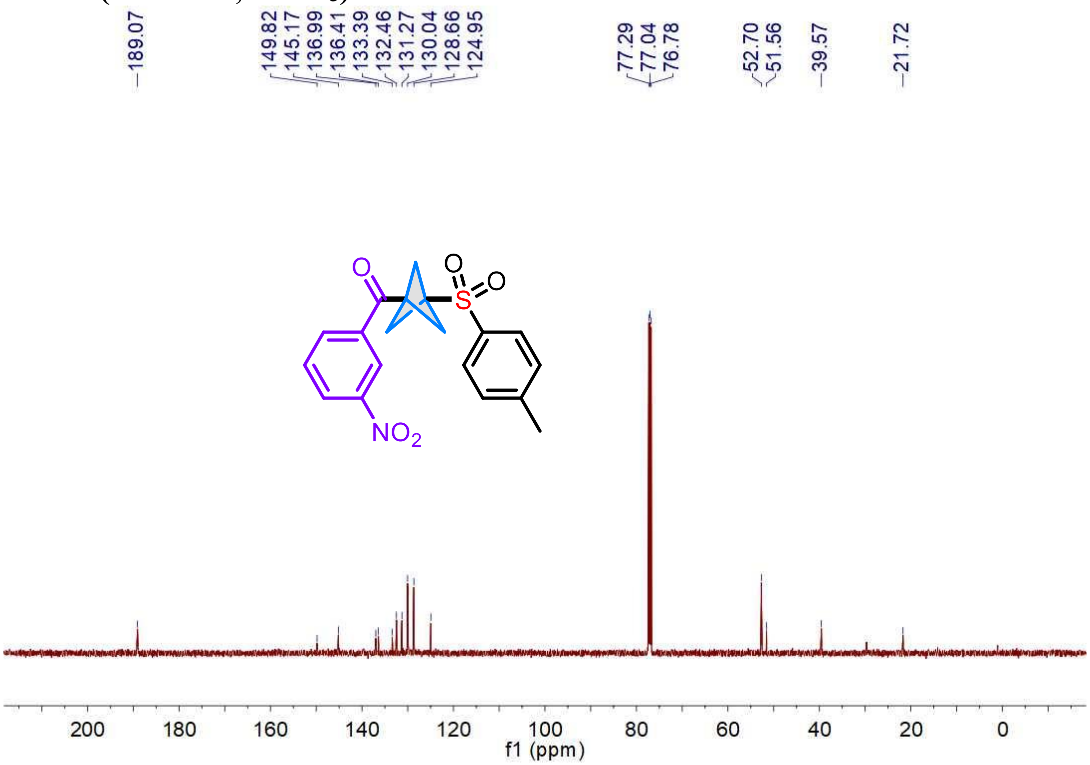

48 ^{1}H NMR (400 MHz, $CDCl_3$)

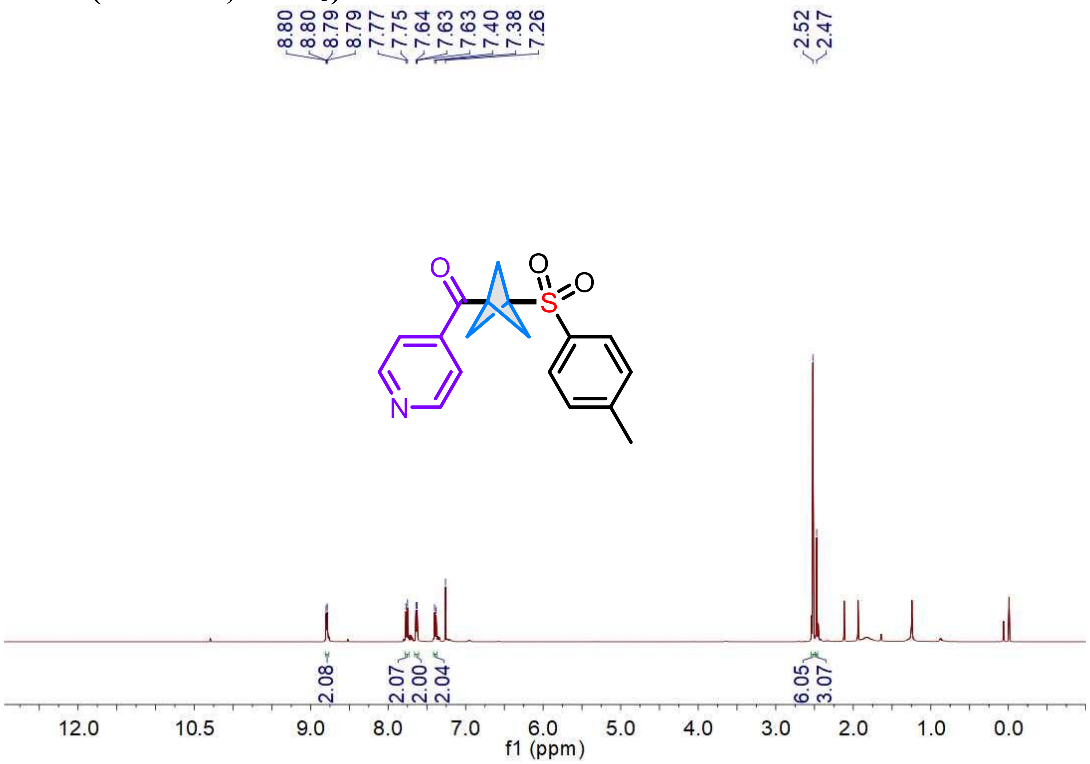

48 ^{13}C NMR (101 MHz, CDCl$_3$)

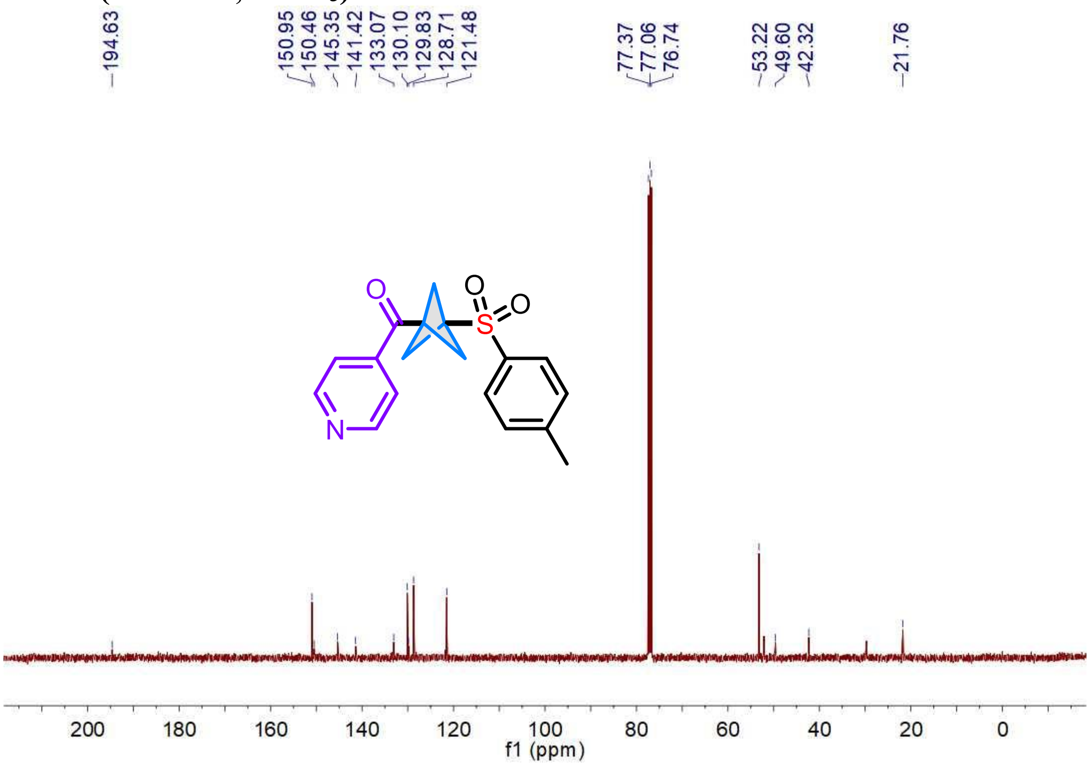

49 ^{1}H NMR (500 MHz, CDCl$_3$)

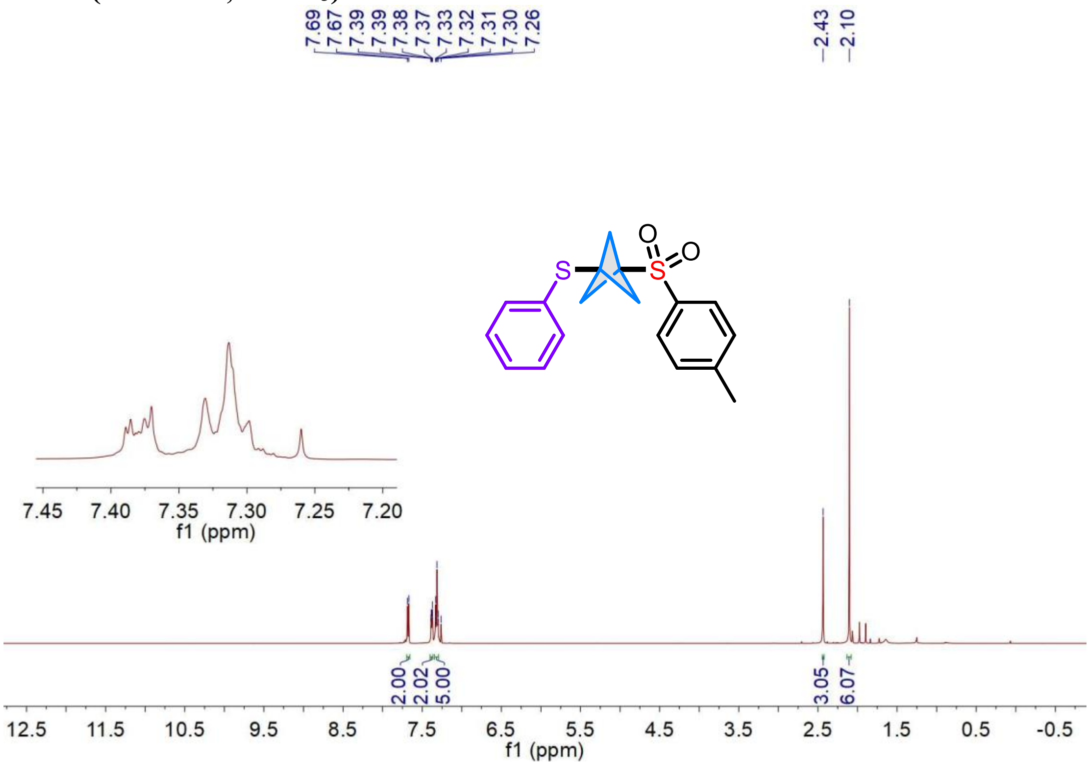

49 ^{13}C NMR (126 MHz, CDCl$_3$)

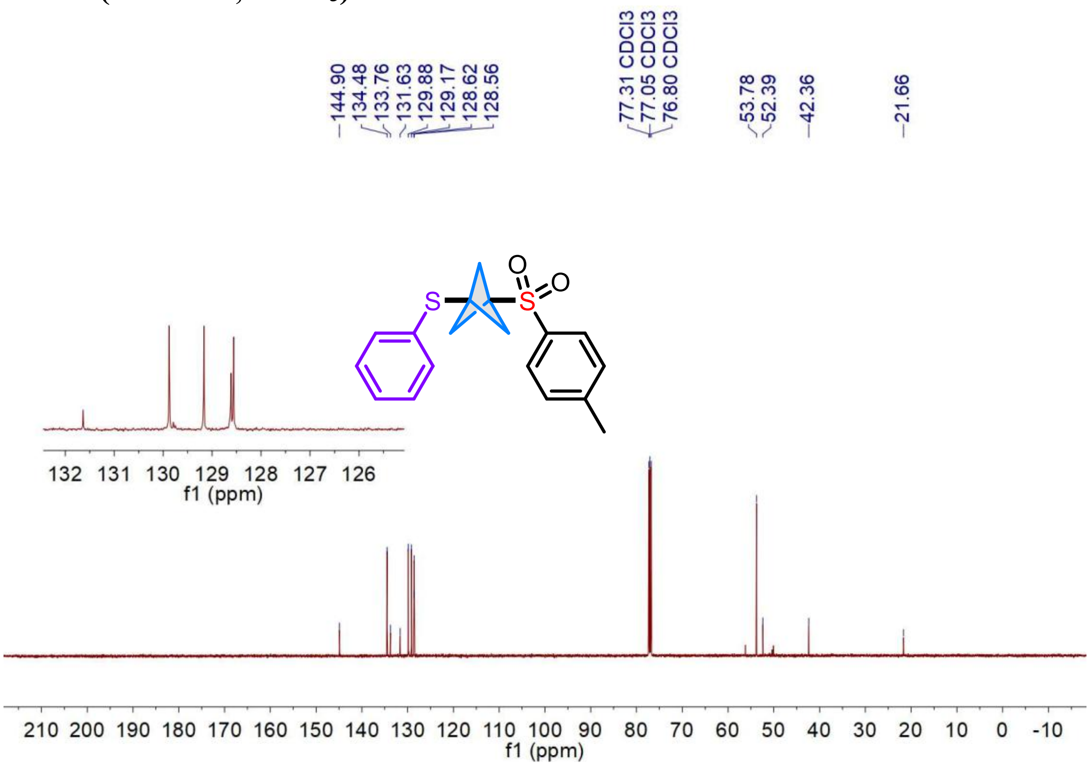

50 ^{1}H NMR (500 MHz, CDCl$_3$)

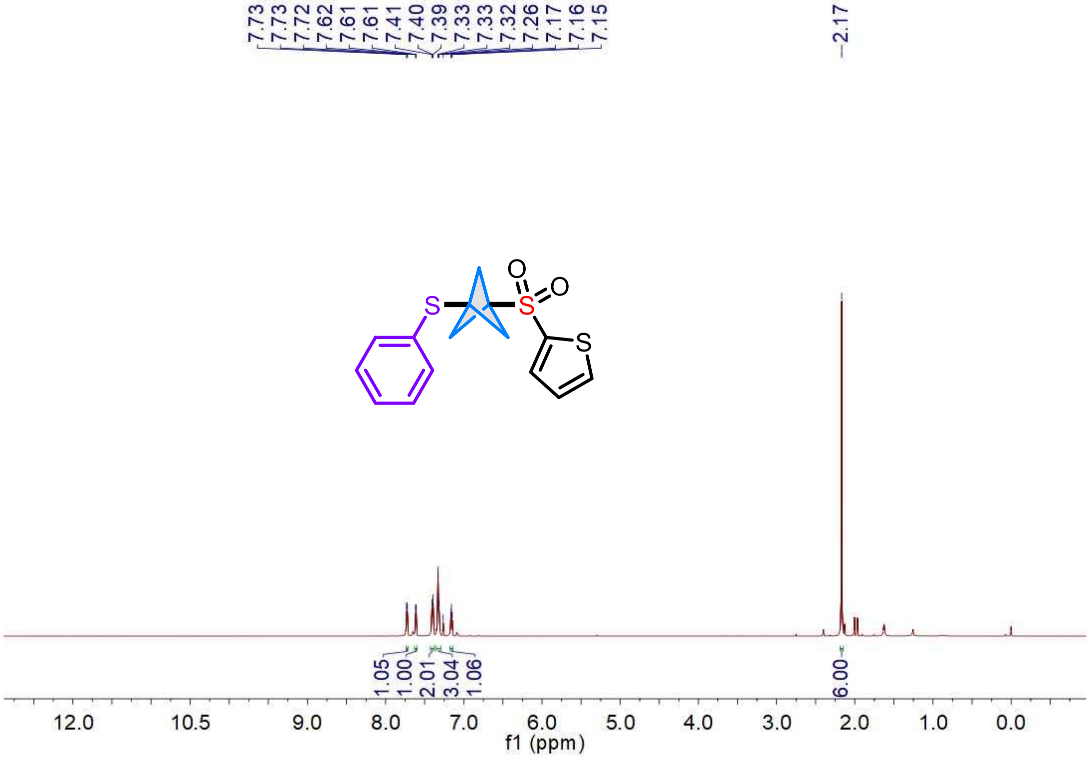

50 ^{13}C NMR (126 MHz, $CDCl_3$)

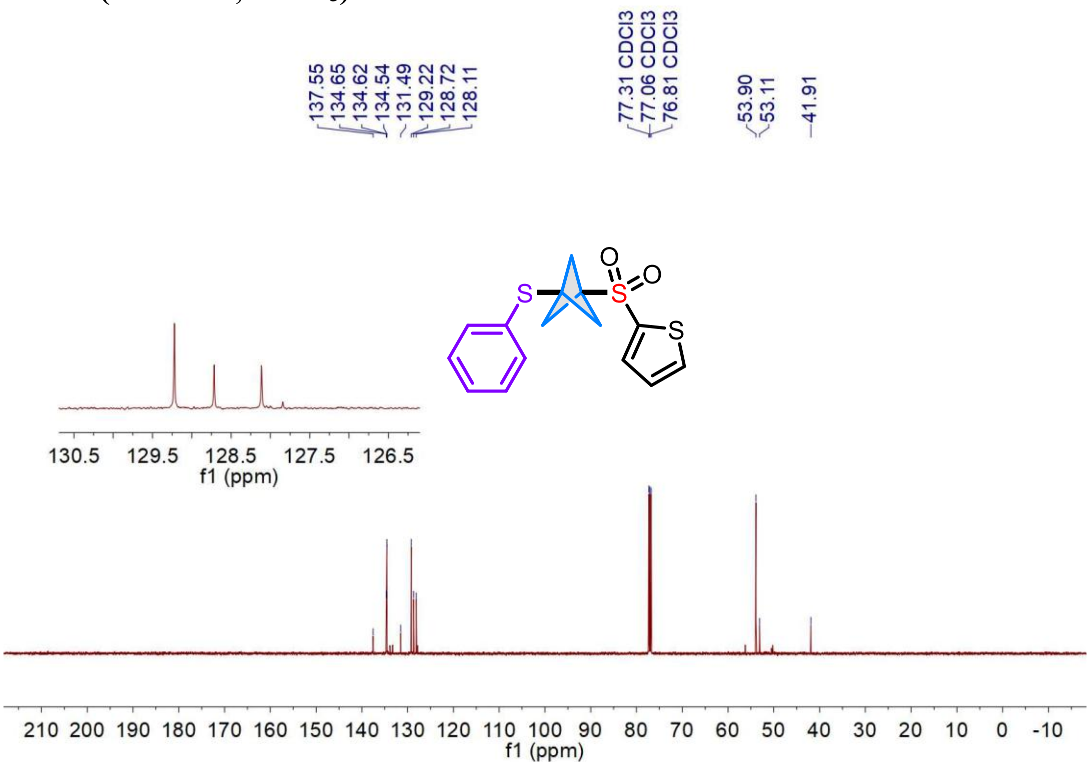

51 ^{1}H NMR (500 MHz, $CDCl_3$)

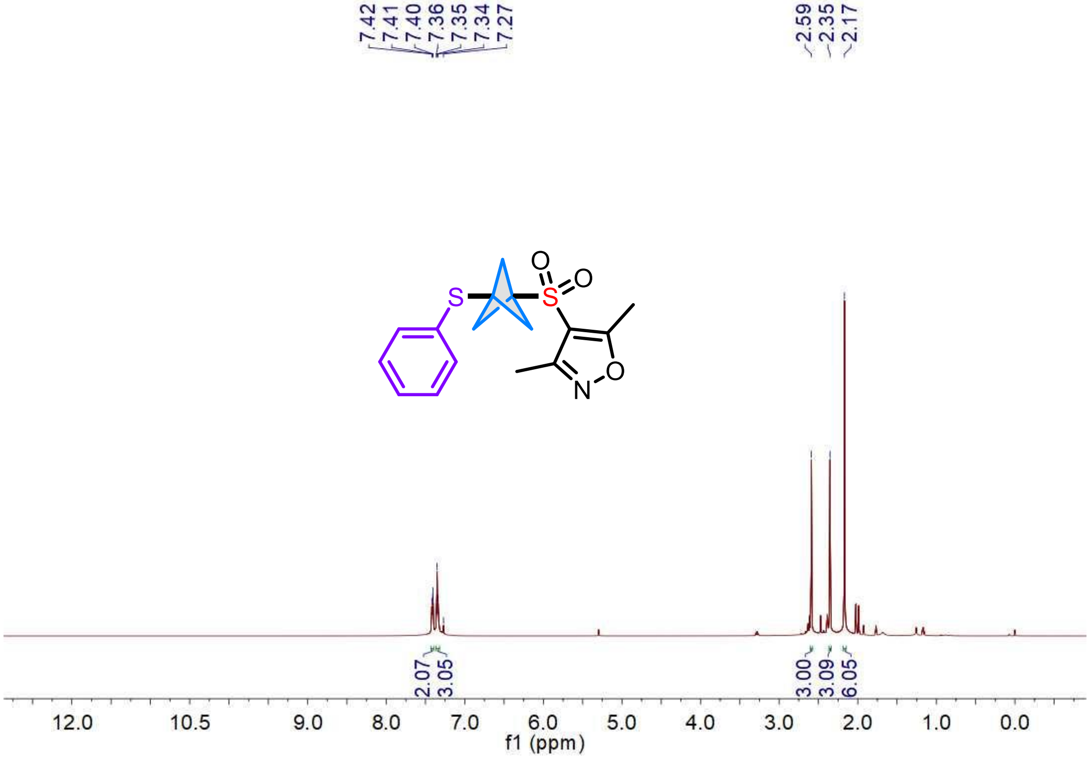

51 ^{13}C NMR (126 MHz, $CDCl_3$)

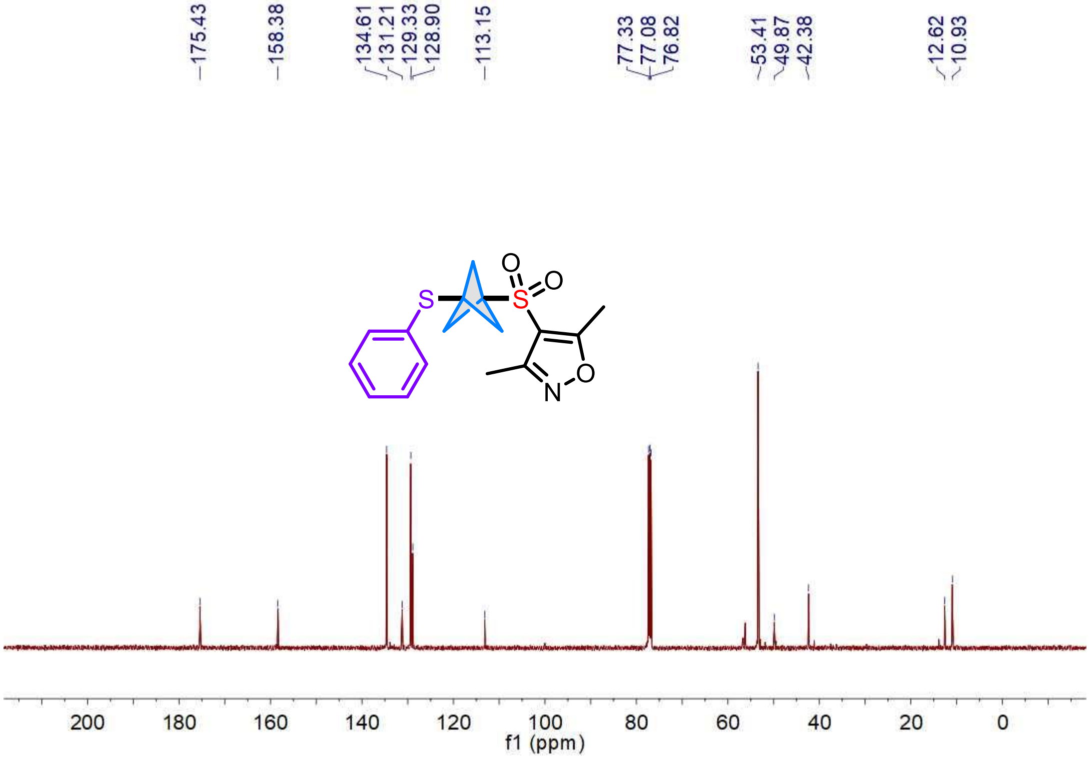

52 ^{1}H NMR (500 MHz, $CDCl_3$)

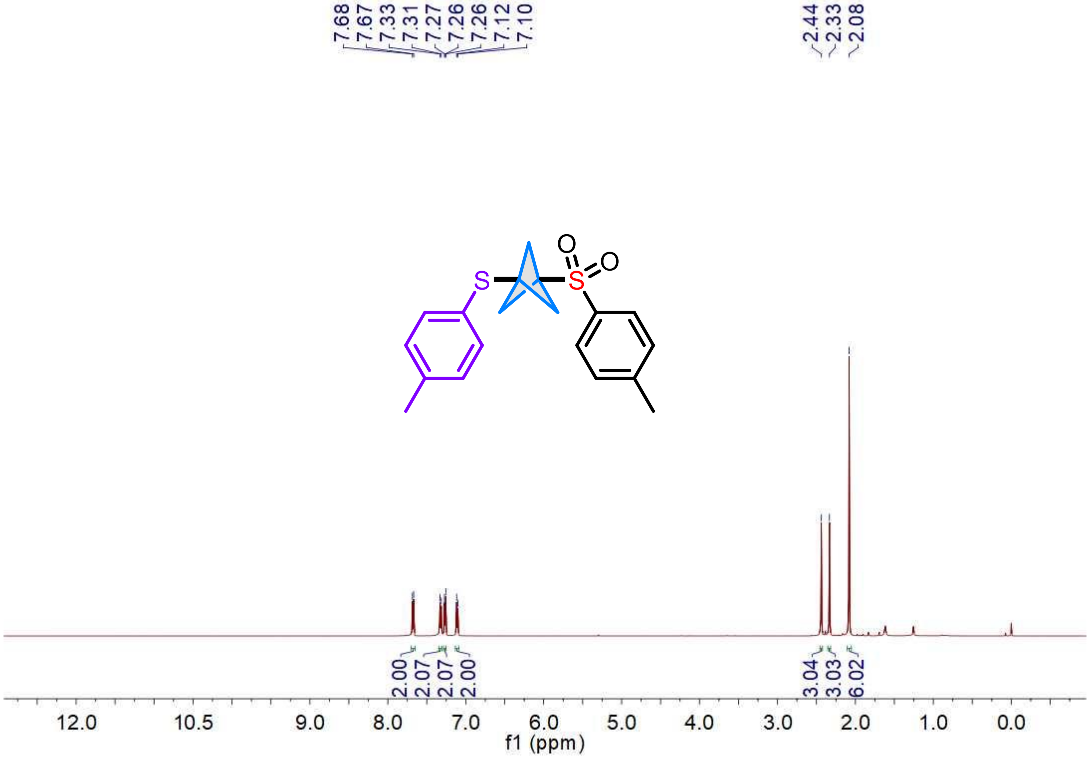

52 ^{13}C NMR (126 MHz, $CDCl_3$)

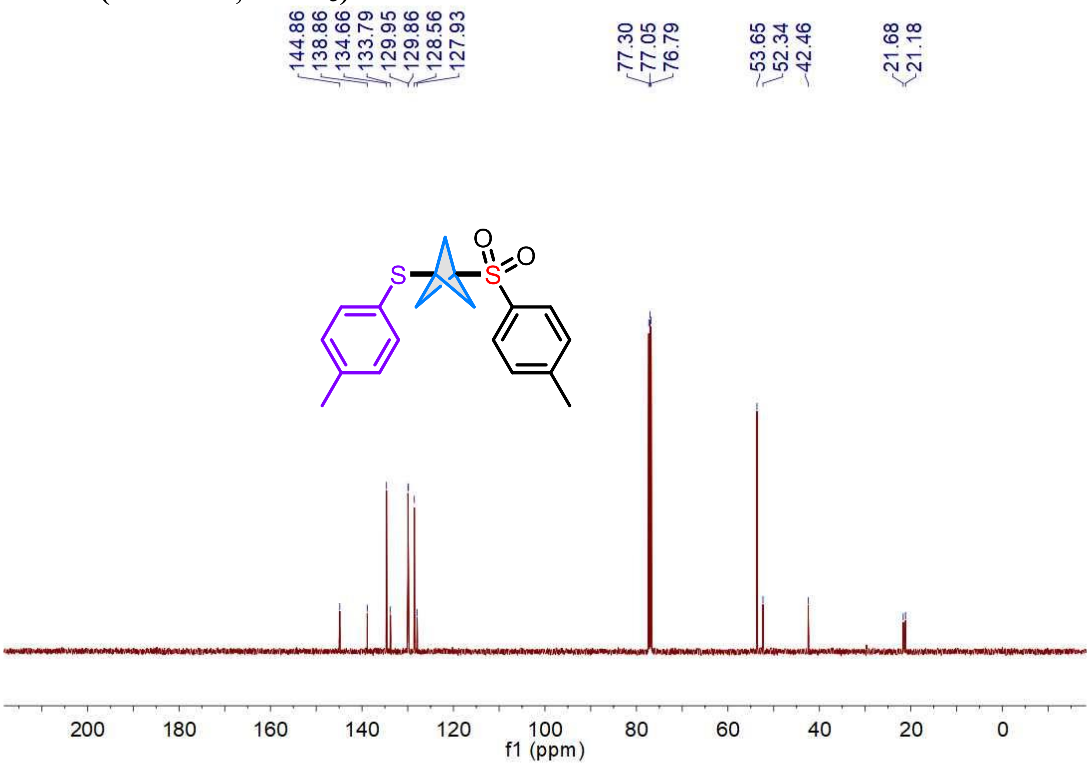

53 ^{1}H NMR (500 MHz, $CDCl_3$)

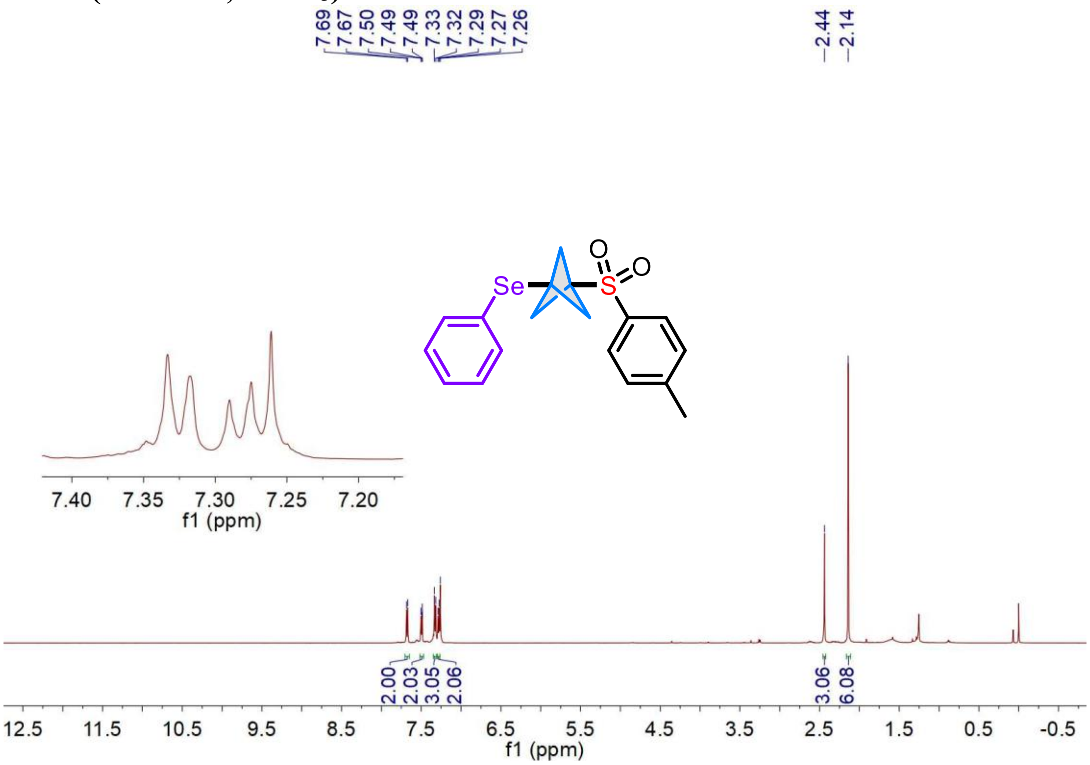

53 ^{13}C NMR (126 MHz, $CDCl_3$)

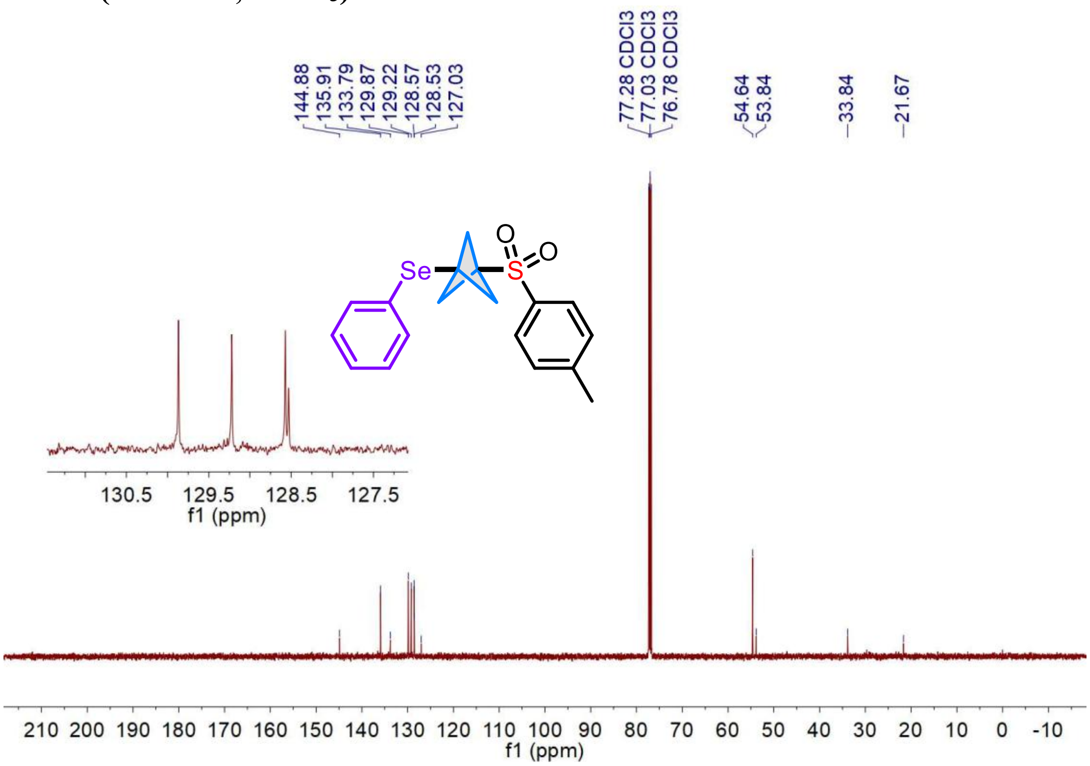

54 ^{1}H NMR (500 MHz, $CDCl_3$)

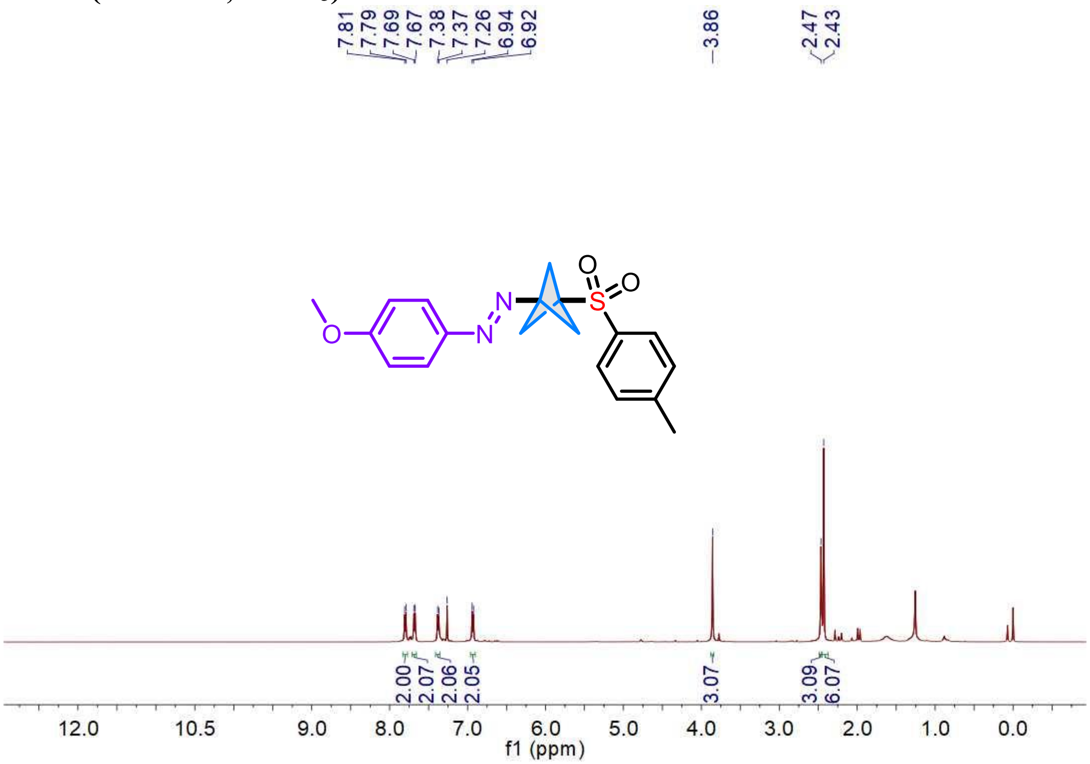

54 ^{13}C NMR (126 MHz, CDCl$_3$)

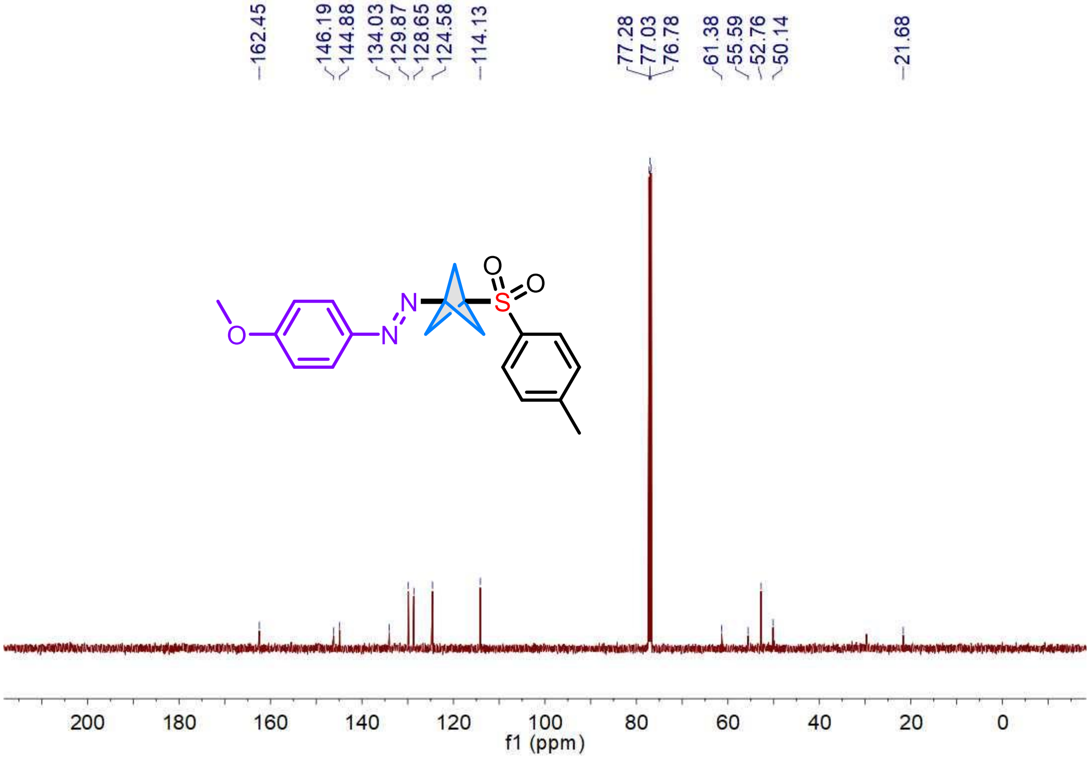

55 ^{1}H NMR (400 MHz, CDCl$_3$)

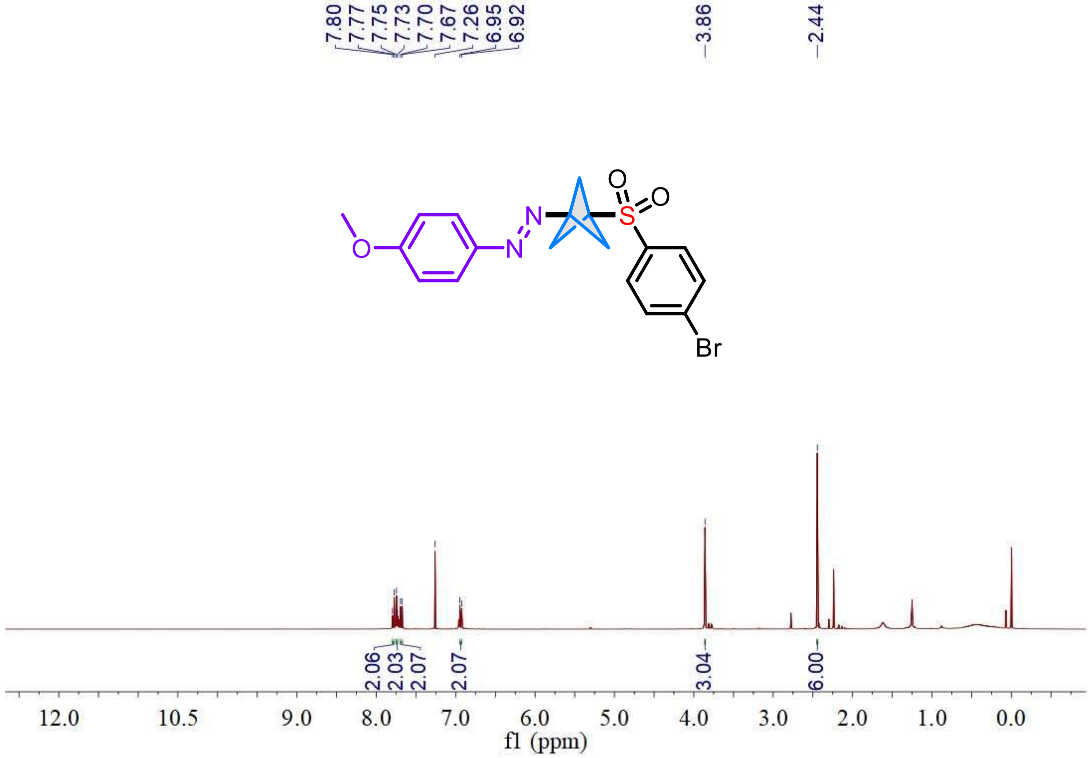

55 ^{13}C NMR (101 MHz, $CDCl_3$)

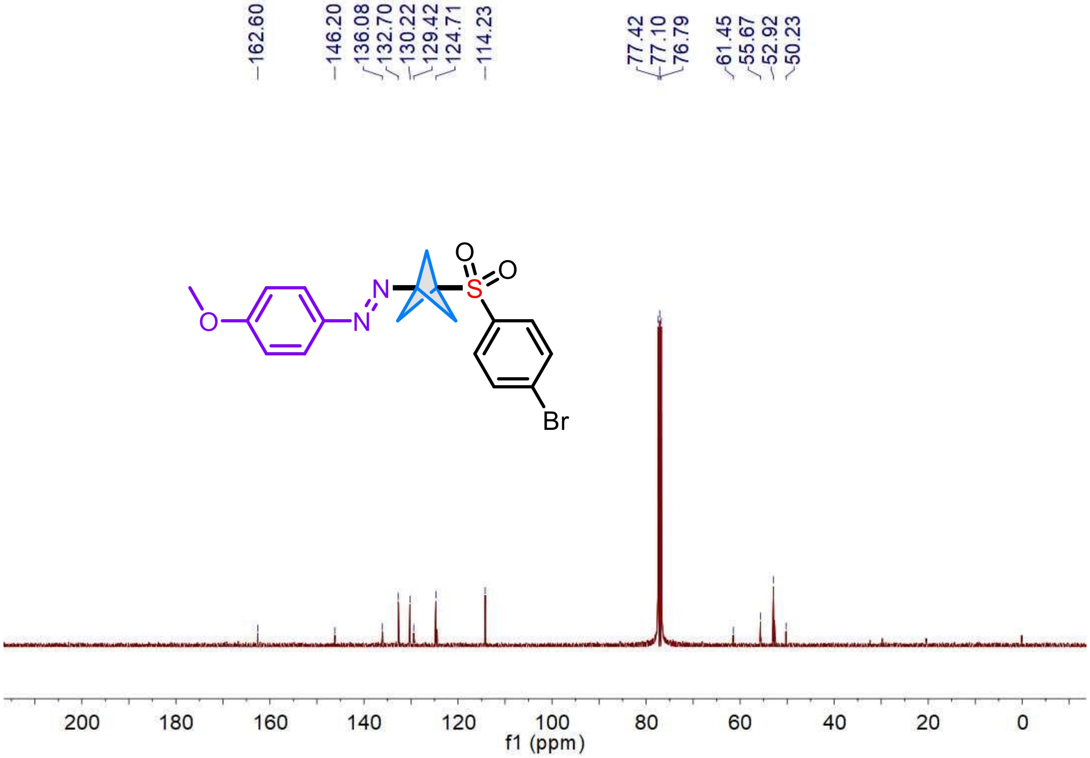

56 ^{1}H NMR (400 MHz, $CDCl_3$)

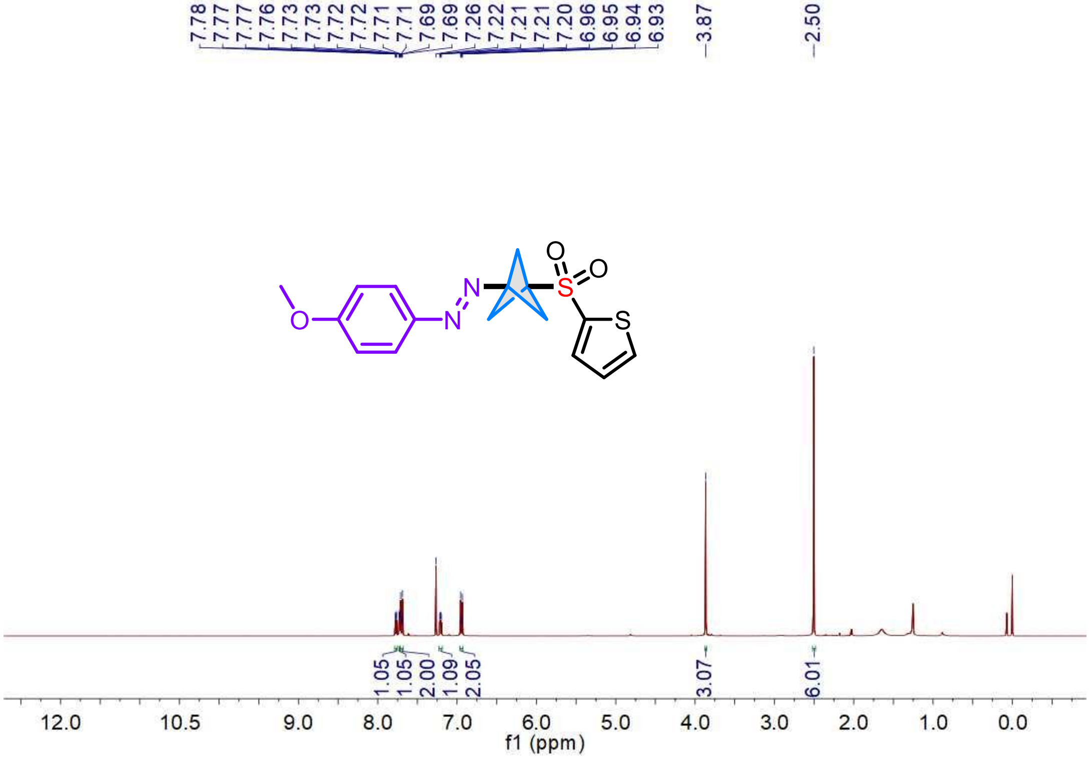

56 ^{13}C NMR (101 MHz, CDCl$_3$)

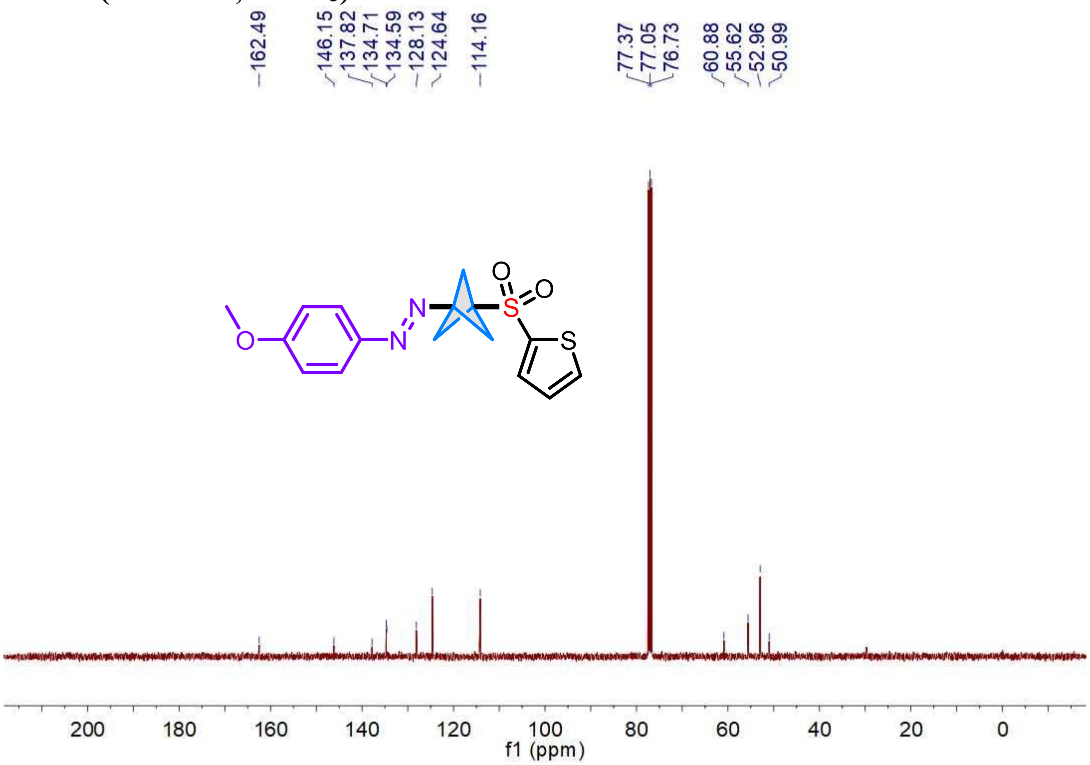

57 ^{1}H NMR (400 MHz, CDCl$_3$)

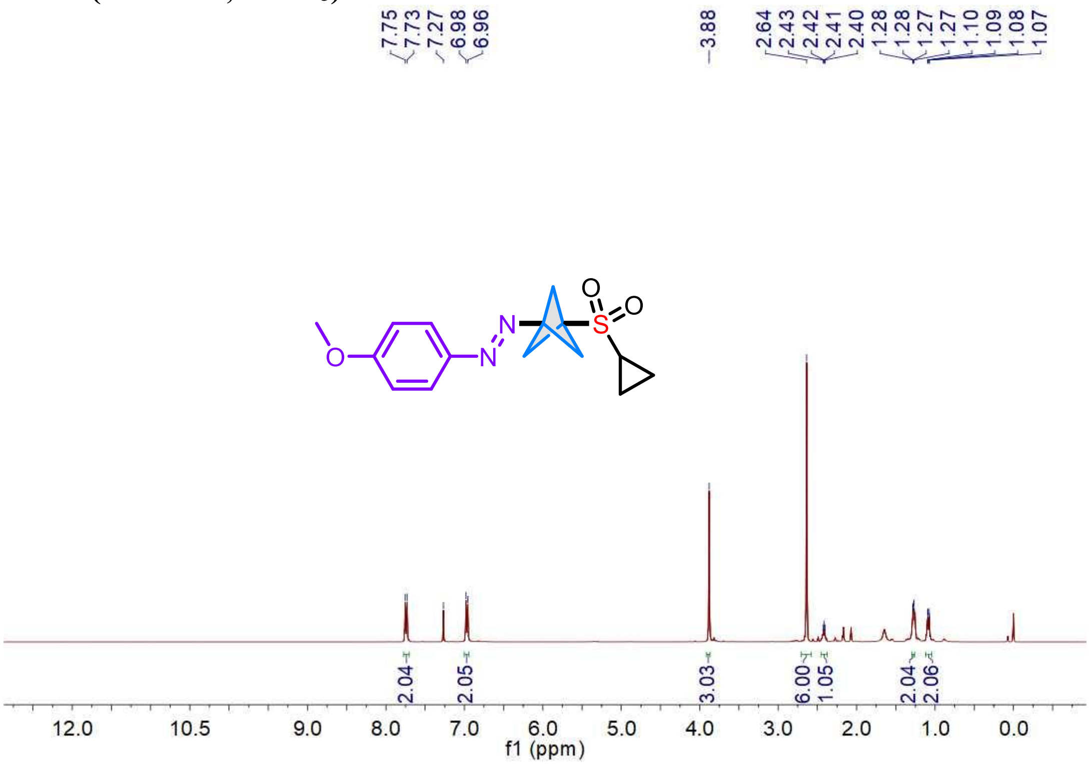

57 ^{13}C NMR (101 MHz, CDCl$_3$)

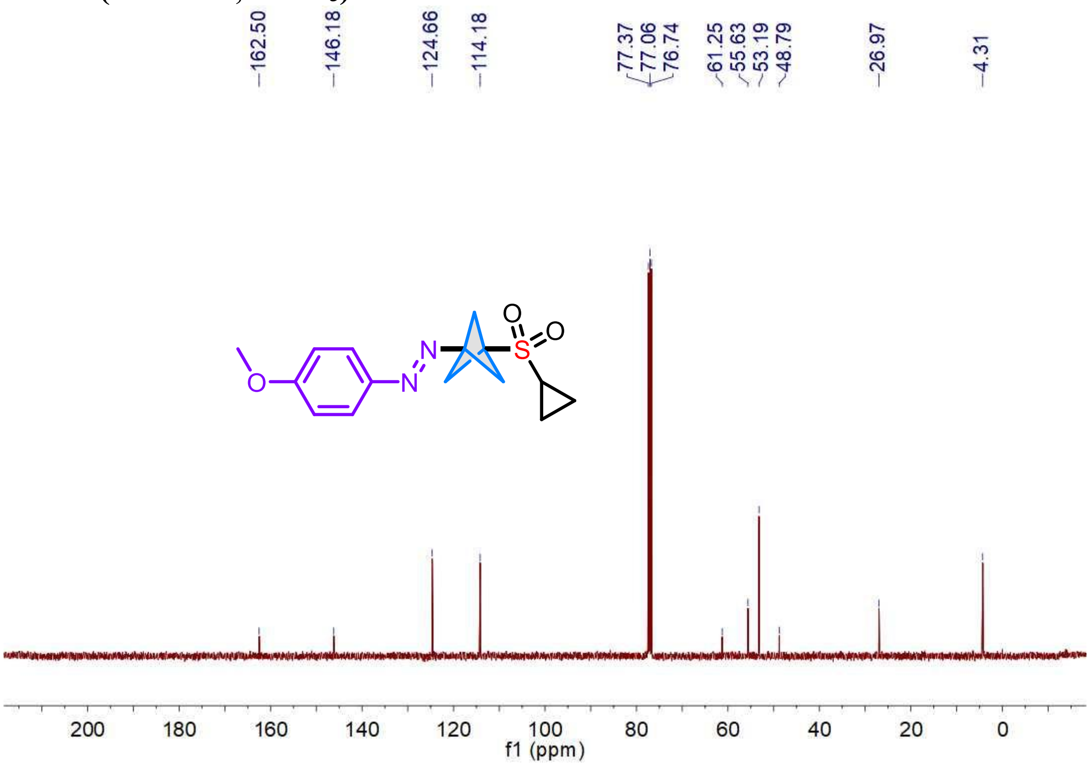

58 ^{1}H NMR (500 MHz, CDCl$_3$)

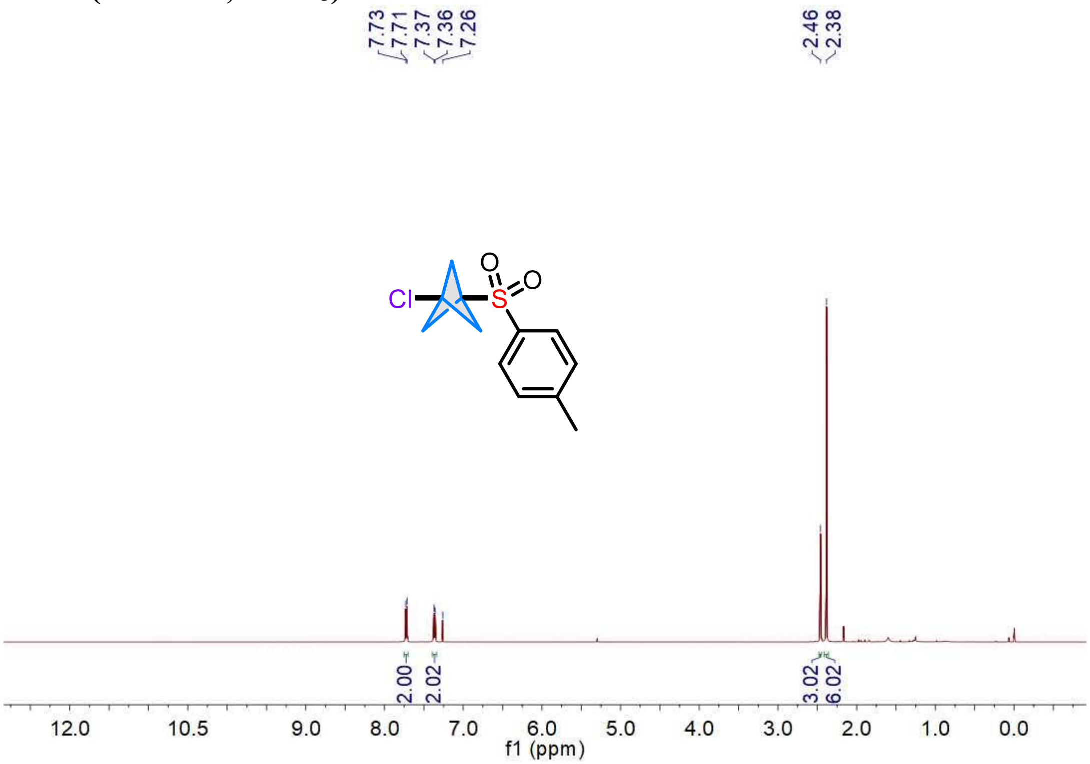

58 ^{13}C NMR (126 MHz, $CDCl_3$)

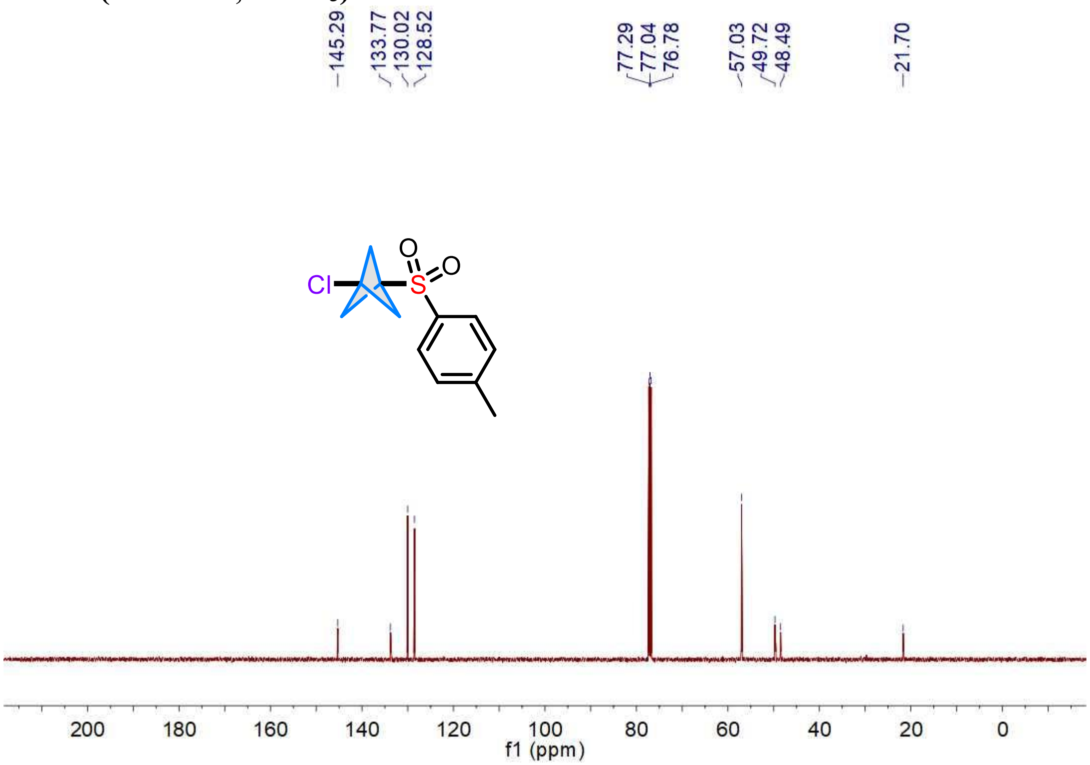

59 ^{1}H NMR (500 MHz, $CDCl_3$)

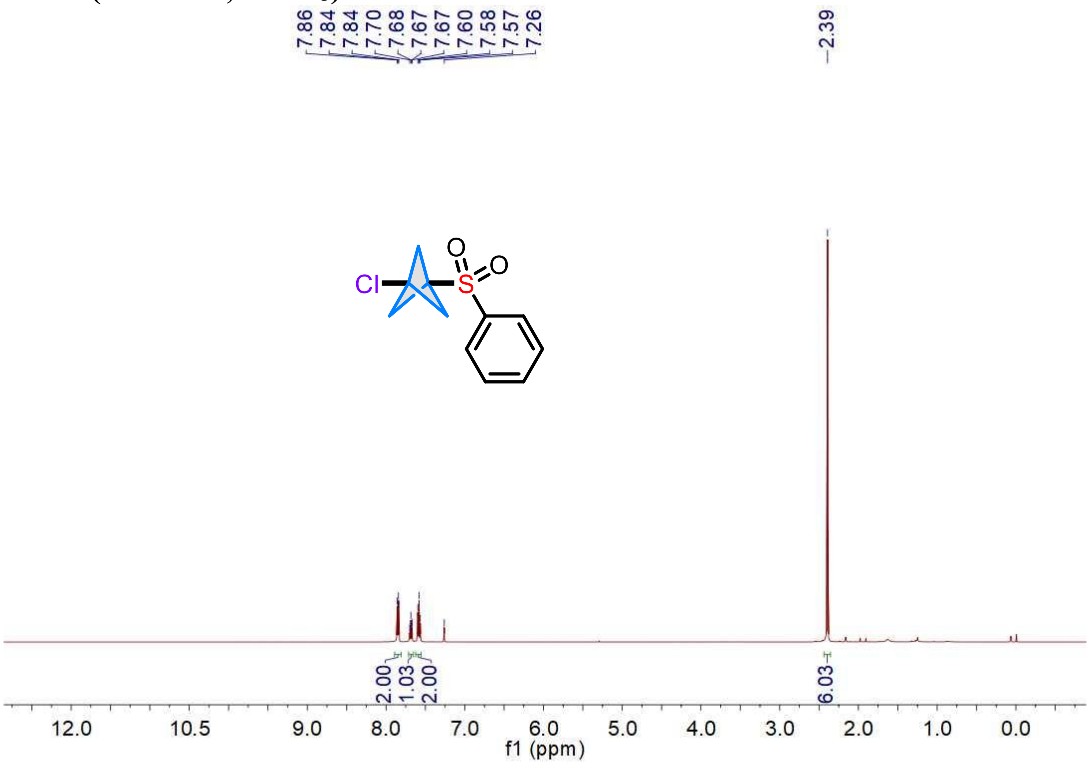

59 ^{13}C NMR (126 MHz, $CDCl_3$)

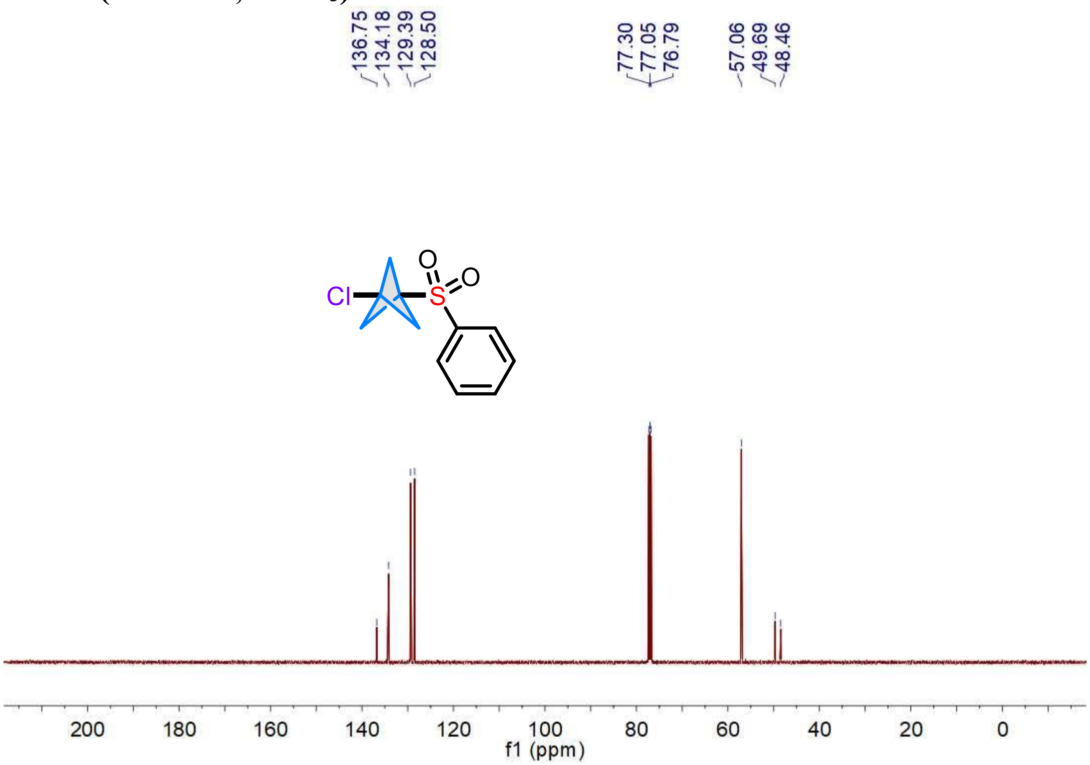

60 ^{1}H NMR (500 MHz, $CDCl_3$)

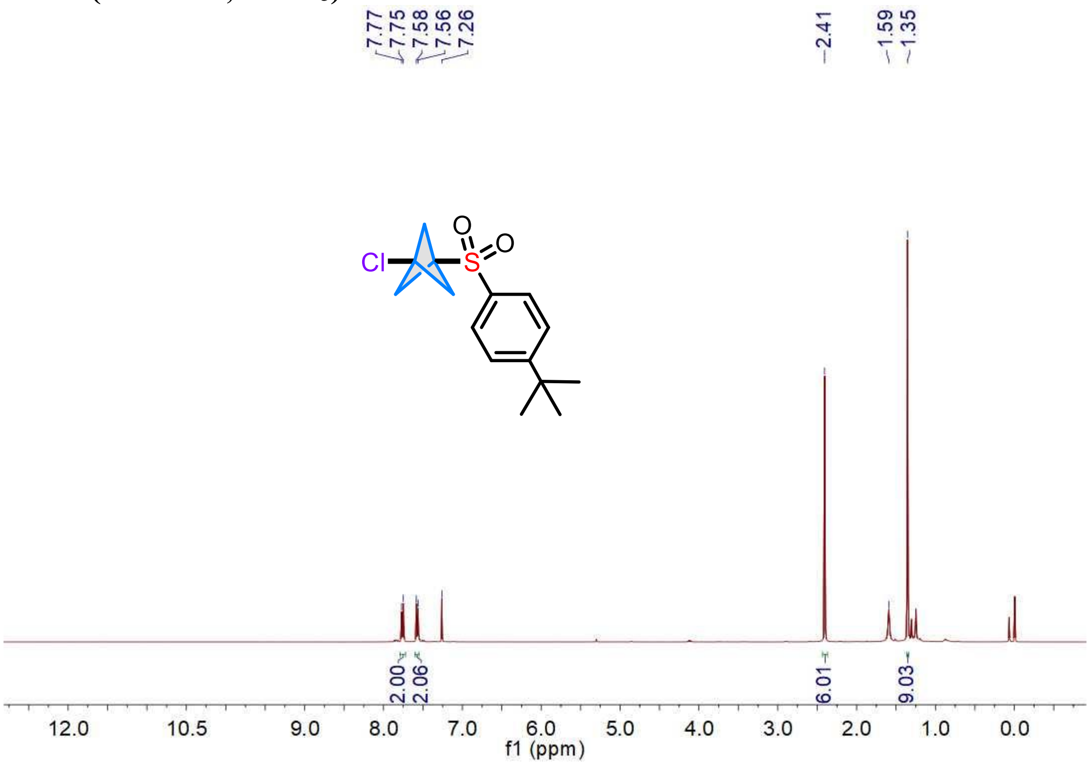

60 ^{13}C NMR (126 MHz, $CDCl_3$)

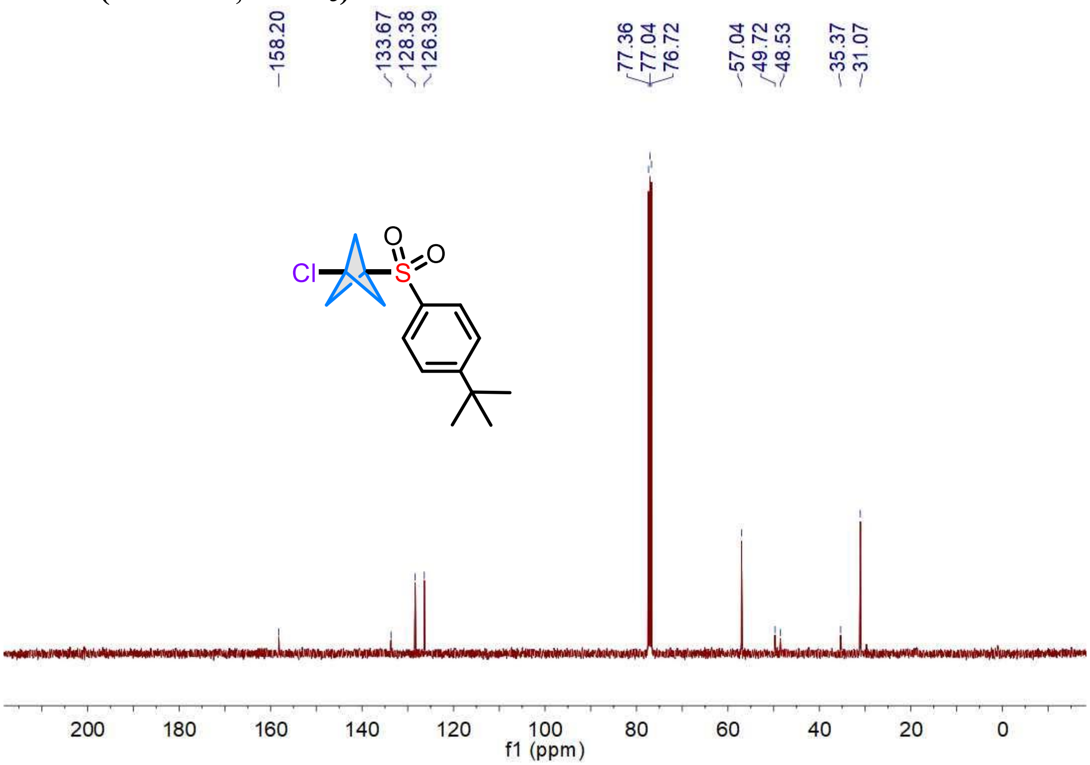

61 ^{1}H NMR (500 MHz, $CDCl_3$)

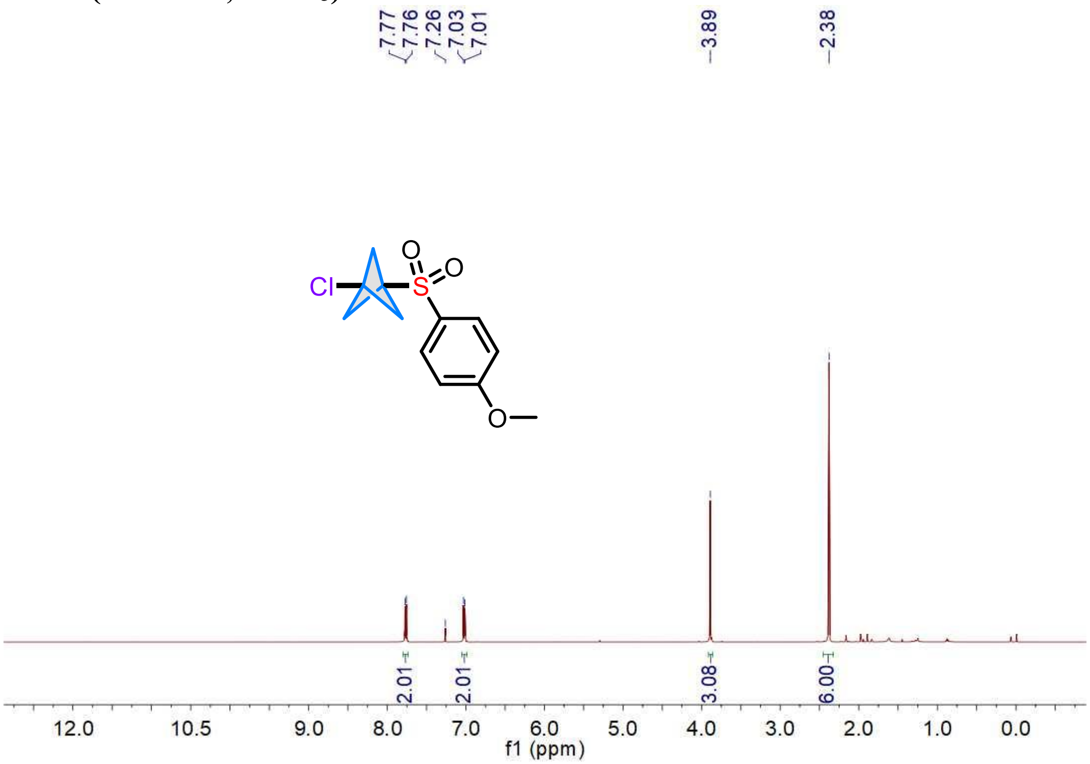

61 ^{13}C NMR (126 MHz, $CDCl_3$)

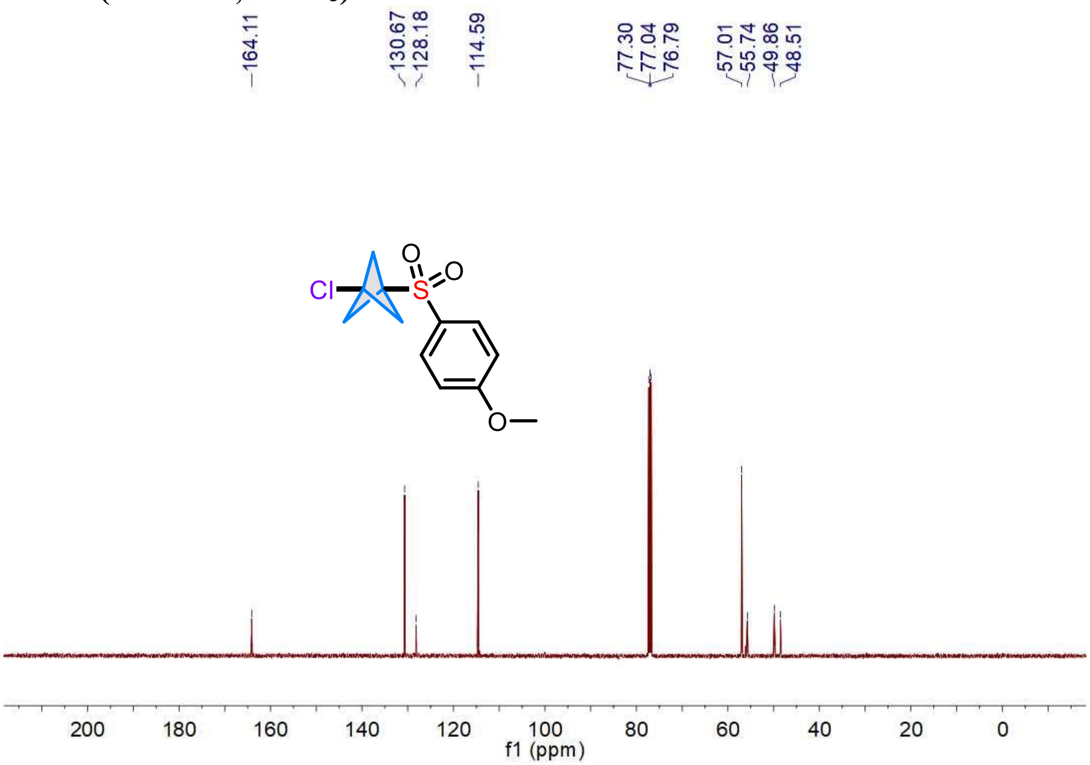

62 ^{1}H NMR (500 MHz, $CDCl_3$)

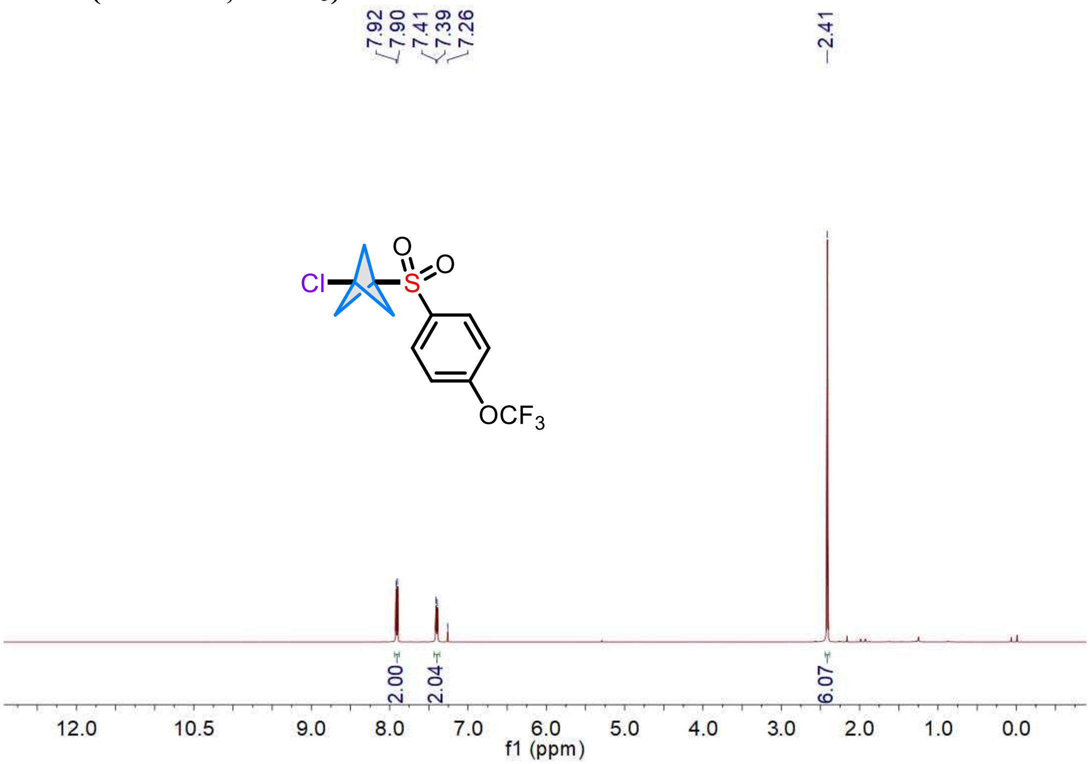

62 ^{13}C NMR (126 MHz, CDCl$_3$)

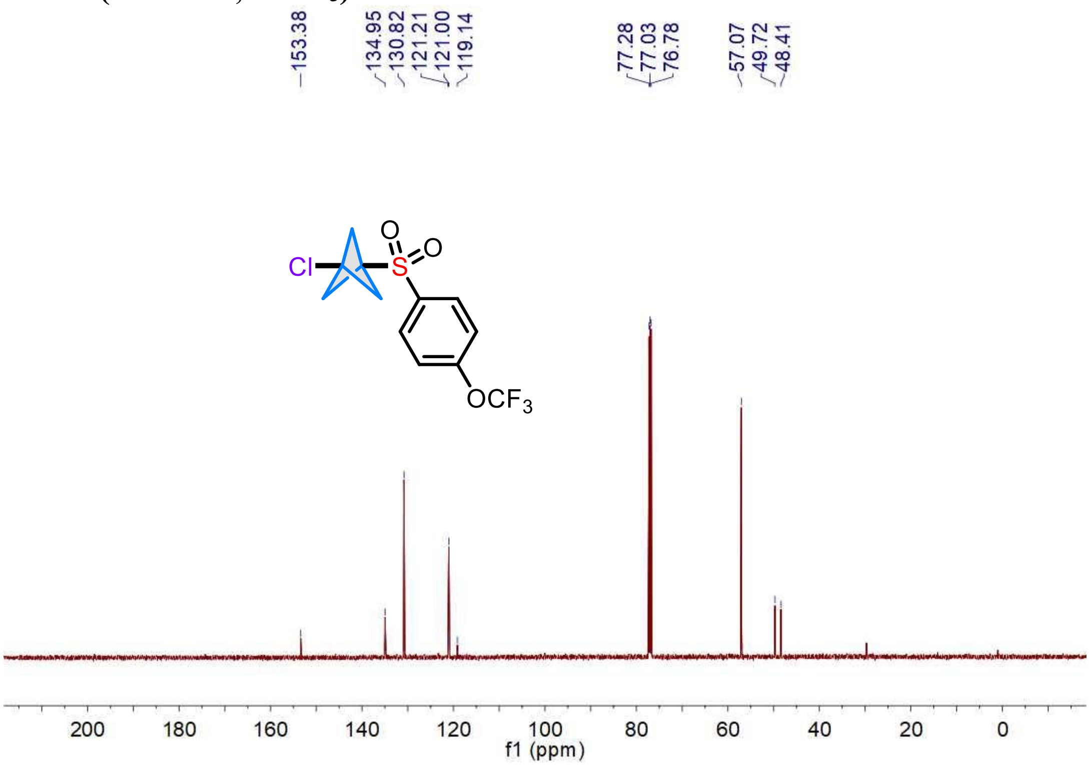

62 ^{19}F NMR (471 MHz, CDCl$_3$)

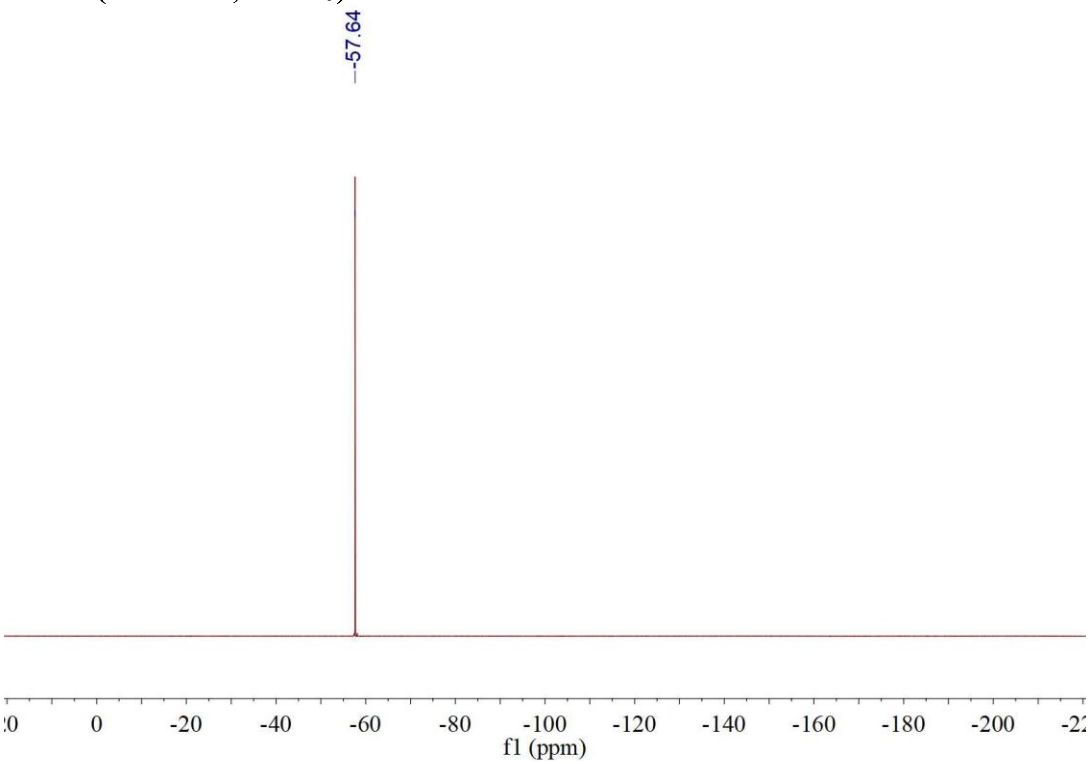

63 ^{1}H NMR (400 MHz, CDCl$_3$)

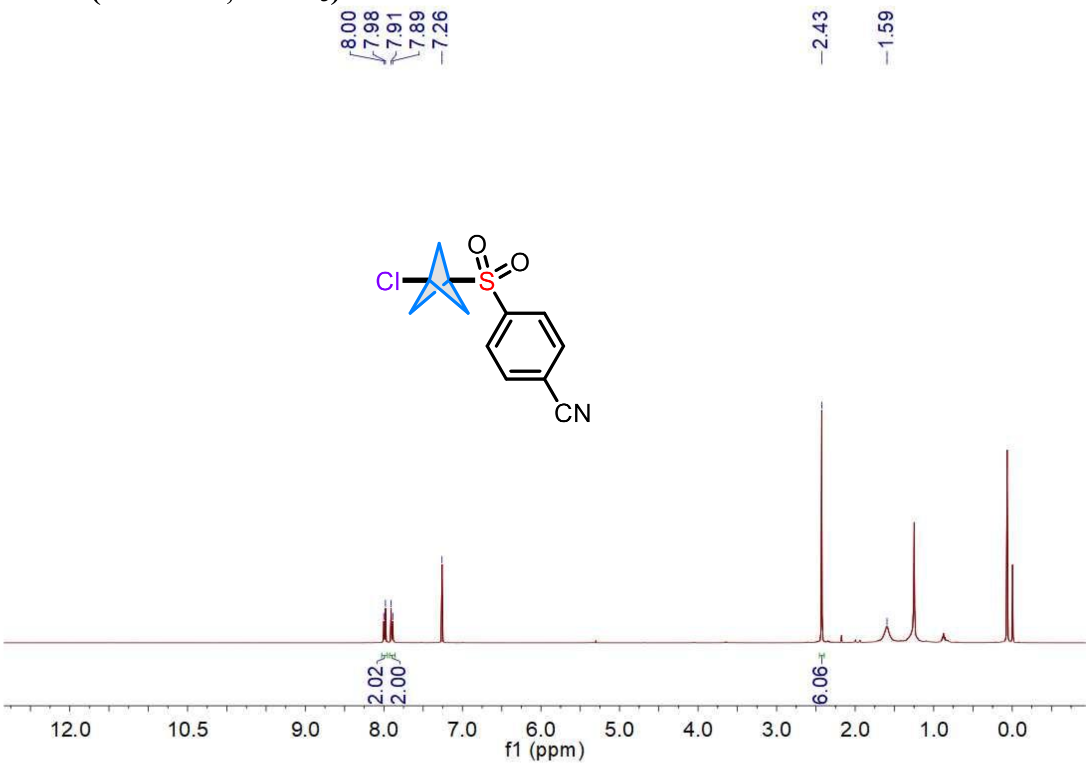

63 ^{13}C NMR (101 MHz, CDCl$_3$)

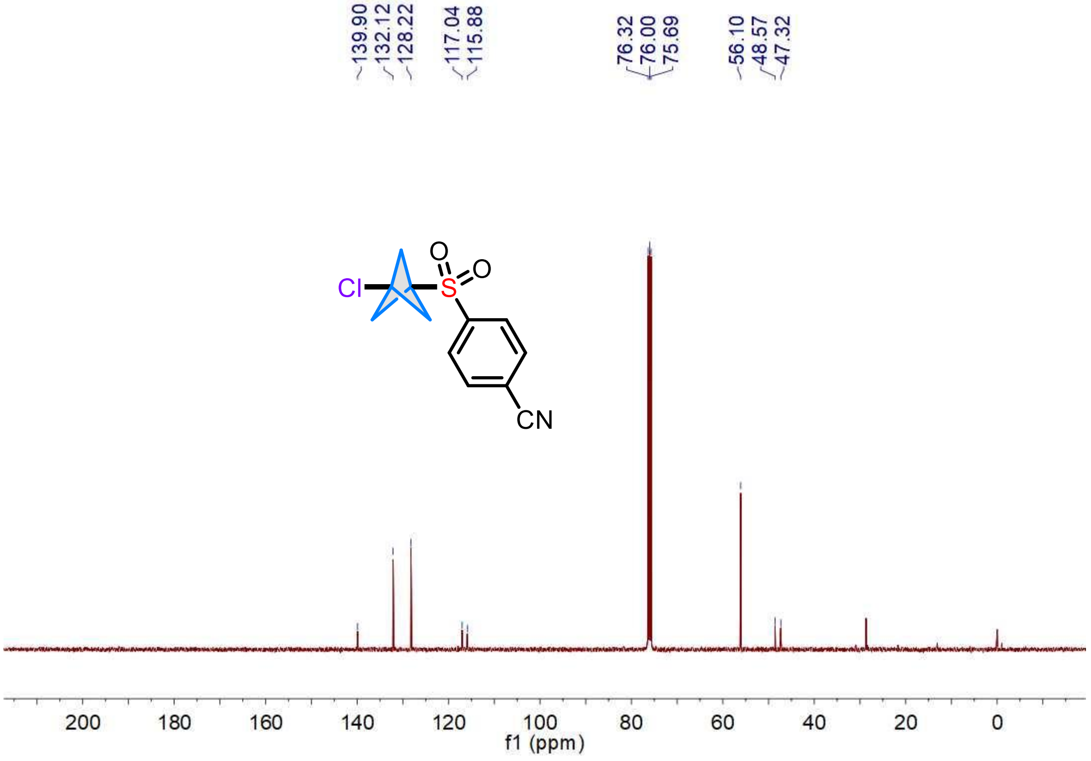

64 ^{1}H NMR (500 MHz, CDCl$_3$)

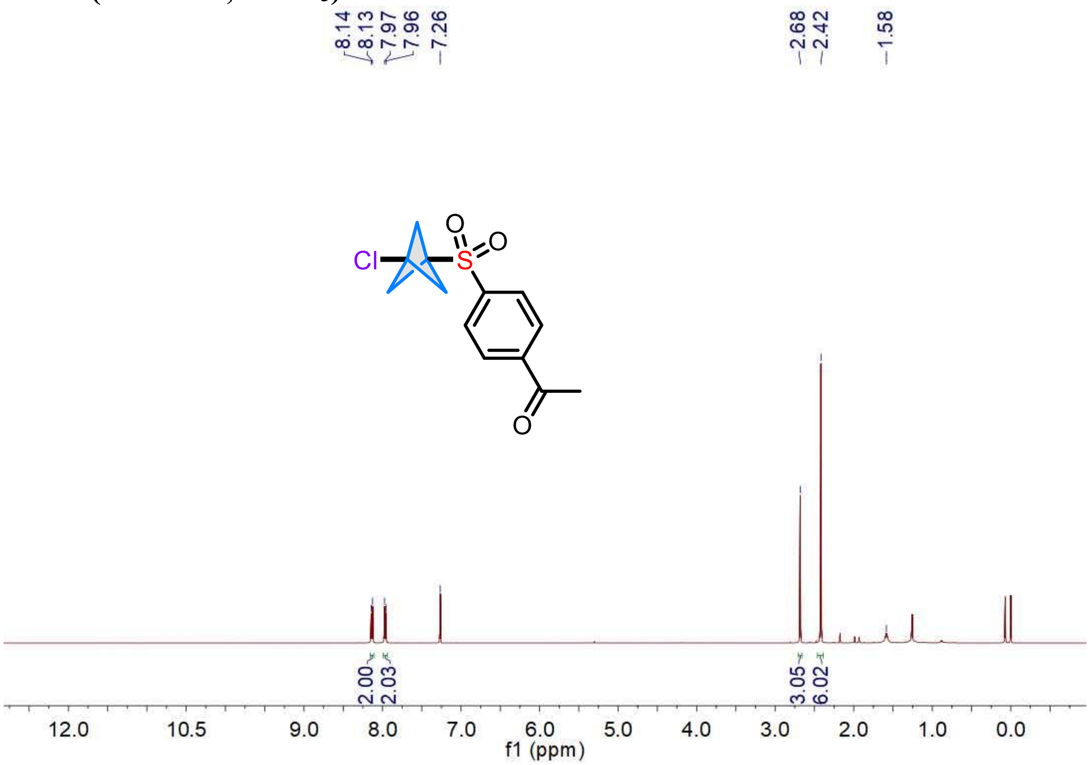

64 ^{13}C NMR (126 MHz, CDCl$_3$)

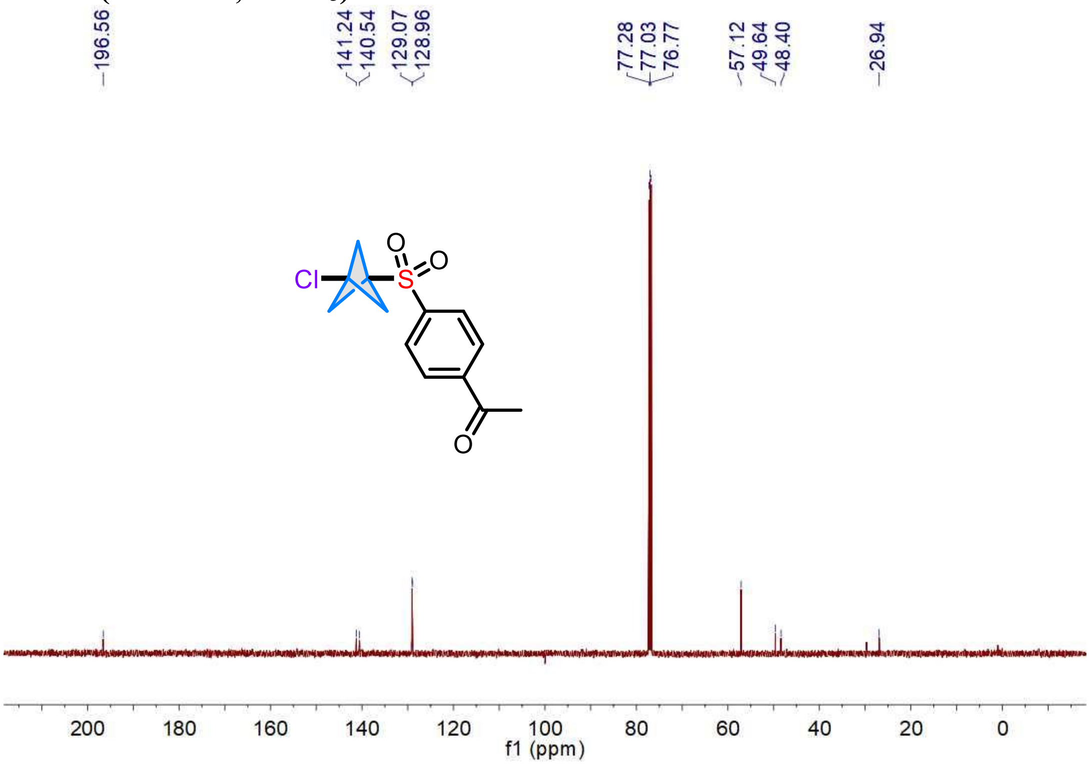

65 ^{1}H NMR (500 MHz, $CDCl_3$)

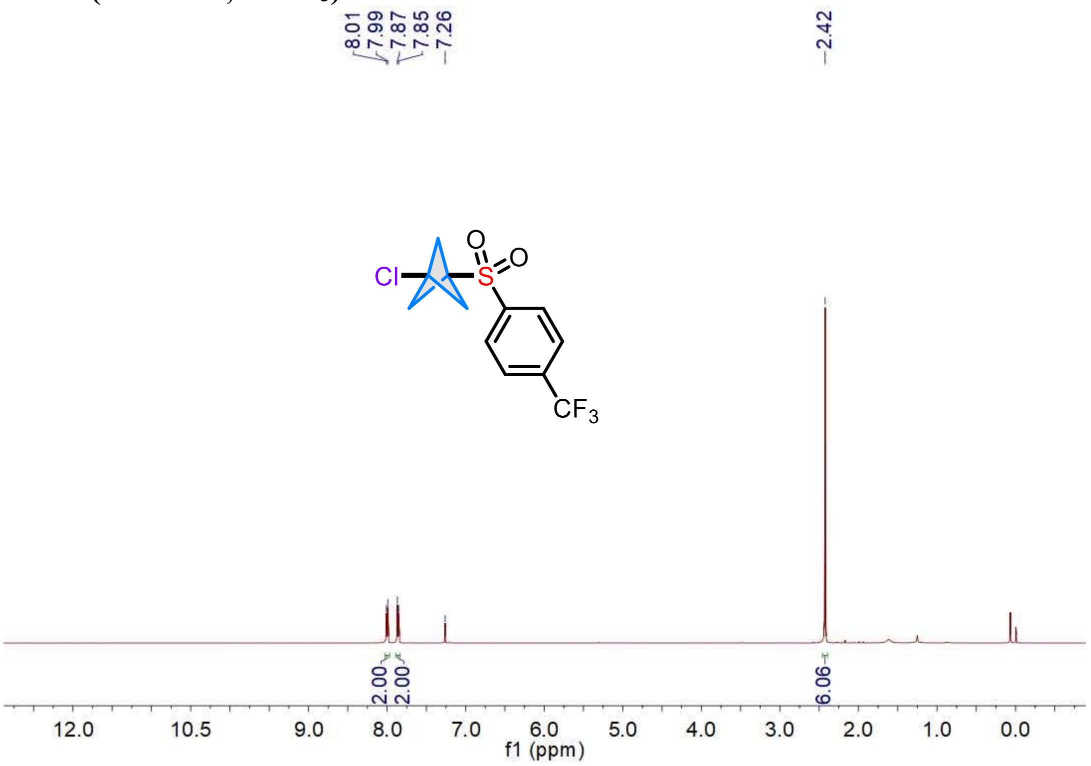

65 ^{13}C NMR (126 MHz, $CDCl_3$)

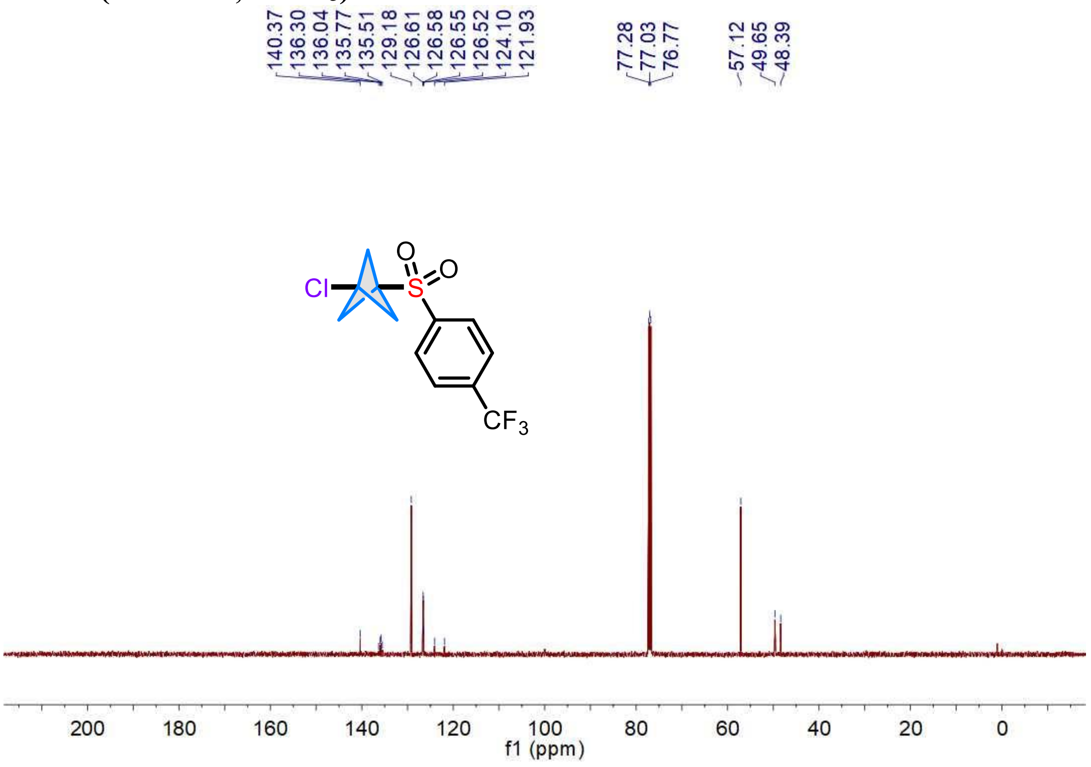

65 ^{19}F NMR (471 MHz, $CDCl_3$)

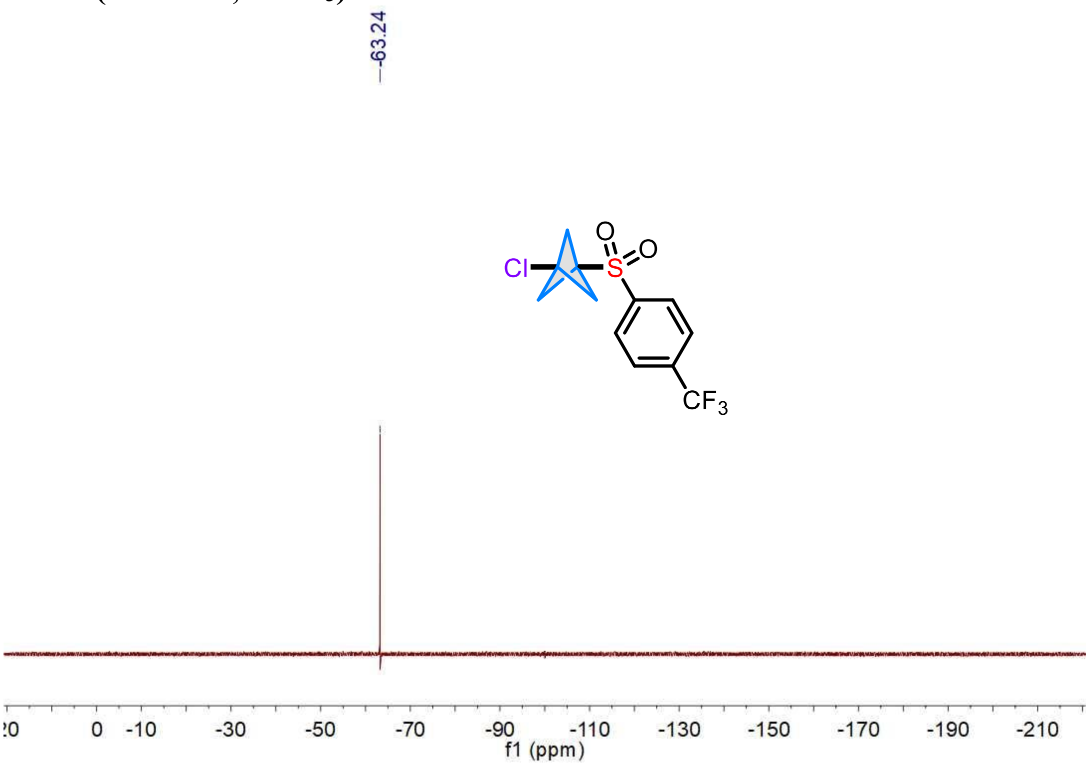

66 ^{1}H NMR (500 MHz, $CDCl_3$)

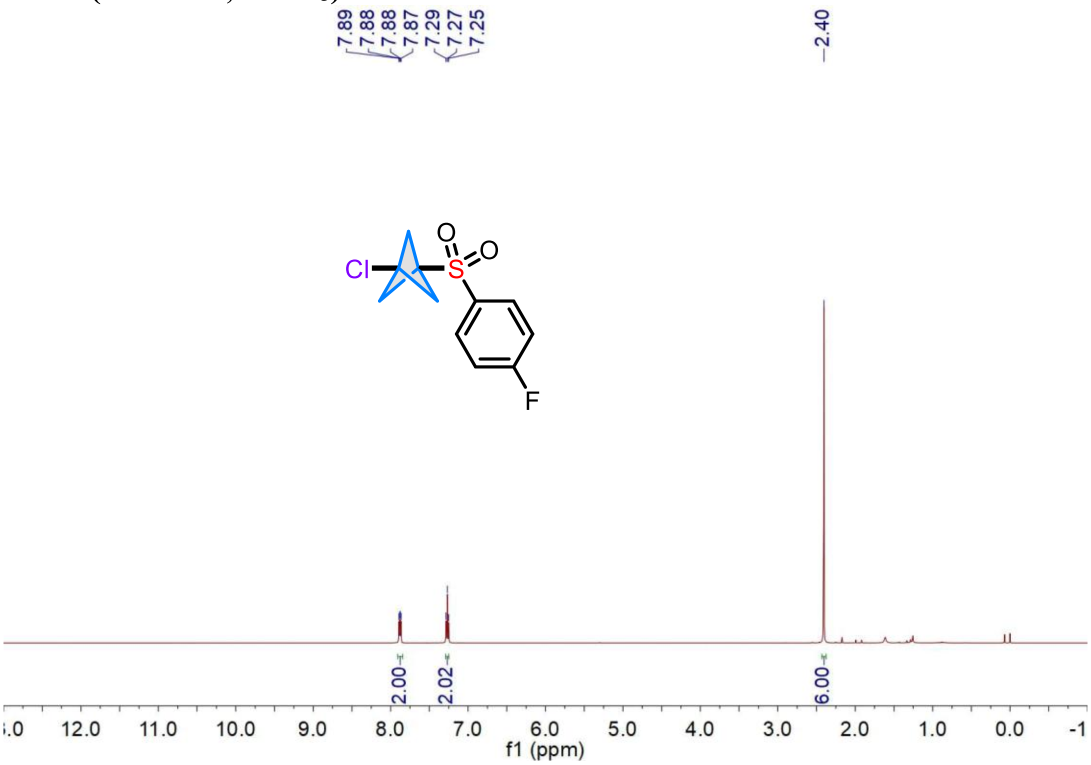

66 ^{13}C NMR (126 MHz, $CDCl_3$)

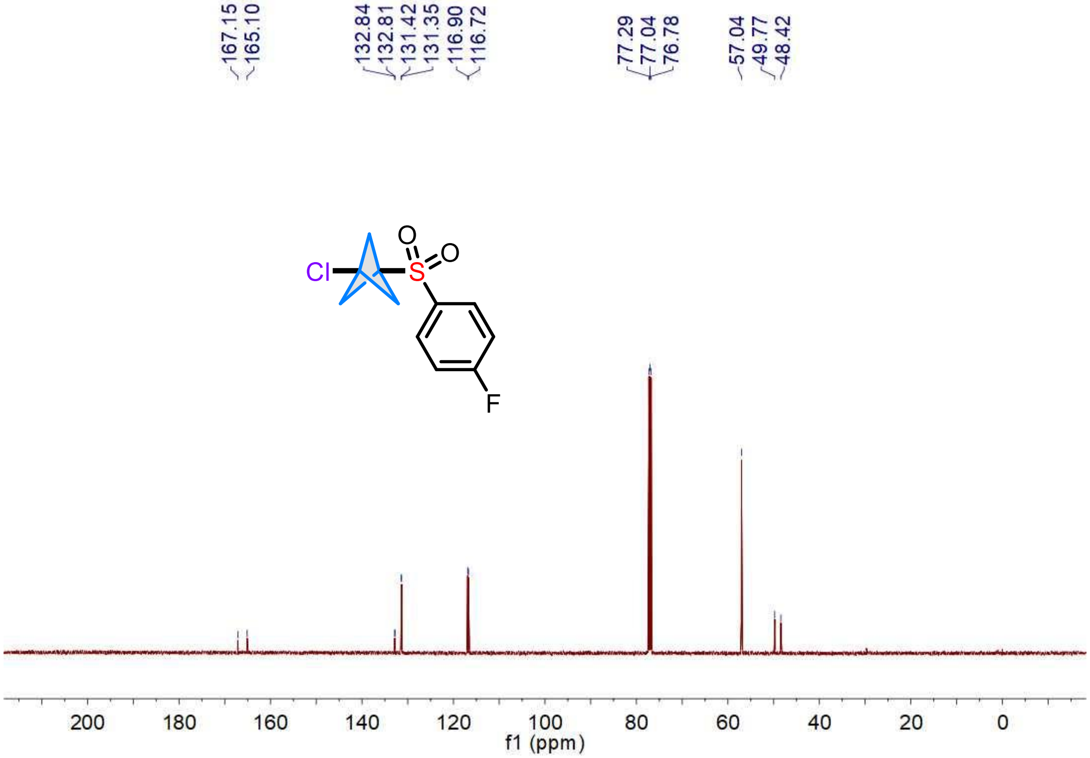

66 ^{19}F NMR (471 MHz, $CDCl_3$)

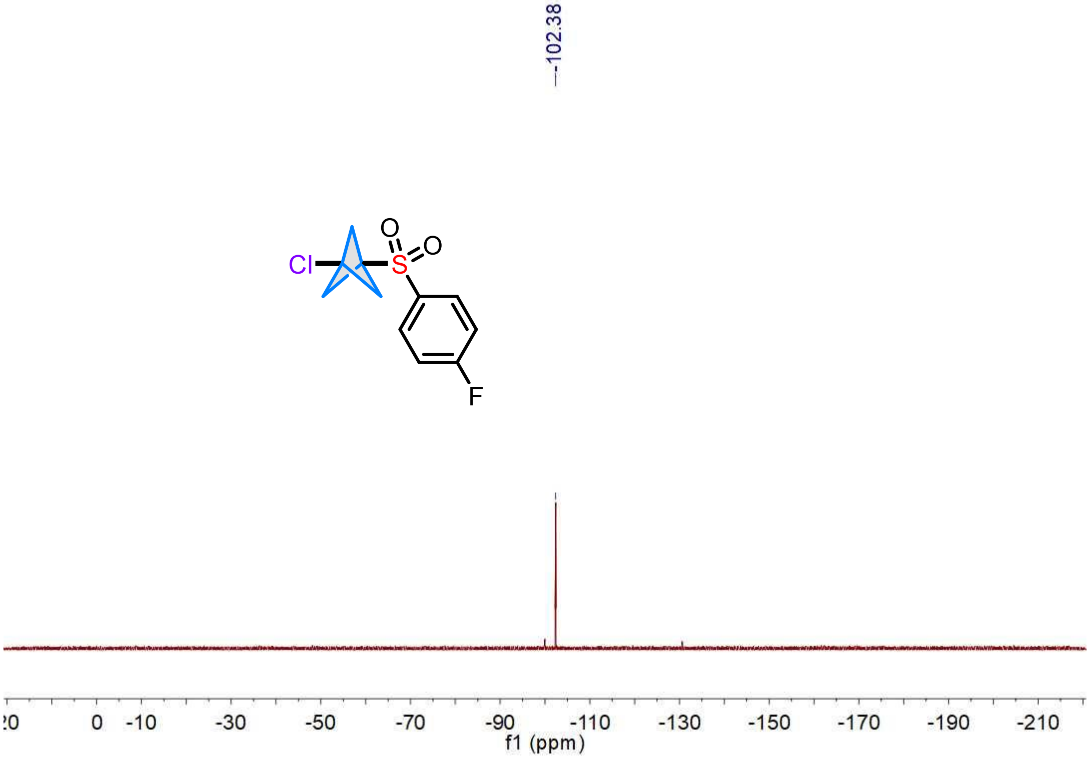

67 ^{1}H NMR (500 MHz, $CDCl_3$)

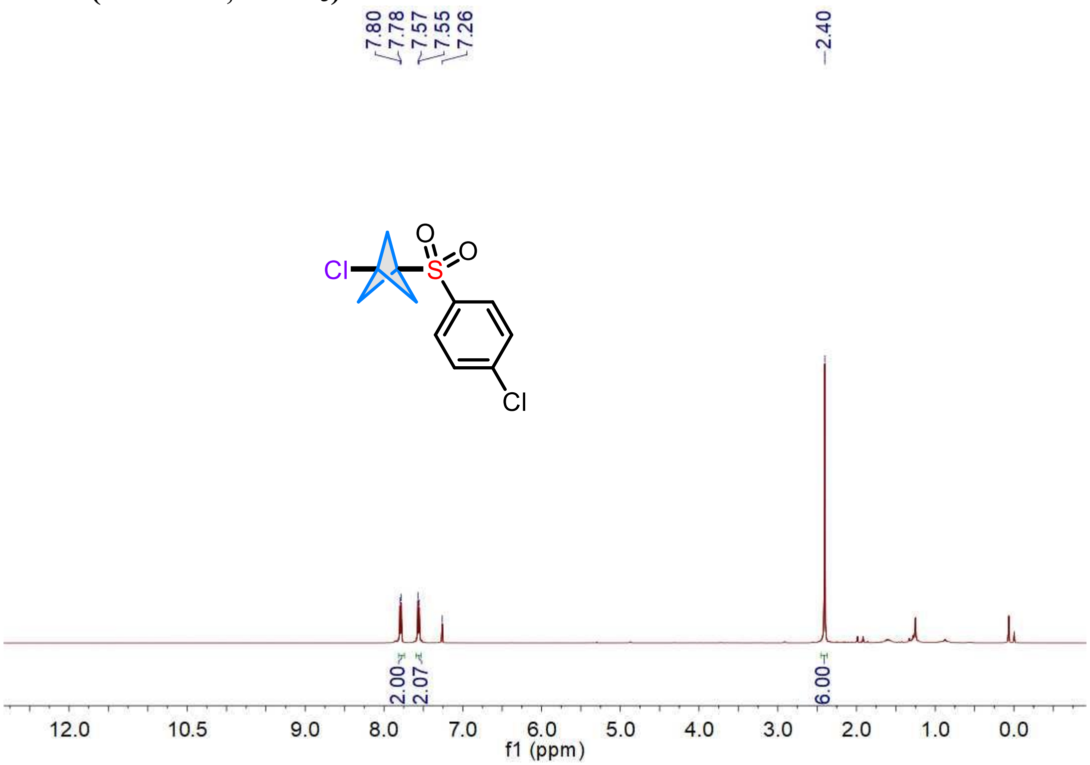

67 ^{13}C NMR (126 MHz, $CDCl_3$)

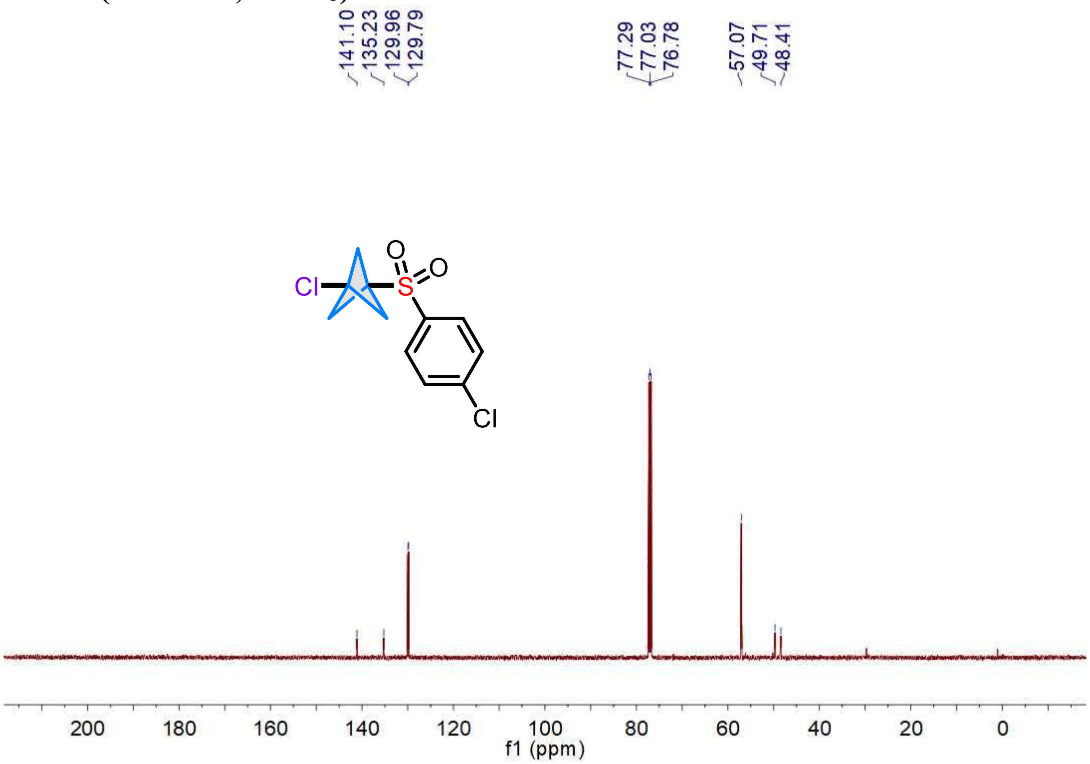

68 ^{1}H NMR (500 MHz, $CDCl_3$)

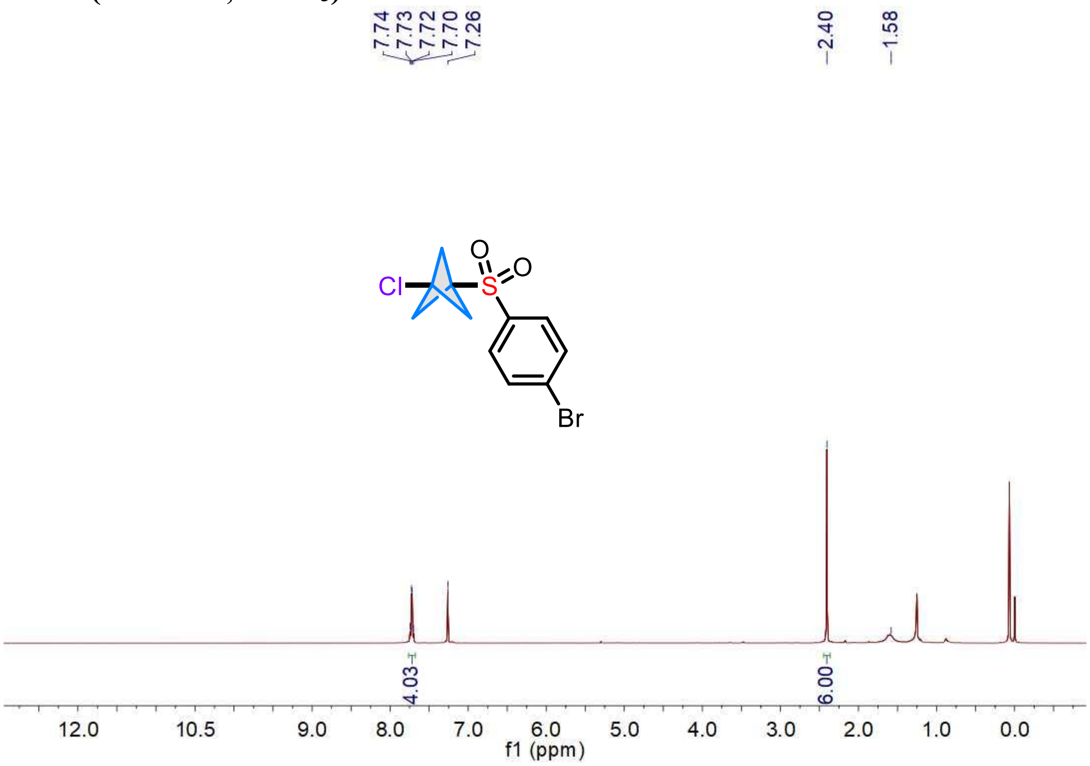

68 ^{13}C NMR (126 MHz, $CDCl_3$)

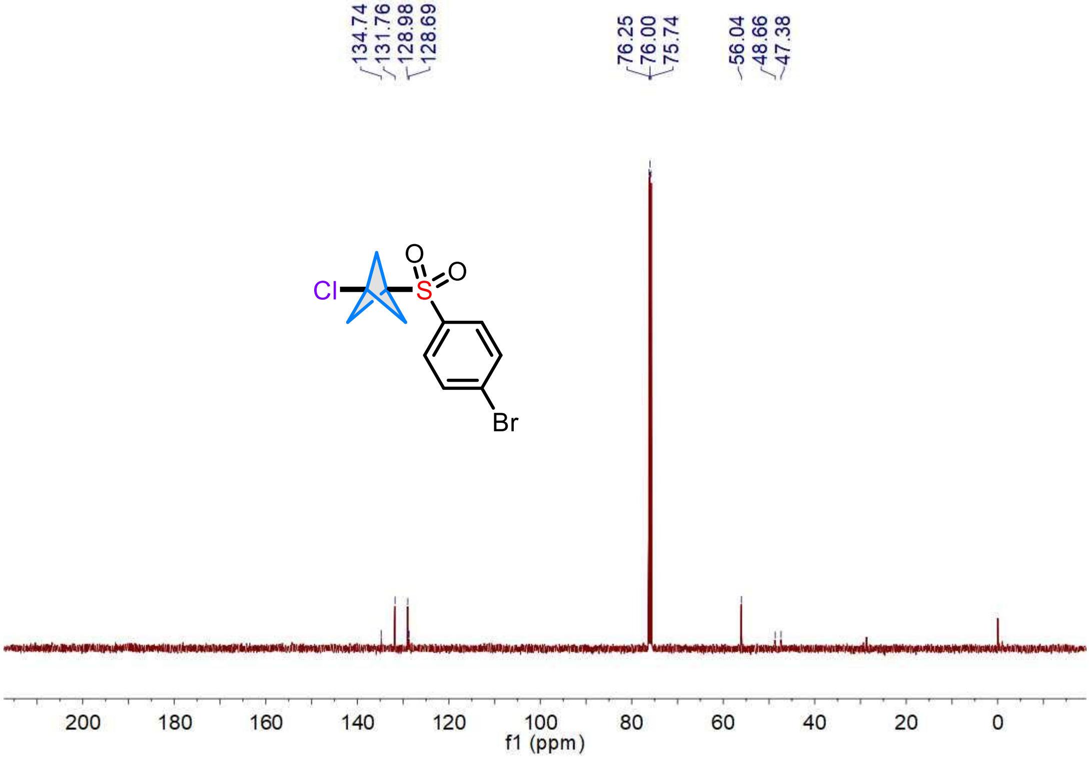

69 ^{1}H NMR (500 MHz, $CDCl_3$)

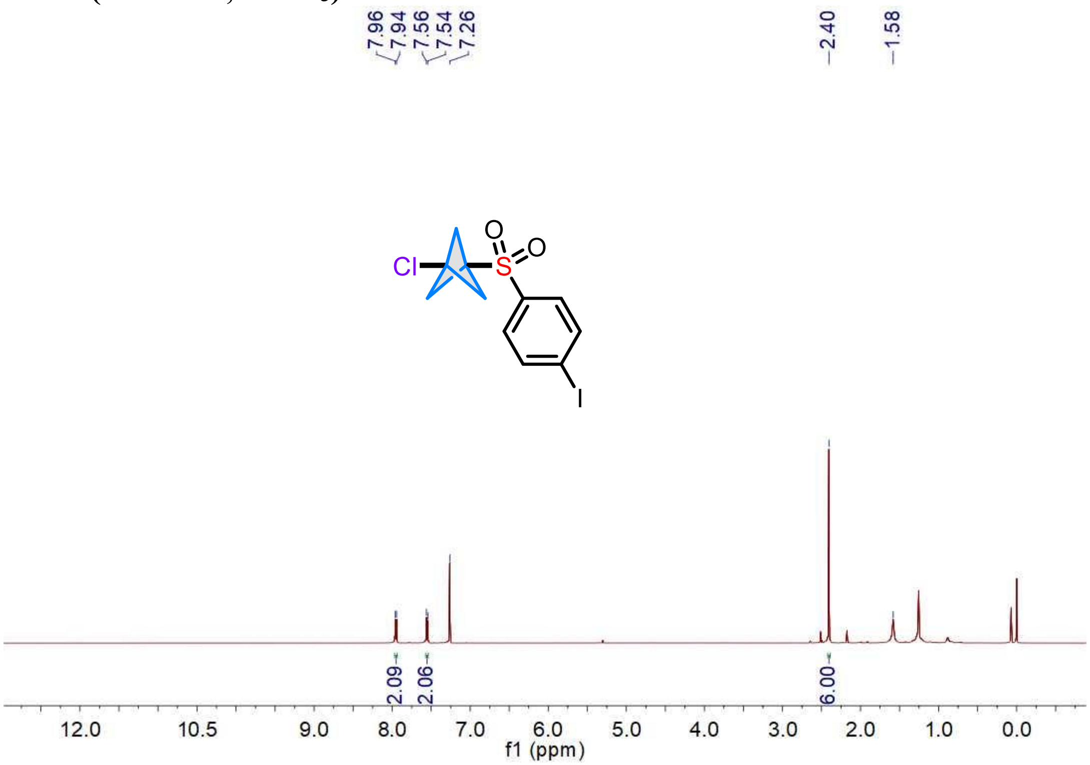

69 ^{13}C NMR (126 MHz, $CDCl_3$)

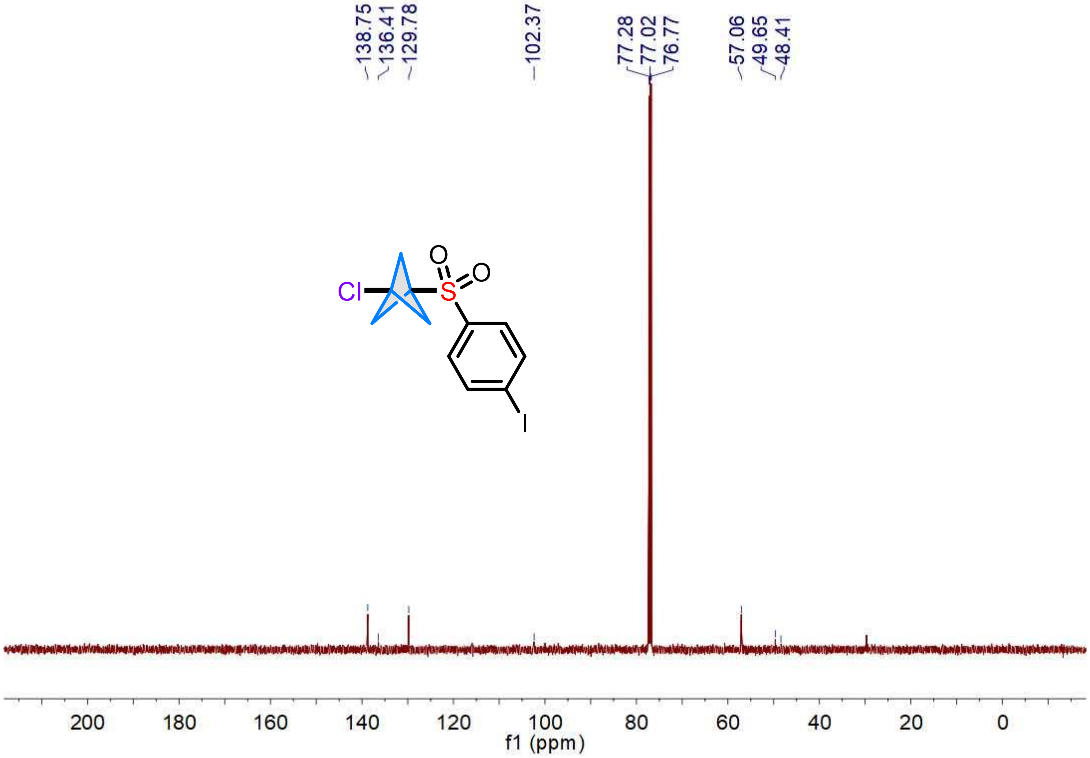

70 ^{1}H NMR (500 MHz, $CDCl_3$)

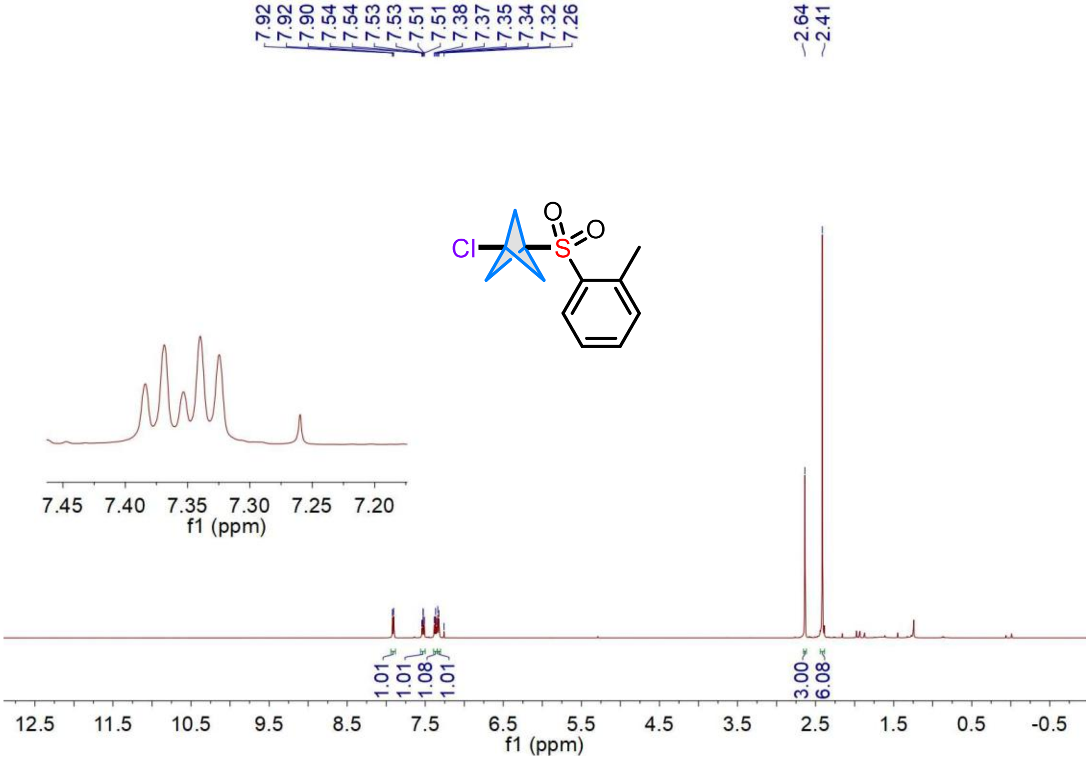

70 ^{13}C NMR (126 MHz, $CDCl_3$)

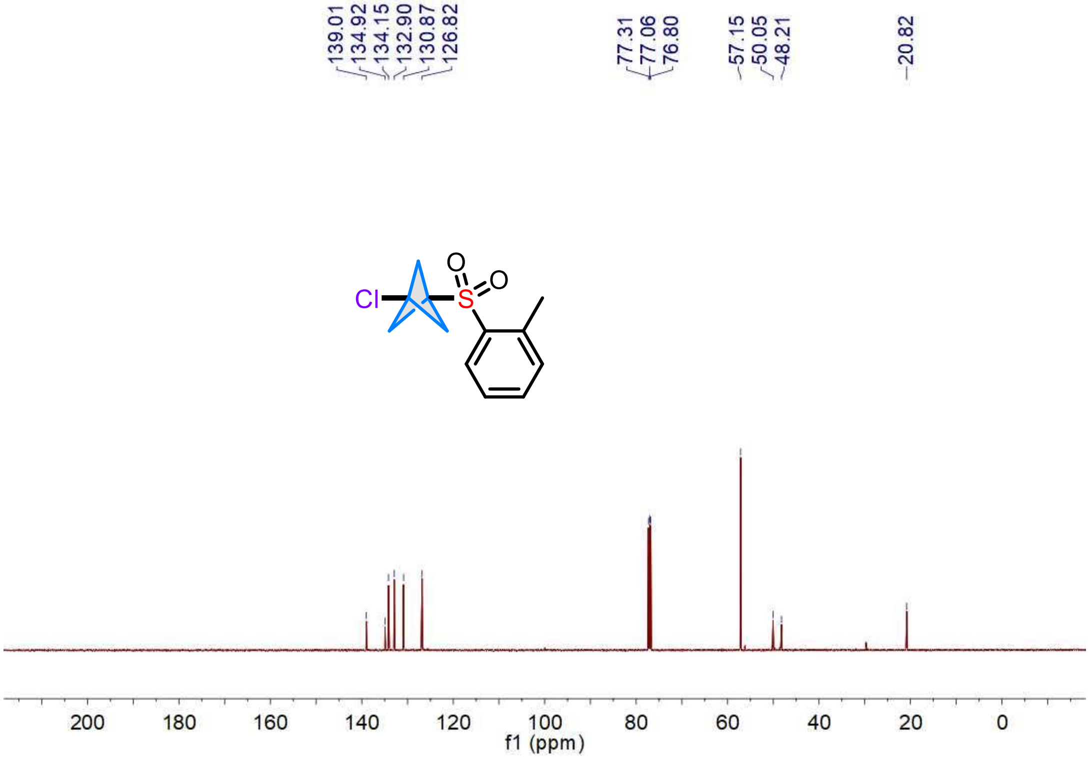

71 ^{1}H NMR (500 MHz, $CDCl_3$)

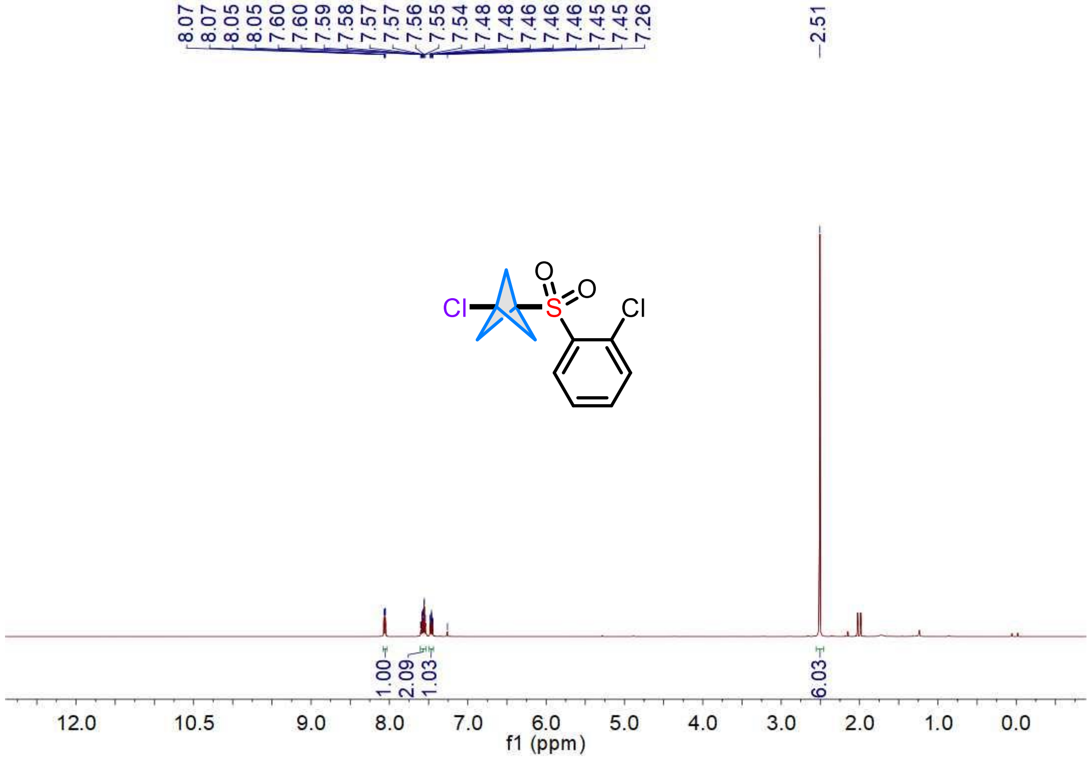

71 ^{13}C NMR (126 MHz, $CDCl_3$)

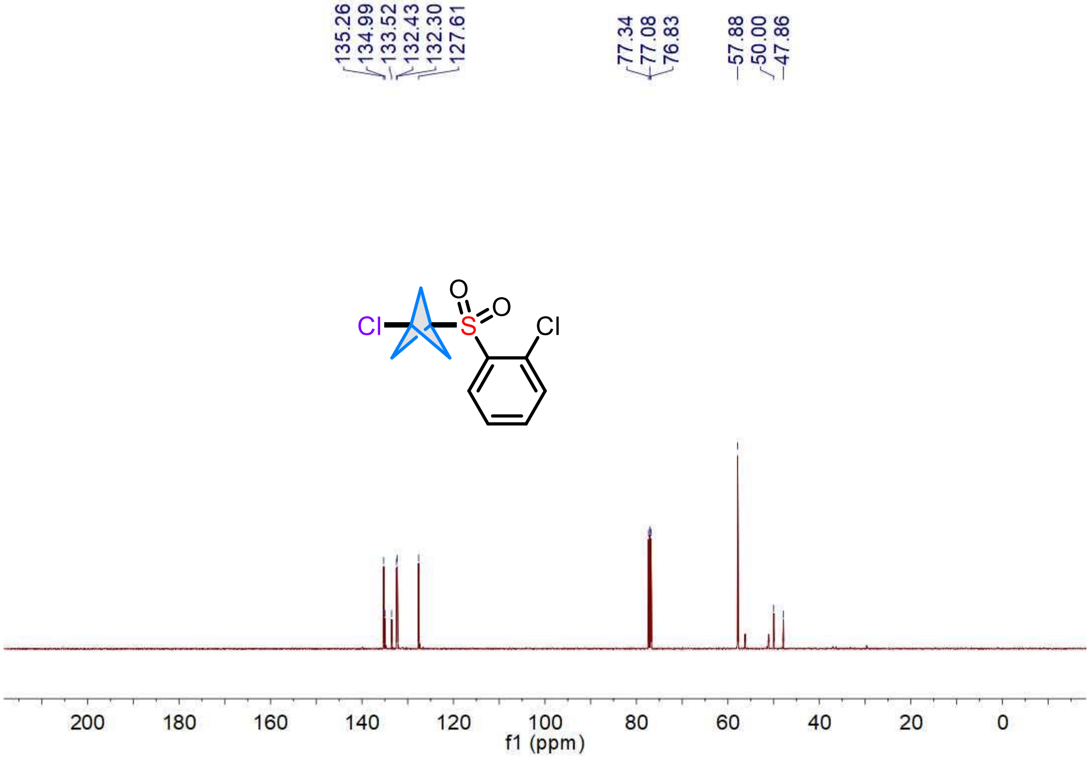

72 ^{1}H NMR (500 MHz, CDCl$_3$)

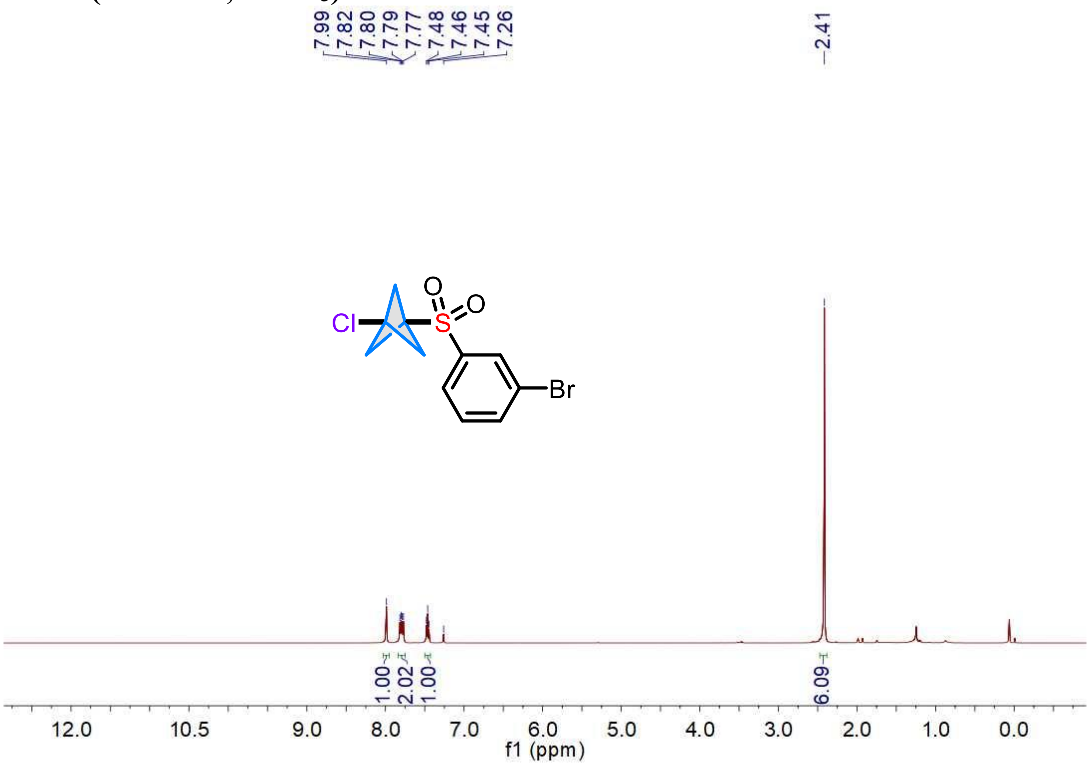

72 ^{13}C NMR (126 MHz, CDCl$_3$)

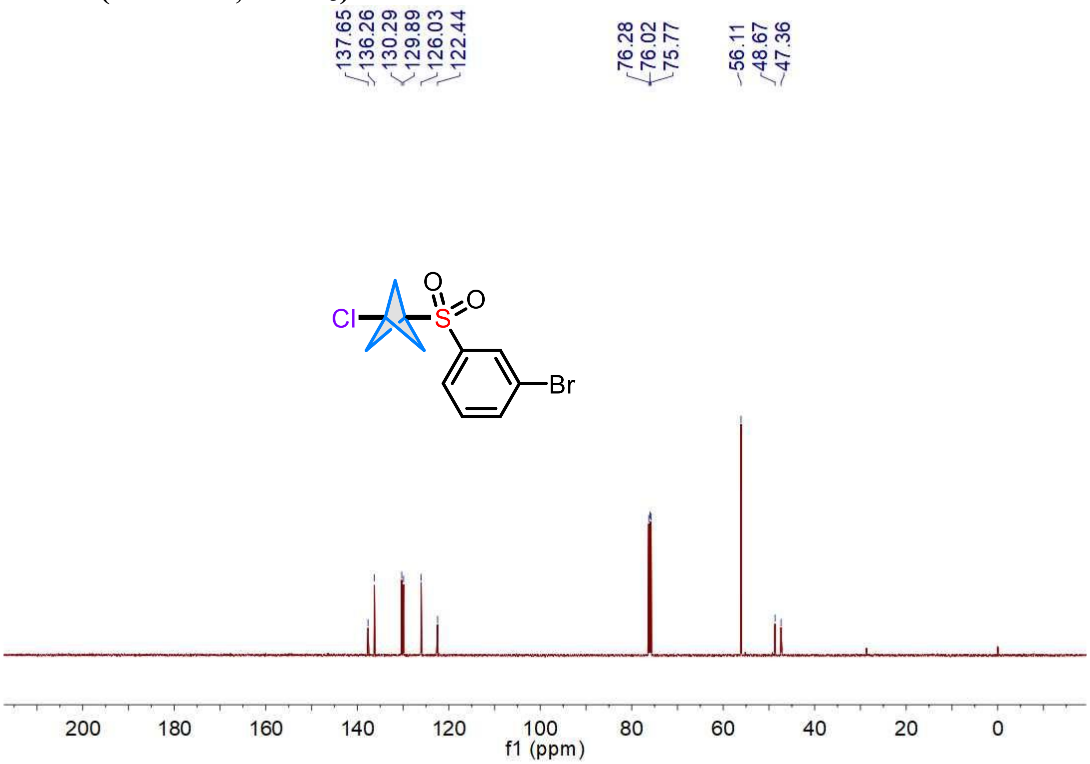

73 ^{1}H NMR (500 MHz, $CDCl_3$)

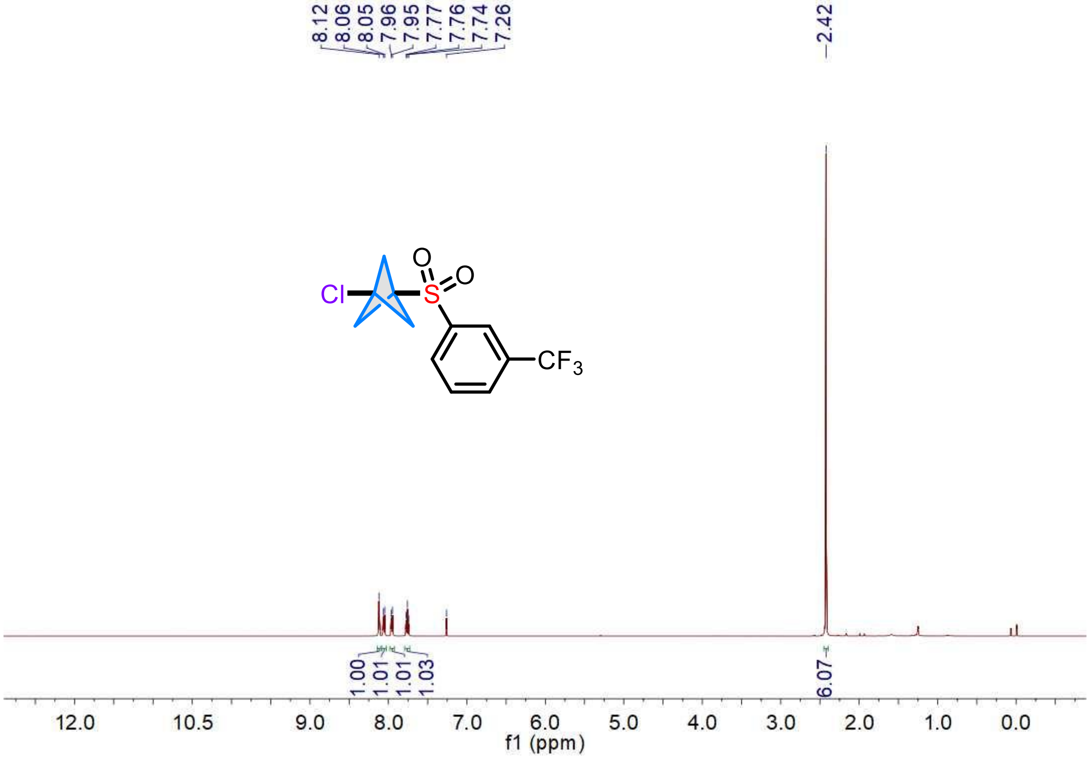

73 ^{13}C NMR (126 MHz, $CDCl_3$)

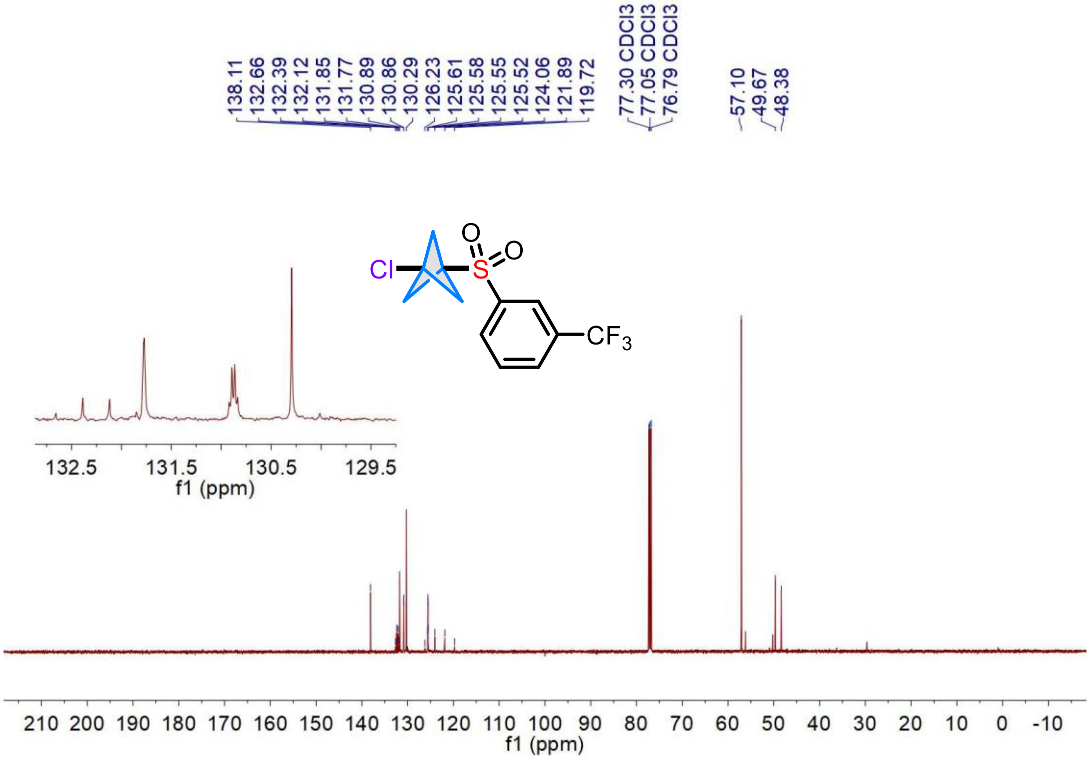

73 ^{19}F NMR (471 MHz, $CDCl_3$)

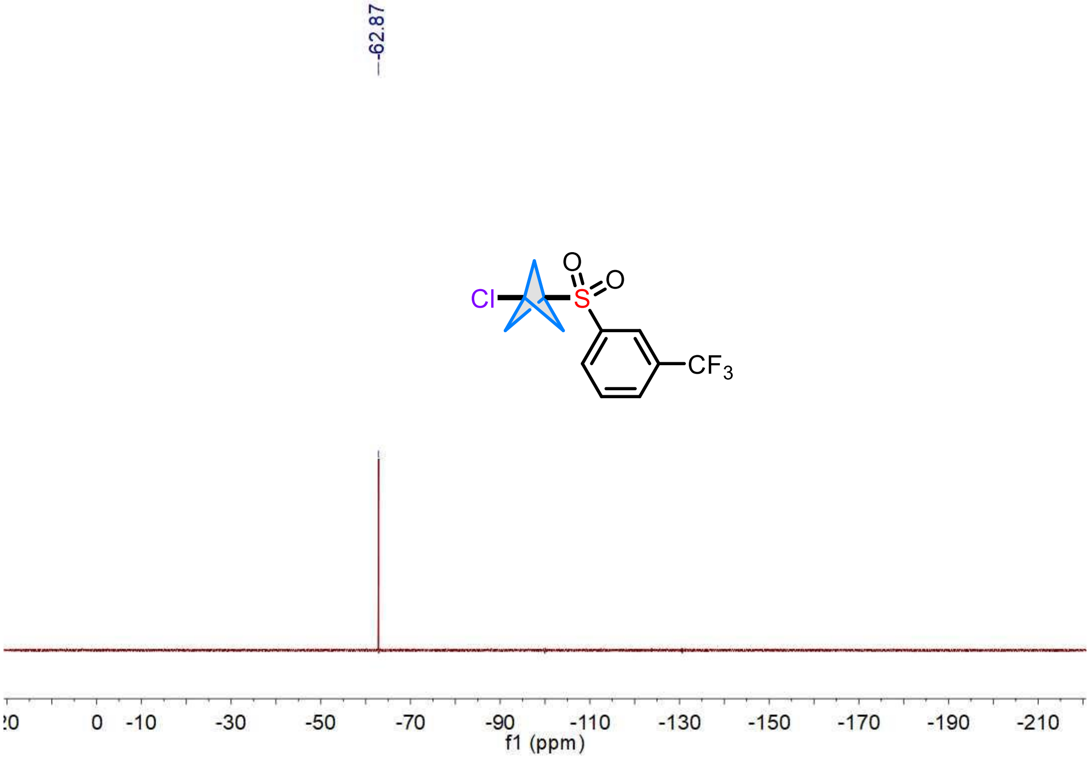

74 ^{1}H NMR (500 MHz, $CDCl_3$)

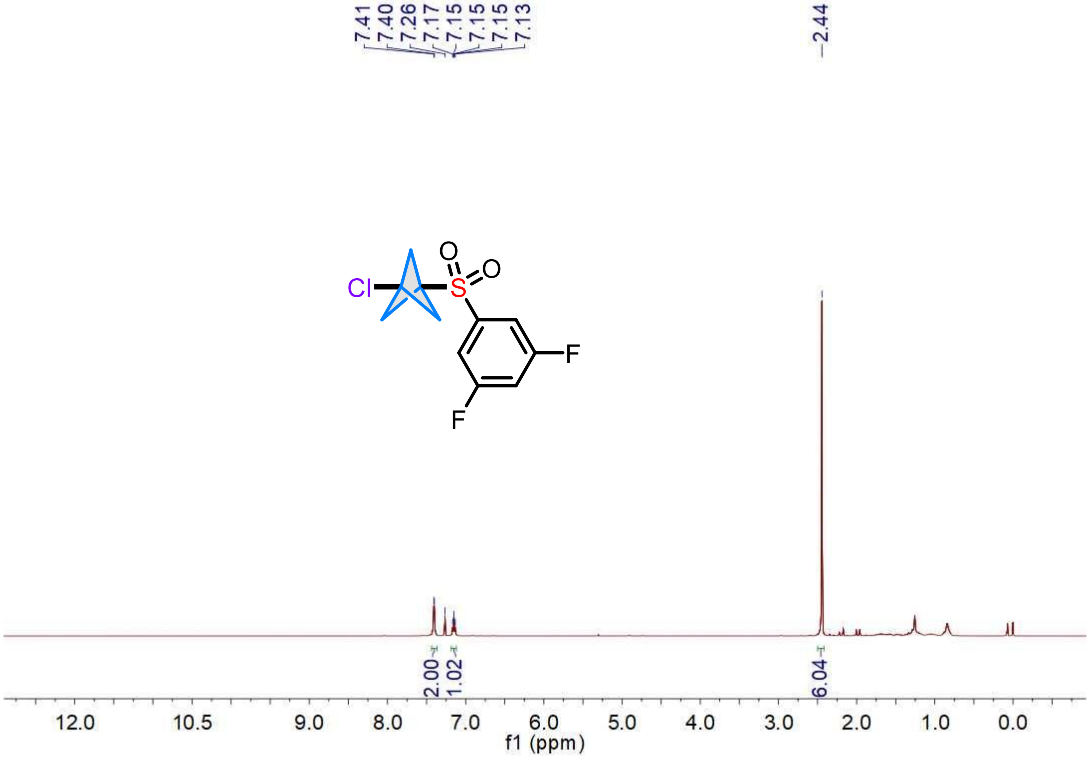

74 ^{13}C NMR (126 MHz, $CDCl_3$)

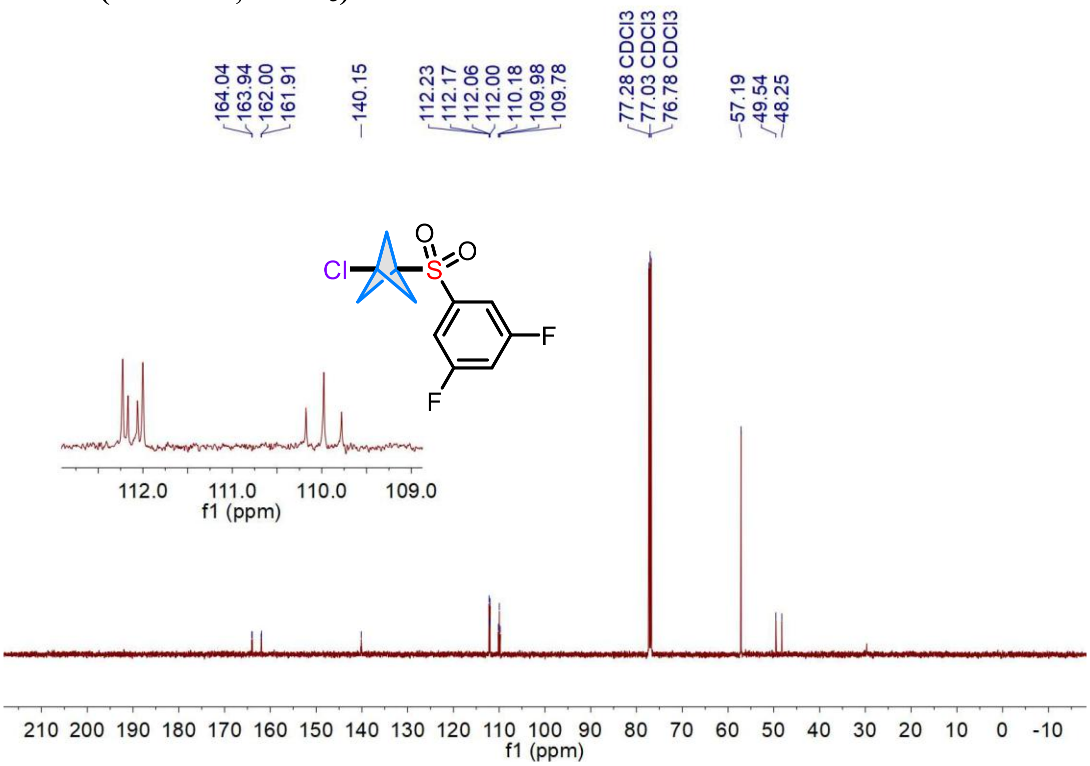

74 ^{19}F NMR (471 MHz, $CDCl_3$)

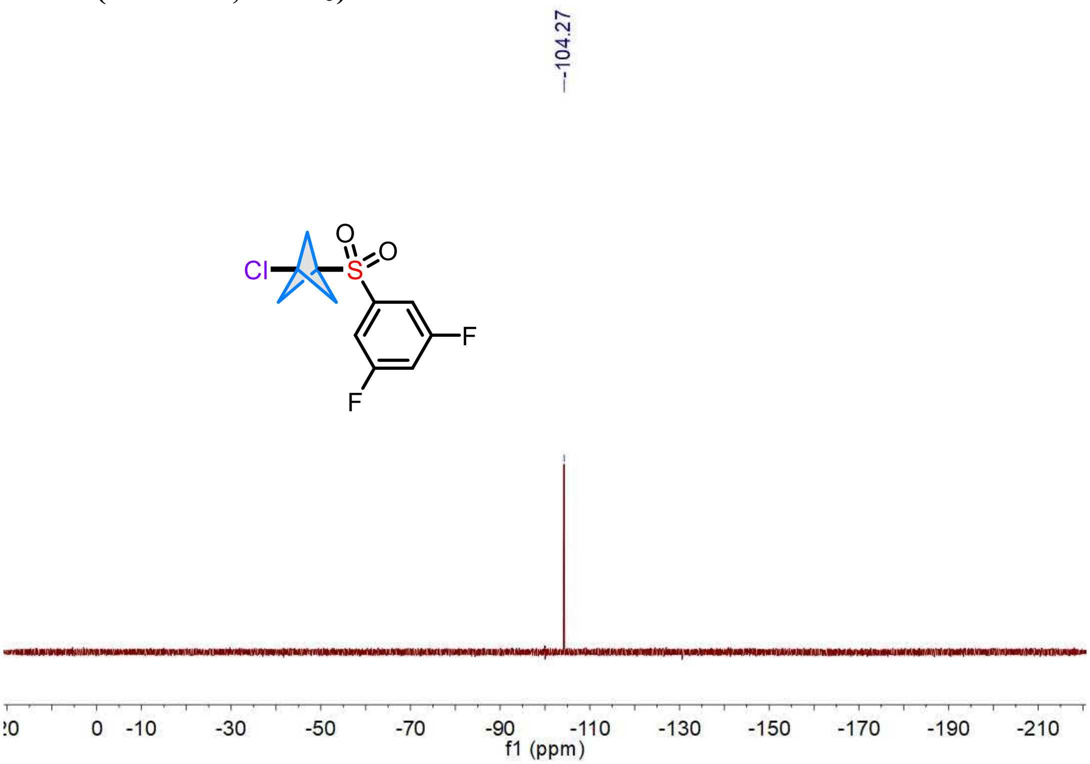

75 ^{1}H NMR (500 MHz, CDCl$_3$)

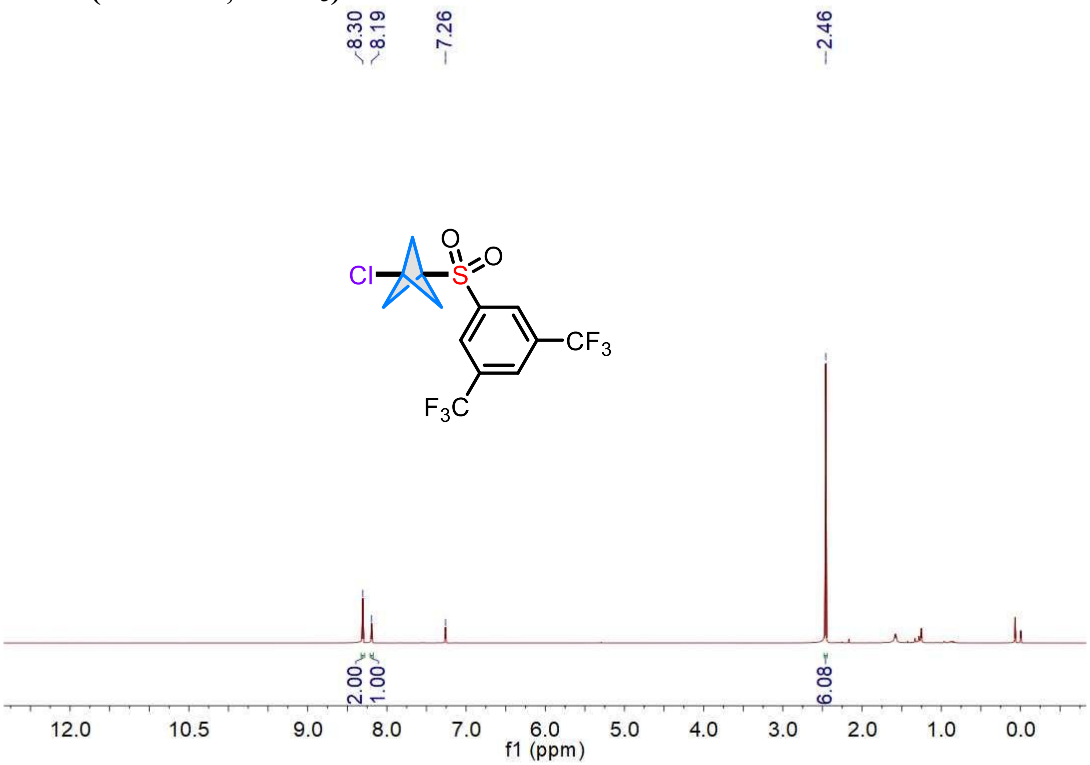

75 ^{13}C NMR (126 MHz, CDCl$_3$)

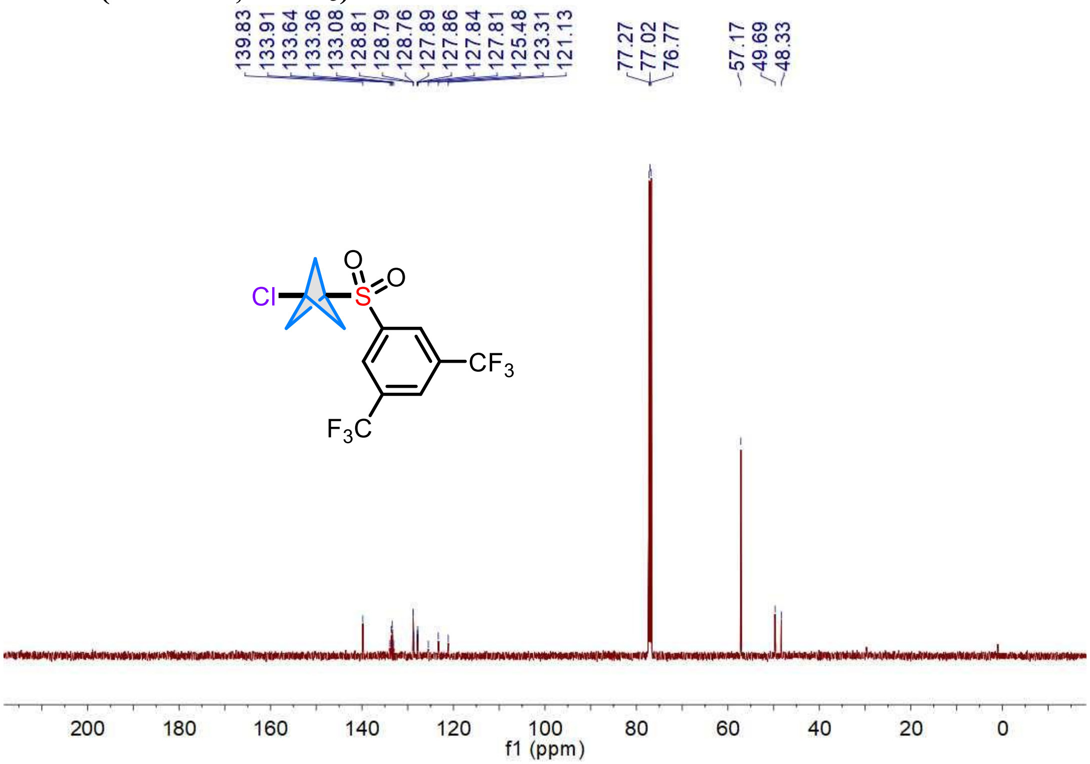

75 ^{19}F NMR (471 MHz, $CDCl_3$)

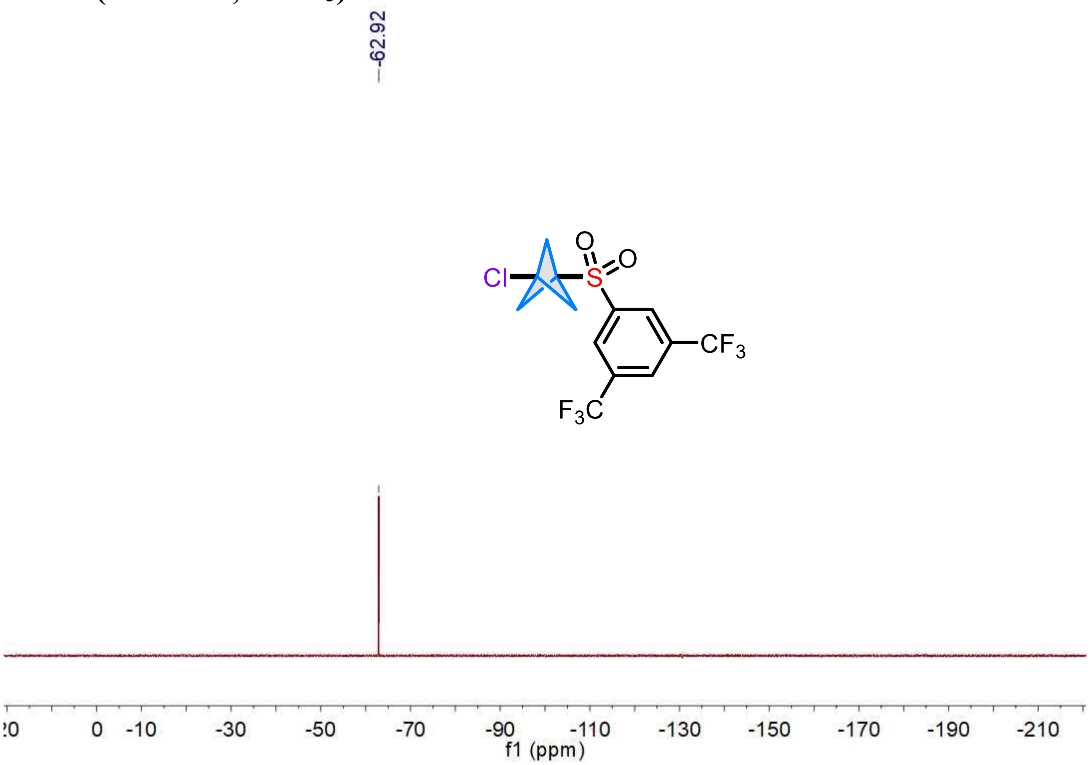

76 ^{1}H NMR (500 MHz, $CDCl_3$)

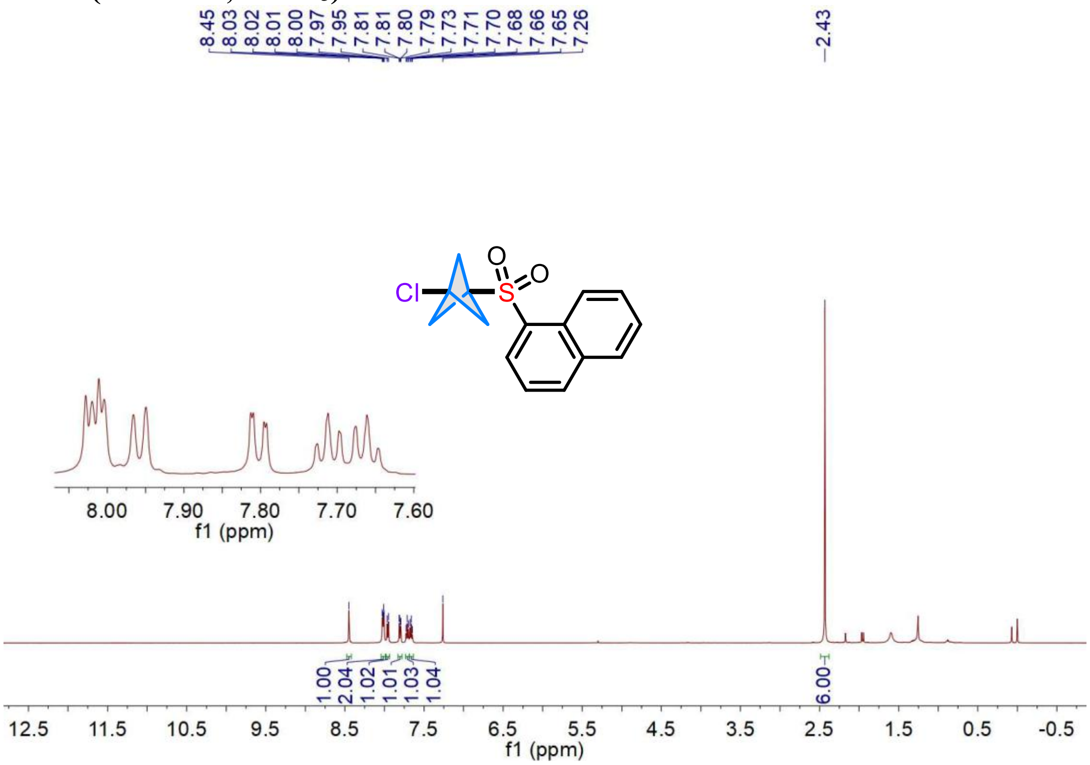

76 ^{13}C NMR (126 MHz, CDCl$_3$)

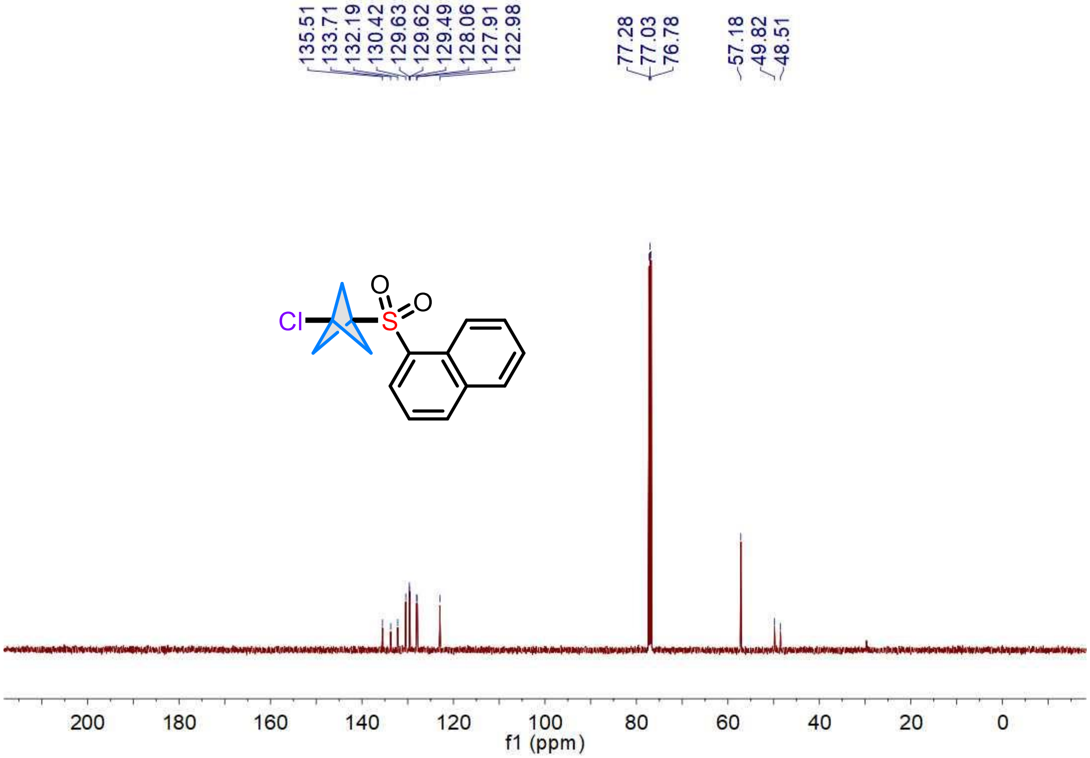

77 ^{1}H NMR (500 MHz, CDCl$_3$)

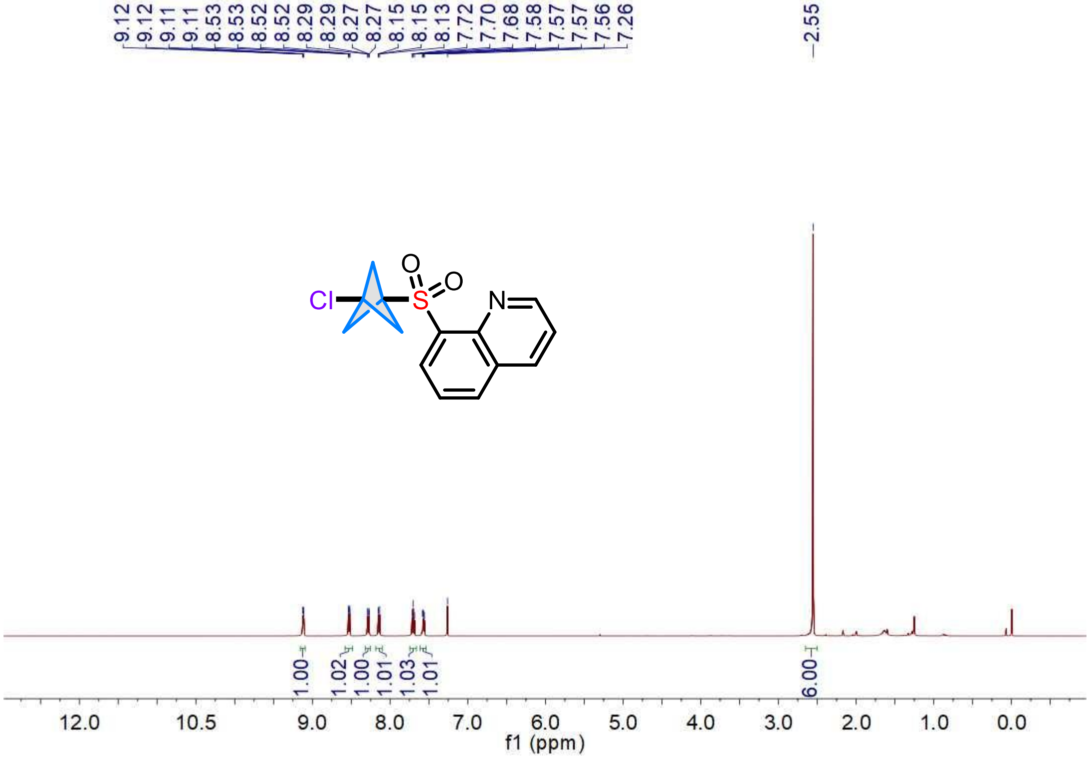

77 ^{13}C NMR (126 MHz, CDCl$_3$)

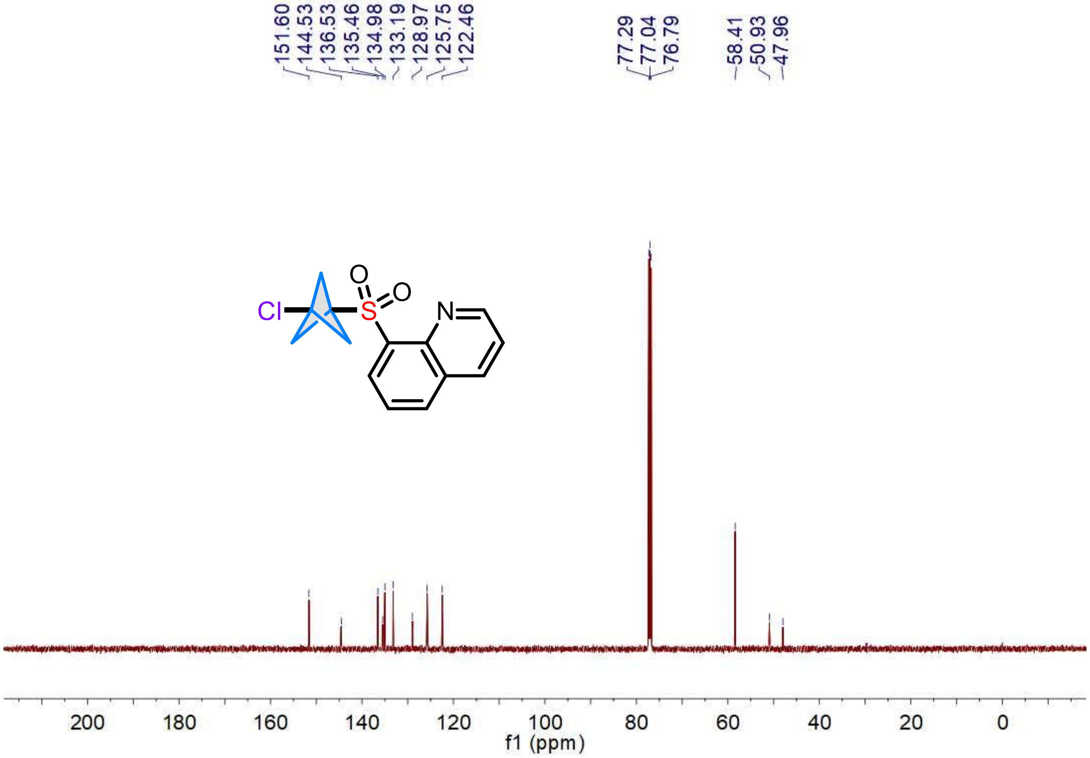

78 ^{1}H NMR (500 MHz, CDCl$_3$)

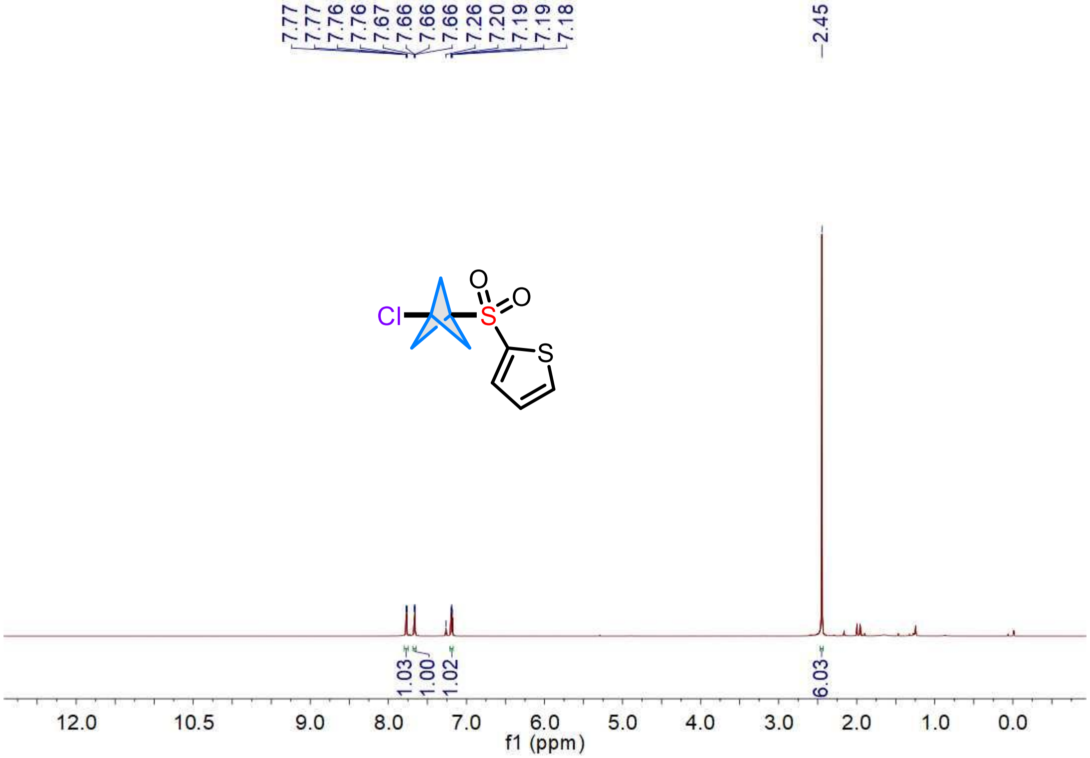

78 ^{13}C NMR (126 MHz, $CDCl_3$)

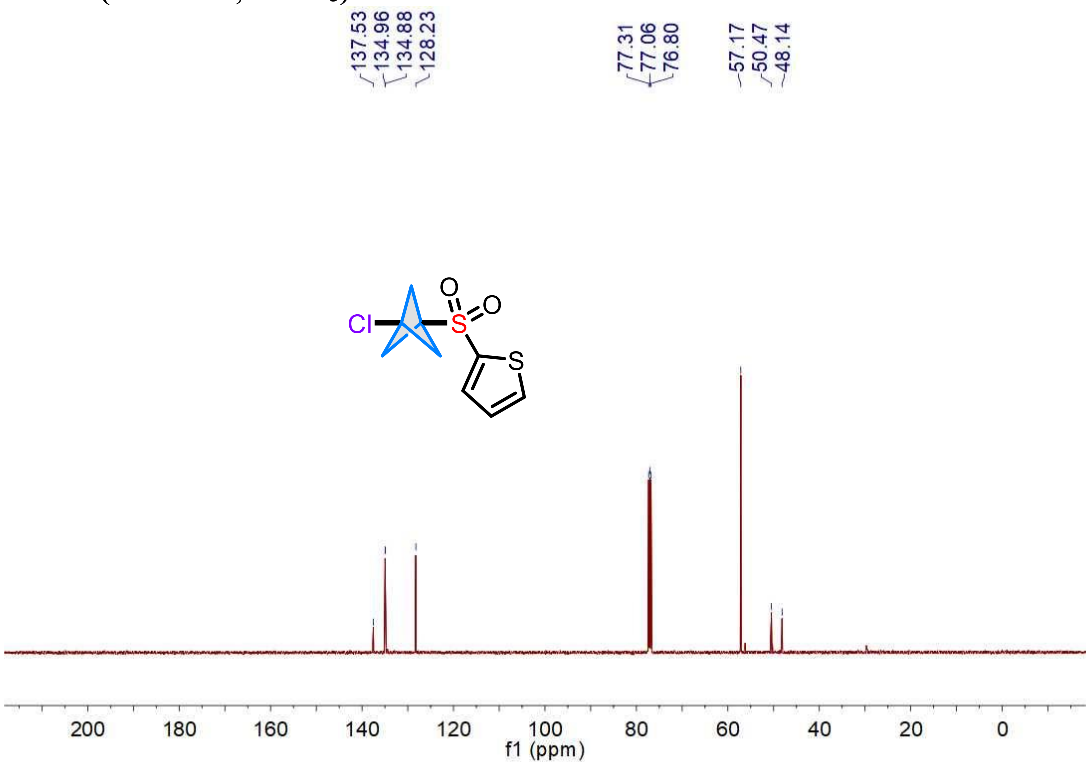

79 ^{1}H NMR (500 MHz, $CDCl_3$)

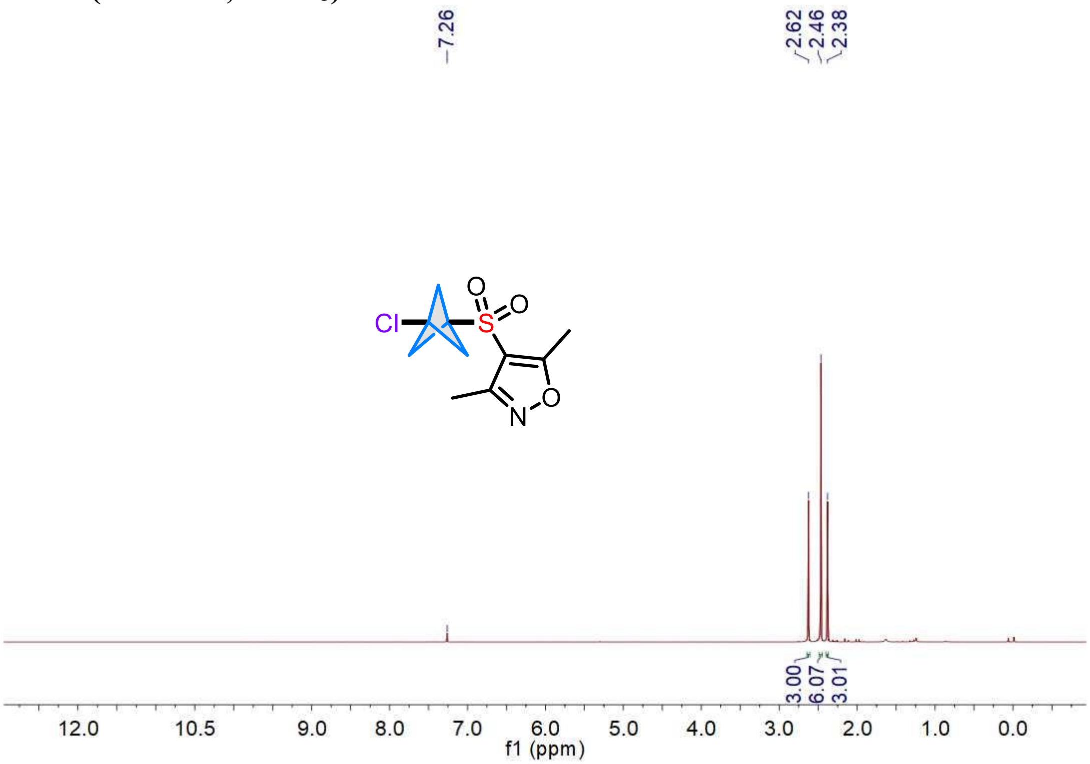

79 ^{13}C NMR (126 MHz, CDCl$_3$)

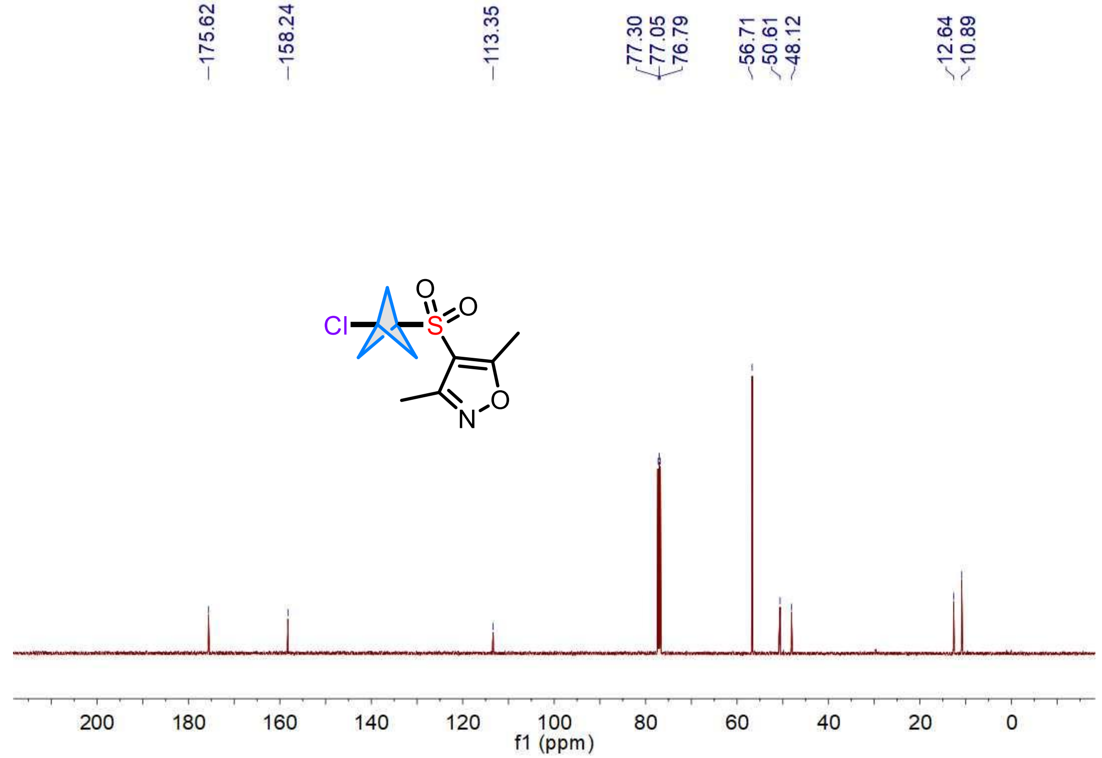

80 ^{1}H NMR (500 MHz, CDCl$_3$)

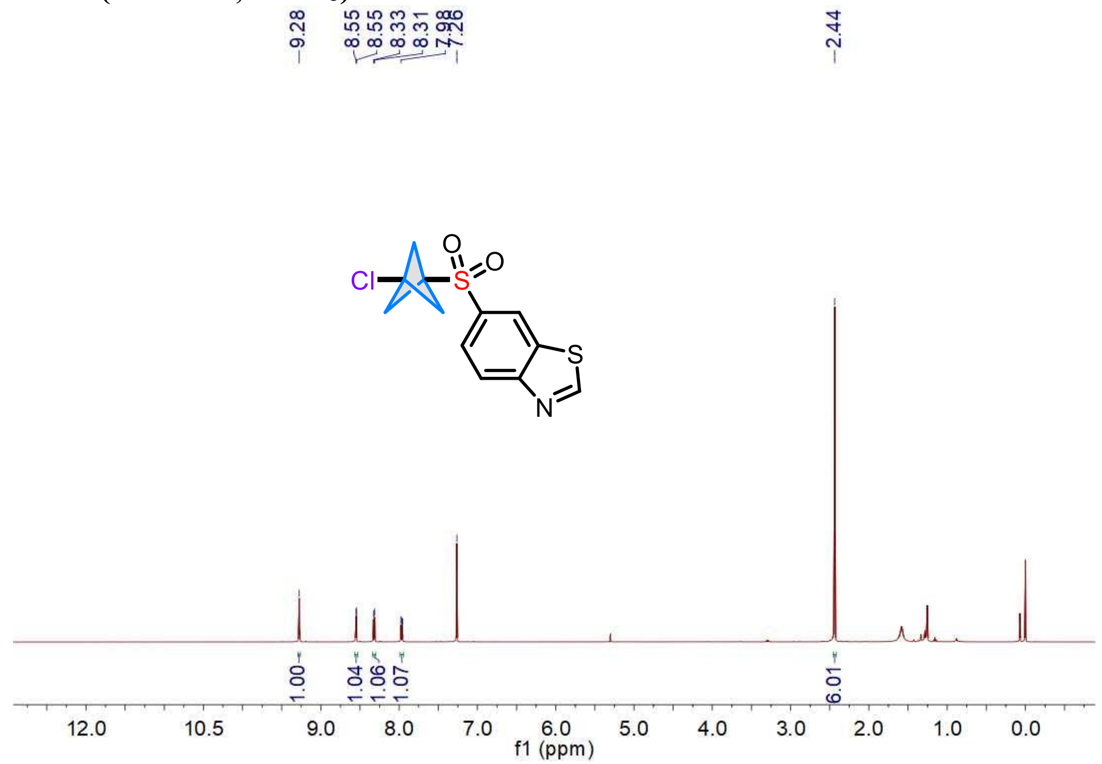

80 ^{13}C NMR (126 MHz, $CDCl_3$)

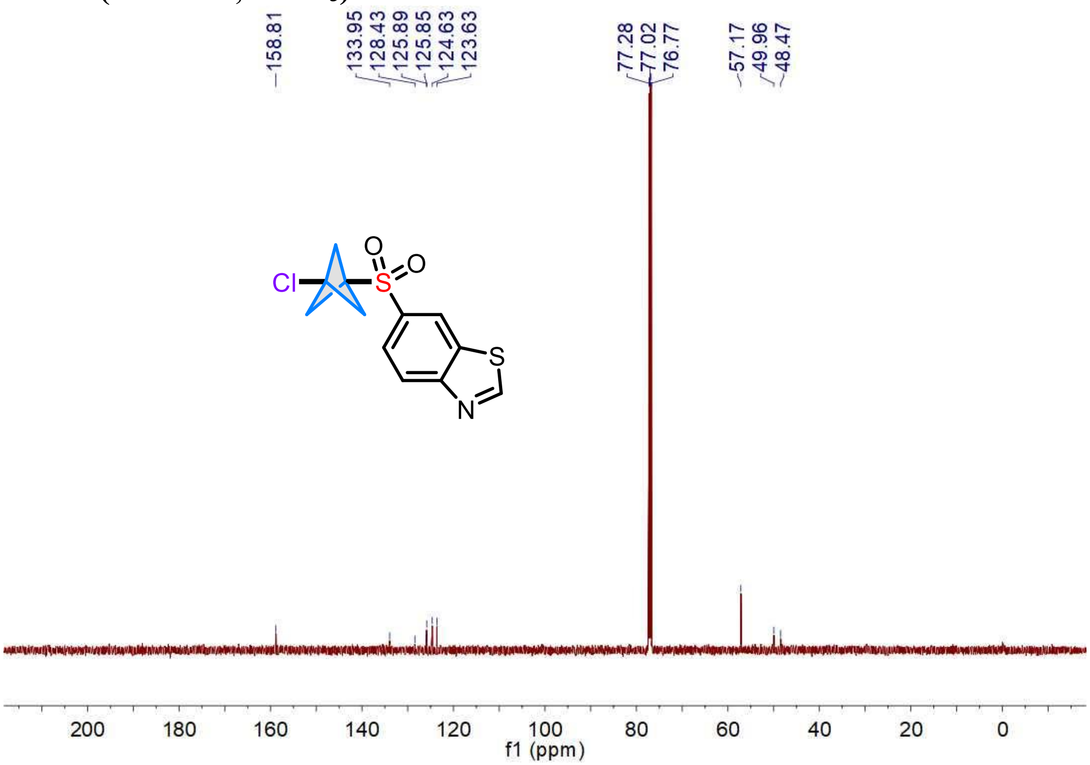

81 ^{1}H NMR (500 MHz, $CDCl_3$)

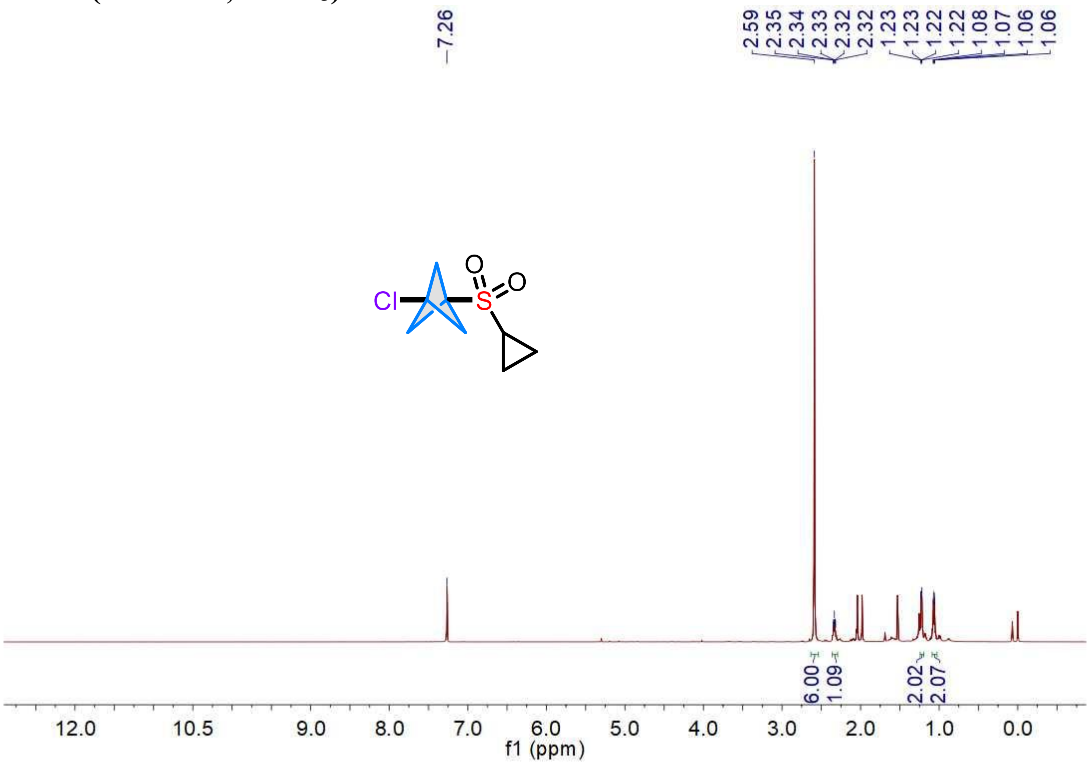

81 ^{13}C NMR (126 MHz, $CDCl_3$)

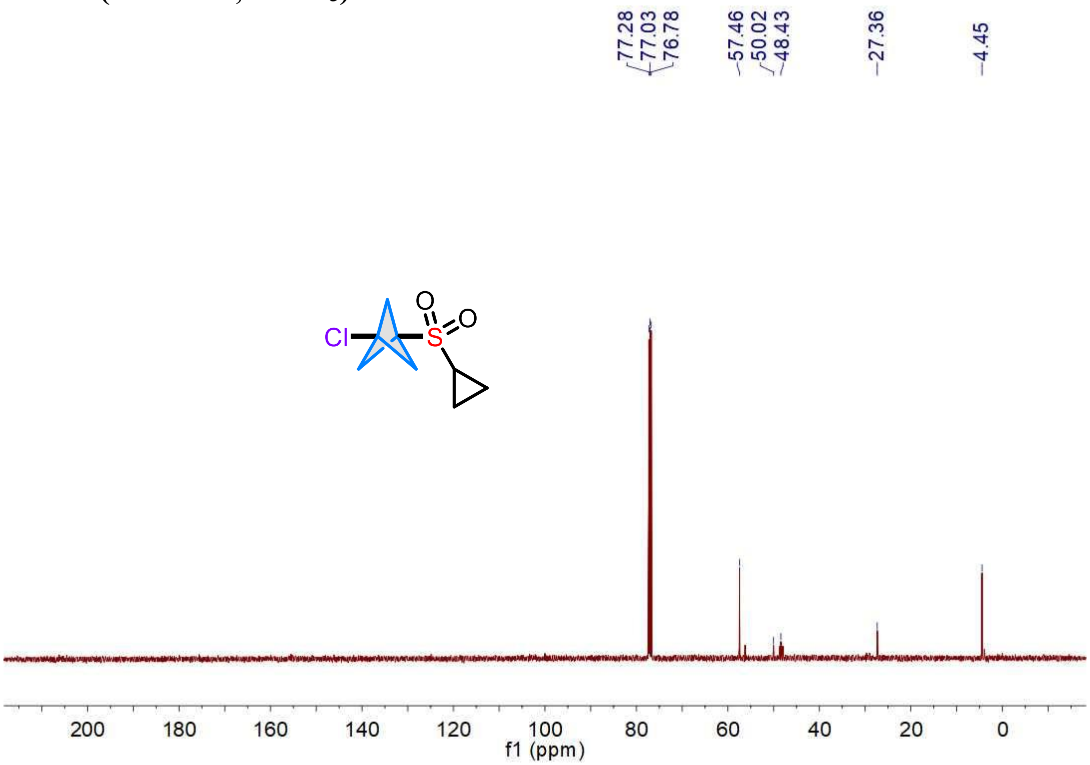

82 ^{1}H NMR (400 MHz, $CDCl_3$)

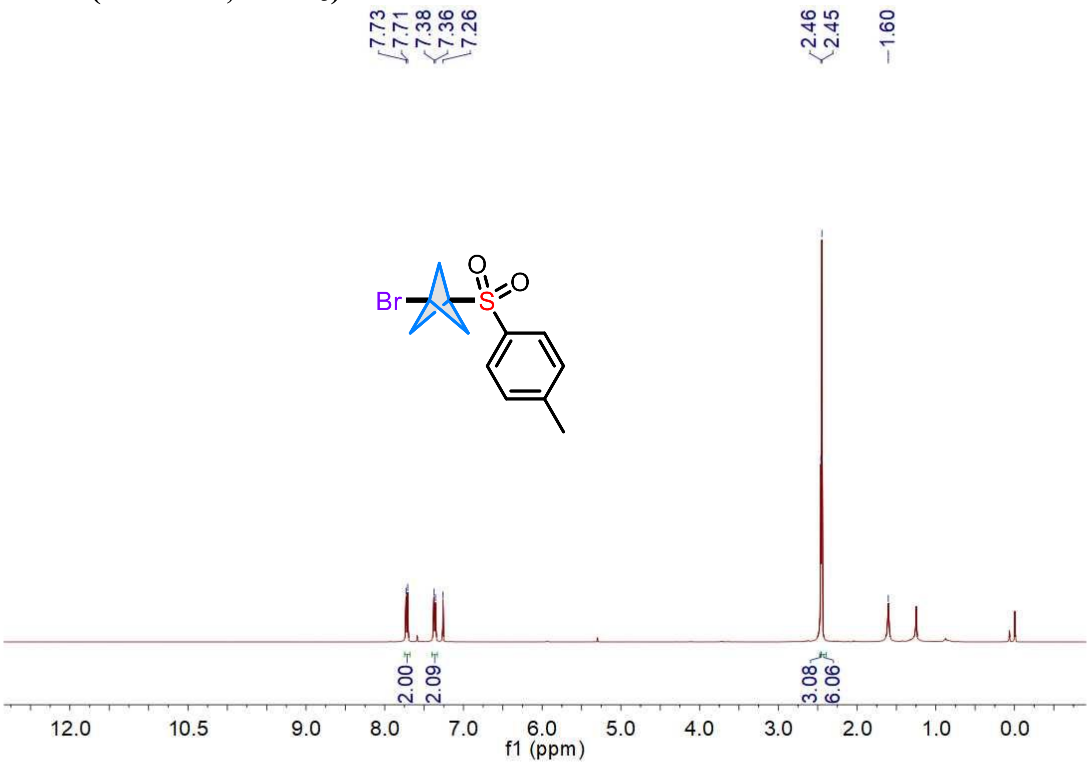

82 ^{13}C NMR (101 MHz, CDCl$_3$)

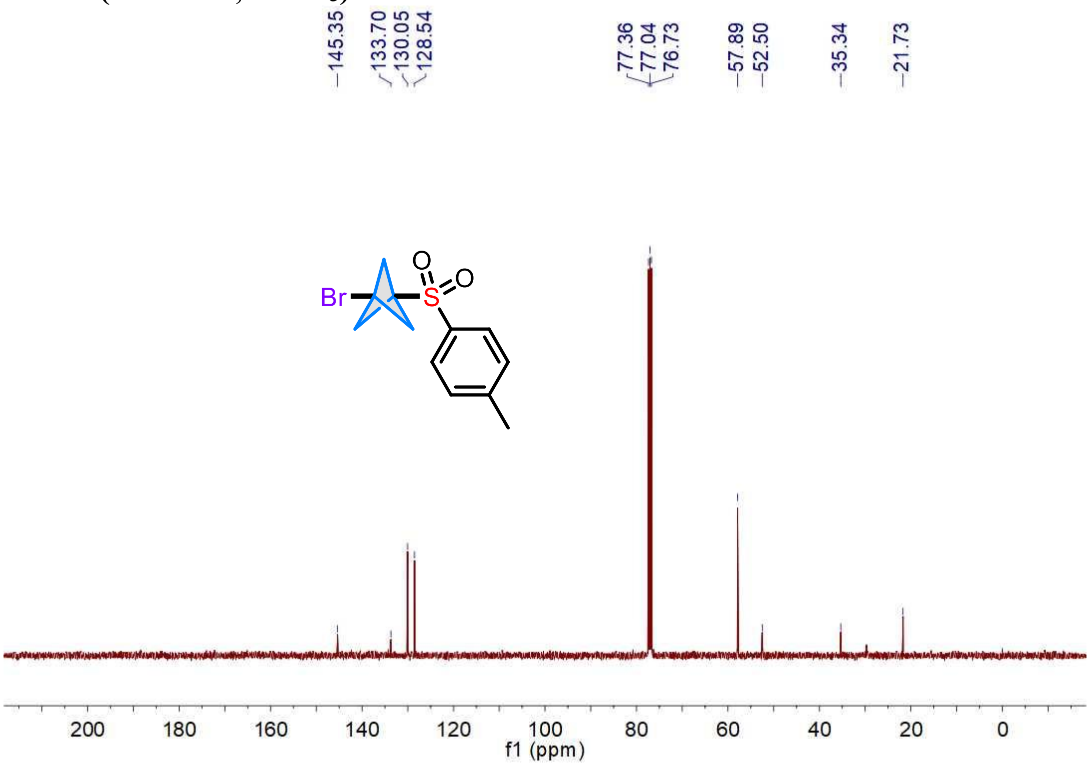

83 ^{1}H NMR (400 MHz, CDCl$_3$)

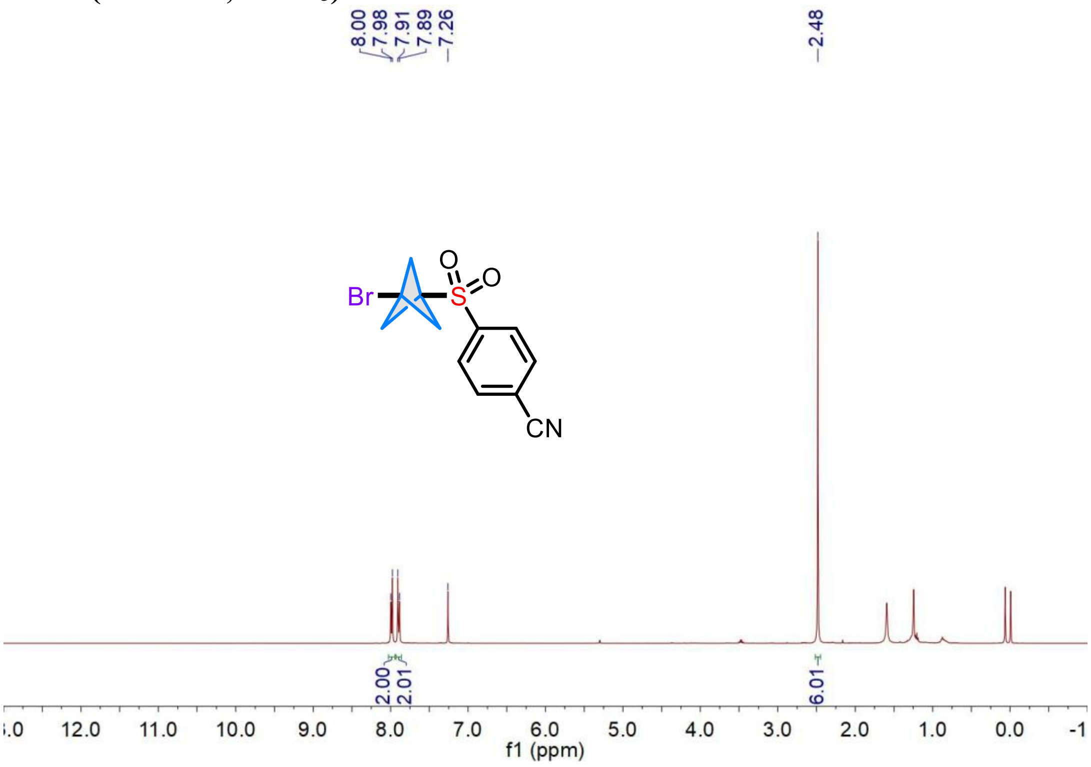

83 ^{13}C NMR (101 MHz, CDCl$_3$)

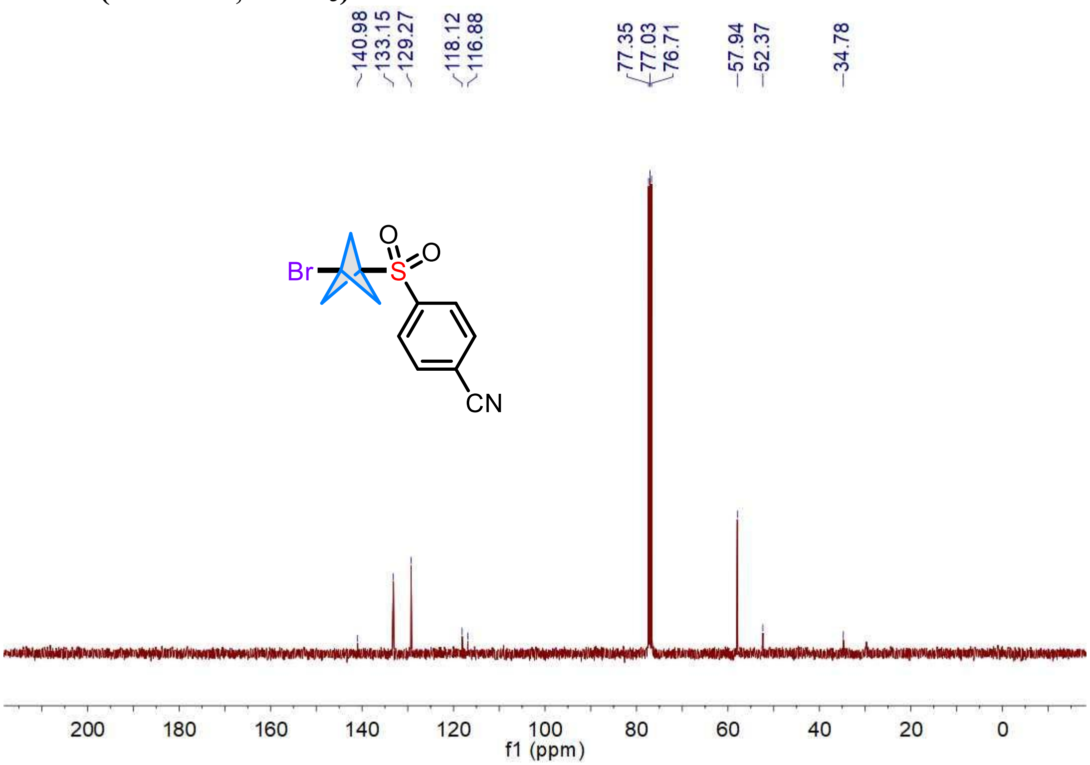

84 ^{1}H NMR (500 MHz, CDCl$_3$)

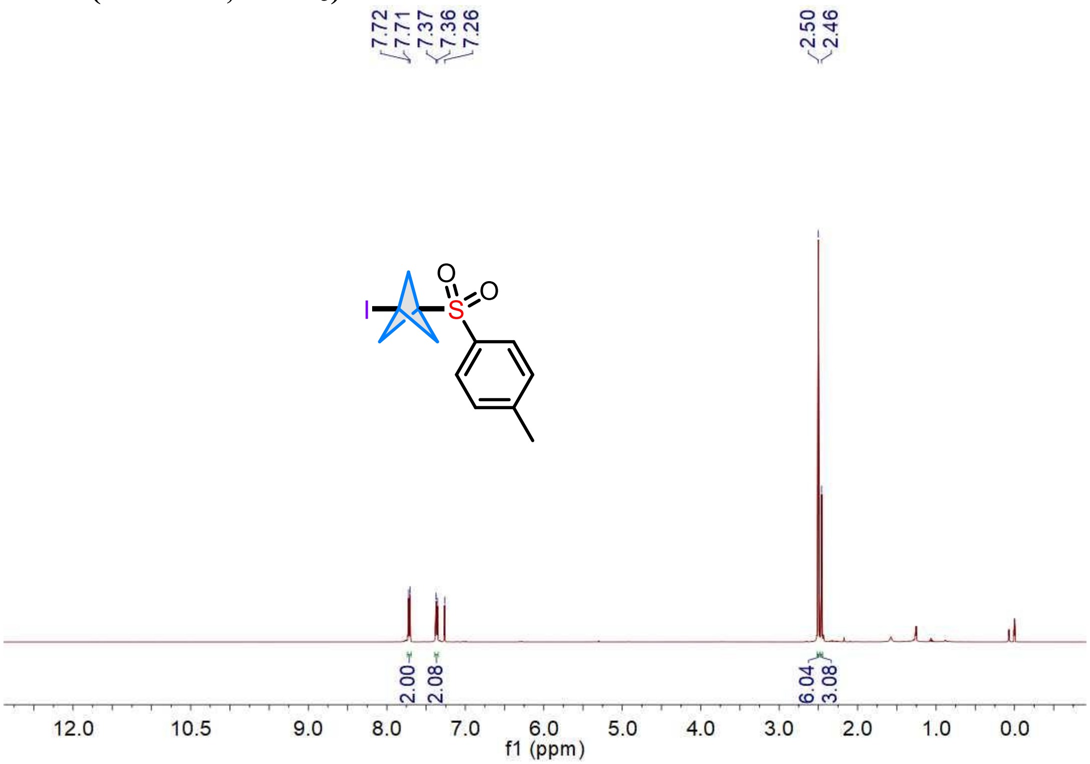

84 ^{13}C NMR (126 MHz, $CDCl_3$)

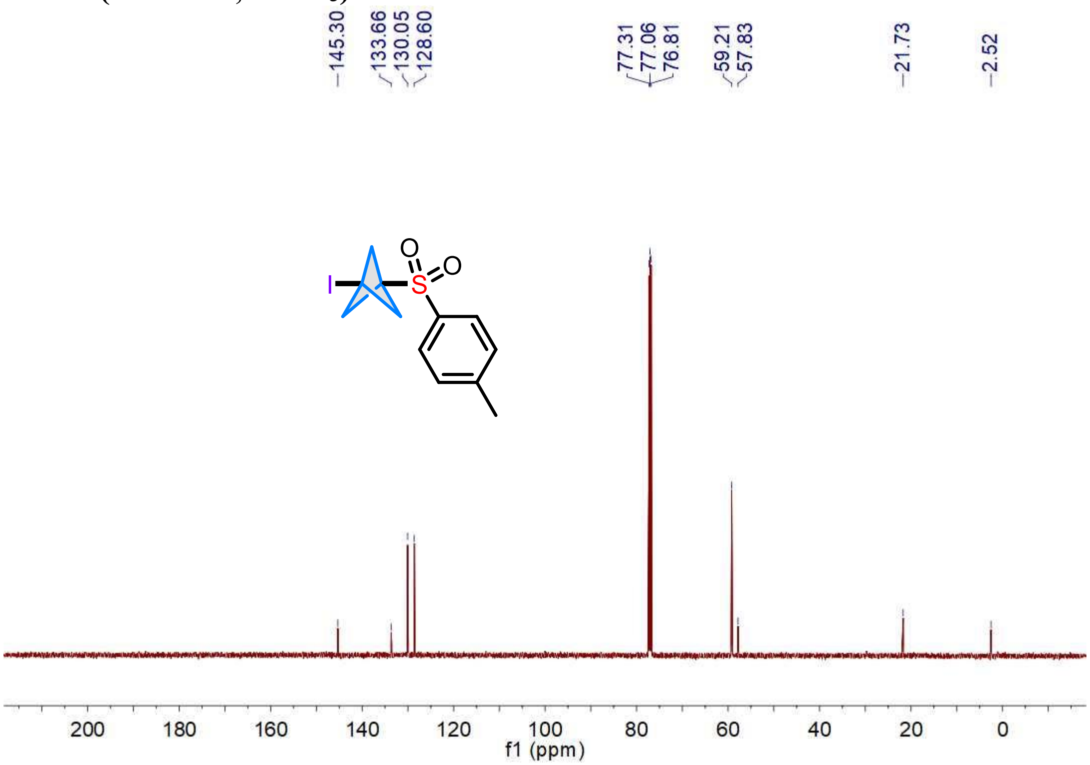

85 ^{1}H NMR (500 MHz, $CDCl_3$)

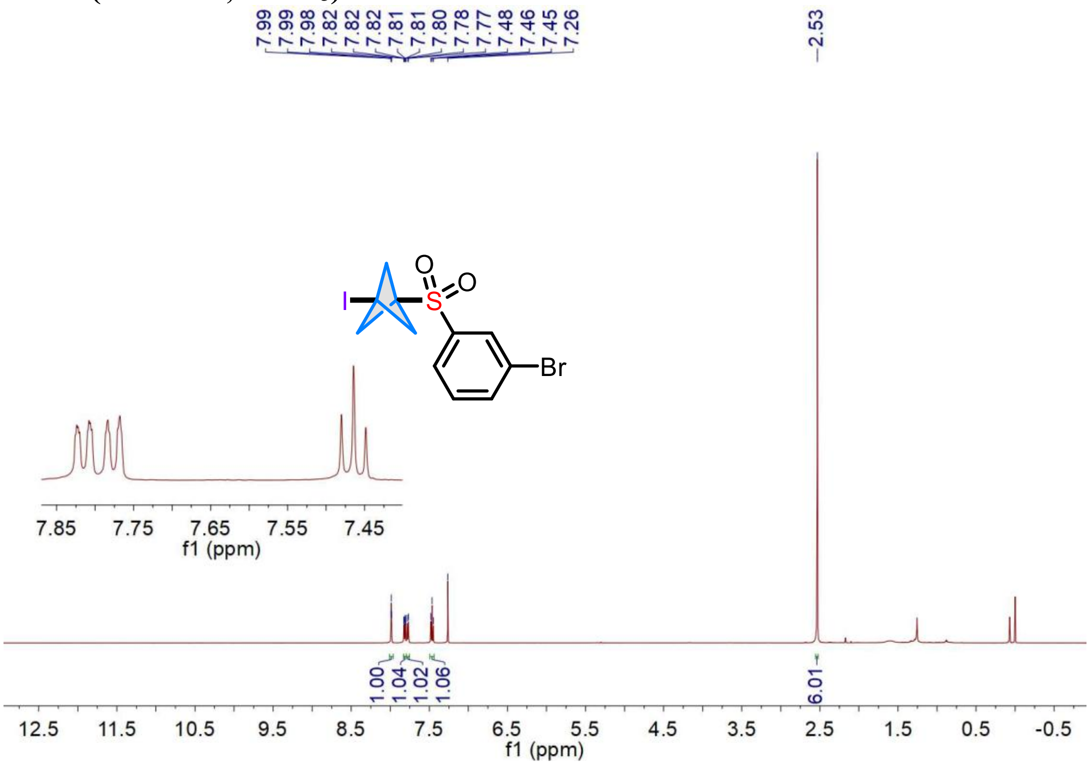

85 ^{13}C NMR (126 MHz, $CDCl_3$)

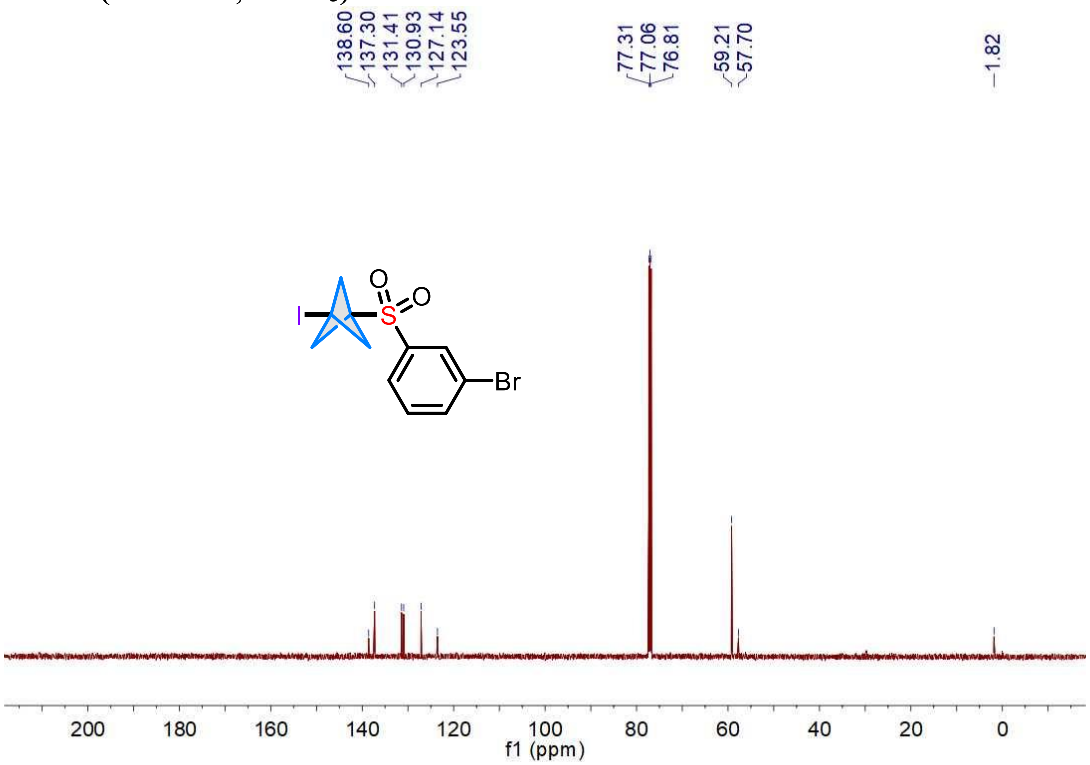

86 ^{1}H NMR (500 MHz, $CDCl_3$)

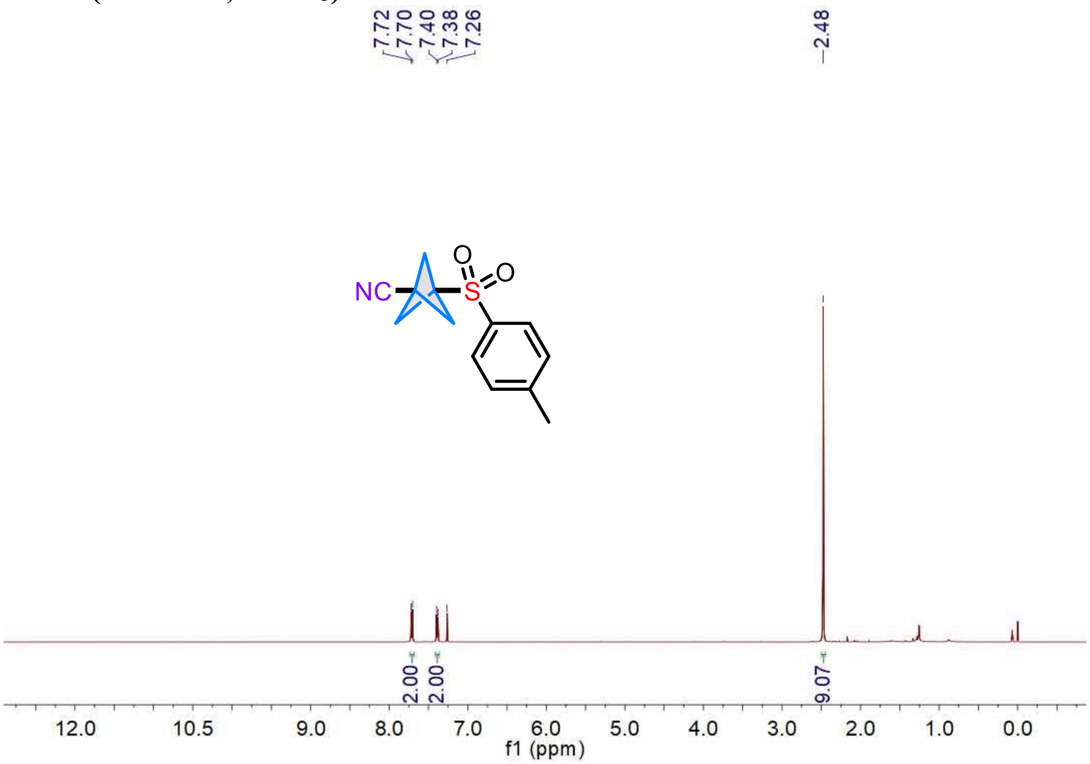

86 ^{13}C NMR (126 MHz, CDCl$_3$)

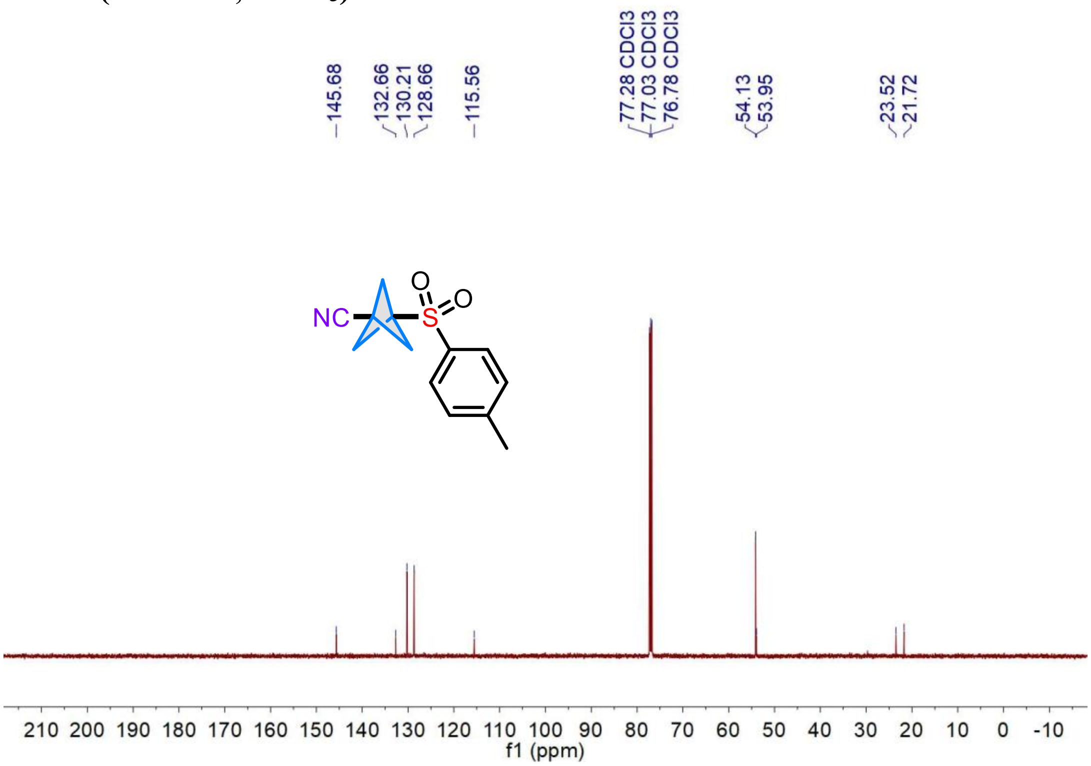

87 ^{1}H NMR (500 MHz, CDCl$_3$)

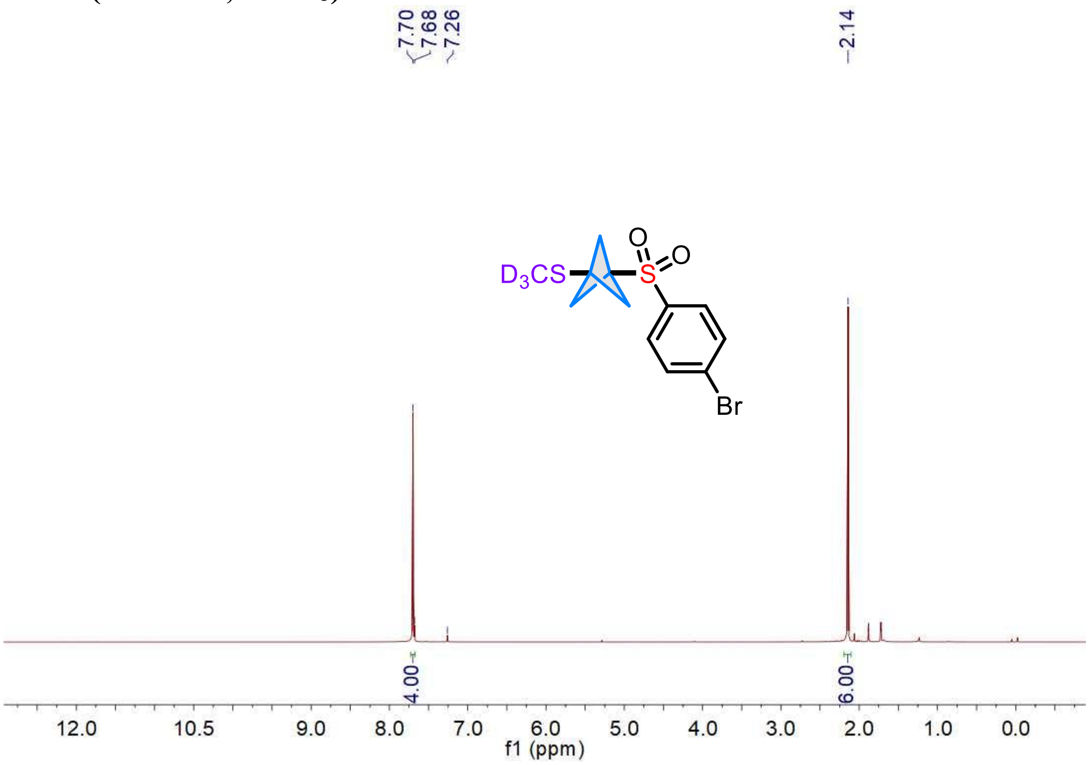

87 ^{13}C NMR (126 MHz, CDCl$_3$)

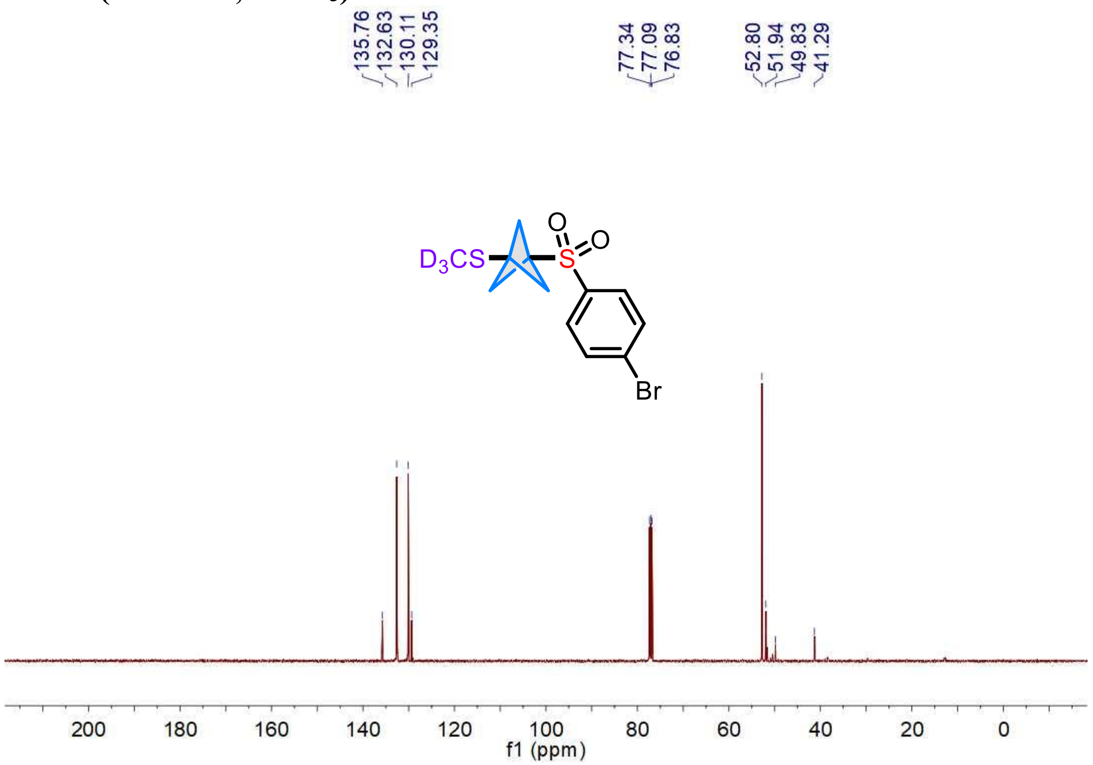

88 ^{1}H NMR (500 MHz, CDCl$_3$)

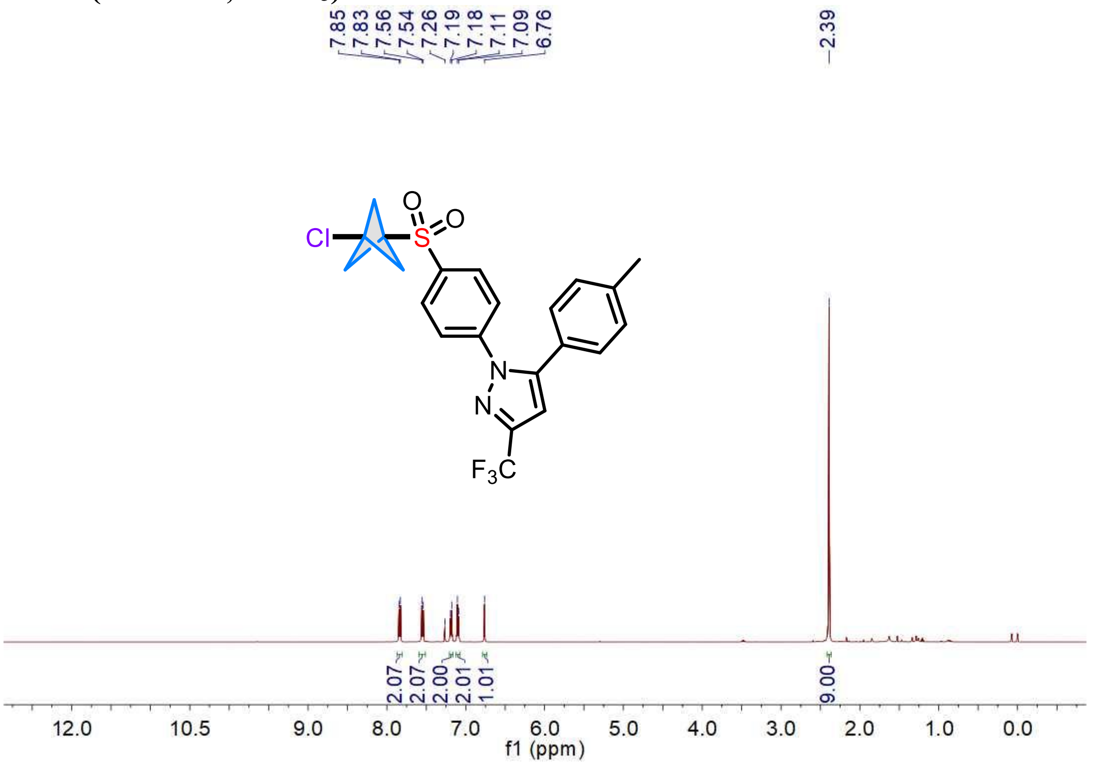

88 ^{13}C NMR (126 MHz, $CDCl_3$)

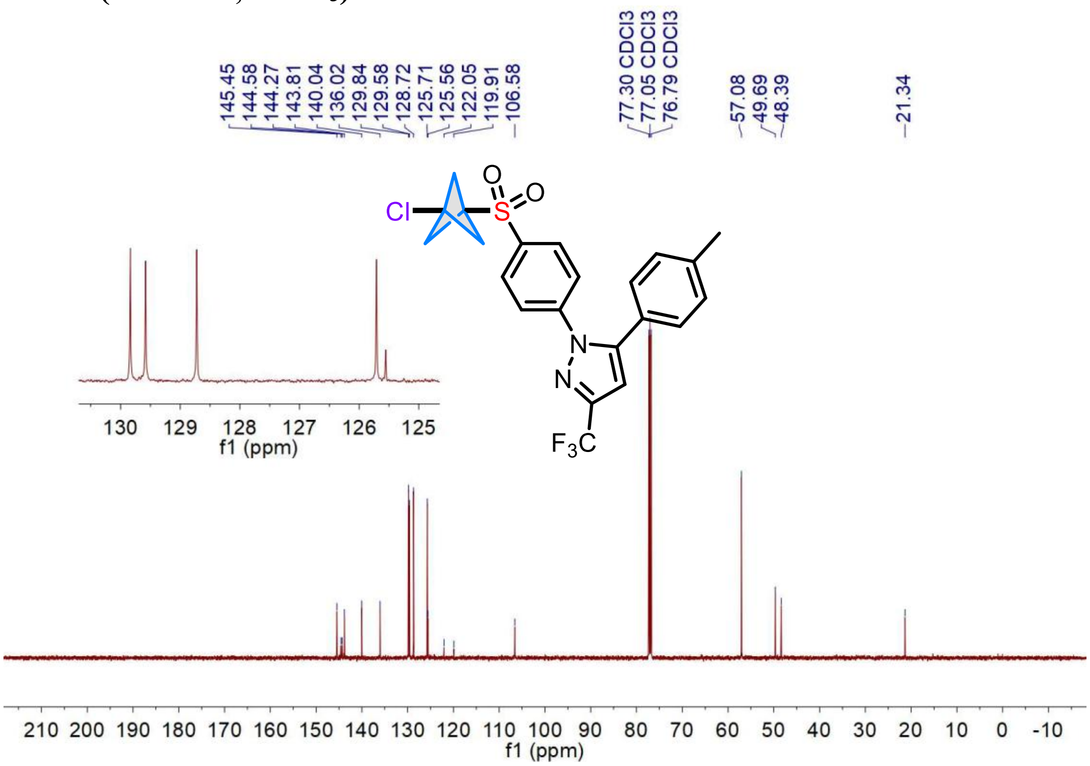

88 ^{19}F NMR (471 MHz, $CDCl_3$)

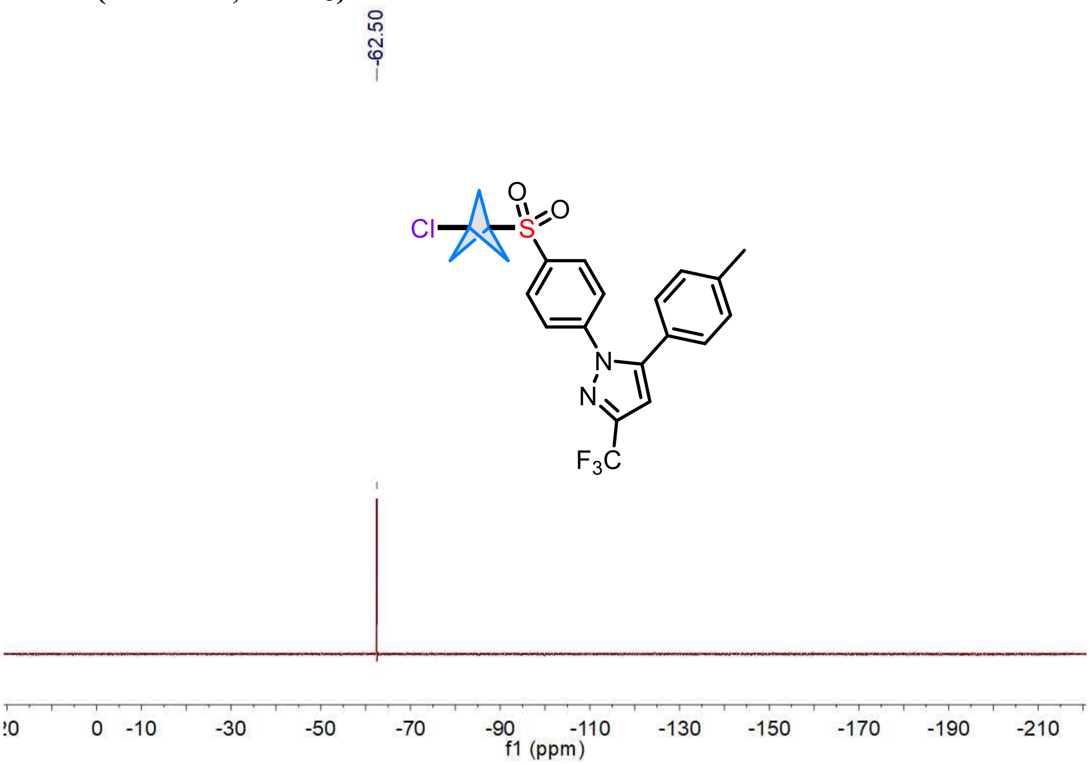

89 ^{1}H NMR (500 MHz, CDCl$_3$)

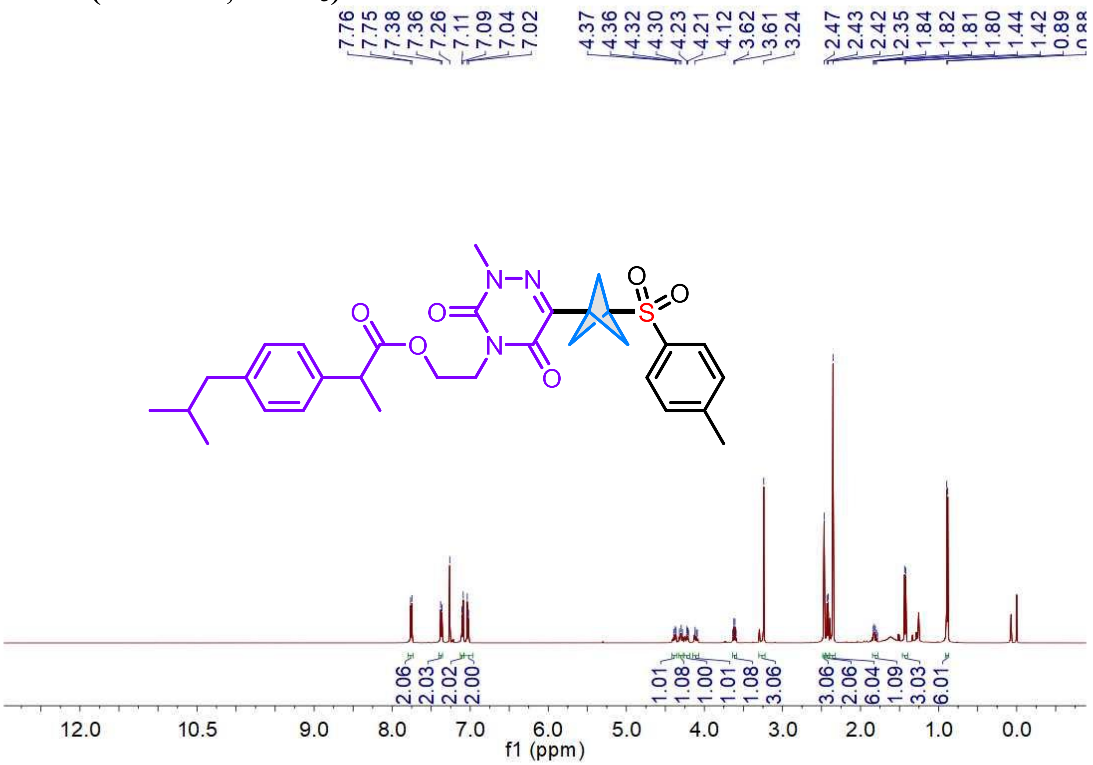

89 ^{13}C NMR (126 MHz, CDCl$_3$)

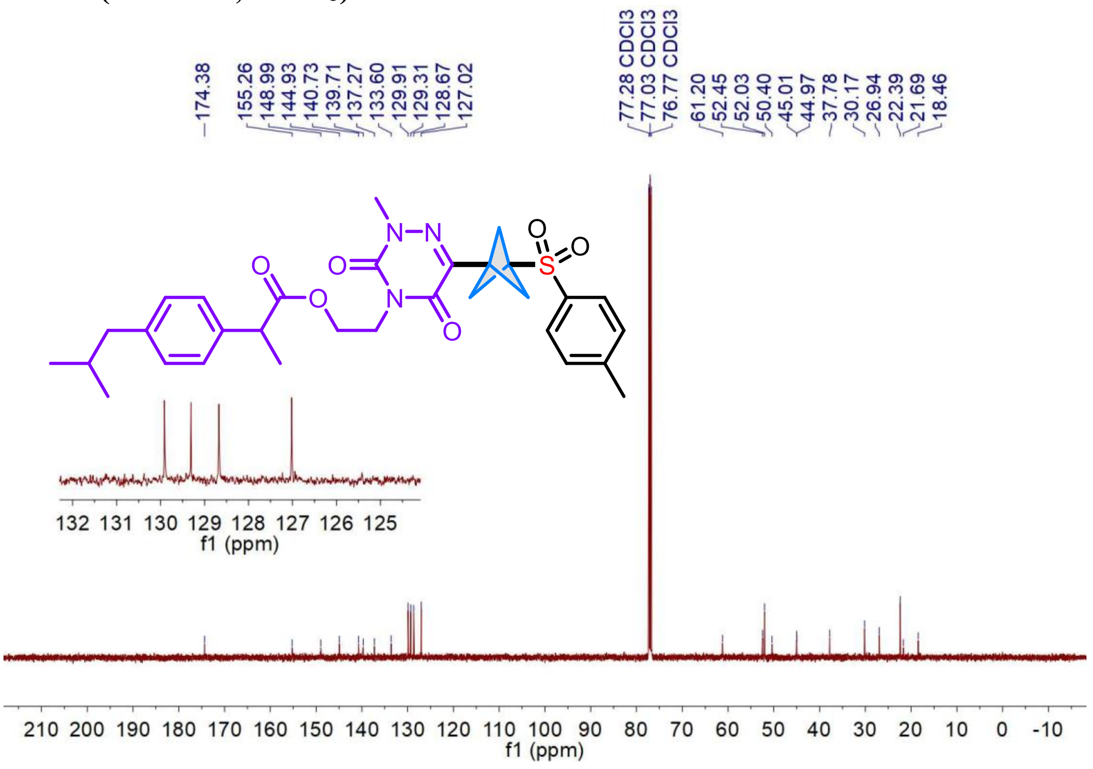

90 ^{1}H NMR (500 MHz, $CDCl_3$)

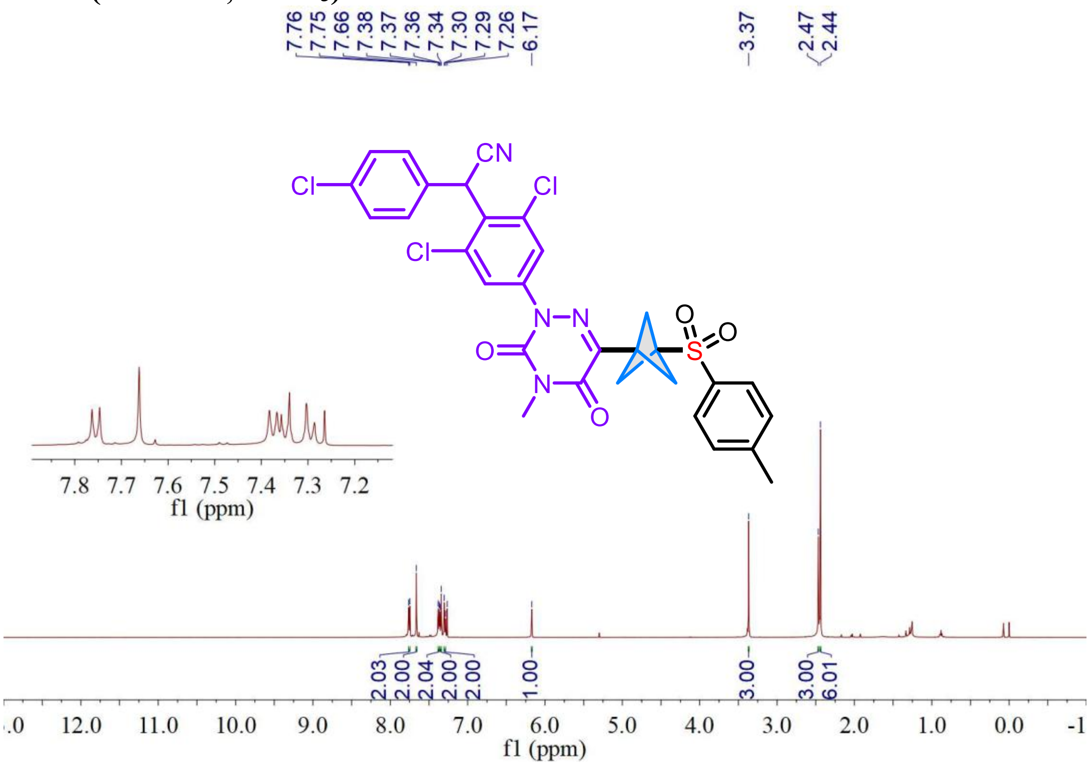

90 ^{13}C NMR (126 MHz, $CDCl_3$)

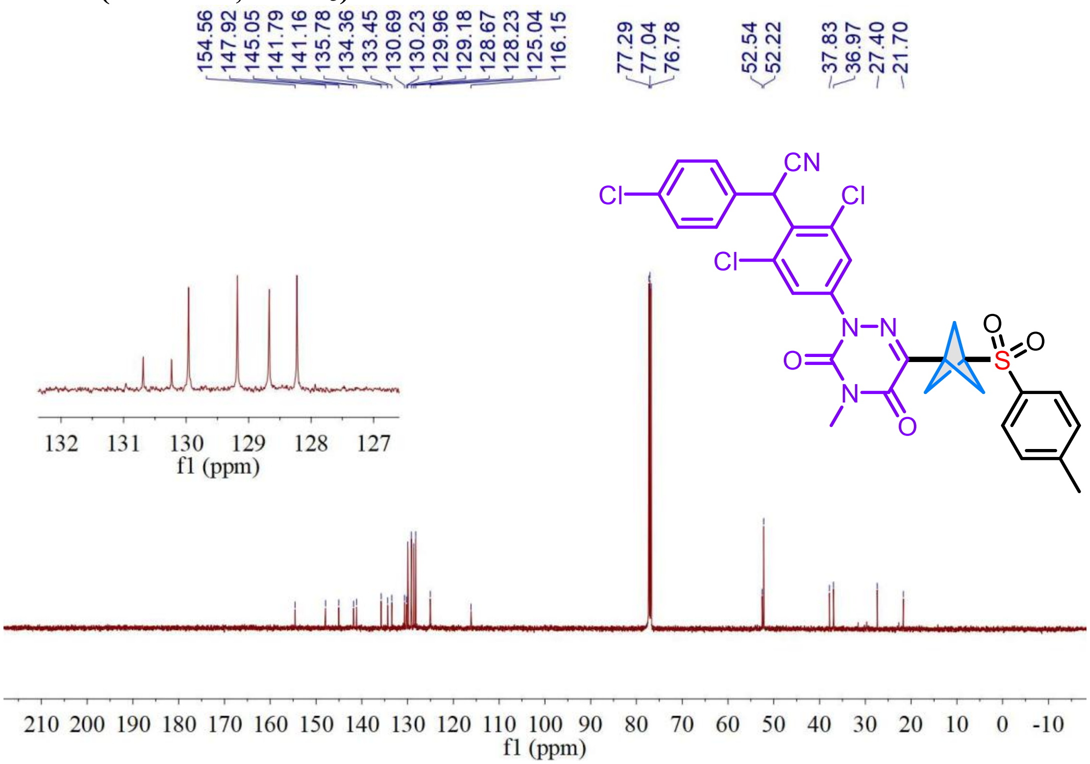

91 ^{1}H NMR (500 MHz, $CDCl_3$)

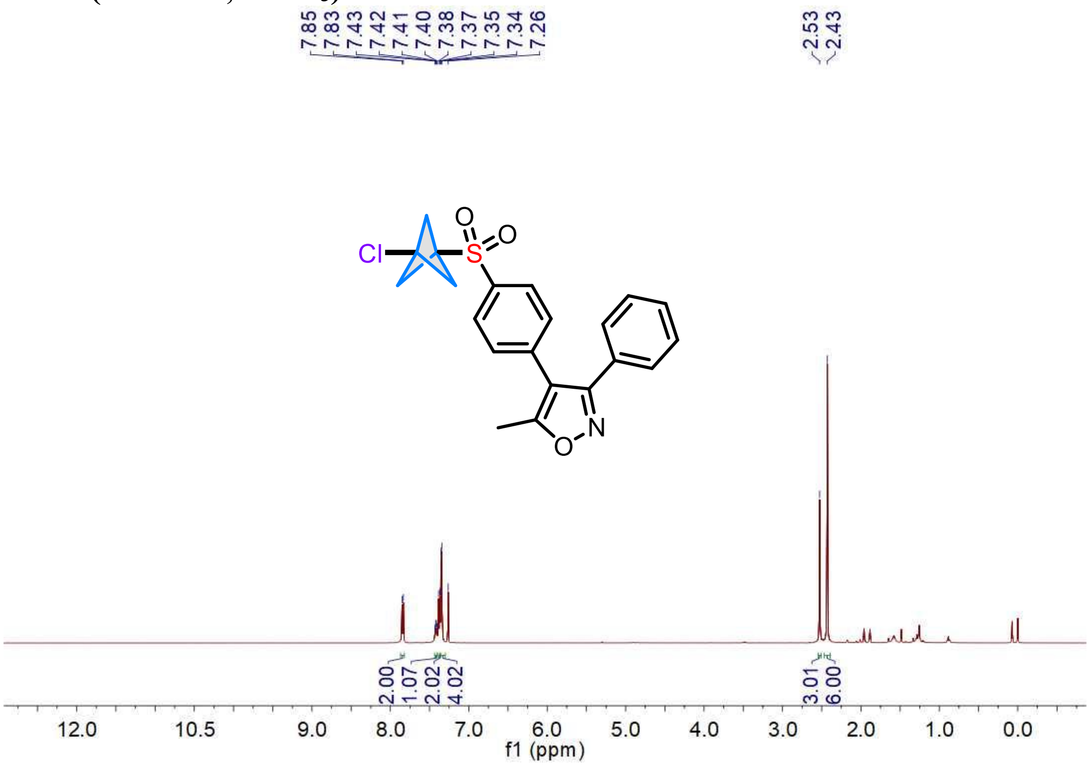

91 ^{13}C NMR (126 MHz, $CDCl_3$)

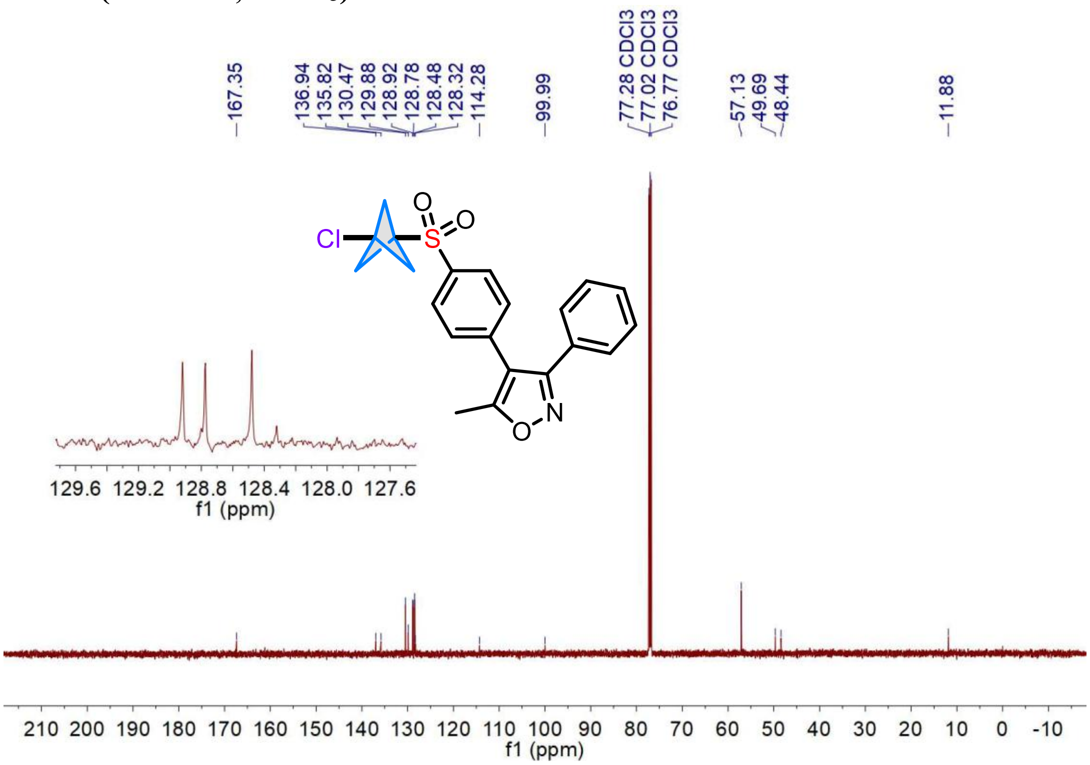

92 ^{1}H NMR (500 MHz, $CDCl_3$)

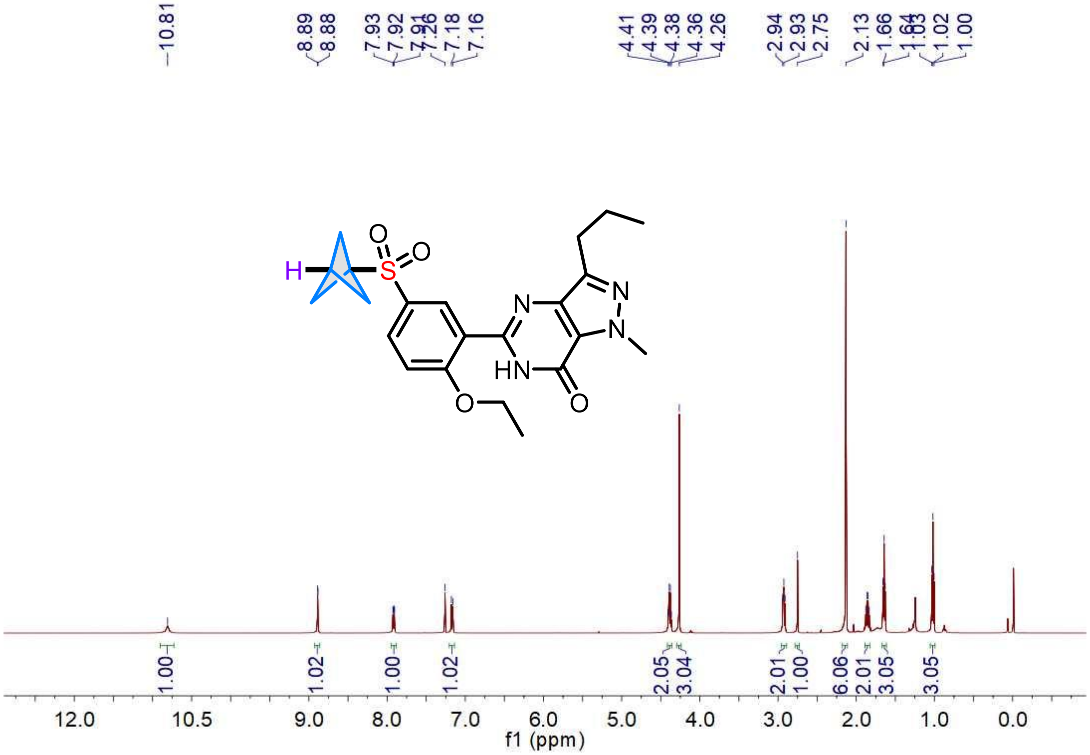

92 ^{13}C NMR (126 MHz, $CDCl_3$)

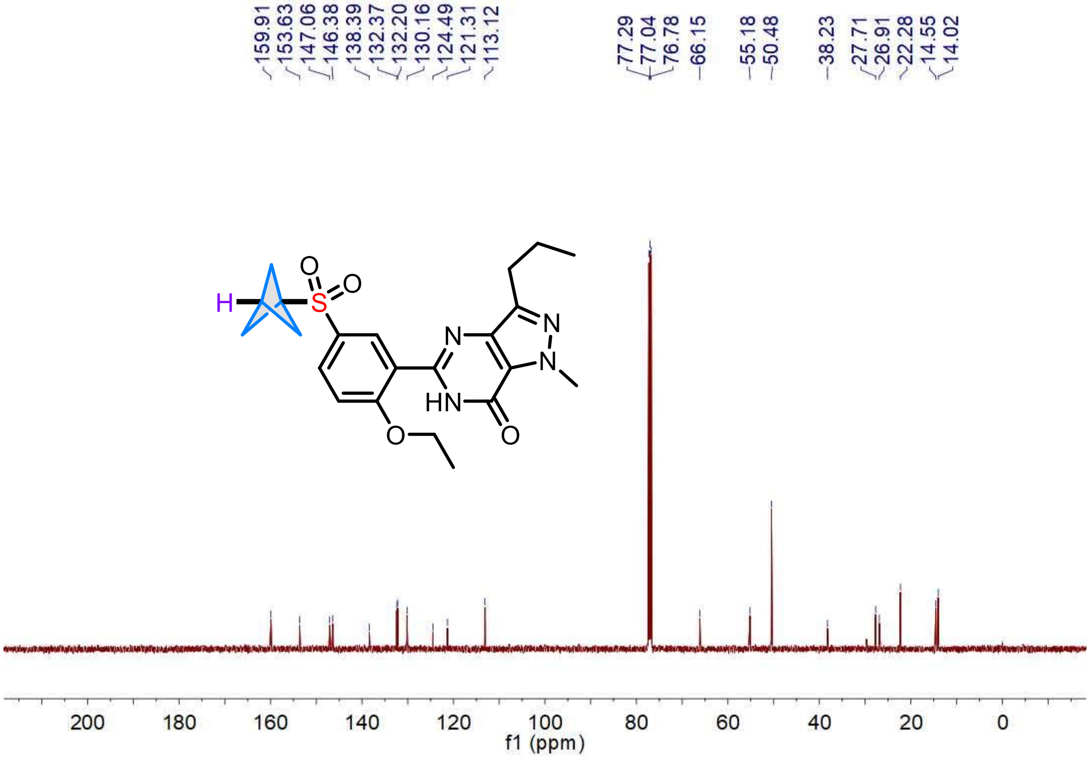

93 ^{1}H NMR (500 MHz, CDCl$_3$)

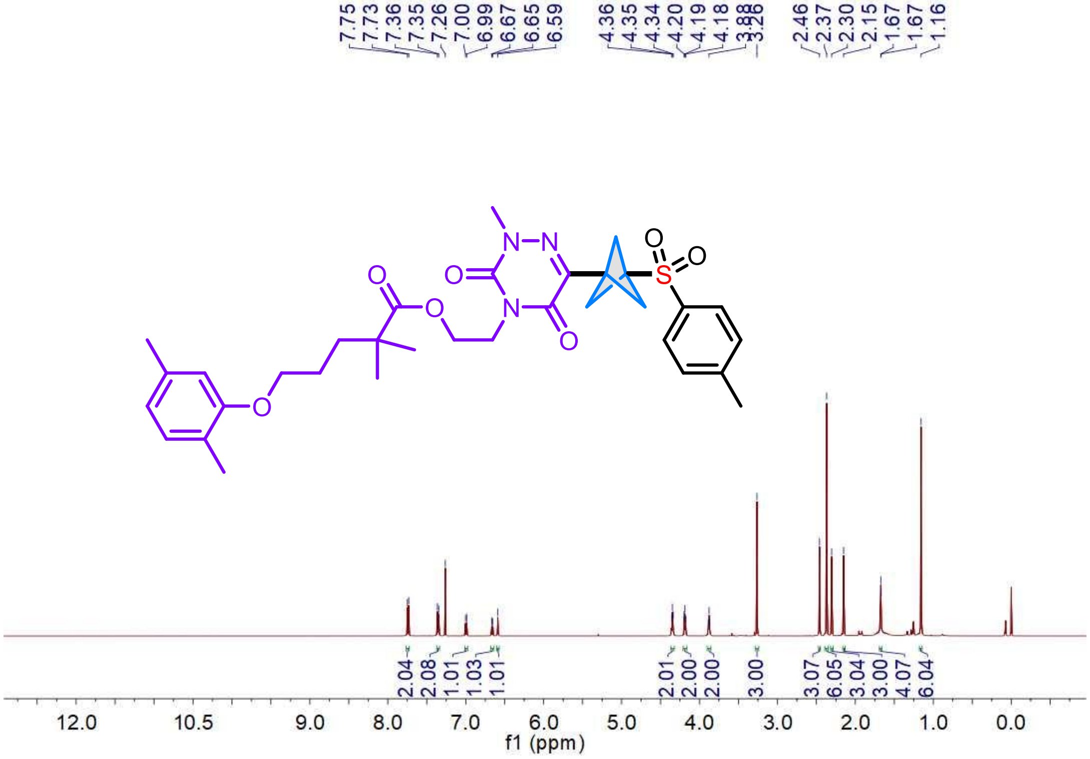

93 ^{13}C NMR (126 MHz, CDCl$_3$)

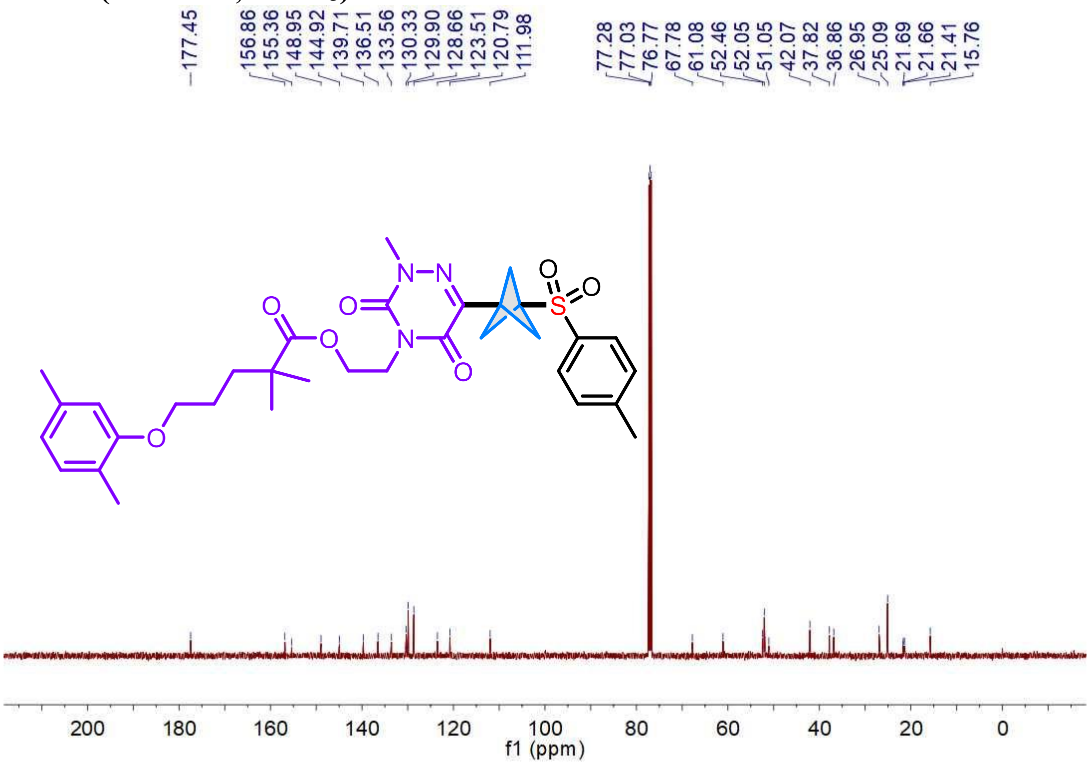

94 ^{1}H NMR (500 MHz, $CDCl_3$)

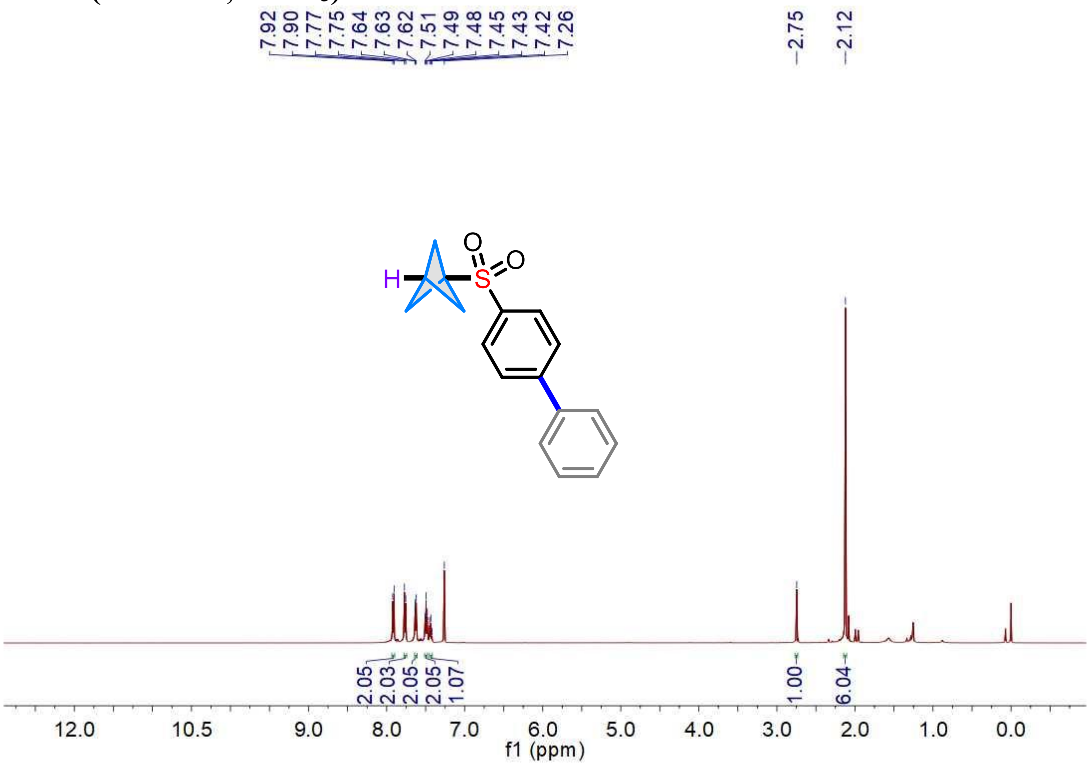

94 ^{13}C NMR (126 MHz, $CDCl_3$)

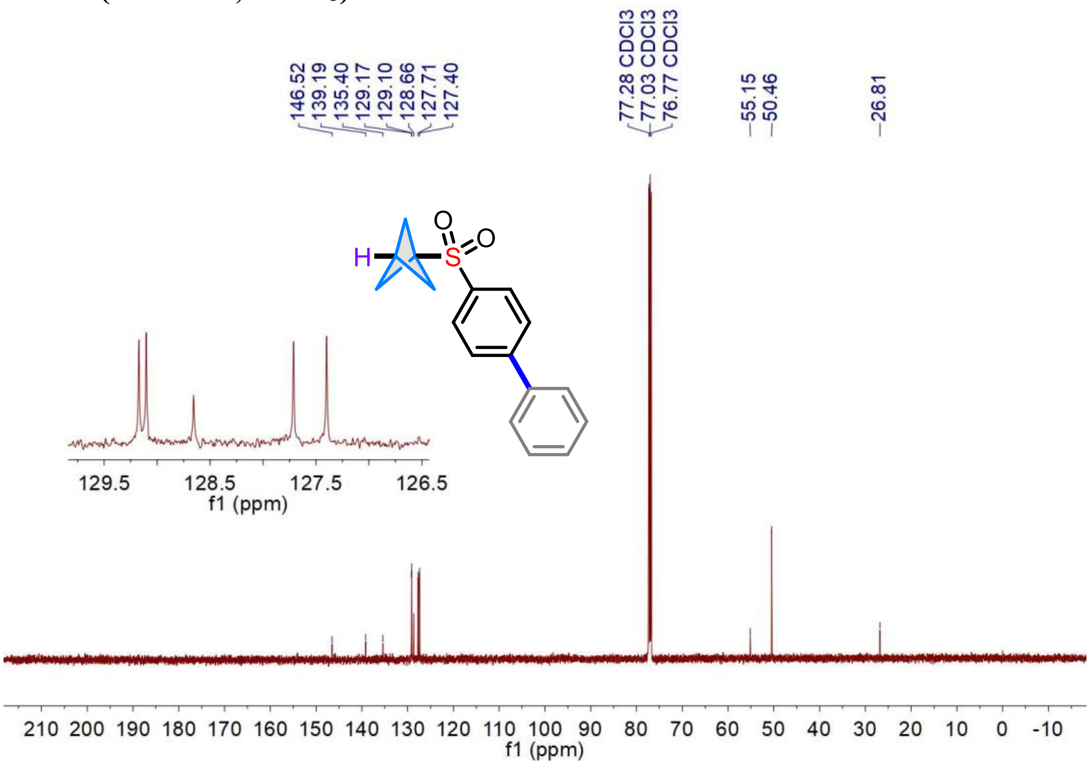

95 ^{1}H NMR (500 MHz, $CDCl_3$)

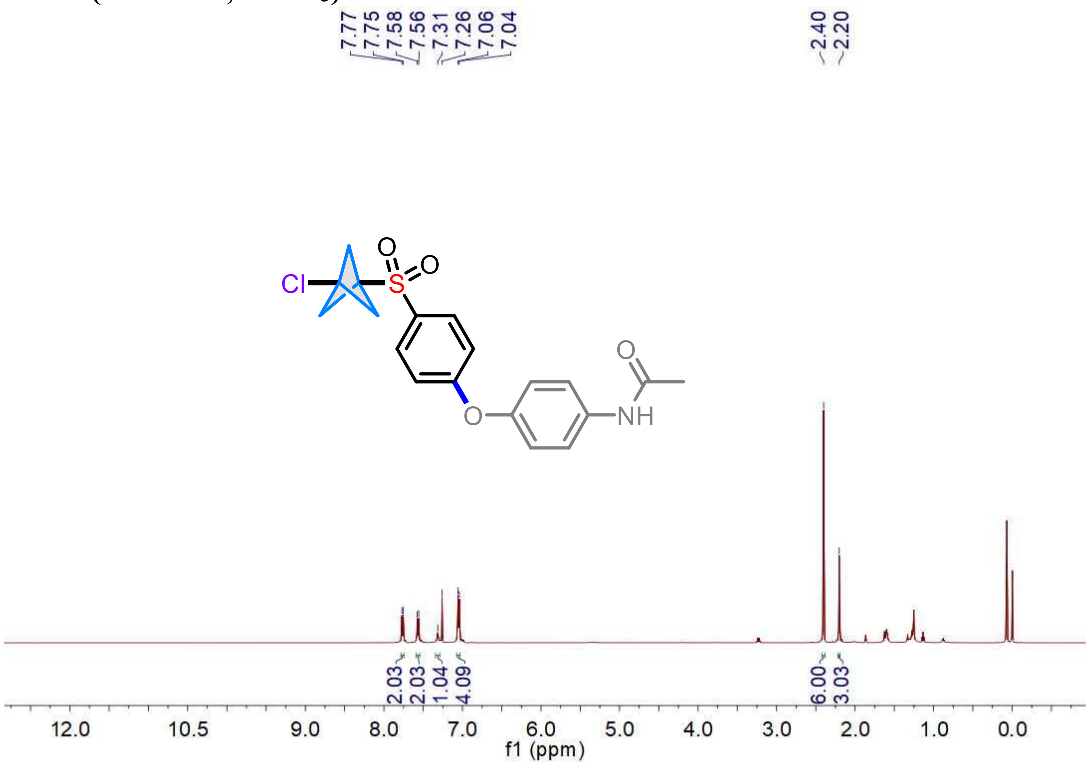

95 ^{13}C NMR (126 MHz, $CDCl_3$)

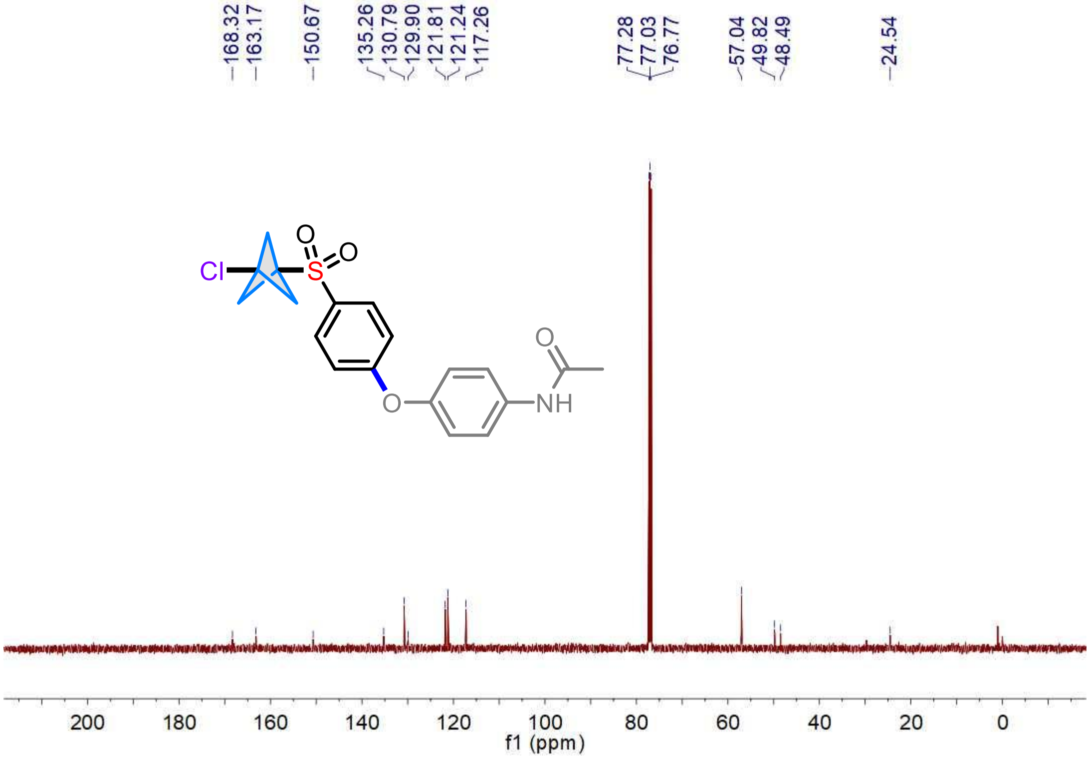

96 ^{1}H NMR (500 MHz, $CDCl_3$)

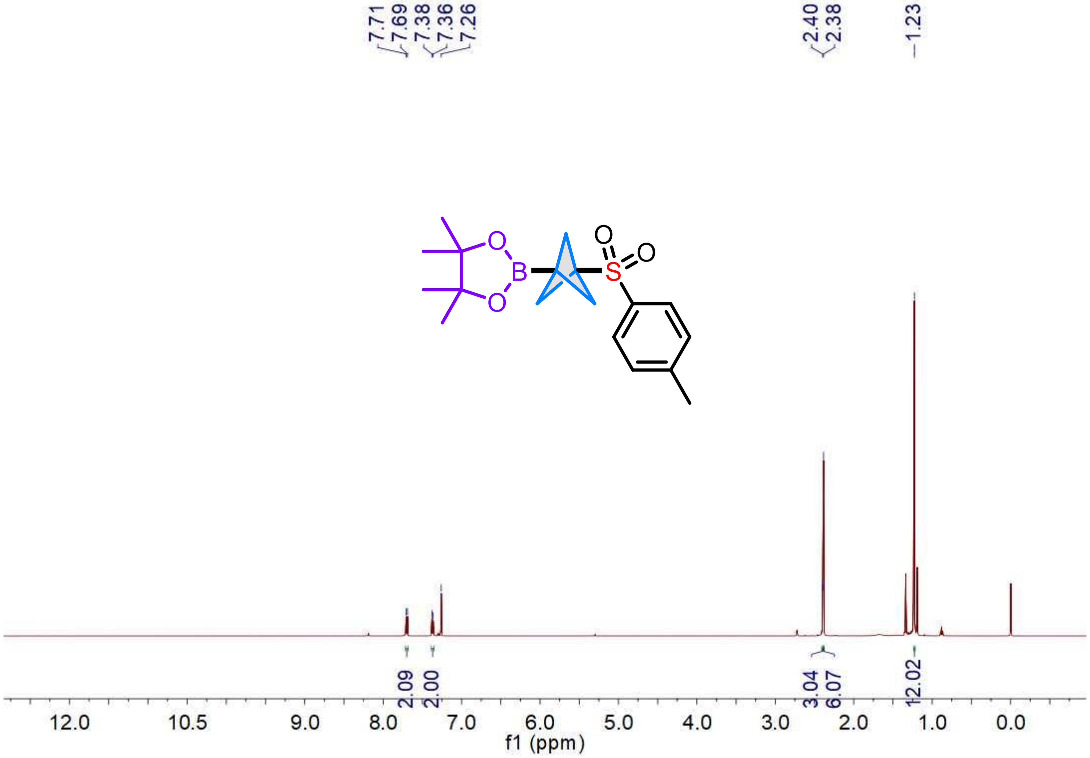

96 ^{13}C NMR (126 MHz, $CDCl_3$)

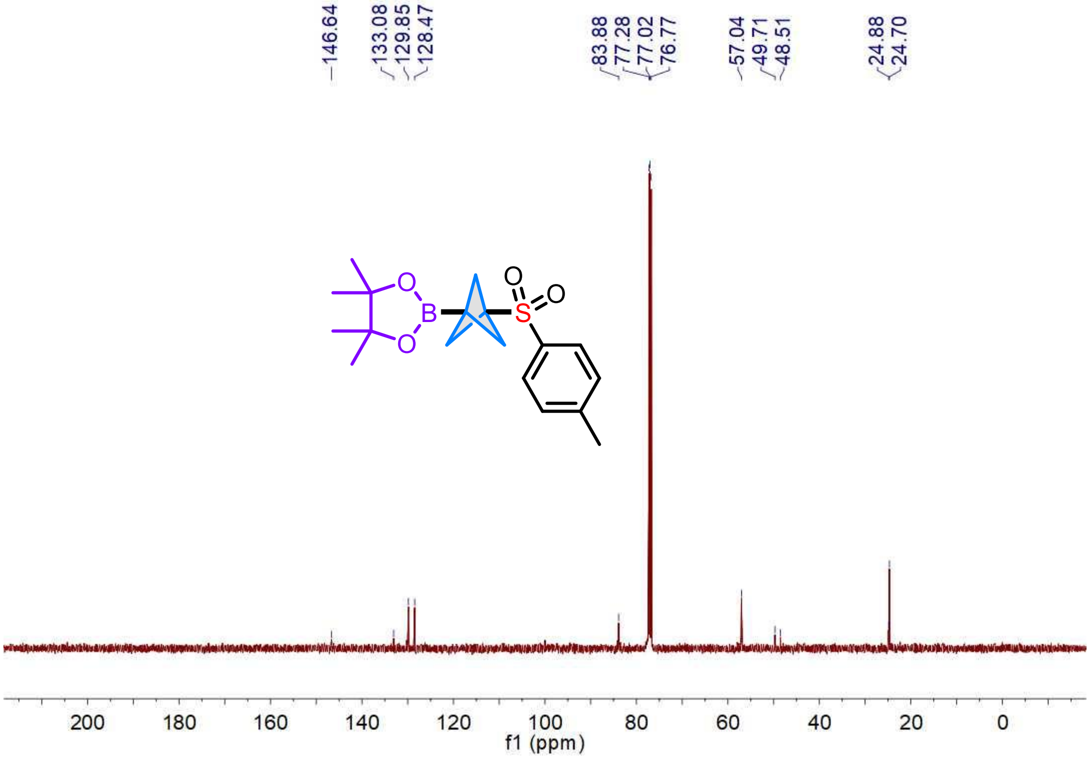

96 ^{11}B NMR (128 MHz, $CDCl_3$)

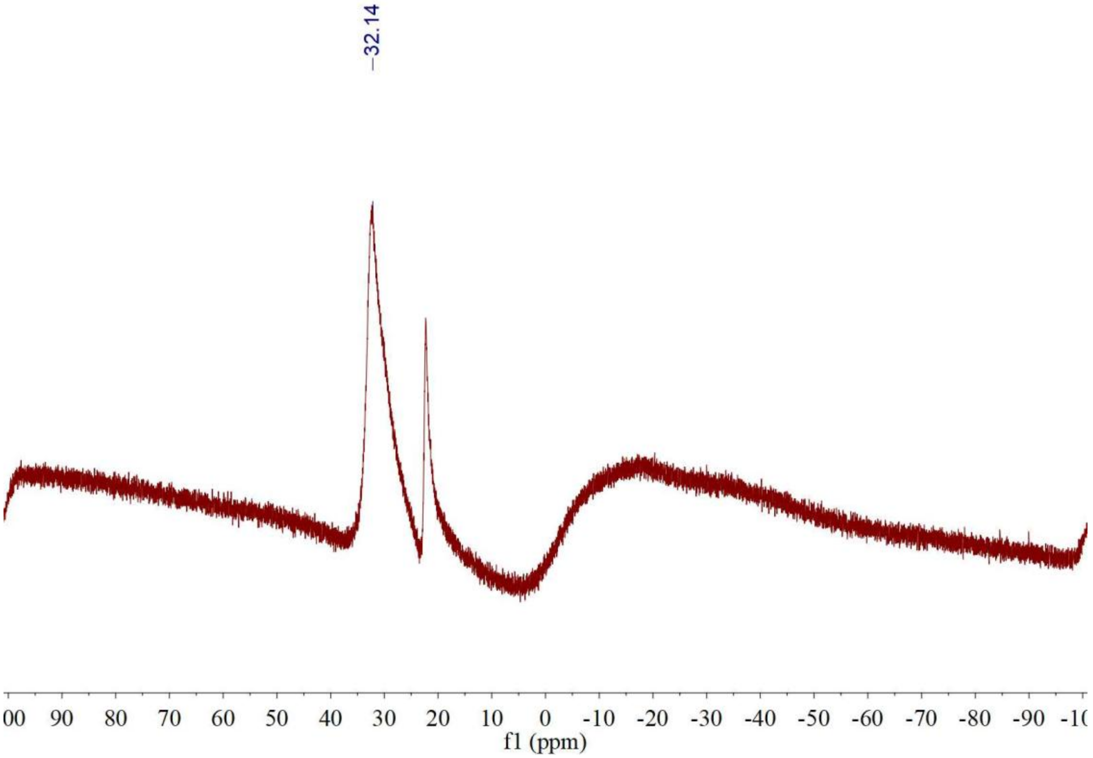

97 ^{1}H NMR (500 MHz, $CDCl_3$)

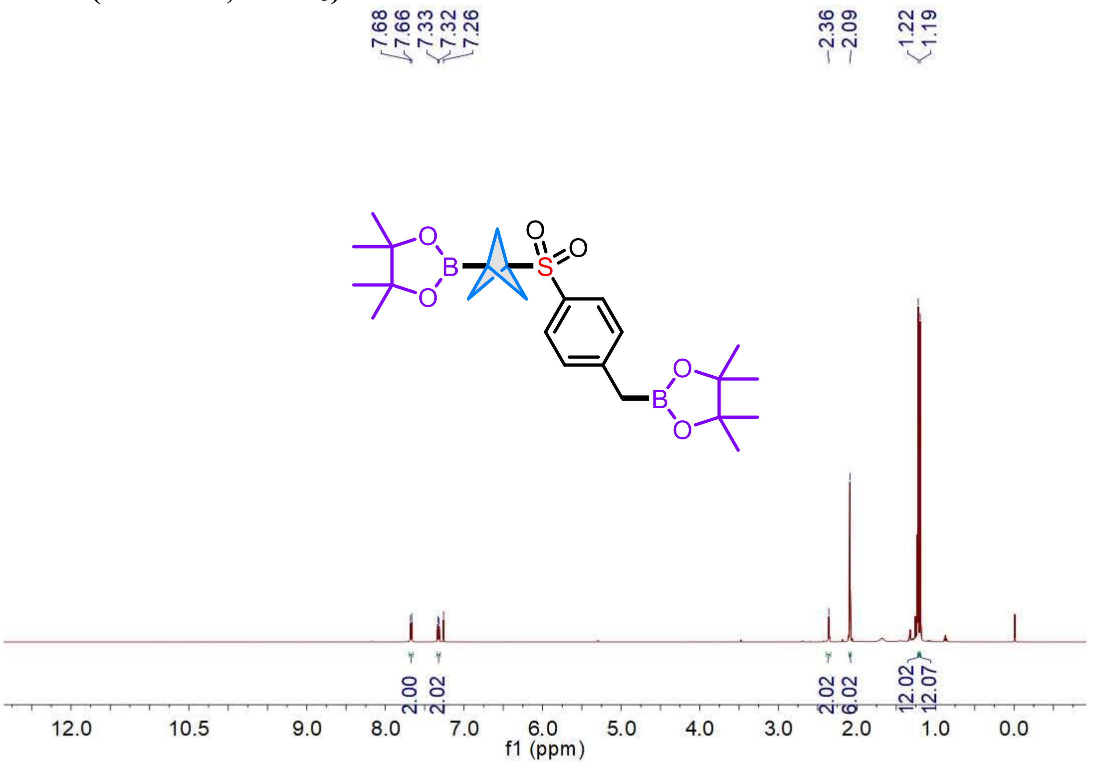

97 ^{13}C NMR (126 MHz, CDCl$_3$)

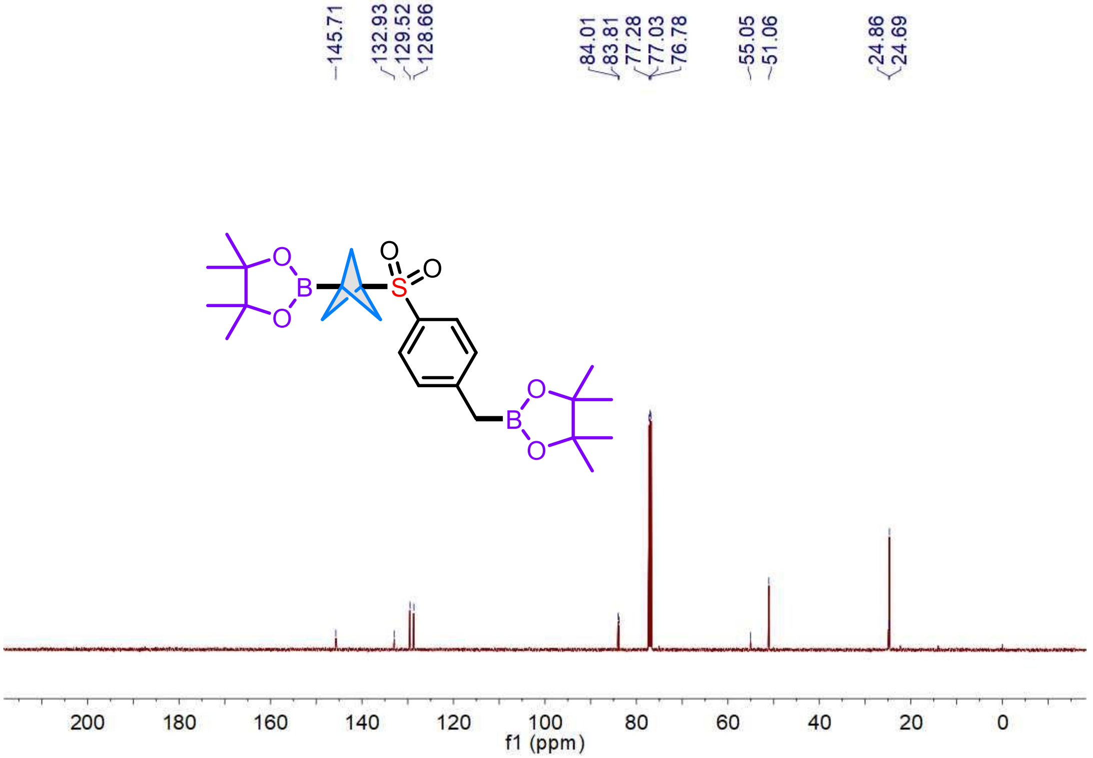

97 ^{11}B NMR (128 MHz, CDCl$_3$)

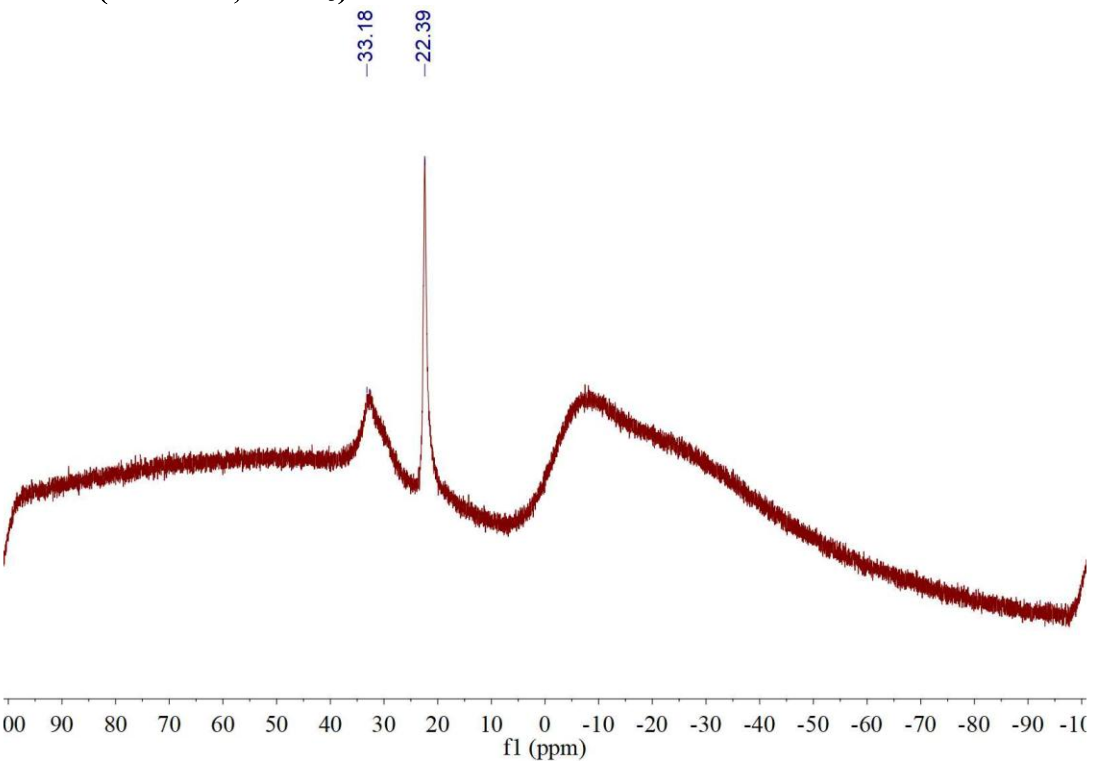

98 ^{1}H NMR (500 MHz, $CDCl_3$)

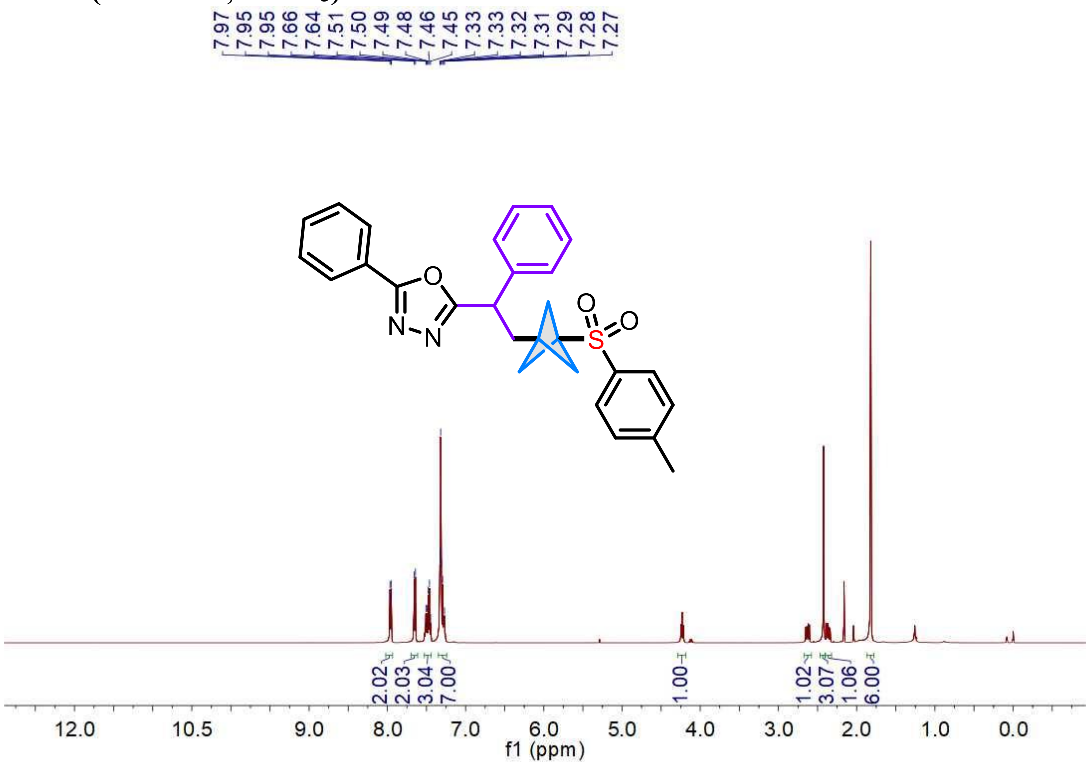

98 ^{13}C NMR (126 MHz, $CDCl_3$)

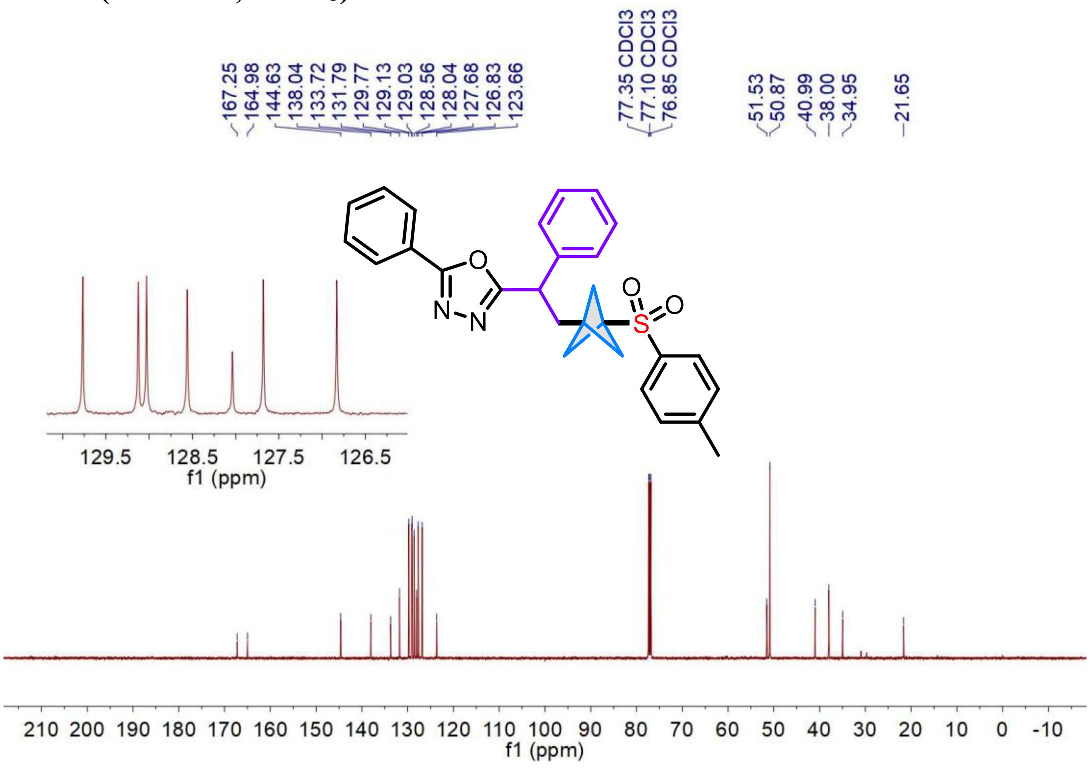

99 ^{1}H NMR (400 MHz, CDCl$_3$)

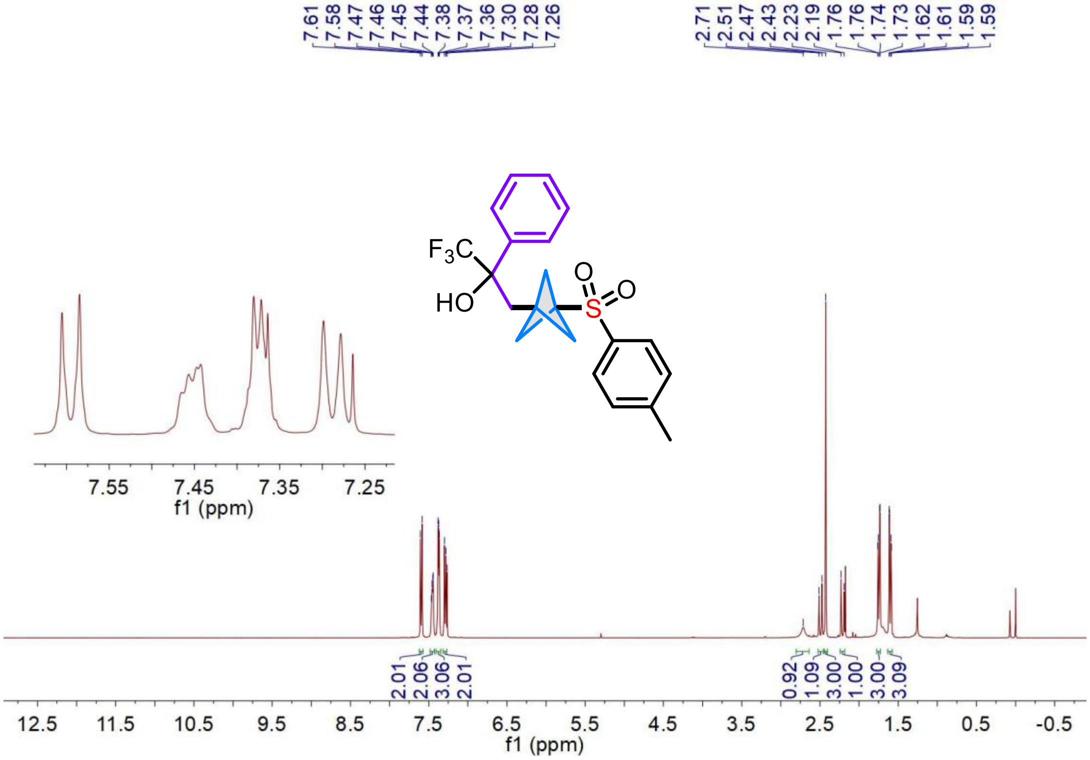

99 ^{13}C NMR (101 MHz, CDCl$_3$)

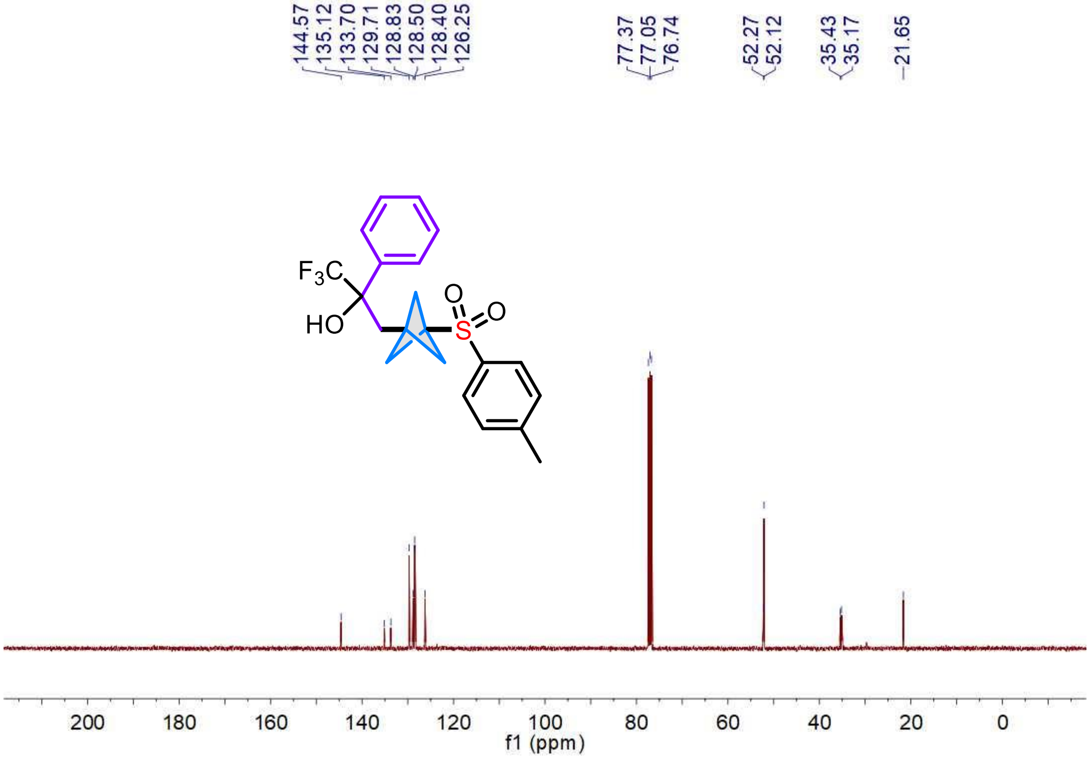

99 ^{19}F NMR (377 MHz, $CDCl_3$)

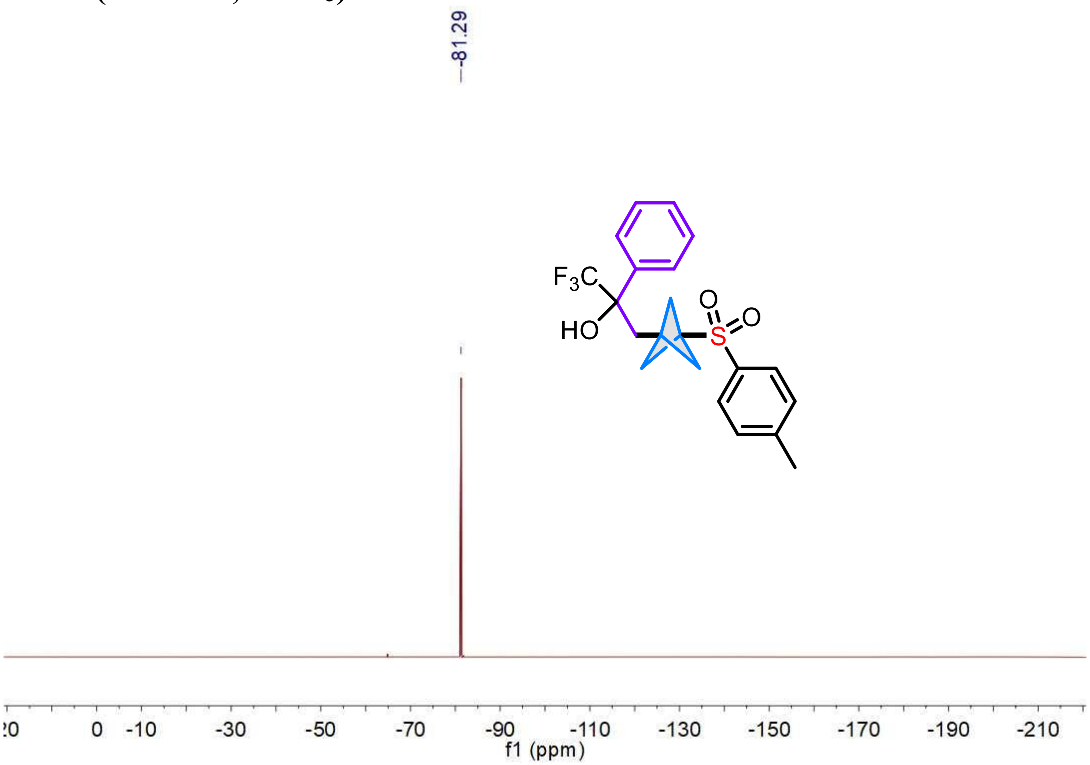

100 ^{1}H NMR (400 MHz, $CDCl_3$)

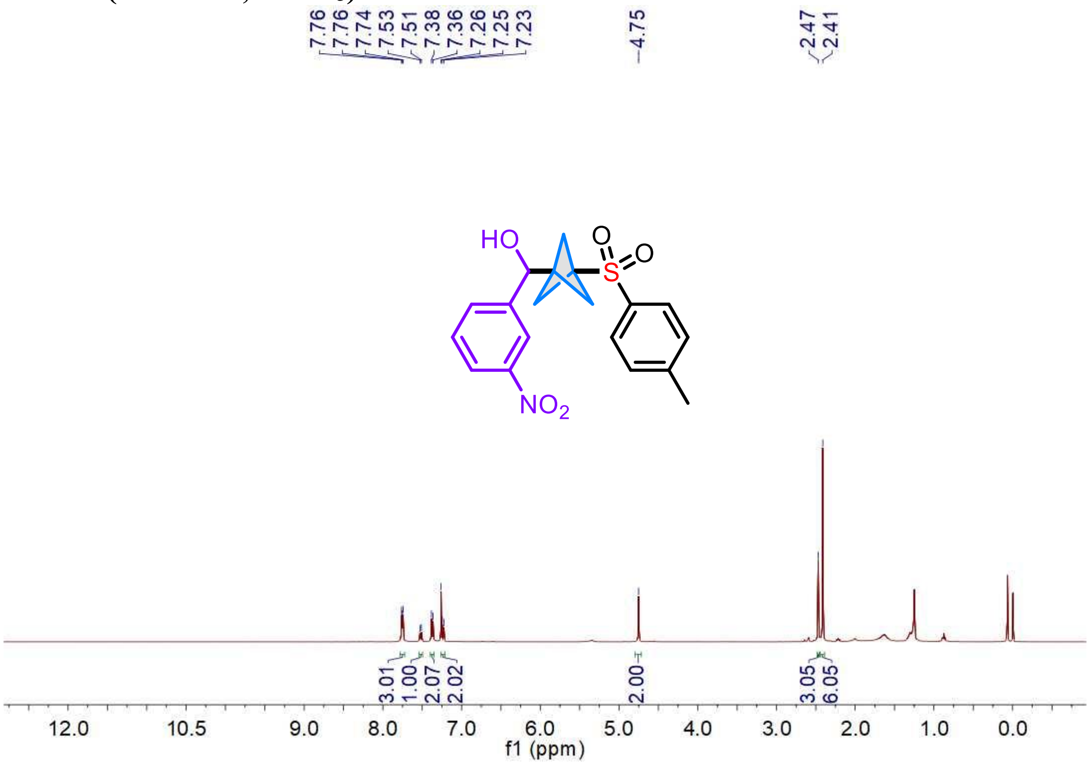

100 ^{13}C NMR (101 MHz, CDCl$_3$)

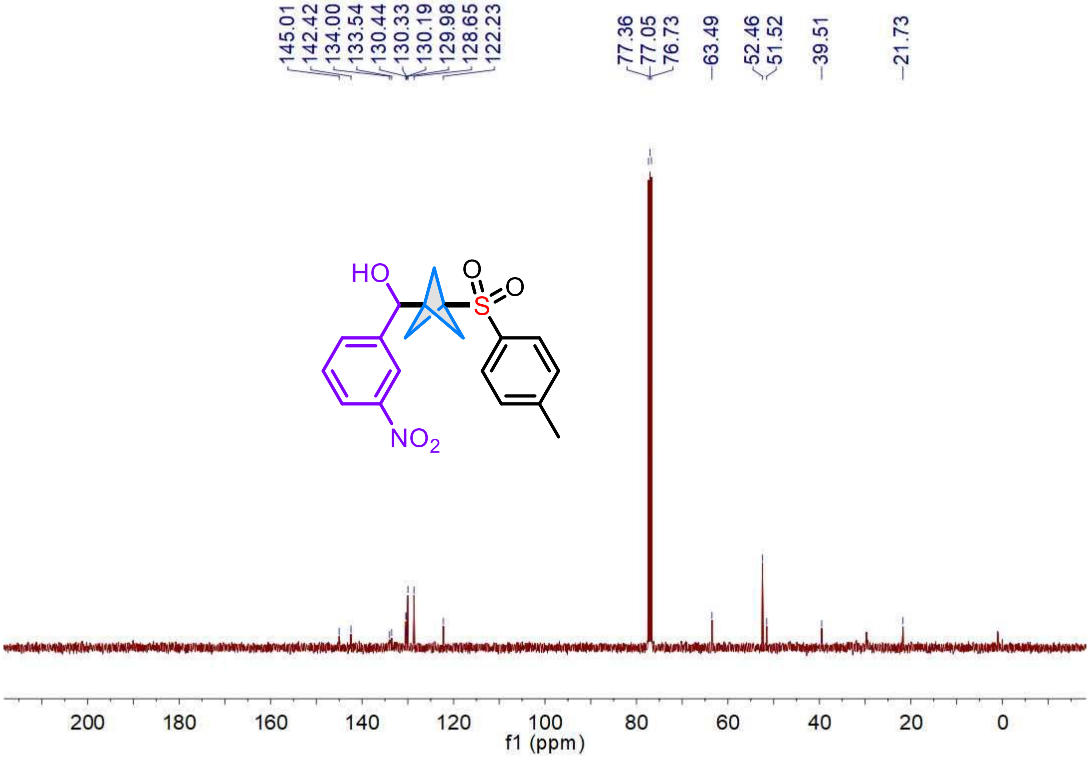

101 ^{1}H NMR (400 MHz, CDCl$_3$)

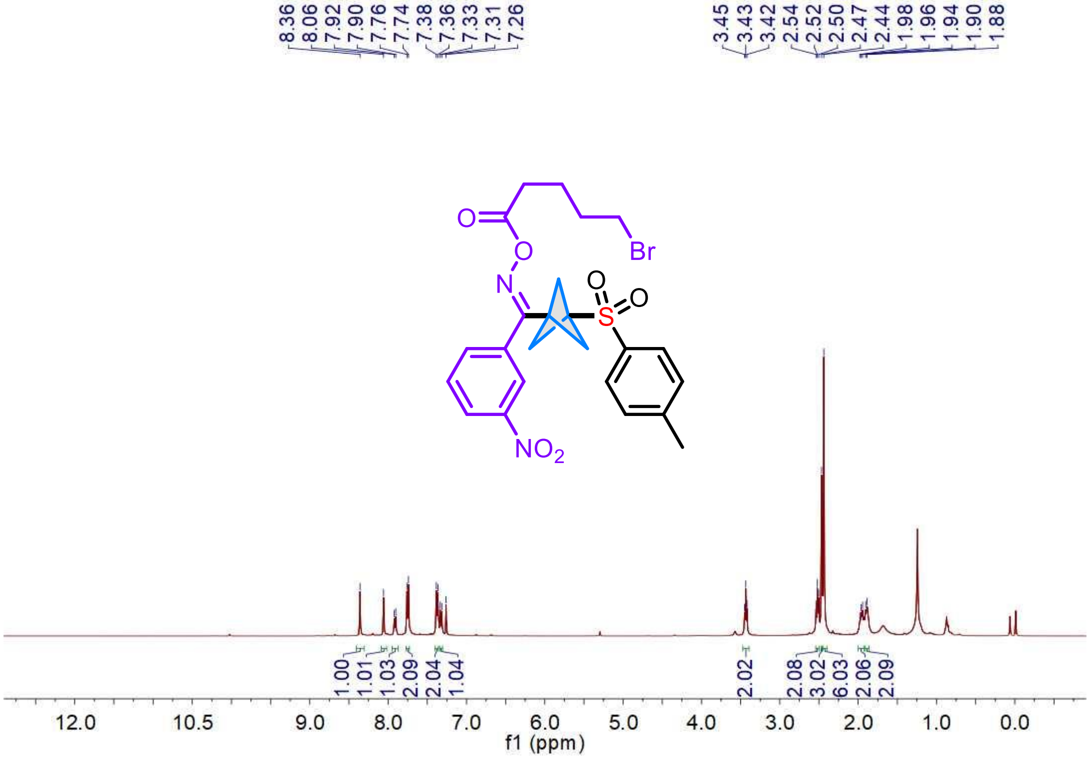

101 ^{13}C NMR (126 MHz, $CDCl_3$)

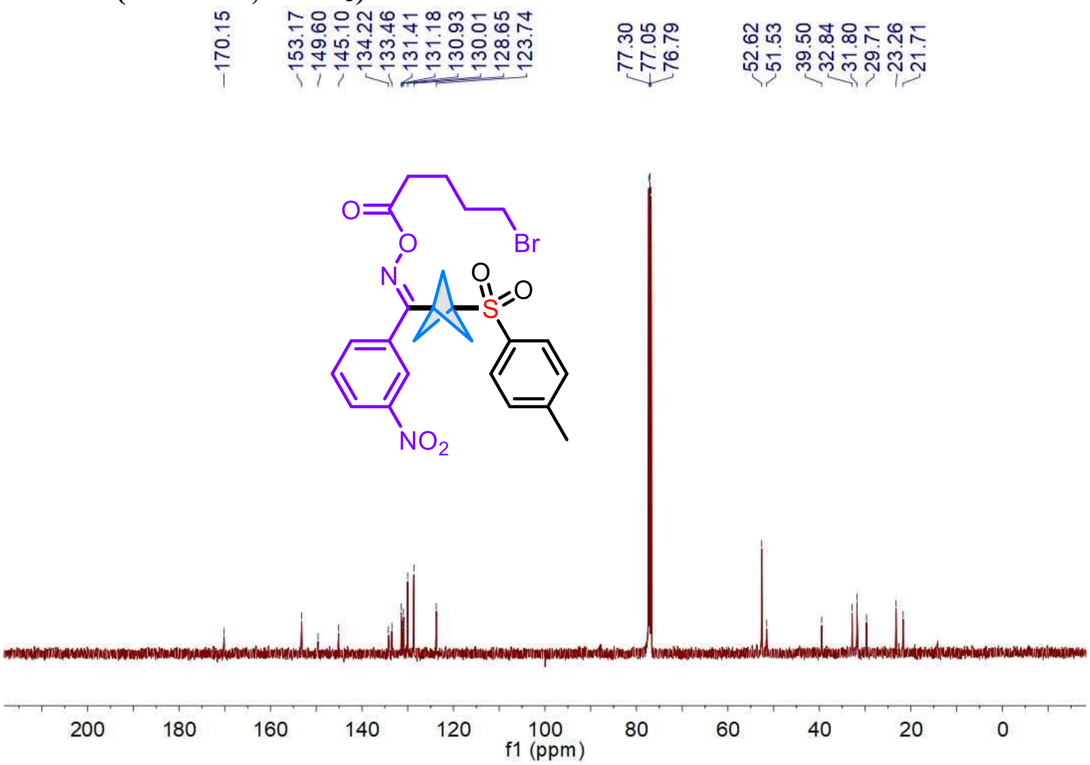

102 ^{1}H NMR (500 MHz, $CDCl_3$)

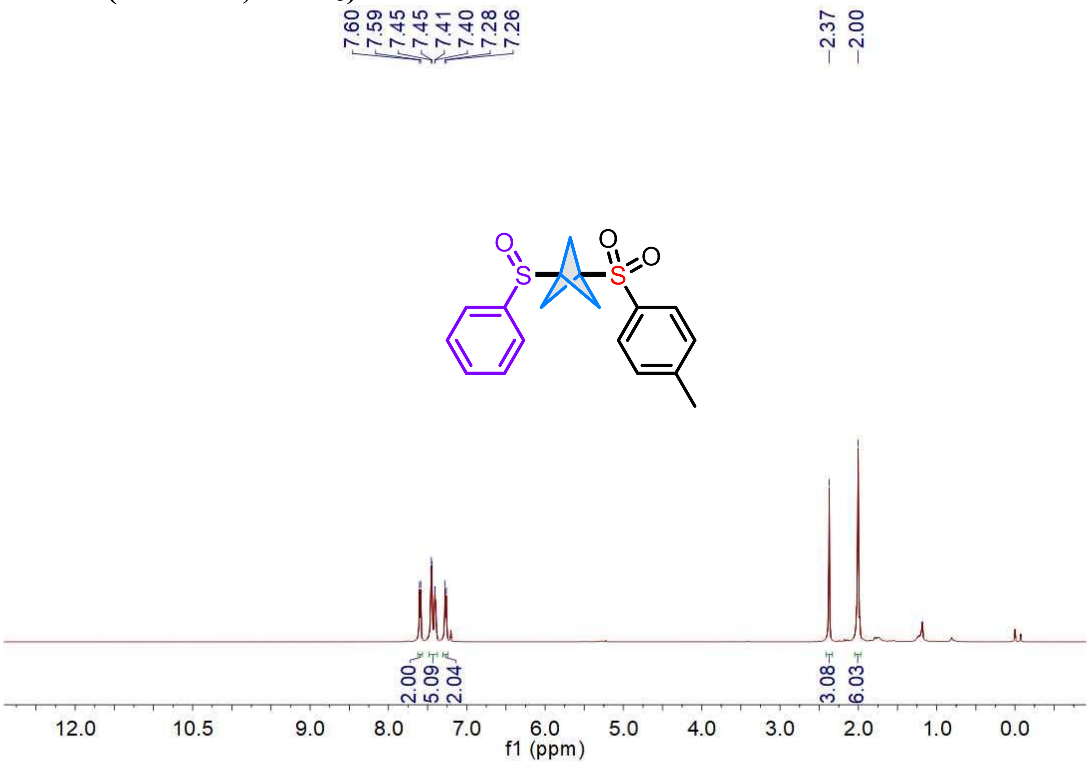

102 ^{13}C NMR (126 MHz, $CDCl_3$)

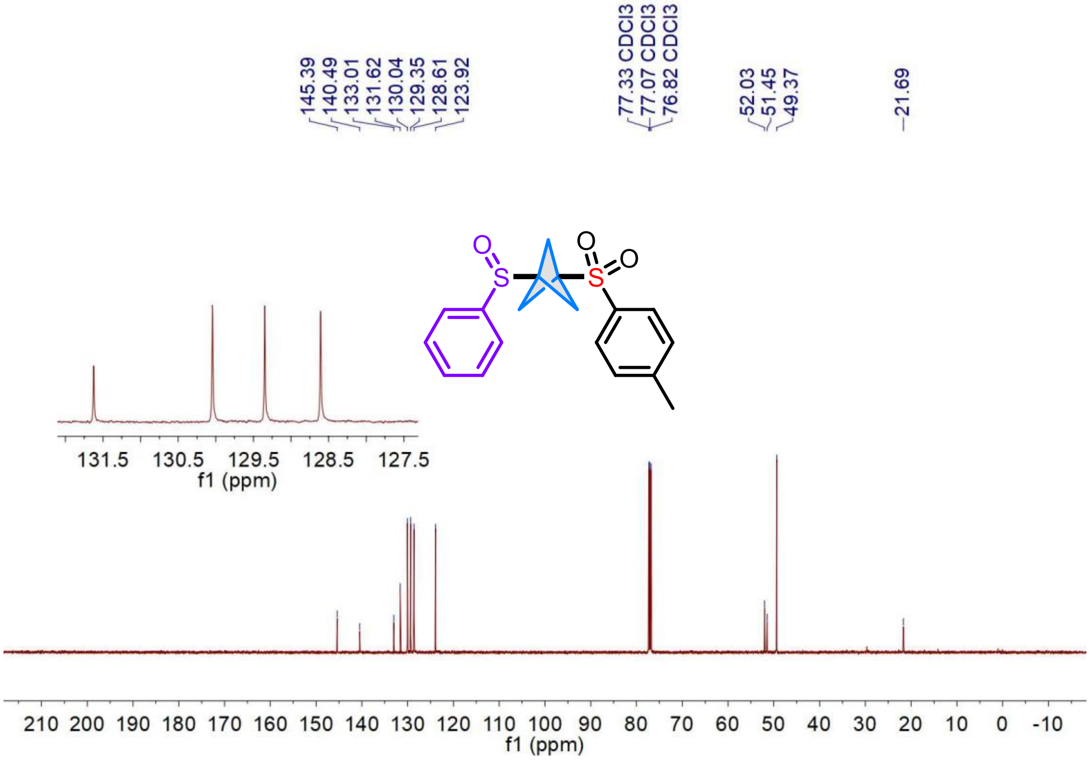

103 ^{1}H NMR (500 MHz, $CDCl_3$)

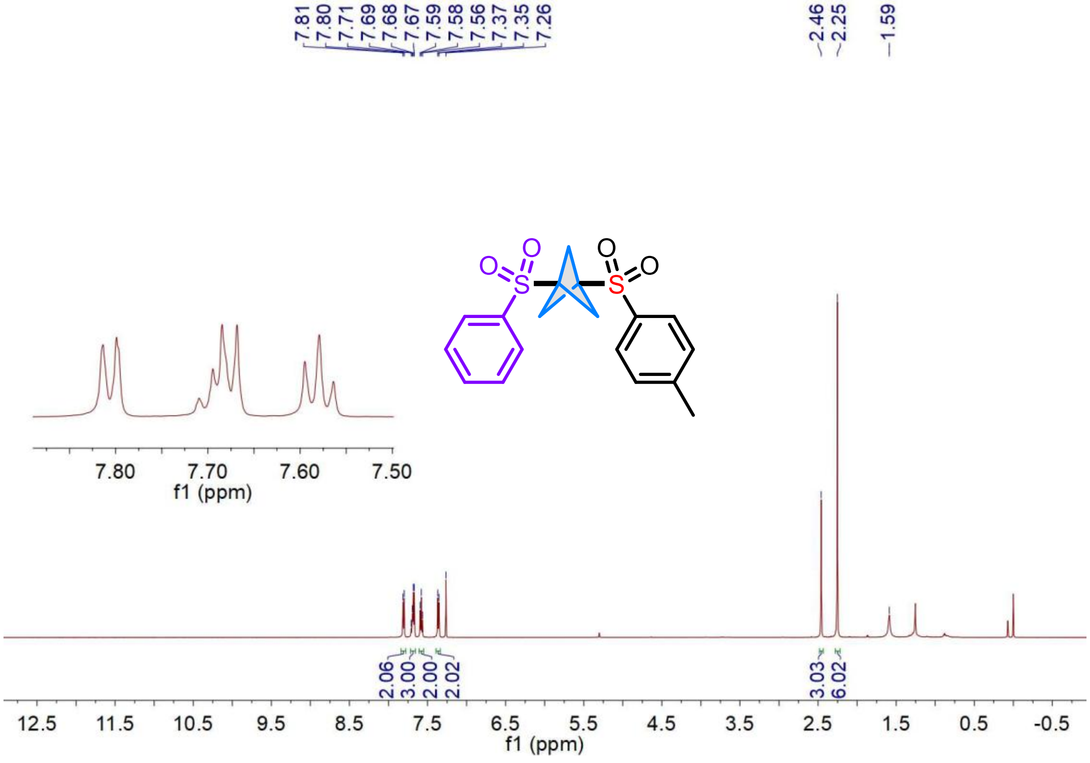

103 ^{13}C NMR (126 MHz, CDCl$_3$)

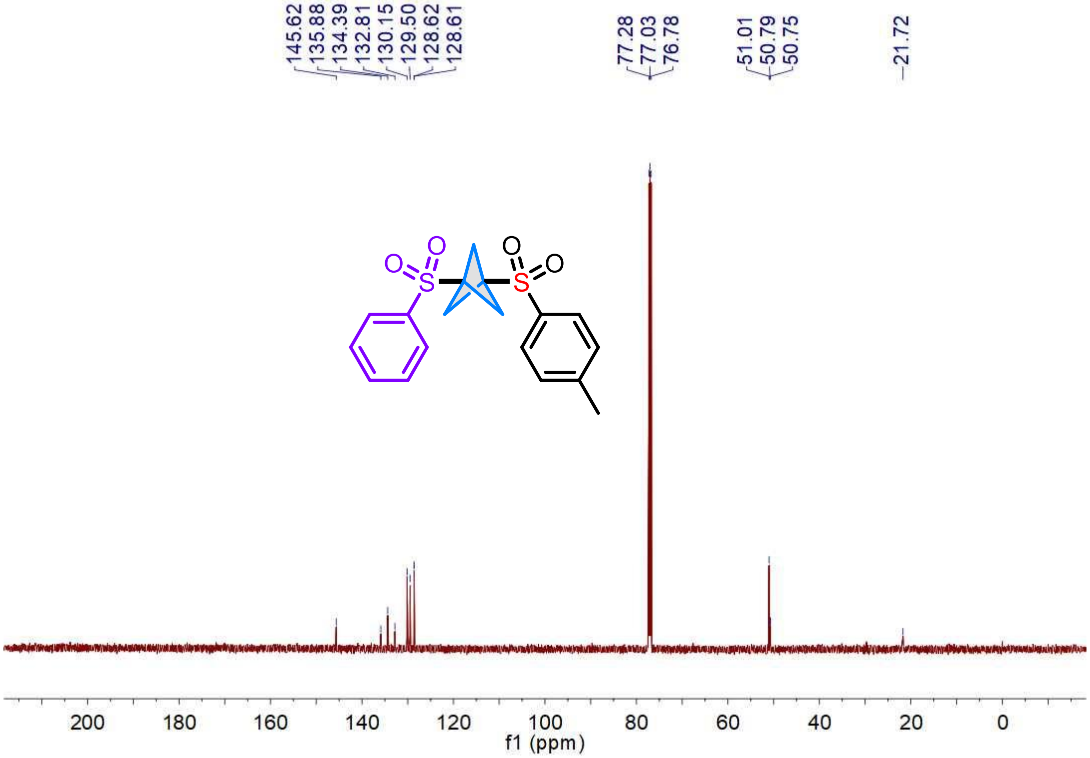

104 ^{1}H NMR (400 MHz, CDCl$_3$)

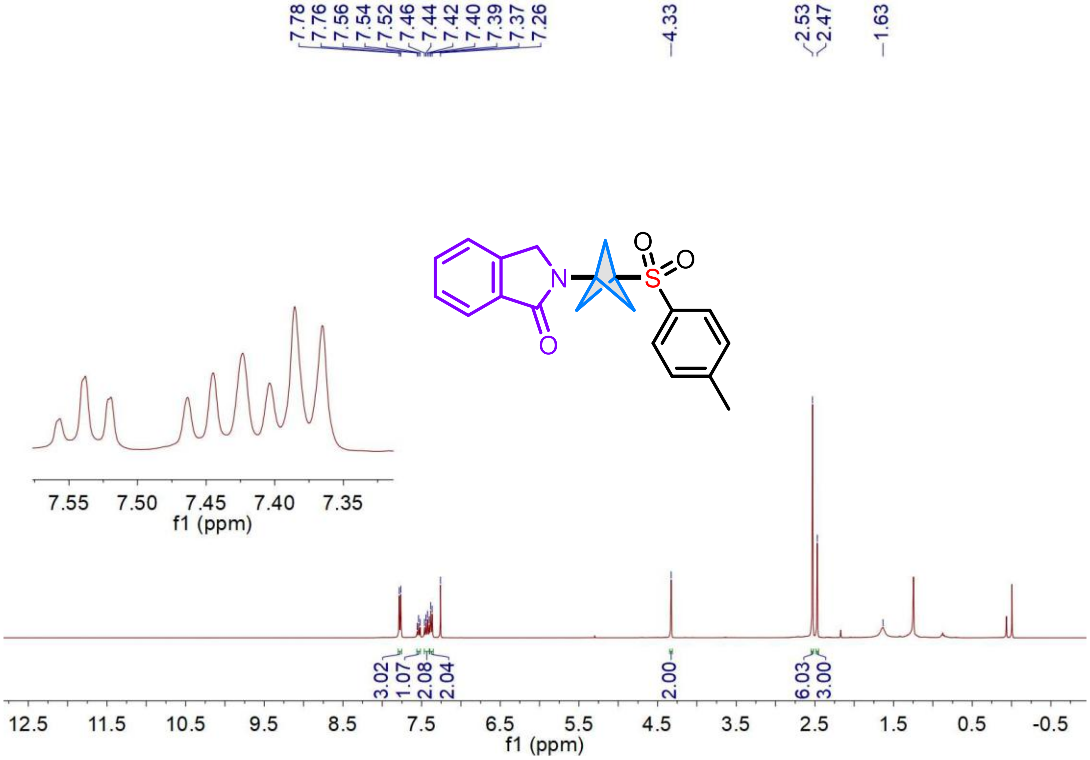

104 ^{13}C NMR (101 MHz, CDCl$_3$)

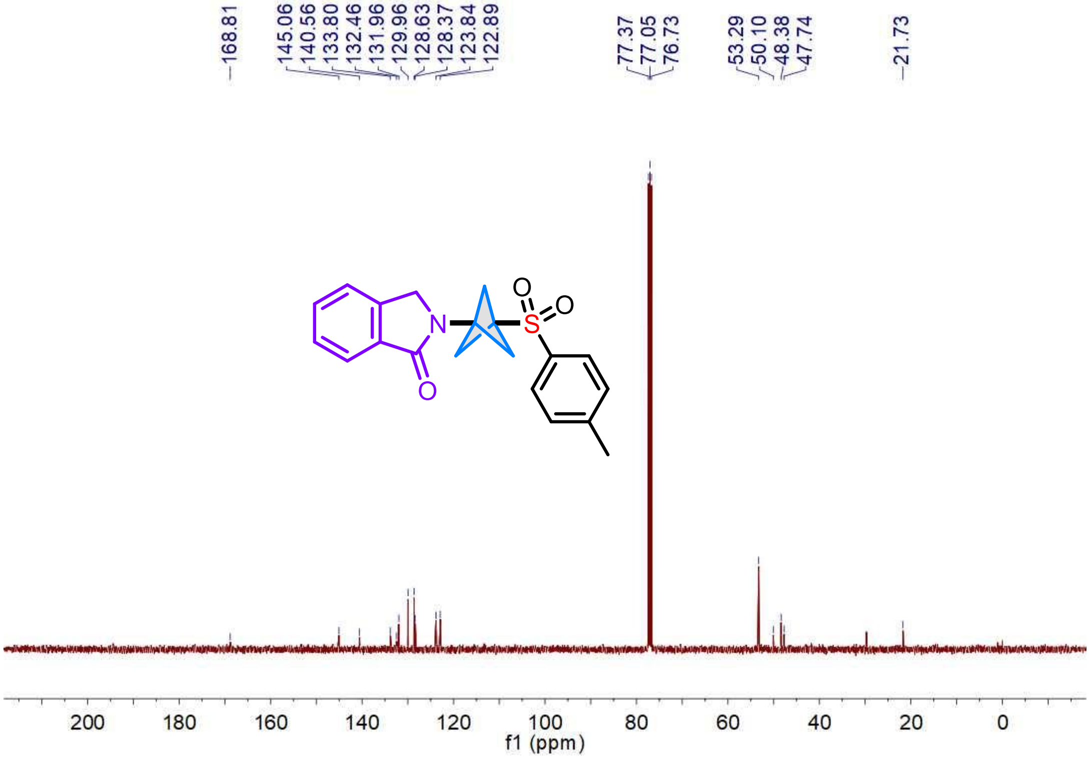

105 ^{1}H NMR (400 MHz, CDCl$_3$)

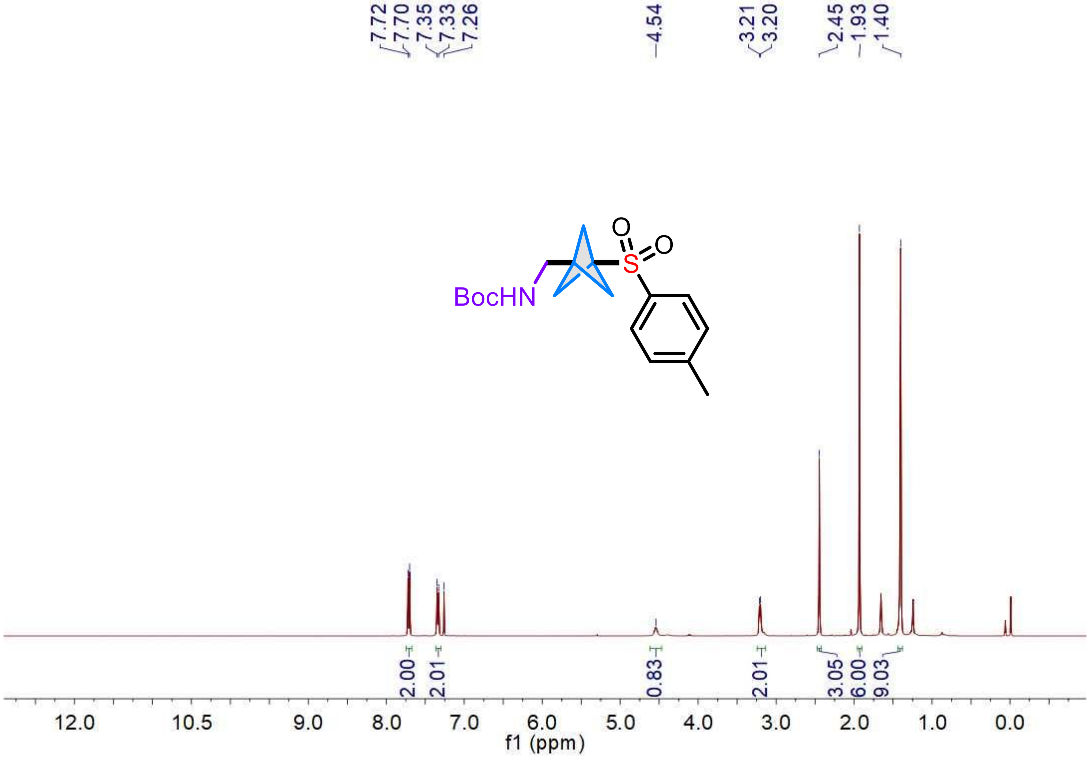

105 ^{13}C NMR (101 MHz, $CDCl_3$)

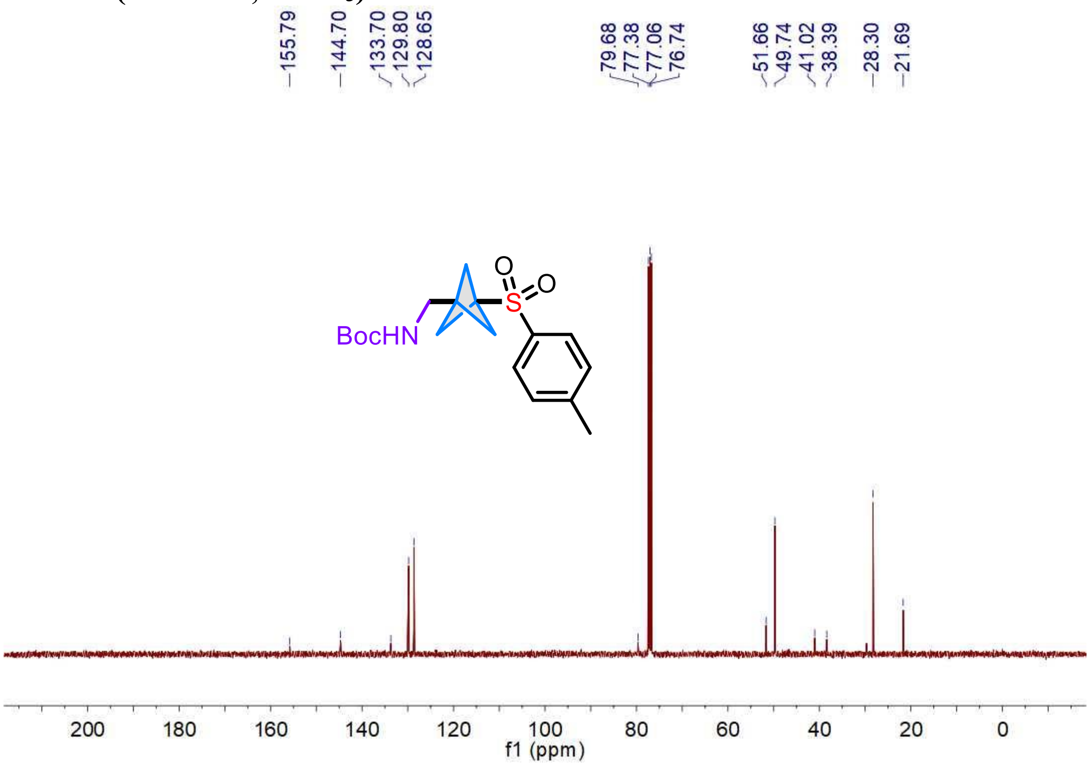

106 ^{1}H NMR (400 MHz, $CDCl_3$)

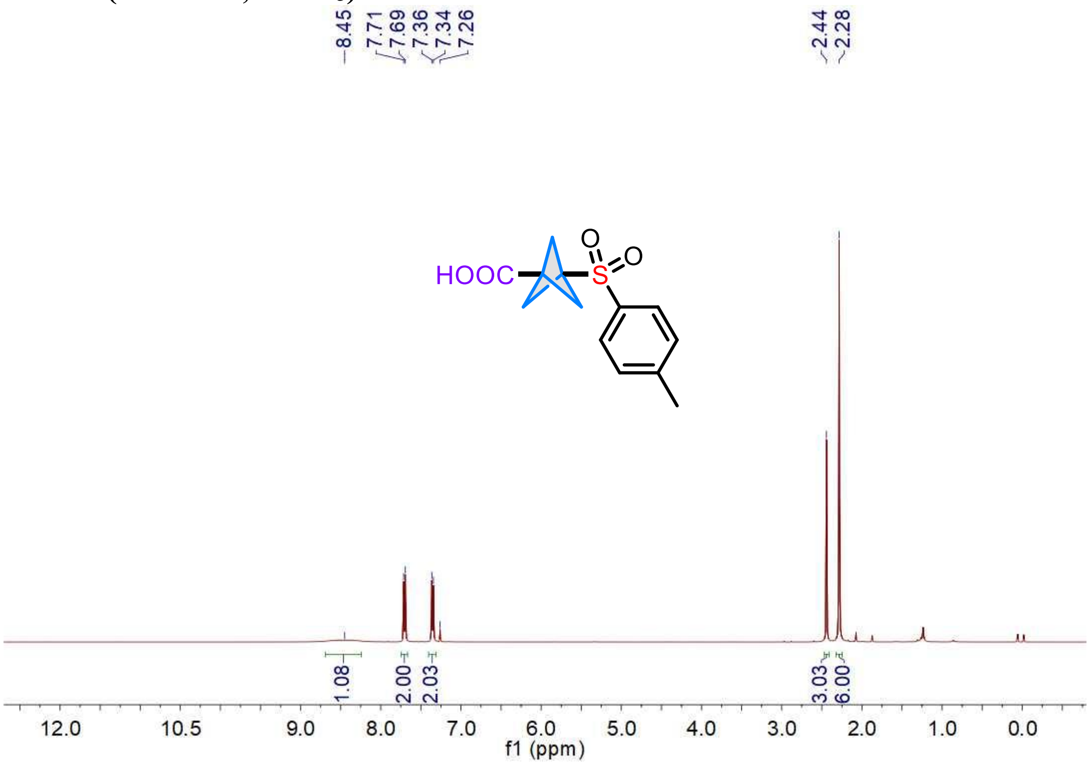

106 ^{13}C NMR (101 MHz, CDCl$_3$)

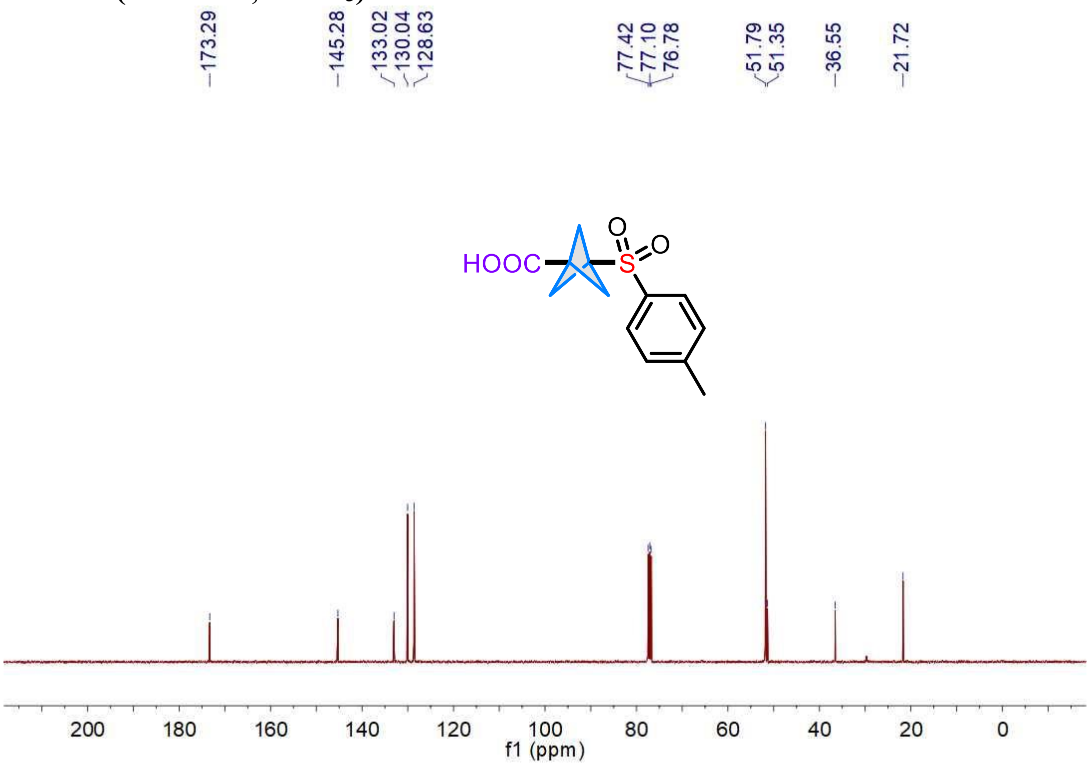

107 ^{1}H NMR (500 MHz, CDCl$_3$)

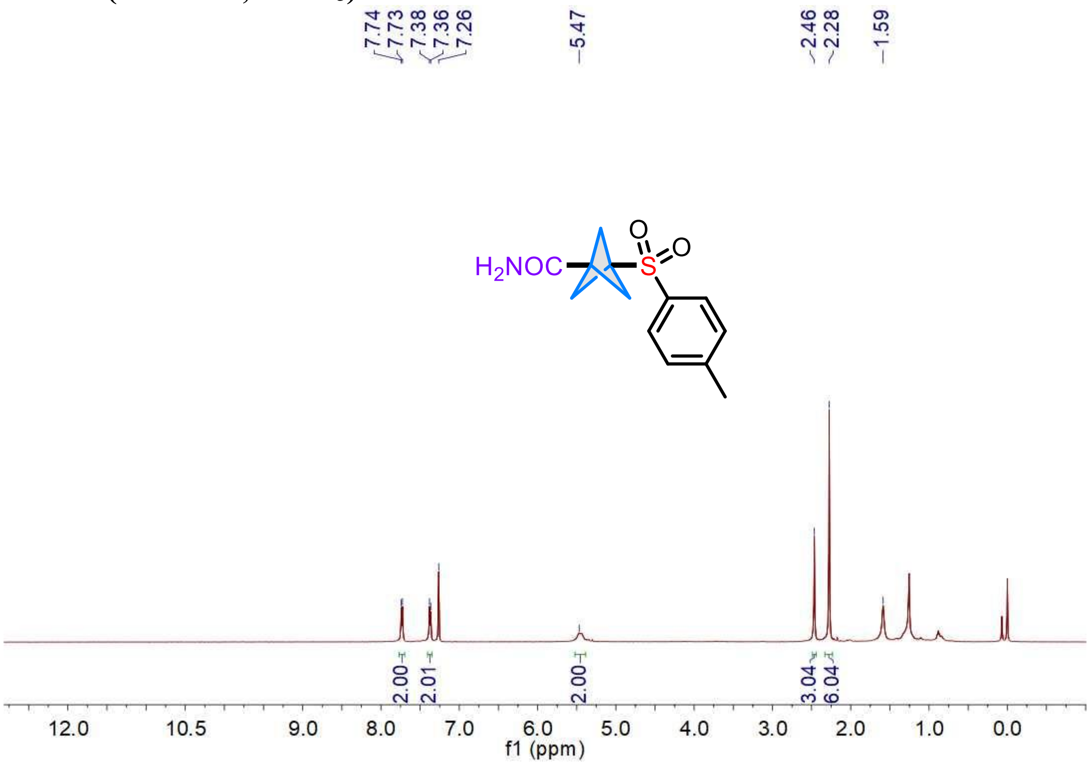

107 ^{13}C NMR (151 MHz, CDCl$_3$)

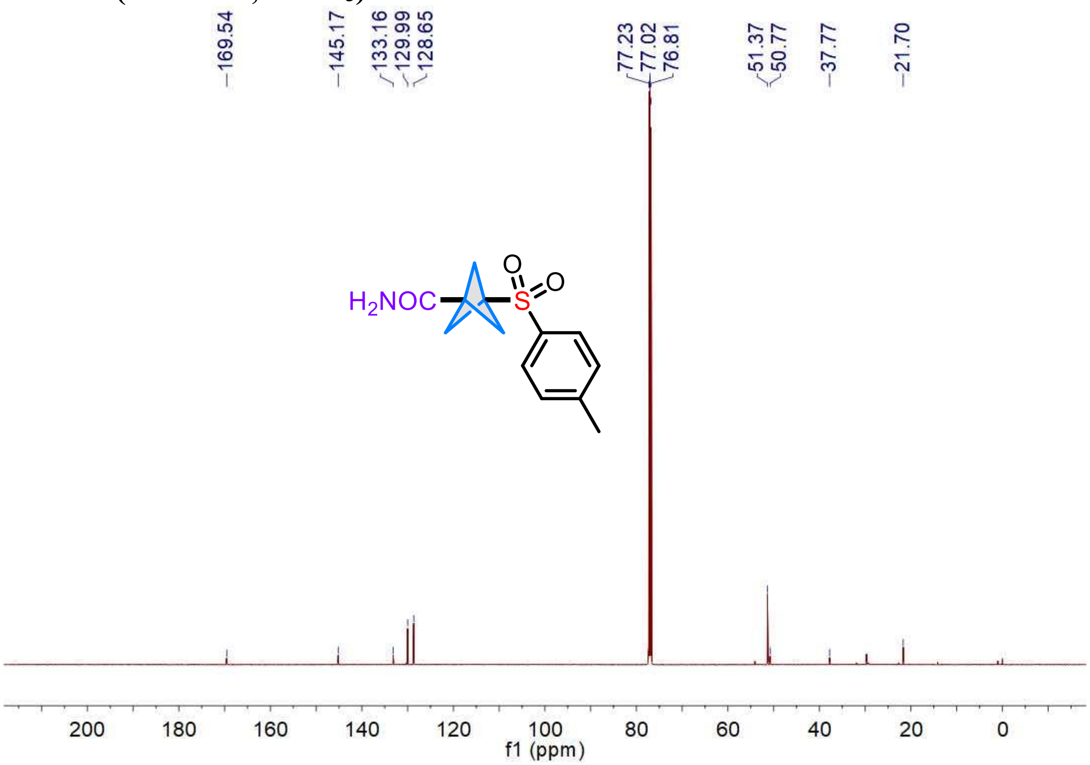

108 ^{1}H NMR (400 MHz, CDCl$_3$)

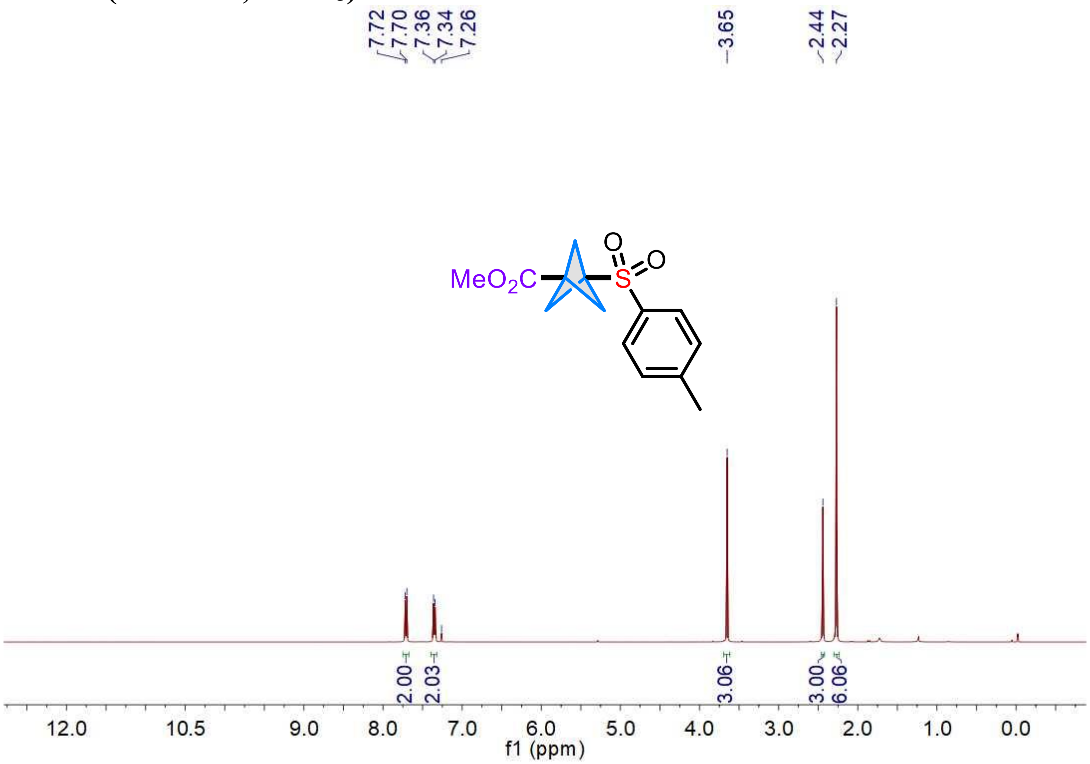

108 ^{13}C NMR (101 MHz, CDCl$_3$)

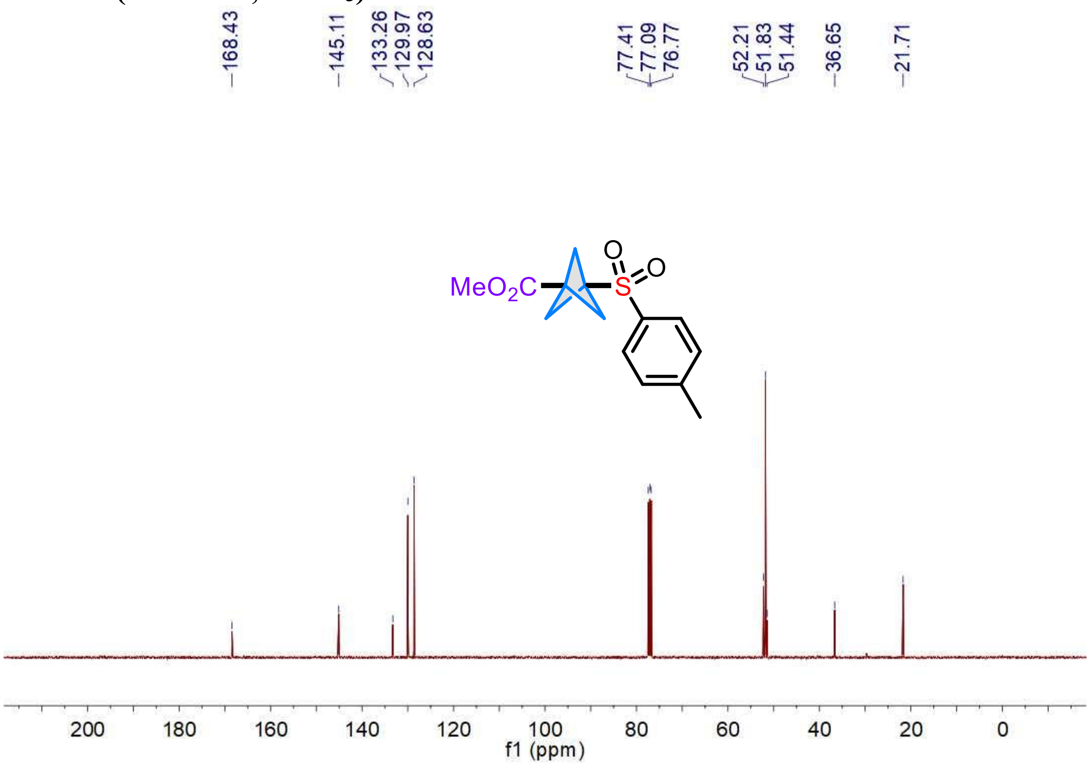

L4 ^{1}H NMR (500 MHz, CDCl$_3$)

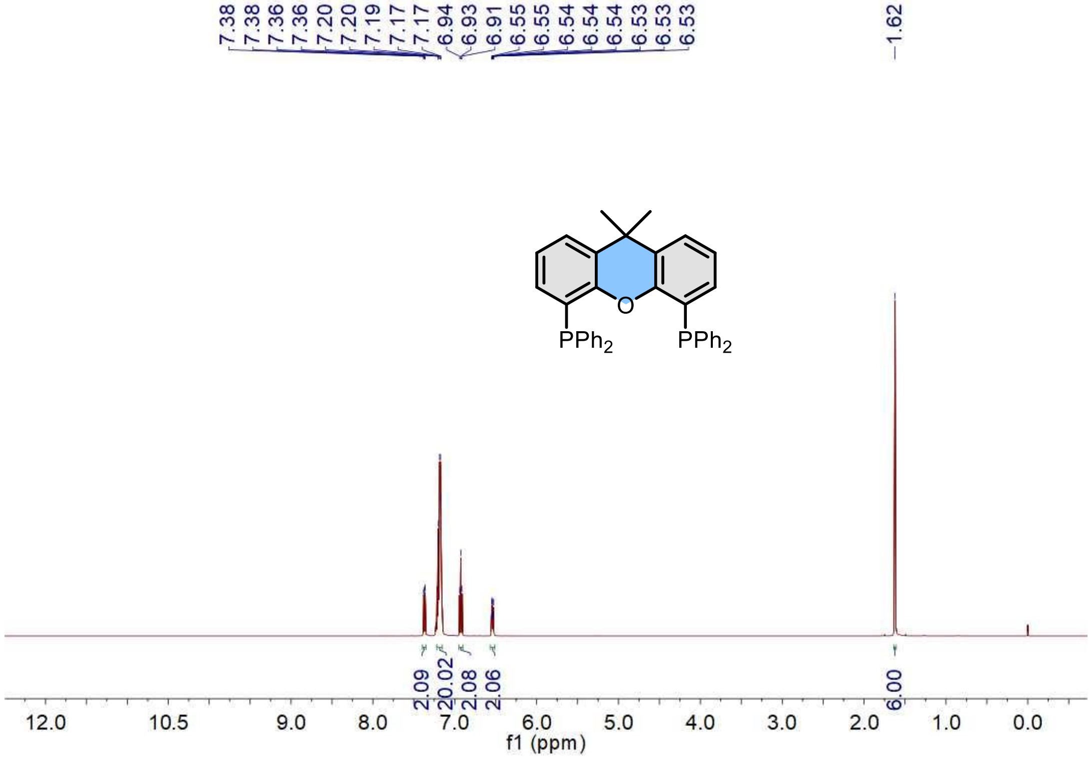

L4 ^{13}C NMR (126 MHz, $CDCl_3$)

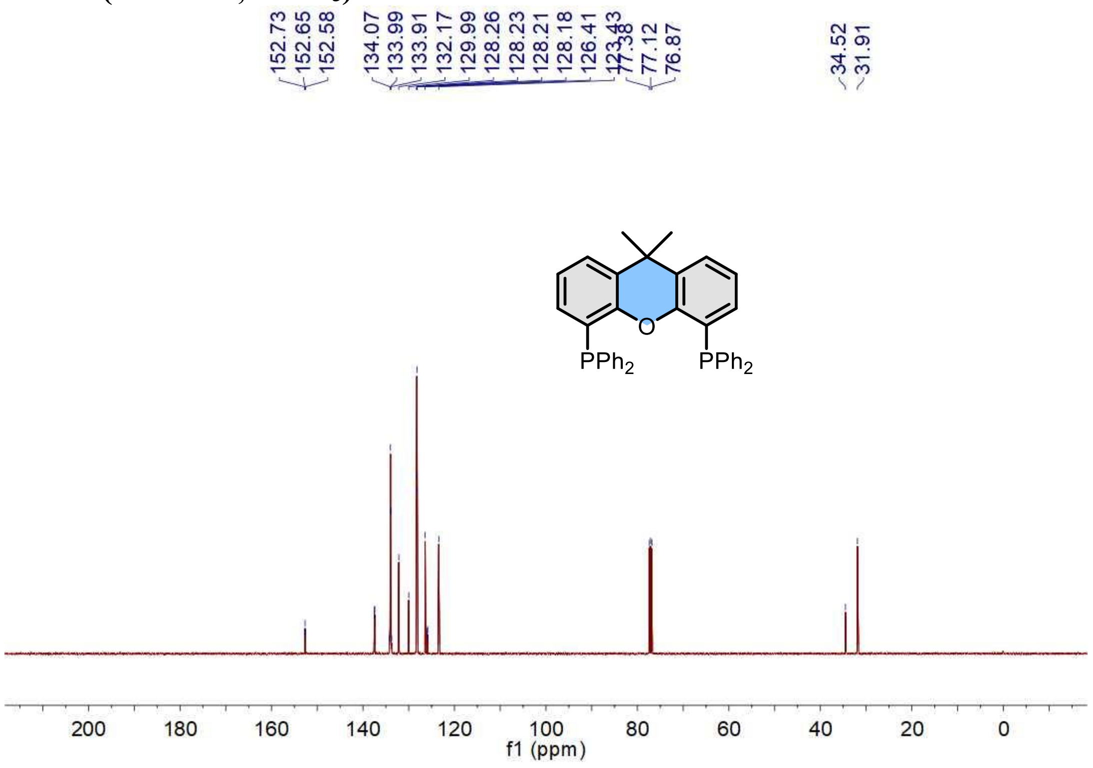

L4 ^{31}P NMR (202 MHz, $CDCl_3$)

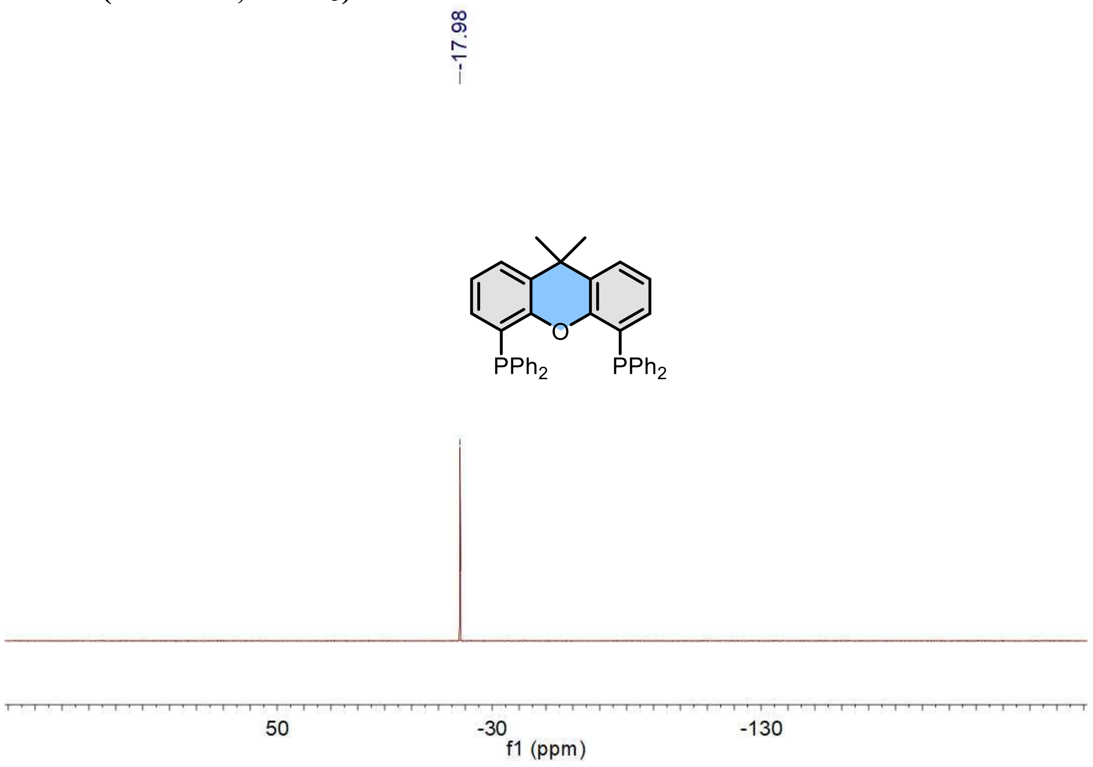

REFERENCES

1. M. A. M. Subbaiah, N. A. Meanwell, Bioisosteres of the phenyl ring: Recent strategic applications in lead optimization and drug design. *J. Med. Chem.* **64**, 14046–14128 (2021).

2. J. Tsien, C. Hu, R. R. Merchant, T. Qin, Three-dimensional saturated C(sp^3)-rich bioisosteres for benzene. *Nat. Rev. Chem.* **8**, 605–627 (2024).

3. Y. Zhang, K.-D. Li, S. Yu, K. Pan, H. Xu, H.-M. Huang, Divergent synthesis of 1-azabicyclo[n.1.1]alkane bioisosteres via photoinduced palladium catalysis. *J. Am. Chem. Soc.* **147**, 33700–33710 (2025).

4. M. Zhang, M. Chapman, B. R. Sarode, B. Xiong, H. Liang, J. K. Chen, E. Weerapana, J. P. Morken, Catalytic asymmetric synthesis of meta benzene isosteres. *Nature* **633**, 90–95 (2024).

5. W. Su, J. Zhu, Y. Chen, X. Zhang, W. Qiu, K. Yang, P. Yu, Q. Song, Copper-catalysed asymmetric hydroboration of alkenes with 1,2-benzazaborines to access chiral naphthalene isosteres. *Nat. Chem.* **16**, 1312–1319 (2024).

6. Y. Hao, J. Chen, Z. Jiao, C.-Y. Su, Visible-light-induced [2π+2σ] cycloaddition enabled by cage-confined photocatalysis for precise bicyclo[2.1.1]hexane construction. *Angew. Chem. Int. Ed.* **64**, e202517394 (2025).

7. C.-Y. Tan, H. Ju, J. Jeong, J. Kim, S. Hong, Photocatalytic radical umpolung for strain-release difunctionalization of (aza)bicyclo[1.1.0]butanes. *Angew. Chem. Int. Ed.* **64**, e202517231 (2025).

8. S. Cuadros, J. Paut, E. Anselmi, G. Dagousset, E. Magnier, L. Dell'Amico, Light-driven synthesis and functionalization of bicycloalkanes, cubanes and related bioisosteres. *Angew. Chem. Int. Ed.* **63**, e202317333 (2024).

9. P. Bellotti, F. Glorius, Strain-release photocatalysis. *J. Am. Chem. Soc.* **145**, 20716–20732 (2023).

10. X. Zhang, R. T. Smith, C. Le, S. J. McCarve, B. T. Shireman, N. I. Carruthers, D. W. C. MacMillan, Copper-mediated synthesis of drug-like bicyclopentanes. *Nature* **580**, 220–226 (2020).

11. K.-N. Yuan, H. Zhuang, J. Wei, Y. Shen, H.-Q. Yao, M.-H. Li, L.-L. Xu, M. Shang, Modular access to saturated bioisosteres of anilines via photoelectrochemical decarboxylative C(sp^3)–N coupling. *Nat. Commun.* **16**, 920 (2025).

12. I. F. Yu, J. L. Manske, A. Diéguez-Vázquez, A. Misale, A. E. Pashenko, P. K. Mykhailiuk, S. V. Ryabukhin, D. M. Volochnyuk, J. F. Hartwig, Catalytic undirected borylation of tertiary C–H bonds in bicyclo[1.1.1]pentanes and bicyclo[2.1.1]hexanes. *Nat. Chem.* **15**, 685–693 (2023).

13. J.-T. Che, H.-B. Zhang, W.-Y. Ding, S.-H. Xiang, B. Tan, Triplet carbene insertion enables modular access to C2-substituted bicyclo[1.1.1]pentanes. *J. Am. Chem. Soc.* **147**, 33879–33887 (2025).

14. K. I. Burton, D. W. C. MacMillan, Rapid access to 3-substituted bicyclo[1.1.1]pentanes. *Chem* **11**, 102537 (2025).

15. Z. Bai, Z. Wang, T. H.-F. Wong, T. Ritter, Thianthrenium-enabled modular synthesis of bicyclo[1.1.1]pentanes. *Nat. Synth.* **4**, 1161–1169 (2025).

16. M. Chen, Y. Cui, X. Chen, R. Shang, X. Zhang, C−F bond activation enables synthesis of aryl difluoromethyl bicyclopentanes as benzophenone-type bioisosteres. *Nat. Commun.* **15**, 419 (2024).

17. H. Takano, H. Katsuyama, H. Hayashi, M. Harukawa, M. Tsurui, S. Shoji, Y. Hasegawa, S. Maeda, T. Mita, Synthesis of bicyclo[1.1.1]pentane (BCP)-based straight-shaped diphosphine ligands. *Angew. Chem. Int. Ed.* **62**, e202303435 (2023).

18. W. Dong, E. Yen-Pon, L. Li, A. Bhattacharjee, A. Jolit, G. A. Molander, Exploiting the sp^2 character of bicyclo[1.1.1]pentyl radicals in the transition-metal-free multi-component difunctionalization of [1.1.1]propellane. *Nat. Chem.* **14**, 1068–1077 (2022).

19. J. H. Kim, A. Ruffoni, Y. S. S. Al-Faiyz, N. S. Sheikh, D. Leonori, Divergent strain-release amino-functionalization of [1.1.1]propellane with electrophilic nitrogen-radicals. *Angew. Chem. Int. Ed.* **59**, 8225–8231 (2020).

20. X. Dang, Z. Li, J. Shang, C. Zhang, C. Wang, Photoinduced C(sp^3)−H bicyclopentylation enabled by an electron donor–acceptor complex-mediated chemoselective three-component radical relay. *Angew. Chem. Int. Ed.* **63**, e202400494 (2024).

21. Y. Wu, X. Wang, Y. Han, X. Chen, R. Zhang, H. Qu, C. Chen, Nickel-catalyzed radical relay enabled chemoselective fluoroalkyl-borylation of [1.1.1]propellane under kinetic control. *Sci. Adv.* **12**, eaea8588 (2026).

22. L. Yi, D. Kong, A. P. Kale, R. Alshehri, H. Yue, A. Gizatullin, B. Maity, R. Kancherla, L. Cavallo, M. Rueping, Halogen bonding initiated difunctionalization of [1.1.1]propellane via photoinduced polarity match additions. *Angew. Chem. Int. Ed.* **63**, e202411961 (2024).

23. L. Kim, W. Lee, S. Hong, Insight into C4 selectivity in the light-driven C−H fluoroalkylation of pyridines and quinolines. *Angew. Chem. Int. Ed.* **63**, e202410408 (2024).

24. J. Liu, R. Purushothaman, F. Hinrichs, M. Surke, S. Warratz, L. Ackermann, Synthesis of diverse gycosyl bicyclo[1.1.1]pentanes enabled by electrochemical functionalization of [1.1.1]propellane. *J. Am. Chem. Soc.* **147**, 34813–34822 (2025).

25. X. Liu, H. Chen, D. Yang, B. Hu, Y. Hu, S. Wang, Y. Lan, A. Lei, J. Li, Anion-tuning of organozincs steering cobalt-catalyzed radical relay couplings. *ACS Catal.* **13**, 9254–9263 (2023).

26. C. Shen, P. Zhang, Q. Sun, S. Bai, T. S. A. Hor, X. Liu, Recent advances in C–S bond formation *via* C–H bond functionalization and decarboxylation. *Chem. Soc. Rev.* **44**, 291–314 (2015).

27. I. P. Beletskaya, V. P. Ananikov, Transition-metal-catalyzed C–S, C–Se, and C–Te bond formations via cross-coupling and atom-economic addition reactions. Achievements and challenges. *Chem. Rev.* **122**, 16110–16293 (2022).

28. P. Devendar, G.-F. Yang, Sulfur-containing agrochemicals. *Top. Curr. Chem.* **375**, 82 (2017).

29. K. A. Scott, J. T. Njardarson, Analysis of US FDA-approved drugs containing sulfur atoms. *Top. Curr. Chem.* **376**, 5 (2018).

30. N. A. Meanwell, Improving drug design: An update on recent applications of efficiency metrics, strategies for replacing problematic elements, and compounds in nontraditional drug space. *Chem. Res. Toxicol.* **29**, 564–616 (2016).

31. R. M. Bär, S. Kirschner, M. Nieger, S. Bräse, Alkyl and aryl thiol addition to [1.1.1]propellane: Scope and limitations of a fast conjugation reaction. *Chem. A Eur. J.* **24**, 1373–1382 (2018).

32. H. D. Pickford, V. Ripenko, R. E. McNamee, S. Holovchuk, A. L. Thompson, R. C. Smith, P. K. Mykhailiuk, E. A. Anderson, Rapid and scalable halosulfonylation of strain-release reagents. *Angew. Chem. Int. Ed.* **62**, e202213508 (2023).

33. Z. Wu, Y. Xu, J. Liu, X. Wu, C. Zhu, A practical access to fluoroalkylthio(seleno)-functionalized bicyclo[1.1.1]pentanes. *Sci. China Chem.* **63**, 1025–1029 (2020).

34. H.-M. Huang, P. Bellotti, J. Ma, T. Dalton, F. Glorius, Bifunctional reagents in organic synthesis. *Nat. Rev. Chem.* **5**, 301–321 (2021).

35. J. Xu, J. Zhang, Z. Wang, J. Li, J. Shen, P. Zhang, Photocatalyzed site-selective formal β-C(sp^3)−H sulfonylation of ketones with sulfinates. *Org. Lett.* **28**, 2777–2782 (2026).

36. É. Lèbre, M. Stringer, K. Svobodova, J. R. Owen, D. Kemp, C. Côte, A. Arratia-Solar, R. K. Valenta, The social and environmental complexities of extracting energy transition metals. *Nat. Commun.* **11**, 4823 (2020).

37. X.-Q. Chu, D. Ge, Y.-Y. Cui, Z.-L. Shen, C.-J. Li, Desulfonylation via radical process: Recent developments in organic synthesis. *Chem. Rev.* **121**, 12548–12680 (2021).

38. F. Juliá, T. Constantin, D. Leonori, Applications of halogen atom transfer (XAT) for the generation of carbon radicals in synthetic photochemistry and pohotocatalysis. *Chem. Rev.* **122**, 2292–2352 (2022).

39. T. Constantin, M. Zanini, A. Regni, N. S. Sheikh, F. Juliá, D. Leonori, Aminoalkyl radicals as halogen-atom transfer agents for activation of alkyl and aryl halides. *Science* **367**, 1021–1026 (2020).

40. B. Górski, A.-L. Barthelemy, J. J. Douglas, F. Juliá, D. Leonori, Copper-catalysed amination of alkyl iodides enabled by halogen-atom transfer. *Nat. Catal.* **4**, 623–630 (2021).

41. A. Y. Chan, I. B. Perry, N. B. Bissonnette, B. F. Buksh, G. A. Edwards, L. I. Frye, O. L. Garry, M. N. Lavagnino, B. X. Li, Y. Liang, E. Mao, A. Millet, J. V. Oakley, N. L. Reed, H. A. Sakai, C. P. Seath, D. W. C. MacMillan, Metallaphotoredox: The merger of photoredox and transition metal catalysis. *Chem. Rev.* **122**, 1485–1542 (2022).

42. K. L. Skubi, T. R. Blum, T. P. Yoon, Dual catalysis strategies in photochemical synthesis. *Chem. Rev.* **116**, 10035–10074 (2016).

43. K. P. S. Cheung, S. Sarkar, V. Gevorgyan, Visible light-induced transition metal catalysis. *Chem. Rev.* **122**, 1543–1625 (2022).

44. X. Hu, C. Zheng, X. Song, B. Hasimujiang, M. Chen, Z. Ruan, Manganese-catalyzed electrochemical diazidation of dehydroalanine peptides. *Adv. Sci.* **12**, e2502711 (2025).

45. B. Sun, S. Zhou, J. Wang, X. Xu, X. Zhuang, W. Su, C. Jin, Hydrogen-bonding-driven site-selective activation of α-C(sp^3)-H in alcohols: A straightforward dual-catalysis strategy for efficient acceptorless dehydrogenation. *Angew. Chem. Int. Ed.* **64**, e20256022 (2025).

46. C. Wang, Z. Chen, J. Sun, L. Tong, W. Wang, S. Song, J. Li, Sulfonamide-directed site-selective functionalization of unactivated C(sp^3)-H enabled by photocatalytic sequential electron/proton transfer. *Nat. Commun.* **15**, 5087 (2024).

47. K. Yu, Q. Nie, Q. Chen, W. Liu, Manganese-catalyzed cyclopropanation of allylic alcohols with sulfones. *Nat. Commun.* **15**, 6798 (2024).

48. L. Lu, J. Luo, D. Milstein, Manganese(I)-pincer catalyzed α-alkylation of sulfones by alcohols. *ACS Catal.* **13**, 5949–5954 (2023).

49. L. Wang, J. M. Lear, S. M. Rafferty, S. C. Fosu, D. A. Nagib, Ketyl radical reactivity via atom transfer catalysis. *Science* **362**, 225–229 (2018).

50. S. M. Rafferty, J. E. Rutherford, L. Zhang, L. Wang, D. A. Nagib, Cross-selective aza-pinacol coupling via atom transfer catalysis. *J. Am. Chem. Soc.* **143**, 5622–5628 (2021).

51. R.-Z. Liu, J. Li, J. Sun, X.-G. Liu, S. Qu, P. Li, B. Zhang, Generation and reactivity of amidyl radicals: Manganese-mediated atom-transfer reaction. *Angew. Chem. Int. Ed.* **59**, 4428–4433 (2020).

52. J. Han, J. Han, S. Chen, T. Zhong, Y. He, X. Yang, G. Wang, C. Zhu, J. Xie, Photoinduced manganese-catalysed hydrofluorocarbofunctionalization of alkenes. *Nat. Synth.* **1**, 475–486 (2022).

53. Y. Hwang, H. Jung, E. Lee, D. Kim, S. Chang, Quantitative analysis on two-point ligand modulation of iridium catalysts for chemodivergent C–H amidation. *J. Am. Chem. Soc.* **142**, 8880–8889 (2020).

54. Z. Wang, L. Hu, N. Chekshin, Z. Zhuang, S. Qian, J. X. Qiao, J.-Q. Yu, Ligand-controlled divergent dehydrogenative reactions of carboxylic acids via C–H activation. *Science* **374**, 1281–1285 (2021).

55. Y.-H. Wang, L.-H. He, Y.-Y. Tan, Z.-T. Zhang, Y.-D. Xu, R. Ding, J. Jiang, W.-M. He, Dual semiconductor-photoredox/halogen-bonding organocatalyzed cascade sulfonylation/cyclization of alkynes and alkenes with RSO_2Cl. *Adv. Sci.* **13**, e15993 (2026).

56. H. Wang, H. Shao, A. Das, S. Dutta, H. T. Chan, C. Daniliuc, K. N. Houk, F. Glorius, Dearomative ring expansion of thiophenes by bicyclobutane insertion. *Science* **381**, 75–81 (2023).

57. H. Wang, J. E. Erchinger, M. Lenz, S. Dutta, C. G. Daniliuc, F. Glorius, *Syn*-selective difunctionalization of bicyclobutanes enabled by photoredox-mediated C–S σ-bond scission. *J. Am. Chem. Soc.* **145**, 23771–23780 (2023).

58. Y. Guo, J. Zhu, Y. Wang, Y. Li, H. Hu, P. Zhang, J. Xu, W. Li, General and modular route to (halo)alkyl BCP-Heteroaryls enabled by α-aminoalkyl radical-mediated halogen-atom transfer. *ACS Catal.* **14**, 619–627 (2024).

59. J. D. Lasso, D. J. Castillo-Pazos, J. M. Salgado, C. Ruchlin, L. Lefebvre, D. Farajat, D. F. Perepichka, C.-J. Li, A general platform for visible light sulfonylation reactions enabled by catalytic triarylamine EDA complexes. *J. Am. Chem. Soc.* **146**, 2583–2592 (2024).

60. J. Xu, J. Cao, X. Wu, H. Wang, X. Yang, X. Tang, R. W. Toh, R. Zhou, E. K. L. Yeow, J. Wu, Unveiling extreme photoreduction potentials of donor–acceptor cyanoarenes to access aryl radicals from aryl chlorides. *J. Am. Chem. Soc.* **143**, 13266–13273 (2021).

61. J. M. Crance, N. Scaramozzino, A. Jouan, D. Garin, Interferon, ribavirin, 6-azauridine and glycyrrhizin: Antiviral compounds active against pathogenic flaviviruses. *Antiviral Res.* **58**, 73–79 (2003).

62. O. Vanparijs, R. Marsboom, L. Desplenter, Diclazuril, a new broad spectrum anticoccidial drug in chickens. 1. Dose titration studies and pilot floor pen trials. *Poult. Sci.* **68**, 489–495 (1989).

63. W. Dong, S. Keess, G. A. Molander, Nickel-mediated alkyl-, acyl-, and sulfonylcyanation of [1.1.1]propellane. *Chem Catal.* **3**, 100608 (2023).

64. Y. Cao, C. Huang, Q. Lu, Photoelectrochemically driven iron-catalysed C(sp^3)−H borylation of alkanes. *Nat. Synth.* **3**, 537–544 (2024).

65. S. Wang, X. Luo, Y. Wang, Z. Liu, Y. Yu, X. Wang, D. Ren, P. Wang, Y.-H. Chen, X. Qi, H. Yi, A. Lei, Radical-triggered translocation of C–C double bond and functional group. *Nat. Chem.* **16**, 1621–1629 (2024).

66. J. Zhu, Y. Hong, Y. Wang, Y. Guo, Y. Zhang, Z. Ni, W. Li, J. Xu, Synthesis of 1-(halo)alkyl-3-heteroaryl bicyclo[1.1.1]pentanes enabled by a photocatalytic Minisci-type multicomponent reaction. *ACS Catal.* **14**, 6247–6258 (2024).

67. P. Ghosh, N. Y. Kwon, S. Kim, S. Han, S. H. Lee, W. An, N. K. Mishra, S. B. Han, I. S. Kim, C-H methylation of iminoamido heterocycles with sulfur ylides. *Angew. Chem. Int. Ed.* **60**, 191–196 (2021).

68. J. Zhou, Q. Ren, N. Xu, C. Wang, S. Song, Z. Chen, J. Li, $K_2S_2O_8$-catalyzed highly regioselective amidoalkylation of diverse N-heteroaromatics in water under visible light irradiation. *Green Chem.* **23**, 5753–5758 (2021).

69. J. H. Ye, P. Bellotti, T. O. Paulisch, C. G. Daniliuc, F. Glorius, Visible-light-induced cycloaddition of α-ketoacylsilanes with Imines: Facile aaccess to β-lactams. *Angew. Chem. Int. Ed.* **60**, 13671–13676 (2021).

70. J. Zhu, Y. Guo, Y. Zhang, W. Li, P. Zhang, J. Xu, Visible-light-induced direct perfluoroalkylation/heteroarylation of [1.1.1]propellane to diverse bicyclo[1.1.1]pentanes (BCPs) under metal and photocatalyst-free conditions. *Green Chem.* **25**, 986–992 (2023).

71. W.-L. Dong, G.-Y. Huang, Z.-M. Li, W.-G. Zhao, A convenient method for the aerobic oxidation of thiols to disulfides. *Phosphorus Sulfur Silicon Relat. Elem.* **184**, 2058–2065 (2009).

72. S. Madabhushi, R. Jillella, V. Sriramoju, R. Singh, Oxyhalogenation of thiols and disulfides into sulfonyl chlorides/bromides using oxone-KX (X = Cl or Br) in water. *Green Chem.* **16**, 3125–3131 (2014).

73. M. R. Mutra, J. Li, Y.-T. Chen, J.-J. Wang, Time and atom economical regio- and chemoselective radical cyclization of unactivated 1,6-enynes under metal- and oxidant-free conditions. *Chem. A Eur. J.* **28**, e202200742 (2022).
